# Supplementary material for: Nurse-led home-visitation programme for first-time mothers in reducing maltreatment and improving child health and development (BB:2-6): longer-term outcomes from a randomised cohort using data linkage
Source: BMJ Open. 2022 Feb 10;12(2):e049960. doi: 10.1136/bmjopen-2021-049960 (PMC8845181; doi:10.1136/bmjopen-2021-049960)
Supplement: Supplementary data [file bmjopen-2021-049960supp001.pdf]

Supplementary appendix

I. Number of valid FNP visits received
- Table S1 BB:2-6 study population data: Valid FNP visits per phase

II. Study assessment domain, outcomes, principal data providers and data obtained
- Table S2 BB:2-6 Study assessment domains, key and exploratory outcomes and principal data providers

III. Data accrued from providers
- Table S3 Numbers (%) of mothers and children with data returned by dataset

IV. Further details of Child in Need outcome

V. Representativeness of study sample
- Table S4 Maternal characteristics of those in BB:0-2 compared to those in the BB:2-6
- Table S5 Child characteristics of those in the BB:0-2 study compared to those in the BB:2-6 study

VI. Descriptive secondary outcomes and additional Key Stage 1 results

- Table S6 Child In Need referrals, Children's Social Services referrals and hospital admissions: descriptives
- Table S7 Emergency attendance to hospital by injury or ingestion, by trial arm
- Table S8 Admissions by injury/ingestion type and trial arm
- Table S9 Hospital admissions for injuries and ingestions by trial arm and broken down by age categories
- Table S10 Child health, developmental and educational: descriptives
- Table S11 Children reaching the expected standard in KS1 assessments by trial arm and academic year
- Figure S1 Distribution of children's month of birth by trial arm
- Table S12 Comparison of parameter estimates before and after adjustment for child's month of birth

VII. Results of planned sub-group analysis
- Table S13 Subgroup analyses for referrals to Children's social care services
- Table S14 Subgroup analyses for child in need status
- Table S15 Predefined subgroup analyses on Early Years Assessment total point score
- Table S16 Subgroup analyses on KS1 assessments reaching the expected standard (RES)
- Table S17 Subgroup analyses on reaching the expected standard in the writing KS1 assessment for 2016/17 academic year

VIII. Treatment efficacy

IX. Exploration of surveillance bias upon Child In Need outcome assessment

- Table S18 Baseline maternal characteristics, school readiness outcomes for children referred to CSC and classed as a Child In Need, before the age of 4 years old by intervention arm.

X. Missing data

XI. Cost Consequence Analysis

Robling M, *et al. BMJ Open* 2022; 12:e049960. doi: 10.1136/bmjopen-2021-049960

- Table S19 Cost-consequence balance sheet for the family-nurse based intervention for the four years between children's ages 2 and 6.

XII. Postpartum resource use data collected for SPHN contacts

I. Number of valid FNP visits received

Of the 766 women in the FNP arm in the BB:2-6 cohort, 69 did not enrol for FNP. For the remaining 697 who were randomised and enrolled, 695 had received at least one valid visit and two did not receive any valid visits across the three phases (pregnancy, infancy and toddlerhood). The descriptives in Table S1 are based on 697 women. A greater proportion of women received no visits during the infancy and toddler phases (40 (5.7%) and 98 (14.1%) respectively) compared to the pregnancy phase (6 (0.7%)). This is similar to that reported in the BB:0-2 cohort with comparable median valid visits and proportions reaching the fidelity goals (see Lancet Supplementary appendix Table S11.3).[1]

Table S1 BB:2-6 study population data: Valid FNP visits per phase

	**Pregnancy**	**Infancy**	**Toddlerhood**
N women randomised and enrolled to FNP	697	697	697
Women not receiving any valid visits n(%)	6 (0·7)	40 (5·7)	98 (14·1)
Women receiving at least one valid visit n(%)	691 (99·3)	657 (94·3)	599 (85·9)
Valid visits received			
Median (25th to 75th centile)	10 (8 to 12)	19 (15 to 22)	13 (10 to 16)
Mean (SD)	9·74 (3·42)	18·7 (5·97)	13·28 (5·46)
Reaching fidelity goal of: n(%)			
80%+	401/697 (57·5)	-	-
65%+	-	364/697 (52·2)	-
60%+	-	-	297/697 (42·6)

[1] Robling M, Bekkers MJ, Bell K, Butler CC, Cannings-John R, Channon S, et al. Effectiveness of a nurse-led intensive home-visitation programme for first-time teenage mothers (Building Blocks): A pragmatic randomised controlled trial. Lancet. 2016;387:146–55. [accessed 2019 Nov 12]. Available from: http://www.thelancet.com/journals/lancet/article/PIIS0140-6736(15)00392-X/fulltext

II. Study assessment domain, outcomes and principal data providers

Table S2 BB:2-6 Study assessment domains, key and exploratory outcomes and principal data providers

Domains	Outcomes	Maternal	Child	HES	ONS	NPD	Data source	Analysis
Primary: Child in Need status recorded at any time during the follow-up period.	CIN status		✓			✓	CIN	LO
	Exploratory:							
	Age at first CIN referral		✓			✓	CIN	*SURV*
	Number of CIN referrals per child		✓			✓	CIN	*PO*
Secondary: (i) Objective measures of maltreatment	CIN categorisation		✓			✓	CIN	MNOM
	CIN duration		✓			✓	CIN	SURV
	Referral to Children's social care services		✓			✓	CIN	LO
	Exploratory: Age at first referral		✓			✓	CIN	SURV
	Child Protection Plan		✓			✓	CIN	LO
	Child Protection Plan categorisation		✓			✓	CIN	MNOM
	Looked after status		✓			✓	CLA	LO
	CLA period of care		✓			✓	CLA	PO
	Legal status of CLA		✓			✓	CLA	D
	Cause of death		✓		✓		ONS- deaths	D
(ii) Associated measures of maltreatment	Injuries and ingestions:							
	Emergency attendance		✓	✓			A&E	LO
	Admission to hospital		✓	✓			IP	LO
	Attendance and/or admission		✓	✓			A&E, IP	LO
	DNA appointments		✓	✓			OP	LO
	Exploratory:							

Robling M, *et al. BMJ Open* 2022; 12:e049960. doi: 10.1136/bmjopen-2021-049960

**Domains**	**Outcomes**	**Maternal**	**Child**	**HES**	**ONS**	**NPD**	**Data source**	**Analysis**
	Number of admissions per child		✓	✓			IP	PO
	Length of hospital admission stay		✓	✓			IP	D
(iii) Maternal outcomes	Subsequent pregnancy	✓		✓			OP, IP, Ab	LO
	Exploratory:							
	Number of births	✓		✓			IP	PO
	Inter-birth interval between first and second child	✓		✓			IP	SURV
(iv) Costs	Health and Social Care resource use	✓	✓	✓		✓	OP, IP, A&E matched with NHS reference costs	D
(v) Child health, developmental and educational outcomes	Special educational needs		✓				PLASC, AP, PRU Census, EYC	LO
	provision		✓	✓		✓		
	Disability		✓			✓	NA	NA
	Early education attendance		✓			✓	PLASC, EYC	LO
	School attendance		✓			✓	Absence	LO
	Early Years assessment		✓			✓	EYFSP	LIN, LO
	KS1 attainment					✓	KS1	LO
	Exploratory:							
	Early education attendance by School or an Ofsted registered PVI establishment		✓			✓	PLASC, EYC	D
	Earliest type of day care		✓			✓	EYC	D

Ab - Abortions; A&E – Accident and Emergency; AP – Alternative Provision; CIN – Child in Need; CLA – Child Looked After; D – Descriptive; DNA – Did not attend; EYC – Early Years Census; EYFSP –Early years foundation stage profile; FNP – Family Nurse Partnership; HES – Hospital Episode Statistics; IP - Inpatients; KS1 – Key Stage 1; LIN – Linear regression; LO – Logistic; MNOM - multinomial NA - Not available; NPD – National Pupil Database; ONS – Office for National Statistics; OP – Outpatients; PLASC – Pupil Level and School Census; PO-Poisson; PRU – Pupil Referral Unit Census.; PVI – Private, Voluntary or Independent (PVI) establishment; SURV – Survival.

III. Data accrued from providers

Table S3 Numbers (%) of mothers and children with data returned by dataset

	Mothers	Children
**Sent for matching to DfE and NHSD**	**N=1537**	**N=1547**
**Department for Education (DfE)**		
Not matched	1248 (81·2)	40 (2·6)
Match to any DfE dataset	289 (18·8)	1507 (97·4)
Pupil Level Annual School Census (PLASC)	<5	1498 (96·8)
Child in Need (CIN)	278 (18·1)	414 (26·8)
Children looked after (CLA)	61 (4·0)	52 (3·4)
Early years foundation stage profile (EYFSP)	N/A	1476 (95·4)
Key Stage one (KS1)	N/A	1472 (95·2)
Early Years Census (EYC)	N/A	642 (41·5)
Pupil referral unit (PRU) Census	<5	0
Alternative Provision (AP)	<5	<5
Absence	<5	1494 (96·6)
**NHS Digital (NHSD)**		
Not matched	36 (2·3)	27 (1·7)
Duplicate match	0	1 (0·06)
Matched to any NHSD dataset	1501 (97·7)	1519 (98·3)
Accident and Emergency (A&E)	1360 (88·5)	1392 (90·0)
Admitted Patient Care (Inpatients) (APC)	1498 (97·5)	1507 (97·4)
Outpatient (OP)	1495 (97·3)	1189 (76·9)
**Sent for matching to Department of Health and Social Care (DHSC)**	**N=1532**	**0**
Abortions dataset	1532 (100)	NA

IV. Further details of Child in Need outcome.

In our primary comparative analysis we examined whether the firstborn ('BB:0-2 child') had ever been referred to social services and classed as a child in need at any point between birth and 6 years of age. There is no flag in the Child In Need dataset to identify whether a child is in need after they have been referred to social services. Therefore, CIN was defined (as per DfE methodology)[1] as all children referred to social services, excluding cases where:

- the referral is flagged as being a referral resulting in no further action; or
- the only activity recorded is an assessment and the reason for the closure of the case being that case was closed after assessment with no further action.

---

1. Department for Education. Characteristics of children in need in England: 2014 to 2015. 2015. [accessed 2019 Nov 12]. Available from: https://www.gov.uk/government/uploads/system/uploads/attachment_data/file/469740/SFR41-2015_Methodology.pdf

V. Representativeness of study sample

Scope and purpose: We assessed whether the study sample for BB:2-6 were similar to women recruited to the original BB:0-2 trial.

Conclusion: The BB:2-6 and BB:0-2 cohorts are similar in terms of maternal (Table S4) and baby characteristics (Table S5).

Table S4 Maternal characteristics of those in BB:0-2 compared to those in the BB:2-6

	**BB:0-2 N=1618**	**BB:2-6 N=1537**
**Minimisation variables at randomisation**		
**Gestational age at baseline** n(%)		
16 weeks or more	658 (40·7)	636 (41·4)
Less than 16 weeks	959 (59·3)	900 (58·6)
**Smoking status** n(%)		
No	860 (53·2)	822 (53·5)
Yes	758 (46·8)	715 (46·5)
**Language** n(%)		
English	1611 (99·6)	1530 (99·5)
Other	6 (0·4)	6 (0·4)
Missing	*1 (0·0)*	*0 (0·0)*

	BB:0-2 N=1618	BB:2-6 N=1537
**Trial arm** n(%)		
FNP	808 (49·9)	766 (49·8)
Usual care	810 (50·1)	771 (50·2)
**Age at recruitment (years) Median (25th to 75th centile)**	17·9 (16·9 to 18·8)	17·9 (16·9 to 18·8)
**Ethnicity** n(%)		
White background	1425 (88·1)	1355 (88·2)
Mixed background	89 (5·5)	82 (5·3)
Asian background	27 (1·7)	25 (1·6)
Black background	71 (4·4)	69 (4·5)
Chinese or Other background	6 (0·4)	6 (0·4)
**Relationship status with baby's father** n(%)		
Married	20 (1·2)	16 (1·0)
Separated	165 (10·2)	150 (10·0)
Closely involved/boyfriend	1222 (75·5)	1168 (76·0)
Just friends	211 (13·0)	203 (13·2)
**Live with father of baby** n(%)		
Yes	368 (22·7)	348 (22·6)
No	1112 (68·7)	1061 (69·0)
Missing	138 (8·5)	128 (8·3)
**NEET status**[a] n(%)		
Yes	663 (41·0)	634 (41·2)
No	717 (44·3)	678 (44·1)
Missing	*4 (0·2)*	*4 (0·3)*
Not applicable (aged 16 years or younger)	*234 (14·5)*	*221 (14·4)*
**Socio-economic status: Overall Index of Multiple Deprivation (IMD) Score**[b] Median (25th to 75th centile)	N=1606 38·2 (25·2 to 52·0)	N=1525 38·1 (25·0 to 52·1)
**Maternal health and wellbeing**		
**Generalized self-efficacy scale (score 10 to 40)**[c] Median (25th to 75th centile)	N=1592 30·0 (28·0 to 33·0)	N=1533 30·0 (28·0 to 33·0)
**Adaptive functioning: Difficulty in at least one basic skill** n(%)		
Yes	430 (26·6)	397 (25·8)
No	1185 (73·2)	1138 (74·0)
Missing	*3 (0·2)*	*2 (0·1)*
**Adaptive functioning: Had 3 or less life skills** n(%)		
Yes	434 (26·8)	403 (26·2)
No	1178 (72·8)	1129 (73·5)
Missing	*6 (0·4)*	*5 (0·3)*
**Adaptive functioning: At least one burden** n(%)		
Yes	476 (29·4)	451 (29·3)
No	1131 (69·9)	1075 (69·9)
Missing	*11 (0·7)*	*11 (0·7)*
**Smoking** (Participant self-reported) n(%)		
Ever smoked: Yes	1291 (79·8)	1227 (79·8)
No	327 (20·0)	310 (20·2)
Missing	*0 (0)*	*0 (0)*
**Child characteristics**	**BB:0-2 sample N=1510**	**BB:2-6 sample N=1547**
**Trial arm** n(%)		

	BB:0-2 N=1618	BB:2-6 N=1537
FNP	742 (49·1)	773 (50·0)
Usual Care	768 (50·9)	774 (50·0)
**Gender** n(%)		
Male	777 (51·5)	787 (50·9)
Female	732 (48·5)	758 (40·1)
Missing	1	72[d]
**Birthweight (grams) Mean (sd)**	3207·3 (599·6)	3219·6 (580·7)
Missing[c]		71
**Neonatal unit (NNU) admission** n(%)		
Yes	149 (10·0)	142 (9·2)
Missing from data collection	13	11
Missing	Not applicable	71[d]

[a] Definition of NEET status: Not in education employment or training (applicable only to those whose age at end of previous academic year at time of baseline interview was >16)
[b] Higher IMD score indicated more deprivation. Mean IMD Score for England in 2010 was 21·67 [60];
[c] Higher score indicates higher level of self-efficacy
[d] Mothers were withdrawn in BB:0-2 but are included in BB:2-6

Table S5 Child characteristics of those in the BB:0-2 study compared to those in the BB:2-6 study

	BB:0-2 sample N=1510	BB:2-6 sample N=1547
**Trial arm** n(%)		
FNP	742 (49·1)	773 (50·0)
Usual Care	768 (50·9)	774 (50·0)
**Gender** n(%)		
Male	777 (51·5)	787 (50·9)
Female	732 (48·5)	758 (40·1)
Missing	1	72[a]
**Birthweight (grams) Mean (sd)**	3207·3 (599·6)	3219·6 (580·7)
Missing[a]		71
**Neonatal unit (NNU) admission** n(%)		
Yes	149 (10·0)	142 (9·2)
Missing from data collection	13	11
Missing[a]	Not applicable	71*

[a] Mothers were withdrawn in BB:0-2 but are included in BB:2-6

Robling M, *et al. BMJ Open* 2022; 12:e049960. doi: 10.1136/bmjopen-2021-049960

VI. Descriptive secondary outcomes and additional Key Stage 1 results

Table S6 Child In Need referrals, Children's Social Services referrals and hospital admissions: descriptives

Outcome	FNP	Usual care (UC)
**Child in Need (CIN) Referrals**	***N=161***	***N=162***
Age at first CIN referral (days)		
Pre birth to < 1year	12 (7.5%)	5 (3.1%)
1 to <2 years	6 (3·7%)	6 (3·7%)
2 to <3 years	15 (9·4%)	28 (17·3%)
3 to <4 years	27 (16·9%)	23 (14·2%)
4 to <5 years	47 (29·4%)	39 (24·1%)
5 to <6 years	33 (20·6%)	41 (25·3%)
6 to <7 years	20 (12·5%)	20 (12·3%)
Missing	*1*	*0*
Unique* number of CIN referrals per child		
1 case	110 (68·3%)	110 (67·9%)
2 cases	42 (26·1%)	42 (25·9%)
3 or more cases	9 (5·5%)	10 (6·1%)
CIN cases closed at 31st March 2017	116 (72·0%)	110 (67·9%)
**Referral to Children's social care services (CSCS)**	***N=760***	***N=746***
Total referrals made N	339	354
Ratio referrals:children	1·65	1·70
Referrals made per child	***N=206***	***N=208***
1	159 (77·2%)	140 (67·3%)
2	34 (16·5%)	51 (24·5%)
3 to 5	13 (6·3%)	27 (8·2%)
**Unit=referral (referred to CSCS encompass any referral)**	***n=339***	***n=354***
Source of referral		
Individual	29 (8·8%)	34 (10·0%)
School/Education	150 (45·7%)	136 (40·0%)
Health services	114 (34·8%)	132 (38·8%)
Local Authority Services	35 (10·7%)	38 (11·2%)
Missing	*11*	*14*
Primary need of referrals		
Abuse or neglect	198 (58·9%)	205 (57·9%)
Family dysfunction	72 (21·4%)	88 (24·9%)
Family in acute stress	19 (5·7%)	21 (5·9%)
Low income/absent parenting/ socially unacceptable behaviour	11 (3·3%)	7 (2·0%)
Parental/Childs disability illness	14 (4·2%)	11 (3·1%)
Cases other than Child in Need	11 (2·3%)	12 (3·4%)
Not stated	11 (3·3%)	9 (2·5%)
Missing	*3*	*1*
Legal status of child looked after†	***N=17***	***N=23***
Interim care order	6 (35·3%)	8 (34·8%)
Full care order	5 (29·4%)	6 (26·1%)

**Outcome**	**FNP**	**Usual care (UC)**
Placement order granted	<5	<5
Single period of accommodation under section 20	<5	<5
**Injuries and Ingestions: Admission to hospital**	**N=760**	**N=759**
N unique admissions	109	119
Ratio admissions: children	1.22	1.20
Length of hospital stay (days)	0·5‡ (0·5 to 1·0)	0·5 (0·5 to 1·0)
	**N=760**	**N=746**
Death	<10	

Data are n (%), n/N (%), or median (25th to 75th centile) unless otherwise stated. <5 = numbers suppressed· FNP = Family Nurse Partnership Programme + Usual Care. * A unique referral is counted as a distinct referral date per child. † Legal status for most recent CLA episode (Of n=52 children with a recent CLA episode, n=12 cases have a missing status). ‡ 0·5 days indicates an admission and discharge on the same day (could be up to 1 day in hospital). Source: National Pupil Database, The Department for Education

Table S7 Emergency attendance to hospital by injury or ingestion, by trial arm

	**Trial arm, n (%)**	
**Injury / ingestion**	**FNP (N = 911)**	**Usual Care (N = 881)**
Laceration	136 (14.9)	158 (17.9)
Contusion/abrasion	90 (9.9)	96 (10.9)
Soft-tissue inflammation	63 (6.9)	64 (7.3)
Head injury	68 (29.4)	249 (28.3)
Dislocation	109 (12.0)	84 (9.5)
Sprain/muscle/tendon injury	35 (3.8)	33 (3.8)
Nerve/vascular injury	74 (8.1)	60 (6.8)
Burns and scalds/electric shock	48 (5.3)	37 (4.2)
Foreign body	40 (4.4) 4	6 (5.2)
Bites and stings	12 (1.3)	20 (2.3)
Poisoning	36 (4.0)	34 (3.9)

Table S8 Admissions by injury/ingestion type and trial arm: For children of all ages (0-6 years) and for children aged 0-2 years old

	**Trial arm, n (%)**			
	**All children**		**Children aged 0-2 years***	
**Diagnosis ICD-10 code**	**FNP (n=109)**	**Usual care (n=119)**	**FNP (n=42)**	**Usual Care (n=63)**
S00–S09 Injuries to the head	52 (47.7)	61 (51.3)	39 (61.9)	27 (64.3)
S20–S29 Injuries to the thorax	9 (8.3)	6 (5.0)		
S30–S39 Injuries to the abdomen, lower back, lumbar spine and pelvis				
S40–S49 Injuries to the shoulder and upper arm			7 (16.7)	7 (11.1)
S50–S59 Injuries to the elbow and forearm				
S60–S69 Injuries to the wrist and hand	8 (7.3)	5 (4.2)		
S70–S79 Injuries to the hip and thigh	11 (10.1)	11 (9.2)		
S80–S89 Injuries to the knee and lower leg				
S90–S99 Injuries to the ankle and foot				
T00–T07 Injuries involving multiple body regions	5 (4.6)	10 (8.4)	15 (35.7)	7 (11.1)
T08–T14 Injuries to unspecified part of trunk, limb or body region				
T20–T32 Burns and corrosions				
T15–T19 Effects of foreign body entering through natural orifice	5 (4.6)	8 (6.7)		
T36–T50 Poisoning by drugs, medicaments and biological substances	11 (10.1)	8 (6.7)		
T51–T65 Toxic effects of substances chiefly non-medicinal as to source (sting, alcohol, solvents etc.)	8 (7.3)	10 (8.4)		
T66–T78 Other and unspecified effects of external causes				

* We undertook further exploratory analyses of length of stay following admission for:
(i) children aged under 1 year old: The median (25th to 75th quartile) length of stay (in days) was, for the n=20 children in FNP group 0.75 (0.5 to 2) and for the n=29 children in the UC group 0.5 (0.5 to 2) with no difference observed (Incidence rate ratio (IRR) 0.68, 95% CI 0.24 to 1.98, Ref = UC).
(ii) children aged between 1 and under 2 years old: The median (25th to 75th centile) length of stay (in days) was, for the n=21 admissions in FNP group, 0.5 (0.5 to 1) and for the n=34 admissions in UC group 0.5 (0.5 to 1) with no observed difference (IRR 0.36, 95% CI 0.05 to 2.43, Ref = UC).

Table S9. Hospital admissions for injuries and ingestions by trial arm and broken down by age categories

	FNP N=760	Usual care N=759
No hospital admissions with an injury or an ingestion (0-6 years)	671 (88.3%)	660 (87.0%)
At least one hospital admission with an injury or an ingestion	89 (11.7%)	99 (13.0%)
By age at admission:		
**Under 1 year**		
Children with no admission n(%)	744 (97.9%)	732 (96.4%)
Children with at least one admission n(%)	16 (2.1%)	27 (3.6%)
95% CI	1.3 to 3.3%	2.5 to 5.1%
Total number of admissions (different days)	20	29
Ratio of admissions: child	1.25	1.08
	n=20	n=29
Length of stay (days) Median (25th to 75th centiles)	0.75 (0.5 to 2)	0.5 (0.5 to 2)
Same day discharge (admission = discharge date)	10 (50%)	15 (51.7%)
Discharge after 1 day or more	10 (50%)	14 (48.3%)
**1 to under 2 years**		
Children with no admission n(%)	738 (97.1%)	725 (95.5%)
Children with at least one admission n(%)	21 (2.8%)	31 (4.1%)
95% CI	1.8 to 4.2%	2.9 to 5.7%
Total number of admissions (different days)	22	34
Ratio of admissions: child	1.05	1.10
	n=21*	n=34
Length of stay (days) Median (25th to 75th centiles)	0.5 (0.5 to 1)	0.5 (0.5 to 1)
Same day discharge (admission = discharge date)	14 (66.7%)	23 (67.6%)
Discharge after 1 day or more	7 (33.3%)	11 (32.4%)
**25 months to under 6 years**		
Children with no admission n(%)	699 (92.0%)	709 (93.4%)
Children with at least one admission n(%)	61 (8.0%)	50 (6.6%)
95% CI	6.3 to 10.2%	5.0 to 8.6%
Total number of admissions (different days)	67	56
Ratio of admissions: child	1.10	1.12
	n=65*	n=56
Length of stay (days) Median (25th to 75th centiles)	0.5 (0.5 to 1)	0.5 (0.5 to 1)
Same day discharge (admission = discharge date)	38 (58.4%)	29 (51.8%)
Discharge after 1 day	18 (27.6%)	17 (30.4%)
Discharge after 2 days or more	11 (16.9%)	10 (17.9%)

* Date of discharge missing – LOS cannot be calculated

LOS=0.5 days indicates an admission and discharge on the same day (could be up to 1 day in hospital).

Table S10 Child health, developmental and educational: descriptives

**Outcome**	**FNP**	**Usual care**	**Absolute risk difference (FNP-Usual care) (95% CI)**
**Early educational attendance**	**N=759**	**N=747**	
Children attending education* up to the age of 4 years			
Yes†	758 (99·7%)	747 (100·0%)	-0·3% (-1·0 to 0·3%)
Children attending school up to the age of:			
• 4 years†	753 (99·1%)	744 (99·6%)	-0·5% (-1·5 to 0·4%)
• 3 years	479 (63·0%)	482 (64·5%)	
• 2 years	137 (18·0%)	147 (19·7%)	
Earliest type of day care	***N=334***	***N=308***	
Private	217 (65·0%)	221 (71·8%)	
Voluntary	52 (15·6%)	57 (18·5%)	
Local Authority Day Nursery	36 (10·8%)	22 (7·1%)	
Other‡	29 (8·7%)	8 (2·6%)	
**School attendance**	**N=754**	**N=740**	
Number of academic sessions§	630 (264 to 638)	630 (262 to 638)	
Overall absence rate Median (25th to 75th centiles)	5·08 (2·69 to 8·73)	5·19 (2·73 to 8·41)	
Overall authorised absence rate Median (25th to 75th centiles)	3·50 (1·96 to 6·09)	3·79 (2·02 to 6·11)	
Overall unauthorised absence rate Median (25th to 75th centiles)	1·84 (0·76 to 4·34)	1·57 (0·63 to 3·88)	
**Early Years assessment**	**N=743**	**N=728**	
Achieving good level of development (GLD) ‖:			
• Communication and Language	642 (86·4%)	613 (84·2%)	2·2% (-1·4 to 5·8%)
• Physical Development	676 (91·0%)	643 (88·3%)	2·7% (-0·5 to 5·8%)

Outcome	FNP	Usual care	Absolute risk difference (FNP-Usual care) (95% CI)
• Personal, Social and Emotional Development	668 (89·9%)	634 (87·1%)	2·8% (-0·4 to 6·1%)
• Literacy	493 (66·4%)	458 (62·9%)	3·4% (-1·4 to 8·3%)
• Maths	545 (73·4%)	516 (70·9%)	2·5% (-2·1 to 7·0%)
Total point score¶			
• Score <34	325 (43·8%)	345 (47·4%)	
• Score = 34	187 (25·2%)	183 (25·1%)	
• Score >34	231 (31·1%)	200 (27·5%)	
**Key Stage 1 assessments**			
Writing academic year 2017/18	**N=242**	**N=245**	
Lower than expected	82 (33·9%)	81 (33·1%)	
Reaching at least the expected standard**	160 (66·1%)	164 (66·9%)	-0·8 (-9·2 to 7·5)
Expected standard	140 (57·9%)	132 (53·9%)	4·0 (-4·8 to 12·7)
Higher standard	20 (8·3%)	32 (13·1%)	-4·8 (-10·4 to 0·7)

Data are n (%), n/N (%), or median (25th to 75th centile) unless otherwise stated. FNP = Family Nurse Partnership Programme + Usual Care.

* School or an Ofsted registered Private, Voluntary or Independent (PVI) establishment;

† = Not estimable; ‡ Childminding Network, Childminder, Registered Independent School, Other; § Total number of sessions over the academic years available (2013 to Spring 2017) and all 6 terms per academic year. ‖ Children achieving a good level of development are those achieving at least the expected level within the prime and specific areas of learning; ¶ Total point score ranges from 17 to 51 with a higher score indicating a better level of development. ** Working at the expected standard and at a greater depth within the expected standard. PLASC (Autumn, Spring and Summer terms), AP, PRU Census, and EYC. If for any of the years no response was recorded then the assumption was that the child was not included in the denominator as either not present on Census day or not in school. Source: National Pupil Database, The Department for Education

Table S11 Children reaching the expected standard in KS1 assessments by trial arm and academic year

		Academic year 2016/17	Academic year 2017/18
Reading	FNP	302/498 (60.6%)	181/242 (74.8%)
	Usual care	263/487 (54.0%)	180/245 (73.5%)
Maths	FNP	280/498 (56.2%)	179/242 (74.0%)
	Usual care	263/487 (54.0%)	186/245 (75.9%)
Science	FNP	341/498 (68.5%)	196/242 (81.0%)
	Usual care	310/487 (63.7%)	203/245 (82.9%)

Source: National Pupil Database, The Department for Education

Figure S1 Distribution of children's month of birth by trial arm

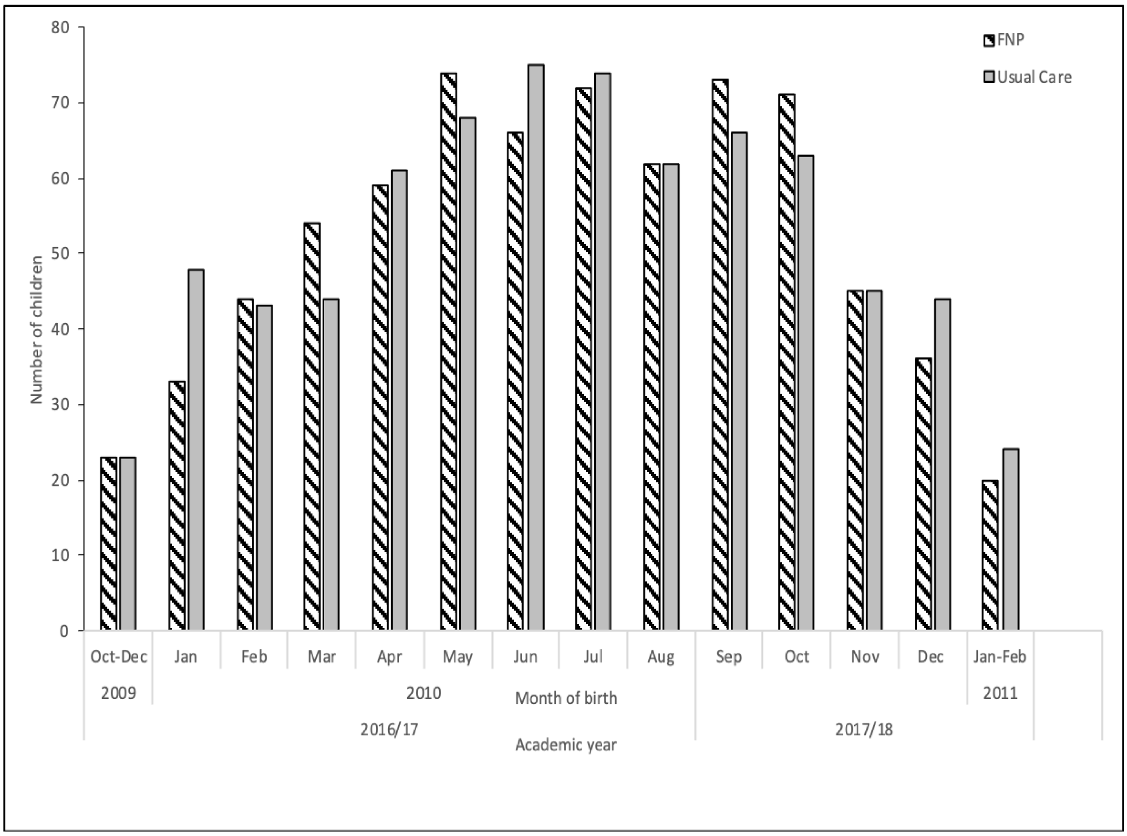

Robling M, *et al. BMJ Open* 2022; 12:e049960. doi: 10.1136/bmjopen-2021-049960

Table S12 Comparison of parameter estimates before and after adjustment for child's month[a] of birth

	Odds ratio[b] (95% CI), p-values	
	**Adjusted for site and minimisation variables[c]**	**Adjusted for site, minimisation variables[c] and month of birth**
**Key Stage 1 outcome**		
**Reading**		
Reaching at least the expected standard	1.23 (0.99 to 1.53), 0.051	1.26 (1.02 to 1.57), 0.035
Expected standard	*1.24 (0.99 to 1.56), 0.056*	*1.27 (1.01 to 1.59), 0.042*
Higher standard	*1.20 (0.88 to 1.65), 0.250*	*1.26 (0.91 to 1.75), 0.161*
**Maths**		
Reaching at least the expected standard	1.04 (0.84 to 1.28), 0.731	1.06 (0.85 to 1.31), 0.613
Expected standard	*1.06 (0.85 to 1.32), 0.611*	*1.07 (0.86 to 1.34), 0.527*
Higher standard	*0.93 (0.64 to 1.35), 0.711*	*0.93 (0.74 to 1.17), 0.522*
**Science**		
Reaching at least the expected standard	1.14 (0.91 to 1.43), 0.254	1.16 (0.92 to 1.47), 0.197
**Writing academic year 2017/18**		
Reaching at least the expected standard	1.24 (0.97 to 1.60), 0.090	1.26 (0.98 to 1.62), 0.076
Expected standard	*1.29 (1.00 to 1.68), 0.054*	*1.30 (1.00 to 1.69), 0.050*
Higher standard	*0.92 (0.51 to 1.64), 0.769*	*0.97 (0.54 to 1.75), 0.917*
**Early Years Assessment**		
Achieving GLD in all five areas of learning[d]	1.26 (1.03 to 1.55), 0.026	1.31 (1.05 to 1.62), 0.015
Achieving GLD in all 17 early learning goals	1.24 (1.01 to 1.52), 0.043	1.27 (1.03 to 1.58), 0.027
Total point score	0.65 (-0.11 to 1.41), 0.094	0.70 (-0.03 to 1.42), 0.060

a Quarter of birth runs from September to November, December to February, March to May, June to August; b Odds ratio represents the FNP arm compared to Usual care; c gestational age, smoking status at recruitment, and first or preferred language; d Children achieving a good level of development (GLD) are those achieving at least the expected level within the prime and specific areas of learning.

Source: National Pupil Database, The Department for Education

VII. Results of planned sub-group analysis

No differential effects due to maternal age, deprivation, participation in employment, education or training, or basic life skills were found in planned sub-group analyses for any of the objective maltreatment outcomes (Table S10 and Table S11). The beneficial impact of FNP on EYFSP total point score was mostly observed for children with mothers aged <16 years rather than those aged ≥16 at study entry but no other differences by study sub-group were found (Table S12).

The differential effects due to maternal age, deprivation, participation in employment, education or training, or basic life skills for KS1 reading, maths and science are displayed in Tables S13a to e and for writing in Table S14. At KS1, sex differences were large with girls outperforming boys in all four subject areas (Table S13a). The intervention effect was greater for boys than girls with a higher proportion reaching the expected standard in the FNP arm, particularly for reading and writing (and strengthened after adjusting for month of birth) and for the writing assessment (academic year 2016/17) this interaction reached statistical significance after adjusting for month of birth (post-hoc analysis). When examining how the intervention effects differed in mothers aged less than 16 years at recruitment compared to mothers aged 16 years or more in each KS1 assessment, differences were observed in maths (Table S13b) and writing (Table S14), with a larger effect seen in mothers aged less than 16 at recruitment, when compared to those aged 16 or more.

Table S13 Subgroup analyses for referrals to Children's social care services

N(%) referred	FNP	Usual care	Adjusted[a] OR (95% CI), p-value	Interaction p-value
**Child Gender**				
Boys	99 (26.3)	108 (27.7)	0.93 (0.65 to 1.33), 0.69	0.78
Girls	107 (27.9)	100 (28.1)	1.01 (0.69 to 1.48), 0.94	
**Maternal age at recruitment**				
<16 years	17 (34.7)	19 (34.6)	1.05 (0.44 to 2.46), 0.92	0.96
≥16 years	189 (26.6)	189 (27.4)	0.96 (0.72 to 1.30), 0.81	
**Adaptive functioning**				
No difficulty	140 (25.4)	149 (26.3)	0.99 (0.71 to 1.38), 0.96	0.67
Difficulty in basic skills	66 (31.7)	59 (33.0)	0.81 (0.59 to 1.41), 0.67	
**NEET status**				
EET	70 (20.3)	75 (23.1)	0.83 (0.54 to 1.29), 0.42	0.18
NEET	100 (32.5)	89 (29.2)	1.19 (0.80 to 1.78), 0.39	
**Deprivation quintile**				

Robling M, *et al. BMJ Open* 2022; 12:e049960. doi: 10.1136/bmjopen-2021-049960

1 – least deprivation	33 (20.4)	34 (23.9)	0.77 (0.44 to 1.36), 0.37	0.32
2	36 (23.8)	30 (20.5)	1.26 (0.61 to 2.60), 0.53	
3	42 (28.8)	43 (27.9)	0.96 (0.50 to 1.86), 0.91	
4	41 (31.1)	39 (25.3)	1.27 (0.69 to 2.31), 0.44	
5 – most deprivation	54 (33.3)	61 (42.4)	0.65 (0.35 to 1.22), 0.18	

[a] FNP compared to usual care. Analysis adjusted for stratification (site), minimisation variables (gestational age, smoking status at recruitment, and first or preferred language). N/EET – Not/In Education, Employment or Training (Source: National Pupil Database, The Department for Education)

Table S14 Subgroup analyses for child in need status

N(%) CIN	FNP	Usual care	Adjusted[a] OR (95% CI), p-value	Interaction p-value
**Gender**				
Boys	80 (21.3)	80 (20.5)	1.05 (0.72 to 1.53), 0.81	0.56
Girls	81 (21.1)	82 (23.0)	0.91 (0.60 to 1.38), 0.65	
**Maternal age at recruitment**				
<16 years	14 (28.6)	16 (29.1)	1.00 (0.41 to 2.43), 0.99	0.98
≥16 years	147 (20.7)	146 (21.1)	0.98 (0.72 to 1.33), 0.88	
**Adaptive functioning**				
No difficulty	106 (19.2)	113 (20.0)	0.99 (0.70 to 1.42), 0.98	0.76
Difficulty in basic skills	55 (26.4)	49 (27.4)	0.93 (0.59 to 1.47), 0.76	
**NEET**				
EET	52 (16.0)	52 (15.1)	0.93 (0.57 to 1.53), 0.78	0.42
NEET	81 (26.3)	72 (23.6)	1.18 (0.77 to 1.77), 0.46	
**Deprivation quintile**				
1 – least deprivation	24 (14.8)	22 (15.5)	0.92 (0.49 to 1.73), 0.79	0.62
2	30 (19.9)	22 (15.1)	1.53 (0.68 to 3.47), 0.31	
3	32 (21.9)	36 (23,4)	0.89 (0.52 to 1.54), 0.68	
4	32 (24.2)	34 (22.1)	1.15 (0.64 to 2.06), 0.64	
5 – most deprivation	43 (26.5)	48 (33.3)	0.72 (0.38 to 1.38), 0.32	

[a] FNP compared to usual care. Analysis adjusted for stratification (site), minimisation variables (gestational age, smoking status at recruitment, and first or preferred language). N/EET – Not/In Education, Employment or Training (Source: National Pupil Database, The Department for Education).

Table S15 Subgroup analyses for Early Years Assessment total point score

Mean (SD) total point score	FNP	Usual care	Adjusted[a] mean difference (95% CI), p-value	Interaction p-value
**Gender**				
Boys	N=369<br>30.57 (7.33)	N=383<br>29.84 (7.56)	0.75 (-0.31 to 1.82), 0.17	0.57
Girls	N=374<br>33.85 (6.79)	N=345<br>33.53 (7.22)	0.34 (-0.67 to 1.35), 0.51	
**Maternal age at recruitment**				
<16 years	N=45<br>32.44 (6.71)	N=52<br>28.91 (7.70)	3.65 (0.76 to 6.55), 0.013	0.046
≥16 years	N= 698<br>32.21 (7.28)	N= 676<br>31.80 (7.58)	0.42 (-0.36 to 1.21), 0.29	
**Adaptive functioning**				
No difficulty	N=540<br>32.99 (7.12)	N=552<br>32.11 (7.65)	0.88 (0.003 to 1.75), 0.049	0.50
Difficulty in basic skills	N=203<br>30.18 (7.20)	N=175<br>29.94 (7.33)	0.34 (-1.11 to 1.79), 0.65	
**NEET status**				
In EET	N=339<br>32.45 (7.12)	N=322<br>32.37 (7.68)	0.15 (-0.99 to 1.28), 0.80	0.95
NEET	N= 304<br>31.72 (7.57)	N=299<br>31.50 (7.43)	0.19 (-1.00 to 1.38), 0.76	
**Index of Multiple Deprivation quintiles**				
1 = least deprived	N=159<br>33.16 (7.44)	N=139<br>31.53 (8.24)	1.72 (-0.07 to 3.50), 0.060	ref
2	N=148<br>32.16 (6.97)	N=144<br>31.59 (7.05)	0.65 (-0.96 to 2.27), 0.43	0.84
3	N=146<br>32.16 (6.79)	N=151<br>32.59 (7.27)	-0.20 (-1.79 to 1.39), 0.80	0.21
4	N=128<br>30.95 (7.53)	N=148<br>31.11 (7.50)	-0.31 (-2.11 to 1.48), 0.73	0.85
5 = most deprived	N=156<br>32.41 (7.46)	N=140<br>31.03 (8.12)	1.45 (-0.33 to 3.22), 0.11	0.74

[a] FNP compared to usual care. Analysis adjusted for stratification (site), minimisation variables (gestational age, smoking status at recruitment, and first or preferred language). N/EET – Not/In Education, Employment or Training (Source: National Pupil Database, The Department for Education).

Table S16 Subgroup analyses for KS1 assessments reaching the expected standard (RES)

(a) Gender

N(%)	Boys		Girls	
	**Not RES**	**RES**	**Not RES**	**RES**
**Reading**				
FNP	152 (41.8)	212 (58.2)	105 (27.9)	271 (72.1)
Usual care	182 (47.3)	203 (52.7)	107 (30.8)	240 (69.2)
Adjusted[a] OR (95% CI), p-value	1.27 (0.95 to 1.69), 0.11		1.16 (0.84 to 1.59), 0.38	
Interaction p-value=0.68				

Adjusted[b] OR (95% CI), p-value	1.30 (0.96 to 1.75), 0.09		1.17 (0.84 to 1.62), 0.36	
Interaction p-value=0.68				
**Maths**				
FNP	149 (40.9)	215 (59.1)	132 (35.1)	244 (64.9)
Usual care	166 (43.1)	219 (56.9)	117 (33.7)	230 (66.3)
Adjusted[a] OR (95% CI), p-value	1.11 (0.83 to 1.49), 0.49		0.95 (0.69 to 1.29), 0.72*	
Interaction p-value=0.46				
Adjusted[b] OR (95% CI), p-value	1.14 (0.84 to 1.54), 0.40		0.94 (0.69 to 1.29), 0.71	
Interaction p-value=0.47				
**Science**				
FNP	120 (33.0)	244 (67.0)	83 (22.1)	293 (77.9)
Usual care	138 (35.8)	247 (64.2)	81 (23.3)	266 (76.7)
Adjusted[a] OR (95% CI), p-value	1.16 (0.86 to 1.58), 0.34		1.08 (0.76 to 1.53), 0.68	
Interaction p-value=0.77				
Adjusted[b] OR (95% CI), p-value	1.18 (0.87 to 1.63), 0.28		1.08 (0.76 to 1.55), 0.66	
Interaction p-value=0.72				

RES = Reaching the expected standard; [a] Analysis adjusted for stratification (site), minimisation variables (gestational age, smoking status at recruitment, and first or preferred language); [b] Analysis adjusted for stratification (site), minimisation variables (gestational age, smoking status at recruitment, and first or preferred language) and month of birth. (Source: National Pupil Database, The Department for Education).

(b) Maternal age at recruitment

N(%)	Maternal age<16 years		Maternal age≥16 years	
	Not RES	RES	Not RES	RES
**Reading**				
FNP	21 (44.7)	26 (55.3)	236 (34.1)	457 (65.9)
Usual care	29 (52.7)	26 (47.3)	260 (38.4)	417 (61.6)
Adjusted[a] OR (95% CI), p-value	1.21 (0.97 to 1.52), 0.09		1.49 (0.65 to 3.41), 0.34	
Interaction p-value=p=0.66				
Adjusted[b] OR (95% CI), p-value	1.50 (0.65 to 3.48), 0.34		1.25 (0.99 to 1.57), 0.07	
Interaction p-value=0.62				
**Maths**				
FNP	16 (34.0)	31 (66.0)	265 (38.2)	428 (61.8)
Usual care	32 (58.2)	23 (41.8)	251 (37.1)	426 (62.9)
Adjusted[a] OR (95% CI), p-value	3.15 (0.94 to 10.48), 0.06		0.96 (0.77 to 1.19), 0.69	
Interaction p-value=0.011				

Robling M, *et al. BMJ Open* 2022; 12:e049960. doi: 10.1136/bmjopen-2021-049960

Adjusted[b] OR (95% CI), p-value	3.23 (1.36 to 7.67), 0.008		0.98 (0.78 to 1.22), 0.83	
Interaction p-value=0.014				
**Science**				
FNP	18 (38.3)	29 (61.7)	185 (26.7)	508 (73.3)
Usual care	25 (45.4)	30 (54.6)	194 (28.7)	483 (71.3)
Adjusted[a] OR (95% CI), p-value	1.54 (0.53 to 4.47), 0.42		1.11 (0.88 to 1.41), 0.39	
Interaction p-value=0.56				
Adjusted[b] OR (95% CI), p-value	1.37 (0.59 to 3.19), 0.467		1.14 (0.89 to 1.45), 0.29	
Interaction p-value=0.69				

RES = Reaching the expected standard; [a] FNP compared to Usual care. Analysis adjusted for stratification (site), minimisation variables (gestational age, smoking status at recruitment, and first or preferred language); [b] Analysis adjusted for stratification (site), minimisation variables (gestational age, smoking status at recruitment, and first or preferred language) and month of birth. (Source: National Pupil Database, The Department for Education).

(c) Adaptive functioning

N(%)	No difficulty with basic skills		Difficulty with basic skills	
	**Not RES**	**RES**	**Not RES**	**RES**
**Reading**				
FNP	152 (30.5)	347 (69.5)	87 (42.9)	116 (57.1)
Usual care	188 (36.2)	332 (63.8)	84 (48.0)	91 (52.0)
Adjusted[a] OR (95% CI), p-value	1.29 (0.98 to 1.70), 0.07		1.23 (0.82 to 1.86), 0.32	
Interaction p-value=0.61				
Adjusted[b] OR (95% CI), p-value	1.29 (0.98 to 1.69), 0.07		1.25 (0.83 to 1.91), 0.29	
Interaction p-value=0.95				
**Maths**				
FNP	186 (34.6)	351 (65.4)	95 (46.8)	108 (53.2)
Usual care	192 (34.5)	364 (65.5)	91 (52.0)	84 (48.0)
Adjusted[a] OR (95% CI), p-value	1.01 (0.78 to 1.29), 0.97		1.27 (0.84 to 1.93), 0.26	
Interaction p-value=0.38				
Adjusted[b] OR (95% CI), p-value	1.01 (0.78 to 1.31), 0.93		1.32 (0.86 to 2.03), 0.20	
Interaction p-value=0.28				
**Science**				
FNP	130 (24.2)	407 (75.8)	73 (36.0)	130 (64.0)
Usual care	153 (27.5)	403 (72.5)	66 (37.7)	109 (62.3)
Adjusted[a] OR (95% CI), p-value	1.20 (0.92 to 1.58), 0.19		1.10 (0.71 to 1.69), 0.68	
Interaction p-value=0.71				

Adjusted[b] OR (95% CI), p-value	1.22 (0.93 to 1.62), 0.16	1.13 (0.73 to 1.74), 0.59
Interaction p-value=0.82		

RES = Reaching the expected standard; [a] FNP compared to Usual care. Analysis adjusted for stratification (site), minimisation variables (gestational age, smoking status at recruitment, and first or preferred language); [b] Analysis adjusted for stratification (site), minimisation variables (gestational age, smoking status at recruitment, and first or preferred language) and month of birth. (Source: National Pupil Database, The Department for Education).

(d) Not in Education, employment or training (NEET) status

N(%)	NEET		In EET	
	**Not RES**	**RES**	**Not RES**	**RES**
**Reading**				
FNP	105 (31.2)	232 (68.8)	109 (36.0)	194 (64.0)
Usual care	115 (35.8)	206 (61.2)	121 (40.5)	178 (59.5)
Adjusted[a] OR (95% CI), p-value	1.26 (0.91 to 1.75), 0.17		1.21 (0.87 to 1.69), 0.26	
Interaction p-value=0.86				
Adjusted[b] OR (95% CI), p-value	1.31 (0.94 to 1.83), 0.17		1.25 (0.89 to 1.76), 0.19	
Interaction p-value=0.83				
**Maths**				
FNP	122 (36.2)	215 (63.8)	122 (40.3)	181 (59.7)
Usual care	107 (33.3)	214 (66.7)	120 (40.1)	179 (59.9)
Adjusted[a] OR (95% CI), p-value	0.89 (0.65 to 1.23), 0.48		0.99 (0.72 to 1.38), 0.97	
Interaction p-value=0.64				
Adjusted[b] OR (95% CI), p-value	0.93 (0.66 to 1.29), 0.65		1.03 (0.73 to 1.42), 0.90	
Interaction p-value=0.68				
**Science**				
FNP	87 (25.8)	250 (74.2)	821 (27.1)	221 (72.9)
Usual care	78 (24.3)	243 (75.7)	98 (32.8)	201 (67.2)
Adjusted[a] OR (95% CI), p-value	0.93 (0.65 to 1.33), 0.69		1.31 (0.92 to 1.87), 0.14	
Interaction p-value=0.20				
Adjusted[b] OR (95% CI), p-value	0.96 (0.67 to 1.39), 0.84		1.36 (0.95 to 1.95), 0.10	
Interaction p-value=0.22				

RES = Reaching the expected standard; [a] FNP compared to Usual care. Analysis adjusted for stratification (site), minimisation variables (gestational age, smoking status at recruitment, and first or preferred language); [b] Analysis adjusted for stratification (site), minimisation variables (gestational age, smoking status at recruitment, and first or preferred language) and month of birth. N/EET – Not/In Education, Employment or Training (Source: National Pupil Database, The Department for Education).

(e) Index of Multiple Deprivation quintiles

	N(%)	Q1=least deprived		Q2		Q3		Q4		Q5=most deprived	
		**Not RES**	**RES**	**Not RES**	**RES**	**Not RES**	**RES**	**Not RES**	**RES**	**Not RES**	**RES**
**Reading**	FNP	49 (30.3)	113 (67.7)	53 (36.3)	93 (63.7)	49 (33.6)	97 (66.4)	48 (37.2)	81 (62.8)	57 (37.8)	94 (62.2)
	Usual care	54 (38.9)	85 (61.1)	64 (44.1)	81 (55.9)	48 (31.8)	103 (68.2)	64 (43.0)	85 (57.0)	56 (39.4)	86 (60.6)
	Adjusted[a] OR (95% CI), p-value	1.57 (0.88 to 2.77), 0.12		1.42 (0.88 to 2.29), 0.16		0.96 (0.59 to 1.57), 0.88		1.33 (0.81 to 2.18), 0.25		1.07 (0.60 to 1.89), 0.83	
	Adjusted[b] OR (95% CI), p-value	1.75 (1.00 to 3.07), 0.05		1.42 (0.85 to 2.37), 0.18		0.89 (0.53 to 1.48), 0.64		1.41 (0.86 to 2.34), 0.18		1.00 (0.57 to 1.75), 0.99	
	Interaction p-value=0.70										
	Interaction p-value=0.37										
Maths	FNP	56 (34.6)	106 (65.4)	57 (39.0)	89 (61.0)	50 (34.3)	96 (65.7)	55 (42.6)	74 (57.4)	62 (41.1)	89 (58.9)
	Usual care	52 (37.4)	87 (62.6)	60 (41.4)	85 (58.6)	56 (37.1)	95 (62.9)	60 (40.3)	89 (59.7)	54 (38.0)	88 (62.0)
	Adjusted[a] OR (95% CI), p-value	1.15 (0.72 to 1.85), 0.56		1.12 (0.69 to 1.82), 0.65		1.17 (0.69 to 1.97), 0.56		0.93 (0.57 to 1.52), 0.77		0.91 (0.56 to 1.47), 0.69	
	Adjusted[b] OR (95% CI), p-value	1.36 (0.82 to 2.24), 0.23		1.12 (0.68 to 1.82), 0.66		1.11 (0.65 to 1.90), 0.69		0.98 (0.60 to 1.62), 0.95		0.88 (0.54 to 1.44), 0.62	
	Interaction p-value=0.91										
	Interaction p-value=0.81										

Robling M, *et al. BMJ Open* 2022; 12:e049960. doi: 10.1136/bmjopen-2021-049960

	N(%)	Q1=least deprived		Q2		Q3		Q4		Q5=most deprived	
		**Not RES**	**RES**	**Not RES**	**RES**	**Not RES**	**RES**	**Not RES**	**RES**	**Not RES**	**RES**
**Science**	FNP	37 (22.8)	125 (77.2)	36 (24.7)	110 (75.3)	40 (27.4)	106 (72.6)	45 (34.9)	84 (65.1)	44 (29.1)	107 (70.9)
	Usual care	45 (32.4)	94 (67.6)	45 (31.0)	100 (69.0)	35 (23.2)	116 (76.8)	47 (31.5)	102 (68.5)	46 (32.4)	96 (67.6)
	Adjusted[a] OR (95% CI), p-value	1.68 (1.00 to 2.81), 0.048		1.39 (0.80 to 2.41), 0.25		0.82 (0.46 to 1.47), 0.51		0.89 (0.52 to 1.51), 0.65		1.24 (0.74 to 2.06), 0.42	
	Adjusted[b] OR (95% CI), p-value	1.94 (1.13 to 3.30), 0.015		1.40 (0.76 to 2.57), 0.28		0.78 (0.43 to 1.40), 0.41		0.92 (0.55 to 1.55), 0.76		1.16 (0.69 to 1.96), 0.57	
	Interaction p-value=0.25										
	Interaction p-value=0.11										

RES = Reaching the expected standard; a FNP compared to Usual care; [a] Analysis adjusted for stratification (site), minimisation variables (gestational age, smoking status at recruitment, and first or preferred language); [b] Analysis adjusted for stratification (site), minimisation variables (gestational age, smoking status at recruitment, and first or preferred language) and month of birth. (Source: National Pupil Database, The Department for Education).

Robling M, *et al. BMJ Open* 2022; 12:e049960. doi: 10.1136/bmjopen-2021-049960

Table S17 Subgroup analyses for reaching the expected standard in the writing KS1 assessment for 2016/17 academic year

N(%)	FNP	Usual care	Adjusted[a] OR (95% CI), p-value	Interaction p-value	Adjusted[b] OR (95% CI), p-value	Interaction p-value
**Gender**						
Boys	109 (42.9)	83 (31.8)	1.61 (1.13 to 2.32), 0.009	0.034	1.62 (1.13 to 2.33), 0.009	0.039
Girls	132 (54.1)	126 (55.8)	1.03 (0.76 to 1.39), 0.87		0.94 (0.65 to 1.37), 0.76	
**Maternal age at recruitment**						
<16 years	18 (54.6)	9 (22.5)	4.67 (1.47 to 14.84), 0.009	0.016	5.28 (1.49 to 18.73), 0.010	0.018
≥16 years	223 (48.0)	200 (44.7)	1.13 (0.87 to 1.47), 0.35		1.15 (0.89 to 1.50), 0.29	
**Adaptive functioning**						
No difficulty	189 (52.5)	174 (46.7)	1.26 (0.93 to 1.70), 0.14	0.72	1.26 (0.93 to 1.70), 0.14	0.67
Difficulty in basic skills	52 (37.7)	34 (30.1)	1.41 (0.77 to 2.58), 0.26		1.53 (0.81 to 2.90), 0.19	
**NEET**						
EET (no not EET)	109 (47.8)	119 (52.2)	0.82 (0.56 to 1.19), 0.29	0.019	0.82 (0.56 to 1.19), 0.29	0.028
NEET	105 (49.1)	77 (38.3)	1.56 (1.05 to 2.30), 0.027		1.56 (1.05 to 2.30), 0.027	
**Deprivation quintile**						
1 – least deprived	63 (53.9)	39 (42.9)	1.65 (0.90 to 3.05), 0.11	0.89	1.83 (0.95 to 3.51), 0.07	0.86
2	48 (46.2)	46 (44.7)	1.00 (0.52 to 1.95), 0.99		1.04 (0.53 to 2.03), 0.92	
3	46 (50.6)	49 (46.7)	1.20 (0.67 to 2.15), 0.53		1.22 (0.66 to 2.26), 0.53	
4	40 (45.5)	35 (38.5)	1.28 (0.70 to 2.35), 0.42		1.28 (0.69 to 2.35), 0.43	
5 – most deprived	42 (44.2)	39 (41.1)	1.16 (0.64 to 2.08), 0.629		1.17 (0.64 to 2.14), 0.602	

Robling M, *et al. BMJ Open* 2022; 12:e049960. doi: 10.1136/bmjopen-2021-049960

[a] FNP compared to usual care. Analysis adjusted for stratification (site), minimisation variables (gestational age, smoking status at recruitment, and first or preferred language); [b] Analysis additionally adjusted for month of birth. N/EET – Not/In Education, Employment or Training (Source: National Pupil Database, The Department for Education).

Robling M, *et al. BMJ Open* 2022; 12:e049960. doi: 10.1136/bmjopen-2021-049960

Domestic abuse and CIN status:

Domestic abuse experienced by the mother was assessed using the Composite Abuse Scale (CAS) in the BB:0-2 trial at 24 months follow-up (with a score 1 or greater indicating some sort of abuse). The CAS could only be administered if the interview was face-to-face (as opposed to by telephone) and only if the participant was alone (N=607). In the BB:2-6 sample, this could be examined in 595 participants (FNP:322, Usual Care:273) and the proportion of participants experiencing some form of abuse was comparable to that found in the BB:0-2 trial (FNP: 122 (27.9%) vs UC:113 (41.4%)). There was also a relationship found between CAS and CIN status with 49 (20.9%) participants who experienced some abuse having a child being in need versus 42 (11.8%) of those who did not experience abuse. Domestic abuse at 24 months was to be explored as a possible effect mediator but due to lack of intervention effect in the child's CIN status, the need to examine the proportion of effect mediated by experience of domestic abuse at 24 months was redundant.

Care-experienced mothers and CIN status:

In addition, care-experienced mothers at time of recruitment to the BB:0-2 trial was examined. This was defined from the CLA dataset. Sixty-one mothers were identified as having experienced a period of care, four of whom were identified as care-experienced only after their BB:0-2 recruitment date. Fifty-seven mothers were examined for their duration of care and the women randomised to receive FNP experienced on average nine months more care than the Usual care arm (median (25th to 75th centile) FNP: 40 (15.5 to 113.0) UC: 31 (7.0 to 69.5) months). After merging these care-experienced mothers into the children's dataset, 56 mothers remained; seven mothers were excluded from the subgroup analyses since their child did not have any NPD data. Children of these care-experienced mothers were at a higher risk of being defined as in need themselves but no interaction was observed between trial arm and mothers care status. The duration of the maternal care status as a possible mediator effect was also stated in the statistical analysis plan but similarly to domestic abuse, due to lack of intervention effect in child's CIN status, then the need to examine the proportion of effect mediated by length of mother's care experience was redundant.

VIII. Treatment efficacy

The analyses of the following outcomes were re-run to investigate the effect of FNP dosage (number of visits over all delivery phases) using complier averaged causal effects modelling by fitting a structural mean model. The programme stated maximum number of visits over the whole period of FNP is 64 (14 in the pregnancy phase, 28 in the infancy phase, 22 in the toddler phase).

Referrals to CSC services: Adjusting the analysis for receipt of full dosage of FNP visits, made no difference to the conclusion of the main analysis for referral to social services (efficacy per visit odds ratio (OR)=1.00, 95% CI=0.99 to 1.004, p=0.613).

CIN status: Adjusting the analysis for receipt of FNP made no difference to the conclusion of the primary outcome (efficacy per visit OR=1.00, 95% CI=0.99 to 1.004, p=0.613).

Early Years assessment total point score: Adjusting the analysis for receipt of FNP we observe a 0.02 increase in point score in the between-arm mean difference in early years total score (as the number of visits increase so does the score) however the confidence interval around this estimate is wide (-0.004 to 0.040). The estimated treatment efficacy for participants receiving all 64 visits was 1.15 (95% CI - 0.26 to 2.56), p=0.106 showing no change to the overall conclusions compared to the primary analysis.

KS1 attainment: Adjusting the analysis for FNP receipt we observe an increase in the odds of an event occurring in the FNP arm compared UC arm for every unit increase in FNP visit received. For all areas of learning (reading, writing, maths and science), adjusting the analysis for FNP receipt strengthens the effect of FNP, but it is the reading and writing assessment where given the full dosage of 64 FNP visits the odds make a significant difference. For reading assessments, the odds of reaching the expected standard increases from OR=1.23 (95% CI=0.99 to 1.53), p=0.051 in the main analysis to 1.38 (1.07 to 1.77), p=0.015. The efficacy per visit for reading was 1.005 (1.001 to 1.009), p=0.015; maths: 1.001 (0.997 to 1.005), p=0.535; science: 1.004 (0.999 to 1.008), p=0.112; writing: 1.013 (1.09 to 1.017), p=0.001.

IX. Exploration of surveillance bias upon Child In Need outcome assessment

Table S18 Baseline maternal characteristics, school readiness outcomes for children referred to CSC and classed as a Child In Need, before the age of 4 years old by intervention arm.

	**FNP N=60**	**Usual Care N=62**	**OR (95% CI)**
Maternal age (years) at recruitment *Median (25th to 75th centiles)*	17.7 (16.9 to 18.3)	17.8 (16.7 to 18.7)	0.97 (0.73 to 1.27)
Not in Education, Employment or Training at recruitment *N(%)*			
Education, Employment or Training	21 (42.0)	11 (22.0)	
Not in Education, Employment or Training	30 (58.0)	39 (78.0)	2.45 (1.04 to 5.93)
Not applicable (<16 years at interview)/ missing	9	12	

Ethnicity *N(%)*	Numbers too small		
Deprivation score[1] *Median (25th to 75th centiles)*	43.0 (25.0 to 56.1)	45.0 (31.8 to 62.7)	0.99 (0.98 to 1.01)
Adaptive functioning			
Had 3 or less life skills			
No	38 (63.3)	43 (69.4)	
Yes	22 (36.7)	19 (30.6)	1.41 (0.67 to 2.98)
Difficulty in at least one basic skills *N(%)*			
No	37 (61.7)	43 (69.4)	
Yes	23 (38.3)	19 (30.6)	1.31 (0.62 to 2.8)
At least one burden *N(%)*			
No	39 (65.0)	42 (68.9)	
Yes	21 (35.0)	19 (31.1)	1.19 (0.56 to 2.54)
Social support			
Maximum social support N(%) (score=100)	10 (16.9)	9 (14.5)	1.20 (0.45 to 3.21)
Relationship quality *(score 7 to 35)*[2]	28 (25 to 30)	30 (25 to 32)	0.97 (0.89 to 1.05)
Self-efficacy *(score 10 to 40)*[3] *Median (25th to 75th centiles)*	30 (28 to 32)	30 (27.25 to 31)	1.05 (0.97 to 1.15)
Family resource *(score 4 to 20)*[4] *Mean (SD)*	12.6 (4.2)	12.7 (3.9)	0.91 (0.64 to 1.30)
CRAFFT *(score 0 to 6)*[5] *Median (25th to 75th centiles)*	1 (0 to 2)	1 (0 to 2)	1.07 (0.85 to 1.35)
**Early Years**			
GLD in all 5 areas of learning *N(%)*	26 (44.1)	22 (37.9)	1.29 (0.62 to 2.70)
GLD in all 17 early learning goals *N(%)*	26 (44.1)	20 (34.5)	1.50 (0.71 to 3.16)
Total point score over all 17 early learning goals *Median (25th to 75th centiles)*	32 (22 to 34)	30.5 (21 to 34)	Mean difference = 1.66 (-1.14 to 4.45)

[1] Higher score indicates more deprived, [2] Higher score indicates a better relationship quality, [3] Higher score indicates higher level of self-efficacy, [4] Higher score indicates higher level of family resource, [5] Greater risk of problems related to drugs and alcohol

X. Missing data

Since only a small number (n=40 (2.7%)) of children were not linked to any NPD dataset and not included in the primary outcome analyses, no multiple imputation was carried out.

XI. Cost Consequence Analysis

Table S19 Cost-consequence balance sheet for the family-nurse based intervention for the four years between children's ages 2 and 6.

**Mothers**						
	Mean resource use per participant			Mean cost per participant (£)		
	FNP	Usual Care	Incremental (FNP-Usual Care)	FNP	Usual Care	Incremental (FNP-Usual Care)
**Health related resource use**						
Inpatient Attendances						
Overnight admissions (N)	3.04	2.99	0.05	2,826.79	2,844.58	-17.79
Overnight length of stay (N nights)	2.32	2.32	0.00			
Day Admittances	2.93	2.91	0.02	1,874.61	1,890.11	-15.50
Outpatient Attendances						
All Outpatient Attendances	23.29	22.61	0.68	2,244.62	2,189.32	55.30
Accident and Emergency						
A&E Visits (N)	5.19	4.93	0.26	411.34	392.03	19.31
Total Resource Use						
				7,357.36	7,316.04	41.32 p=0.393
**Children**						
	Mean resource use per participant			Mean cost per participant (£)		
	FNP	Usual Care	Incremental (FNP-Usual Care)	FNP	Usual Care	Incremental (FNP-Usual Care)
**Health related resource use**						
Inpatient Attendances - All						
Overnight admissions (N)	1.57	1.55	0.02	1,962.95	2,033.71	-70.76
Overnight length of stay (N nights)	3.57	3.39	0.18			
Day Admittances	0.98	0.97	0.01	744.94	724.90	20.04
Inpatient Attendances – All (Excluding Injuries and Ingestions)						
Overnight admissions (N)	1.51	1.49	0.02	1,883.44	1,951.15	-67.71
Overnight length of stay (N nights)	3.61	3.44	0.17			
Day Admittances	0.90	0.88	0.02	689.22	667.09	22.13

Inpatient Attendances – Injuries and Ingestions Only						
Overnight admissions (N)	0.06	0.06	0.00			
Overnight length of stay (N nights)	2.50	2.05	0.45	79.51	82.55	-3.04
Day Admittances	0.08	0.09	-0.01	55.72	57.81	-2.09
Outpatient Attendances						
All Outpatient Attendances	6.89	7.44	-0.55	673.24	727.79	-54.55
Accident and Emergency - All						
A&E Visits (N)	4.93	4.62	0.31	395.73	366.83	28.90
Accident and Emergency - All (Excluding Injuries and Ingestions)						
A&E Visits (N)	3.80	3.52	0.28	305.12	280.20	24.92
Accident and Emergency – Injuries and Ingestions Only						
A&E Visits (N)	1.12	1.10	0.02	75.09	69.91	5.18
Total Resource Use						
				3,778.13	3,853.22	-75.09 p=0.865
**Consequences**						
Child in Need status recorded at any time during the follow-up period.						OR: 0.98 (95%CI: 0.74 to 1.31) p=0.902
Referral to Social Services						OR: 0.97 (0.74 to 1.28) p=0.829
Child Protection registration (CPP)						OR: 1.04 (0.69 to 1.57) p=0.846
Details of a CPP						
• Neglect						Reference
• Emotional						OR: 2.77 (1.02 to 7.56) p=0.046
• Physical						OR: 1.25 (0.32 to 4.88) p=0.743
• Sexual and multiple						OR: 1.13 (0.33 to 3.86) p=0.848
CIN categorisation						
• Abuse or Neglect						Reference
• Family dysfunction						OR: 0.95 (0.54 to 1.69) p=0.868
• Family in acute stress						OR: 1.47 (0.61 to 3.54) p=0.389
• Low income, absent parenting, socially unacceptable behaviour						OR: 2.38 (0.86 to 6.57) p=0.093
• Child/parent disability						OR: 1.47 (0.45 to 4.84) p=0.525
Looked after status						OR: 0.90 (0.52 to 1.57) p=0.712
Child looked after period of care (months)						OR: 0.75 (0.65 to 0.86) p<0.001
DNA appointments						
• DNA at least once						OR: 1.00 (0.79 to 1.26) p=0.997
Injuries and ingestions						
• Emergency attendance						
o No						Reference

○ Yes	OR: 1.17 (0.95 to 1.45) p=0.149
• Admission to hospital	
○ No	Reference
○ Yes	OR: 0.87 (0.63 to 1.20) p=0.407
• Either hospital attendance and/or admission to hospital	
○ No	Reference
○ Yes	OR: 1.11 (0.89 to 1.37) p=0.351
Special Educational Needs	OR: 0.83 (0.67 to 1.03) p=0.097
Early Years assessment	
• Achieving good level of development in all five areas of learning	OR: 1.26 (1.03 to 1.55) p=0.026
• Achieving good level of development in all 17 early learning goals	OR: 1.24 (1.01 to 1.52) p=0.043
School attendance	
• Overall absences	
○ No absences	Reference
○ At least one absence	OR: 1.00 (0.47 to 2.12) p=0.998
• Overall unauthorised absences	
○ No absences	Reference
○ At least one absence	OR: 0.95 (0.76 to 1.18) p=0.620
• Overall authorised absences	
○ No absences	Reference
○ At least one absence	OR: 1.01 (0.58 to 1.75) p=0.984
KS1 attainment	
• Reading	
○ Lower than expected	Reference
○ Reaching the expected standard	OR: 1.23 (0.99 to 1.53) p=0.051
• Writing academic year 2016/17	
○ Lower than expected	Reference
○ Reaching the expected standard	OR: 1.24 (0.97 to 1.60) p=0.090
• Maths	
○ Lower than expected	Reference
○ Reaching the expected standard	OR: 1.04 (0.84 to 1.28) p=0.731
• Science	
○ Lower than expected	Reference
○ Reaching the expected standard	OR: 1.14 (0.91 to 1.43) p=0.254

Robling M, *et al. BMJ Open* 2022; 12:e049960. doi: 10.1136/bmjopen-2021-049960

XII. Postpartum resource use data collected for SPHN contacts

The 6-, 12- and 18-months telephone interview CRF included the following items regarding SPHN (ie health visitor) contacts:

6-month interview question:

Since your baby(ies) was born can I ask how many times you have seen any of the following people?

Note to interviewer: For each positive question ask & record

1. How many times do you think you have seen them in that time?
2. Where did you see them (e.g. hospital, clinic, home)?

	Number of home visits	Number of clinic visits
Health visitor		

12-month interview question:

In the last 6 months can I ask how many times you have seen any of the following people?

Note to interviewer: For each positive question ask & record

1. How many times do you think you have seen them in that time?
2. Where did you see them (e.g. hospital, clinic, home)?

	Number of home visits	Number of clinic visits
Health visitor		

18-month interview question:

In the last 6 months can I ask how many times you have seen any of the following people?

Note to interviewer: For each positive question ask & record

1. How many times do you think you have seen them in that time?
2. Where did you see them (e.g. hospital, clinic, home)?

	Number of home visits	Number of clinic visits
Health visitor		

Robling M, *et al. BMJ Open* 2022; 12:e049960. doi: 10.1136/bmjopen-2021-049960
