# Supplementary material for: Nurse-led home-visitation programme for first-time mothers in reducing maltreatment and improving child health and development (BB:2-6): longer-term outcomes from a randomised cohort using data linkage
Source: BMJ Open. 2022 Feb 10;12(2):e049960. doi: 10.1136/bmjopen-2021-049960 (PMC8845181; doi:10.1136/bmjopen-2021-049960)
Supplement: Author's manuscript [file bmjopen-2021-049960.draft_revisions.pdf]

BMJ Open is committed to open peer review. As part of this commitment we make the peer review history of every article we publish publicly available.

When an article is published we post the peer reviewers' comments and the authors' responses online. We also post the versions of the paper that were used during peer review. These are the versions that the peer review comments apply to.

The versions of the paper that follow are the versions that were submitted during the peer review process. They are not the versions of record or the final published versions. They should not be cited or distributed as the published version of this manuscript.

BMJ Open is an open access journal and the full, final, typeset and author-corrected version of record of the manuscript is available on our site with no access controls, subscription charges or pay-per-view fees (http://bmjopen.bmj.com).

If you have any questions on BMJ Open's open peer review process please email info.bmjopen@bmj.com

BMJ Open

A nurse-led home-visitation programme for first-time mothers in reducing maltreatment and improving child health and development (BB:2-6): longer-term outcomes from data-linkage

Journal:	*BMJ Open*
Manuscript ID	bmjopen-2021-049960
Article Type:	Original research
Date Submitted by the Author:	05-Feb-2021
Complete List of Authors:	Robling, Michael; Cardiff University, Centre for Trials Research; DECIPHer, Cardiff University School of Social Sciences<br>Lugg-Widger, Fiona; Cardiff University, Centre for Trials Research<br>Cannings-John, Rebecca; Cardiff University Centre for Trials Research,<br>Angel, Lianna; Cardiff University, Centre for Trials Research<br>Channon, Sue; Cardiff University, Centre for Trials Research<br>Fitzsimmons, Deborah; Swansea University, College of Human and Health Sciences<br>Hood, Kerenza; Cardiff University,<br>Kenkre, Joyce; University of South Wales, Faculty of Life Sciences and Education<br>Moody, Gwenllian; Cardiff University, Centre for Trials Research<br>Owen-Jones, Eleri; Cardiff University, Centre for Trials Research<br>Pockett, Rhys; Swansea University, Swansea Centre for Health Economics<br>Sanders, Julia; Cardiff University, School of Healthcare Sciences<br>Segrott, Jeremy; Cardiff University, Centre for Trials Research; Cardiff University, DECIPHer Centre, Cardiff School of Social Sciences<br>Slater, Thomas; School of Social Sciences
Keywords:	PUBLIC HEALTH, Child protection < PAEDIATRICS, PREVENTIVE MEDICINE

SCHOLARONE™
Manuscripts

I, the Submitting Author has the right to grant and does grant on behalf of all authors of the Work (as defined in the below author licence), an exclusive licence and/or a non-exclusive licence for contributions from authors who are: i) UK Crown employees; ii) where BMJ has agreed a CC-BY licence shall apply, and/or iii) in accordance with the terms applicable for US Federal Government officers or employees acting as part of their official duties; on a worldwide, perpetual, irrevocable, royalty-free basis to BMJ Publishing Group Ltd ("BMJ") its licensees and where the relevant Journal is co-owned by BMJ to the co-owners of the Journal, to publish the Work in this journal and any other BMJ products and to exploit all rights, as set out in our licence.

The Submitting Author accepts and understands that any supply made under these terms is made by BMJ to the Submitting Author unless you are acting as an employee on behalf of your employer or a postgraduate student of an affiliated institution which is paying any applicable article publishing charge ("APC") for Open Access articles. Where the Submitting Author wishes to make the Work available on an Open Access basis (and intends to pay the relevant APC), the terms of reuse of such Open Access shall be governed by a Creative Commons licence – details of these licences and which Creative Commons licence will apply to this Work are set out in our licence referred to above.

Other than as permitted in any relevant BMJ Author's Self Archiving Policies, I confirm this Work has not been accepted for publication elsewhere, is not being considered for publication elsewhere and does not duplicate material already published. I confirm all authors consent to publication of this Work and authorise the granting of this licence.

For peer review only

Title: A nurse-led home-visitation programme for first-time mothers in reducing maltreatment and improving child health and development (BB:2-6): longer-term outcomes from data-linkage

Authorship:

Michael Robling, [1,2] (Director of Population Health Trials), RoblingMR@cardiff.ac.uk

Fiona Lugg-Widger, [1] (Research Fellow), LuggFV@cardiff.ac.uk

Rebecca Cannings-John, [1] (Senior Research Fellow), CanningsRL@cardiff.ac.uk

Lianna Angel, [2] (Research Associate), AngelL@cardiff.ac.uk

Susan Channon, [1] (Director Research Design and Conduct Service) ChannonS2@cardiff.ac.uk

Deborah Fitzsimmons, [3] (Personal Chair, Public Health, Policy and Social Sciences) d.fitzsimmons@swansea.ac.uk

Kerenza Hood, [1] (Director of the Centre for Trials Research) HoodK1@cardiff.ac.uk

Joyce Kenkre, [4] (Professor of Primary Care) joyce.kenkre@southwales.ac.uk

Gwenllian Moody, [1] (Research Associate) MoodyG@cardiff.ac.uk

Eleri Owen-Jones, [1] (Research Associate) Owen-JonesCE@cardiff.ac.uk

Rhys Pockett, [3] (Senior Lecturer) r.d.pockett@swansea.ac.uk

Julia Sanders, [5] (Professor of Clinical Nursing and Midwifery) SandersJ3@cardiff.ac.uk

Jeremy Segrott, [1,2] (Senior lecturer) SegrottJ@cardiff.ac.uk

Thomas Slater, [6] (Senior lecturer) slaterTB1@cardiff.ac.uk

Affiliations:

1. Centre for Trials Research, Cardiff University, Cardiff, UK
2. DECIPHer, Cardiff University, UK
3. Swansea Centre for Health Economics, Swansea University, UK
4. Faculty of Life Sciences and Education, University of South Wales, UK
5. School of Healthcare Sciences, Cardiff University, UK
6. School of Social Sciences, Cardiff University, UK

Corresponding author:

Michael Robling, Director of Population Health Trials, RoblingMR@cardiff.ac.uk, Centre for Trials Research, College of Biomedical & Life Sciences, Cardiff University, 7th Floor, Neuadd Meirionnydd, Heath Park, Cardiff. CF14 4YS

Keywords: Home-visiting, maltreatment, child development, routine data, cohort

For peer review only - http://bmjopen.bmj.com/site/about/guidelines.xhtml

Abstract (300 words)

Objectives To measure effectiveness of the Family Nurse Partnership (FNP) home-visiting programme in reducing maltreatment and improving maternal health and child health, developmental and educational outcomes; to explore effect moderators, mediators; to describe costs.

Design Follow-up of BB:0-2 trial cohort up to age 7 years in England using record linkage.

Participants 1,618 mothers and their firstborn child(ren) recruited to BB:0-2 trial who were not mandatorily withdrawn from the trial or opted out. Intervention families received up to 64 home visits by specially trained nurses from pregnancy until firstborn child was 2 years old, plus usually provided health and social care support. Comparator was usual care alone.

Outcome measures The primary outcome was state-verified child-in-need status recorded at any time during follow-up. Secondary outcomes were referral to social services, child protection registration (plan), child-in-need categorisation, looked-after status, recorded injuries and ingestions at any time during follow-up, early childcare and educational attendance, school readiness and attainment at Key Stage 1 (KS1) and healthcare costs.

Results Match rates for 1,547 eligible children (1517 singletons, 15 sets of twins) were 98.3% (NHS Digital) and 97.4% (National Pupil Database). There was no difference between study arms in the proportion of children being registered as in need (adjusted OR=0.98, 95% CI=0.74,1.31), or for any other measure of maltreatment. Children in the FNP arm were more likely to achieve a good level of development at reception age (school readiness) (adjusted OR=1.24, 95% CI= 1.01,1.52). After adjusting for birth month, children in FNP arm were more likely to reach the expected standard in reading at KS1 (adjusted OR=1.26, 95% CI=1.02,1.57). We found no other trial arm differences including for resource use and costs.

Conclusions FNP did not result in benefit for maltreatment or maternal outcomes. There was evidence of small advantages in school readiness and attainment at KS1.

For peer review only

Strengths and Limitations

- We used administrative records and novel data linkage to provide longer-term follow-up for a well characterised trial cohort
- This has maximised cohort retention and minimised bias
- We used data from a range of sectors including health, social care and education to assess the broad impact of the specialist home visiting programme

For peer review only - http://bmjopen.bmj.com/site/about/guidelines.xhtml

Manuscript (Word count is 4206)

Introduction

Protecting children from maltreatment and promoting their healthy development are UK policy priorities. The Family Nurse Partnership (FNP) is a preventative home-visiting programme for young women expecting their first child. FNP was developed in the USA as the Nurse Family Partnership (NFP) and subsequently adapted for implementation in England in 2007.

Three US trials have demonstrated programme improvements in prenatal health behaviours and birth outcomes, sensitive childcare, maternal life-course and child functioning.[1–3] A subgroup analysis of poor unmarried teenage mothers in the first US trial (n=54) found verified maltreatment by age 2 years in 19% of 32 control children and 4% of the 22 children in the group in receipt of NFP during both pregnancy and infancy (mean % difference: 0.15, 95% confidence interval (CI) of −0.01 to 0.31).[1] Additionally, there was a 56% relative reduction in emergency department encounters for injuries and ingestions during the second year of life. For children with state-verified maltreatment reported by age 4, children in the NFP group had fewer risks for harm than the control group between 25 and 50 months of life. A trial of VoorZorg, the Dutch adaptation of NFP, found a reduction in child-protecting agency reports by age three years for families in the home-visiting arm.[4,5]

We evaluated short-term FNP programme outcomes to age 2 years in our Building Blocks trial (BB:0-2). [ref] We found no difference for four primary outcomes: maternal tobacco use in late pregnancy, birth weight of the baby, proportion of women with a second pregnancy within 24 months postpartum and emergency attendances and hospital admissions for the child within 24 months postpartum. There were some differences in favour of FNP for secondary child development outcomes including maternal-reported cognitive function and language at 24 months.[6] As the previous US trials showed benefit for maltreatment outcomes increasingly after age four we sought to establish whether FNP moderates maltreatment outcomes over a medium-term period. Through access to administrative data, we aimed to determine impact across a range of child maltreatment outcomes and key indicators of neglect (e.g. injuries and ingestions). We also sought to assess impacts on other programme relevant developmental and maternal outcomes as well as costs.

Objectives

Primary: to determine programme effectiveness in reducing objectively measured maltreatment , when compared with usually provided health and social care alone. Secondary: to determine programme effectiveness in reducing maltreatment, medium-term programme outcomes such as subsequent pregnancies, school readiness and educational outcomes, the impact of moderators of programme effect and the cost and consequences of the programme.

For peer review only - http://bmjopen.bmj.com/site/about/guidelines.xhtml

Methods

Study design, setting and participants

This study followed a cohort of mothers and children who had participated in the BB:0-2 trial for a further five years using administrative data only.[7] Data were retrieved from public sector providers and linked to the trial dataset using opt-out consent, following REC approval (14/WA10062) and s251 support (CAG 10-08(b)/2014). Children and mothers were followed up until the child reached Key Stage 1 teacher-based assessment (KS1) by which time most children were seven years old.

Building Blocks: 2-6 (BB:2-6) study participants were women and their first child (or twins, if relevant) who were not mandatorily withdrawn from the BB:0-2 trial; did not electively withdraw and/or did not opt-out of this follow-on study.[6,8] . BB:0-2 trial participants were eligible if women were nulliparous, aged 19 years or younger, were recruited at less than 25 weeks' gestation, living within the catchment area of a local FNP team, able to provide consent and speak English. Women expecting multiple births and those with a previous pregnancy ending in miscarriage, stillbirth, or termination were eligible. Women planning to have their child adopted or to move beyond the FNP catchment area for more than 3 months were not eligible. [6,7] Trial participants had been randomly assigned to FNP or usual care, with randomisation stratified by site and minimised by gestation (<16 weeks vs ≥16 weeks), smoking (yes vs no), and preferred language of data collection (English vs non-English) and weighted towards minimising the imbalance in trial groups with probability 0·8.

Intervention (BB:0-2 Trial)

FNP programme provides up to 64 home visits from specially trained family nurses during pregnancy until the child is two years old addressing personal and environmental health, life course development, maternal role, family and friends and access to health and social services. All trial participants received usually provided health and social care services. Participants in the usual care (UC) arm received these services alone.[6,8,9]

Procedure and data sources

The BB:0-2 trial consented 1618 eligible women between June 2009 and July 2010 and we concluded all BB:2-6 follow-up in May 2018. Maternal and child identifiers were sent to NHS Digital and the National Pupil Database (NPD) for matching with their databases. Maternal identifiers were also sent to the Department of Health and Social Care (DHSC). Matching with NHS Digital and DHSC used a combination of NHS number, date of birth, postcode and sex. NHS Digital provided a linked file to Office for National Statistics (ONS) for mortality data. Matching with NPD used exact matching on first name and surname, date of birth and postcode (of both mother and child for social care data; all other datasets, just child). All matched data were sent to a third-party data safe haven (Secure

For peer review only - http://bmjopen.bmj.com/site/about/guidelines.xhtml

Anonymised Information Linkage (SAIL) Databank) and linked by project identifiers to trial data and analysed via remote access.[10–12]

Outcomes

The primary outcome was Child in Need (CIN) status recorded at any time by a local department of children's social care services (CSCS) (and sourced from the NPD). This includes children assessed as unlikely to achieve or maintain a reasonable level of health or development, or whose health and development is likely to be significantly or further impaired, without the provision of services; or is a child who is disabled. Secondary outcomes were additional objective measures of maltreatment (referral to CSCS, child being on a Child Protection Plan, CIN categorisation, Looked after status (mother, child)); Associated measures of maltreatment (recorded injuries and ingestions, non-attendance rates for hospital appointments); Maternal outcomes (subsequent pregnancies); Child health, developmental and educational outcomes (special educational needs, early educational attendance and assessments (Early Years Foundation Stage Profile (EYFSP), KS1)); and health resource use. Study assessment domains, outcomes and principal data providers and analysis are described in appendix table S1.1.

For peer review only

Power calculation

Our sample size was fixed by the cohort available from BB:0-2. However, for CIN status, while UK data on rates are not specific to the age-range of interest, the rate per 10,000 general population aged 5-9 years is 4.6% for local authorities covering BB:0-2 trial sites.[13] The rate of CIN status would be expected to be greater in the specific study sample. Therefore, we assumed a rate of 8%, hypothesised that FNP would reduce the presence of CIN in the first six years and assumed a difference of 4% would be important. To detect a difference of 4% (4% vs 8%) would require 602 children in each arm with 80% power and a two-sided 5% alpha level. With 1562 children available for follow-up (i.e. excluding mandatory withdrawals) and follow up through medical records with 10% loss in tracking and linkage we would have 1405 participants, sufficient data to assess the primary outcome.

Efforts to address sources of bias

All maltreatment and child developmental outcomes were independently state-verified (social worker) reports or teacher-assessments respectively, recorded in administrative service data and abstracted from national data providers. By using administrative data and established linking fields we were able to minimise bias due to loss to follow-up. An a priori statistical analysis plan described primary, secondary (including sub-group) and sensitivity analyses, and any additional exploratory analysis is clearly highlighted.

Statistical analysis

Analysis was conducted on participants whose identifiers could be sent, linked and data released by the data providers. This excluded participants whose individual data could be used but could not be matched due to incorrect linking fields, other exclusions from health or education: e.g. private or home schooling and National opt-outs. Imbalance between the BB:0-2 and eligible BB:2-6 participants was quantified using descriptive statistics in baseline demographics, clinical and questionnaire data. We conducted all analyses on an intention-to-treat (ITT) basis with complete case population (with follow-up data). Therefore, participants remained in the BB:0-2 trial groups they were randomised to regardless of the intervention received. We used multilevel modelling to allow for clustering of effect within the 18 sites and family nurse level and both fitted as random effects. Where clustering was minimal at the family nurse level, results from the two-level model are presented. Parameter estimates are reported alongside a 95% CI and p-value. We adjusted for variables used in randomisation (smoking status, gestational age, language). We defined loss to follow up in this study as a child death or adoption, both of which we were able to determine. We excluded children from analyses of binary outcomes where a child had no event, and where follow-up was incomplete. With time-to-event analyses, children were censored at these events.

For peer review only - http://bmjopen.bmj.com/site/about/guidelines.xhtml
For peer review only

Primary comparative analysis examined whether the firstborn child(ren) ('BB:0-2') had ever been referred to CSCS and classed as a child in need at any point between birth and 6 years of age (appendix). For this binary outcome we used logistic multilevel modelling to investigate differences in the proportion defined as in need between the trial arms. We present the resulting estimate as an odds ratio. For binary and categorical outcomes, comparative analysis used logistic and multinomial modelling respectively with results presented as odds ratios (OR). For continuous outcomes we used linear regression and present mean differences. For count data we used Poisson regression modelling; where event distribution displayed signs of over dispersion a Negative Binomial regression model was used (or zero-inflated model if appropriate). Results are presented as incidence rate ratios (IRR). Time to event data used Cox regression modelling presented with hazard ratios (HR).

KS1 data were received in two tranches from NPD, firstly children assessed in 2016/17 academic year (received in May 2018) and then younger children who were assessed in 2017/18 (received May 2019). Reading, maths and science were examined for the whole cohort over both academic years. However, following changes made within the 2017/18 writing assessment (such that judgements in 2018 are not directly comparable to those made using the previous interim frameworks in 2016/2017), writing was only statistically assessed using 2016/17 data and the data from 2017/18 were presented descriptively. Sensitivity analyses for primary and selected secondary outcomes (EYFSP, KS1, referral to CSCS) included adjusting for hypothesised confounders at baseline, dosage effects (i.e. number of visits) using complier average causal effects (CACE) modelling and sub-group analysis of potential effect moderators (maternal deprivation, adaptive functioning, NEET (not in education, employment or training) status at recruitment, maternal age at recruitment, child sex) and mediators of programme effect (domestic violence at 24 months, maternal care status) as interaction terms in the main comparative models. Results of all planned sensitivity analyses are presented in the appendix (S7).

Economic Analysis

A cost consequences analysis of FNP over the full follow-up period (BB:0–2 and BB:2–6) took a secondary health care (UK NHS) perspective. The principal data source was Hospital Episode Statistics (HES) records (inpatient, outpatient, A&E), which were coded in Healthcare Resource Group (HRG) Grouper and matched to appropriate NHS Reference costs. Maternal and child resource use were costed separately and valued in £ sterling. Only direct healthcare costs were analysed. Indirect costs such as lost wages or childcare costs were not available within the routinely collected data. Where data were absent in HES records it was assumed no resource was incurred. No primary care data were available beyond 18 months postpartum. Costs were discounted back from year of event to baseline (2009/10) at an annual rate of 3.5% as described by Central Government's guidance on appraisal and evaluation.[14]

For peer review only

Data management

Small numbers were handled according to SAIL Databank rules where any cell counts under five were suppressed in reporting. Where abortion data are presented, we handled in accordance with the data sharing agreement with DHSC where counts less than 9 were suppressed. Data were analysed using Stata v16 and IBM SPSS v23.0.

Patient and public involvement

Activities involved four groups of young people (CASCADE Voices, Our Place, FNP graduates and ALPHA) . These activities contributed to three aspects of the study: data-linkage methods (eg to optimise acceptability of the proposed dissent model, use of data linkage and communication of these methods)[15], study outcomes (by seeking views on the importance of the study outcomes) and dissemination of findings (including best way to present results, method of dissemination).

Results

The study population following the BB:0-2 trial tracing and matching process is shown in Figure 1. Five women were ineligible and 78 were mandatory withdrawals mainly due to fetal or infant death and adoption. The 110 women who electively withdrew during the trial were given the opportunity to opt-out from further data usage, 16 of whom removed consent for any further contact. Thus 94 women who had electively withdrawn from BB:0-2 were included, and their details sent for tracing alongside the remaining 1,452 mothers (1,546 mother-child dyads in total) to update contact details to improve matching. One dyad was subsequently removed and the remaining 1,545 contacted by letter, email or text of whom eight dissented from having their records linked. 1,537 mothers and 1,547 children were sent to the NPD and NHS Digital for matching. These formed the population for the BB:2-6 study. Match rates for children were 98.3% (NHS Digital) and 97.4% (NPD).
Numbers matched to and returned for analysis differed by data source and population (Table 1). For selected maternal and child baseline variables and delivery of FNP assessed against programme fidelity goals, the BB:2-6 sample appears broadly representative of the original BB:0-2 cohort (Tables S4.1 and S4.2). Baseline characteristics of the BB:2-6 mothers and children were well balanced between trial arms (Table 2).

Table 1 Numbers (%) of mothers and children with data returned by dataset

	**Mothers**	**Children**
**Sent for matching to NPD and NHSD**	**N=1537**	**N=1547**
**National Pupil Dataset (NPD)**		
Not matched	1248 (81·2)	40 (2·6)
Match to any NPD dataset	289 (18·8)	1507 (97·4)

For peer review only - http://bmjopen.bmj.com/site/about/guidelines.xhtml

Pupil Level Annual School Census (PLASC)	<5	1498 (96·8)
Child in Need (CIN)	278 (18·1)	414 (26·8)
Children looked after (CLA)	61 (4·0)	52 (3·4)
Early years foundation stage profile (EYFSP)	N/A	1476 (95·4)
Key Stage one (KS1)	N/A	1472 (95·2)
Early Years Census (EYC)	N/A	642 (41·5)
Pupil referral unit (PRU) Census	<5	0
Alternative Provision (AP)	<5	<5
Absence	<5	1494 (96·6)
**NHS Digital**		
Not matched	36 (2·3)	27 (1·7)
Duplicate match	0	1 (0·06)
Matched to any NHSD dataset	1501 (97·7)	1519 (98·3)
Accident and Emergency (A&E)	1360 (88·5)	1392 (90·0)
Admitted Patient Care (Inpatients) (APC)	1498 (97·5)	1507 (97·4)
Outpatient (OP)	1495 (97·3)	1189 (76·9)
**Sent for matching to DHSC**	**N=1532**	**0**
Abortions dataset	1532 (100)	NA

Table 2 Socio-demographic characteristics of mother and babies at baseline by trial arm

	FNP<br>N=766	Usual care<br>N=771
**Maternal age at recruitment (years)**	17·9 (17·0 to 18·8)	17·9 (16·9 to 18·8)
**Ethnicity**		
White background	675 (88·1%)	680 (88·2%)
Mixed background	44 (5·7%)	38 (4·9%)
Asian background	15 (2·0%)	10 (1·3%)
Black background	29 (3·8%)	40 (5·2%)
Chinese or Other background	<5	<5
**Relationship status with baby's father**		
Married	6 (0·8%)	10 (1·3%)
Separate	72 (9·4%)	78 (10·1%)
Closely involved/boyfriend	582 (76·0%)	586 (76·0%)
Just friends	106 (13·8%)	97 (12·6%)
**NEET Status***		
Yes	315/764 (41·1%)	319/769 (41·4%)
No	345/764 (45·0%)	333/769 (43·2%)
**IMD Overall Score†**	38·1 (24·6 to 52·6)‡	38·1 (25·5 to 51·6)§

For peer review only - http://bmjopen.bmj.com/site/about/guidelines.xhtml

	FNP<br>N=766	Usual care<br>N=771
**Generalised self-efficacy scale (score 10 to 40)¶**	30·0 (28·0 to 33·0)‖	30·0 (27·0 to 32·0)**
**Adaptive functioning:**		
**Difficulty in at least one basic skill**		
Yes	213/765 (27·8%)	184/770 (23·9%)
No	552/765 (72·2%)	586/770 (76·1%)
**Had 3 or less life skills (out of 5)**		
Yes	185/763 (24·2%)	218/769 (28·3%)
No	578/763 (75·8%)	551/769 (71·7%)
**At least one burden**		
Yes	214/759 (28·2%)	237/767 (30·9%)
No	545/759 (71·8%)	530/767 (69·1%)
**Cigarette smoking participant self-reported**		
Ever smoked		
Yes	615 (80·3%)	612 (79·4%)
No	151 (19·7%)	159 (20·6%)
**Child characteristics**	**FNP<br>N=773**	**Usual Care<br>N=774**
**Sex**		
Male	381/773 (49·3)	406/773 (52·5)
Female	392/773 (50·7)	367/773 (47·5)
**Birthweight (grams)** Mean (SD)	3223·81 (606·0)††	3215·52 (555·56)‡‡
**NNU admission (direct or subsequent)**		
Yes	76/716 (10·6)	66/749 (8·8)
No	640/716 (89·4)	683/749 (91·2)

Data are n (%), n/N (%), or median (25th to 75th centile) unless otherwise stated. <5 = numbers suppressed.
FNP = Family Nurse Partnership Programme + Usual Care. IMD = Index of Multiple Deprivation. SD= standard deviation. NNU = Neonatal unit. *Definition of NEET status: Not in education employment or training (applicable only to those whose age at end of previous academic year at time of baseline interview was >16). †Higher IMD score indicated more deprivation. Mean IMD Score for England in 2010 was 21.67 [Wilkinson DL, Sniehotta FF, Michie S. Targeting those in need: Baseline data from the first English National Health Service (NHS) health trainer service: *Psychology, health & medicine* 2011; **16**(6): 736-48], ‡N=760. §N=765. ¶ Higher score indicates higher level of self-efficacy. ‖N=764. **N=769. ††N=724. ‡‡N=752.

For the primary outcome of a child in need, 277 referrals resulting in no further action were excluded and 634 referrals remained associated with a CIN status (appendix). Overall, 323 (21.5%) of children in the BB:2-6 cohort had at least one referral to CSCS which resulted as being classed as a child in need at any point between pregnancy (includes pre-birth CIN) and 6 years of age. No difference in rate of children in need was found between FNP (21.2%) and UC (21.7%) arms for the 1,506 children in the analysis (adjusted OR=0.98, 95% CI=0.74, 1.31) (Table S6.1). The parameter estimate was not

For peer review only - http://bmjopen.bmj.com/site/about/guidelines.xhtml

affected when adjusting for the number of FNP visits received (efficacy per visit OR=1.00, 95% CI=0.99, 1.004).

There was no difference for any other maltreatment outcome. This included the secondary outcomes of referral to CSCS, the age at which the first referral was made or the age at which the child was classified as in need, the proportion of children with a protection plan. A small number of children were ever Looked After within the 6-year follow-up period, with no evidence of a difference between trial arms (Table S6.1). However, the UC arm experienced on average two more months in care compared to the FNP arm. The total number of child deaths across the full follow-up period was less than ten and not further reported by study arm, consistent with non-disclosive data management policy. Data informing the disability outcome were not available for the years requested. In addition, no difference was found when examining age at first referral or CIN by study arm.

No between arm differences were found in children not taken to at least one outpatient appointment (did not attend rate), emergency attendances or hospital admissions for an injury or an ingestion (Table 3). Overall, 78% of mothers had a subsequent pregnancy (using inpatients, outpatients and abortions data) with no differences by arm (Table 4). Similarly, just over two thirds of the cohort had at least one registerable birth (using only admission data as a source) during the follow-up period with a comparable inter-birth interval.

For peer review only

Table 3 Associated measures of maltreatment secondary outcomes

Outcome	FNP N=760	Usual care N=759	Adjusted* parameter estimate (95% CI)	p-value	Absolute risk difference (FNP-Usual care) (95% CI)
**Injuries and Ingestions**					
**Emergency attendance†**					
None	317 (41·7%)	344 (45·3%)	Reference		
At least one	443 (58·3%)	415 (54·7%)	1·17‡ (0·95 to 1·45)	0·149	3·6% (-1·4 to 8·6%)
Number of attendances per child	2 (1 to 2)	2 (1 to 3)	1·09§ (0·93 to 1·28)	0·281	
**Admission to hospital**					
None	671 (88·3%)	660 (87·0%)	Reference		
At least one	89 (11·7%)	99 (13·0%)	0·87‡ (0·63 to 1·20)	0·407	-1·3% (-4·7 to 2·0%)
N unique admissions	109	119			
Ratio admissions: children	1·22	1·20			
Number of admissions per child	1 (1 to 1)	1 (1 to 1)	0·93§ (0·67 to 1·29)	0·663	
Length of hospital stay (days)	0·5¶ (0·5 to 1·0)	0·5 (0·5 to 1·0)	*NT*		
**Attendance and/or admission**					
None	306 (40·3%)	324 (52·7%)	Reference		
At least one	454 (59·7%)	435 (57·3%)	1·11‡ (0·89 to 1·37)	0·351	2·4% (-2·5 to 7·4%)
**DNA outpatient appointment**	**N=580**	**N=577**			
Attended all appointments	290 (50·0%)	289 (50·1%)	Reference		
DNA at least once	290 (50·0%)	288 (49·9%)	1·00‡ (0·79 to 1·26)	0·997	0·09% (-5·6 to 5·8%)

Data are n (%), n/N (%), or median (25th to 75th centile) unless otherwise stated. <5 = numbers suppressed· FNP = Family Nurse Partnership Programme + Usual Care.
* FNP compared to usual care. Analysis adjusted for stratification (site), minimisation variables (gestational age, smoking status at recruitment, and first or preferred language); † Using diagnosis in any position; ‡ Odds ratio from logistic model; § Incidence rate ratio from Poisson model; ¶ 0·5 days indicates an admission and discharge on the same day (could be up to 1 day in hospital).
Source: National Pupil Database, The Department for Education

For peer review only

Table 4 Subsequent pregnancies and registerable birth

	FNP	Usual care	Adjusted* parameter estimate (95% CI)	p-value	Absolute risk difference (FNP-Usual care) (95% CI)
**Subsequent pregnancy**	**N=753**	**N=753**			
No	163 (21·7%)	163 (21·7%)	Reference		
Yes	590 (78·4%)	590 (78·4%)	1·00† (0·79 to 1·28)	0·984	0·0% (-4·2 to 4·2%)
**Subsequent registerable birth**	**N=752**	**N=749**			
No	276 (36·7%)	266 (35·5%)	Reference		
Yes	476 (63·3%)	483 (64·5%)	0·95† (0·77 to 1·18)	0·662	-1·2% (-6·0 to 3·7%)
One birth	326 (68·5)	331 (68·5)	0·95‡ (0·76 to 1·19)	0·655	
Two	124 (26·1)	121 (25·1)	0·99‡ (0·73 to 1·34)	0·955	
Three births or more	26 (5·5)	31 (6·4)	0·80‡ (0·46 to 1·39)	0·435	
**Inter-birth interval between first and second child (days)**					
Median (25th to 75th centiles)	1027 (590 to 1506·75)	1065 (665 to 1538)	0·99§ (0·88 to 1·13)	0·938	

Data are n (%), n/N (%), or median (25th to 75th centile) unless otherwise stated. <5 = numbers suppressed· FNP = Family Nurse Partnership Programme + Usual Care.
* FNP compared to usual care. Analysis adjusted for stratification (site), minimisation variables (gestational age, smoking status at recruitment, and first or preferred language); † Odds ratio from logistic model; ‡ Relative risk ratio from multinomial model; § Hazard ratio from Cox model.
Source: National Pupil Database, The Department for Education

For peer review only

Some secondary outcomes provided evidence of positive programme impacts (Table S6.2). These were found in educational outcomes assessed at the end of the reception year and at KS1 (by which time the child would be seven years old). Children in the FNP arm were more likely to reach a good level of development across all five areas of learning by the end of the reception year (58.0%) than children in the UC arm (52.2%) and also to achieve a good level of development in all 17 early learning goals (FNP: 55.5%, UC: 50.1%). The small advantage for children of nurse-visited mothers was consistent across all five areas of learning although not tested statistically. For KS1 reading, writing, maths and science assessments there was no evidence of a difference between arms in the proportion of children reaching at least the expected standard nor specifically in those working at the expected level or at a greater depth of knowledge (Table S6.2). What is observed from the writing assessment is the increase in the rates of children reaching at least the expected standard between the two academic years (Overall 45.7% in 2016/17 to 66.5% in 2017/18), reflective of all assessments across the two academic years in each arm (appendix table S6.3). This is mainly explained by the distribution of births for the children for each academic year. Reflecting the recruitment period of the trial, children with KS1 results available in the academic year 2016/17 were born between October 2009 and August 2010 but skewed towards the summer born children (42% born between June and August 2010) whereas children appearing in the 2017/18 academic year were born between September 2010 and February 2011 (**Error! Reference source not found.** figure S6.4). There is a nationally observed relationship between rates of children reaching the expected standard and month of birth e.g. a 17% difference in reading for children born in August and September.[16] Therefore, our 2017/18 cohort of children do not represent the whole academic year, but are biased towards those that are more likely to achieve. This indicates that the rates of children reaching the expected standard varies by their month of birth and is important as a moderator of programme effect. Importantly, the original trial allocation ensured study arms remained balanced with regards month of birth and the intervention had no impact on total weeks gestation.[6] As a sensitivity analysis, the main analyses were adjusted for the child's month of birth (categorised into quarter of birth) and evidence of a between arm difference was found in reading assessments. In addition, the Early Years assessments were adjusted for child's month of birth, which strengthened the association found in Table 6 (appendix table S6.5).

Cost

There were negligible resource use and cost differences for both mothers (p=0.393) and children (p=0.865) between study arms (Table S10.1). The adjusted incremental costs of programme delivery per women in BB:0-2 (£1,811) remain the key observed cost difference between study arms.

For peer review only - http://bmjopen.bmj.com/site/about/guidelines.xhtml

Discussion

In this data linkage study we found that adding FNP to usually provided health and social care provided no additional benefit for maltreatment outcomes including the numbers of children referred to CSCS, registered as in need of additional support, given a child protection plan, entering care, attending an emergency department or being admitted for an injury or ingestion, or duration of stay if admitted. The total number of child deaths found was less than ten and therefore disclosive and cannot be reported. High rates of children ever registered as in need in both study arms indicates substantial need amongst families. We found the programme led to more children reaching a good level of development at the end of school reception year and, when taking month of birth into account, improvements in reading at KS1. Writing scores improved as a result of FNP for boys, children of younger mothers and of mothers who were not in education, employment or training when first recruited to the study. We found no other difference between families who received FNP and those who did not. With no differences in secondary health resource use and costs for FNP, the programme can be considered cost-neutral compared to usual care in the medium-term (2-6 years).

The prioritisation of outcomes in our original trial attracted some debate.[6,17–19] However, the trial and the current follow-on study are both comprehensively reported, and preceded by a priori analysis plans providing transparency and guarding against undue post hoc interpretation.[6,7,20] Although a primary outcome of child being in need of additional support was incorporated into the study design, we adopted a multimethod multisource approach to look at the range of maltreatment outcomes in drawing our conclusions. The consistent pattern of results within the two principal outcome domains provides greater confidence in what we can conclude.

The available maltreatment data provides an essential overview of family experience as recorded in official records but may lack some depth which could have allowed a more nuanced understanding for children referred. The educational data are teacher-reported statutory tests but similarly provide less detailed insight into child functioning which research instruments may have provided. The lack of detailed social care and any primary care resource use data limited the perspective of the economic analysis. However, using administrative data has enabled comprehensive cohort follow-up, with few cases lost for analysis preserving the benefits of the original trial randomisation. The data included are also objective, allow for easy comparison nationally and over time. Our approach has gathered more data at less cost than comparable prospective data collection and minimised family burden.

The strongest evidence for the programme preventing maltreatment remains from the original US Elmira trial.[21,22] While the programme reduced the number of substantiated reports of abuse and neglect in the first 15 years of the child's life, the advantage emerged after age four years.[22] We found

For peer review only - http://bmjopen.bmj.com/site/about/guidelines.xhtml

differences in duration spent in care for looked after children, but the numbers involved are small and across the range of maltreatment outcomes the pattern is of no overall group difference. The programme was no more effective in tested sub-groups. It is worth recognising differences in the population of clients in receipt of the programme across the different study populations, their social circumstances (which also may have changed over time) and how that may determine potential to benefit from FNP.[9] We have not directly explored differences in social care systems between England and the US but these may vary the underlying risk of maltreatment and its likelihood of detection.[23]

School entry reading and maths is of important predictive value for later school achievement.[24] Early childhood educational investment programmes typically show a modest impact upon cognitive and achievement scores (eg weighted average of 0.21 standard deviations) with some decline in observed impact being attributed to general improvements in both school and home environments.[25,26] The question of comparator is therefore of particular relevance. In this study, a range of state and third sector funded services (including midwifery and health visiting) would have been available as usual care, possibly limiting the incremental benefit possible by the family nurse.[9] Nevertheless, women allocated to FNP received a mean of 9.71, 18.63 and 13.22 valid family nurse visits per programme phase (pregnancy, infancy, toddlerhood).

Home visiting interventions, which aim to impact upon a range of outcomes, have shown similarly modest effects on child cognitive outcomes (eg mean ES 0.25, 96% CI 0.11, 0.38) with considerable variability due to programme composition (eg greater effects with programmes addressing parental responsiveness, sensitivity to care and nurturing and rehearsal and role-playing).[27] Our BB:0-2 study found benefits at age two years for children in the FNP arm for both language and developmental outcomes but these outcomes were maternally reported and potentially subject to reporting bias in the open trial.6 This new objectively obtained medium-term evidence for programme impact upon developmental outcomes adds to that picture, although the size of effect remains small. Similar medium-term programme impact (to age six years old) on developmental outcomes (eg, intellectual functioning and receptive language scores) was reported from the Memphis trial.[28] A reanalysis of those data found cognitive benefits at age 6 years attributed to both programme induced improvements in maternal traits and family life investments at age 2.[29]

Given small but positive findings for developmental outcomes, tracking the cohort through to subsequent school years is warranted. As social care data are equally accessible via the NPD there is value in observing the trajectory for maltreatment outcomes given that the principal maltreatment findings from the US Elmira trial are located in the 15-year follow-up. Linkage to data in other sectors such as welfare would provide a greater understanding of broader impact for families. Such work should be driven by the programme’s logic model, existing evidence for long-term programme

For peer review only - http://bmjopen.bmj.com/site/about/guidelines.xhtml

impact, informed by programme evolution in England and supplemented by work to understand what may explain differing programme benefits across national settings.[22,30]

Conclusions

There are no evident programme benefits for maltreatment outcomes by age six but the FNP generates higher rates of child attainment at the end of the reception year and at KS1. The adapted programme remains locally commissioned and delivered in England.[30,31] Local needs and priorities may determine the weight attached to these different sets of outcomes.

For peer review only

Acknowledgements

The Centre for Trials Research receives funding from Health and Care Research Wales and Cancer Research UK. The work was undertaken with the support of The Centre for the Development and Evaluation of Complex Interventions for Public Health Improvement (DECIPHer), a UKCRC Public Health Research Centre of Excellence. Joint funding (MR/KO232331/1) from the British Heart Foundation, Cancer Research UK, Economic and Social Research Council, Medical Research Council, the Welsh Government and the Wellcome Trust, under the auspices of the UK Clinical Research Collaboration, is gratefully acknowledged.

Data re-use statement: Copyright © (2019), the Health and Social Care Information Centre. Re-used with the permission of the Health and Social Care Information Centre. All rights reserved.

We would like to thank all members of CASCADE voices, ALPHA and Our Place for contributing to the study including those who co-ordinated access to these individuals. Thanks are due to the FNP teams in Bristol, Bath and Wiltshire who helped us to make contact with FNP clients and graduates during our PPI work. We would also like to acknowledge the contribution of members of the study steering committee - Ann Louise Kinmonth (Chair), David Low, Gordon Taylor, Nina Biehal, Rachel Tonkin, Stavros Petrou, and Silvia van den Heijkant.

This work uses data provided by patients and collected by the NHS as part of their care and support as well as data from schools and local authorities. Using patient and other public data is vital to improve health and care for everyone. There is huge potential to make better use of information from people's patient records, to understand more about disease, develop new treatments, monitor safety, and plan NHS services. Patient and other public data should be kept safe and secure, to protect everyone's privacy, and it's important that there are safeguards to make sure that it is stored and used responsibly. Everyone should be able to

find out about how patient data is used. #datasaveslives You can find out more about the background to this citation here: https://understandingpatientdata.org.uk/data-citation. [To note. This has been adapted to include other data sources not made available from the NHS such as education and social services data].

Footnotes

Author contributions: MR was the chief investigator and acts as guarantor for the study. All authors contributed to study design. FLW was the study manager. RCJ was the lead statistician. DF supervised the health economics. All authors were responsible for study management. GM and LA were the data managers. JK and JS led for public involvement. GM and LA cleaned the data. RCJ designed and conducted the analysis. RP conducted the economic analysis. MR, JSa, RCJ, SC, KH, JK, EOJ, JSe were co-applicants on the original funding application. All authors interpreted the results of the analysis. RC-J and RP were responsible for producing the tables. MR, FLW, RC-J and RP drafted the study report. All authors reviewed manuscript drafts, revised for important intellectual content and approved the final version.

Funding: This project was funded by the National Institute for Health Research Public Health Research (NIHR PHR) Programme (reference:11/3002/11). The views and opinions expressed therein are those of the authors and do not necessarily reflect those of the NIHR PHR Programme or the Department of Health. The funders had no role in considering the study design or in the collection, analysis, interpretation of data, writing of the report, or decision to submit the article for publication.

Competing interests: All authors have completed the ICMJE uniform disclosure form at www.icmje.org/coi_disclosure.pdf and declare: all authors declare financial support from NIHR PHR for the submitted work; no financial relationships with any organisations that might have an interest in the submitted work in the previous three years; no other relationships or activities that could appear to have influenced the submitted work.

Ethical approval: Ethical approval for this study was granted by the Health Research Authority research ethics committee (Wales REC 3; REC reference 14/WA10062) and section 251 support was provided by the Confidentiality Advisory Group (CAG reference CAG 10-08(b)/2014).

Data sharing: Anyone wishing to access the linked datasets for research purposes should apply via the CAG to the Health Research Authority to access patient identifiable data without consent and then to the NHS Digital, National Pupil Database and the abortion statistics – Department for Health and

For peer review only - http://bmjopen.bmj.com/site/about/guidelines.xhtml

Social Care. In the first instance, enquiries about access to the data should be addressed to Professor Mike Robling, Centre for Trials Research, Cardiff University.

The lead author (MR) affirms that the manuscript is an honest, accurate, and transparent account of the study being reported; that no important aspects of the study have been omitted; and that any discrepancies from the study as planned (and, if relevant, registered) have been explained.

Dissemination to participants and related public communities: The findings from the BB:2-6 study, including those from this paper will be disseminated directly to participants where valid contact details remain available, via social media and conference presentations. This will include the use of lay-informed infographic materials. Summary results will also be made available on the study webpage [insert link].

References

1. Olds DL, Henderson CR, Chamberlin R, Tatelbaum R. Preventing Child Abuse and Neglect: A Randomized Trial of Nurse Home Visitation. Pediatrics. 1986;78(1) [accessed 2017 May 5]. Available from: http://pediatrics.aappublications.org/content/pediatrics/78/1/65.full.pdf
2. Kitzman H, Olds DL, Henderson CR, Hanks C, Cole R, Tatelbaum R, et al. Effect of Prenatal and Infancy Home Visitation by Nurses on Pregnancy Outcomes, Childhood Injuries, and Repeated Childbearing. JAMA. 1997;278(8):644 [accessed 2017 May 5]. Available from: http://jama.jamanetwork.com/article.aspx?doi=10.1001/jama.1997.03550080054039
3. Olds DL, Robinson J, O'brien R, Luckey DW, Pettitt LM, Henderson CR, et al. Home Visiting by Paraprofessionals and by Nurses: A Randomized, Controlled Trial. Pediatrics. 2002;110(3) [accessed 2017 May 5]. Available from: http://pediatrics.aappublications.org/content/110/3/486.short
4. Mejdoubi J, Van Den Heijkant SCCM, Van Leerdam FJM, Heymans MW, Crijnen A, Hirasing RA. The effect of VoorZorg, the dutch nurse-family partnership, on child maltreatment and development: A randomized controlled trial. PLoS One. 2015;10(4).
5. Olds DL, Kitzman H, Anson E, Smith JA, Knudtson MD, Miller T, et al. Prenatal and infancy nurse home visiting effects on mothers: 18-year follow-up of a randomized trial. Pediatrics. 2019;144(6) [accessed 2020 Jul 23]. Available from: https://pediatrics.aappublications.org/content/144/6/e20183889
6. Robling M, Bekkers MJ, Bell K, Butler CC, Cannings-John R, Channon S, et al. Effectiveness of a nurse-led intensive home-visitation programme for first-time teenage mothers (Building Blocks): A pragmatic randomised controlled trial. Lancet. 2016;387:146–55. Available from: http://www.thelancet.com/journals/lancet/article/PIIS0140-6736(15)00392-X/fulltext

For peer review only

7. Lugg-Widger F, Cannings-John R, Channon Sue, Fitzsimmons D, Hood K, Jones K, et al. Assessing the medium-term impact of a home-visiting programme on child maltreatment in England: protocol for a routine data linkage study. BMJ Open. 2017;7(e015728) [accessed 2017 Jul 17]. Available from: http://bmjopen.bmj.com/content/bmjopen/7/7/e015728.full.pdf

8. Owen-Jones E, Bekkers M-J, Butler CC, Cannings-John R, Channon S, Hood K, et al. The effectiveness and cost-effectiveness of the Family Nurse Partnership home visiting programme for first time teenage mothers in England: a protocol for the Building Blocks randomised controlled trial. BMC Pediatr. 2013;13:1.

9. Robling M, Cannings-John R, Channon S, Hood K, Moody G, Poole R, et al. What is usual care for teenagers expecting their first child in England? A process evaluation using key informant mapping and participant survey as part of the Building Blocks randomised controlled trial of specialist home visiting. BMJ Open. 2018;8(5).

10. Lyons RA, Jones KH, John G, Brooks CJ, Verplancke J-P, Ford D V, et al. The SAIL databank: linking multiple health and social care datasets. BMC Med Inform Decis Mak. 2009;9(9) [accessed 2017 May 12]. Available from: http://www.biomedcentral.com/1472-6947/9/3

11. Jones KH, Ford D V., Jones C, Dsilva R, Thompson S, Brooks CJ, et al. A case study of the Secure Anonymous Information Linkage (SAIL) Gateway: A privacy-protecting remote access system for health-related research and evaluation. J Biomed Inform. 2014;50:196–204 [accessed 2017 May 12]. Available from: http://www.sciencedirect.com/science/article/pii/S1532046414000045

12. Jones KH, Ford DV, Thompson S, Lyons R. A Profile of the SAIL Databank on the UK Secure Research Platform. Int J Popul Data Sci. 2019;4(2) [accessed 2020 Jun 30]. Available from: https://ijpds.org/article/view/1134

13. Department for Education. Children In Need in England, including their characteristics and further information on children who were the subject of a child protection plan (2009-10 Children in Need census, Final. 2010.

14. HM Treasury. The Green Book, Central Government Guidance on Appraisal and Evaluation. 2020. [accessed 2020 Dec 7]. Available from: https://assets.publishing.service.gov.uk/government/uploads/system/uploads/attachment_data/file/938046/The_Green_Book_2020.pdf

15. Lugg-widger F, Cannings-john R, Angel L, Moody G, Segrott J, Kenkre J, et al. Assessing the impact of specialist home visiting upon maltreatment in England : a feasibility study of data linkage from a public health trial to routine health and social care data. Pilot feasibility Stud. 2018;4(1):98.

16. Department for Education. Phonics screening check and Kay stage 1 assessments in England, 2016 (SFR 42/2016). 2016.

For peer review only - http://bmjopen.bmj.com/site/about/guidelines.xhtml

17. Olds D. Building evidence to improve maternal and child health. Vol. 387, The Lancet. Lancet Publishing Group; 2016. p. 105–7 [accessed 2020 Jul 23]. Available from: http://dx.doi.org/10.1016/S0140-6736

18. Barlow J, Barnes J, Sylva K, Fonagy P, Fearon P. Questioning the outcome of the Building Blocks trial. Vol. 387, The Lancet. Lancet Publishing Group; 2016. p. 1615–6 [accessed 2020 Jul 23]. Available from: http://www.dh.gov.uk/

19. Robling M, Torgerson D, Pickett K, Channon S, Sanders J. Questioning the outcome of the Building Blocks trial - Authors' reply. Vol. 387, The Lancet. Lancet Publishing Group; 2016. p. 1616–7 [accessed 2020 Jul 23]. Available from: http://www.dh.gov.uk/

20. Robling M, Bekkers MJ, Bell K, Butler CC, Cannings-John R, Channon S, et al. The Building Blocks Trial (Main report). 2014. Available from: https://www.cardiff.ac.uk/__data/assets/pdf_file/0009/504729/Building-Blocks-Full-Study-Report.pdf

21. Zielinski DS, Eckenrode J, Olds DL. Nurse home visitation and the prevention of child maltreatment: Impact on the timing of official reports. Dev Psychopathol. 2009;21(2):441–53 [accessed 2019 Aug 2]. Available from: https://www.cambridge.org/core/product/identifier/S0954579409000248/type/journal_article

22. Olds DL, Eckenrode J, Henderson CR, Kitzman H, Powers J, Cole R, et al. Long-term Effects of Home Visitation on Maternal Life Course and Child Abuse and Neglect Fifteen-Year Follow-up of a Randomized Trial. JAMA. 1997;278(8):637–43.

23. Moody G, Cannings-John R, Hood K, Kemp A, Robling M. Establishing the international prevalence of self-reported child maltreatment: A systematic review by maltreatment type and gender. Vol. 18, BMC Public Health. BioMed Central Ltd.; 2018. p. 1164 [accessed 2020 Jul 23]. Available from: https://bmcpublichealth.biomedcentral.com/articles/10.1186/s12889-018-6044-y

24. Duncan GJ, Dowsett CJ, Claessens A, Magnuson K, Huston AC, Klebanov P, et al. School Readiness and Later Achievement. Dev Psychol. 2007;43(6):1428–46.

25. Duncan G, Magnusson K. Early childhood interventions for low-income children. Focus (Madison). 2014;31.

26. Duncan G, Magnuson K. Investing in Preschool Programs. J Econ Perspect. 2013;27(2):109–31.

27. Filene JH, Kaminski JW, Valle LA, Cachat P. Components associated with home visiting program outcomes: A meta-analysis. Pediatrics. 2013;132(SUPPL.2):S100–9 [accessed 2021 Jan 8]. Available from: www.pediatrics.org/cgi/doi/10.1542/peds.2013-1021H

28. Olds DL, Kitzman H, Cole R, Robinson J, Sidora K, Luckey DW, et al. Effects of Nurse Home-Visiting on Maternal Life Course and Child Development: Age 6 Follow-Up Results of a Randomized Trial. Pediatrics. 2004;114(6):1550–9. Available from:

For peer review only - http://bmjopen.bmj.com/site/about/guidelines.xhtml

http://pediatrics.aappublications.org/cgi/doi/10.1542/peds.2004-0962

29. Heckman JJ, Holland ML, Makino KK, Pinto R, Rosales-Rueda M. An Analysis of the Memphis Nurse-Family Partnership Program. NBER Work Pap. 2017;23610. Available from: http://www.nber.org/papers/w23610%0Ahttp://www.nber.org/papers/w23610

30. Family Nurse Partnership National Unit & Dartington Service Design Lab. FNP ADAPT: Using evidence, pragmatism and collaboration to change the FNP programme in England. London, UK; 2020. Available from: www.fnp.nhs.uk

31. The Family Nurse Partnership |. [accessed 2020 Jul 23]. Available from: https://www.fnp.nhs.uk/

For peer review only

Figure 1 Flow chart

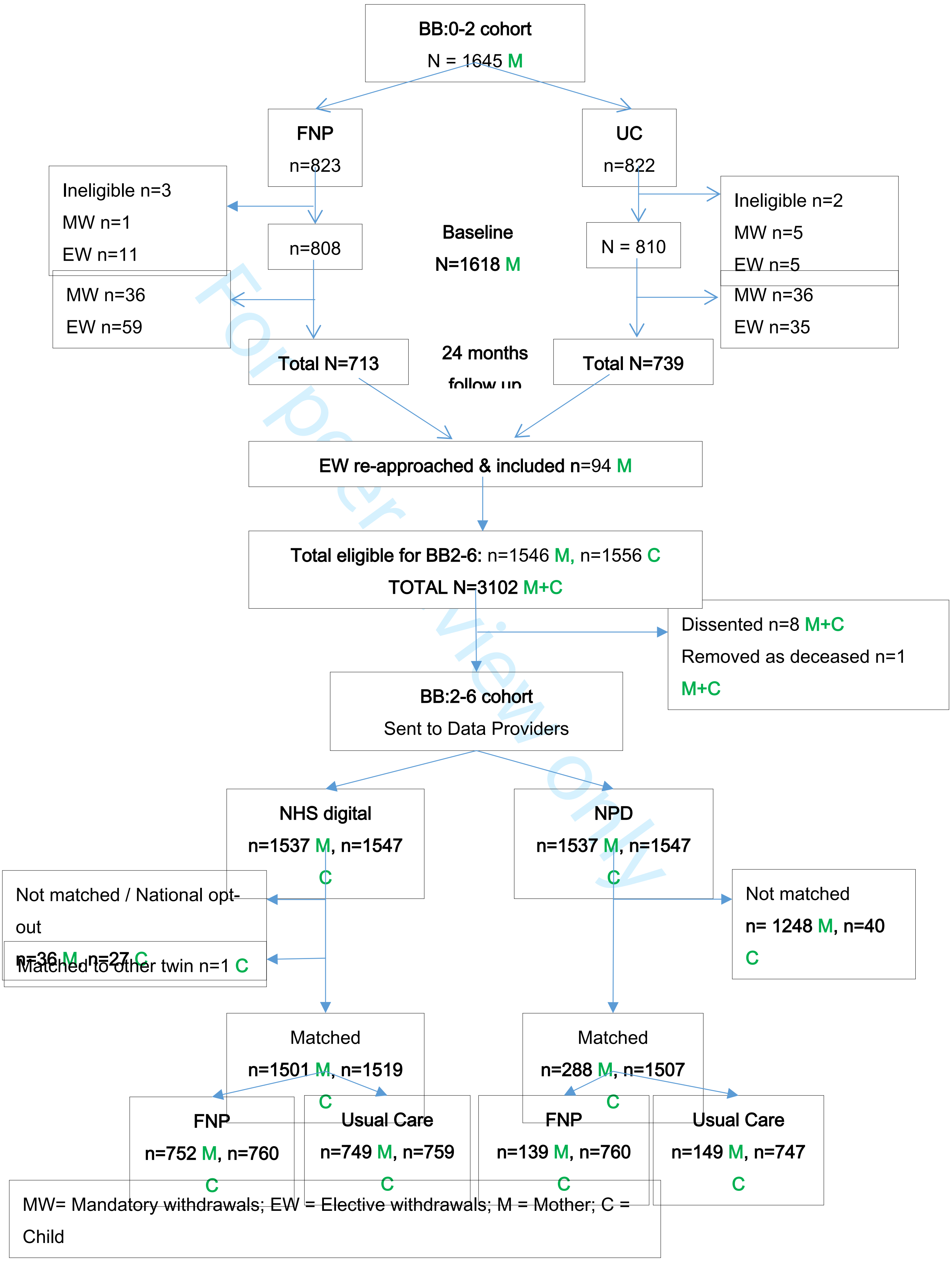

For peer review only - http://bmjopen.bmj.com/site/about/guidelines.xhtml

For peer review only

Supplementary appendix

1. Study assessment domain, outcomes and principal data providers
   - Table S1.1 BB:2-6 Study assessment domains, key and exploratory outcomes and principal data providers

2. Sample size calculation

3. Further details of Child in Need outcome

4. Representativeness of study sample
   - Table S4.1 Maternal characteristics of those in BB:0-2 compared to those in the BB:2-6
   - Table S4.2 Child characteristics of those in the BB:0-2 study compared to those in the BB:2-6 study

5. Number of valid FNP visits received
   - Table S5.1 BB:2-6 study population data: Valid FNP visits per phase

6. Primary outcomes, secondary outcomes and additional Key Stage 1 results

   - Table S6.1 Objective measures of maltreatment: primary and secondary outcomes
   - Table S6.2 Child health, developmental and educational secondary outcomes
   - Table S6.3 Proportion of children reaching the expected standard in KS1 assessments by trial arm and academic year
   - Figure S6.4 Distribution of children's month of birth by trial arm
   - Table S6.5 Comparison of parameter estimates before and after adjustment for child's month of birth

7. Results of planned sub-group analysis
   - Table S7.1 Subgroup analyses for referrals to Children's social care services
   - Table S7.2 Subgroup analyses for child in need status
   - Table S7.3 Predefined subgroup analyses on Early Years Assessment total point score
   - Table S7.4 Subgroup analyses on KS1 assessments reaching the expected standard (RES)
   - Table S7.5 Subgroup analyses on reaching the expected standard in the writing KS1 assessment for 2016/17 academic year

8. Treatment efficacy

9. Missing data

10. Cost Consequence Analysis
   - Table S10.1 Cost-consequence balance sheet for the family-nurse based intervention for the four years between children's ages 2 and 6.

For peer review only

1. Study assessment domain, outcomes and principal data providers

Table S1.1 BB:2-6 Study assessment domains, key and exploratory outcomes and principal data providers

Domains	Outcomes	Maternal	Child	HES	ONS	NPD	Data source	Analysis
Primary: Child in Need status recorded at any time during the follow-up period.	CIN status		✓			✓	CIN	LO
	Exploratory:<br>*Age at first CIN referral*<br>*Number of CIN referrals per child*		 ✓<br>✓			 ✓<br>✓	 CIN<br>CIN	 *SURV*<br>*PO*
Secondary:<br>(i) Objective measures of maltreatment	CIN categorisation<br>CIN duration		✓<br>✓			✓<br>✓	CIN<br>CIN	MNOM<br>SURV
	Referral to Children's social care services		✓			✓	CIN	LO
	Exploratory: Age at first referral		✓			✓	CIN	SURV
	Child Protection Plan<br>Child Protection Plan categorisation		✓<br>✓			✓<br>✓	CIN<br>CIN	LO<br>MNOM
	Looked after status<br>CLA period of care<br>Legal status of CLA		✓<br>✓<br>✓			✓<br>✓<br>✓	CLA<br>CLA<br>CLA	LO<br>PO<br>D
	Cause of death		✓		✓		ONS- deaths	D
(ii) Associated measures of maltreatment	Injuries and ingestions:<br>Emergency attendance<br>Admission to hospital<br>Attendance and/or admission<br>DNA appointments		 ✓<br>✓<br>✓<br>✓	 ✓<br>✓<br>✓<br>✓			 A&E<br>IP<br>A&E, IP<br>OP	 LO<br>LO<br>LO<br>LO
	Exploratory:<br>*Number of admissions per child*<br>*Length of hospital admission stay*		 ✓<br>✓	 ✓<br>✓			 IP<br>IP	 PO<br>D
(iii) Maternal outcomes	Subsequent pregnancy	✓		✓			OP, IP, Ab	LO
	Exploratory:							

For peer review only - http://bmjopen.bmj.com/site/about/guidelines.xhtml

Domains	Outcomes	Maternal	Child	HES	ONS	NPD	Data source	Analysis
	Number of births	ü		ü			IP	PO
	Inter-birth interval between first and second child	✓		✓			IP	SURV
(iv) Costs	Health and Social Care resource use	✓	✓	✓		✓	OP, IP, A&E matched with NHS reference costs	D
(v) Child health, developmental and educational outcomes	Special educational needs provision		✓<br>✓	✓		✓	PLASC, AP, PRU Census, EYC	LO
	Disability		✓			✓	NA	NA
	Early education attendance		✓			✓	PLASC, EYC	LO
	School attendance		✓			✓	Absence	LO
	Early Years assessment		✓			✓	EYFSP	LIN, LO
	KS1 attainment					✓	KS1	LO
	Exploratory:							
	Early education attendance by School or an Ofsted registered PVI establishment		✓			✓	PLASC, EYC	D
	Earliest type of day care		✓			✓	EYC	D

Ab - Abortions; A&E – Accident and Emergency; AP – Alternative Provision; CIN – Child in Need; CLA – Child Looked After; D – Descriptive; DNA – Did not attend; EYC – Early Years Census; EYFSP –Early years foundation stage profile; FNP – Family Nurse Partnership; HES – Hospital Episode Statistics; IP - Inpatients; KS1 – Key Stage 1; LIN – Linear regression; LO – Logistic; MNOM - multinomial NA - Not available; NPD – National Pupil Database; ONS – Office for National Statistics; OP – Outpatients; PLASC – Pupil Level and School Census; PO-Poisson; PRU – Pupil Referral Unit Census.; PVI – Private, Voluntary or Independent (PVI) establishment; SURV – Survival.

For peer review only

2. Sample size calculation

The sample size was fixed by the available trial cohort. For the primary outcome of CIN registration available UK data on rates are not specific to the age range of interest. However, the rate for the general population of children aged 5–9 years old in BB:0-2 study sites is 4.6%. We expect that the rate would be greater in the specific study sample, and therefore, we have assumed a rate of 8%. We hypothesised that FNP would reduce the rate of CIN registration in the first 6 years and that a difference of 4% would be important. To detect a difference of 4% (FNP of 4% vs UC of 8%) would require 602 children in each study arm (1204 in total) using 80% power and a two-sided 5% alpha level. The BB:0–2 trial recruited 1645 women, with 1562 available for follow-up (ie, excluding those subject to a mandatory withdrawal). Follow-up through administrative records (assuming 10% loss in tracking and linkage) would result in 1405 participants

3. Further details of Child in Need outcome.

In our primary comparative analysis we examined whether the firstborn ('BB:0-2 child') had ever been referred to social services and classed as a child in need at any point between birth and 6 years of age. There is no flag in the Child In Need dataset to identify whether a child is in need after they have been referred to social services. Therefore, CIN was defined (as per DfE methodology)[1] as all children referred to social services, excluding cases where:

- the referral is flagged as being a referral resulting in no further action; or
- the only activity recorded is an assessment and the reason for the closure of the case being that case was closed after assessment with no further action.

---

1. Department for Education. Characteristics of children in need in England: 2014 to 2015. 2015. [accessed 2019 Nov 12]. Available from: https://www.gov.uk/government/uploads/system/uploads/attachment_data/file/469740/SFR41-2015_Methodology.pdf

For peer review only - http://bmjopen.bmj.com/site/about/guidelines.xhtml

For peer review only

For peer review only - http://bmjopen.bmj.com/site/about/guidelines.xhtml

4. Representativeness of study sample

Scope and purpose: We assessed whether the study sample for BB:2-6 were similar to women recruited to the original BB:0-2 trial.

Conclusion: The BB:2-6 and BB:0-2 cohorts are similar in terms of maternal (Table S4.1) and baby characteristics (Table S4.2).

Table S4.1 Maternal characteristics of those in BB:0-2 compared to those in the BB:2-6

	**BB:0-2 N=1618**	**BB:2-6 N=1537**
**Minimisation variables at randomisation**		
**Gestational age at baseline** n(%)		
16 weeks or more	658 (40·7)	636 (41·4)
Less than 16 weeks	959 (59·3)	900 (58·6)
**Smoking status** n(%)		
No	860 (53·2)	822 (53·5)
Yes	758 (46·8)	715 (46·5)
**Language** n(%)		
English	1611 (99·6)	1530 (99·5)
Other	6 (0·4)	6 (0·4)
Missing	*1 (0·0)*	*0 (0·0)*
**Trial arm** n(%)		
FNP	808 (49·9)	766 (49·8)
Usual care	810 (50·1)	771 (50·2)
**Age at recruitment (years) Median (25th to 75th centile)**	17·9 (16·9 to 18·8)	17·9 (16·9 to 18·8)
**Ethnicity** n(%)		
White background	1425 (88·1)	1355 (88·2)
Mixed background	89 (5·5)	82 (5·3)
Asian background	27 (1·7)	25 (1·6)
Black background	71 (4·4)	69 (4·5)
Chinese or Other background	6 (0·4)	6 (0·4)
**Relationship status with baby's father** n(%)		
Married	20 (1·2)	16 (1·0)
Separated	165 (10·2)	150 (10·0)
Closely involved/boyfriend	1222 (75·5)	1168 (76·0)
Just friends	211 (13·0)	203 (13·2)
**Live with father of baby** n(%)		
Yes	368 (22·7)	348 (22·6)
No	1112 (68·7)	1061 (69·0)
Missing	138 (8·5)	128 (8·3)
**NEET status**[a] n(%)		
Yes	663 (41·0)	634 (41·2)
No	717 (44·3)	678 (44·1)
Missing	*4 (0·2)*	*4 (0·3)*
Not applicable (aged 16 years or younger)	*234 (14·5)*	*221 (14·4)*

For peer review only - http://bmjopen.bmj.com/site/about/guidelines.xhtml

	BB:0-2 N=1618	BB:2-6 N=1537
**Socio-economic status: Overall Index of Multiple Deprivation (IMD) Score**[b] Median (25th to 75th centile)	N=1606 38·2 (25·2 to 52·0)	N=1525 38·1 (25·0 to 52·1)
**Maternal health and wellbeing**		
**Generalized self-efficacy scale (score 10 to 40)**[c] Median (25th to 75th centile)	N=1592 30·0 (28·0 to 33·0)	N=1533 30·0 (28·0 to 33·0)
**Adaptive functioning: Difficulty in at least one basic skill** n(%)		
Yes	430 (26·6)	397 (25·8)
No	1185 (73·2)	1138 (74·0)
Missing	*3 (0·2)*	*2 (0·1)*
**Adaptive functioning: Had 3 or less life skills** n(%)		
Yes	434 (26·8)	403 (26·2)
No	1178 (72·8)	1129 (73·5)
Missing	*6 (0·4)*	*5 (0·3)*
**Adaptive functioning: At least one burden** n(%)		
Yes	476 (29·4)	451 (29·3)
No	1131 (69·9)	1075 (69·9)
Missing	*11 (0·7)*	*11 (0·7)*
**Smoking** (Participant self-reported) n(%)		
Ever smoked: Yes	1291 (79·8)	1227 (79·8)
No	327 (20·0)	310 (20·2)
Missing	*0 (0)*	*0 (0)*
**Child characteristics**	**BB:0-2 sample N=1510**	**BB:2-6 sample N=1547**
**Trial arm** n(%)		
FNP	742 (49·1)	773 (50·0)
Usual Care	768 (50·9)	774 (50·0)
**Gender** n(%)		
Male	777 (51·5)	787 (50·9)
Female	732 (48·5)	758 (40·1)
Missing	1	72[d]
**Birthweight (grams) Mean (sd)**	3207·3 (599·6)	3219·6 (580·7)
Missing[c]		71
**Neonatal unit (NNU) admission** n(%)		
Yes	149 (10·0)	142 (9·2)
Missing from data collection	13	11
Missing	Not applicable	71[d]

[a] *Definition of NEET status: Not in education employment or training (applicable only to those whose age at end of previous academic year at time of baseline interview was >16)*
[b] *Higher IMD score indicated more deprivation. Mean IMD Score for England in 2010 was 21·67* [60]*;*
[c] *Higher score indicates higher level of self-efficacy*
[d] *Mothers were withdrawn in BB:0-2 but are included in BB:2-6*

For peer review only

Table S4.2 Child characteristics of those in the BB:0-2 study compared to those in the BB:2-6 study

	**BB:0-2 sample N=1510**	**BB:2-6 sample N=1547**
**Trial arm** n(%)		
FNP	742 (49·1)	773 (50·0)
Usual Care	768 (50·9)	774 (50·0)
**Gender** n(%)		
Male	777 (51·5)	787 (50·9)
Female	732 (48·5)	758 (40·1)
Missing	1	72[a]
**Birthweight (grams) Mean (sd)**	3207·3 (599·6)	3219·6 (580·7)
Missing[a]		71
**Neonatal unit (NNU) admission** n(%)		
Yes	149 (10·0)	142 (9·2)
Missing from data collection	13	11
Missing[a]	Not applicable	71*

[a] *Mothers were withdrawn in BB:0-2 but are included in BB:2-6*

For peer review only - http://bmjopen.bmj.com/site/about/guidelines.xhtml

5. Number of valid FNP visits received

Of the 766 women in the FNP arm in the BB:2-6 cohort, 69 did not enrol for FNP. For the remaining 697 who were randomised and enrolled, 695 had received at least one valid visit and two did not receive any valid visits across the three phases (pregnancy, infancy and toddlerhood). The descriptives in Table S5.1 are based on 697 women. A greater proportion of women received no visits during the infancy and toddler phases (40 (5.7%) and 98 (14.1%) respectively) compared to the pregnancy phase (6 (0.7%)). This is similar to that reported in the BB:0-2 cohort with comparable median valid visits and proportions reaching the fidelity goals (see Lancet Supplementary appendix Table S11.3).[1]

Table S5.1 BB:2-6 study population data: Valid FNP visits per phase

	**Pregnancy**	**Infancy**	**Toddlerhood**
N women randomised and enrolled to FNP	697	697	697
Women not receiving any valid visits n(%)	6 (0·7)	40 (5·7)	98 (14·1)
Women receiving at least one valid visit n(%)	691 (99·3)	657 (94·3)	599 (85·9)
Valid visits received			
Median (25th to 75th centile)	10 (8 to 12)	19 (15 to 22)	13 (10 to 16)
Mean (SD)	9·74 (3·42)	18·7 (5·97)	13·28 (5·46)
Reaching fidelity goal of: n(%)			
80%+	401/697 (57·5)	-	-
65%+	-	364/697 (52·2)	-
60%+	-	-	297/697 (42·6)

[1] Robling M, Bekkers MJ, Bell K, Butler CC, Cannings-John R, Channon S, et al. Effectiveness of a nurse-led intensive home-visitation programme for first-time teenage mothers (Building Blocks): A pragmatic randomised controlled trial. Lancet. 2016;387:146–55. [accessed 2019 Nov 12]. Available from: http://www.thelancet.com/journals/lancet/article/PIIS0140-6736(15)00392-X/fulltext

For peer review only - http://bmjopen.bmj.com/site/about/guidelines.xhtml

6. Primary outcomes, secondary outcomes and additional Key Stage 1

Table S6.1 Objective measures of maltreatment: Primary and Secondary outcomes

**Outcome**	**FNP N=760**	**Usual care (UC) N=746**	**Adjusted* parameter estimate (95% CI)**	**p-value**	**Absolute risk difference (FNP-UC) (95% CI)**
**Primary outcome: Child in need (CIN)**					
**Child in need (CIN) status at any time during the follow-up period**					
No	599 (78·8%)	584 (78·3%)	Reference		
Yes	161 (21·2%)	162 (21·7%)	0·98† (0·74 to 1·31)	0·902	-0·5% (-0·5 to 0·4%)
**Secondary outcomes**					
**Age at first CIN referral (days)**					
Median (25th to 75th centile)	1602·5 (1153 to 1978·75)	1649·5 (1130·75 to 1980·25)	0·98‡ (0·79 to 1·23)	0·875	
**Age at first CIN referral (days)**					
Pre birth	<5	<5	*NT*		
Birth to <1 years	<5	<5			
1 to <2 years	6 (3·7%)	6 (3·7%)			
2 to <3 years	15 (9·4%)	28 (17·3%)			
3 to <4 years	27 (16·9%)	23 (14·2%)			
4 to <5 years	47 (29·4%)	39 (24·1%)			
5 to <6 years	33 (20·6%)	41 (25·3%)			
6 to <7 years	20 (12·5%)	20 (12·3%)			
Missing	*1*	*0*			

For peer review only - http://bmjopen.bmj.com/site/about/guidelines.xhtml

Outcome	FNP<br>N=760	Usual care (UC)<br>N=746	Adjusted* parameter estimate (95% CI)	p-value	Absolute risk difference (FNP-UC) (95% CI)
**Unique§ number of CIN referrals per child**	1 (1 to 2)	1 (1 to 2)	0·99¶ (0·82 to 1·19)	0·890	
1 case	110 (68·3%)	110 (67·9%)	*NT*		
2 cases	42 (26·1%)	42 (25·9%)			
3 or more cases	9 (5·5%)	10 (6·1%)			
**CIN cases closed at 31st March 2017**	116 (72·0%)	110 (67·9%)	*NT*		
**CIN duration (days)**	210 (70·5 to 571·25)	216 (72·75 to 503·25)	1·20‡ (0·90 to 1·59)	0·216	
**CIN categorisation (primary need) of first case**					
Abuse or neglect	92 (57·1%)	102 (63·0%)	Reference		
Family dysfunction	29 (18·0%)	34 (21·0%)	0·95‖ (0·54 to 1·69)	0·868	
Family in acute stress	13 (8·1%)	10 (6·2%)	1·47‖ (0·61 to 3·54)	0·389	
Low income, absent parenting, socially unacceptable behaviour, not stated	13 (8·1%)	7 (3·7%)	2·38‖ (0·86 to 6·57)	0·093	
Child/parent disability	7 (4·3%)	5 (3·1%)	1·47‖ (0·45 to 4·84)	0·525	
Cases other than CIN	7 (4·3%)	5 (3·1%)	1·64‖ (0·50 to 5·37)	0·417	
**Referred to Children's social care services (CSCS)**					
No	554 (72·9%)	538 (72·1%)	Reference		
Yes	206 (27·1%)	208 (27·9%)	0·97† (0·74 to 1·28)	0·829	-0·8% (-5·3 to 3·7%)
**Total referrals made** N	339	354			
**Ratio referrals:children**	1·65	1·70			
**Referrals made per child**					

For peer review only

Outcome	FNP N=760	Usual care (UC) N=746	Adjusted* parameter estimate (95% CI)	p-value	Absolute risk difference (FNP-UC) (95% CI)
1	159 (77·2%)	140 (67·3%)	*NT*		
2	34 (16·5%)	51 (24·5%)			
3 to 5	13 (6·3%)	27 (8·2%)			
**Age at first referral (days)** Median (25th to 75th centiles)	1541·5 (1272·75 to 1975·75)	1631·0 (1190·5 to 1984·75)	0·96‡ (0·79 to 1·17)	0·694	
**Unit=referral (referred to CSCS encompass any referral)**	**n=339**	**n=354**			
**Source of referral**					
Individual	29 (8·8%)	34 (10·0%)	*NT*		
School/Education	150 (45·7%)	136 (40·0%)			
Health services	114 (34·8%)	132 (38·8%)			
Local Authority Services	35 (10·7%)	38 (11·2%)			
Missing	*11*	*14*			
**Primary need of referrals**					
Abuse or neglect	198 (58·9%)	205 (57·9%)	*NT*		
Family dysfunction	72 (21·4%)	88 (24·9%)			
Family in acute stress	19 (5·7%)	21 (5·9%)			
Low income/absent parenting/ socially unacceptable behaviour	11 (3·3%)	7 (2·0%)			
Parental/Childs disability illness	14 (4·2%)	11 (3·1%)			
Cases other than Child in Need	11 (2·3%)	12 (3·4%)			
Not stated	11 (3·3%)	9 (2·5%)			

For peer review only - http://bmjopen.bmj.com/site/about/guidelines.xhtml

Outcome	FNP<br>N=760	Usual care (UC)<br>N=746	Adjusted* parameter estimate (95% CI)	p-value	Absolute risk difference (FNP-UC) (95% CI)
Missing	*3*	*1*			
**Child Protection Plan**					
No	708 (93·2%)	697 (93·4%)	Reference		
Yes	52 (6·8%)	49 (6·6%)	1·04† (0·69 to 1·57)	0·846	0·2% (-0·2 to 0·3%)
**Category of child protection plan**	**n=52**	**n=49**			
Neglect	17 (32·7%)	22 (44·9%)	Reference		
Emotional	22 (42·3%)	12 (24·5%)	2·77‖ (1·02 to 7·56)	0·046	
Physical	6 (11·5%)	6 (12·2%)	1·25‖ (0·32 to 4·88)	0·743	
Sexual and multiple**	7 (13·5%)	9 (18·4%)	1·13‖ (0·33 to 3·86)	0·848	
**Looked after status[a]**					
No	735 (96·7%)	719 (96·4%)	Reference		
Yes	25 (3·3%)	27 (3·6%)	0·90† (0·52 to 1·57)	0·712	-0·3% (-0·2 to 0·2%)
**Child looked after period of care (months)**	10·0 (4·5 to 37·5)	12·0 (6·0 to 33·0)	0·75¶ (0·65 to 0·86)	<0·001	
**Legal status of child looked after**					
Interim care order	6 (35·3%)	8 (34·8%)	*NT*		
Full care order	5 (29·4%)	6 (26·1%)			
Placement order granted	<5	<5			
Single period of accommodation under section 20	<5	<5			
**Death**	<10		*NT*		

Data are n (%), n/N (%), or median (25th to 75th centile) unless otherwise stated. <5 = numbers suppressed· FNP = Family Nurse Partnership Programme + Usual Care· NT = Not tested, descriptive only. a We reflect here the terminology used at the time of the trial but note the more contemporary ‘care experienced’ as a more familiar and accepted term.

For peer review only - http://bmjopen.bmj.com/site/about/guidelines.xhtml

* FNP compared to usual care. Analysis adjusted for stratification (site), minimisation variables (gestational age, smoking status at recruitment, and first or preferred language); † Odds ratio from logistic model; ‡ Hazard ratio from Cox model; § An unique referral is counted as a distinct referral date per child; ¶ Incidence rate ratio from Poisson model; ‖ Relative risk ratio from multinomial model; **Multiple indicates when more than one category of abuse is relevant to the child's current plan. Source: National Pupil Database, The Department for Education

Table S6.2 Child health, developmental and educational secondary outcomes

Outcome	FNP	Usual care	Adjusted* odds ratio (95% CI)	p-value	Absolute risk difference (FNP-Usual care) (95% CI)
**Special educational needs (SEN)† provision**	**N=759**	**N=747**			
No	540 (71·1%)	502 (67·2%)	Reference		
Yes	219 (28·9%)	245 (32·8%)	0·83 (0·67 to 1·03)	0·097	-3·9% (-8·6% to 0·7)
**Early educational attendance**	**N=759**	**N=747**			
**Children attending education‡ up to the age of 4 years**					
Yes	758 (99·7%)	747 (100·0%)	*NE*	-	-0·3% (-1·0 to 0·3%)
**Children attending school up to the age of:**					
4 years	753 (99·1%)	744 (99·6%)	*NE*	-	-0·5% (-1·5 to 0·4%)
3 years	479 (63·0%)	482 (64·5%)	*NT*		
2 years	137 (18·0%)	147 (19·7%)	*NT*		
**Attending an Ofsted registered private, voluntary and independent establishment up to the age of 4 years**	334 (43·9%)	308 (41·2%)	1·13 (0·91 to 1·40)	0·281	2·7% (-2·3 to 7·7%)
**Earliest type of day care**	**N=334**	**N=308**			

For peer review only - http://bmjopen.bmj.com/site/about/guidelines.xhtml

Outcome	FNP	Usual care	Adjusted* odds ratio (95% CI)	p-value	Absolute risk difference (FNP-Usual care) (95% CI)
Private	217 (65·0%)	221 (71·8%)	*NT*		
Voluntary	52 (15·6%)	57 (18·5%)			
Local Authority Day Nursery	36 (10·8%)	22 (7·1%)			
Other§	29 (8·7%)	8 (2·6%)			
**School attendance**	**N=754**	**N=740**			
**Number of academic sessions¶**	630 (264 to 638)	630 (262 to 638)			
**Overall absence**					
No absences	14 (1·9%)	14 (1·9%)	ref		
At least one absence	740 (98·1%)	726 (98·1%)	1·00 (0·47 to 2·12)	0·998	0·0% (-1·4 to 1·5%)
Rate of absence rate	5·08 (2·69 to 8·73)	5·19 (2·73 to 8·41)			
**Overall authorised absence**					
No absences	26 (3·4%)	26 (3·5%)	ref		
At least one absence	728 (96·6%)	714 (96·5%)	1·01 (0·58 to 1·75)	0·984	0·1% (-1·8 to 2.8%)
Rate of absence rate	3·50 (1·96 to 6·09)	3·79 (2·02 to 6·11)			
**Overall unauthorised absence**					
No absences	256 (34·0%)	245 (33·1%)	ref		
At least one absence	498 (66·0%)	495 (66·9%)	0·95 (0·76 to 1·18)	0·620	-0·8% (-5·6 to 3·9%)
Rate of absence rate	1·84 (0·76 to 4·34)	1·57 (0·63 to 3·88)			
**Early Years assessment**	**N=743**	**N=728**			
**Achieving good level of development (GLD)**					
Achieving GLD in all five areas of learning‖:	431 (58·0%)	380 (52·2%)	1·26 (1·03 to 1·55)	0·026	5·8% (0·7 to 10·9%)

For peer review only - http://bmjopen.bmj.com/site/about/guidelines.xhtml

**Outcome**	**FNP**	**Usual care**	**Adjusted* odds ratio (95% CI)**	**p-value**	**Absolute risk difference (FNP-Usual care) (95% CI)**
Communication and Language	*642 (86·4%)*	*613 (84·2%)*	*NT*		*2·2% (-1·4 to 5·8%)*
Physical Development	*676 (91·0%)*	*643 (88·3%)*	*NT*		*2·7% (-0·5 to 5·8%)*
Personal, Social and Emotional Development	*668 (89·9%)*	*634 (87·1%)*	*NT*		*2·8% (-0·4 to 6·1%)*
Literacy	*493 (66·4%)*	*458 (62·9%)*	*NT*		*3·4% (-1·4 to 8·3%)*
Maths	*545 (73·4%)*	*516 (70·9%)*	*NT*		*2·5% (-2·1 to 7·0%)*
Achieving GLD in all 17 early learning goals	412 (55·5%)	365 (50·1%)	1·24 (1·01 to 1·52)	0·043	5·3% (0·2 to 10·4%)
**Total point score**** Mean (SD)	32·22 (7·25)	31·59 (7·62)	0·65†† (-0·11 to 1·41)	0·094	
Score <34	325 (43·8%)	345 (47·4%)	*NT*		
Score = 34	187 (25·2%)	183 (25·1%)			
Score >34	231 (31·1%)	200 (27·5%)			
**Key Stage 1 assessments**	**N=740**	**N=732**			
**Reading**					
Lower than expected	257 (34·7%)	289 (39·5%)	Reference		
Reaching at least the expected standard‡‡	483 (65·3%)	443 (60·5%)	1·23 (0·99 to 1·53)	0·051	4·8 (-0·2 to 9·7)
Expected standard	*371 (50·1%)*	*337 (46·0%)*	*1·24 (0·99 to 1·56)*	*0·056*	*4·1 (-1·0 to 9·2)*
Higher standard	*112 (15·1%)*	*106 (14·5%)*	*1·20 (0·88 to 1·65)*	*0·250*	*0·7 (-3·0 to 4·3)*
**Maths**					
Lower than expected	281 (38·0%)	283 (38·7%)	Reference		
Reaching at least the expected standard‡‡	459 (62·0%)	449 (61·3%)	1·04 (0·84 to 1·28)	0·731	0·7 (-4·3 to 5·6)

For peer review only - http://bmjopen.bmj.com/site/about/guidelines.xhtml

Outcome	FNP	Usual care	Adjusted* odds ratio (95% CI)	p-value	Absolute risk difference (FNP-Usual care) (95% CI)
Expected standard	*392 (53·0%)*	*376 (51·4%)*	*1·06 (0·85 to 1·32)*	*0·611*	*1·6 (-3·5 to 6·7)*
Higher standard	*67 (9·1%)*	*73 (10·0%)*	*0·93 (0·64 to 1·35)*	*0·711*	*-0·9 (-3·9 to 2·1)*
**Science**					
Lower than expected	203 (27·4%)	219 (29·9%)	Reference		
Reaching at least the expected standard§§	537 (72·6%)	513 (70·1%)	1·14 (0·91 to 1·43)	0·254	2·5 (-2·1 to 7·1)
**Writing academic year**	**N=498**	**N=487**			
**2016/17**					
Lower than expected	257 (51·6%)	278 (57·1%)	Reference		
Reaching at least the expected standard‡‡	241 (48·4%)	209 (42·9%)	1·24 (0·97 to 1·60)	0·090	5·5 (-0·7 to 11·6)
Expected standard	*218 (43·8%)*	*182 (37·4%)*	*1·29 (1·00 to 1·68)*	*0·054*	*6·4 (0·3 to 12·5)*
Higher standard	*23 (4·6%)*	*27 (5·5%)*	*0·92 (0·51 to 1·64)*	*0·769*	*-0·9 (-3·8 to 1·9)*
**2017/18**	**N=242**	**N=245**			
Lower than expected	82 (33·9%)	81 (33·1%)			
Reaching at least the expected standard‡‡	160 (66·1%)	164 (66·9%)	*NT*		-0·8 (-9·2 to 7·5)
Expected standard	*140 (57·9%)*	*132 (53·9%)*	*NT*		*4·0 (-4·8 to 12·7)*
Higher standard	*20 (8·3%)*	*32 (13·1%)*	*NT*		*-4·8 (-10·4 to 0·7)*

Data are n (%), n/N (%), or median (25th to 75th centile) unless otherwise stated. <5 = numbers suppressed. FNP = Family Nurse Partnership Programme + Usual Care. SD=standard deviation. NT = Not tested, descriptive only. NE = Not estimable. *FNP compared to usual care. Analysis adjusted for stratification (site), minimisation variables (gestational age, smoking status at recruitment, and first or preferred language); † A child with SEN provision is recorded using the following codes (A = School Action, P = School Action Plus, E = Education, Health and Care Plan, S=Statement, K= SEN support) and the N code was used to indicate No SEN support in any of the following datasets between 2013 and 2017. PLASC (Autumn, Spring and Summer terms), AP, PRU Census, and EYC. If for any of the years no response was recorded then the assumption was that the child was not included in the denominator as either not present on Census day or not in school. ‡ School or an Ofsted registered Private, Voluntary or Independent (PVI) establishment; § Childminding Network, Childminder, Registered Independent School, Other; ¶

For peer review only - http://bmjopen.bmj.com/site/about/guidelines.xhtml

Total number of sessions over the academic years available (2013 to Spring 2017) and all 6 terms per academic year. ‖ Children achieving a good level of development are those achieving at least the expected level within the prime and specific areas of learning; **Total point score ranges from 17 to 51 with a higher score indicating a better level of development; †† Mean difference from linear model. ‡‡ Working at the expected standard and at a greater depth within the expected standard; §§Working at a greater depth within the expected standard is not applicable in science. Source: National Pupil Database, The Department for Education

For peer review only

Table S6.3 Proportion of children reaching the expected standard in KS1 assessments by trial arm and academic year

		Academic year 2016/17	Academic year 2017/18
Reading	FNP	60.6%	74.8%
	Usual care	54.0%	73.5%
Maths	FNP	56.2%	74.0%
	Usual care	54.0%	75.9%
Science	FNP	68.5%	81.0%
	Usual care	63.7%	82.9%

Source: National Pupil Database, The Department for Education

Figure S6.4 Distribution of children's month of birth by trial arm

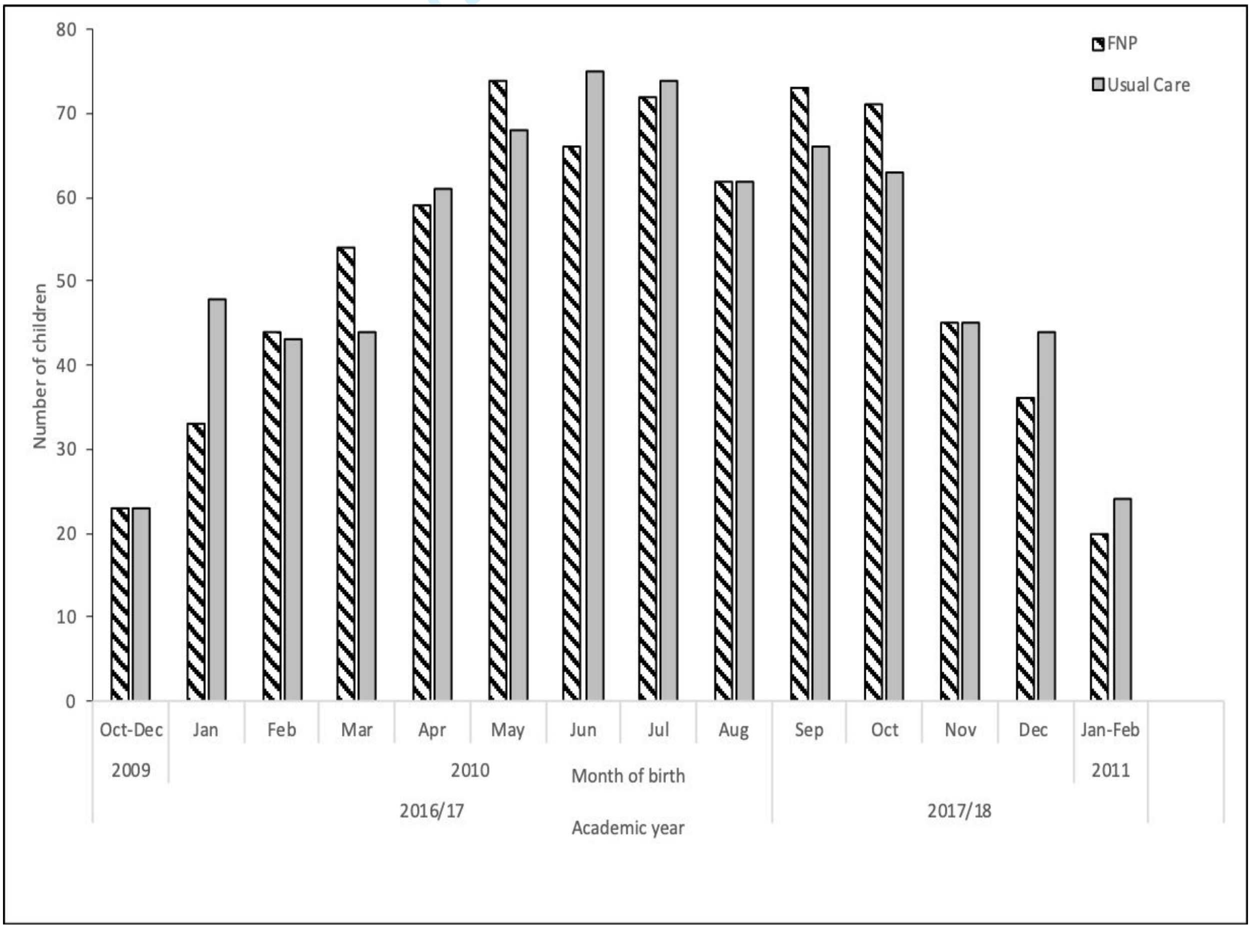

For peer review only - http://bmjopen.bmj.com/site/about/guidelines.xhtml

Table S6.5 Comparison of parameter estimates before and after adjustment for child's month[a] of birth

	Odds ratio[b] (95% CI), p-values	
	**Adjusted for site and minimisation variables[c]**	**Adjusted for site, minimisation variables[c] and month of birth**
**Key Stage 1 outcome**		
**Reading**		
Reaching at least the expected standard	1.23 (0.99 to 1.53), 0.051	1.26 (1.02 to 1.57), 0.035
Expected standard	*1.24 (0.99 to 1.56), 0.056*	*1.27 (1.01 to 1.59), 0.042*
Higher standard	*1.20 (0.88 to 1.65), 0.250*	*1.26 (0.91 to 1.75), 0.161*
**Maths**		
Reaching at least the expected standard	1.04 (0.84 to 1.28), 0.731	1.06 (0.85 to 1.31), 0.613
Expected standard	*1.06 (0.85 to 1.32), 0.611*	*1.07 (0.86 to 1.34), 0.527*
Higher standard	*0.93 (0.64 to 1.35), 0.711*	*0.93 (0.74 to 1.17), 0.522*
**Science**		
Reaching at least the expected standard	1.14 (0.91 to 1.43), 0.254	1.16 (0.92 to 1.47), 0.197
**Writing academic year 2017/18**		
Reaching at least the expected standard	1.24 (0.97 to 1.60), 0.090	1.26 (0.98 to 1.62), 0.076
Expected standard	*1.29 (1.00 to 1.68), 0.054*	*1.30 (1.00 to 1.69), 0.050*
Higher standard	*0.92 (0.51 to 1.64), 0.769*	*0.97 (0.54 to 1.75), 0.917*
**Early Years Assessment**		
Achieving GLD in all five areas of learning[d]	1.26 (1.03 to 1.55), 0.026	1.31 (1.05 to 1.62), 0.015
Achieving GLD in all 17 early learning goals	1.24 (1.01 to 1.52), 0.043	1.27 (1.03 to 1.58), 0.027
Total point score	0.65 (-0.11 to 1.41), 0.094	0.70 (-0.03 to 1.42), 0.060

a Quarter of birth runs from September to November, December to February, March to May, June to August; b Odds ratio represents the FNP arm compared to Usual care; c gestational age, smoking status at recruitment, and first or preferred language; d Children achieving a good level of development (GLD) are those achieving at least the expected level within the prime and specific areas of learning.

Source: National Pupil Database, The Department for Education

For peer review only

7. Results of planned sub-group analysis

No differential effects due to maternal age, deprivation, participation in employment, education or training, or basic life skills were found in planned sub-group analyses for any of the objective maltreatment outcomes (Table S7.1 and 7.2). The beneficial impact of FNP on EYFSP total point score was mostly observed for children with mothers aged <16 years rather than those aged ≥16 at study entry but no other differences by study sub-group were found (Table S7.3).

The differential effects due to maternal age, deprivation, participation in employment, education or training, or basic life skills for KS1 reading, maths and science are displayed in Tables S7.4a to e and for writing in Table S7.5. At KS1, sex differences were large with girls outperforming boys in all four subject areas (Table S7.4a). The intervention effect was greater for boys than girls with a higher proportion reaching the expected standard in the FNP arm, particularly for reading and writing (and strengthened after adjusting for month of birth) and for the writing assessment (academic year 2016/17) this interaction reached statistical significance after adjusting for month of birth (post-hoc analysis). When examining how the intervention effects differed in mothers aged less than 16 years at recruitment compared to mothers aged 16 years or more in each KS1 assessment, differences were observed in maths (Table S7.4b) and writing (Table S7.5), with a larger effect seen in mothers aged less than 16 at recruitment, when compared to those aged 16 or more.

Table S7.1 Subgroup analyses for referrals to Children's social care services

N(%) referred	FNP	Usual care	Adjusted[a] OR (95% CI), p-value	Interaction p-value
**Child Gender**				
Boys	99 (26.3)	108 (27.7)	0.93 (0.65 to 1.33), 0.69	0.78
Girls	107 (27.9)	100 (28.1)	1.01 (0.69 to 1.48), 0.94	
**Maternal age at recruitment**				
<16 years	17 (34.7)	19 (34.6)	1.05 (0.44 to 2.46), 0.92	0.96
≥16 years	189 (26.6)	189 (27.4)	0.96 (0.72 to 1.30), 0.81	
**Adaptive functioning**				
No difficulty	140 (25.4)	149 (26.3)	0.99 (0.71 to 1.38), 0.96	0.67
Difficulty in basic skills	66 (31.7)	59 (33.0)	0.81 (0.59 to 1.41), 0.67	
**NEET status**				
EET	70 (20.3)	75 (23.1)	0.83 (0.54 to 1.29), 0.42	0.18
NEET	100 (32.5)	89 (29.2)	1.19 (0.80 to 1.78), 0.39	
**Deprivation quintile**				

For peer review only - http://bmjopen.bmj.com/site/about/guidelines.xhtml

1 – least deprivation	33 (20.4)	34 (23.9)	0.77 (0.44 to 1.36), 0.37	0.32
2	36 (23.8)	30 (20.5)	1.26 (0.61 to 2.60), 0.53	
3	42 (28.8)	43 (27.9)	0.96 (0.50 to 1.86), 0.91	
4	41 (31.1)	39 (25.3)	1.27 (0.69 to 2.31), 0.44	
5 – most deprivation	54 (33.3)	61 (42.4)	0.65 (0.35 to 1.22), 0.18	

[a] FNP compared to usual care. Analysis adjusted for stratification (site), minimisation variables (gestational age, smoking status at recruitment, and first or preferred language). N/EET – Not/In Education, Employment or Training (Source: National Pupil Database, The Department for Education)

Table S7.2 Subgroup analyses for child in need status

N(%) CIN	FNP	Usual care	Adjusted[a] OR (95% CI), p-value	Interaction p-value
**Gender**				
Boys	80 (21.3)	80 (20.5)	1.05 (0.72 to 1.53), 0.81	0.56
Girls	81 (21.1)	82 (23.0)	0.91 (0.60 to 1.38), 0.65	
**Maternal age at recruitment**				
<16 years	14 (28.6)	16 (29.1)	1.00 (0.41 to 2.43), 0.99	0.98
≥16 years	147 (20.7)	146 (21.1)	0.98 (0.72 to 1.33), 0.88	
**Adaptive functioning**				
No difficulty	106 (19.2)	113 (20.0)	0.99 (0.70 to 1.42), 0.98	0.76
Difficulty in basic skills	55 (26.4)	49 (27.4)	0.93 (0.59 to 1.47), 0.76	
**NEET**				
EET	52 (16.0)	52 (15.1)	0.93 (0.57 to 1.53), 0.78	0.42
NEET	81 (26.3)	72 (23.6)	1.18 (0.77 to 1.77), 0.46	
**Deprivation quintile**				
1 – least deprivation	24 (14.8)	22 (15.5)	0.92 (0.49 to 1.73), 0.79	0.62
2	30 (19.9)	22 (15.1)	1.53 (0.68 to 3.47), 0.31	
3	32 (21.9)	36 (23,4)	0.89 (0.52 to 1.54), 0.68	
4	32 (24.2)	34 (22.1)	1.15 (0.64 to 2.06), 0.64	
5 – most deprivation	43 (26.5)	48 (33.3)	0.72 (0.38 to 1.38), 0.32	

[a] FNP compared to usual care. Analysis adjusted for stratification (site), minimisation variables (gestational age, smoking status at recruitment, and first or preferred language). N/EET – Not/In Education, Employment or Training (Source: National Pupil Database, The Department for Education).

For peer review only - http://bmjopen.bmj.com/site/about/guidelines.xhtml

Table S7.3 Subgroup analyses for Early Years Assessment total point score

Mean (SD) total point score	FNP	Usual care	Adjusted[a] mean difference (95% CI), p-value	Interaction p-value
**Gender**				
Boys	N=369<br>30.57 (7.33)	N=383<br>29.84 (7.56)	0.75 (-0.31 to 1.82), 0.17	0.57
Girls	N=374<br>33.85 (6.79)	N=345<br>33.53 (7.22)	0.34 (-0.67 to 1.35), 0.51	
**Maternal age at recruitment**				
<16 years	N=45<br>32.44 (6.71)	N=52<br>28.91 (7.70)	3.65 (0.76 to 6.55), 0.013	0.046
≥16 years	N= 698<br>32.21 (7.28)	N= 676<br>31.80 (7.58)	0.42 (-0.36 to 1.21), 0.29	
**Adaptive functioning**				
No difficulty	N=540<br>32.99 (7.12)	N=552<br>32.11 (7.65)	0.88 (0.003 to 1.75), 0.049	0.50
Difficulty in basic skills	N=203<br>30.18 (7.20)	N=175<br>29.94 (7.33)	0.34 (-1.11 to 1.79), 0.65	
**NEET status**				
In EET	N=339<br>32.45 (7.12)	N=322<br>32.37 (7.68)	0.15 (-0.99 to 1.28), 0.80	0.95
NEET	N= 304<br>31.72 (7.57)	N=299<br>31.50 (7.43)	0.19 (-1.00 to 1.38), 0.76	
**Index of Multiple Deprivation quintiles**				
1 = least deprived	N=159<br>33.16 (7.44)	N=139<br>31.53 (8.24)	1.72 (-0.07 to 3.50), 0.060	ref
2	N=148<br>32.16 (6.97)	N=144<br>31.59 (7.05)	0.65 (-0.96 to 2.27), 0.43	0.84
3	N=146<br>32.16 (6.79)	N=151<br>32.59 (7.27)	-0.20 (-1.79 to 1.39), 0.80	0.21
4	N=128<br>30.95 (7.53)	N=148<br>31.11 (7.50)	-0.31 (-2.11 to 1.48), 0.73	0.85
5 = most deprived	N=156<br>32.41 (7.46)	N=140<br>31.03 (8.12)	1.45 (-0.33 to 3.22), 0.11	0.74

[a] FNP compared to usual care. Analysis adjusted for stratification (site), minimisation variables (gestational age, smoking status at recruitment, and first or preferred language). N/EET – Not/In Education, Employment or Training (Source: National Pupil Database, The Department for Education).

Table S7.4 Subgroup analyses for KS1 assessments reaching the expected standard (RES)

(a) Gender

N(%)	Boys		Girls	
	Not RES	RES	Not RES	RES
**Reading**				
FNP	152 (41.8)	212 (58.2)	105 (27.9)	271 (72.1)
Usual care	182 (47.3)	203 (52.7)	107 (30.8)	240 (69.2)
Adjusted[a] OR (95% CI), p-value	1.27 (0.95 to 1.69), 0.11		1.16 (0.84 to 1.59), 0.38	
Interaction p-value=0.68				

For peer review only - http://bmjopen.bmj.com/site/about/guidelines.xhtml

Adjusted[b] OR (95% CI), p-value	1.30 (0.96 to 1.75), 0.09		1.17 (0.84 to 1.62), 0.36	
Interaction p-value=0.68				
**Maths**				
FNP	149 (40.9)	215 (59.1)	132 (35.1)	244 (64.9)
Usual care	166 (43.1)	219 (56.9)	117 (33.7)	230 (66.3)
Adjusted[a] OR (95% CI), p-value	1.11 (0.83 to 1.49), 0.49		0.95 (0.69 to 1.29), 0.72*	
Interaction p-value=0.46				
Adjusted[b] OR (95% CI), p-value	1.14 (0.84 to 1.54), 0.40		0.94 (0.69 to 1.29), 0.71	
Interaction p-value=0.47				
**Science**				
FNP	120 (33.0)	244 (67.0)	83 (22.1)	293 (77.9)
Usual care	138 (35.8)	247 (64.2)	81 (23.3)	266 (76.7)
Adjusted[a] OR (95% CI), p-value	1.16 (0.86 to 1.58), 0.34		1.08 (0.76 to 1.53), 0.68	
Interaction p-value=0.77				
Adjusted[b] OR (95% CI), p-value	1.18 (0.87 to 1.63), 0.28		1.08 (0.76 to 1.55), 0.66	
Interaction p-value=0.72				

RES = Reaching the expected standard; [a] Analysis adjusted for stratification (site), minimisation variables (gestational age, smoking status at recruitment, and first or preferred language); [b] Analysis adjusted for stratification (site), minimisation variables (gestational age, smoking status at recruitment, and first or preferred language) and month of birth. (Source: National Pupil Database, The Department for Education).

(b) Maternal age at recruitment

N(%)	Maternal age<16 years		Maternal age≥16 years	
	**Not RES**	**RES**	**Not RES**	**RES**
**Reading**				
FNP	21 (44.7)	26 (55.3)	236 (34.1)	457 (65.9)
Usual care	29 (52.7)	26 (47.3)	260 (38.4)	417 (61.6)
Adjusted[a] OR (95% CI), p-value	1.21 (0.97 to 1.52), 0.09		1.49 (0.65 to 3.41), 0.34	
Interaction p-value=p=0.66				
Adjusted[b] OR (95% CI), p-value	1.50 (0.65 to 3.48), 0.34		1.25 (0.99 to 1.57), 0.07	
Interaction p-value=0.62				
**Maths**				
FNP	16 (34.0)	31 (66.0)	265 (38.2)	428 (61.8)
Usual care	32 (58.2)	23 (41.8)	251 (37.1)	426 (62.9)
Adjusted[a] OR (95% CI), p-value	3.15 (0.94 to 10.48), 0.06		0.96 (0.77 to 1.19), 0.69	
Interaction p-value=0.011				

For peer review only - http://bmjopen.bmj.com/site/about/guidelines.xhtml

Adjusted[b] OR (95% CI), p-value	3.23 (1.36 to 7.67), 0.008		0.98 (0.78 to 1.22), 0.83	
Interaction p-value=0.014				
**Science**				
FNP	18 (38.3)	29 (61.7)	185 (26.7)	508 (73.3)
Usual care	25 (45.4)	30 (54.6)	194 (28.7)	483 (71.3)
Adjusted[a] OR (95% CI), p-value	1.54 (0.53 to 4.47), 0.42		1.11 (0.88 to 1.41), 0.39	
Interaction p-value=0.56				
Adjusted[b] OR (95% CI), p-value	1.37 (0.59 to 3.19), 0.467		1.14 (0.89 to 1.45), 0.29	
Interaction p-value=0.69				

RES = Reaching the expected standard; [a] FNP compared to Usual care. Analysis adjusted for stratification (site), minimisation variables (gestational age, smoking status at recruitment, and first or preferred language); [b] Analysis adjusted for stratification (site), minimisation variables (gestational age, smoking status at recruitment, and first or preferred language) and month of birth. (Source: National Pupil Database, The Department for Education).

(c) Adaptive functioning

N(%)	No difficulty with basic skills		Difficulty with basic skills	
	Not RES	RES	Not RES	RES
**Reading**				
FNP	152 (30.5)	347 (69.5)	87 (42.9)	116 (57.1)
Usual care	188 (36.2)	332 (63.8)	84 (48.0)	91 (52.0)
Adjusted[a] OR (95% CI), p-value	1.29 (0.98 to 1.70), 0.07		1.23 (0.82 to 1.86), 0.32	
Interaction p-value=0.61				
Adjusted[b] OR (95% CI), p-value	1.29 (0.98 to 1.69), 0.07		1.25 (0.83 to 1.91), 0.29	
Interaction p-value=0.95				
**Maths**				
FNP	186 (34.6)	351 (65.4)	95 (46.8)	108 (53.2)
Usual care	192 (34.5)	364 (65.5)	91 (52.0)	84 (48.0)
Adjusted[a] OR (95% CI), p-value	1.01 (0.78 to 1.29), 0.97		1.27 (0.84 to 1.93), 0.26	
Interaction p-value=0.38				
Adjusted[b] OR (95% CI), p-value	1.01 (0.78 to 1.31), 0.93		1.32 (0.86 to 2.03), 0.20	
Interaction p-value=0.28				
**Science**				
FNP	130 (24.2)	407 (75.8)	73 (36.0)	130 (64.0)
Usual care	153 (27.5)	403 (72.5)	66 (37.7)	109 (62.3)
Adjusted[a] OR (95% CI), p-value	1.20 (0.92 to 1.58), 0.19		1.10 (0.71 to 1.69), 0.68	
Interaction p-value=0.71				

For peer review only - http://bmjopen.bmj.com/site/about/guidelines.xhtml

Adjusted[b] OR (95% CI), p-value	1.22 (0.93 to 1.62), 0.16	1.13 (0.73 to 1.74), 0.59
Interaction p-value=0.82		

RES = Reaching the expected standard; [a] FNP compared to Usual care. Analysis adjusted for stratification (site), minimisation variables (gestational age, smoking status at recruitment, and first or preferred language); [b] Analysis adjusted for stratification (site), minimisation variables (gestational age, smoking status at recruitment, and first or preferred language) and month of birth. (Source: National Pupil Database, The Department for Education).

(d) Not in Education, employment or training (NEET) status

N(%)	NEET		In EET	
	Not RES	RES	Not RES	RES
**Reading**				
FNP	105 (31.2)	232 (68.8)	109 (36.0)	194 (64.0)
Usual care	115 (35.8)	206 (61.2)	121 (40.5)	178 (59.5)
Adjusted[a] OR (95% CI), p-value	1.26 (0.91 to 1.75), 0.17		1.21 (0.87 to 1.69), 0.26	
Interaction p-value=0.86				
Adjusted[b] OR (95% CI), p-value	1.31 (0.94 to 1.83), 0.17		1.25 (0.89 to 1.76), 0.19	
Interaction p-value=0.83				
**Maths**				
FNP	122 (36.2)	215 (63.8)	122 (40.3)	181 (59.7)
Usual care	107 (33.3)	214 (66.7)	120 (40.1)	179 (59.9)
Adjusted[a] OR (95% CI), p-value	0.89 (0.65 to 1.23), 0.48		0.99 (0.72 to 1.38), 0.97	
Interaction p-value=0.64				
Adjusted[b] OR (95% CI), p-value	0.93 (0.66 to 1.29), 0.65		1.03 (0.73 to 1.42), 0.90	
Interaction p-value=0.68				
**Science**				
FNP	87 (25.8)	250 (74.2)	821 (27.1)	221 (72.9)
Usual care	78 (24.3)	243 (75.7)	98 (32.8)	201 (67.2)
Adjusted[a] OR (95% CI), p-value	0.93 (0.65 to 1.33), 0.69		1.31 (0.92 to 1.87), 0.14	
Interaction p-value=0.20				
Adjusted[b] OR (95% CI), p-value	0.96 (0.67 to 1.39), 0.84		1.36 (0.95 to 1.95), 0.10	
Interaction p-value=0.22				

RES = Reaching the expected standard; [a] FNP compared to Usual care. Analysis adjusted for stratification (site), minimisation variables (gestational age, smoking status at recruitment, and first or preferred language); [b] Analysis adjusted for stratification (site), minimisation variables (gestational age, smoking status at recruitment, and first or preferred language) and month of birth. N/EET – Not/In Education, Employment or Training (Source: National Pupil Database, The Department for Education).

For peer review only - http://bmjopen.bmj.com/site/about/guidelines.xhtml

(e) Index of Multiple Deprivation quintiles

	N(%)	Q1=least deprived		Q2		Q3		Q4		Q5=most deprived	
		**Not RES**	**RES**	**Not RES**	**RES**	**Not RES**	**RES**	**Not RES**	**RES**	**Not RES**	**RES**
**Reading**	FNP	49 (30.3)	113 (67.7)	53 (36.3)	93 (63.7)	49 (33.6)	97 (66.4)	48 (37.2)	81 (62.8)	57 (37.8)	94 (62.2)
	Usual care	54 (38.9)	85 (61.1)	64 (44.1)	81 (55.9)	48 (31.8)	103 (68.2)	64 (43.0)	85 (57.0)	56 (39.4)	86 (60.6)
	Adjusted[a] OR (95% CI), p-value	1.57 (0.88 to 2.77), 0.12		1.42 (0.88 to 2.29), 0.16		0.96 (0.59 to 1.57), 0.88		1.33 (0.81 to 2.18), 0.25		1.07 (0.60 to 1.89), 0.83	
	Adjusted[b] OR (95% CI), p-value	1.75 (1.00 to 3.07), 0.05		1.42 (0.85 to 2.37), 0.18		0.89 (0.53 to 1.48), 0.64		1.41 (0.86 to 2.34), 0.18		1.00 (0.57 to 1.75), 0.99	
	Interaction p-value=0.70										
	Interaction p-value=0.37										
Maths	FNP	56 (34.6)	106 (65.4)	57 (39.0)	89 (61.0)	50 (34.3)	96 (65.7)	55 (42.6)	74 (57.4)	62 (41.1)	89 (58.9)
	Usual care	52 (37.4)	87 (62.6)	60 (41.4)	85 (58.6)	56 (37.1)	95 (62.9)	60 (40.3)	89 (59.7)	54 (38.0)	88 (62.0)
	Adjusted[a] OR (95% CI), p-value	1.15 (0.72 to 1.85), 0.56		1.12 (0.69 to 1.82), 0.65		1.17 (0.69 to 1.97), 0.56		0.93 (0.57 to 1.52), 0.77		0.91 (0.56 to 1.47), 0.69	
	Adjusted[b] OR (95% CI), p-value	1.36 (0.82 to 2.24), 0.23		1.12 (0.68 to 1.82), 0.66		1.11 (0.65 to 1.90), 0.69		0.98 (0.60 to 1.62), 0.95		0.88 (0.54 to 1.44), 0.62	
	Interaction p-value=0.91										
	Interaction p-value=0.81										

For peer review only - http://bmjopen.bmj.com/site/about/guidelines.xhtml

	N(%)	Q1=least deprived		Q2		Q3		Q4		Q5=most deprived	
		**Not RES**	**RES**	**Not RES**	**RES**	**Not RES**	**RES**	**Not RES**	**RES**	**Not RES**	**RES**
**Science**	FNP	37 (22.8)	125 (77.2)	36 (24.7)	110 (75.3)	40 (27.4)	106 (72.6)	45 (34.9)	84 (65.1)	44 (29.1)	107 (70.9)
	Usual care	45 (32.4)	94 (67.6)	45 (31.0)	100 (69.0)	35 (23.2)	116 (76.8)	47 (31.5)	102 (68.5)	46 (32.4)	96 (67.6)
	Adjusted[a] OR (95% CI), p-value	1.68 (1.00 to 2.81), 0.048		1.39 (0.80 to 2.41), 0.25		0.82 (0.46 to 1.47), 0.51		0.89 (0.52 to 1.51), 0.65		1.24 (0.74 to 2.06), 0.42	
	Adjusted[b] OR (95% CI), p-value	1.94 (1.13 to 3.30), 0.015		1.40 (0.76 to 2.57), 0.28		0.78 (0.43 to 1.40), 0.41		0.92 (0.55 to 1.55), 0.76		1.16 (0.69 to 1.96), 0.57	
	Interaction p-value=0.25										
	Interaction p-value=0.11										

RES = Reaching the expected standard; a FNP compared to Usual care; [a] Analysis adjusted for stratification (site), minimisation variables (gestational age, smoking status at recruitment, and first or preferred language); [b] Analysis adjusted for stratification (site), minimisation variables (gestational age, smoking status at recruitment, and first or preferred language) and month of birth. (Source: National Pupil Database, The Department for Education).

For peer review only

Table S7.5 Subgroup analyses for reaching the expected standard in the writing KS1 assessment for 2016/17 academic year

N(%)	FNP	Usual care	Adjusted[a] OR (95% CI), p-value	Interaction p-value	Adjusted[b] OR (95% CI), p-value	Interaction p-value
**Gender**						
Boys	109 (42.9)	83 (31.8)	1.61 (1.13 to 2.32), 0.009	0.034	1.62 (1.13 to 2.33), 0.009	0.039
Girls	132 (54.1)	126 (55.8)	1.03 (0.76 to 1.39), 0.87		0.94 (0.65 to 1.37), 0.76	
**Maternal age at recruitment**						
<16 years	18 (54.6)	9 (22.5)	4.67 (1.47 to 14.84), 0.009	0.016	5.28 (1.49 to 18.73), 0.010	0.018
≥16 years	223 (48.0)	200 (44.7)	1.13 (0.87 to 1.47), 0.35		1.15 (0.89 to 1.50), 0.29	
**Adaptive functioning**						
No difficulty	189 (52.5)	174 (46.7)	1.26 (0.93 to 1.70), 0.14	0.72	1.26 (0.93 to 1.70), 0.14	0.67
Difficulty in basic skills	52 (37.7)	34 (30.1)	1.41 (0.77 to 2.58), 0.26		1.53 (0.81 to 2.90), 0.19	
**NEET**						
EET (no not EET)	109 (47.8)	119 (52.2)	0.82 (0.56 to 1.19), 0.29	0.019	0.82 (0.56 to 1.19), 0.29	0.028
NEET	105 (49.1)	77 (38.3)	1.56 (1.05 to 2.30), 0.027		1.56 (1.05 to 2.30), 0.027	
**Deprivation quintile**						
1 – least deprived	63 (53.9)	39 (42.9)	1.65 (0.90 to 3.05), 0.11	0.89	1.83 (0.95 to 3.51), 0.07	0.86
2	48 (46.2)	46 (44.7)	1.00 (0.52 to 1.95), 0.99		1.04 (0.53 to 2.03), 0.92	
3	46 (50.6)	49 (46.7)	1.20 (0.67 to 2.15), 0.53		1.22 (0.66 to 2.26), 0.53	
4	40 (45.5)	35 (38.5)	1.28 (0.70 to 2.35), 0.42		1.28 (0.69 to 2.35), 0.43	
5 – most deprived	42 (44.2)	39 (41.1)	1.16 (0.64 to 2.08), 0.629		1.17 (0.64 to 2.14), 0.602	

For peer review only - http://bmjopen.bmj.com/site/about/guidelines.xhtml

[a] FNP compared to usual care. Analysis adjusted for stratification (site), minimisation variables (gestational age, smoking status at recruitment, and first or preferred language); [b] Analysis adjusted for stratification (site), minimisation variables (gestational age, smoking status at recruitment, and first or preferred language) and month of birth. N/EET – Not/In Education, Employment or Training (Source: National Pupil Database, The Department for Education).

For peer review only

For peer review only - http://bmjopen.bmj.com/site/about/guidelines.xhtml

Domestic abuse and CIN status:

Domestic abuse experienced by the mother was assessed using the Composite Abuse Scale (CAS) in the BB:0-2 trial at 24 months follow-up (with a score 1 or greater indicating some sort of abuse). The CAS could only be administered if the interview was face-to-face (as opposed to by telephone) and only if the participant was alone (N=607). In the BB:2-6 sample, this could be examined in 595 participants (FNP:322, Usual Care:273) and the proportion of participants experiencing some form of abuse was comparable to that found in the BB:0-2 trial (FNP: 122 (27.9%) vs UC:113 (41.4%)). There was also a relationship found between CAS and CIN status with 49 (20.9%) participants who experienced some abuse having a child being in need versus 42 (11.8%) of those who did not experience abuse. Domestic abuse at 24 months was to be explored as a possible effect mediator but due to lack of intervention effect in the child's CIN status, the need to examine the proportion of effect mediated by experience of domestic abuse at 24 months was redundant.

Care-experienced mothers and CIN status:

In addition, care-experienced mothers at time of recruitment to the BB:0-2 trial was examined. This was defined from the CLA dataset. Sixty-one mothers were identified as having experienced a period of care, four of whom were identified as care-experienced only after their BB:0-2 recruitment date. Fifty-seven mothers were examined for their duration of care and the women randomised to receive FNP experienced on average nine months more care than the Usual care arm (median (25th to 75th centile) FNP: 40 (15.5 to 113.0) UC: 31 (7.0 to 69.5) months). After merging these care-experienced mothers into the children's dataset, 56 mothers remained; seven mothers were excluded from the subgroup analyses since their child did not have any NPD data. Children of these care-experienced mothers were at a higher risk of being defined as in need themselves but no interaction was observed between trial arm and mothers care status. The duration of the maternal care status as a possible mediator effect was also stated in the statistical analysis plan but similarly to domestic abuse, due to lack of intervention effect in child's CIN status, then the need to examine the proportion of effect mediated by length of mother's care experience was redundant.

8. Treatment efficacy

The analyses of the following outcomes were re-run to investigate the effect of FNP dosage (number of visits over all delivery phases) using complier averaged causal effects modelling by fitting a structural mean model. The programme stated maximum number of visits over the whole period of FNP is 64 (14 in the pregnancy phase, 28 in the infancy phase, 22 in the toddler phase).

For peer review only - http://bmjopen.bmj.com/site/about/guidelines.xhtml

<u>Referrals to CSC services:</u> Adjusting the analysis for receipt of full dosage of FNP visits, made no difference to the conclusion of the main analysis for referral to social services (efficacy per visit odds ratio (OR)=1.00, 95% CI=0.99 to 1.004, p=0.613).

<u>CIN status:</u> Adjusting the analysis for receipt of FNP made no difference to the conclusion of the primary outcome (efficacy per visit OR=1.00, 95% CI=0.99 to 1.004, p=0.613).

<u>Early Years assessment total point score:</u> Adjusting the analysis for receipt of FNP we observe a 0.02 increase in point score in the between-arm mean difference in early years total score (as the number of visits increase so does the score) however the confidence interval around this estimate is wide (-0.004 to 0.040). The estimated treatment efficacy for participants receiving all 64 visits was 1.15 (95% CI -0.26 to 2.56), p=0.106 showing no change to the overall conclusions compared to the primary analysis.

<u>KS1 attainment:</u> Adjusting the analysis for FNP receipt we observe an increase in the odds of an event occurring in the FNP arm compared UC arm for every unit increase in FNP visit received. For all areas of learning (reading, writing, maths and science), adjusting the analysis for FNP receipt strengthens the effect of FNP, but it is the reading and writing assessment where given the full dosage of 64 FNP visits the odds make a significant difference. For reading assessments, the odds of reaching the expected standard increases from OR=1.23 (95% CI=0.99 to 1.53), p=0.051 in the main analysis to 1.38 (1.07 to 1.77), p=0.015. The efficacy per visit for reading was 1.005 (1.001 to 1.009), p=0.015; maths: 1.001 (0.997 to 1.005), p=0.535; science: 1.004 (0.999 to 1.008), p=0.112; writing: 1.013 (1.09 to 1.017), p=0.001.

9. Missing data

Since only a small number (n=40 (2.7%)) of children were not linked to any NPD dataset and not included in the primary outcome analyses, no multiple imputation was carried out.

For peer review only - http://bmjopen.bmj.com/site/about/guidelines.xhtml

10. Cost Consequence Analysis

Table S10.1 Cost-consequence balance sheet for the family-nurse based intervention for the four years between children's ages 2 and 6.

**Mothers**						
	Mean resource use per participant			Mean cost per participant (£)		
	FNP	Usual Care	Incremental (FNP-Usual Care)	FNP	Usual Care	Incremental (FNP-Usual Care)
**Health related resource use**						
Inpatient Attendances						
Overnight admissions (N)	3.04	2.99	0.05			
Overnight length of stay (N nights)	2.32	2.32	0.00	2,826.79	2,844.58	-17.79
Day Admittances	2.93	2.91	0.02	1,874.61	1,890.11	-15.50
Outpatient Attendances						
All Outpatient Attendances	23.29	22.61	0.68	2,244.62	2,189.32	55.30
Accident and Emergency						
A&E Visits (N)	5.19	4.93	0.26	411.34	392.03	19.31
Total Resource Use						
				7,357.36	7,316.04	41.32 p=0.393
**Children**						
	Mean resource use per participant			Mean cost per participant (£)		
	FNP	Usual Care	Incremental (FNP-Usual Care)	FNP	Usual Care	Incremental (FNP-Usual Care)
**Health related resource use**						
Inpatient Attendances - All						
Overnight admissions (N)	1.57	1.55	0.02			
Overnight length of stay (N nights)	3.57	3.39	0.18	1,962.95	2,033.71	-70.76
Day Admittances	0.98	0.97	0.01	744.94	724.90	20.04
Inpatient Attendances – All (Excluding Injuries and Ingestions)						
Overnight admissions (N)	1.51	1.49	0.02	1,883.44	1,951.15	-67.71
Overnight length of stay (N nights)	3.61	3.44	0.17			
Day Admittances	0.90	0.88	0.02	689.22	667.09	22.13
Inpatient Attendances – Injuries and Ingestions Only						
Overnight admissions (N)	0.06	0.06	0.00	79.51	82.55	-3.04

For peer review only - http://bmjopen.bmj.com/site/about/guidelines.xhtml

Overnight length of stay (N nights)	2.50	2.05	0.45			
Day Admittances	0.08	0.09	-0.01	55.72	57.81	-2.09
Outpatient Attendances						
All Outpatient Attendances	6.89	7.44	-0.55	673.24	727.79	-54.55
Accident and Emergency - All						
A&E Visits (N)	4.93	4.62	0.31	395.73	366.83	28.90
Accident and Emergency - All (Excluding Injuries and Ingestions)						
A&E Visits (N)	3.80	3.52	0.28	305.12	280.20	24.92
Accident and Emergency – Injuries and Ingestions Only						
A&E Visits (N)	1.12	1.10	0.02	75.09	69.91	5.18
Total Resource Use						
				3,778.13	3,853.22	-75.09 p=0.865
**Consequences**						
Child in Need status recorded at any time during the follow-up period.						OR: 0.98 (95%CI: 0.74 to 1.31) p=0.902
Referral to Social Services						OR: 0.97 (0.74 to 1.28) p=0.829
Child Protection registration (CPP)						OR: 1.04 (0.69 to 1.57) p=0.846
Details of a CPP						
• Neglect						Reference
• Emotional						OR: 2.77 (1.02 to 7.56) p=0.046
• Physical						OR: 1.25 (0.32 to 4.88) p=0.743
• Sexual and multiple						OR: 1.13 (0.33 to 3.86) p=0.848
CIN categorisation						
• Abuse or Neglect						Reference
• Family dysfunction						OR: 0.95 (0.54 to 1.69) p=0.868
• Family in acute stress						OR: 1.47 (0.61 to 3.54) p=0.389
• Low income, absent parenting, socially unacceptable behaviour						OR: 2.38 (0.86 to 6.57) p=0.093
• Child/parent disability						OR: 1.47 (0.45 to 4.84) p=0.525
Looked after status						OR: 0.90 (0.52 to 1.57) p=0.712
Child looked after period of care (months)						OR: 0.75 (0.65 to 0.86) p<0.001
DNA appointments						
• DNA at least once						OR: 1.00 (0.79 to 1.26) p=0.997
Injuries and ingestions						
• Emergency attendance						
○ No						Reference
○ Yes						OR: 1.17 (0.95 to 1.45) p=0.149
• Admission to hospital						

For peer review only - http://bmjopen.bmj.com/site/about/guidelines.xhtml

For peer review only

○ No	Reference
○ Yes	OR: 0.87 (0.63 to 1.20) p=0.407
• Either hospital attendance and/or admission to hospital	
○ No	Reference
○ Yes	OR: 1.11 (0.89 to 1.37) p=0.351
Special Educational Needs	OR: 0.83 (0.67 to 1.03) p=0.097
Early Years assessment	
• Achieving good level of development in all five areas of learning	OR: 1.26 (1.03 to 1.55) p=0.026
• Achieving good level of development in all 17 early learning goals	OR: 1.24 (1.01 to 1.52) p=0.043
School attendance	
• Overall absences	
○ No absences	Reference
○ At least one absence	OR: 1.00 (0.47 to 2.12) p=0.998
• Overall unauthorised absences	
○ No absences	Reference
○ At least one absence	OR: 0.95 (0.76 to 1.18) p=0.620
• Overall authorised absences	
○ No absences	Reference
○ At least one absence	OR: 1.01 (0.58 to 1.75) p=0.984
KS1 attainment	
• Reading	
○ Lower than expected	Reference
○ Reaching the expected standard	OR: 1.23 (0.99 to 1.53) p=0.051
• Writing academic year 2016/17	
○ Lower than expected	Reference
○ Reaching the expected standard	OR: 1.24 (0.97 to 1.60) p=0.090
• Maths	
○ Lower than expected	Reference
○ Reaching the expected standard	OR: 1.04 (0.84 to 1.28) p=0.731
• Science	
○ Lower than expected	Reference
○ Reaching the expected standard	OR: 1.14 (0.91 to 1.43) p=0.254

For peer review only

For peer review only - http://bmjopen.bmj.com/site/about/guidelines.xhtml

The RECORD statement – checklist of items, extended from the STROBE statement, that should be reported in observational studies using routinely collected health data.

	Item No.	STROBE items	Location in manuscript where items are reported	RECORD items	Location in manuscript where items are reported
**Title and abstract**					
	1	(a) Indicate the study's design with a commonly used term in the title or the abstract (b) Provide in the abstract an informative and balanced summary of what was done and what was found	1a/b Abstract	RECORD 1.1: The type of data used should be specified in the title or abstract. When possible, the name of the databases used should be included.<br><br>RECORD 1.2: If applicable, the geographic region and timeframe within which the study took place should be reported in the title or abstract.<br><br>RECORD 1.3: If linkage between databases was conducted for the study, this should be clearly stated in the title or abstract.	1.1 Abstract<br>1.2 Abstract<br>1.3 Abstract
**Introduction**					
Background rationale	2	Explain the scientific background and rationale for the investigation being reported	Introduction		
Objectives	3	State specific objectives, including any prespecified hypotheses	Introduction > Objectives		
**Methods**					
Study Design	4	Present key elements of study design early in the paper	Methods> Study design, setting and participants		
Setting	5	Describe the setting, locations, and relevant dates, including periods of recruitment, exposure, follow-up, and data collection	Methods>Procedure and data sources		

For peer review only

Participants	6	*(a) Cohort study* - Give the eligibility criteria, and the sources and methods of selection of participants. Describe methods of follow-up<br>*Case-control study* - Give the eligibility criteria, and the sources and methods of case ascertainment and control selection. Give the rationale for the choice of cases and controls<br>*Cross-sectional study* - Give the eligibility criteria, and the sources and methods of selection of participants<br><br>*(b) Cohort study* - For matched studies, give matching criteria and number of exposed and unexposed<br>*Case-control study* - For matched studies, give matching criteria and the number of controls per case	6a Methods> Study design, setting and participants<br><br>6b NA	RECORD 6.1: The methods of study population selection (such as codes or algorithms used to identify subjects) should be listed in detail. If this is not possible, an explanation should be provided.<br><br>RECORD 6.2: Any validation studies of the codes or algorithms used to select the population should be referenced. If validation was conducted for this study and not published elsewhere, detailed methods and results should be provided.<br><br>RECORD 6.3: If the study involved linkage of databases, consider use of a flow diagram or other graphical display to demonstrate the data linkage process, including the number of individuals with linked data at each stage.	6.1 Methods> Study design, setting and participants<br><br>6.2 NA<br><br>6.3 Protocol paper cited, Figure 1
Variables	7	Clearly define all outcomes, exposures, predictors, potential confounders, and effect modifiers. Give diagnostic criteria, if applicable.	Methods> Study design, setting and participants, Outcomes; Appendix	RECORD 7.1: A complete list of codes and algorithms used to classify exposures, outcomes, confounders, and effect modifiers should be provided. If these cannot be reported, an explanation should be provided.	Methods> Study design, setting and participants, Outcomes
Data sources/ measurement	8	For each variable of interest, give sources of data and details of methods of assessment (measurement).<br>Describe comparability of assessment methods if there is more than one group	Methods> Study design, setting and participants, Outcomes; Appendix		

For peer review only

Bias	9	Describe any efforts to address potential sources of bias	Methods> Efforts to address sources of bias		
Study size	10	Explain how the study size was arrived at	Methods> Power calculation		
Quantitative variables	11	Explain how quantitative variables were handled in the analyses. If applicable, describe which groupings were chosen, and why	Methods>Statistical analysis		
Statistical methods	12	(a) Describe all statistical methods, including those used to control for confounding<br>(b) Describe any methods used to examine subgroups and interactions<br>(c) Explain how missing data were addressed<br>(d) *Cohort study* - If applicable, explain how loss to follow-up was addressed<br>*Case-control study* - If applicable, explain how matching of cases and controls was addressed<br>*Cross-sectional study* - If applicable, describe analytical methods taking account of sampling strategy<br>(e) Describe any sensitivity analyses	12a-c, e: Methods>Statistical analysis; Economic analysis<br>12d: NA		
Data access and cleaning methods		..		RECORD 12.1: Authors should describe the extent to which the investigators had access to the database population used to create the study population.	Methods> Study design, setting and participants, Outcomes; Appendix

For peer review only

				RECORD 12.2: Authors should provide information on the data cleaning methods used in the study.	
Linkage		..		RECORD 12.3: State whether the study included person-level, institutional-level, or other data linkage across two or more databases. The methods of linkage and methods of linkage quality evaluation should be provided.	Methods> Procedure and data sources
**Results**					
Participants	13	(a) Report the numbers of individuals at each stage of the study (*e.g.*, numbers potentially eligible, examined for eligibility, confirmed eligible, included in the study, completing follow-up, and analysed)<br>(b) Give reasons for non-participation at each stage.<br>(c) Consider use of a flow diagram	Table 1, Figure 1	RECORD 13.1: Describe in detail the selection of the persons included in the study (*i.e.,* study population selection) including filtering based on data quality, data availability and linkage. The selection of included persons can be described in the text and/or by means of the study flow diagram.	Figure 1
Descriptive data	14	(a) Give characteristics of study participants (*e.g.*, demographic, clinical, social) and information on exposures and potential confounders<br>(b) Indicate the number of participants with missing data for each variable of interest<br>(c) *Cohort study* - summarise follow-up time (*e.g.*, average and total amount)	14a: Table 2<br>14b: Table 1, Table 3, Table 4, Appendix tables (S6.1-2)		
Outcome data	15	*Cohort study* - Report numbers of outcome events or summary measures over time<br>*Case-control study* - Report numbers in each exposure	Table 3, table 4, Appendix tables (S6.1-2)		

For peer review only

		category, or summary measures of exposure *Cross-sectional study* - Report numbers of outcome events or summary measures			
Main results	16	(a) Give unadjusted estimates and, if applicable, confounder-adjusted estimates and their precision (e.g., 95% confidence interval). Make clear which confounders were adjusted for and why they were included (b) Report category boundaries when continuous variables were categorized (c) If relevant, consider translating estimates of relative risk into absolute risk for a meaningful time period	Table 3, table 4, Appendix tables (S6.1-2)		
Other analyses	17	Report other analyses done—e.g., analyses of subgroups and interactions, and sensitivity analyses	Appendix		
**Discussion**					
Key results	18	Summarise key results with reference to study objectives	Discussion> Principal findings		
Limitations	19	Discuss limitations of the study, taking into account sources of potential bias or imprecision. Discuss both direction and magnitude of any potential bias		RECORD 19.1: Discuss the implications of using data that were not created or collected to answer the specific research question(s). Include discussion of misclassification bias, unmeasured confounding, missing data, and changing eligibility over time, as they pertain to the study being reported.	Discussion> Strengths and weaknesses of this study, Strengths and weaknesses in comparison to other studies
Interpretation	20	Give a cautious overall interpretation of results considering objectives,			Discussion> Strengths and weaknesses of this study, Strengths

For peer review only

		limitations, multiplicity of analyses, results from similar studies, and other relevant evidence			and weaknesses in comparison to other studies
Generalisability	21	Discuss the generalisability (external validity) of the study results			Discussion> Strengths and weaknesses in comparison to other studies
**Other Information**					
Funding	22	Give the source of funding and the role of the funders for the present study and, if applicable, for the original study on which the present article is based			Described in Funding section
Accessibility of protocol, raw data, and programming code		..		RECORD 22.1: Authors should provide information on how to access any supplemental information such as the study protocol, raw data, or programming code.	Protocol paper cited in references; access to data described in Data sharing statement

*Reference: Benchimol EI, Smeeth L, Guttmann A, Harron K, Moher D, Petersen I, Sørensen HT, von Elm E, Langan SM, the RECORD Working Committee. The REporting of studies Conducted using Observational Routinely-collected health Data (RECORD) Statement. *PLoS Medicine* 2015; in press.

*Checklist is protected under Creative Commons Attribution (CC BY) license.

For peer review only

BMJ Open

A nurse-led home-visitation programme for first-time mothers in reducing maltreatment and improving child health and development (BB:2-6): longer-term outcomes from a randomised cohort using data-linkage.

Journal:	*BMJ Open*
Manuscript ID	bmjopen-2021-049960.R1
Article Type:	Original research
Date Submitted by the Author:	18-Jun-2021
Complete List of Authors:	Robling, Michael; Cardiff University, Centre for Trials Research; DECIPHer, Cardiff University School of Social Sciences<br>Lugg-Widger, Fiona; Cardiff University, Centre for Trials Research<br>Cannings-John, Rebecca; Cardiff University Centre for Trials Research,<br>Angel, Lianna; Cardiff University, Centre for Trials Research<br>Channon, Sue; Cardiff University, Centre for Trials Research<br>Fitzsimmons, Deborah; Swansea University, College of Human and Health Sciences<br>Hood, Kerenza; Cardiff University,<br>Kenkre, Joyce; University of South Wales, Faculty of Life Sciences and Education<br>Moody, Gwenllian; Cardiff University, Centre for Trials Research<br>Owen-Jones, Eleri; Cardiff University, Centre for Trials Research<br>Pockett, Rhys; Swansea University, Swansea Centre for Health Economics<br>Sanders, Julia; Cardiff University, School of Healthcare Sciences<br>Segrott, Jeremy; Cardiff University, Centre for Trials Research; Cardiff University, DECIPHer Centre, Cardiff School of Social Sciences<br>Slater, Thomas; School of Social Sciences
**Primary Subject Heading**:	Public health
Secondary Subject Heading:	Nursing
Keywords:	PUBLIC HEALTH, Child protection < PAEDIATRICS, PREVENTIVE MEDICINE

SCHOLARONE™
Manuscripts

I, the Submitting Author has the right to grant and does grant on behalf of all authors of the Work (as defined in the below author licence), an exclusive licence and/or a non-exclusive licence for contributions from authors who are: i) UK Crown employees; ii) where BMJ has agreed a CC-BY licence shall apply, and/or iii) in accordance with the terms applicable for US Federal Government officers or employees acting as part of their official duties; on a worldwide, perpetual, irrevocable, royalty-free basis to BMJ Publishing Group Ltd ("BMJ") its licensees and where the relevant Journal is co-owned by BMJ to the co-owners of the Journal, to publish the Work in this journal and any other BMJ products and to exploit all rights, as set out in our licence.

The Submitting Author accepts and understands that any supply made under these terms is made by BMJ to the Submitting Author unless you are acting as an employee on behalf of your employer or a postgraduate student of an affiliated institution which is paying any applicable article publishing charge ("APC") for Open Access articles. Where the Submitting Author wishes to make the Work available on an Open Access basis (and intends to pay the relevant APC), the terms of reuse of such Open Access shall be governed by a Creative Commons licence – details of these licences and which Creative Commons licence will apply to this Work are set out in our licence referred to above.

Other than as permitted in any relevant BMJ Author's Self Archiving Policies, I confirm this Work has not been accepted for publication elsewhere, is not being considered for publication elsewhere and does not duplicate material already published. I confirm all authors consent to publication of this Work and authorise the granting of this licence.

For peer review only

Title: A nurse-led home-visitation programme for first-time mothers in reducing maltreatment and improving child health and development (BB:2-6): longer-term outcomes from a randomised cohort using data-linkage

Authorship:

Michael Robling, [1,2] (Director of Population Health Trials), RoblingMR@cardiff.ac.uk

Fiona Lugg-Widger, [1] (Research Fellow), LuggFV@cardiff.ac.uk

Rebecca Cannings-John, [1] (Senior Research Fellow), CanningsRL@cardiff.ac.uk

Lianna Angel, [2] (Research Associate), AngelL@cardiff.ac.uk

Susan Channon, [1] (Director Research Design and Conduct Service) ChannonS2@cardiff.ac.uk

Deborah Fitzsimmons, [3] (Personal Chair, Public Health, Policy and Social Sciences) d.fitzsimmons@swansea.ac.uk

Kerenza Hood, [1] (Director of the Centre for Trials Research) HoodK1@cardiff.ac.uk

Joyce Kenkre, [4] (Professor of Primary Care) joyce.kenkre@southwales.ac.uk

Gwenllian Moody, [1] (Research Associate) MoodyG@cardiff.ac.uk

Eleri Owen-Jones, [1] (Research Associate) Owen-JonesCE@cardiff.ac.uk

Rhys Pockett, [3] (Senior Lecturer) r.d.pockett@swansea.ac.uk

Julia Sanders, [5] (Professor of Clinical Nursing and Midwifery) SandersJ3@cardiff.ac.uk

Jeremy Segrott, [1,2] (Senior lecturer) SegrottJ@cardiff.ac.uk

Thomas Slater, [6] (Senior lecturer) slaterTB1@cardiff.ac.uk

Affiliations:

1. Centre for Trials Research, Cardiff University, Cardiff, UK
2. DECIPHer, Cardiff University, UK
3. Swansea Centre for Health Economics, Swansea University, UK
4. Faculty of Life Sciences and Education, University of South Wales, UK
5. School of Healthcare Sciences, Cardiff University, UK
6. School of Social Sciences, Cardiff University, UK

Corresponding author:

Michael Robling, Director of Population Health Trials, RoblingMR@cardiff.ac.uk, Centre for Trials Research, College of Biomedical & Life Sciences, Cardiff University, 7th Floor, Neuadd Meirionnydd, Heath Park, Cardiff. CF14 4YS

Keywords: Home-visiting, maltreatment, child development, routine data, cohort

For peer review only

Abstract (300 words)

Objectives Measure effectiveness of Family Nurse Partnership (FNP) home-visiting programme in reducing maltreatment and improving maternal health and child health, developmental and educational outcomes; explore effect moderators, mediators; describe costs.

Design Follow-up of BB:0-2 trial cohort (ISRCTN:23019866) up to age 7 years in England using record linkage.

Participants 1,618 mothers aged 19 years or younger and their firstborn child(ren) recruited to BB:0-2 trial at less than 25 weeks gestation and not mandatorily withdrawn from trial or opted out. Intervention families were offered up to a maximum of 64 home visits by specially trained nurses from pregnancy until firstborn child was 2 years old, plus usually provided health and social care support. Comparator was usual care alone.

Outcome measures Primary outcome: state-verified child-in-need status recorded at any time during follow-up. Secondary outcomes: referral to social services, child protection registration (plan), child-in-need categorisation, looked-after status, recorded injuries and ingestions any time during follow-up, early childcare and educational attendance, school readiness and attainment at Key Stage 1 (KS1), healthcare costs.

Results Match rates for 1,547 eligible children (1517 singletons, 15 sets of twins) were 98.3% (NHS Digital) and 97.4% (National Pupil Database). There was no difference between study arms in the proportion of children being registered as in need (adjusted OR=0.98, 95% CI=0.74,1.31), or for any other measure of maltreatment. Children in the FNP arm were more likely to achieve a good level of development at reception age (school readiness) (adjusted OR=1.24, 95% CI= 1.01,1.52). After adjusting for birth month, children in FNP arm were more likely to reach the expected standard in reading at KS1 (adjusted OR=1.26, 95% CI=1.02,1.57). We found no trial arm differences for resource use and costs.

Conclusions FNP did not improve maltreatment or maternal outcomes. There was evidence of small advantages in school readiness and attainment at KS1.

Strengths and Limitations

- We used administrative records and novel data linkage to provide longer-term follow-up for a well characterised trial cohort
- This has maximised cohort retention and minimised bias
- We used data from a range of sectors including health, social care and education to assess the broad impact of the specialist home visiting programme

For peer review only

Manuscript (Word count is 5026)

Introduction

Protecting children from maltreatment and promoting their healthy development are UK policy priorities. The Family Nurse Partnership (FNP) is a preventative home-visiting programme for young women expecting their first child. FNP was developed in the USA as the Nurse Family Partnership (NFP) and subsequently adapted for implementation in England in 2007.

Three US trials have demonstrated programme improvements in prenatal health behaviours and birth outcomes, sensitive childcare, maternal life-course and child functioning.[1–3] A subgroup analysis of poor unmarried teenage mothers in the first US trial (n=54) found verified maltreatment by age 2 years in 19% of 32 control children and 4% of the 22 children in the group in receipt of NFP during both pregnancy and infancy (mean % difference: 0.15, 95% confidence interval (CI) of −0.01 to 0.31).[1] Additionally, there was a 56% relative reduction in emergency department encounters for injuries and ingestions during the second year of life. For children with state-verified maltreatment reported by age 4, children in the NFP group had fewer risks for harm than the control group between 25 and 50 months of life. A trial of VoorZorg, the Dutch adaptation of NFP, found a reduction in child-protecting agency reports by age three years for families in the home-visiting arm.[4,5]

We evaluated short-term FNP programme outcomes to age 2 years in our Building Blocks trial (BB:0-2).[6] We found no difference for four primary outcomes: maternal tobacco use in late pregnancy, birth weight of the baby, proportion of women with a second pregnancy within 24 months postpartum and emergency attendances and hospital admissions for the child within 24 months postpartum. There were some differences in favour of FNP for secondary child development outcomes including maternal-reported cognitive function and language at 24 months.[6] As the previous US trials showed benefit for maltreatment outcomes increasingly after age four we sought to establish whether FNP moderates maltreatment outcomes over a medium-term period. Through access to administrative data, we aimed to determine impact across a range of child maltreatment outcomes and key indicators of neglect (e.g. injuries and ingestions). We also sought to assess impacts on other programme relevant developmental and maternal outcomes as well as costs.

Objectives

Primary: to determine programme effectiveness in reducing objectively measured maltreatment, when compared with usually provided health and social care alone. Secondary: to determine programme effectiveness in reducing maltreatment, medium-term programme outcomes such as

For peer review only - http://bmjopen.bmj.com/site/about/guidelines.xhtml

subsequent pregnancies, school readiness and educational outcomes, the impact of moderators of programme effect and the cost and consequences of the programme.

Methods

Study design, setting and participants

This study followed a cohort of mothers and children who had participated in the BB:0-2 trial for a further five years using administrative data only.[7] Data were retrieved from public sector providers and linked to the trial dataset using opt-out consent, following REC approval (14/WA10062) and s251 support (CAG 10-08(b)/2014). Children and mothers were followed up until the child reached Key Stage 1 teacher-based assessment (KS1) by which time most children were seven years old.

Building Blocks: 2-6 (BB:2-6) study participants were women and their first child (or twins, if relevant) who were not mandatorily withdrawn from the BB:0-2 trial; did not electively withdraw and/or did not opt-out of this follow-on study.[6,8] BB:0-2 trial participants were eligible if women were nulliparous, aged 19 years or younger, were recruited at less than 25 weeks' gestation, living within the catchment area of a local FNP team, able to provide consent and speak English. Women expecting multiple births and those with a previous pregnancy ending in miscarriage, stillbirth, or termination were eligible. Women planning to have their child adopted or to move beyond the FNP catchment area for more than 3 months were not eligible. [6,7] Trial participants had been randomly assigned to FNP or usual care, with randomisation stratified by site and minimised by gestation (<16 weeks vs ≥16 weeks), smoking (yes vs no), and preferred language of data collection (English vs non- English) and weighted towards minimising the imbalance in trial groups with probability 0.8.

Intervention and comparator (BB:0-2 Trial)

The FNP programme provides up to a maximum of 64 home visits from specially trained family nurses during pregnancy until the child is two years old addressing personal and environmental health, life course development, maternal role, family and friends and access to health and social services. For the 697 women randomised and enrolled to FNP in BB:0-2 and then retained in the BB:2-6 cohort, the average number (SD) of valid visits received by FNP clients by programme phase was: Pregnancy 9.74 (3.42), Infancy 18.7 (5.97) and Toddlerhood 13.28 (5.46) (Table S1). In the BB:0-2 trial, mean (SD) visit duration varied by delivery phase (Pregnancy: 79.14 mins (13.78), Infancy: 73.17 (11.61), Toddlerhood: 74.75 (13.50) which exceeded the programme target of 60 minutes.[8] All trial participants received usually provided health and social care services. Participants in the usual care (UC) arm received these services alone.[6,9,10] The number (SD) of midwifery contacts were recorded in the BB:0-2 trial and were for FNP clients 10.68 (5.25) and for women in UC arm 10.69 (5.34).[10] The number (SD) of home visits from specialist public health nurses (SPHN) reported by trial participants by 18 months postpartum was for FNP clients 4.70 (7.81) and for women in the UC

arm 5.01 (5.5). The average number (SD) of clinic contacts with SPHNs was 6.31 (7.07) for women in the control arm and 0.70 (2.29) in the FNP arm.

Procedure and data sources

The BB:0-2 trial consented 1618 eligible women between June 2009 and July 2010 and we concluded all BB:2-6 follow-up in May 2018. Maternal and child identifiers were sent to NHS Digital and the Department for Education (DfE) for matching with their databases. Maternal identifiers were also sent to the Department of Health and Social Care (DHSC) for matching with abortion statistics. Matching with NHS Digital and DHSC used a combination of NHS number, date of birth, postcode and sex. NHS Digital also provided a linked Hospital Episode Statistics (HES) - Office for National Statistics (ONS) extract for mortality data. Matching with the DfE used exact matching on first name and surname, date of birth and postcode (of both mother and child for social care data; all other datasets, just child). All matched data were sent to a third-party data safe haven (Secure Anonymised Information Linkage (SAIL) Databank) and linked by project identifiers to trial data and analysed via remote access.[11-13]

Outcomes

The primary outcome was Child in Need (CIN) status recorded at any time by a local department of children's social care services (CSCS) (and sourced from the NPD). This includes children assessed as unlikely to achieve or maintain a reasonable level of health or development, or whose health and development is likely to be significantly or further impaired, without the provision of services; or is a child who is disabled. Secondary outcomes were additional objective measures of maltreatment (referral to CSCS, child being on a Child Protection Plan, CIN categorisation, Looked after status (mother, child)); Associated measures of maltreatment (recorded injuries and ingestions, non-attendance rates for hospital appointments); Maternal outcomes (subsequent pregnancies); Child health, developmental and educational outcomes (special educational needs, early educational attendance and assessments (Early Years Foundation Stage Profile (EYFSP), KS1)); and health resource use. Study assessment domains, outcomes and principal data providers and analysis are described in the appendix (Table S2).

For peer review only

Power calculation

Our sample size was fixed by the cohort available from BB:0-2. However, for CIN status, while UK data on rates are not specific to the age-range of interest, the rate per 10,000 general population aged 5-9 years is 4.6% for local authorities covering BB:0-2 trial sites.[14] The rate of CIN status would be expected to be greater in the specific study sample. Therefore, we assumed a rate of 8%, hypothesised that FNP would reduce the presence of CIN in the first six years and assumed a difference of 4% would be important. To detect a difference of 4% (4% vs 8%) would require 602 children in each arm with 80% power and a two-sided 5% alpha level. With 1562 children available for follow-up (i.e. excluding mandatory withdrawals) and follow up through administrative records with 10% loss in tracking and linkage we would have 1405 participants, sufficient data to assess the primary outcome.

Efforts to address sources of bias

All maltreatment and child developmental outcomes were independently state-verified (social worker) reports or teacher-assessments respectively, recorded in administrative service data and abstracted from national data providers. By using administrative data and established linking fields we were able to minimise bias due to loss to follow-up. An a priori statistical analysis plan described primary, secondary (including sub-group) and sensitivity analyses, and any additional exploratory analysis is clearly highlighted.

Statistical analysis

Analysis was conducted on participants whose identifiers could be sent, linked and data released by the data providers. This excluded participants whose individual data could be used but could not be matched due to incorrect linking fields, other exclusions from health or education: e.g. private or home schooling and National opt-outs. Imbalance between the BB:0-2 and eligible BB:2-6 participants was quantified using descriptive statistics in baseline demographics, clinical and questionnaire data. We conducted all analyses on an intention-to-treat (ITT) basis with complete case population (with follow-up data). Therefore, participants remained in the BB:0-2 trial groups they were randomised to regardless of the intervention received. We used multilevel modelling to allow for clustering of effect within the 18 sites and family nurse level and both fitted as random effects. Where clustering was minimal at the family nurse level, results from the two-level model are presented. Parameter estimates are reported alongside a 95% CI and p-value. We adjusted for variables used in randomisation (smoking status, gestational age, language). We defined loss to follow up in this study as a child death or adoption, both of which we were able to determine. We excluded children from analyses of binary outcomes where follow-up was incomplete and no event was observed. With time-to-event analyses, children were censored at these events.

For peer review only - http://bmjopen.bmj.com/site/about/guidelines.xhtml

Primary comparative analysis examined whether the firstborn child(ren) ('BB:0-2') had ever been referred to CSCS and classed as a child in need at any point between birth and 6 years of age (appendix). For this binary outcome we used logistic multilevel modelling to investigate differences in the proportion defined as in need between the trial arms. We present the resulting estimate as an odds ratio. For binary and categorical outcomes, comparative analysis used logistic and multinomial modelling respectively with results presented as odds ratios (OR). For continuous outcomes we used linear regression and present mean differences. For count data we used Poisson regression modelling; where event distribution displayed signs of over dispersion a Negative Binomial regression model was used (or zero-inflated model if appropriate). Results are presented as incidence rate ratios (IRR). Time to event data used Cox regression modelling presented with hazard ratios (HR). To reduce problems with multiple comparisons, some outcomes were only assessed descriptively (for example, we prioritised and tested overall scores for a Good Level of Development in the Early Years Profile, rather than the individual areas of learning). Similarly, for outcomes that we expected to reflect a small number of events or higher levels of missingness we also took a descriptive approach. Nevertheless, we present all a priori comparative analyses in the main tables and all other descriptive and exploratory analyses in the supplementary materials.

KS1 data were received in two tranches from NPD, firstly children assessed in 2016/17 academic year (received in May 2018) and then younger children who were assessed in 2017/18 (received May 2019). Reading, maths and science were examined for the whole cohort over both academic years. However, following changes made within the 2017/18 writing assessment (such that judgements in 2018 are not directly comparable to those made using the previous interim frameworks in 2016/2017), writing was only statistically assessed using 2016/17 data and the data from 2017/18 were presented descriptively. Sensitivity analyses for primary and selected secondary outcomes (EYFSP, KS1, referral to CSCS) included adjusting for hypothesised confounders at baseline, dosage effects (i.e. number of visits) using complier average causal effects (CACE) modelling and sub-group analysis of potential effect moderators and mediators (maternal deprivation, adaptive functioning, NEET (not in education, employment or training) status at recruitment, maternal age at recruitment, child sex, maternal care status, duration of maternal care and domestic abuse self-reported at 24 months) as interaction terms in the main comparative models.

Results of all planned sensitivity analyses are presented in the appendix. An additional post hoc sensitivity analysis adjusted for the child's month of birth (categorised into quarter of birth) in the EYFSP and KS1 outcomes.

For peer review only

Economic Analysis

A cost consequences analysis of FNP over the full follow-up period (BB:0–2 and BB:2–6) took a secondary health care (UK NHS) perspective. The principal data source was Hospital Episode Statistics (HES) records (inpatient, outpatient, A&E), which were coded in Healthcare Resource Group (HRG) Grouper and matched to appropriate NHS Reference costs. Maternal and child resource use were costed separately and valued in £ sterling. Only direct healthcare costs were analysed. Indirect costs such as lost wages or childcare costs were not available within the routinely collected data. Where data were absent in HES records it was assumed no resource was incurred. No primary care data were available beyond 18 months postpartum. Costs were discounted back from year of event to baseline (2009/10) at an annual rate of 3.5% as described by Central Government's guidance on appraisal and evaluation.[15]

Data management

Small numbers were handled according to SAIL Databank rules where any cell counts under five were suppressed in reporting. Where abortion data are presented, we handled in accordance with the data sharing agreement with DHSC where counts less than 9 were suppressed. Data were analysed using Stata v16 and IBM SPSS v23.0.

Patient and public involvement

Activities involved four groups of young people (CASCADE Voices, Our Place, FNP graduates and ALPHA). These activities contributed to three aspects of the study: data-linkage methods (e.g. to optimise acceptability of the proposed dissent model, use of data linkage and communication of these methods)[16], study outcomes (by seeking views on the importance of the study outcomes) and dissemination of findings (including best way to present results, method of dissemination).

Results

The study population following the BB:0-2 trial tracing and matching process is shown in Figure 1. Five women were ineligible due to not meeting original trial entry criteria and 78 were mandatory withdrawals mainly due to fetal or infant death and adoption.[6] The 110 women who electively withdrew during the trial were given the opportunity to opt-out from further data usage, 16 of whom removed consent for any further contact. Thus 94 women who had electively withdrawn from BB:0-2 were included, and their details sent for tracing alongside the remaining 1,452 mothers (1,546 mother-child dyads in total) to update contact details to improve matching. One dyad was subsequently removed as deceased and the remaining 1,545 contacted by letter, email or text of whom eight dissented from having their records linked. 1,537 mothers and 1,547 children were sent to the DfE and

For peer review only

NHS Digital for matching. These formed the population for the BB:2-6 study. Match rates for children were 98.3% (NHS Digital) and 97.4% (DfE).

Numbers matched to and returned for analysis differed by data source and population (Table S3). Owing to having discontinued their education, few mothers, compared with children, were matched to any DfE data set (19% vs. 97%, respectively); these events were based mainly on the CIN data set. For selected maternal and child baseline variables and delivery of FNP assessed against programme fidelity goals, the BB:2-6 sample appears broadly representative of the original BB:0-2 cohort (Tables S4 and S5). Baseline characteristics of the BB:2-6 mothers and children were well balanced between trial arms (Table 1).

Table 1 Socio-demographic characteristics of mother and babies at baseline by trial arm

	FNP N=766	Usual care N=771
**Maternal age at recruitment (years)**	17·9 (17·0 to 18·8)	17·9 (16·9 to 18·8)
**Ethnicity**		
White background	675 (88·1%)	680 (88·2%)
Mixed background	44 (5·7%)	38 (4·9%)
Asian background	15 (2·0%)	10 (1·3%)
Black background	29 (3·8%)	40 (5·2%)
Chinese or Other background	<5	<5
**Relationship status with baby's father**		
Married	6 (0·8%)	10 (1·3%)
Separated	72 (9·4%)	78 (10·1%)
Closely involved/boyfriend	582 (76·0%)	586 (76·0%)
Just friends	106 (13·8%)	97 (12·6%)
**NEET Status***		
Yes	315/764 (41·1%)	319/769 (41·4%)
No	345/764 (45·0%)	333/769 (43·2%)
**IMD Overall Score†**	38·1 (24·6 to 52·6)‡	38·1 (25·5 to 51·6)§
**Generalised self-efficacy scale (score 10 to 40)¶**	30·0 (28·0 to 33·0)‖	30·0 (27·0 to 32·0)**
**Adaptive functioning:**		
**Difficulty in at least one basic skill**		
Yes	213/765 (27·8%)	184/770 (23·9%)
No	552/765 (72·2%)	586/770 (76·1%)
**Had 3 or less life skills (out of 5)**		

For peer review only - http://bmjopen.bmj.com/site/about/guidelines.xhtml

	FNP N=766	Usual care N=771
Yes	185/763 (24·2%)	218/769 (28·3%)
No	578/763 (75·8%)	551/769 (71·7%)
**At least one burden**		
Yes	214/759 (28·2%)	237/767 (30·9%)
No	545/759 (71·8%)	530/767 (69·1%)
**Cigarette smoking participant self-reported**		
Ever smoked		
Yes	615 (80·3%)	612 (79·4%)
No	151 (19·7%)	159 (20·6%)
**Child characteristics**	**FNP N=773**	**Usual Care N=774**
**Sex**		
Male	381/773 (49·3%)	406/773 (52·5%)
Female	392/773 (50·7%)	367/773 (47·5%)
**Birthweight (grams)** Mean (SD)	3223·81 (606·0)††	3215·52 (555·56)‡‡
**NNU admission (direct or subsequent)**		
Yes	76/716 (10·6%)	66/749 (8·8%)
No	640/716 (89·4%)	683/749 (91·2%)

Data are n (%), n/N (%), or median (25th to 75th centile) unless otherwise stated. <5 = numbers suppressed. FNP = Family Nurse Partnership Programme + Usual Care. IMD = Index of Multiple Deprivation. SD= standard deviation. NNU = Neonatal unit. *Definition of NEET status: Not in education employment or training (applicable only to those whose age at end of previous academic year at time of baseline interview was >16). †Higher IMD score indicated more deprivation. Mean IMD Score for England in 2010 was 21.67 [Wilkinson DL, Sniehotta FF, Michie S. Targeting those in need: Baseline data from the first English National Health Service (NHS) health trainer service: *Psychology, health & medicine* 2011; **16**(6): 736-48], ‡N=760. §N=765. ¶ Higher score indicates higher level of self-efficacy. ‖N=764. **N=769. ††N=724. ‡‡N=752.

For the primary outcome of a child in need, 277 referrals resulting in no further action were excluded and 634 referrals remained associated with a CIN status. Overall, 323 (21.5%) of children in the BB:2-6 cohort had at least one referral to CSCS which resulted as being classed as a child in need at any point between pregnancy (includes pre-birth CIN) and 6 years of age. No difference in rate of children in need was found between FNP (21.2%) and UC (21.7%) arms for the 1,506 children in the analysis (adjusted OR=0.98, 95% CI=0.74, 1.31) (Table 2). The parameter estimate was not affected when adjusting for the number of FNP visits received (efficacy per visit OR=1.00, 95% CI=0.99, 1.004).

There were no between arm differences for referral to CSCS, the age at which the first referral was made, the age at which the child was classified as in need and the proportion of children with a protection plan. A small number of children were ever Looked After within the 6-year follow-up

For peer review only - http://bmjopen.bmj.com/site/about/guidelines.xhtml

period, with no evidence of a difference between trial arms (Table 2). However, the UC arm experienced on average two more months in care compared to the FNP arm. The total number of child deaths across the full follow-up period was less than ten and not further reported by study arm, consistent with the non-disclosive data management policy. Data informing the disability outcome were not available for the years requested. In addition, no difference was found when examining age at first referral or CIN by study arm.

No between arm differences were found in children not taken to at least one outpatient appointment (did not attend rate), emergency attendances or hospital admissions for an injury or an ingestion (Table 2). Overall, 78% of mothers had a subsequent pregnancy (using inpatients, outpatients and abortions data) with no differences by arm (Table 3). Similarly, just over two thirds of the cohort had at least one registerable birth (using only admission data as a source) during the follow-up period with a comparable inter-birth interval. Descriptives for non-tested maltreatment outcomes are shown in Table S6.

For peer review only

For peer review only - http://bmjopen.bmj.com/site/about/guidelines.xhtml

Table 2 Primary and secondary outcomes: Objective and associated measures of maltreatment

**Outcome**	**FNP**	**Usual care (UC)**	**Adjusted* parameter estimate (95% CI)**	**p-value**	**Absolute risk difference (FNP-UC) (95% CI)**
**Child protection outcomes**	**N=760**	**N=746**			
Child in need (CIN) status at any time during the follow-up period					
No	599 (78·8%)	584 (78·3%)	Reference		
Yes	161 (21·2%)	162 (21·7%)	0·98† (0·74 to 1·31)	0·902	-0·5% (-0·5 to 0·4%)
Age at first CIN referral (days)					
Median (25th to 75th centile)	1602·5 (1153.0 to 1978·75)	1649·5 (1130·75 to 1980·25)	0·98‡ (0·79 to 1·23)	0·875	
Missing	*1*	*0*			
Unique§ number of CIN referrals per child	1 (1 to 2)	1 (1 to 2)	0·99‖ (0·82 to 1·19)	0·890	
CIN duration (days)	210 (70·5 to 571·25)	216 (72·75 to 503·25)	1·20‡ (0·90 to 1·59)	0·216	
CIN categorisation (primary need) of first case	***N=161***	***N=162***			
Abuse or neglect	92 (57·1%)	102 (63·0%)	Reference		
Family dysfunction	29 (18·0%)	34 (21·0%)	0·95¶ (0·54 to 1·69)	0·868	
Family in acute stress	13 (8·1%)	10 (6·2%)	1·47¶ (0·61 to 3·54)	0·389	

For peer review only - http://bmjopen.bmj.com/site/about/guidelines.xhtml

Outcome	FNP	Usual care (UC)	Adjusted* parameter estimate (95% CI)	p-value	Absolute risk difference (FNP-UC) (95% CI)
Low income, absent parenting, socially unacceptable behaviour, not stated	13 (8·1%)	6 (3·7%)	2·38‖ (0·86 to 6·57)	0·093	
Child/parent disability	7 (4·3%)	5 (3·1%)	1·47¶ (0·45 to 4·84)	0·525	
Cases other than CIN	7 (4·3%)	5 (3·1%)	1·64¶ (0·50 to 5·37)	0·417	
Referred to Children's social care services (CSCS)					
No	554 (72·9%)	538 (72·1%)	Reference		
Yes	206 (27·1%)	208 (27·9%)	0·97† (0·74 to 1·28)	0·829	-0·8% (-5·3 to 3·7%)
Age at first referral (days)<br>Median (25th to 75th centiles)	1541·5 (1272·75 to 1975·75)	1631·0 (1190·5 to 1984·75)	0·96‡ (0·79 to 1·17)	0·694	
Child Protection Plan					
No	708 (93·2%)	697 (93·4%)	Reference		
Yes	52 (6·8%)	49 (6·6%)	1·04† (0·69 to 1·57)	0·846	0·2% (-0·2 to 0·3%)
Category of child protection plan	***n=52***	***n=49***			
Neglect	17 (32·7%)	22 (44·9%)	Reference		
Emotional	22 (42·3%)	12 (24·5%)	2·77‖ (1·02 to 7·56)	0·046	
Physical	6 (11·5%)	6 (12·2%)	1·25‖ (0·32 to 4·88)	0·743	
Sexual and multiple**	7 (13·5%)	9 (18·4%)	1·13‖ (0·33 to 3·86)	0·848	

For peer review only - http://bmjopen.bmj.com/site/about/guidelines.xhtml

Outcome	FNP	Usual care (UC)	Adjusted* parameter estimate (95% CI)	p-value	Absolute risk difference (FNP-UC) (95% CI)
Looked after status††					
No	735 (96·7%)	719 (96·4%)	Reference		
Yes	25 (3·3%)	27 (3·6%)	0·90† (0·52 to 1·57)	0·712	-0·3% (-0·2 to 0·2%)
Child looked after period of care (months)	10·0 (4·5 to 37·5)	12·0 (6·0 to 33·0)	0·75‖ (0·65 to 0·86)	<0·001	
**Injuries and Ingestions**	**N=760**	**N=759**			
Emergency attendance‡‡					
None	317 (41·7%)	344 (45·3%)	Reference		
At least one	443 (58·3%)	415 (54·7%)	1·17† (0·95 to 1·45)	0·149	3·6% (-1·4 to 8·6%)
Number of attendances per child	2 (1 to 2)	2 (1 to 3)	1·09‖ (0·93 to 1·28)	0·281	
Admission to hospital					
None	671 (88·3%)	660 (87·0%)	Reference		
At least one	89 (11·7%)	99 (13·0%)	0·87† (0·63 to 1·20)	0·407	-1·3% (-4·7 to 2·0%)
Number of admissions per child	1 (1 to 1)	1 (1 to 1)	0·93‖ (0·67 to 1·29)	0·663	
Attendance and/or admission					
None	306 (40·3%)	324 (52·7%)	Reference		
At least one	454 (59·7%)	435 (57·3%)	1·11† (0·89 to 1·37)	0·351	2·4% (-2·5 to 7·4%)
DNA outpatient appointment	***N=580***	***N=577***			
Attended all appointments	290 (50·0%)	289 (50·1%)	Reference		

For peer review only

Outcome	FNP	Usual care (UC)	Adjusted* parameter estimate (95% CI)	p-value	Absolute risk difference (FNP-UC) (95% CI)
DNA at least once	290 (50·0%)	288 (49·9%)	1·00† (0·79 to 1·26)	0·997	0·09% (-5·6 to 5·8%)

Data are n (%), n/N (%), or median (25th to 75th centile) unless otherwise stated. FNP = Family Nurse Partnership Programme + Usual Care. * FNP compared to usual care. Analysis adjusted for stratification (site), minimisation variables (gestational age, smoking status at recruitment, and first or preferred language); † Odds ratio from logistic model; ‡ Hazard ratio from Cox model; § A unique referral is counted as a distinct referral date per child; ‖ Incidence rate ratio from Poisson model; ¶ Relative risk ratio from multinomial model; ‡‡Using diagnosis in any position; **Multiple indicates when more than one category of abuse is relevant to the child's current plan; †† We reflect here the terminology used at the time of the trial but note the more contemporary 'care experienced' as a more familiar and accepted term. Source: National Pupil Database, The Department for Education

For peer review only

Table 3 Secondary outcomes: Subsequent pregnancies and registerable birth

	FNP	Usual care	Adjusted* parameter estimate (95% CI)	p-value	Absolute risk difference (FNP-Usual care) (95% CI)
Subsequent pregnancy	***N=753***	***N=753***			
No	163 (21·7%)	163 (21·7%)	Reference		
Yes	590 (78·4%)	590 (78·4%)	1·00† (0·79 to 1·28)	0·984	0·0% (-4·2 to 4·2%)
Subsequent registerable birth	***N=752***	***N=749***			
No	276 (36·7%)	266 (35·5%)	Reference		
Yes	476 (63·3%)	483 (64·5%)	0·95† (0·77 to 1·18)	0·662	-1·2% (-6·0 to 3·7%)
One birth	326 (68·5)	331 (68·5)	0·95‡ (0·76 to 1·19)	0·655	
Two	124 (26·1)	121 (25·1)	0·99‡ (0·73 to 1·34)	0·955	
Three births or more	26 (5·5)	31 (6·4)	0·80‡ (0·46 to 1·39)	0·435	
Inter-birth interval between first and second child (days)<br>Median (25th to 75th centiles)	1027 (590 to 1506·75)	1065 (665 to 1538)	0·99§ (0·88 to 1·13)	0·938	

Data are n (%), n/N (%), or median (25th to 75th centile) unless otherwise stated. FNP = Family Nurse Partnership Programme + Usual Care. * FNP compared to usual care. Analysis adjusted for stratification (site), minimisation variables (gestational age, smoking status at recruitment, and first or preferred language); † Odds ratio from logistic model; ‡ Relative risk ratio from multinomial model; § Hazard ratio from Cox model.

For peer review only - http://bmjopen.bmj.com/site/about/guidelines.xhtml

Table 4 Secondary outcomes: Child health, developmental and educational

Outcome	FNP	Usual care	Adjusted* odds ratio (95% CI)	p-value	Absolute risk difference (FNP-Usual care) (95% CI)
Special educational needs (SEN)† provision	**N=759**	**N=747**			
No	540 (71·1%)	502 (67·2%)	Reference		
Yes	219 (28·9%)	245 (32·8%)	0·83 (0·67 to 1·03)	0·097	-3·9% (-8·6 to 0·7%)
**Early educational attendance**	**N=759**	**N=747**			
Attending an Ofsted registered private, voluntary and independent establishment up to the age of 4 years	334 (43·9%)	308 (41·2%)	1·13 (0·91 to 1·40)	0·281	2·7% (-2·3 to 7·7%)
**School attendance**	**N=754**	**N=740**			
Overall absence					
No absences	14 (1·9%)	14 (1·9%)	Ref		
At least one absence	740 (98·1%)	726 (98·1%)	1·00 (0·47 to 2·12)	0·998	0·0% (-1·4 to 1·5%)
Overall authorised absence					
No absences	26 (3·4%)	26 (3·5%)	Ref		
At least one absence	728 (96·6%)	714 (96·5%)	1·01 (0·58 to 1·75)	0·984	0·1% (-1·8 to 2.8%)
Overall unauthorised absence					
No absences	256 (34·0%)	245 (33·1%)	Ref		
At least one absence	498 (66·0%)	495 (66·9%)	0·95 (0·76 to 1·18)	0·620	-0·8% (-5·6 to 3·9%)

For peer review only - http://bmjopen.bmj.com/site/about/guidelines.xhtml

Outcome	FNP	Usual care	Adjusted* odds ratio (95% CI)	p-value	Absolute risk difference (FNP-Usual care) (95% CI)
**Early Years assessment**	**N=743**	**N=728**			
**Achieving good level of development (GLD) ‡**					
Achieving GLD in all five areas of learning‖	431 (58·0%)	380 (52·2%)	1·26 (1·03 to 1·55)	0·026	5·8% (0·7 to 10·9%)
Achieving GLD in all 17 early learning goals	412 (55·5%)	365 (50·1%)	1·24 (1·01 to 1·52)	0·043	5·3% (0·2 to 10·4%)
**Total point score**§ Mean (SD)	32·22 (7·25)	31·59 (7·62)	0·65 ‖ (-0·11 to 1·41)	0·094	
**Key Stage 1 assessments**	**N=740**	**N=732**			
**Reading**					
Lower than expected	257 (34·7%)	289 (39·5%)	Reference		
Reaching at least the expected standard¶	483 (65·3%)	443 (60·5%)	1·23 (0·99 to 1·53)	0·051	4·8 (-0·2 to 9·7%)
Expected standard	*371 (50·1%)*	*337 (46·0%)*	*1·24 (0·99 to 1·56)*	*0·056*	*4·1 (-1·0 to 9·2%)*
Higher standard	*112 (15·1%)*	*106 (14·5%)*	*1·20 (0·88 to 1·65)*	*0·250*	*0·7 (-3·0 to 4·3%)*
**Maths**					
Lower than expected	281 (38·0%)	283 (38·7%)	Reference		
Reaching at least the expected standard¶	459 (62·0%)	449 (61·3%)	1·04 (0·84 to 1·28)	0·731	0·7 (-4·3 to 5·6%)
Expected standard	*392 (53·0%)*	*376 (51·4%)*	*1·06 (0·85 to 1·32)*	*0·611*	*1·6 (-3·5 to 6·7%)*
Higher standard	*67 (9·1%)*	*73 (10·0%)*	*0·93 (0·64 to 1·35)*	*0·711*	*-0·9 (-3·9 to 2·1%)*

For peer review only - http://bmjopen.bmj.com/site/about/guidelines.xhtml

Outcome	FNP	Usual care	Adjusted* odds ratio (95% CI)	p-value	Absolute risk difference (FNP-Usual care) (95% CI)
**Science**					
Lower than expected	203 (27·4%)	219 (29·9%)	Reference		
Reaching at least the expected standard**	537 (72·6%)	513 (70·1%)	1·14 (0·91 to 1·43)	0·254	2·5 (-2·1 to 7·1%)
**Writing academic year 2016/17**	**N=498**	**N=487**			
Lower than expected	257 (51·6%)	278 (57·1%)	Reference		
Reaching at least the expected standard¶	241 (48·4%)	209 (42·9%)	1·24 (0·97 to 1·60)	0·090	5·5 (-0·7 to 11·6%)
Expected standard	*218 (43·8%)*	*182 (37·4%)*	*1·29 (1·00 to 1·68)*	*0·054*	*6·4 (0·3 to 12·5%)*
Higher standard	*23 (4·6%)*	*27 (5·5%)*	*0·92 (0·51 to 1·64)*	*0·769*	*-0·9 (-3·8 to 1·9%)*

Data are n (%), n/N (%), or median (25th to 75th centile) unless otherwise stated. FNP = Family Nurse Partnership Programme + Usual Care. SD=standard deviation. *FNP compared to usual care. Analysis adjusted for stratification (site), minimisation variables (gestational age, smoking status at recruitment, and first or preferred language); † A child with SEN provision is recorded using the following codes (A = School Action, P = School Action Plus, E = Education, Health and Care Plan, S=Statement, K= SEN support) and the N code was used to indicate No SEN support in any of the following datasets between 2013 and 2017. PLASC (Autumn, Spring and Summer terms), AP, PRU Census, and EYC. If for any of the years no response was recorded then the assumption was that the child was not included in the denominator as either not present on Census day or not in school; ‡ Children achieving a good level of development are those achieving at least the expected level within the prime and specific areas of learning; §Total point score ranges from 17 to 51 with a higher score indicating a better level of development; ‖ Mean difference from linear model; ¶ Working at the expected standard and at a greater depth within the expected standard; **Working at a greater depth within the expected standard is not applicable in science. Source: National Pupil Database, The Department for Education

For peer review only - http://bmjopen.bmj.com/site/about/guidelines.xhtml

Evidence of positive programme impacts were found for some educational outcomes assessed at the end of the reception year and at KS1 by which time the child would be seven years old (Table 4). Children in the FNP arm were more likely to reach a good level of development across all five areas of learning by the end of the reception year (58.0%) than children in the UC arm (52.2%) and also to achieve a good level of development in all 17 early learning goals (FNP: 55.5%, UC: 50.1%). The small advantage for children of nurse-visited mothers was consistent across all five areas of learning although not tested statistically (Table S7).

For KS1 reading, writing, maths and science assessments there was no evidence of a difference between arms in the proportion of children reaching at least the expected standard nor specifically in those working at the expected level or at a greater depth of knowledge (Table 4). What is observed from the writing assessment is the increase in the rates of children reaching at least the expected standard between the two academic years (Overall 45.7% in 2016/17 to 66.5% in 2017/18), reflective of all assessments across the two academic years in each arm (Table S8). This is mainly explained by the distribution of births for the children for each academic year (Figure S1). Reflecting the recruitment period of the trial, children with KS1 results available in the academic year 2016/17 were born between October 2009 and August 2010 but skewed towards the summer born children (42% born between June and August 2010) whereas children appearing in the 2017/18 academic year were born between September 2010 and February 2011. There is a nationally observed relationship between rates of children reaching the expected standard and month of birth e.g. a 17% difference in reading for children born in August and September.[17] Therefore, our 2017/18 cohort of children do not represent the whole academic year, but are biased towards those that are more likely to achieve. This indicates that the rates of children reaching the expected standard varies by their month of birth and is important as a moderator of programme effect. Importantly, the original trial allocation ensured study arms remained balanced with regards month of birth and the intervention had no impact on total weeks gestation.[6] As a sensitivity analysis, the main analyses were adjusted for the child's month of birth (categorised into quarter of birth) and evidence of a between arm difference was found in reading assessments (Table S9). In addition, the Early Years assessments were adjusted for child's month of birth, which strengthened the association found in Table 4.

Details of all planned sub-group analyses and further exploratory analyses are shown in the appendix (Tables S10 to S14).

Cost

There were negligible resource use and cost differences for both mothers (p=0.393) and children (p=0.865) between study arms (Table S15). The adjusted incremental costs of programme delivery per women in BB:0-2 (£1,811) remain the key observed cost difference between study arms.

For peer review only - http://bmjopen.bmj.com/site/about/guidelines.xhtml

Discussion

Principal findings

In this data linkage study we found that adding FNP to usually provided health and social care provided no additional benefit for maltreatment outcomes including the numbers of children referred to CSCS, registered as in need of additional support, given a child protection plan, entering care, attending an emergency department or being admitted for an injury or ingestion, or duration of stay if admitted. While there was no difference between study arms in the small number of children in care, the UC arm experienced on average two more months in care compared to the FNP arm. The total number of child deaths found was less than ten and therefore disclosive and cannot be reported. High rates of children ever registered as in need in both study arms indicates substantial need amongst families. We found the programme led to more children reaching a good level of development at the end of school reception year and, when taking month of birth into account, improvements in reading at KS1. Writing scores improved as a result of FNP for boys, children of younger mothers and of mothers who were not in education, employment or training when first recruited to the study. We found no other difference between families who received FNP and those who did not. With no differences in secondary health resource use and costs for FNP, the programme can be considered cost-neutral compared to usual care in the medium-term (2-6 years).

Strengths and weaknesses of the study

The prioritisation of outcomes in our original trial attracted some debate.[6,18-20] However, the trial and the current follow-on study are both comprehensively reported, and preceded by a priori analysis plans providing transparency and guarding against undue post hoc interpretation.[6-8] Although a primary outcome of child being in need of additional support was incorporated into the study design, we adopted a multimethod multisource approach to look at the range of maltreatment outcomes in drawing our conclusions. The consistent pattern of results within the two principal outcome domains provides greater confidence in what we can conclude. While the study benefited from random allocation in the BB:0-2 trial, when assessing educational outcomes we additionally adjusted for month of birth. This was because at a national level, month of birth is strongly associated with educational outcomes and the distribution of participants recruited to the trial varied by month. The potential to show benefit associated with additional support therefore differed by actual month of birth.

The available maltreatment data provides an essential overview of family experience as recorded in official records but may lack some depth which could have allowed a more nuanced understanding for children referred. The educational data are teacher-reported statutory tests but similarly provide less detailed insight into child functioning which research instruments may have provided. The lack of

For peer review only - http://bmjopen.bmj.com/site/about/guidelines.xhtml

detailed social care and any primary care resource use data limited the perspective of the economic analysis. However, using administrative data has enabled comprehensive cohort follow-up, with few cases lost for analysis preserving the benefits of the original trial randomisation. The data included are also objectively recorded rather than self-reported, allow for easy comparison nationally and over time. Our approach has gathered more data at less cost than comparable prospective data collection and minimised family burden.

Potential sources of bias

As an open label study, it is possible that service professionals may have attempted to compensate for the lack of specialist support from FNP for participants in the usual care arm. However, it should be noted that enhanced care would have been expected as part of usual practice for teenage mothers, for example, through teenage pregnancy midwives. The number of midwifery contacts were recorded in the trial and found to be equivalent between FNP and UC arms.[10] The number of home visits from specialist public health nurses (SPHN) reported by trial participants by 18 months was also equivalent between study arms. As would have been expected, the number of clinic contacts with SPHNs was greater for women in the control arm than in the FNP arm. In contrast, women allocated to the intervention arm would have received an average of just over 40 home visits from a family nurse before graduating from the programme. Overall, this suggests a common core of usual care provided across both trial arms, a higher level of SPHN input to the usual care arm reflecting in part the responsibility of delivering the Healthy Child Programme (which was formally allocated to FNP nurses rather than SPHNs for their clients). The capacity of SPHNs to provide additional support will also be limited by their caseload. The maximum caseload currently recommended by the Institute of Health Visiting is one practitioner per 250 children. However, in 2015, 28% of surveyed practitioners in England reported caseloads in excess of 400 children and 12% reported between 500 and 1000+ families.[21]

In categorising our outcomes we delineated between those which were objective indicators of maltreatment and other markers. This was to recognise those outcomes assessed independently of maternal report or presentation by third party agents - specifically local departments of children social services. However, both the presentation or referral of families and their assessment against legally defined statuses such as being a Child in Need still involves interpretation and remains a potential source of bias. It has been suggested that surveillance bias is not likely to be problematic for assessing maltreatment using verified service data (i.e. by inflating the numbers of children being reported when in receipt of home-visiting).[22-23] As others have found this could be higher when clients are actively engaged with services; the potential for such bias still needs to be acknowledged.[24]

Comparison with other studies

For peer review only - http://bmjopen.bmj.com/site/about/guidelines.xhtml

The strongest evidence for the programme preventing maltreatment remains from the original US Elmira trial.[25,26] While the programme reduced the number of substantiated reports of abuse and neglect in the first 15 years of the child's life, the advantage emerged after age four years.[26] We found differences in duration spent in care for looked after children, but the numbers involved are small and across the range of maltreatment outcomes the pattern is of no overall group difference. The programme was no more effective in tested sub-groups. It is worth recognising differences in the population of clients in receipt of the programme across the different study populations, their social circumstances (which also may have changed over time) and how that may determine potential to benefit from FNP.[10] We have not directly explored differences in social care systems between England and the US but these may vary the underlying risk of maltreatment and its likelihood of detection.[27]

School entry reading and maths is of important predictive value for later school achievement.[28] Early childhood educational investment programmes typically show a modest impact upon cognitive and achievement scores (eg weighted average of 0.21 standard deviations) with some decline in observed impact being attributed to general improvements in both school and home environments.[29,30] The question of comparator is therefore of particular relevance. In this study, a range of state and third sector funded services (including midwifery and health visiting) would have been available as usual care, possibly limiting the incremental benefit possible from the family nurse partnership programme.[10] Nevertheless, as noted above women allocated to FNP received substantial input from family nurses, albeit fewer than possible according to the maximum fidelity targets at the time of the trial.

Home visiting interventions, which aim to impact upon a range of outcomes, have shown similarly modest effects on child cognitive outcomes (eg mean Effect Size 0.25, 96% CI 0.11, 0.38) with considerable variability due to programme composition (eg greater effects with programmes addressing parental responsiveness, sensitivity to care and nurturing and rehearsal and role-playing).[31] Our BB:0-2 study found benefits at age two years for children in the FNP arm for both language and developmental outcomes but these outcomes were maternally reported and potentially subject to reporting bias in the open trial.[6] This new objectively obtained medium-term evidence for programme impact upon developmental outcomes adds to that picture, although the size of effect remains small. Similar medium-term programme impact (to age six years old) on developmental outcomes (eg, intellectual functioning and receptive language scores) was reported from the Memphis trial.[32] A reanalysis of those data found cognitive benefits at age 6 years attributed to both programme induced improvements in maternal traits and family life investments at age 2.[33]

Unanswered questions and future research

For peer review only - http://bmjopen.bmj.com/site/about/guidelines.xhtml

Given small but positive findings for developmental outcomes, tracking the cohort through to subsequent school years is warranted. As social care data are equally accessible via the NPD there is value in observing the trajectory for maltreatment outcomes given that the principal maltreatment findings from the US Elmira trial are located in the 15-year follow-up. Linkage to data in other sectors such as welfare would provide a greater understanding of broader impact for families. Such work should be driven by the programme's logic model, existing evidence for long-term programme impact, informed by programme evolution in England and supplemented by work to understand what may explain differing programme benefits across national settings.[26,34]

Conclusions

There are no evident programme benefits for maltreatment outcomes by age six but the FNP generates higher rates of child attainment at the end of the reception year and at KS1. The adapted programme remains locally commissioned and delivered in England.[34,35] Local needs and priorities may determine the weight attached to these different sets of outcomes.

Acknowledgements

The Centre for Trials Research receives funding from Health and Care Research Wales and Cancer Research UK. The work was undertaken with the support of The Centre for the Development and Evaluation of Complex Interventions for Public Health Improvement (DECIPHer), a UKCRC Public Health Research Centre of Excellence. Joint funding (MR/KO232331/1) from the British Heart Foundation, Cancer Research UK, Economic and Social Research Council, Medical Research Council, the Welsh Government and the Wellcome Trust, under the auspices of the UK Clinical Research Collaboration, is gratefully acknowledged.

Data re-use statement: Copyright © (2019), the Health and Social Care Information Centre. Re-used with the permission of the Health and Social Care Information Centre. All rights reserved.

We would like to thank all members of CASCADE voices, ALPHA and Our Place for contributing to the study including those who co-ordinated access to these individuals. Thanks are due to the FNP teams in Bristol, Bath and Wiltshire who helped us to make contact with FNP clients and graduates during our PPI work. We would also like to acknowledge the contribution of members of the study steering committee - Ann Louise Kinmonth (Chair), David Low, Gordon Taylor, Nina Biehal, Rachel Tonkin, Stavros Petrou, and Silvia van den Heijkant.

For peer review only - http://bmjopen.bmj.com/site/about/guidelines.xhtml

This work uses data provided by patients and collected by the NHS as part of their care and support as well as data from schools and local authorities. Using patient and other public data is vital to improve health and care for everyone. There is huge potential to make better use of information from people's patient records, to understand more about disease, develop new treatments, monitor safety, and plan NHS services. Patient and other public data should be kept safe and secure, to protect everyone's privacy, and it's important that there are safeguards to make sure that it is stored and used responsibly. Everyone should be able to find out about how patient data is used. #datasaveslives You can find out more about the background to this citation here: https://understandingpatientdata.org.uk/data-citation. [To note. This has been adapted to include other data sources not made available from the NHS such as education and social services data].

Footnotes

Author contributions: MR was the chief investigator and acts as guarantor for the study. MR, FLW, RCJ, LA, SC, DF, KH, JK, GM, EOJ, RP, JSa, JSe, TS contributed to study design. FLW was the study manager. RCJ was the lead statistician. DF supervised the health economics. MR, FLW, RCJ, LA, SC, DF, KH, JK, GM, EOJ, RP, JSa, JSe, TS were responsible for study management. GM and LA were the data managers. JK and JS led for public involvement. GM and LA cleaned the data. RCJ designed and conducted the analysis. RP conducted the economic analysis. MR, JSa, RCJ, SC, KH, JK, EOJ, JSe were co-applicants on the original funding application. MR, FLW, RCJ, LA, SC, DF, KH, JK, GM, EOJ, RP, JSa, JSe, TS interpreted the results of the analysis. RC-J and RP were responsible for producing the tables. MR, FLW, RC-J and RP drafted the study report. MR, FLW, RCJ, LA, SC, DF, KH, JK, GM, EOJ, RP, JSa, JSe, TS reviewed manuscript drafts, revised for important intellectual content and approved the final version.

Funding: This project was funded by the National Institute for Health Research Public Health Research (NIHR PHR) Programme (reference:11/3002/11). The views and opinions expressed therein are those of the authors and do not necessarily reflect those of the NIHR PHR Programme or the Department of Health. The funders had no role in considering the study design or in the collection, analysis, interpretation of data, writing of the report, or decision to submit the article for publication.

Competing interests: All authors have completed the ICMJE uniform disclosure form at www.icmje.org/coi_disclosure.pdf and declare: all authors declare financial support from NIHR PHR for the submitted work; no financial relationships with any organisations that might have an interest in the submitted work in the previous three years; no other relationships or activities that could appear to have influenced the submitted work.

For peer review only - http://bmjopen.bmj.com/site/about/guidelines.xhtml

Ethical approval: Ethical approval for this study was granted by the Health Research Authority research ethics committee (Wales REC 3; REC reference 14/WA10062) and section 251 support was provided by the Confidentiality Advisory Group (CAG reference CAG 10-08(b)/2014).

Data sharing: Anyone wishing to access the linked datasets for research purposes should apply via the CAG to the Health Research Authority to access patient identifiable data without consent and then to the NHS Digital, National Pupil Database and the abortion statistics – Department for Health and Social Care. In the first instance, enquiries about access to the data should be addressed to Professor Mike Robling, Centre for Trials Research, Cardiff University.

The lead author (MR) affirms that the manuscript is an honest, accurate, and transparent account of the study being reported; that no important aspects of the study have been omitted; and that any discrepancies from the study as planned (and, if relevant, registered) have been explained.

Dissemination to participants and related public communities: The findings from the BB:2-6 study, including those from this paper will be disseminated directly to participants where valid contact details remain available, via social media and conference presentations. This will include the use of lay-informed infographic materials. Summary results will also be made available on the study webpage [https://evidencinghomevisiting.co.uk/].

References

1. Olds DL, Henderson CR, Chamberlin R, Tatelbaum R. Preventing Child Abuse and Neglect: A Randomized Trial of Nurse Home Visitation. Pediatrics. 1986;78(1) [accessed 2017 May 5]. Available from: http://pediatrics.aappublications.org/content/pediatrics/78/1/65.full.pdf

2. Kitzman H, Olds DL, Henderson CR, Hanks C, Cole R, Tatelbaum R, et al. Effect of Prenatal and Infancy Home Visitation by Nurses on Pregnancy Outcomes, Childhood Injuries, and Repeated Childbearing. JAMA. 1997;278(8):644 [accessed 2017 May 5]. Available from: http://jama.jamanetwork.com/article.aspx?doi=10.1001/jama.1997.03550080054039

3. Olds DL, Robinson J, O'brien R, Luckey DW, Pettitt LM, Henderson CR, et al. Home Visiting by Paraprofessionals and by Nurses: A Randomized, Controlled Trial. Pediatrics. 2002;110(3) [accessed 2017 May 5]. Available from: http://pediatrics.aappublications.org/content/110/3/486.short

4. Mejdoubi J, Van Den Heijkant SCCM, Van Leerdam FJM, Heymans MW, Crijnen A, Hirasing RA. The effect of VoorZorg, the dutch nurse-family partnership, on child maltreatment and development: A randomized controlled trial. PLoS One. 2015;10(4).

5. Olds DL, Kitzman H, Anson E, Smith JA, Knudtson MD, Miller T, et al. Prenatal and infancy nurse home visiting effects on mothers: 18-year follow-up of a randomized trial. Pediatrics.

For peer review only - http://bmjopen.bmj.com/site/about/guidelines.xhtml

2019;144(6) [accessed 2020 Jul 23]. Available from: https://pediatrics.aappublications.org/content/144/6/e20183889

6. Robling M, Bekkers MJ, Bell K, Butler CC, Cannings-John R, Channon S, et al. Effectiveness of a nurse-led intensive home-visitation programme for first-time teenage mothers (Building Blocks): A pragmatic randomised controlled trial. Lancet. 2016;387:146–55. Available from: http://www.thelancet.com/journals/lancet/article/PIIS0140-6736(15)00392-X/fulltext

7. Lugg-Widger F, Cannings-John R, Channon Sue, Fitzsimmons D, Hood K, Jones K, et al. Assessing the medium-term impact of a home-visiting programme on child maltreatment in England: protocol for a routine data linkage study. BMJ Open. 2017;7(e015728) [accessed 2017 Jul 17]. Available from: http://bmjopen.bmj.com/content/bmjopen/7/7/e015728.full.pdf

8. Robling M, Bekkers MJ, Bell K, Butler CC, Cannings-John R, Channon S, et al. The Building Blocks Trial (Main report). 2014. Available from: https://www.cardiff.ac.uk/__data/assets/pdf_file/0009/504729/Building-Blocks-Full-Study-Report.pdf

9. Owen-Jones E, Bekkers M-J, Butler CC, Cannings-John R, Channon S, Hood K, et al. The effectiveness and cost-effectiveness of the Family Nurse Partnership home visiting programme for first time teenage mothers in England: a protocol for the Building Blocks randomised controlled trial. BMC Pediatr. 2013;13:1.

10. Robling M, Cannings-John R, Channon S, Hood K, Moody G, Poole R, et al. What is usual care for teenagers expecting their first child in England? A process evaluation using key informant mapping and participant survey as part of the Building Blocks randomised controlled trial of specialist home visiting. BMJ Open. 2018;8(5).

11. Lyons RA, Jones KH, John G, Brooks CJ, Verplancke J-P, Ford D V, et al. The SAIL databank: linking multiple health and social care datasets. BMC Med Inform Decis Mak. 2009;9(9) [accessed 2017 May 12]. Available from: http://www.biomedcentral.com/1472-6947/9/3

12. Jones KH, Ford D V., Jones C, Dsilva R, Thompson S, Brooks CJ, et al. A case study of the Secure Anonymous Information Linkage (SAIL) Gateway: A privacy-protecting remote access system for health-related research and evaluation. J Biomed Inform. 2014;50:196–204 [accessed 2017 May 12]. Available from: http://www.sciencedirect.com/science/article/pii/S1532046414000045

13. Jones KH, Ford DV, Thompson S, Lyons R. A Profile of the SAIL Databank on the UK Secure Research Platform. Int J Popul Data Sci. 2019;4(2) [accessed 2020 Jun 30]. Available from: https://ijpds.org/article/view/1134

14. Department for Education. Children In Need in England, including their characteristics and further information on children who were the subject of a child protection plan (2009-10 Children in Need census, Final. 2010.

15. HM Treasury. The Green Book, Central Government Guidance on Appraisal and Evaluation. 2020. [accessed 2020 Dec 7]. Available from: https://assets.publishing.service.gov.uk/government/uploads/system/uploads/attachment_data/file/938046/The_Green_Book_2020.pdf

16. Lugg-widger F, Cannings-john R, Angel L, Moody G, Segrott J, Kenkre J, et al. Assessing the impact of specialist home visiting upon maltreatment in England : a feasibility study of data linkage from a public health trial to routine health and social care data. Pilot feasibility Stud. 2018;4(1):98.

17. Department for Education. Phonics screening check and Kay stage 1 assessments in England, 2016 (SFR 42/2016). 2016.

For peer review only - http://bmjopen.bmj.com/site/about/guidelines.xhtml

18. Olds D. Building evidence to improve maternal and child health. Vol. 387, The Lancet. Lancet Publishing Group; 2016. p. 105–7 [accessed 2020 Jul 23]. Available from: http://dx.doi.org/10.1016/S0140-6736

19. Barlow J, Barnes J, Sylva K, Fonagy P, Fearon P. Questioning the outcome of the Building Blocks trial. Vol. 387, The Lancet. Lancet Publishing Group; 2016. p. 1615–6 [accessed 2020 Jul 23]. Available from: http://www.dh.gov.uk/

20.. Robling M, Torgerson D, Pickett K, Channon S, Sanders J. Questioning the outcome of the Building Blocks trial - Authors' reply. Vol. 387, The Lancet. Lancet Publishing Group; 2016. p. 1616–7 [accessed 2020 Jul 23]. Available from: http://www.dh.gov.uk/

25. Zielinski DS, Eckenrode J, Olds DL. Nurse home visitation and the prevention of child maltreatment: Impact on the timing of official reports. Dev Psychopathol. 2009;21(2):441–53 [accessed 2019 Aug 2]. Available from: https://www.cambridge.org/core/product/identifier/S0954579409000248/type/journal_article

26. Olds DL, Eckenrode J, Henderson CR, Kitzman H, Powers J, Cole R, et al. Long-term Effects of Home Visitation on Maternal Life Course and Child Abuse and Neglect Fifteen-Year Follow-up of a Randomized Trial. JAMA. 1997;278(8):637–43.

27. Moody G, Cannings-John R, Hood K, Kemp A, Robling M. Establishing the international prevalence of self-reported child maltreatment: A systematic review by maltreatment type and gender. Vol. 18, BMC Public Health. BioMed Central Ltd.; 2018. p. 1164 [accessed 2020 Jul 23]. Available from: https://bmcpublichealth.biomedcentral.com/articles/10.1186/s12889-018-6044-y

28. Duncan GJ, Dowsett CJ, Claessens A, Magnuson K, Huston AC, Klebanov P, et al. School Readiness and Later Achievement. Dev Psychol. 2007;43(6):1428–46.

29. Duncan G, Magnusson K. Early childhood interventions for low-income children. Focus (Madison). 2014;31.

30. Duncan G, Magnuson K. Investing in Preschool Programs. J Econ Perspect. 2013;27(2):109–31.

31. Filene JH, Kaminski JW, Valle LA, Cachat P. Components associated with home visiting program outcomes: A meta-analysis. Pediatrics. 2013;132(SUPPL.2):S100–9 [accessed 2021 Jan 8]. Available from: www.pediatrics.org/cgi/doi/10.1542/peds.2013-1021H

32. Olds DL, Kitzman H, Cole R, Robinson J, Sidora K, Luckey DW, et al. Effects of Nurse Home-Visiting on Maternal Life Course and Child Development: Age 6 Follow-Up Results of a Randomized Trial. Pediatrics. 2004;114(6):1550–9. Available from: http://pediatrics.aappublications.org/cgi/doi/10.1542/peds.2004-0962

33. Heckman JJ, Holland ML, Makino KK, Pinto R, Rosales-Rueda M. An Analysis of the Memphis Nurse-Family Partnership Program. NBER Work Pap. 2017;23610. Available from: http://www.nber.org/papers/w23610%0Ahttp://www.nber.org/papers/w23610

34. Family Nurse Partnership National Unit & Dartington Service Design Lab. FNP ADAPT: Using evidence, pragmatism and collaboration to change the FNP programme in England. London, UK; 2020. Available from: www.fnp.nhs.uk

35. The Family Nurse Partnership |. [accessed 2020 Jul 23]. Available from: https://www.fnp.nhs.uk/

For peer review only

Figure Legend

Figure 1 Participant flow chart

Describing follow-up within the BB0:0-2 trial and formation of the BB:2-6 study cohort. MW=Mandatory withdrawals; EW=elective withdrawals; M=Mother; C=Child, FNP=Family Nurse Partnership (trial arm); UC=Usual care (trial arm); BB:0-2=Building Blocks Randomised Controlled Trial; BB:2-6=Building Blocks Cohort Study; NPD=National Pupil Database

For peer review only

For peer review only - http://bmjopen.bmj.com/site/about/guidelines.xhtml

Figure 1 Flow chart

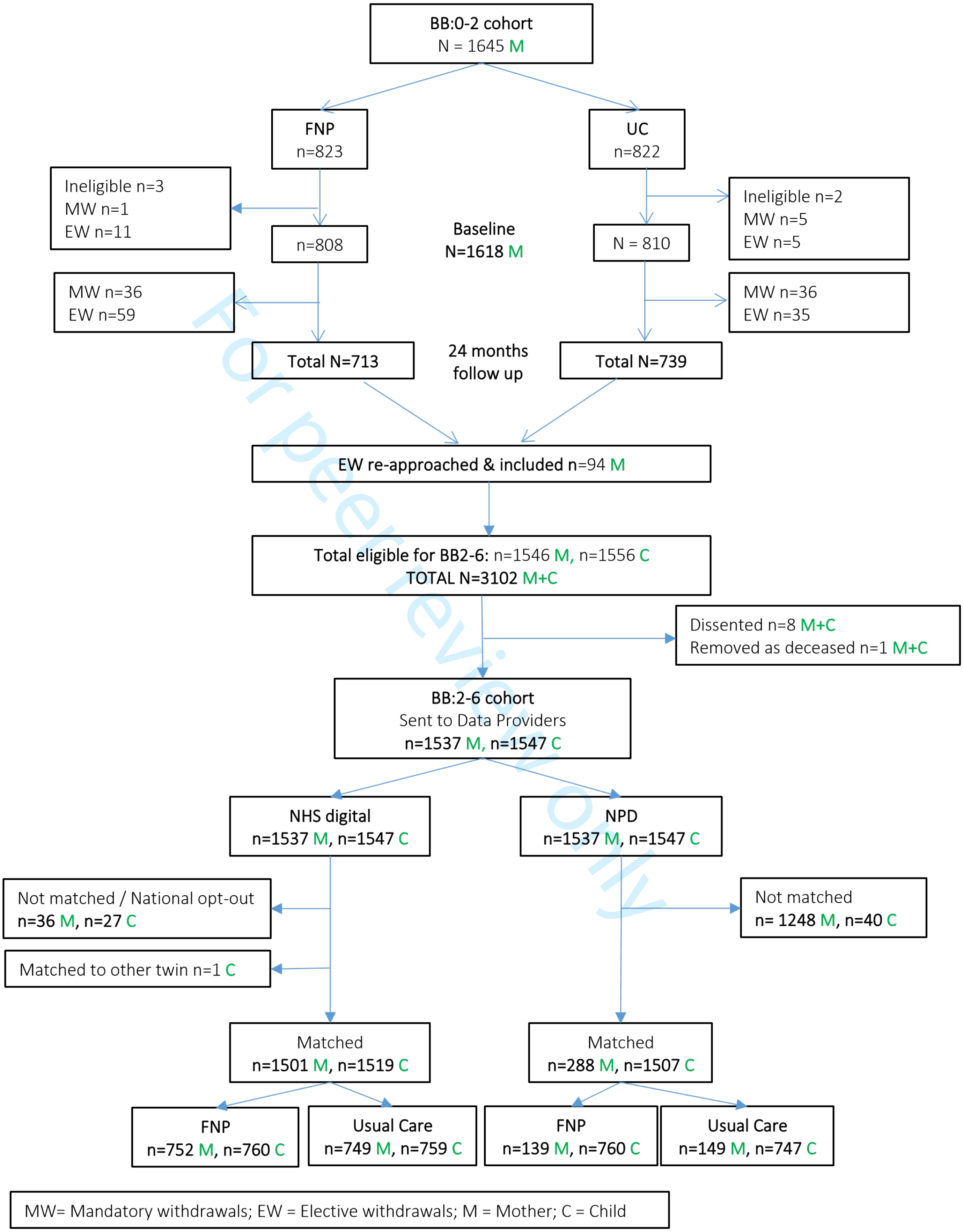

For peer review only

Supplementary appendix

I. Number of valid FNP visits received

- Table S1 BB:2-6 study population data: Valid FNP visits per phase

II. Study assessment domain, outcomes, principal data providers and data obtained

- Table S2 BB:2-6 Study assessment domains, key and exploratory outcomes and principal data providers

III. Data accrued from providers

- Table S3 Numbers (%) of mothers and children with data returned by dataset

IV. Further details of Child in Need outcome

V. Representativeness of study sample

- Table S4 Maternal characteristics of those in BB:0-2 compared to those in the BB:2-6
- Table S5 Child characteristics of those in the BB:0-2 study compared to those in the BB:2-6 study

VI. Descriptive secondary outcomes and additional Key Stage 1 results

- Table S6 Objective and associated measures of maltreatment: descriptives
- Table S7 Child health, developmental and educational: descriptives
- Table S8 Children reaching the expected standard in KS1 assessments by trial arm and academic year
- Figure S1 Distribution of children's month of birth by trial arm
- Table S9 Comparison of parameter estimates before and after adjustment for child's month of birth

VII. Results of planned sub-group analysis

- Table S10 Subgroup analyses for referrals to Children's social care services
- Table S11 Subgroup analyses for child in need status
- Table S12 Predefined subgroup analyses on Early Years Assessment total point score
- Table S13 Subgroup analyses on KS1 assessments reaching the expected standard (RES)
- Table S14 Subgroup analyses on reaching the expected standard in the writing KS1 assessment for 2016/17 academic year

VIII. Treatment efficacy

IX. Missing data

X. Cost Consequence Analysis

- Table S15 Cost-consequence balance sheet for the family-nurse based intervention for the four years between children's ages 2 and 6.

For peer review only - http://bmjopen.bmj.com/site/about/guidelines.xhtml

For peer review only

I. Number of valid FNP visits received

Of the 766 women in the FNP arm in the BB:2-6 cohort, 69 did not enrol for FNP. For the remaining 697 who were randomised and enrolled, 695 had received at least one valid visit and two did not receive any valid visits across the three phases (pregnancy, infancy and toddlerhood). The descriptives in Table S1 are based on 697 women. A greater proportion of women received no visits during the infancy and toddler phases (40 (5.7%) and 98 (14.1%) respectively) compared to the pregnancy phase (6 (0.7%)). This is similar to that reported in the BB:0-2 cohort with comparable median valid visits and proportions reaching the fidelity goals (see Lancet Supplementary appendix Table S11.3).[1]

Table S1 BB:2-6 study population data: Valid FNP visits per phase

	**Pregnancy**	**Infancy**	**Toddlerhood**
N women randomised and enrolled to FNP	697	697	697
Women not receiving any valid visits n(%)	6 (0·7)	40 (5·7)	98 (14·1)
Women receiving at least one valid visit n(%)	691 (99·3)	657 (94·3)	599 (85·9)
Valid visits received			
Median (25th to 75th centile)	10 (8 to 12)	19 (15 to 22)	13 (10 to 16)
Mean (SD)	9·74 (3·42)	18·7 (5·97)	13·28 (5·46)
Reaching fidelity goal of: n(%)			
80%+	401/697 (57·5)	-	-
65%+	-	364/697 (52·2)	-
60%+	-	-	297/697 (42·6)

[1] Robling M, Bekkers MJ, Bell K, Butler CC, Cannings-John R, Channon S, et al. Effectiveness of a nurse-led intensive home-visitation programme for first-time teenage mothers (Building Blocks): A pragmatic randomised controlled trial. Lancet. 2016;387:146–55. [accessed 2019 Nov 12]. Available from: http://www.thelancet.com/journals/lancet/article/PIIS0140-6736(15)00392-X/fulltext

For peer review only - http://bmjopen.bmj.com/site/about/guidelines.xhtml

II. Study assessment domain, outcomes and principal data providers

Table S2 BB:2-6 Study assessment domains, key and exploratory outcomes and principal data providers

**Domains**	**Outcomes**	**Maternal**	**Child**	**HES**	**ONS**	**NPD**	**Data source**	**Analysis**
Primary: Child in Need status recorded at any time during the follow-up period.	CIN status		✓			✓	CIN	LO
	Exploratory:<br>*Age at first CIN referral*<br>*Number of CIN referrals per child*		 ✓<br>✓			 ✓<br>✓	 CIN<br>CIN	 *SURV*<br>*PO*
Secondary:<br>(i) Objective measures of maltreatment	CIN categorisation<br>CIN duration		✓<br>✓			✓<br>✓	CIN<br>CIN	MNOM<br>SURV
	Referral to Children's social care services		✓			✓	CIN	LO
	Exploratory: Age at first referral		✓			✓	CIN	SURV
	Child Protection Plan<br>Child Protection Plan categorisation		✓<br>✓			✓<br>✓	CIN<br>CIN	LO<br>MNOM
	Looked after status<br>CLA period of care<br>Legal status of CLA		✓<br>✓<br>✓			✓<br>✓<br>✓	CLA<br>CLA<br>CLA	LO<br>PO<br>D
	Cause of death		✓		✓		ONS- deaths	D
(ii) Associated measures of maltreatment	Injuries and ingestions:<br>Emergency attendance<br>Admission to hospital<br>Attendance and/or admission<br>DNA appointments		 ✓<br>✓<br>✓<br>✓	 ✓<br>✓<br>✓<br>✓			 A&E<br>IP<br>A&E, IP<br>OP	 LO<br>LO<br>LO<br>LO
	Exploratory:							

For peer review only - http://bmjopen.bmj.com/site/about/guidelines.xhtml

Domains	Outcomes	Maternal	Child	HES	ONS	NPD	Data source	Analysis
	Number of admissions per child		✓	✓			IP	PO
	Length of hospital admission stay		✓	✓			IP	D
(iii) Maternal outcomes	Subsequent pregnancy	✓		✓			OP, IP, Ab	LO
	Exploratory:							
	Number of births	✓		✓			IP	PO
	Inter-birth interval between first and second child	✓		✓			IP	SURV
(iv) Costs	Health and Social Care resource use	✓	✓	✓		✓	OP, IP, A&E matched with NHS reference costs	D
(v) Child health, developmental and educational outcomes	Special educational needs provision		✓	✓		✓	PLASC, AP, PRU Census, EYC	LO
	Disability		✓			✓	NA	NA
	Early education attendance		✓			✓	PLASC, EYC	LO
	School attendance		✓			✓	Absence	LO
	Early Years assessment		✓			✓	EYFSP	LIN, LO
	KS1 attainment		✓			✓	KS1	LO
	Exploratory:							
	Early education attendance by School or an Ofsted registered PVI establishment		✓			✓	PLASC, EYC	D
	Earliest type of day care		✓			✓	EYC	D

Ab - Abortions; A&E – Accident and Emergency; AP – Alternative Provision; CIN – Child in Need; CLA – Child Looked After; D – Descriptive; DNA – Did not attend; EYC – Early Years Census; EYFSP –Early years foundation stage profile; FNP – Family Nurse Partnership; HES – Hospital Episode Statistics; IP - Inpatients; KS1 – Key Stage 1; LIN – Linear regression; LO – Logistic; MNOM - multinomial NA - Not available; NPD – National Pupil Database; ONS – Office for National Statistics; OP – Outpatients; PLASC – Pupil Level and School Census; PO-Poisson; PRU – Pupil Referral Unit Census.; PVI – Private, Voluntary or Independent (PVI) establishment; SURV – Survival.

For peer review only - http://bmjopen.bmj.com/site/about/guidelines.xhtml

III. Data accrued from providers

Table S3 Numbers (%) of mothers and children with data returned by dataset

	Mothers	Children
**Sent for matching to DfE and NHSD**	**N=1537**	**N=1547**
**Department for Education (DfE)**		
Not matched	1248 (81·2)	40 (2·6)
Match to any DfE dataset	289 (18·8)	1507 (97·4)
Pupil Level Annual School Census (PLASC)	<5	1498 (96·8)
Child in Need (CIN)	278 (18·1)	414 (26·8)
Children looked after (CLA)	61 (4·0)	52 (3·4)
Early years foundation stage profile (EYFSP)	N/A	1476 (95·4)
Key Stage one (KS1)	N/A	1472 (95·2)
Early Years Census (EYC)	N/A	642 (41·5)
Pupil referral unit (PRU) Census	<5	0
Alternative Provision (AP)	<5	<5
Absence	<5	1494 (96·6)
**NHS Digital (NHSD)**		
Not matched	36 (2·3)	27 (1·7)
Duplicate match	0	1 (0·06)
Matched to any NHSD dataset	1501 (97·7)	1519 (98·3)
Accident and Emergency (A&E)	1360 (88·5)	1392 (90·0)
Admitted Patient Care (Inpatients) (APC)	1498 (97·5)	1507 (97·4)
Outpatient (OP)	1495 (97·3)	1189 (76·9)
**Sent for matching to Department of Health and Social Care (DHSC)**	**N=1532**	**0**
Abortions dataset	1532 (100)	NA

For peer review only

For peer review only - http://bmjopen.bmj.com/site/about/guidelines.xhtml

IV. Further details of Child in Need outcome.

In our primary comparative analysis we examined whether the firstborn ('BB:0-2 child') had ever been referred to social services and classed as a child in need at any point between birth and 6 years of age. There is no flag in the Child In Need dataset to identify whether a child is in need after they have been referred to social services. Therefore, CIN was defined (as per DfE methodology)[1] as all children referred to social services, excluding cases where:

- the referral is flagged as being a referral resulting in no further action; or
- the only activity recorded is an assessment and the reason for the closure of the case being that case was closed after assessment with no further action.

1. Department for Education. Characteristics of children in need in England: 2014 to 2015. 2015. [accessed 2019 Nov 12]. Available from: https://www.gov.uk/government/uploads/system/uploads/attachment_data/file/469740/SFR41-2015_Methodology.pdf

V. Representativeness of study sample

Scope and purpose: We assessed whether the study sample for BB:2-6 were similar to women recruited to the original BB:0-2 trial.

Conclusion: The BB:2-6 and BB:0-2 cohorts are similar in terms of maternal (Table S4) and baby characteristics (Table S5).

Table S4 Maternal characteristics of those in BB:0-2 compared to those in the BB:2-6

	**BB:0-2 N=1618**	**BB:2-6 N=1537**
**Minimisation variables at randomisation**		
**Gestational age at baseline** n(%)		
16 weeks or more	658 (40·7)	636 (41·4)
Less than 16 weeks	959 (59·3)	900 (58·6)
**Smoking status** n(%)		
No	860 (53·2)	822 (53·5)
Yes	758 (46·8)	715 (46·5)
**Language** n(%)		
English	1611 (99·6)	1530 (99·5)
Other	6 (0·4)	6 (0·4)
Missing	*1 (0·0)*	*0 (0·0)*

For peer review only - http://bmjopen.bmj.com/site/about/guidelines.xhtml

	BB:0-2 N=1618	BB:2-6 N=1537
**Trial arm** n(%)		
FNP	808 (49·9)	766 (49·8)
Usual care	810 (50·1)	771 (50·2)
**Age at recruitment (years) Median (25th to 75th centile)**	17·9 (16·9 to 18·8)	17·9 (16·9 to 18·8)
**Ethnicity** n(%)		
White background	1425 (88·1)	1355 (88·2)
Mixed background	89 (5·5)	82 (5·3)
Asian background	27 (1·7)	25 (1·6)
Black background	71 (4·4)	69 (4·5)
Chinese or Other background	6 (0·4)	6 (0·4)
**Relationship status with baby's father** n(%)		
Married	20 (1·2)	16 (1·0)
Separated	165 (10·2)	150 (10·0)
Closely involved/boyfriend	1222 (75·5)	1168 (76·0)
Just friends	211 (13·0)	203 (13·2)
**Live with father of baby** n(%)		
Yes	368 (22·7)	348 (22·6)
No	1112 (68·7)	1061 (69·0)
Missing	138 (8·5)	128 (8·3)
**NEET status**[a] n(%)		
Yes	663 (41·0)	634 (41·2)
No	717 (44·3)	678 (44·1)
Missing	*4 (0·2)*	*4 (0·3)*
Not applicable (aged 16 years or younger)	*234 (14·5)*	*221 (14·4)*
**Socio-economic status: Overall Index of Multiple Deprivation (IMD) Score**[b] Median (25th to 75th centile)	N=1606 38·2 (25·2 to 52·0)	N=1525 38·1 (25·0 to 52·1)
**Maternal health and wellbeing**		
**Generalized self-efficacy scale (score 10 to 40)**[c] Median (25th to 75th centile)	N=1592 30·0 (28·0 to 33·0)	N=1533 30·0 (28·0 to 33·0)
**Adaptive functioning: Difficulty in at least one basic skill** n(%)		
Yes	430 (26·6)	397 (25·8)
No	1185 (73·2)	1138 (74·0)
Missing	*3 (0·2)*	*2 (0·1)*
**Adaptive functioning: Had 3 or less life skills** n(%)		
Yes	434 (26·8)	403 (26·2)
No	1178 (72·8)	1129 (73·5)
Missing	*6 (0·4)*	*5 (0·3)*
**Adaptive functioning: At least one burden** n(%)		
Yes	476 (29·4)	451 (29·3)
No	1131 (69·9)	1075 (69·9)
Missing	*11 (0·7)*	*11 (0·7)*
**Smoking** (Participant self-reported) n(%)		
Ever smoked: Yes	1291 (79·8)	1227 (79·8)
No	327 (20·0)	310 (20·2)
Missing	*0 (0)*	*0 (0)*
**Child characteristics**	**BB:0-2 sample N=1510**	**BB:2-6 sample N=1547**
**Trial arm** n(%)		

For peer review only - http://bmjopen.bmj.com/site/about/guidelines.xhtml

	BB:0-2 N=1618	BB:2-6 N=1537
FNP	742 (49·1)	773 (50·0)
Usual Care	768 (50·9)	774 (50·0)
**Gender** n(%)		
Male	777 (51·5)	787 (50·9)
Female	732 (48·5)	758 (40·1)
Missing	1	72[d]
**Birthweight (grams) Mean (sd)**	3207·3 (599·6)	3219·6 (580·7)
Missing[c]		71
**Neonatal unit (NNU) admission** n(%)		
Yes	149 (10·0)	142 (9·2)
Missing from data collection	13	11
Missing	Not applicable	71[d]

[a] *Definition of NEET status: Not in education employment or training (applicable only to those whose age at end of previous academic year at time of baseline interview was >16)*
[b] *Higher IMD score indicated more deprivation. Mean IMD Score for England in 2010 was 21·67 [60];*
[c] *Higher score indicates higher level of self-efficacy*
[d] *Mothers were withdrawn in BB:0-2 but are included in BB:2-6*

Table S5 Child characteristics of those in the BB:0-2 study compared to those in the BB:2-6 study

	BB:0-2 sample N=1510	BB:2-6 sample N=1547
**Trial arm** n(%)		
FNP	742 (49·1)	773 (50·0)
Usual Care	768 (50·9)	774 (50·0)
**Gender** n(%)		
Male	777 (51·5)	787 (50·9)
Female	732 (48·5)	758 (40·1)
Missing	1	72[a]
**Birthweight (grams) Mean (sd)**	3207·3 (599·6)	3219·6 (580·7)
Missing[a]		71
**Neonatal unit (NNU) admission** n(%)		
Yes	149 (10·0)	142 (9·2)
Missing from data collection	13	11
Missing[a]	Not applicable	71*

[a] *Mothers were withdrawn in BB:0-2 but are included in BB:2-6*

For peer review only - http://bmjopen.bmj.com/site/about/guidelines.xhtml

VI. Descriptive secondary outcomes and additional Key Stage 1 results

Table S6 Objective and associated measures of maltreatment: descriptives

Outcome	FNP	Usual care (UC)
**Child in Need (CIN) Referrals**	***N=161***	***N=162***
Age at first CIN referral (days)		
Pre birth	<5	<5
Birth to <1 years	<5	<5
1 to <2 years	6 (3·7%)	6 (3·7%)
2 to <3 years	15 (9·4%)	28 (17·3%)
3 to <4 years	27 (16·9%)	23 (14·2%)
4 to <5 years	47 (29·4%)	39 (24·1%)
5 to <6 years	33 (20·6%)	41 (25·3%)
6 to <7 years	20 (12·5%)	20 (12·3%)
Missing	*1*	*0*
Unique* number of CIN referrals per child		
1 case	110 (68·3%)	110 (67·9%)
2 cases	42 (26·1%)	42 (25·9%)
3 or more cases	9 (5·5%)	10 (6·1%)
CIN cases closed at 31st March 2017	116 (72·0%)	110 (67·9%)
**Referral to Children's social care services (CSCS)**	***N=760***	***N=746***
Total referrals made N	339	354
Ratio referrals:children	1·65	1·70
Referrals made per child	***N=206***	***N=208***
1	159 (77·2%)	140 (67·3%)
2	34 (16·5%)	51 (24·5%)
3 to 5	13 (6·3%)	27 (8·2%)
**Unit=referral (referred to CSCS encompass any referral)**	***n=339***	***n=354***
Source of referral		
Individual	29 (8·8%)	34 (10·0%)
School/Education	150 (45·7%)	136 (40·0%)
Health services	114 (34·8%)	132 (38·8%)
Local Authority Services	35 (10·7%)	38 (11·2%)
Missing	*11*	*14*
Primary need of referrals		
Abuse or neglect	198 (58·9%)	205 (57·9%)
Family dysfunction	72 (21·4%)	88 (24·9%)
Family in acute stress	19 (5·7%)	21 (5·9%)
Low income/absent parenting/ socially unacceptable behaviour	11 (3·3%)	7 (2·0%)
Parental/Childs disability illness	14 (4·2%)	11 (3·1%)
Cases other than Child in Need	11 (2·3%)	12 (3·4%)
Not stated	11 (3·3%)	9 (2·5%)
Missing	*3*	*1*
Legal status of child looked after†	***N=17***	***N=23***
Interim care order	6 (35·3%)	8 (34·8%)
Full care order	5 (29·4%)	6 (26·1%)

For peer review only

For peer review only - http://bmjopen.bmj.com/site/about/guidelines.xhtml

Outcome	FNP	Usual care (UC)
Placement order granted	<5	<5
Single period of accommodation under section 20	<5	<5
**Injuries and Ingestions: Admission to hospital**	**N=760**	**N=759**
N unique admissions	109	119
Ratio admissions: children	1.22	1.20
Length of hospital stay (days)	0·5‡ (0·5 to 1·0)	0·5 (0·5 to 1·0)
	**N=760**	**N=746**
Death	<10	

Data are n (%), n/N (%), or median (25th to 75th centile) unless otherwise stated. <5 = numbers suppressed· FNP = Family Nurse Partnership Programme + Usual Care. * A unique referral is counted as a distinct referral date per child. † Legal status for most recent CLA episode (Of n=52 children with a recent CLA episode, n=12 cases have a missing status). ‡ 0·5 days indicates an admission and discharge on the same day (could be up to 1 day in hospital). Source: National Pupil Database, The Department for Education

For peer review only

Table S7 Child health, developmental and educational: descriptives

Outcome	FNP	Usual care	Absolute risk difference (FNP-Usual care) (95% CI)
**Early educational attendance**	**N=759**	**N=747**	
Children attending education* up to the age of 4 years			
Yes†	758 (99·7%)	747 (100·0%)	-0·3% (-1·0 to 0·3%)
Children attending school up to the age of:			
• 4 years†	753 (99·1%)	744 (99·6%)	-0·5% (-1·5 to 0·4%)
• 3 years	479 (63·0%)	482 (64·5%)	
• 2 years	137 (18·0%)	147 (19·7%)	
Earliest type of day care	***N=334***	***N=308***	
Private	217 (65·0%)	221 (71·8%)	
Voluntary	52 (15·6%)	57 (18·5%)	
Local Authority Day Nursery	36 (10·8%)	22 (7·1%)	
Other‡	29 (8·7%)	8 (2·6%)	
**School attendance**	**N=754**	**N=740**	
Number of academic sessions§	630 (264 to 638)	630 (262 to 638)	
Overall absence rate Median (25th to 75th centiles)	5·08 (2·69 to 8·73)	5·19 (2·73 to 8·41)	
Overall authorised absence rate Median (25th to 75th centiles)	3·50 (1·96 to 6·09)	3·79 (2·02 to 6·11)	
Overall unauthorised absence rate Median (25th to 75th centiles)	1·84 (0·76 to 4·34)	1·57 (0·63 to 3·88)	
**Early Years assessment**	**N=743**	**N=728**	
Achieving good level of development (GLD) ‖:			
• Communication and Language	642 (86·4%)	613 (84·2%)	2·2% (-1·4 to 5·8%)
• Physical Development	676 (91·0%)	643 (88·3%)	2·7% (-0·5 to 5·8%)

For peer review only - http://bmjopen.bmj.com/site/about/guidelines.xhtml

Outcome	FNP	Usual care	Absolute risk difference (FNP-Usual care) (95% CI)
• Personal, Social and Emotional Development	668 (89·9%)	634 (87·1%)	2·8% (-0·4 to 6·1%)
• Literacy	493 (66·4%)	458 (62·9%)	3·4% (-1·4 to 8·3%)
• Maths	545 (73·4%)	516 (70·9%)	2·5% (-2·1 to 7·0%)
Total point score¶			
• Score <34	325 (43·8%)	345 (47·4%)	
• Score = 34	187 (25·2%)	183 (25·1%)	
• Score >34	231 (31·1%)	200 (27·5%)	
**Key Stage 1 assessments**			
Writing academic year 2017/18	**N=242**	**N=245**	
Lower than expected	82 (33·9%)	81 (33·1%)	
Reaching at least the expected standard**	160 (66·1%)	164 (66·9%)	-0·8 (-9·2 to 7·5)
Expected standard	140 (57·9%)	132 (53·9%)	4·0 (-4·8 to 12·7)
Higher standard	20 (8·3%)	32 (13·1%)	-4·8 (-10·4 to 0·7)

Data are n (%), n/N (%), or median (25th to 75th centile) unless otherwise stated. FNP = Family Nurse Partnership Programme + Usual Care.
* School or an Ofsted registered Private, Voluntary or Independent (PVI) establishment;
† = Not estimable; ‡ Childminding Network, Childminder, Registered Independent School, Other; § Total number of sessions over the academic years available (2013 to Spring 2017) and all 6 terms per academic year. ‖ Children achieving a good level of development are those achieving at least the expected level within the prime and specific areas of learning; ¶ Total point score ranges from 17 to 51 with a higher score indicating a better level of development. ** Working at the expected standard and at a greater depth within the expected standard. PLASC (Autumn, Spring and Summer terms), AP, PRU Census, and EYC. If for any of the years no response was recorded then the assumption was that the child was not included in the denominator as either not present on Census day or not in school. Source: National Pupil Database, The Department for Education

For peer review only

Table S8 Children reaching the expected standard in KS1 assessments by trial arm and academic year

		Academic year 2016/17	Academic year 2017/18
Reading	FNP	302/498 (60.6%)	181/242 (74.8%)
	Usual care	263/487 (54.0%)	180/245 (73.5%)
Maths	FNP	280/498 (56.2%)	179/242 (74.0%)
	Usual care	263/487 (54.0%)	186/245 (75.9%)
Science	FNP	341/498 (68.5%)	196/242 (81.0%)
	Usual care	310/487 (63.7%)	203/245 (82.9%)

Source: National Pupil Database, The Department for Education

Figure S1 Distribution of children's month of birth by trial arm

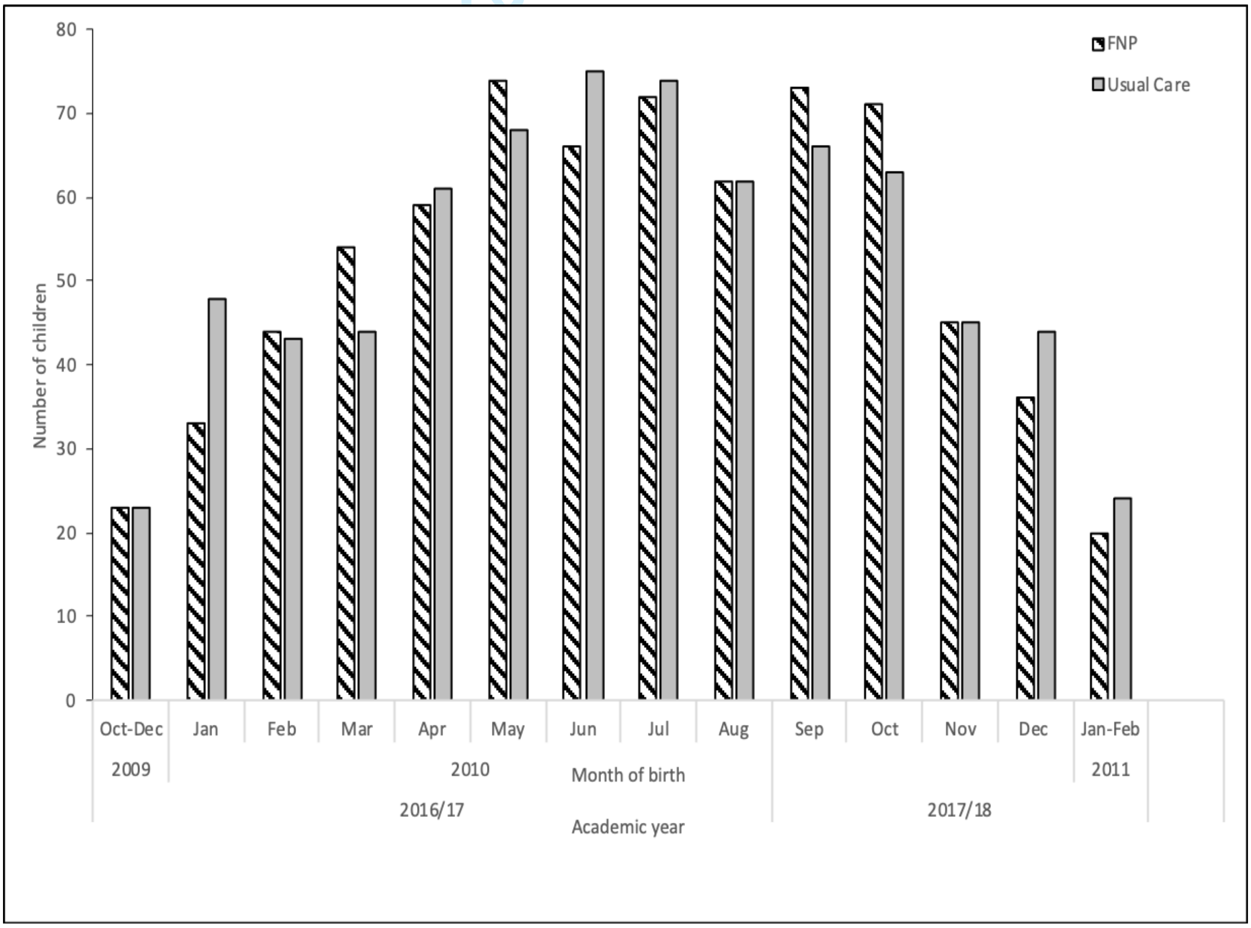

Table S9 Comparison of parameter estimates before and after adjustment for child's month[a] of birth

	Odds ratio[b] (95% CI), p-values	
	Adjusted for site and minimisation variables[c]	Adjusted for site, minimisation variables[c] and month of birth
**Key Stage 1 outcome**		
**Reading**		
Reaching at least the expected standard	1.23 (0.99 to 1.53), 0.051	1.26 (1.02 to 1.57), 0.035
Expected standard	*1.24 (0.99 to 1.56), 0.056*	*1.27 (1.01 to 1.59), 0.042*
Higher standard	*1.20 (0.88 to 1.65), 0.250*	*1.26 (0.91 to 1.75), 0.161*
**Maths**		
Reaching at least the expected standard	1.04 (0.84 to 1.28), 0.731	1.06 (0.85 to 1.31), 0.613
Expected standard	*1.06 (0.85 to 1.32), 0.611*	*1.07 (0.86 to 1.34), 0.527*
Higher standard	*0.93 (0.64 to 1.35), 0.711*	*0.93 (0.74 to 1.17), 0.522*
**Science**		
Reaching at least the expected standard	1.14 (0.91 to 1.43), 0.254	1.16 (0.92 to 1.47), 0.197
**Writing academic year 2017/18**		
Reaching at least the expected standard	1.24 (0.97 to 1.60), 0.090	1.26 (0.98 to 1.62), 0.076
Expected standard	*1.29 (1.00 to 1.68), 0.054*	*1.30 (1.00 to 1.69), 0.050*
Higher standard	*0.92 (0.51 to 1.64), 0.769*	*0.97 (0.54 to 1.75), 0.917*
**Early Years Assessment**		
Achieving GLD in all five areas of learning[d]	1.26 (1.03 to 1.55), 0.026	1.31 (1.05 to 1.62), 0.015
Achieving GLD in all 17 early learning goals	1.24 (1.01 to 1.52), 0.043	1.27 (1.03 to 1.58), 0.027
Total point score	0.65 (-0.11 to 1.41), 0.094	0.70 (-0.03 to 1.42), 0.060

a Quarter of birth runs from September to November, December to February, March to May, June to August; b Odds ratio represents the FNP arm compared to Usual care; c gestational age, smoking status at recruitment, and first or preferred language; d Children achieving a good level of development (GLD) are those achieving at least the expected level within the prime and specific areas of learning.

Source: National Pupil Database, The Department for Education

For peer review only

VII. Results of planned sub-group analysis

No differential effects due to maternal age, deprivation, participation in employment, education or training, or basic life skills were found in planned sub-group analyses for any of the objective maltreatment outcomes (Table S10 and Table S11). The beneficial impact of FNP on EYFSP total point score was mostly observed for children with mothers aged <16 years rather than those aged ≥16 at study entry but no other differences by study sub-group were found (Table S12).

The differential effects due to maternal age, deprivation, participation in employment, education or training, or basic life skills for KS1 reading, maths and science are displayed in Tables S13a to e and for writing in Table S14. At KS1, sex differences were large with girls outperforming boys in all four subject areas (Table S13a). The intervention effect was greater for boys than girls with a higher proportion reaching the expected standard in the FNP arm, particularly for reading and writing (and strengthened after adjusting for month of birth) and for the writing assessment (academic year 2016/17) this interaction reached statistical significance after adjusting for month of birth (post-hoc analysis). When examining how the intervention effects differed in mothers aged less than 16 years at recruitment compared to mothers aged 16 years or more in each KS1 assessment, differences were observed in maths (Table S13b) and writing (Table S14), with a larger effect seen in mothers aged less than 16 at recruitment, when compared to those aged 16 or more.

Table S10 Subgroup analyses for referrals to Children's social care services

N(%) referred	FNP	Usual care	Adjusted[a] OR (95% CI), p-value	Interaction p-value
**Child Gender**				
Boys	99 (26.3)	108 (27.7)	0.93 (0.65 to 1.33), 0.69	0.78
Girls	107 (27.9)	100 (28.1)	1.01 (0.69 to 1.48), 0.94	
**Maternal age at recruitment**				
<16 years	17 (34.7)	19 (34.6)	1.05 (0.44 to 2.46), 0.92	0.96
≥16 years	189 (26.6)	189 (27.4)	0.96 (0.72 to 1.30), 0.81	
**Adaptive functioning**				
No difficulty	140 (25.4)	149 (26.3)	0.99 (0.71 to 1.38), 0.96	0.67
Difficulty in basic skills	66 (31.7)	59 (33.0)	0.81 (0.59 to 1.41), 0.67	
**NEET status**				
EET	70 (20.3)	75 (23.1)	0.83 (0.54 to 1.29), 0.42	0.18
NEET	100 (32.5)	89 (29.2)	1.19 (0.80 to 1.78), 0.39	
**Deprivation quintile**				

For peer review only - http://bmjopen.bmj.com/site/about/guidelines.xhtml

1 – least deprivation	33 (20.4)	34 (23.9)	0.77 (0.44 to 1.36), 0.37	0.32
2	36 (23.8)	30 (20.5)	1.26 (0.61 to 2.60), 0.53	
3	42 (28.8)	43 (27.9)	0.96 (0.50 to 1.86), 0.91	
4	41 (31.1)	39 (25.3)	1.27 (0.69 to 2.31), 0.44	
5 – most deprivation	54 (33.3)	61 (42.4)	0.65 (0.35 to 1.22), 0.18	

[a] FNP compared to usual care. Analysis adjusted for stratification (site), minimisation variables (gestational age, smoking status at recruitment, and first or preferred language). N/EET – Not/In Education, Employment or Training (Source: National Pupil Database, The Department for Education)

Table S11 Subgroup analyses for child in need status

N(%) CIN	FNP	Usual care	Adjusted[a] OR (95% CI), p-value	Interaction p-value
**Gender**				
Boys	80 (21.3)	80 (20.5)	1.05 (0.72 to 1.53), 0.81	0.56
Girls	81 (21.1)	82 (23.0)	0.91 (0.60 to 1.38), 0.65	
**Maternal age at recruitment**				
<16 years	14 (28.6)	16 (29.1)	1.00 (0.41 to 2.43), 0.99	0.98
≥16 years	147 (20.7)	146 (21.1)	0.98 (0.72 to 1.33), 0.88	
**Adaptive functioning**				
No difficulty	106 (19.2)	113 (20.0)	0.99 (0.70 to 1.42), 0.98	0.76
Difficulty in basic skills	55 (26.4)	49 (27.4)	0.93 (0.59 to 1.47), 0.76	
**NEET**				
EET	52 (16.0)	52 (15.1)	0.93 (0.57 to 1.53), 0.78	0.42
NEET	81 (26.3)	72 (23.6)	1.18 (0.77 to 1.77), 0.46	
**Deprivation quintile**				
1 – least deprivation	24 (14.8)	22 (15.5)	0.92 (0.49 to 1.73), 0.79	0.62
2	30 (19.9)	22 (15.1)	1.53 (0.68 to 3.47), 0.31	
3	32 (21.9)	36 (23,4)	0.89 (0.52 to 1.54), 0.68	
4	32 (24.2)	34 (22.1)	1.15 (0.64 to 2.06), 0.64	
5 – most deprivation	43 (26.5)	48 (33.3)	0.72 (0.38 to 1.38), 0.32	

[a] FNP compared to usual care. Analysis adjusted for stratification (site), minimisation variables (gestational age, smoking status at recruitment, and first or preferred language). N/EET – Not/In Education, Employment or Training (Source: National Pupil Database, The Department for Education).

For peer review only

Table S12 Subgroup analyses for Early Years Assessment total point score

Mean (SD) total point score	FNP	Usual care	Adjusted[a] mean difference (95% CI), p-value	Interaction p-value
**Gender**				
Boys	N=369<br>30.57 (7.33)	N=383<br>29.84 (7.56)	0.75 (-0.31 to 1.82), 0.17	0.57
Girls	N=374<br>33.85 (6.79)	N=345<br>33.53 (7.22)	0.34 (-0.67 to 1.35), 0.51	
**Maternal age at recruitment**				
<16 years	N=45<br>32.44 (6.71)	N=52<br>28.91 (7.70)	3.65 (0.76 to 6.55), 0.013	0.046
≥16 years	N= 698<br>32.21 (7.28)	N= 676<br>31.80 (7.58)	0.42 (-0.36 to 1.21), 0.29	
**Adaptive functioning**				
No difficulty	N=540<br>32.99 (7.12)	N=552<br>32.11 (7.65)	0.88 (0.003 to 1.75), 0.049	0.50
Difficulty in basic skills	N=203<br>30.18 (7.20)	N=175<br>29.94 (7.33)	0.34 (-1.11 to 1.79), 0.65	
**NEET status**				
In EET	N=339<br>32.45 (7.12)	N=322<br>32.37 (7.68)	0.15 (-0.99 to 1.28), 0.80	0.95
NEET	N= 304<br>31.72 (7.57)	N=299<br>31.50 (7.43)	0.19 (-1.00 to 1.38), 0.76	
**Index of Multiple Deprivation quintiles**				
1 = least deprived	N=159<br>33.16 (7.44)	N=139<br>31.53 (8.24)	1.72 (-0.07 to 3.50), 0.060	ref
2	N=148<br>32.16 (6.97)	N=144<br>31.59 (7.05)	0.65 (-0.96 to 2.27), 0.43	0.84
3	N=146<br>32.16 (6.79)	N=151<br>32.59 (7.27)	-0.20 (-1.79 to 1.39), 0.80	0.21
4	N=128<br>30.95 (7.53)	N=148<br>31.11 (7.50)	-0.31 (-2.11 to 1.48), 0.73	0.85
5 = most deprived	N=156<br>32.41 (7.46)	N=140<br>31.03 (8.12)	1.45 (-0.33 to 3.22), 0.11	0.74

[a] *FNP compared to usual care. Analysis adjusted for stratification (site), minimisation variables (gestational age, smoking status at recruitment, and first or preferred language). N/EET – Not/In Education, Employment or Training (Source: National Pupil Database, The Department for Education).*

Table S13 Subgroup analyses for KS1 assessments reaching the expected standard (RES)

(a) Gender

N(%)	Boys		Girls	
	**Not RES**	**RES**	**Not RES**	**RES**
**Reading**				
FNP	152 (41.8)	212 (58.2)	105 (27.9)	271 (72.1)
Usual care	182 (47.3)	203 (52.7)	107 (30.8)	240 (69.2)
Adjusted[a] OR (95% CI), p-value	1.27 (0.95 to 1.69), 0.11		1.16 (0.84 to 1.59), 0.38	
Interaction p-value=0.68				

For peer review only - http://bmjopen.bmj.com/site/about/guidelines.xhtml

Adjusted[b] OR (95% CI), p-value	1.30 (0.96 to 1.75), 0.09		1.17 (0.84 to 1.62), 0.36	
Interaction p-value=0.68				
**Maths**				
FNP	149 (40.9)	215 (59.1)	132 (35.1)	244 (64.9)
Usual care	166 (43.1)	219 (56.9)	117 (33.7)	230 (66.3)
Adjusted[a] OR (95% CI), p-value	1.11 (0.83 to 1.49), 0.49		0.95 (0.69 to 1.29), 0.72*	
Interaction p-value=0.46				
Adjusted[b] OR (95% CI), p-value	1.14 (0.84 to 1.54), 0.40		0.94 (0.69 to 1.29), 0.71	
Interaction p-value=0.47				
**Science**				
FNP	120 (33.0)	244 (67.0)	83 (22.1)	293 (77.9)
Usual care	138 (35.8)	247 (64.2)	81 (23.3)	266 (76.7)
Adjusted[a] OR (95% CI), p-value	1.16 (0.86 to 1.58), 0.34		1.08 (0.76 to 1.53), 0.68	
Interaction p-value=0.77				
Adjusted[b] OR (95% CI), p-value	1.18 (0.87 to 1.63), 0.28		1.08 (0.76 to 1.55), 0.66	
Interaction p-value=0.72				

RES = Reaching the expected standard; [a] Analysis adjusted for stratification (site), minimisation variables (gestational age, smoking status at recruitment, and first or preferred language); [b] Analysis adjusted for stratification (site), minimisation variables (gestational age, smoking status at recruitment, and first or preferred language) and month of birth. (Source: National Pupil Database, The Department for Education).

(b) Maternal age at recruitment

N(%)	Maternal age<16 years		Maternal age≥16 years	
	**Not RES**	**RES**	**Not RES**	**RES**
**Reading**				
FNP	21 (44.7)	26 (55.3)	236 (34.1)	457 (65.9)
Usual care	29 (52.7)	26 (47.3)	260 (38.4)	417 (61.6)
Adjusted[a] OR (95% CI), p-value	1.21 (0.97 to 1.52), 0.09		1.49 (0.65 to 3.41), 0.34	
Interaction p-value=p=0.66				
Adjusted[b] OR (95% CI), p-value	1.50 (0.65 to 3.48), 0.34		1.25 (0.99 to 1.57), 0.07	
Interaction p-value=0.62				
**Maths**				
FNP	16 (34.0)	31 (66.0)	265 (38.2)	428 (61.8)
Usual care	32 (58.2)	23 (41.8)	251 (37.1)	426 (62.9)
Adjusted[a] OR (95% CI), p-value	3.15 (0.94 to 10.48), 0.06		0.96 (0.77 to 1.19), 0.69	
Interaction p-value=0.011				

For peer review only - http://bmjopen.bmj.com/site/about/guidelines.xhtml

Adjusted[b] OR (95% CI), p-value	3.23 (1.36 to 7.67), 0.008		0.98 (0.78 to 1.22), 0.83	
Interaction p-value=0.014				
**Science**				
FNP	18 (38.3)	29 (61.7)	185 (26.7)	508 (73.3)
Usual care	25 (45.4)	30 (54.6)	194 (28.7)	483 (71.3)
Adjusted[a] OR (95% CI), p-value	1.54 (0.53 to 4.47), 0.42		1.11 (0.88 to 1.41), 0.39	
Interaction p-value=0.56				
Adjusted[b] OR (95% CI), p-value	1.37 (0.59 to 3.19), 0.467		1.14 (0.89 to 1.45), 0.29	
Interaction p-value=0.69				

RES = Reaching the expected standard; [a] FNP compared to Usual care. Analysis adjusted for stratification (site), minimisation variables (gestational age, smoking status at recruitment, and first or preferred language); [b] Analysis adjusted for stratification (site), minimisation variables (gestational age, smoking status at recruitment, and first or preferred language) and month of birth. (Source: National Pupil Database, The Department for Education).

(c) Adaptive functioning

N(%)	No difficulty with basic skills		Difficulty with basic skills	
	**Not RES**	**RES**	**Not RES**	**RES**
**Reading**				
FNP	152 (30.5)	347 (69.5)	87 (42.9)	116 (57.1)
Usual care	188 (36.2)	332 (63.8)	84 (48.0)	91 (52.0)
Adjusted[a] OR (95% CI), p-value	1.29 (0.98 to 1.70), 0.07		1.23 (0.82 to 1.86), 0.32	
Interaction p-value=0.61				
Adjusted[b] OR (95% CI), p-value	1.29 (0.98 to 1.69), 0.07		1.25 (0.83 to 1.91), 0.29	
Interaction p-value=0.95				
**Maths**				
FNP	186 (34.6)	351 (65.4)	95 (46.8)	108 (53.2)
Usual care	192 (34.5)	364 (65.5)	91 (52.0)	84 (48.0)
Adjusted[a] OR (95% CI), p-value	1.01 (0.78 to 1.29), 0.97		1.27 (0.84 to 1.93), 0.26	
Interaction p-value=0.38				
Adjusted[b] OR (95% CI), p-value	1.01 (0.78 to 1.31), 0.93		1.32 (0.86 to 2.03), 0.20	
Interaction p-value=0.28				
**Science**				
FNP	130 (24.2)	407 (75.8)	73 (36.0)	130 (64.0)
Usual care	153 (27.5)	403 (72.5)	66 (37.7)	109 (62.3)
Adjusted[a] OR (95% CI), p-value	1.20 (0.92 to 1.58), 0.19		1.10 (0.71 to 1.69), 0.68	
Interaction p-value=0.71				

For peer review only

Adjusted[b] OR (95% CI), p-value	1.22 (0.93 to 1.62), 0.16	1.13 (0.73 to 1.74), 0.59
Interaction p-value=0.82		

RES = Reaching the expected standard; [a] FNP compared to Usual care. Analysis adjusted for stratification (site), minimisation variables (gestational age, smoking status at recruitment, and first or preferred language); [b] Analysis adjusted for stratification (site), minimisation variables (gestational age, smoking status at recruitment, and first or preferred language) and month of birth. (Source: National Pupil Database, The Department for Education).

(d) Not in Education, employment or training (NEET) status

N(%)	NEET		In EET	
	Not RES	RES	Not RES	RES
**Reading**				
FNP	105 (31.2)	232 (68.8)	109 (36.0)	194 (64.0)
Usual care	115 (35.8)	206 (61.2)	121 (40.5)	178 (59.5)
Adjusted[a] OR (95% CI), p-value	1.26 (0.91 to 1.75), 0.17		1.21 (0.87 to 1.69), 0.26	
Interaction p-value=0.86				
Adjusted[b] OR (95% CI), p-value	1.31 (0.94 to 1.83), 0.17		1.25 (0.89 to 1.76), 0.19	
Interaction p-value=0.83				
**Maths**				
FNP	122 (36.2)	215 (63.8)	122 (40.3)	181 (59.7)
Usual care	107 (33.3)	214 (66.7)	120 (40.1)	179 (59.9)
Adjusted[a] OR (95% CI), p-value	0.89 (0.65 to 1.23), 0.48		0.99 (0.72 to 1.38), 0.97	
Interaction p-value=0.64				
Adjusted[b] OR (95% CI), p-value	0.93 (0.66 to 1.29), 0.65		1.03 (0.73 to 1.42), 0.90	
Interaction p-value=0.68				
**Science**				
FNP	87 (25.8)	250 (74.2)	821 (27.1)	221 (72.9)
Usual care	78 (24.3)	243 (75.7)	98 (32.8)	201 (67.2)
Adjusted[a] OR (95% CI), p-value	0.93 (0.65 to 1.33), 0.69		1.31 (0.92 to 1.87), 0.14	
Interaction p-value=0.20				
Adjusted[b] OR (95% CI), p-value	0.96 (0.67 to 1.39), 0.84		1.36 (0.95 to 1.95), 0.10	
Interaction p-value=0.22				

RES = Reaching the expected standard; [a] FNP compared to Usual care. Analysis adjusted for stratification (site), minimisation variables (gestational age, smoking status at recruitment, and first or preferred language); [b] Analysis adjusted for stratification (site), minimisation variables (gestational age, smoking status at recruitment, and first or preferred language) and month of birth. N/EET – Not/In Education, Employment or Training (Source: National Pupil Database, The Department for Education).

For peer review only - http://bmjopen.bmj.com/site/about/guidelines.xhtml

(e) Index of Multiple Deprivation quintiles

	N(%)	Q1=least deprived		Q2		Q3		Q4		Q5=most deprived	
		Not RES	RES	Not RES	RES	Not RES	RES	Not RES	RES	Not RES	RES
Reading	FNP	49 (30.3)	113 (67.7)	53 (36.3)	93 (63.7)	49 (33.6)	97 (66.4)	48 (37.2)	81 (62.8)	57 (37.8)	94 (62.2)
	Usual care	54 (38.9)	85 (61.1)	64 (44.1)	81 (55.9)	48 (31.8)	103 (68.2)	64 (43.0)	85 (57.0)	56 (39.4)	86 (60.6)
	Adjusted[a] OR (95% CI), p-value	1.57 (0.88 to 2.77), 0.12		1.42 (0.88 to 2.29), 0.16		0.96 (0.59 to 1.57), 0.88		1.33 (0.81 to 2.18), 0.25		1.07 (0.60 to 1.89), 0.83	
	Adjusted[b] OR (95% CI), p-value	1.75 (1.00 to 3.07), 0.05		1.42 (0.85 to 2.37), 0.18		0.89 (0.53 to 1.48), 0.64		1.41 (0.86 to 2.34), 0.18		1.00 (0.57 to 1.75), 0.99	
	Interaction p-value=0.70										
	Interaction p-value=0.37										
Maths	FNP	56 (34.6)	106 (65.4)	57 (39.0)	89 (61.0)	50 (34.3)	96 (65.7)	55 (42.6)	74 (57.4)	62 (41.1)	89 (58.9)
	Usual care	52 (37.4)	87 (62.6)	60 (41.4)	85 (58.6)	56 (37.1)	95 (62.9)	60 (40.3)	89 (59.7)	54 (38.0)	88 (62.0)
	Adjusted[a] OR (95% CI), p-value	1.15 (0.72 to 1.85), 0.56		1.12 (0.69 to 1.82), 0.65		1.17 (0.69 to 1.97), 0.56		0.93 (0.57 to 1.52), 0.77		0.91 (0.56 to 1.47), 0.69	
	Adjusted[b] OR (95% CI), p-value	1.36 (0.82 to 2.24), 0.23		1.12 (0.68 to 1.82), 0.66		1.11 (0.65 to 1.90), 0.69		0.98 (0.60 to 1.62), 0.95		0.88 (0.54 to 1.44), 0.62	
	Interaction p-value=0.91										
	Interaction p-value=0.81										

For peer review only - http://bmjopen.bmj.com/site/about/guidelines.xhtml

	N(%)	Q1=least deprived		Q2		Q3		Q4		Q5=most deprived	
		**Not RES**	**RES**	**Not RES**	**RES**	**Not RES**	**RES**	**Not RES**	**RES**	**Not RES**	**RES**
**Science**	FNP	37 (22.8)	125 (77.2)	36 (24.7)	110 (75.3)	40 (27.4)	106 (72.6)	45 (34.9)	84 (65.1)	44 (29.1)	107 (70.9)
	Usual care	45 (32.4)	94 (67.6)	45 (31.0)	100 (69.0)	35 (23.2)	116 (76.8)	47 (31.5)	102 (68.5)	46 (32.4)	96 (67.6)
	Adjusted[a] OR (95% CI), p-value	1.68 (1.00 to 2.81), 0.048		1.39 (0.80 to 2.41), 0.25		0.82 (0.46 to 1.47), 0.51		0.89 (0.52 to 1.51), 0.65		1.24 (0.74 to 2.06), 0.42	
	Adjusted[b] OR (95% CI), p-value	1.94 (1.13 to 3.30), 0.015		1.40 (0.76 to 2.57), 0.28		0.78 (0.43 to 1.40), 0.41		0.92 (0.55 to 1.55), 0.76		1.16 (0.69 to 1.96), 0.57	
	Interaction p-value=0.25										
	Interaction p-value=0.11										

RES = Reaching the expected standard; a FNP compared to Usual care; [a] Analysis adjusted for stratification (site), minimisation variables (gestational age, smoking status at recruitment, and first or preferred language); [b] Analysis adjusted for stratification (site), minimisation variables (gestational age, smoking status at recruitment, and first or preferred language) and month of birth. (Source: National Pupil Database, The Department for Education).

For peer review only

Table S14 Subgroup analyses for reaching the expected standard in the writing KS1 assessment for 2016/17 academic year

N(%)	FNP	Usual care	Adjusted[a] OR (95% CI), p-value	Interaction p-value	Adjusted[b] OR (95% CI), p-value	Interaction p-value
**Gender**						
Boys	109 (42.9)	83 (31.8)	1.61 (1.13 to 2.32), 0.009	0.034	1.62 (1.13 to 2.33), 0.009	0.039
Girls	132 (54.1)	126 (55.8)	1.03 (0.76 to 1.39), 0.87		0.94 (0.65 to 1.37), 0.76	
**Maternal age at recruitment**						
<16 years	18 (54.6)	9 (22.5)	4.67 (1.47 to 14.84), 0.009	0.016	5.28 (1.49 to 18.73), 0.010	0.018
≥16 years	223 (48.0)	200 (44.7)	1.13 (0.87 to 1.47), 0.35		1.15 (0.89 to 1.50), 0.29	
**Adaptive functioning**						
No difficulty	189 (52.5)	174 (46.7)	1.26 (0.93 to 1.70), 0.14	0.72	1.26 (0.93 to 1.70), 0.14	0.67
Difficulty in basic skills	52 (37.7)	34 (30.1)	1.41 (0.77 to 2.58), 0.26		1.53 (0.81 to 2.90), 0.19	
**NEET**						
EET (no not EET)	109 (47.8)	119 (52.2)	0.82 (0.56 to 1.19), 0.29	0.019	0.82 (0.56 to 1.19), 0.29	0.028
NEET	105 (49.1)	77 (38.3)	1.56 (1.05 to 2.30), 0.027		1.56 (1.05 to 2.30), 0.027	
**Deprivation quintile**						
1 – least deprived	63 (53.9)	39 (42.9)	1.65 (0.90 to 3.05), 0.11	0.89	1.83 (0.95 to 3.51), 0.07	0.86
2	48 (46.2)	46 (44.7)	1.00 (0.52 to 1.95), 0.99		1.04 (0.53 to 2.03), 0.92	
3	46 (50.6)	49 (46.7)	1.20 (0.67 to 2.15), 0.53		1.22 (0.66 to 2.26), 0.53	
4	40 (45.5)	35 (38.5)	1.28 (0.70 to 2.35), 0.42		1.28 (0.69 to 2.35), 0.43	
5 – most deprived	42 (44.2)	39 (41.1)	1.16 (0.64 to 2.08), 0.629		1.17 (0.64 to 2.14), 0.602	

For peer review only - http://bmjopen.bmj.com/site/about/guidelines.xhtml

[a] FNP compared to usual care. Analysis adjusted for stratification (site), minimisation variables (gestational age, smoking status at recruitment, and first or preferred language); [b] Analysis additionally adjusted for month of birth. N/EET – Not/In Education, Employment or Training (Source: National Pupil Database, The Department for Education).

For peer review only

Domestic abuse and CIN status:

Domestic abuse experienced by the mother was assessed using the Composite Abuse Scale (CAS) in the BB:0-2 trial at 24 months follow-up (with a score 1 or greater indicating some sort of abuse). The CAS could only be administered if the interview was face-to-face (as opposed to by telephone) and only if the participant was alone (N=607). In the BB:2-6 sample, this could be examined in 595 participants (FNP:322, Usual Care:273) and the proportion of participants experiencing some form of abuse was comparable to that found in the BB:0-2 trial (FNP: 122 (27.9%) vs UC:113 (41.4%)). There was also a relationship found between CAS and CIN status with 49 (20.9%) participants who experienced some abuse having a child being in need versus 42 (11.8%) of those who did not experience abuse. Domestic abuse at 24 months was to be explored as a possible effect mediator but due to lack of intervention effect in the child's CIN status, the need to examine the proportion of effect mediated by experience of domestic abuse at 24 months was redundant.

Care-experienced mothers and CIN status:

In addition, care-experienced mothers at time of recruitment to the BB:0-2 trial was examined. This was defined from the CLA dataset. Sixty-one mothers were identified as having experienced a period of care, four of whom were identified as care-experienced only after their BB:0-2 recruitment date. Fifty-seven mothers were examined for their duration of care and the women randomised to receive FNP experienced on average nine months more care than the Usual care arm (median (25th to 75th centile) FNP: 40 (15.5 to 113.0) UC: 31 (7.0 to 69.5) months). After merging these care-experienced mothers into the children's dataset, 56 mothers remained; seven mothers were excluded from the subgroup analyses since their child did not have any NPD data. Children of these care-experienced mothers were at a higher risk of being defined as in need themselves but no interaction was observed between trial arm and mothers care status. The duration of the maternal care status as a possible mediator effect was also stated in the statistical analysis plan but similarly to domestic abuse, due to lack of intervention effect in child's CIN status, then the need to examine the proportion of effect mediated by length of mother's care experience was redundant.

VIII. Treatment efficacy

The analyses of the following outcomes were re-run to investigate the effect of FNP dosage (number of visits over all delivery phases) using complier averaged causal effects modelling by fitting a structural mean model. The programme stated maximum number of visits over the whole period of FNP is 64 (14 in the pregnancy phase, 28 in the infancy phase, 22 in the toddler phase).

For peer review only - http://bmjopen.bmj.com/site/about/guidelines.xhtml

Referrals to CSC services: Adjusting the analysis for receipt of full dosage of FNP visits, made no difference to the conclusion of the main analysis for referral to social services (efficacy per visit odds ratio (OR)=1.00, 95% CI=0.99 to 1.004, p=0.613).

CIN status: Adjusting the analysis for receipt of FNP made no difference to the conclusion of the primary outcome (efficacy per visit OR=1.00, 95% CI=0.99 to 1.004, p=0.613).

Early Years assessment total point score: Adjusting the analysis for receipt of FNP we observe a 0.02 increase in point score in the between-arm mean difference in early years total score (as the number of visits increase so does the score) however the confidence interval around this estimate is wide (-0.004 to 0.040). The estimated treatment efficacy for participants receiving all 64 visits was 1.15 (95% CI -0.26 to 2.56), p=0.106 showing no change to the overall conclusions compared to the primary analysis.

KS1 attainment: Adjusting the analysis for FNP receipt we observe an increase in the odds of an event occurring in the FNP arm compared UC arm for every unit increase in FNP visit received. For all areas of learning (reading, writing, maths and science), adjusting the analysis for FNP receipt strengthens the effect of FNP, but it is the reading and writing assessment where given the full dosage of 64 FNP visits the odds make a significant difference. For reading assessments, the odds of reaching the expected standard increases from OR=1.23 (95% CI=0.99 to 1.53), p=0.051 in the main analysis to 1.38 (1.07 to 1.77), p=0.015. The efficacy per visit for reading was 1.005 (1.001 to 1.009), p=0.015; maths: 1.001 (0.997 to 1.005), p=0.535; science: 1.004 (0.999 to 1.008), p=0.112; writing: 1.013 (1.09 to 1.017), p=0.001.

IX. Missing data

Since only a small number (n=40 (2.7%)) of children were not linked to any NPD dataset and not included in the primary outcome analyses, no multiple imputation was carried out.

For peer review only

X. Cost Consequence Analysis

Table S15 Cost-consequence balance sheet for the family-nurse based intervention for the four years between children's ages 2 and 6.

**Mothers**						
	Mean resource use per participant			Mean cost per participant (£)		
	FNP	Usual Care	Incremental (FNP-Usual Care)	FNP	Usual Care	Incremental (FNP-Usual Care)
**Health related resource use**						
Inpatient Attendances						
Overnight admissions (N)	3.04	2.99	0.05			
Overnight length of stay (N nights)	2.32	2.32	0.00	2,826.79	2,844.58	-17.79
Day Admittances	2.93	2.91	0.02	1,874.61	1,890.11	-15.50
Outpatient Attendances						
All Outpatient Attendances	23.29	22.61	0.68	2,244.62	2,189.32	55.30
Accident and Emergency						
A&E Visits (N)	5.19	4.93	0.26	411.34	392.03	19.31
Total Resource Use						
				7,357.36	7,316.04	41.32 p=0.393
**Children**						
	Mean resource use per participant			Mean cost per participant (£)		
	FNP	Usual Care	Incremental (FNP-Usual Care)	FNP	Usual Care	Incremental (FNP-Usual Care)
**Health related resource use**						
Inpatient Attendances - All						
Overnight admissions (N)	1.57	1.55	0.02			
Overnight length of stay (N nights)	3.57	3.39	0.18	1,962.95	2,033.71	-70.76
Day Admittances	0.98	0.97	0.01	744.94	724.90	20.04
Inpatient Attendances – All (Excluding Injuries and Ingestions)						
Overnight admissions (N)	1.51	1.49	0.02	1,883.44	1,951.15	-67.71
Overnight length of stay (N nights)	3.61	3.44	0.17			
Day Admittances	0.90	0.88	0.02	689.22	667.09	22.13
Inpatient Attendances – Injuries and Ingestions Only						
Overnight admissions (N)	0.06	0.06	0.00	79.51	82.55	-3.04

For peer review only - http://bmjopen.bmj.com/site/about/guidelines.xhtml

For peer review only

Overnight length of stay (N nights)	2.50	2.05	0.45			
Day Admittances	0.08	0.09	-0.01	55.72	57.81	-2.09
Outpatient Attendances						
All Outpatient Attendances	6.89	7.44	-0.55	673.24	727.79	-54.55
Accident and Emergency - All						
A&E Visits (N)	4.93	4.62	0.31	395.73	366.83	28.90
Accident and Emergency - All (Excluding Injuries and Ingestions)						
A&E Visits (N)	3.80	3.52	0.28	305.12	280.20	24.92
Accident and Emergency – Injuries and Ingestions Only						
A&E Visits (N)	1.12	1.10	0.02	75.09	69.91	5.18
Total Resource Use						
				3,778.13	3,853.22	-75.09 p=0.865
**Consequences**						
Child in Need status recorded at any time during the follow-up period.						OR: 0.98 (95%CI: 0.74 to 1.31) p=0.902
Referral to Social Services						OR: 0.97 (0.74 to 1.28) p=0.829
Child Protection registration (CPP)						OR: 1.04 (0.69 to 1.57) p=0.846
Details of a CPP						
• Neglect						Reference
• Emotional						OR: 2.77 (1.02 to 7.56) p=0.046
• Physical						OR: 1.25 (0.32 to 4.88) p=0.743
• Sexual and multiple						OR: 1.13 (0.33 to 3.86) p=0.848
CIN categorisation						
• Abuse or Neglect						Reference
• Family dysfunction						OR: 0.95 (0.54 to 1.69) p=0.868
• Family in acute stress						OR: 1.47 (0.61 to 3.54) p=0.389
• Low income, absent parenting, socially unacceptable behaviour						OR: 2.38 (0.86 to 6.57) p=0.093
• Child/parent disability						OR: 1.47 (0.45 to 4.84) p=0.525
Looked after status						OR: 0.90 (0.52 to 1.57) p=0.712
Child looked after period of care (months)						OR: 0.75 (0.65 to 0.86) p<0.001
DNA appointments						
• DNA at least once						OR: 1.00 (0.79 to 1.26) p=0.997
Injuries and ingestions						
• Emergency attendance						
○ No						Reference
○ Yes						OR: 1.17 (0.95 to 1.45) p=0.149
• Admission to hospital						

For peer review only

For peer review only - http://bmjopen.bmj.com/site/about/guidelines.xhtml

○ No	Reference
○ Yes	OR: 0.87 (0.63 to 1.20) p=0.407
• Either hospital attendance and/or admission to hospital	
○ No	Reference
○ Yes	OR: 1.11 (0.89 to 1.37) p=0.351
Special Educational Needs	OR: 0.83 (0.67 to 1.03) p=0.097
Early Years assessment	
• Achieving good level of development in all five areas of learning	OR: 1.26 (1.03 to 1.55) p=0.026
• Achieving good level of development in all 17 early learning goals	OR: 1.24 (1.01 to 1.52) p=0.043
School attendance	
• Overall absences	
○ No absences	Reference
○ At least one absence	OR: 1.00 (0.47 to 2.12) p=0.998
• Overall unauthorised absences	
○ No absences	Reference
○ At least one absence	OR: 0.95 (0.76 to 1.18) p=0.620
• Overall authorised absences	
○ No absences	Reference
○ At least one absence	OR: 1.01 (0.58 to 1.75) p=0.984
KS1 attainment	
• Reading	
○ Lower than expected	Reference
○ Reaching the expected standard	OR: 1.23 (0.99 to 1.53) p=0.051
• Writing academic year 2016/17	
○ Lower than expected	Reference
○ Reaching the expected standard	OR: 1.24 (0.97 to 1.60) p=0.090
• Maths	
○ Lower than expected	Reference
○ Reaching the expected standard	OR: 1.04 (0.84 to 1.28) p=0.731
• Science	
○ Lower than expected	Reference
○ Reaching the expected standard	OR: 1.14 (0.91 to 1.43) p=0.254

For peer review only

For peer review only - http://bmjopen.bmj.com/site/about/guidelines.xhtml

The RECORD statement – checklist of items, extended from the STROBE statement, that should be reported in observational studies using routinely collected health data.

	Item No.	STROBE items	Location in manuscript where items are reported	RECORD items	Location in manuscript where items are reported
**Title and abstract**					
	1	(a) Indicate the study's design with a commonly used term in the title or the abstract (b) Provide in the abstract an informative and balanced summary of what was done and what was found	1a/b Abstract	RECORD 1.1: The type of data used should be specified in the title or abstract. When possible, the name of the databases used should be included.<br><br>RECORD 1.2: If applicable, the geographic region and timeframe within which the study took place should be reported in the title or abstract.<br><br>RECORD 1.3: If linkage between databases was conducted for the study, this should be clearly stated in the title or abstract.	1.1 Abstract<br>1.2 Abstract<br>1.3 Abstract
**Introduction**					
Background rationale	2	Explain the scientific background and rationale for the investigation being reported	Introduction		
Objectives	3	State specific objectives, including any prespecified hypotheses	Introduction > Objectives		
**Methods**					
Study Design	4	Present key elements of study design early in the paper	Methods> Study design, setting and participants		
Setting	5	Describe the setting, locations, and relevant dates, including periods of recruitment, exposure, follow-up, and data collection	Methods>Procedure and data sources		

For peer review only

Participants	6	*(a) Cohort study* - Give the eligibility criteria, and the sources and methods of selection of participants. Describe methods of follow-up<br>*Case-control study* - Give the eligibility criteria, and the sources and methods of case ascertainment and control selection. Give the rationale for the choice of cases and controls<br>*Cross-sectional study* - Give the eligibility criteria, and the sources and methods of selection of participants<br><br>*(b) Cohort study* - For matched studies, give matching criteria and number of exposed and unexposed<br>*Case-control study* - For matched studies, give matching criteria and the number of controls per case	6a Methods> Study design, setting and participants<br><br>6b NA	RECORD 6.1: The methods of study population selection (such as codes or algorithms used to identify subjects) should be listed in detail. If this is not possible, an explanation should be provided.<br><br>RECORD 6.2: Any validation studies of the codes or algorithms used to select the population should be referenced. If validation was conducted for this study and not published elsewhere, detailed methods and results should be provided.<br><br>RECORD 6.3: If the study involved linkage of databases, consider use of a flow diagram or other graphical display to demonstrate the data linkage process, including the number of individuals with linked data at each stage.	6.1 Methods> Study design, setting and participants<br><br>6.2 NA<br><br>6.3 Protocol paper cited, Figure 1
Variables	7	Clearly define all outcomes, exposures, predictors, potential confounders, and effect modifiers. Give diagnostic criteria, if applicable.	Methods> Study design, setting and participants, Outcomes; Appendix	RECORD 7.1: A complete list of codes and algorithms used to classify exposures, outcomes, confounders, and effect modifiers should be provided. If these cannot be reported, an explanation should be provided.	Methods> Study design, setting and participants, Outcomes
Data sources/ measurement	8	For each variable of interest, give sources of data and details of methods of assessment (measurement).<br>Describe comparability of assessment methods if there is more than one group	Methods> Study design, setting and participants, Outcomes; Appendix		

For peer review only

Bias	9	Describe any efforts to address potential sources of bias	Methods> Efforts to address sources of bias		
Study size	10	Explain how the study size was arrived at	Methods> Power calculation		
Quantitative variables	11	Explain how quantitative variables were handled in the analyses. If applicable, describe which groupings were chosen, and why	Methods>Statistical analysis		
Statistical methods	12	(a) Describe all statistical methods, including those used to control for confounding<br>(b) Describe any methods used to examine subgroups and interactions<br>(c) Explain how missing data were addressed<br>(d) *Cohort study* - If applicable, explain how loss to follow-up was addressed<br>*Case-control study* - If applicable, explain how matching of cases and controls was addressed<br>*Cross-sectional study* - If applicable, describe analytical methods taking account of sampling strategy<br>(e) Describe any sensitivity analyses	12a-c, e: Methods>Statistical analysis; Economic analysis<br>12d: NA		
Data access and cleaning methods		..		RECORD 12.1: Authors should describe the extent to which the investigators had access to the database population used to create the study population.	Methods> Study design, setting and participants, Outcomes; Appendix

For peer review only

				RECORD 12.2: Authors should provide information on the data cleaning methods used in the study.	
Linkage		..		RECORD 12.3: State whether the study included person-level, institutional-level, or other data linkage across two or more databases. The methods of linkage and methods of linkage quality evaluation should be provided.	Methods> Procedure and data sources
**Results**					
Participants	13	(a) Report the numbers of individuals at each stage of the study (*e.g.*, numbers potentially eligible, examined for eligibility, confirmed eligible, included in the study, completing follow-up, and analysed)<br>(b) Give reasons for non-participation at each stage.<br>(c) Consider use of a flow diagram	Table S3, Figure 1	RECORD 13.1: Describe in detail the selection of the persons included in the study (*i.e.,* study population selection) including filtering based on data quality, data availability and linkage. The selection of included persons can be described in the text and/or by means of the study flow diagram.	Figure 1
Descriptive data	14	(a) Give characteristics of study participants (*e.g.*, demographic, clinical, social) and information on exposures and potential confounders<br>(b) Indicate the number of participants with missing data for each variable of interest<br>(c) *Cohort study* - summarise follow-up time (*e.g.*, average and total amount)	14a: Table 1<br>14b: Table S3, Tables 2-4		
Outcome data	15	*Cohort study* - Report numbers of outcome events or summary measures over time<br>*Case-control study* - Report numbers in each exposure	Tables 2-4		

For peer review only

		category, or summary measures of exposure<br>*Cross-sectional study* - Report numbers of outcome events or summary measures			
Main results	16	(a) Give unadjusted estimates and, if applicable, confounder-adjusted estimates and their precision (e.g., 95% confidence interval). Make clear which confounders were adjusted for and why they were included<br>(b) Report category boundaries when continuous variables were categorized<br>(c) If relevant, consider translating estimates of relative risk into absolute risk for a meaningful time period	Tables 2-4		
Other analyses	17	Report other analyses done—e.g., analyses of subgroups and interactions, and sensitivity analyses	Appendix		
**Discussion**					
Key results	18	Summarise key results with reference to study objectives	Discussion> Principal findings		
Limitations	19	Discuss limitations of the study, taking into account sources of potential bias or imprecision. Discuss both direction and magnitude of any potential bias		RECORD 19.1: Discuss the implications of using data that were not created or collected to answer the specific research question(s). Include discussion of misclassification bias, unmeasured confounding, missing data, and changing eligibility over time, as they pertain to the study being reported.	Discussion> Strengths and weaknesses of this study, Strengths and weaknesses in comparison to other studies
Interpretation	20	Give a cautious overall interpretation of results considering objectives,			Discussion> Strengths and weaknesses of this study, Strengths

For peer review only

		limitations, multiplicity of analyses, results from similar studies, and other relevant evidence			and weaknesses in comparison to other studies
Generalisability	21	Discuss the generalisability (external validity) of the study results			Discussion> Strengths and weaknesses in comparison to other studies
**Other Information**					
Funding	22	Give the source of funding and the role of the funders for the present study and, if applicable, for the original study on which the present article is based			Described in Funding section
Accessibility of protocol, raw data, and programming code		..		RECORD 22.1: Authors should provide information on how to access any supplemental information such as the study protocol, raw data, or programming code.	Protocol paper cited in references; access to data described in Data sharing statement

*Reference: Benchimol EI, Smeeth L, Guttmann A, Harron K, Moher D, Petersen I, Sørensen HT, von Elm E, Langan SM, the RECORD Working Committee. The REporting of studies Conducted using Observational Routinely-collected health Data (RECORD) Statement. *PLoS Medicine* 2015; in press.

*Checklist is protected under Creative Commons Attribution (CC BY) license.

For peer review only

BMJ Open

A nurse-led home-visitation programme for first-time mothers in reducing maltreatment and improving child health and development (BB:2-6): longer-term outcomes from a randomised cohort using data-linkage.

Journal:	*BMJ Open*
Manuscript ID	bmjopen-2021-049960.R2
Article Type:	Original research
Date Submitted by the Author:	08-Aug-2021
Complete List of Authors:	Robling, Michael; Cardiff University, Centre for Trials Research; DECIPHer, Cardiff University School of Social Sciences<br>Lugg-Widger, Fiona; Cardiff University, Centre for Trials Research<br>Cannings-John, Rebecca; Cardiff University Centre for Trials Research,<br>Angel, Lianna; Cardiff University, Centre for Trials Research<br>Channon, Sue; Cardiff University, Centre for Trials Research<br>Fitzsimmons, Deborah; Swansea University, College of Human and Health Sciences<br>Hood, Kerenza; Cardiff University,<br>Kenkre, Joyce; University of South Wales, Faculty of Life Sciences and Education<br>Moody, Gwenllian; Cardiff University, Centre for Trials Research<br>Owen-Jones, Eleri; Cardiff University, Centre for Trials Research<br>Pockett, Rhys; Swansea University, Swansea Centre for Health Economics<br>Sanders, Julia; Cardiff University, School of Healthcare Sciences<br>Segrott, Jeremy; Cardiff University, Centre for Trials Research; Cardiff University, DECIPHer Centre, Cardiff School of Social Sciences<br>Slater, Thomas; School of Social Sciences
**Primary Subject Heading**:	Public health
Secondary Subject Heading:	Nursing
Keywords:	PUBLIC HEALTH, Child protection < PAEDIATRICS, PREVENTIVE MEDICINE

SCHOLARONE™
Manuscripts

I, the Submitting Author has the right to grant and does grant on behalf of all authors of the Work (as defined in the below author licence), an exclusive licence and/or a non-exclusive licence for contributions from authors who are: i) UK Crown employees; ii) where BMJ has agreed a CC-BY licence shall apply, and/or iii) in accordance with the terms applicable for US Federal Government officers or employees acting as part of their official duties; on a worldwide, perpetual, irrevocable, royalty-free basis to BMJ Publishing Group Ltd ("BMJ") its licensees and where the relevant Journal is co-owned by BMJ to the co-owners of the Journal, to publish the Work in this journal and any other BMJ products and to exploit all rights, as set out in our licence.

The Submitting Author accepts and understands that any supply made under these terms is made by BMJ to the Submitting Author unless you are acting as an employee on behalf of your employer or a postgraduate student of an affiliated institution which is paying any applicable article publishing charge ("APC") for Open Access articles. Where the Submitting Author wishes to make the Work available on an Open Access basis (and intends to pay the relevant APC), the terms of reuse of such Open Access shall be governed by a Creative Commons licence – details of these licences and which Creative Commons licence will apply to this Work are set out in our licence referred to above.

Other than as permitted in any relevant BMJ Author's Self Archiving Policies, I confirm this Work has not been accepted for publication elsewhere, is not being considered for publication elsewhere and does not duplicate material already published. I confirm all authors consent to publication of this Work and authorise the granting of this licence.

For peer review only

Title: A nurse-led home-visitation programme for first-time mothers in reducing maltreatment and improving child health and development (BB:2-6): longer-term outcomes from a randomised cohort using data-linkage

Authorship:

Michael Robling, [1,2] (Director of Population Health Trials), RoblingMR@cardiff.ac.uk
Fiona Lugg-Widger, [1] (Research Fellow), LuggFV@cardiff.ac.uk
Rebecca Cannings-John, [1] (Senior Research Fellow), CanningsRL@cardiff.ac.uk
Lianna Angel, [2] (Research Associate), AngelL@cardiff.ac.uk
Susan Channon, [1] (Director Research Design and Conduct Service) ChannonS2@cardiff.ac.uk
Deborah Fitzsimmons, [3] (Personal Chair, Public Health, Policy and Social Sciences) d.fitzsimmons@swansea.ac.uk
Kerenza Hood, [1] (Director of the Centre for Trials Research) HoodK1@cardiff.ac.uk
Joyce Kenkre, [4] (Professor of Primary Care) joyce.kenkre@southwales.ac.uk
Gwenllian Moody, [1] (Research Associate) MoodyG@cardiff.ac.uk
Eleri Owen-Jones, [1] (Research Associate) Owen-JonesCE@cardiff.ac.uk
Rhys Pockett, [3] (Senior Lecturer) r.d.pockett@swansea.ac.uk
Julia Sanders, [5] (Professor of Clinical Nursing and Midwifery) SandersJ3@cardiff.ac.uk
Jeremy Segrott, [1,2] (Senior lecturer) SegrottJ@cardiff.ac.uk
Thomas Slater, [6] (Senior lecturer) slaterTB1@cardiff.ac.uk

Affiliations:

1. Centre for Trials Research, Cardiff University, Cardiff, UK
2. DECIPHer, Cardiff University, UK
3. Swansea Centre for Health Economics, Swansea University, UK
4. Faculty of Life Sciences and Education, University of South Wales, UK
5. School of Healthcare Sciences, Cardiff University, UK
6. School of Social Sciences, Cardiff University, UK

Corresponding author:

Michael Robling, Director of Population Health Trials, RoblingMR@cardiff.ac.uk, Centre for Trials Research, College of Biomedical & Life Sciences, Cardiff University, 7th Floor, Neuadd Meirionnydd, Heath Park, Cardiff. CF14 4YS

Keywords: Home-visiting, maltreatment, child development, routine data, cohort

For peer review only

Abstract (300 words)

Objectives Measure effectiveness of Family Nurse Partnership (FNP) home-visiting programme in reducing maltreatment and improving maternal health and child health, developmental and educational outcomes; explore effect moderators, mediators; describe costs.

Design Follow-up of BB:0-2 trial cohort (ISRCTN:23019866) up to age 7 years in England using record linkage.

Participants 1,618 mothers aged 19 years or younger and their firstborn child(ren) recruited to BB:0-2 trial at less than 25 weeks gestation and not mandatorily withdrawn from trial or opted out. Intervention families were offered up to a maximum of 64 home visits by specially trained nurses from pregnancy until firstborn child was 2 years old, plus usually provided health and social care support. Comparator was usual care alone.

Outcome measures Primary outcome: state-verified child-in-need status recorded at any time during follow-up. Secondary outcomes: referral to social services, child protection registration (plan), child-in-need categorisation, looked-after status, recorded injuries and ingestions any time during follow-up, early childcare and educational attendance, school readiness and attainment at Key Stage 1 (KS1), healthcare costs.

Results Match rates for 1,547 eligible children (1517 singletons, 15 sets of twins) were 98.3% (NHS Digital) and 97.4% (National Pupil Database). There was no difference between study arms in the proportion of children being registered as in need (adjusted OR=0.98, 95% CI=0.74,1.31), or for any other measure of maltreatment. Children in the FNP arm were more likely to achieve a good level of development at reception age (school readiness) (adjusted OR=1.24, 95% CI= 1.01,1.52). After adjusting for birth month, children in FNP arm were more likely to reach the expected standard in reading at KS1 (adjusted OR=1.26, 95% CI=1.02,1.57). We found no trial arm differences for resource use and costs.

Conclusions FNP did not improve maltreatment or maternal outcomes. There was evidence of small advantages in school readiness and attainment at KS1.

Strengths and Limitations

- We used administrative records and novel data linkage to provide longer-term follow-up for a well characterised trial cohort
- This has maximised cohort retention and minimised bias
- We used data from a range of sectors including health, social care and education to assess the broad impact of the specialist home visiting programme

For peer review only

Manuscript (Word count is 5773)

Introduction

Protecting children from maltreatment and promoting their healthy development are UK policy priorities. The Family Nurse Partnership (FNP) is a preventative home-visiting programme for young women expecting their first child. FNP was developed in the USA as the Nurse Family Partnership (NFP) and subsequently adapted for implementation in England in 2007.

Three US trials have demonstrated programme improvements in prenatal health behaviours and birth outcomes, sensitive childcare, maternal life-course and child functioning.[1–3] A subgroup analysis of poor unmarried teenage mothers in the first US trial (n=54) found verified maltreatment by age 2 years in 19% of 32 control children and 4% of the 22 children in the group in receipt of NFP during both pregnancy and infancy (mean % difference: 0.15, 95% confidence interval (CI) of −0.01 to 0.31).[1] Additionally, there was a 56% relative reduction in emergency department encounters for injuries and ingestions during the second year of life. For children with state-verified maltreatment reported by age 4, children in the NFP group had fewer risks for harm than the control group between 25 and 50 months of life. A trial of VoorZorg, the Dutch adaptation of NFP, found a reduction in child-protecting agency reports by age three years for families in the home-visiting arm.[4,]

We evaluated short-term FNP programme outcomes to age 2 years in our Building Blocks trial (BB:0-2).[5] We found no difference for four primary outcomes: maternal tobacco use in late pregnancy, birth weight of the baby, proportion of women with a second pregnancy within 24 months postpartum and emergency attendances and hospital admissions for the child within 24 months postpartum. There were some differences in favour of FNP for secondary child development outcomes including maternal-reported cognitive function and language at 24 months.[5] As the previous US trials showed benefit for maltreatment outcomes increasingly after age four we sought to establish whether FNP moderates maltreatment outcomes over a medium-term period. Through access to administrative data, we aimed to determine impact across a range of child maltreatment outcomes and key indicators of neglect (e.g. injuries and ingestions). We also sought to assess impacts on other programme relevant developmental and maternal outcomes as well as costs.

Objectives

Primary: to determine programme effectiveness in reducing maltreatment, when compared with usually provided health and social care alone. Secondary: to determine programme effectiveness in reducing maltreatment, medium-term programme outcomes such as subsequent pregnancies, school

For peer review only - http://bmjopen.bmj.com/site/about/guidelines.xhtml

readiness and educational outcomes, the impact of moderators of programme effect and the cost and consequences of the programme.

Methods

Study design, setting and participants

This study followed a cohort of mothers and children who had participated in the BB:0-2 trial for a further five years using administrative data only.[6,7] Data were retrieved from public sector providers and linked to the trial dataset using opt-out consent, following REC approval (14/WA10062) and s251 support (CAG 10-08(b)/2014). Children and mothers were followed up until the child reached Key Stage 1 teacher-based assessment (KS1) by which time most children were seven years old.

Building Blocks: 2-6 (BB:2-6) study participants were women and their first child (or twins, if relevant) who were not mandatorily withdrawn from the BB:0-2 trial; did not electively withdraw and/or did not opt-out of this follow-on study.[5-7] BB:0-2 trial participants were eligible if women were nulliparous, aged 19 years or younger, were recruited at less than 25 weeks' gestation, living within the catchment area of a local FNP team, able to provide consent and speak English. Women expecting multiple births and those with a previous pregnancy ending in miscarriage, stillbirth, or termination were eligible. Women planning to have their child adopted or to move beyond the FNP catchment area for more than 3 months were not eligible. [5-8] Trial participants had been randomly assigned to FNP or usual care, with randomisation stratified by site and minimised by gestation (<16 weeks vs ≥16 weeks), smoking (yes vs no), and preferred language of data collection (English vs non- English) and weighted towards minimising the imbalance in trial groups with probability 0.8.

Intervention and comparator (BB:0-2 Trial)

The FNP programme provides up to a maximum of 64 home visits from specially trained family nurses during pregnancy until the child is two years old addressing personal and environmental health, life course development, maternal role, family and friends and access to health and social services. For the 697 women randomised and enrolled to FNP in BB:0-2 and then retained in the BB:2-6 cohort, the average number (SD) of valid visits received by FNP clients by programme phase was: Pregnancy 9.74 (3.42), Infancy 18.7 (5.97) and Toddlerhood 13.28 (5.46) (Table S1). In the BB:0-2 trial, mean (SD) visit duration varied by delivery phase (Pregnancy: 79.14 mins (13.78), Infancy: 73.17 (11.61), Toddlerhood: 74.75 (13.50) which exceeded the programme target of 60 minutes.[7] All trial participants received usually provided health and social care services. Participants in the usual care (UC) arm received these services alone.[5,7-9] The number (SD) of midwifery contacts were recorded in the BB:0-2 trial and were for FNP clients 10.68 (5.25) and for women in UC arm 10.69 (5.34).[9] The number (SD) of home visits from specialist public health nurses (SPHN) reported by trial participants by 18 months postpartum was for FNP clients 4.70 (7.81) and for women in the UC arm

For peer review only - http://bmjopen.bmj.com/site/about/guidelines.xhtml

5.01 (5.5). The average number (SD) of clinic contacts with SPHNs was 0.70 (2.29) for women in the FNP arm and 6.31 (7.07) for women in the control arm.

Procedure and data sources

The BB:0-2 trial consented 1618 eligible women between June 2009 and July 2010 and we concluded all BB:2-6 follow-up in May 2018. Maternal and child identifiers were sent to NHS Digital and the Department for Education (DfE) for matching with their databases. Maternal identifiers were also sent to the Department of Health and Social Care (DHSC) for matching with abortion statistics. Matching with NHS Digital and DHSC used a combination of NHS number, date of birth, postcode and sex. NHS Digital also provided a linked Hospital Episode Statistics (HES) - Office for National Statistics (ONS) extract for mortality data. Matching with the DfE used exact matching on first name and surname, date of birth and postcode (of both mother and child for social care data; all other datasets, just child). All matched data were sent to a third-party data safe haven (Secure Anonymised Information Linkage (SAIL) Databank) and linked by project identifiers to trial data and analysed via remote access.[10-12]

Outcomes

The primary outcome was Child in Need (CIN) status recorded at any time by a local department of children's social care services (CSCS) (and sourced from the NPD). This includes children assessed as unlikely to achieve or maintain a reasonable level of health or development, or whose health and development is likely to be significantly or further impaired, without the provision of services; or is a child who is disabled. Secondary outcomes were additional formally reported measures of maltreatment (referral to CSCS, child being on a Child Protection Plan, CIN categorisation, Looked after status (mother, child)); Associated measures of maltreatment (recorded injuries and ingestions, non-attendance rates for hospital appointments); Maternal outcomes (subsequent pregnancies); Child health, developmental and educational outcomes (special educational needs, early educational attendance and assessments (Early Years Foundation Stage Profile (EYFSP), KS1)); and health resource use. Study assessment domains, outcomes and principal data providers and analysis are described in the appendix (Table S2).

For peer review only - http://bmjopen.bmj.com/site/about/guidelines.xhtml

Power calculation

Our sample size was fixed by the cohort available from BB:0-2. However, for CIN status, while UK data on rates are not specific to the age-range of interest, the rate per 10,000 general population aged 5-9 years is 4.6% for local authorities covering BB:0-2 trial sites. [13] The rate of CIN status would be expected to be greater in the specific study sample. Therefore, we assumed a rate of 8%, hypothesised that FNP would reduce the presence of CIN in the first six years and assumed a difference of 4% would be important. To detect a difference of 4% (4% vs 8%) would require 602 children in each arm with 80% power and a two-sided 5% alpha level. With 1562 children available for follow-up (i.e. excluding mandatory withdrawals) and follow up through administrative records with 10% loss in tracking and linkage we would have 1405 participants, sufficient data to assess the primary outcome.

Efforts to address sources of bias

All maltreatment and child developmental outcomes were independently state-verified (social worker) reports or teacher-assessments respectively, recorded in administrative service data and abstracted from national data providers. By using administrative data and established linking fields we were able to minimise bias due to loss to follow-up. An a priori statistical analysis plan described primary, secondary (including sub-group) and sensitivity analyses, and any additional exploratory analysis is clearly highlighted.

Statistical analysis

Analysis was conducted on participants whose identifiers could be sent, linked and data released by the data providers. This excluded participants whose individual data could be used but could not be matched due to incorrect linking fields, other exclusions from health or education: e.g. private or home schooling and National opt-outs. Imbalance between the BB:0-2 and eligible BB:2-6 participants was quantified using descriptive statistics in baseline demographics, clinical and questionnaire data. We conducted all analyses on an intention-to-treat (ITT) basis with complete case population (with follow-up data). Therefore, participants remained in the BB:0-2 trial groups they were randomised to regardless of the intervention received. We used multilevel modelling to allow for clustering of effect within the 18 sites and family nurse level and both fitted as random effects. Where clustering was minimal at the family nurse level, results from the two-level model are presented. Parameter estimates are reported alongside a 95% CI and p-value. We adjusted for variables used in randomisation (smoking status, gestational age, language). We defined loss to follow up in this study as a child death or adoption, both of which we were able to determine. We excluded children from analyses of binary outcomes where follow-up was incomplete and no event was observed. With time-to-event analyses, children were censored at these events.

For peer review only - http://bmjopen.bmj.com/site/about/guidelines.xhtml

Primary comparative analysis examined whether the firstborn child(ren) ('BB:0-2') had ever been referred to CSCS and classed as a child in need at any point between birth and 6 years of age (appendix). For this binary outcome we used logistic multilevel modelling to investigate differences in the proportion defined as in need between the trial arms. We present the resulting estimate as an odds ratio. For binary and categorical outcomes, comparative analysis used logistic and multinomial modelling respectively with results presented as odds ratios (OR). For continuous outcomes we used linear regression and present mean differences. For count data we used Poisson regression modelling; where event distribution displayed signs of over dispersion a Negative Binomial regression model was used (or zero-inflated model if appropriate). Results are presented as incidence rate ratios (IRR). Time to event data used Cox regression modelling presented with hazard ratios (HR). To reduce problems with multiple comparisons, some outcomes were only assessed descriptively (for example, we prioritised and tested overall scores for a Good Level of Development in the Early Years Profile, rather than the individual areas of learning). Similarly, for outcomes that we expected to reflect a small number of events or higher levels of missingness we also took a descriptive approach. Nevertheless, we present all a priori comparative analyses in the main tables and all other descriptive and exploratory analyses in the supplementary materials.

KS1 data were received in two tranches from NPD, firstly children assessed in 2016/17 academic year (received in May 2018) and then younger children who were assessed in 2017/18 (received May 2019). Reading, maths and science were examined for the whole cohort over both academic years. However, following changes made within the 2017/18 writing assessment (such that judgements in 2018 are not directly comparable to those made using the previous interim frameworks in 2016/2017), writing was only statistically assessed using 2016/17 data and the data from 2017/18 were presented descriptively. Sensitivity analyses for primary and selected secondary outcomes (EYFSP, KS1, referral to CSCS) included adjusting for hypothesised confounders at baseline, dosage effects (i.e. number of visits) using complier average causal effects (CACE) modelling and sub-group analysis of potential effect moderators and mediators (maternal deprivation, adaptive functioning, NEET (not in education, employment or training) status at recruitment, maternal age at recruitment, child sex, maternal care status, duration of maternal care and domestic abuse self-reported at 24 months) as interaction terms in the main comparative models.

Results of all planned sensitivity analyses are presented in the appendix. An additional post hoc sensitivity analysis adjusted for the child's month of birth (categorised into quarter of birth) in the EYFSP and KS1 outcomes.

For peer review only - http://bmjopen.bmj.com/site/about/guidelines.xhtml

Economic Analysis

A cost consequences analysis of FNP over the full follow-up period (BB:0–2 and BB:2–6) took a secondary health care (UK NHS) perspective. The principal data source was Hospital Episode Statistics (HES) records (inpatient, outpatient, A&E), which were coded in Healthcare Resource Group (HRG) Grouper and matched to appropriate NHS Reference costs. Maternal and child resource use were costed separately and valued in £ sterling. Only direct healthcare costs were analysed. Indirect costs such as lost wages or childcare costs were not available within the routinely collected data. Where data were absent in HES records it was assumed no resource was incurred. No primary care data were available beyond 18 months postpartum. Costs were discounted back from year of event to baseline (2009/10) at an annual rate of 3.5% as described by Central Government's guidance on appraisal and evaluation. [14]

Data management

Small numbers were handled according to SAIL Databank rules where any cell counts under five were suppressed in reporting. Where abortion data are presented, we handled in accordance with the data sharing agreement with DHSC where counts less than 9 were suppressed. Data were analysed using Stata v16 and IBM SPSS v23.0.

Patient and public involvement

Activities involved four groups of young people (CASCADE Voices, Our Place, FNP graduates and ALPHA). These activities contributed to three aspects of the study: data-linkage methods (e.g. to optimise acceptability of the proposed dissent model, use of data linkage and communication of these methods)[15], study outcomes (by seeking views on the importance of the study outcomes) and dissemination of findings (including best way to present results, method of dissemination).

Results

The study population following the BB:0-2 trial tracing and matching process is shown in Figure 1. Five women were ineligible due to not meeting original trial entry criteria and 78 were mandatory withdrawals mainly due to fetal or infant death and adoption.[5] The 110 women who electively withdrew during the trial were given the opportunity to opt-out from further data usage, 16 of whom removed consent for any further contact. Thus 94 women who had electively withdrawn from BB:0-2 were included, and their details sent for tracing alongside the remaining 1,452 mothers (1,546 mother-child dyads in total) to update contact details to improve matching. One dyad was subsequently removed as deceased and the remaining 1,545 contacted by letter, email or text of whom eight dissented from having their records linked. 1,537 mothers and 1,547 children were sent to the DfE and

For peer review only

NHS Digital for matching. These formed the population for the BB:2-6 study. Match rates for children were 98.3% (NHS Digital) and 97.4% (DfE).

Numbers matched to and returned for analysis differed by data source and population (Table S3). Owing to having discontinued their education, few mothers, compared with children, were matched to any DfE data set (19% vs. 97%, respectively); these events were based mainly on the CIN data set. For selected maternal and child baseline variables and delivery of FNP assessed against programme fidelity goals, the BB:2-6 sample appears broadly representative of the original BB:0-2 cohort (Tables S4 and S5). Baseline characteristics of the BB:2-6 mothers and children were well balanced between trial arms (Table 1).

Table 1 Socio-demographic characteristics of mother and babies at baseline by trial arm

	FNP N=766	Usual care N=771
**Maternal age at recruitment (years)**	17·9 (17·0 to 18·8)	17·9 (16·9 to 18·8)
**Ethnicity**		
White background	675 (88·1%)	680 (88·2%)
Mixed background	44 (5·7%)	38 (4·9%)
Asian background	15 (2·0%)	10 (1·3%)
Black background	29 (3·8%)	40 (5·2%)
Chinese or Other background	<5	<5
**Relationship status with baby's father**		
Married	6 (0·8%)	10 (1·3%)
Separated	72 (9·4%)	78 (10·1%)
Closely involved/boyfriend	582 (76·0%)	586 (76·0%)
Just friends	106 (13·8%)	97 (12·6%)
**NEET Status***		
Yes	315/764 (41·1%)	319/769 (41·4%)
No	345/764 (45·0%)	333/769 (43·2%)
**IMD Overall Score†**	38·1 (24·6 to 52·6)‡	38·1 (25·5 to 51·6)§
**Generalised self-efficacy scale (score 10 to 40)¶**	30·0 (28·0 to 33·0)‖	30·0 (27·0 to 32·0)**
**Adaptive functioning:**		
**Difficulty in at least one basic skill**		
Yes	213/765 (27·8%)	184/770 (23·9%)
No	552/765 (72·2%)	586/770 (76·1%)
**Had 3 or less life skills (out of 5)**		

For peer review only - http://bmjopen.bmj.com/site/about/guidelines.xhtml

	FNP N=766	Usual care N=771
Yes	185/763 (24·2%)	218/769 (28·3%)
No	578/763 (75·8%)	551/769 (71·7%)
**At least one burden**		
Yes	214/759 (28·2%)	237/767 (30·9%)
No	545/759 (71·8%)	530/767 (69·1%)
**Cigarette smoking participant self-reported**		
Ever smoked		
Yes	615 (80·3%)	612 (79·4%)
No	151 (19·7%)	159 (20·6%)
**Child characteristics**	**FNP N=773**	**Usual Care N=774**
**Sex**		
Male	381/773 (49·3%)	406/773 (52·5%)
Female	392/773 (50·7%)	367/773 (47·5%)
**Birthweight (grams)** Mean (SD)	3223·81 (606·0)††	3215·52 (555·56)‡‡
**NNU admission (direct or subsequent)**		
Yes	76/716 (10·6%)	66/749 (8·8%)
No	640/716 (89·4%)	683/749 (91·2%)

Data are n (%), n/N (%), or median (25th to 75th centile) unless otherwise stated. <5 = numbers suppressed. FNP = Family Nurse Partnership Programme + Usual Care. IMD = Index of Multiple Deprivation. SD= standard deviation. NNU = Neonatal unit. *Definition of NEET status: Not in education employment or training (applicable only to those whose age at end of previous academic year at time of baseline interview was >16). †Higher IMD score indicated more deprivation. Mean IMD Score for England in 2010 was 21.67 [Wilkinson DL, Sniehotta FF, Michie S. Targeting those in need: Baseline data from the first English National Health Service (NHS) health trainer service: *Psychology, health & medicine* 2011; **16**(6): 736-48], ‡N=760. §N=765. ¶ Higher score indicates higher level of self-efficacy. ‖N=764. **N=769. ††N=724. ‡‡N=752.

For the definition of a child in need referrals resulting in no further action (i.e. not classed as in need) were excluded. Overall, 323 (21.5%) of children in the BB:2-6 cohort had at least one referral to CSCS which resulted as being classed as a child in need at any point between pregnancy (includes pre-birth CIN) and 6 years of age. No difference in rate of children in need was found between FNP (21.2%) and UC (21.7%) arms for the 1,506 children in the analysis (adjusted OR=0.98, 95% CI=0.74, 1.31) (Table 2). The parameter estimate was not affected when adjusting for the number of FNP visits received (efficacy per visit OR=1.00, 95% CI=0.99, 1.004). The number of children referred to CSC and assessed as not requiring additional support were FNP (n=45) and UC (n=46). There were no between arm differences for referral to CSCS, the age at which the first referral was made, the age at which the child was classified as in need and the proportion of children with a protection plan. A small number of children were ever Looked After within the 6-year follow-up

For peer review only - http://bmjopen.bmj.com/site/about/guidelines.xhtml

period, with no evidence of a difference between trial arms (Table 2). However, the UC arm experienced on average two more months in care compared to the FNP arm. The total number of child deaths across the full follow-up period was less than ten and not further reported by study arm, consistent with the non-disclosive data management policy. Data informing the disability outcome were not available for the years requested. In addition, no difference was found when examining age at first referral or CIN by study arm.

No between arm differences were found in children not taken to at least one outpatient appointment (did not attend rate), emergency attendances or hospital admissions for an injury or an ingestion (Table 2). Overall, 78% of mothers had a subsequent pregnancy (using inpatients, outpatients and abortions data) with no differences by arm (Table 3). Similarly, just over two thirds of the cohort had at least one registerable birth (using only admission data as a source) during the follow-up period with a comparable inter-birth interval. Descriptives for non-tested maltreatment outcomes are shown in Table S6. Descriptives for the number of emergency room attendances and hospital admissions for injuries or ingestions are shown for each trial arm in Tables S7-S8. Exploratory analyses of hospital admissions for injuries and ingestions by trial arm shown by age categories (Under 1 year, 1 to 2 years, 25 months to under 6 years) are shown in Table S9.

For peer review only

Table 2 Primary and secondary outcomes: CPS outcomes, healthcare encounters for injuries / ingestions and out-patient non-attendance

Outcome	FNP	Usual care (UC)	Adjusted* parameter estimate (95% CI)	p-value	Absolute risk difference (FNP-UC) (95% CI)
**Child protection outcomes**	**N=760**	**N=746**			
Child in need (CIN) status at any time during the follow-up period					
No	599 (78·8%)	584 (78·3%)	Reference		
Yes	161 (21·2%)	162 (21·7%)	0·98† (0·74 to 1·31)	0·902	-0·5% (-0·5 to 0·4%)
Age at first CIN referral (days)					
Median (25th to 75th centile)	1602·5 (1153.0 to 1978·75)	1649·5 (1130·75 to 1980·25)	0·98‡ (0·79 to 1·23)	0·875	
Missing	*1*	*0*			
Unique§ number of CIN referrals per child	1 (1 to 2)	1 (1 to 2)	0·99‖ (0·82 to 1·19)	0·890	
CIN duration (days)	210 (70·5 to 571·25)	216 (72·75 to 503·25)	1·20‡ (0·90 to 1·59)	0·216	
CIN categorisation (primary need) of first case	***N=161***	***N=162***			
Abuse or neglect	92 (57·1%)	102 (63·0%)	Reference		
Family dysfunction	29 (18·0%)	34 (21·0%)	0·95¶ (0·54 to 1·69)	0·868	
Family in acute stress	13 (8·1%)	10 (6·2%)	1·47¶ (0·61 to 3·54)	0·389	

For peer review only - http://bmjopen.bmj.com/site/about/guidelines.xhtml

Outcome	FNP	Usual care (UC)	Adjusted* parameter estimate (95% CI)	p-value	Absolute risk difference (FNP-UC) (95% CI)
Low income, absent parenting, socially unacceptable behaviour, not stated	13 (8·1%)	6 (3·7%)	2·38‖ (0·86 to 6·57)	0·093	
Child/parent disability	7 (4·3%)	5 (3·1%)	1·47¶ (0·45 to 4·84)	0·525	
Cases other than CIN	7 (4·3%)	5 (3·1%)	1·64¶ (0·50 to 5·37)	0·417	
Referred to Children's social care services (CSCS)					
No	554 (72·9%)	538 (72·1%)	Reference		
Yes	206 (27·1%)	208 (27·9%)	0·97† (0·74 to 1·28)	0·829	-0·8% (-5·3 to 3·7%)
Age at first referral (days)<br>Median (25th to 75th centiles)	1541·5 (1272·75 to 1975·75)	1631·0 (1190·5 to 1984·75)	0·96‡ (0·79 to 1·17)	0·694	
Child Protection Plan					
No	708 (93·2%)	697 (93·4%)	Reference		
Yes	52 (6·8%)	49 (6·6%)	1·04† (0·69 to 1·57)	0·846	0·2% (-0·2 to 0·3%)
Category of child protection plan	***n=52***	***n=49***			
Neglect	17 (32·7%)	22 (44·9%)	Reference		
Emotional	22 (42·3%)	12 (24·5%)	2·77‖ (1·02 to 7·56)	0·046	
Physical	6 (11·5%)	6 (12·2%)	1·25‖ (0·32 to 4·88)	0·743	
Sexual and multiple**	7 (13·5%)	9 (18·4%)	1·13‖ (0·33 to 3·86)	0·848	

For peer review only - http://bmjopen.bmj.com/site/about/guidelines.xhtml

Outcome	FNP	Usual care (UC)	Adjusted* parameter estimate (95% CI)	p-value	Absolute risk difference (FNP-UC) (95% CI)
Looked after status††					
No	735 (96·7%)	719 (96·4%)	Reference		
Yes	25 (3·3%)	27 (3·6%)	0·90† (0·52 to 1·57)	0·712	-0·3% (-0·2 to 0·2%)
Child looked after period of care (months)	10·0 (4·5 to 37·5)	12·0 (6·0 to 33·0)	0·75‖ (0·65 to 0·86)	<0·001	
**Injuries and Ingestions**	**N=760**	**N=759**			
Emergency attendance‡‡					
None	317 (41·7%)	344 (45·3%)	Reference		
At least one	443 (58·3%)	415 (54·7%)	1·17† (0·95 to 1·45)	0·149	3·6% (-1·4 to 8·6%)
Number of attendances per child	2 (1 to 2)	2 (1 to 3)	1·09‖ (0·93 to 1·28)	0·281	
Admission to hospital					
None	671 (88·3%)	660 (87·0%)	Reference		
At least one	89 (11·7%)	99 (13·0%)	0·87† (0·63 to 1·20)	0·407	-1·3% (-4·7 to 2·0%)
Number of admissions per child	1 (1 to 1)	1 (1 to 1)	0·93‖ (0·67 to 1·29)	0·663	
Attendance and/or admission					
None	306 (40·3%)	324 (52·7%)	Reference		
At least one	454 (59·7%)	435 (57·3%)	1·11† (0·89 to 1·37)	0·351	2·4% (-2·5 to 7·4%)
DNA outpatient appointment	***N=580***	***N=577***			
Attended all appointments	290 (50·0%)	289 (50·1%)	Reference		

For peer review only - http://bmjopen.bmj.com/site/about/guidelines.xhtml

Outcome	FNP	Usual care (UC)	Adjusted* parameter estimate (95% CI)	p-value	Absolute risk difference (FNP-UC) (95% CI)
DNA at least once	290 (50·0%)	288 (49·9%)	1·00† (0·79 to 1·26)	0·997	0·09% (-5·6 to 5·8%)

Data are n (%), n/N (%), or median (25th to 75th centile) unless otherwise stated. FNP = Family Nurse Partnership Programme + Usual Care. * FNP compared to usual care. Analysis adjusted for stratification (site), minimisation variables (gestational age, smoking status at recruitment, and first or preferred language); † Odds ratio from logistic model; ‡ Hazard ratio from Cox model; § A unique referral is counted as a distinct referral date per child; ‖ Incidence rate ratio from Poisson model; ¶ Relative risk ratio from multinomial model; ‡‡Using diagnosis in any position; **Multiple indicates when more than one category of abuse is relevant to the child's current plan; †† We reflect here the terminology used at the time of the trial but note the more contemporary 'care experienced' as a more familiar and accepted term. Source: National Pupil Database, The Department for Education

For peer review only

Table 3 Secondary outcomes: Subsequent pregnancies and registerable birth

	**FNP**	**Usual care**	**Adjusted* parameter estimate (95% CI)**	**p-value**	**Absolute risk difference (FNP-Usual care) (95% CI)**
Subsequent pregnancy	***N=753***	***N=753***			
No	163 (21·7%)	163 (21·7%)	Reference		
Yes	590 (78·4%)	590 (78·4%)	1·00† (0·79 to 1·28)	0·984	0·0% (-4·2 to 4·2%)
Subsequent registerable birth	***N=752***	***N=749***			
No	276 (36·7%)	266 (35·5%)	Reference		
Yes	476 (63·3%)	483 (64·5%)	0·95† (0·77 to 1·18)	0·662	-1·2% (-6·0 to 3·7%)
One birth	326 (68·5)	331 (68·5)	0·95‡ (0·76 to 1·19)	0·655	
Two	124 (26·1)	121 (25·1)	0·99‡ (0·73 to 1·34)	0·955	
Three births or more	26 (5·5)	31 (6·4)	0·80‡ (0·46 to 1·39)	0·435	
Inter-birth interval between first and second child (days)<br>Median (25th to 75th centiles)	1027 (590 to 1506·75)	1065 (665 to 1538)	0·99§ (0·88 to 1·13)	0·938	

Data are n (%), n/N (%), or median (25th to 75th centile) unless otherwise stated. FNP = Family Nurse Partnership Programme + Usual Care. * FNP compared to usual care. Analysis adjusted for stratification (site), minimisation variables (gestational age, smoking status at recruitment, and first or preferred language); † Odds ratio from logistic model; ‡ Relative risk ratio from multinomial model; § Hazard ratio from Cox model.

For peer review only - http://bmjopen.bmj.com/site/about/guidelines.xhtml

Table 4 Secondary outcomes: Child health, developmental and educational

**Outcome**	**FNP**	**Usual care**	**Adjusted* odds ratio (95% CI)**	**p-value**	**Absolute risk difference (FNP-Usual care) (95% CI)**
Special educational needs (SEN)† provision	**N=759**	**N=747**			
No	540 (71·1%)	502 (67·2%)	Reference		
Yes	219 (28·9%)	245 (32·8%)	0·83 (0·67 to 1·03)	0·097	-3·9% (-8·6 to 0·7%)
**Early educational attendance**	**N=759**	**N=747**			
Attending an Ofsted registered private, voluntary and independent establishment up to the age of 4 years	334 (43·9%)	308 (41·2%)	1·13 (0·91 to 1·40)	0·281	2·7% (-2·3 to 7·7%)
**School attendance**	**N=754**	**N=740**			
Overall absence					
No absences	14 (1·9%)	14 (1·9%)	Ref		
At least one absence	740 (98·1%)	726 (98·1%)	1·00 (0·47 to 2·12)	0·998	0·0% (-1·4 to 1·5%)
Overall authorised absence					
No absences	26 (3·4%)	26 (3·5%)	Ref		
At least one absence	728 (96·6%)	714 (96·5%)	1·01 (0·58 to 1·75)	0·984	0·1% (-1·8 to 2.8%)
Overall unauthorised absence					
No absences	256 (34·0%)	245 (33·1%)	Ref		
At least one absence	498 (66·0%)	495 (66·9%)	0·95 (0·76 to 1·18)	0·620	-0·8% (-5·6 to 3·9%)

For peer review only - http://bmjopen.bmj.com/site/about/guidelines.xhtml

Outcome	FNP	Usual care	Adjusted* odds ratio (95% CI)	p-value	Absolute risk difference (FNP-Usual care) (95% CI)
**Early Years assessment**	**N=743**	**N=728**			
**Achieving good level of development (GLD) ‡**					
Achieving GLD in all five areas of learning‖	431 (58·0%)	380 (52·2%)	1·26 (1·03 to 1·55)	0·026	5·8% (0·7 to 10·9%)
Achieving GLD in all 17 early learning goals	412 (55·5%)	365 (50·1%)	1·24 (1·01 to 1·52)	0·043	5·3% (0·2 to 10·4%)
**Total point score§** Mean (SD)	32·22 (7·25)	31·59 (7·62)	0·65 ‖ (-0·11 to 1·41)	0·094	
**Key Stage 1 assessments**	**N=740**	**N=732**			
**Reading**					
Lower than expected	257 (34·7%)	289 (39·5%)	Reference		
Reaching at least the expected standard¶	483 (65·3%)	443 (60·5%)	1·23 (0·99 to 1·53)	0·051	4·8 (-0·2 to 9·7%)
Expected standard	*371 (50·1%)*	*337 (46·0%)*	*1·24 (0·99 to 1·56)*	*0·056*	*4·1 (-1·0 to 9·2%)*
Higher standard	*112 (15·1%)*	*106 (14·5%)*	*1·20 (0·88 to 1·65)*	*0·250*	*0·7 (-3·0 to 4·3%)*
**Maths**					
Lower than expected	281 (38·0%)	283 (38·7%)	Reference		
Reaching at least the expected standard¶	459 (62·0%)	449 (61·3%)	1·04 (0·84 to 1·28)	0·731	0·7 (-4·3 to 5·6%)
Expected standard	*392 (53·0%)*	*376 (51·4%)*	*1·06 (0·85 to 1·32)*	*0·611*	*1·6 (-3·5 to 6·7%)*
Higher standard	*67 (9·1%)*	*73 (10·0%)*	*0·93 (0·64 to 1·35)*	*0·711*	*-0·9 (-3·9 to 2·1%)*

For peer review only - http://bmjopen.bmj.com/site/about/guidelines.xhtml

Outcome	FNP	Usual care	Adjusted* odds ratio (95% CI)	p-value	Absolute risk difference (FNP-Usual care) (95% CI)
**Science**					
Lower than expected	203 (27·4%)	219 (29·9%)	Reference		
Reaching at least the expected standard**	537 (72·6%)	513 (70·1%)	1·14 (0·91 to 1·43)	0·254	2·5 (-2·1 to 7·1%)
**Writing academic year 2016/17**	**N=498**	**N=487**			
Lower than expected	257 (51·6%)	278 (57·1%)	Reference		
Reaching at least the expected standard¶	241 (48·4%)	209 (42·9%)	1·24 (0·97 to 1·60)	0·090	5·5 (-0·7 to 11·6%)
Expected standard	*218 (43·8%)*	*182 (37·4%)*	*1·29 (1·00 to 1·68)*	*0·054*	*6·4 (0·3 to 12·5%)*
Higher standard	*23 (4·6%)*	*27 (5·5%)*	*0·92 (0·51 to 1·64)*	*0·769*	*-0·9 (-3·8 to 1·9%)*

Data are n (%), n/N (%), or median (25th to 75th centile) unless otherwise stated. FNP = Family Nurse Partnership Programme + Usual Care. SD=standard deviation. *FNP compared to usual care. Analysis adjusted for stratification (site), minimisation variables (gestational age, smoking status at recruitment, and first or preferred language); † A child with SEN provision is recorded using the following codes (A = School Action, P = School Action Plus, E = Education, Health and Care Plan, S=Statement, K= SEN support) and the N code was used to indicate No SEN support in any of the following datasets between 2013 and 2017. PLASC (Autumn, Spring and Summer terms), AP, PRU Census, and EYC. If for any of the years no response was recorded then the assumption was that the child was not included in the denominator as either not present on Census day or not in school; ‡ Children achieving a good level of development are those achieving at least the expected level within the prime and specific areas of learning; §Total point score ranges from 17 to 51 with a higher score indicating a better level of development; ‖ Mean difference from linear model; ¶ Working at the expected standard and at a greater depth within the expected standard; **Working at a greater depth within the expected standard is not applicable in science. Source: National Pupil Database, The Department for Education

For peer review only - http://bmjopen.bmj.com/site/about/guidelines.xhtml

Evidence of positive programme impacts were found for some educational outcomes assessed at the end of the reception year and at KS1 by which time the child would be seven years old (Table 4). Children in the FNP arm were more likely to reach a good level of development across all five areas of learning by the end of the reception year (58.0%) than children in the UC arm (52.2%) and also to achieve a good level of development in all 17 early learning goals (FNP: 55.5%, UC: 50.1%). The small advantage for children of nurse-visited mothers was consistent across all five areas of learning although not tested statistically (Table S10).

For KS1 reading, writing, maths and science assessments there was no evidence of a difference between arms in the proportion of children reaching at least the expected standard nor specifically in those working at the expected level or at a greater depth of knowledge (Table 4). What is observed from the writing assessment is the increase in the rates of children reaching at least the expected standard between the two academic years (Overall 45.7% in 2016/17 to 66.5% in 2017/18), reflective of all assessments across the two academic years in each arm (Table S11). This is mainly explained by the distribution of births for the children for each academic year (Figure S1). Reflecting the recruitment period of the trial, children with KS1 results available in the academic year 2016/17 were born between October 2009 and August 2010 but skewed towards the summer born children (42% born between June and August 2010) whereas children appearing in the 2017/18 academic year were born between September 2010 and February 2011. There is a nationally observed relationship between rates of children reaching the expected standard and month of birth e.g. a 17% difference in reading for children born in August and September.[16] Therefore, our 2017/18 cohort of children do not represent the whole academic year, but are biased towards those that are more likely to achieve. This indicates that the rates of children reaching the expected standard varies by their month of birth and is important as a moderator of programme effect. Importantly, the original trial allocation ensured study arms remained balanced with regards month of birth and the intervention had no impact on total weeks gestation.[5] As a sensitivity analysis, the main analyses were adjusted for the child's month of birth (categorised into quarter of birth) and evidence of a between arm difference was found in reading assessments (Table S12). In addition, the Early Years assessments were adjusted for child's month of birth, which strengthened the association found in Table 4.

Details of all planned sub-group analyses and further exploratory analyses are shown in the appendix (Tables S13 to S17).

Cost

There were negligible resource use and cost differences for both mothers (p=0.393) and children (p=0.865) between study arms (Table S18). The adjusted incremental costs of programme delivery per women in BB:0-2 (£1,811) remain the key observed cost difference between study arms.

For peer review only - http://bmjopen.bmj.com/site/about/guidelines.xhtml

Discussion

Principal findings

In this data linkage study we found that adding FNP to usually provided health and social care provided no additional benefit for maltreatment outcomes including the numbers of children referred to CSCS, registered as in need of additional support, given a child protection plan, entering care, attending an emergency department or being admitted for an injury or ingestion, or duration of stay if admitted. While there was no difference between study arms in the small number of children in care, the UC arm experienced on average two more months in care compared to the FNP arm. The total number of child deaths found was less than ten and therefore disclosive and cannot be reported. High rates of children ever registered as in need in both study arms indicates substantial need amongst families. We found the programme led to more children reaching a good level of development at the end of school reception year and, when taking month of birth into account, improvements in reading at KS1. Writing scores improved as a result of FNP for boys, children of younger mothers and of mothers who were not in education, employment or training when first recruited to the study. We found no other difference between families who received FNP and those who did not. With no differences in secondary health resource use and costs for FNP, the programme can be considered cost-neutral compared to usual care in the medium-term (2-6 years).

Strengths and weaknesses of the study

The prioritisation of outcomes in our original trial attracted some debate.[17-19] However, the trial and the current follow-on study are both comprehensively reported, and preceded by a priori analysis plans providing transparency and guarding against undue post hoc interpretation.[6-8] Although a primary outcome of child being in need of additional support was incorporated into the study design, we adopted a multimethod multisource approach to look at the range of maltreatment outcomes in drawing our conclusions. The consistent pattern of results within the two principal outcome domains provides greater confidence in what we can conclude. While the study benefited from random allocation in the BB:0-2 trial, when assessing educational outcomes we additionally adjusted for month of birth. This was because at a national level, month of birth is strongly associated with educational outcomes and the distribution of participants recruited to the trial varied by month.

The available maltreatment data provides an essential overview of family experience as recorded in official records but may lack some depth which could have allowed a more nuanced understanding for children referred. The educational data are teacher-reported statutory tests but similarly provide less detailed insight into child functioning which research instruments may have provided. The lack of detailed social care and any primary care resource use data limited the perspective of the economic analysis. However, using administrative data has enabled comprehensive cohort follow-up, with few

For peer review only - http://bmjopen.bmj.com/site/about/guidelines.xhtml

cases lost for analysis preserving the benefits of the original trial randomisation. The data included are also objectively recorded rather than maternally self-reported, allow for easy comparison nationally and over time. Our approach has gathered more data at less cost than comparable prospective data collection and minimised family burden.

In our previous trial report of outcomes at 24 months postpartum, there were higher rates of safeguarding events recorded in GP notes for children in the FNP arm (n=64/469, 13.6%), compared to the usual care arm (n=38/476, 8.0%).[5] Additionally, more mothers in the FNP arm reported their child had ever been referred to social services (n=119/580, 20.5%) compared to mothers in the usual care arm (n=91/541, 16.8%). These finding can be indicative of surveillance bias or more accurate reporting by mothers in the FNP arm respectively. In the current study a tendency for children to be referred at lower thresholds of concern or closer involvement by attending FNP nurses may have been evidenced through a greater proportion of children referred to social services being assessed as requiring no additional support. However, we did not observe this in our cohort.

With no absolute external criterion for maltreatment, a reliance solely upon formal child protection reports may be limiting. In the US Elmira trial, a review of 42 children (intervention arm n=13, control arm n=29) referred to CPS between birth and their fourth year of life found few differences in the nature of maltreatment recorded. However, measures of the quality of home environment, parenting, child intellectual functioning and use of healthcare services between children's 25th and 50th months of life favoured families in the intervention arm.[20] It has been suggested that both parental and professional behaviour may lead to programme enrolled CPS reported children being at lower risk of harm than their counterparts in receipt of usual care alone. Thresholds for referral to social services may differ between specialist home-visiting nurses with high contact time, more in-depth relationship with clients and different approaches to risk and safety management and other health, social and educational care professionals.[21] CPS referrals for children in receipt of FNP at lower levels of apparent concern would make it harder to compare on such outcomes, particularly during the programme delivery period (ie up to the child's second birthday). However, subsequent progress of children through the child protection system and later occurring outcomes (for example, children being taken into care) may be less liable to such influences but in our sample such numbers are small.

Verified outcomes documented in administrative records contrast with maternal subjective self-reports for which there may be no potential external criterion (ie a child being referred to CPS is a verifiable event, whereas maternal well-being is not). Nevertheless, the presentation or referral of families and their assessment against legally defined statuses such as being a Child in Need requires professional judgement. It has been suggested that surveillance bias is not likely to be problematic for assessing maltreatment using verified service data (i.e. by inflating the numbers of children being

For peer review only - http://bmjopen.bmj.com/site/about/guidelines.xhtml

reported when in receipt of home-visiting).[22-23] As others have found this could be higher when clients are actively engaged with services and as noted above, the potential for such bias remains.[24]

It has been proposed that other documented evidence such as hospital admission due to injury and ingestions and in particular duration of subsequent stay may provide a better indicator of maltreatment severity. In the follow-up of the US Memphis trial cohort at two years of the child's life, children in the intervention arm admitted following an injury or ingestion spent fewer days in hospital than their counterparts in the control arm. In the US Elmira trial, no difference between trial arms were found in the number of days hospitalised during the 25- to 50-month period of follow-up after adjustments were made for one outlier (in the intervention arm) assessed as being not relevant to parental caregiving.[25] We explored length of hospital stay following admission for an injury and ingestion both overall and for time periods representing periods of increasing of child mobility (ie under 1 year of age, 1 to under 2 years of age and 25 months to under 6 years of age). These were similar in each case but factors other than severity of clinical need may influence how long children remain in hospital. Wide differences exist between countries for hospital stay duration following injuries, ingestions and other consequences of external causation (when assessed across adult and paediatric populations).[26] One observational UK study of predictors of length of stay amongst paediatric inpatients (all causes) shows the contribution of ,health service factors in addition to clinical and social factors.[27] Interestingly, a large US study amongst paediatric inpatients found that non-accidental trauma compared to accidental trauma was a strong predictor of having a prolonged hospital stay even after adjusting for injury severity.[28]Therefore, length of hospital stay may be indicative of maltreatment, severity of clinical presentation, health service factors and possibly social factors. Further clarifying the factors that independently drive length of hospital stay and the validity of this outcome as a measure of maltreatment will be valuable.

Potential sources of bias

As an open label study, it is possible that service professionals may have attempted to compensate for the lack of specialist support from FNP for participants in the usual care arm. However, it should be noted that enhanced care would have been expected as part of usual practice for teenage mothers, for example, through teenage pregnancy midwives. The number of midwifery contacts were recorded in the trial and found to be equivalent between FNP and UC arms.[9] The number of home visits from specialist public health nurses (SPHN) reported by trial participants by 18 months was also equivalent between study arms. As would have been expected, the number of clinic contacts with SPHNs was greater for women in the control arm than in the FNP arm. In contrast, women allocated to the intervention arm would have received an average of just over 40 home visits from a family nurse before graduating from the programme. Overall, this suggests a common core of usual care provided across both trial arms, a higher level of SPHN input to the usual care arm reflecting in part the

For peer review only - http://bmjopen.bmj.com/site/about/guidelines.xhtml

responsibility of delivering the Healthy Child Programme (which was formally allocated to FNP nurses rather than SPHNs for their clients). The capacity of SPHNs to provide additional support will also be limited by their caseload. The maximum caseload currently recommended by the Institute of Health Visiting is one practitioner per 250 children. However, in 2015, 28% of surveyed practitioners in England reported caseloads in excess of 400 children and 12% reported between 500 and 1000+ families.[29]

Comparison with other studies

The strongest evidence for the programme preventing maltreatment remains from the original US Elmira trial. While the programme reduced the number of substantiated reports of abuse and neglect in the first 15 years of the child's life, the advantage emerged after age four years.[30] We found differences in duration spent in care for looked after children, but the numbers involved are small and across the range of maltreatment outcomes the pattern is of no overall group difference. The programme was no more effective in tested sub-groups. It is worth recognising differences in the population of clients in receipt of the programme across the different study populations, their social circumstances (which also may have changed over time) and how that may determine potential to benefit from FNP.[9] We have not directly explored differences in social care systems between England and the US but these may vary the underlying risk of maltreatment and its likelihood of detection.[31]

School entry reading and maths is of important predictive value for later school achievement.[32] Early childhood educational investment programmes typically show a modest impact upon cognitive and achievement scores (e.g. weighted average of 0.21 standard deviations) with some decline in observed impact being attributed to general improvements in both school and home environments.[33,34] The question of comparator is therefore of particular relevance. In this study, a range of state and third sector funded services (including midwifery and health visiting) would have been available as usual care, possibly limiting the incremental benefit possible from the family nurse partnership programme.[9] Nevertheless, as noted above women allocated to FNP received substantial input from family nurses, albeit fewer than possible according to the maximum fidelity targets at the time of the trial.

Home visiting interventions, which aim to impact upon a range of outcomes, have shown similarly modest effects on child cognitive outcomes (e.g. mean Effect Size 0.25, 96% CI 0.11, 0.38) with considerable variability due to programme composition (e.g. greater effects with programmes addressing parental responsiveness, sensitivity to care and nurturing and rehearsal and role-playing).[35] Our BB:0-2 study found benefits at age two years for children in the FNP arm for both language and developmental outcomes but these outcomes were maternally reported and potentially subject to reporting bias in the open trial.[8] This new medium-term evidence for programme impact upon

For peer review only - http://bmjopen.bmj.com/site/about/guidelines.xhtml

developmental outcomes adds to that picture, although the size of effect remains small. Similar medium-term programme impact (to age six years old) on developmental outcomes (eg, intellectual functioning and receptive language scores) was reported from the Memphis trial.[36] A reanalysis of those data found cognitive benefits at age 6 years attributed to both programme induced improvements in maternal traits and family life investments at age 2.[37]

Unanswered questions and future research

Given small but positive findings for developmental outcomes, tracking the cohort through to subsequent school years is warranted. As social care data are equally accessible via the NPD there is value in observing the trajectory for maltreatment outcomes given that the principal maltreatment findings from the US Elmira trial are located in the 15-year follow-up. Linkage to data in other sectors such as welfare would provide a greater understanding of broader impact for families. Such work should be driven by the programme's logic model, existing evidence for long-term programme impact, informed by programme evolution in England and supplemented by work to understand what may explain differing programme benefits across national settings.[30,38]

Conclusions

There are no evident programme benefits for maltreatment outcomes by age six but the FNP generates higher rates of child attainment at the end of the reception year and at KS1. The adapted programme remains locally commissioned and delivered in England.[38,39] Local needs and priorities may determine the weight attached to these different sets of outcomes.

Acknowledgements

The Centre for Trials Research receives funding from Health and Care Research Wales and Cancer Research UK. The work was undertaken with the support of The Centre for the Development and Evaluation of Complex Interventions for Public Health Improvement (DECIPHer), a UKCRC Public Health Research Centre of Excellence. Joint funding (MR/KO232331/1) from the British Heart Foundation, Cancer Research UK, Economic and Social Research Council, Medical Research Council, the Welsh Government and the Wellcome Trust, under the auspices of the UK Clinical Research Collaboration, is gratefully acknowledged.

Data re-use statement: Copyright © (2019), the Health and Social Care Information Centre. Re-used with the permission of the Health and Social Care Information Centre. All rights reserved.

We would like to thank all members of CASCADE voices, ALPHA and Our Place for contributing to the study including those who co-ordinated access to these individuals. Thanks are due to the FNP teams in Bristol, Bath and Wiltshire who helped us to make contact with FNP clients and graduates

For peer review only - http://bmjopen.bmj.com/site/about/guidelines.xhtml

during our PPI work. We would also like to acknowledge the contribution of members of the study steering committee - Ann Louise Kinmonth (Chair), David Low, Gordon Taylor, Nina Biehal, Rachel Tonkin, Stavros Petrou, and Silvia van den Heijkant.

This work uses data provided by patients and collected by the NHS as part of their care and support as well as data from schools and local authorities. Using patient and other public data is vital to improve health and care for everyone. There is huge potential to make better use of information from people's patient records, to understand more about disease, develop new treatments, monitor safety, and plan NHS services. Patient and other public data should be kept safe and secure, to protect everyone's privacy, and it's important that there are safeguards to make sure that it is stored and used responsibly. Everyone should be able to find out about how patient data is used. #datasaveslives You can find out more about the background to this citation here: https://understandingpatientdata.org.uk/data-citation. [To note. This has been adapted to include other data sources not made available from the NHS such as education and social services data].

Footnotes

Author contributions: MR was the chief investigator and acts as guarantor for the study. MR, FLW, RCJ, LA, SC, DF, KH, JK, GM, EOJ, RP, JSa, JSe, TS contributed to study design. FLW was the study manager. RCJ was the lead statistician. DF supervised the health economics. MR, FLW, RCJ, LA, SC, DF, KH, JK, GM, EOJ, RP, JSa, JSe, TS were responsible for study management. GM and LA were the data managers. JK and JS led for public involvement. GM and LA cleaned the data. RCJ designed and conducted the analysis. RP conducted the economic analysis. MR, JSa, RCJ, SC, KH, JK, EOJ, JSe were co-applicants on the original funding application. MR, FLW, RCJ, LA, SC, DF, KH, JK, GM, EOJ, RP, JSa, JSe, TS interpreted the results of the analysis. RC-J and RP were responsible for producing the tables. MR, FLW, RC-J and RP drafted the study report. MR, FLW, RCJ, LA, SC, DF, KH, JK, GM, EOJ, RP, JSa, JSe, TS reviewed manuscript drafts, revised for important intellectual content and approved the final version.

Funding: This project was funded by the National Institute for Health Research Public Health Research (NIHR PHR) Programme (reference:11/3002/11). The views and opinions expressed therein are those of the authors and do not necessarily reflect those of the NIHR PHR Programme or the Department of Health. The funders had no role in considering the study design or in the collection, analysis, interpretation of data, writing of the report, or decision to submit the article for publication.

Competing interests: All authors have completed the ICMJE uniform disclosure form at www.icmje.org/coi_disclosure.pdf and declare: all authors declare financial support from NIHR PHR

For peer review only

for the submitted work; no financial relationships with any organisations that might have an interest in the submitted work in the previous three years; no other relationships or activities that could appear to have influenced the submitted work.

Ethical approval: Ethical approval for this study was granted by the Health Research Authority research ethics committee (Wales REC 3; REC reference 14/WA10062) and section 251 support was provided by the Confidentiality Advisory Group (CAG reference CAG 10-08(b)/2014).

Data sharing: Anyone wishing to access the linked datasets for research purposes should apply via the CAG to the Health Research Authority to access patient identifiable data without consent and then to the NHS Digital, National Pupil Database and the abortion statistics – Department for Health and Social Care. In the first instance, enquiries about access to the data should be addressed to Professor Mike Robling, Centre for Trials Research, Cardiff University.

The lead author (MR) affirms that the manuscript is an honest, accurate, and transparent account of the study being reported; that no important aspects of the study have been omitted; and that any discrepancies from the study as planned (and, if relevant, registered) have been explained.

Dissemination to participants and related public communities: The findings from the BB:2-6 study, including those from this paper will be disseminated directly to participants where valid contact details remain available, via social media and conference presentations. This will include the use of lay-informed infographic materials. Summary results will also be made available on the study webpage [https://evidencinghomevisiting.co.uk/].

References

1. Olds DL, Henderson CR, Chamberlin R, Tatelbaum R. Preventing Child Abuse and Neglect: A Randomized Trial of Nurse Home Visitation. Pediatrics. 1986;78(1) [accessed 2017 May 5]. Available from: http://pediatrics.aappublications.org/content/pediatrics/78/1/65.full.pdf

2. Kitzman H, Olds DL, Henderson CR, Hanks C, Cole R, Tatelbaum R, et al. Effect of Prenatal and Infancy Home Visitation by Nurses on Pregnancy Outcomes, Childhood Injuries, and Repeated Childbearing. JAMA. 1997;278(8):644 [accessed 2017 May 5]. Available from: http://jama.jamanetwork.com/article.aspx?doi=10.1001/jama.1997.03550080054039

3. Olds DL, Robinson J, O'brien R, Luckey DW, Pettitt LM, Henderson CR, et al. Home Visiting by Paraprofessionals and by Nurses: A Randomized, Controlled Trial. Pediatrics. 2002;110(3) [accessed 2017 May 5]. Available from: http://pediatrics.aappublications.org/content/110/3/486.short

4. Mejdoubi J, van den Heijkant SC, van Leerdam FJ, Heymans MW, Crijnen A, Hirasing RA. The effect of VoorZorg, the Dutch nurse–family partnership, on child maltreatment and development:

For peer review only

a randomized controlled trial. PLOS ONE 2015;10:e0120182. https://doi.org/10.1371/journal.pone.0120182

5. Robling M, Bekkers MJ, Bell K, Butler CC, Cannings-John R, Channon S, et al. Effectiveness of a nurse-led intensive home-visitation programme for first-time teenage mothers (Building Blocks): A pragmatic randomised controlled trial. Lancet. 2016;387:146–55. Available from: http://www.thelancet.com/journals/lancet/article/PIIS0140-6736(15)00392-X/fulltext

6. Lugg-Widger F, Cannings-John R, Channon Sue, Fitzsimmons D, Hood K, Jones K, et al. Assessing the medium-term impact of a home-visiting programme on child maltreatment in England: protocol for a routine data linkage study. BMJ Open. 2017;7(e015728) [accessed 2017 Jul 17]. Available from: http://bmjopen.bmj.com/content/bmjopen/7/7/e015728.full.pdf

7. Robling M, Bekkers MJ, Bell K, Butler CC, Cannings-John R, Channon S, et al. The Building Blocks Trial (Main report). 2014. Available from: https://www.cardiff.ac.uk/__data/assets/pdf_file/0009/504729/Building-Blocks-Full-Study-Report.pdf

8. Owen-Jones E, Bekkers M-J, Butler CC, Cannings-John R, Channon S, Hood K, et al. The effectiveness and cost-effectiveness of the Family Nurse Partnership home visiting programme for first time teenage mothers in England: a protocol for the Building Blocks randomised controlled trial. BMC Pediatr. 2013;13:1.

9. Robling M, Cannings-John R, Channon S, Hood K, Moody G, Poole R, et al. What is usual care for teenagers expecting their first child in England? A process evaluation using key informant mapping and participant survey as part of the Building Blocks randomised controlled trial of specialist home visiting. BMJ Open. 2018;8(5).

10. Lyons RA, Jones KH, John G, Brooks CJ, Verplancke J-P, Ford D V, et al. The SAIL databank: linking multiple health and social care datasets. BMC Med Inform Decis Mak. 2009;9(9) [accessed 2017 May 12]. Available from: http://www.biomedcentral.com/1472-6947/9/3

11. Jones KH, Ford D V., Jones C, Dsilva R, Thompson S, Brooks CJ, et al. A case study of the Secure Anonymous Information Linkage (SAIL) Gateway: A privacy-protecting remote access system for health-related research and evaluation. J Biomed Inform. 2014;50:196–204 [accessed 2017 May 12]. Available from: http://www.sciencedirect.com/science/article/pii/S1532046414000045

12. Jones KH, Ford DV, Thompson S, Lyons R. A Profile of the SAIL Databank on the UK Secure Research Platform. Int J Popul Data Sci. 2019;4(2) [accessed 2020 Jun 30]. Available from: https://ijpds.org/article/view/1134

13. Department for Education. Children In Need in England, including their characteristics and further information on children who were the subject of a child protection plan (2009-10 Children in Need census, Final. 2010.

14. HM Treasury. The Green Book, Central Government Guidance on Appraisal and Evaluation. 2020. [accessed 2020 Dec 7]. Available from: https://assets.publishing.service.gov.uk/government/uploads/system/uploads/attachment_data/file/938046/The_Green_Book_2020.pdf

15. Lugg-widger F, Cannings-john R, Angel L, Moody G, Segrott J, Kenkre J, et al. Assessing the impact of specialist home visiting upon maltreatment in England : a feasibility study of data linkage from a public health trial to routine health and social care data. Pilot feasibility Stud. 2018;4(1):98.

16. Department for Education. Phonics screening check and Key stage 1 assessments in England, 2016 (SFR 42/2016). London: Department for Education; 2016.

For peer review only - http://bmjopen.bmj.com/site/about/guidelines.xhtml

17. Olds D. Building evidence to improve maternal and child health. Vol. 387, The Lancet. Lancet Publishing Group; 2016. p. 105–7 [accessed 2020 Jul 23]. Available from: http://dx.doi.org/10.1016/S0140-6736

18. Barlow J, Barnes J, Sylva K, Fonagy P, Fearon P. Questioning the outcome of the Building Blocks trial. Vol. 387, The Lancet. Lancet Publishing Group; 2016. p. 1615–6 [accessed 2020 Jul 23]. Available from: http://www.dh.gov.uk/

19. Robling M, Torgerson D, Pickett K, Channon S, Sanders J. Questioning the outcome of the Building Blocks trial - Authors' reply. Vol. 387, The Lancet. Lancet Publishing Group; 2016. p. 1616–7 [accessed 2020 Jul 23]. Available from: http://www.dh.gov.uk/

20. Olds D, Henderson CR, Kitzman H, Cole R. Effects of prenatal and infancy nurse home visitation on surveillance of child maltreatment. Pediatrics 1995; 95; 365-372.

21. Williams, VN, Ayele, R, Shimasaki, S, Tung, GJ, Olds, D. "Risk assessment practices among home visiting nurses and child protection caseworkers in Colorado, United States: A qualitative investigation." Health & Social Care in the Community. 2019;27(5): 1344-52. doi.org/10.1111/hsc.12773

22. Drake B, Jonson-Reid M, Kim H. Surveillance bias in child maltreatment: a tempest in a teapot. Int J Environ Res Public Health 2017;14:E971. https://doi.org/10.3390/ijerph14090971

23. Chaffin M, Bard D. Impact of intervention surveillance bias on analyses of child welfare report outcomes. Child Maltreat 2006;11:301–12. https://doi.org/10.1177/107755950629

24. Zielinski DS, Eckenrode J, Olds DL. Nurse home visitation and the prevention of child maltreatment: Impact on the timing of official reports. Dev Psychopathol. 2009;21(2):441–53 [accessed 2019 Aug 2]. Available from: https://www.cambridge.org/core/product/identifier/S0954579409000248/type/journal_article

25. Olds DL, Henderson Jr CR, Kitzman H. Does prenatal and infancy nurse home visitation have enduring effects on qualities of parental caregiving and child health at 25 to 50 months of Life? Pediatrics 1994; 93; 89-98.

26. https://ec.europa.eu/eurostat/statistics-explained/index.php?title=Hospital_discharges_and_length_of_stay_statistics#Average_length_of_hospital_stay_for_in-patients Date accessed: 30.7.21

27. Heys M, Rajan M, Blair M. Length of paediatric inpatient stay, socio-economic status and hospital configuration: a retrospective cohort study. BMC Health Services Research 2017 17: 274 DOI 10.1186/s12913-017-2171-x

28. Livingston JK, Grigorian A, Kuza CM, Lekawa M, Bernal N, Allen A, Nahmais J. Non-accidental trauma increases length of stay and mortality in pediatric trauma. Pediatric Surgery International (2019) 35:779–784 doi.org/10.1007/s00383-019-04482-5

29. Institute of Health Visiting. Health Visiting in England: State of Health Visiting in England: Results from a survey of 1040 practising health visitors. 2020 [accessed 2021 May 21]. Available from: https://www.activematters.org/wp-content/uploads/2020/03/State_of_Health_Visiting_survey.pdf

30. Olds DL, Eckenrode J, Henderson CR, Kitzman H, Powers J, Cole R, et al. Long-term Effects of Home Visitation on Maternal Life Course and Child Abuse and Neglect Fifteen-Year Follow-up of a Randomized Trial. JAMA. 1997;278(8):637–43.

For peer review only

31. Moody G, Cannings-John R, Hood K, Kemp A, Robling M. Establishing the international prevalence of self-reported child maltreatment: A systematic review by maltreatment type and gender. Vol. 18, BMC Public Health. BioMed Central Ltd.; 2018. p. 1164 [accessed 2020 Jul 23]. Available from: https://bmcpublichealth.biomedcentral.com/articles/10.1186/s12889-018-6044-y

32. Duncan GJ, Dowsett CJ, Claessens A, Magnuson K, Huston AC, Klebanov P, et al. School Readiness and Later Achievement. Dev Psychol. 2007;43(6):1428–46.

33. Duncan G, Magnusson K. Early childhood interventions for low-income children. Focus (Madison). 2014;31.

34. Duncan G, Magnuson K. Investing in Preschool Programs. J Econ Perspect. 2013;27(2):109–31.

35. Filene JH, Kaminski JW, Valle LA, Cachat P. Components associated with home visiting program outcomes: A meta-analysis. Pediatrics. 2013;132(SUPPL.2):S100–9 [accessed 2021 Jan 8]. Available from: www.pediatrics.org/cgi/doi/10.1542/peds.2013-1021H

36. Olds DL, Kitzman H, Cole R, Robinson J, Sidora K, Luckey DW, et al. Effects of Nurse Home-Visiting on Maternal Life Course and Child Development: Age 6 Follow-Up Results of a Randomized Trial. Pediatrics. 2004;114(6):1550–9. Available from: http://pediatrics.aappublications.org/cgi/doi/10.1542/peds.2004-0962

37. Heckman JJ, Holland ML, Makino KK, Pinto R, Rosales-Rueda M. An Analysis of the Memphis Nurse-Family Partnership Program. NBER Work Pap. 2017;23610. Available from: http://www.nber.org/papers/w23610%0Ahttp://www.nber.org/papers/w23610

38. Family Nurse Partnership National Unit & Dartington Service Design Lab. FNP ADAPT: Using evidence, pragmatism and collaboration to change the FNP programme in England. London, UK; 2020. Available from: www.fnp.nhs.uk

39. The Family Nurse Partnership |. [accessed 2020 Jul 23]. Available from: https://www.fnp.nhs.uk/

For peer review only

Figure Legend

Figure 1 Participant flow chart

Describing follow-up within the BB0:0-2 trial and formation of the BB:2-6 study cohort. MW=Mandatory withdrawals; EW=elective withdrawals; M=Mother; C=Child, FNP=Family Nurse Partnership (trial arm); UC=Usual care (trial arm); BB:0-2=Building Blocks Randomised Controlled Trial; BB:2-6=Building Blocks Cohort Study; NPD=National Pupil Database

For peer review only

Figure 1 Flow chart

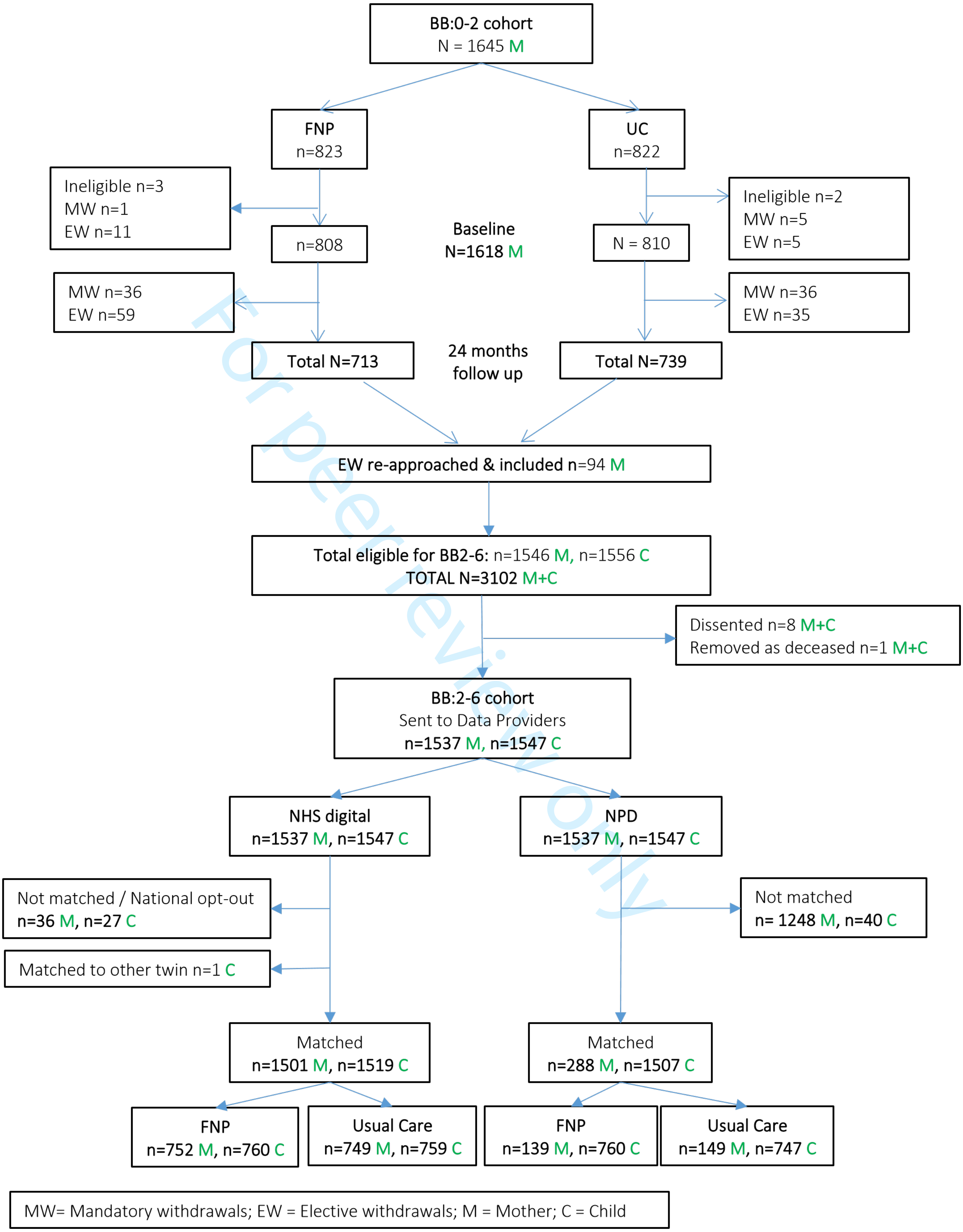

Supplementary appendix

I. Number of valid FNP visits received

- Table S1 BB:2-6 study population data: Valid FNP visits per phase

II. Study assessment domain, outcomes, principal data providers and data obtained

- Table S2 BB:2-6 Study assessment domains, key and exploratory outcomes and principal data providers

III. Data accrued from providers

- Table S3 Numbers (%) of mothers and children with data returned by dataset

IV. Further details of Child in Need outcome

V. Representativeness of study sample

- Table S4 Maternal characteristics of those in BB:0-2 compared to those in the BB:2-6
- Table S5 Child characteristics of those in the BB:0-2 study compared to those in the BB:2-6 study

VI. Descriptive secondary outcomes and additional Key Stage 1 results

- Table S6 Child In Need referrals, Children's Social Services referrals and hospital admissions: descriptives
- Table S7 Emergency attendance to hospital by injury or ingestion, by trial arm
- Table S8 Admissions by injury/ingestion type and trial arm
- Table S9 Hospital admissions for injuries and ingestions by trial arm and broken down by age categories
- Table S10 Child health, developmental and educational: descriptives
- Table S11 Children reaching the expected standard in KS1 assessments by trial arm and academic year
- Figure S1 Distribution of children's month of birth by trial arm
- Table S12 Comparison of parameter estimates before and after adjustment for child's month of birth

VII. Results of planned sub-group analysis

- Table S13 Subgroup analyses for referrals to Children's social care services
- Table S14 Subgroup analyses for child in need status
- Table S15 Predefined subgroup analyses on Early Years Assessment total point score
- Table S16 Subgroup analyses on KS1 assessments reaching the expected standard (RES)
- Table S17 Subgroup analyses on reaching the expected standard in the writing KS1 assessment for 2016/17 academic year

VIII. Treatment efficacy

IX. Missing data

X. Cost Consequence Analysis

- Table S18 Cost-consequence balance sheet for the family-nurse based intervention for the four years between children's ages 2 and 6.

For peer review only - http://bmjopen.bmj.com/site/about/guidelines.xhtml

I. Number of valid FNP visits received

Of the 766 women in the FNP arm in the BB:2-6 cohort, 69 did not enrol for FNP. For the remaining 697 who were randomised and enrolled, 695 had received at least one valid visit and two did not receive any valid visits across the three phases (pregnancy, infancy and toddlerhood). The descriptives in Table S1 are based on 697 women. A greater proportion of women received no visits during the infancy and toddler phases (40 (5.7%) and 98 (14.1%) respectively) compared to the pregnancy phase (6 (0.7%)). This is similar to that reported in the BB:0-2 cohort with comparable median valid visits and proportions reaching the fidelity goals (see Lancet Supplementary appendix Table S11.3).[1]

Table S1 BB:2-6 study population data: Valid FNP visits per phase

	**Pregnancy**	**Infancy**	**Toddlerhood**
N women randomised and enrolled to FNP	697	697	697
Women not receiving any valid visits n(%)	6 (0·7)	40 (5·7)	98 (14·1)
Women receiving at least one valid visit n(%)	691 (99·3)	657 (94·3)	599 (85·9)
Valid visits received			
Median (25th to 75th centile)	10 (8 to 12)	19 (15 to 22)	13 (10 to 16)
Mean (SD)	9·74 (3·42)	18·7 (5·97)	13·28 (5·46)
Reaching fidelity goal of: n(%)			
80%+	401/697 (57·5)	-	-
65%+	-	364/697 (52·2)	-
60%+	-	-	297/697 (42·6)

[1] Robling M, Bekkers MJ, Bell K, Butler CC, Cannings-John R, Channon S, et al. Effectiveness of a nurse-led intensive home-visitation programme for first-time teenage mothers (Building Blocks): A pragmatic randomised controlled trial. Lancet. 2016;387:146–55. [accessed 2019 Nov 12]. Available from: http://www.thelancet.com/journals/lancet/article/PIIS0140-6736(15)00392-X/fulltext

For peer review only - http://bmjopen.bmj.com/site/about/guidelines.xhtml

II. Study assessment domain, outcomes and principal data providers

Table S2 BB:2-6 Study assessment domains, key and exploratory outcomes and principal data providers

Domains	Outcomes	Maternal	Child	HES	ONS	NPD	Data source	Analysis
Primary: Child in Need status recorded at any time during the follow-up period.	CIN status		✓			✓	CIN	LO
	Exploratory:							
	Age at first CIN referral		✓			✓	CIN	*SURV*
	Number of CIN referrals per child		✓			✓	CIN	*PO*
Secondary: (i) Objective measures of maltreatment	CIN categorisation		✓			✓	CIN	MNOM
	CIN duration		✓			✓	CIN	SURV
	Referral to Children's social care services		✓			✓	CIN	LO
	Exploratory: Age at first referral		✓			✓	CIN	SURV
	Child Protection Plan		✓			✓	CIN	LO
	Child Protection Plan categorisation		✓			✓	CIN	MNOM
	Looked after status		✓			✓	CLA	LO
	CLA period of care		✓			✓	CLA	PO
	Legal status of CLA		✓			✓	CLA	D
	Cause of death		✓		✓		ONS- deaths	D
(ii) Associated measures of maltreatment	Injuries and ingestions:							
	Emergency attendance		✓	✓			A&E	LO
	Admission to hospital		✓	✓			IP	LO
	Attendance and/or admission		✓	✓			A&E, IP	LO
	DNA appointments		✓	✓			OP	LO
	Exploratory:							

For peer review only - http://bmjopen.bmj.com/site/about/guidelines.xhtml

Domains	Outcomes	Maternal	Child	HES	ONS	NPD	Data source	Analysis
	Number of admissions per child<br>*Length of hospital admission stay*		✓<br>✓	✓<br>✓			IP<br>IP	PO<br>D
(iii) Maternal outcomes	Subsequent pregnancy	✓		✓			OP, IP, Ab	LO
	Exploratory:<br>Number of births<br>Inter-birth interval between first and second child	✓<br>✓		✓<br>✓			IP<br>IP	PO<br>SURV
(iv) Costs	Health and Social Care resource use	✓	✓	✓		✓	OP, IP, A&E matched with NHS reference costs	D
(v) Child health, developmental and educational outcomes	Special educational needs provision<br>Disability<br>Early education attendance<br>School attendance<br>Early Years assessment<br>KS1 attainment		✓<br>✓<br>✓<br>✓<br>✓<br>✓	✓		✓<br>✓<br>✓<br>✓<br>✓<br>✓	PLASC, AP, PRU Census, EYC<br>NA<br>PLASC, EYC<br>Absence<br>EYFSP<br>KS1	LO<br>NA<br>LO<br>LO<br>LIN, LO<br>LO
	Exploratory:<br>Early education attendance by School or an Ofsted registered PVI establishment<br>Earliest type of day care		✓<br>✓			✓<br>✓	PLASC, EYC<br>EYC	D<br>D

Ab - Abortions; A&E – Accident and Emergency; AP – Alternative Provision; CIN – Child in Need; CLA – Child Looked After; D – Descriptive; DNA – Did not attend; EYC – Early Years Census; EYFSP –Early years foundation stage profile; FNP – Family Nurse Partnership; HES – Hospital Episode Statistics; IP - Inpatients; KS1 – Key Stage 1; LIN – Linear regression; LO – Logistic; MNOM - multinomial NA - Not available; NPD – National Pupil Database; ONS – Office for National Statistics; OP – Outpatients; PLASC – Pupil Level and School Census; PO-Poisson; PRU – Pupil Referral Unit Census.; PVI – Private, Voluntary or Independent (PVI) establishment; SURV – Survival.

For peer review only - http://bmjopen.bmj.com/site/about/guidelines.xhtml

III. Data accrued from providers

Table S3 Numbers (%) of mothers and children with data returned by dataset

	Mothers	Children
**Sent for matching to DfE and NHSD**	**N=1537**	**N=1547**
**Department for Education (DfE)**		
Not matched	1248 (81·2)	40 (2·6)
Match to any DfE dataset	289 (18·8)	1507 (97·4)
Pupil Level Annual School Census (PLASC)	<5	1498 (96·8)
Child in Need (CIN)	278 (18·1)	414 (26·8)
Children looked after (CLA)	61 (4·0)	52 (3·4)
Early years foundation stage profile (EYFSP)	N/A	1476 (95·4)
Key Stage one (KS1)	N/A	1472 (95·2)
Early Years Census (EYC)	N/A	642 (41·5)
Pupil referral unit (PRU) Census	<5	0
Alternative Provision (AP)	<5	<5
Absence	<5	1494 (96·6)
**NHS Digital (NHSD)**		
Not matched	36 (2·3)	27 (1·7)
Duplicate match	0	1 (0·06)
Matched to any NHSD dataset	1501 (97·7)	1519 (98·3)
Accident and Emergency (A&E)	1360 (88·5)	1392 (90·0)
Admitted Patient Care (Inpatients) (APC)	1498 (97·5)	1507 (97·4)
Outpatient (OP)	1495 (97·3)	1189 (76·9)
**Sent for matching to Department of Health and Social Care (DHSC)**	**N=1532**	**0**
Abortions dataset	1532 (100)	NA

For peer review only - http://bmjopen.bmj.com/site/about/guidelines.xhtml

IV. Further details of Child in Need outcome.

In our primary comparative analysis we examined whether the firstborn ('BB:0-2 child') had ever been referred to social services and classed as a child in need at any point between birth and 6 years of age. There is no flag in the Child In Need dataset to identify whether a child is in need after they have been referred to social services. Therefore, CIN was defined (as per DfE methodology)[1] as all children referred to social services, excluding cases where:

- the referral is flagged as being a referral resulting in no further action; or
- the only activity recorded is an assessment and the reason for the closure of the case being that case was closed after assessment with no further action.

1. Department for Education. Characteristics of children in need in England: 2014 to 2015. 2015. [accessed 2019 Nov 12]. Available from: https://www.gov.uk/government/uploads/system/uploads/attachment_data/file/469740/SFR41-2015_Methodology.pdf

V. Representativeness of study sample

Scope and purpose: We assessed whether the study sample for BB:2-6 were similar to women recruited to the original BB:0-2 trial.
Conclusion: The BB:2-6 and BB:0-2 cohorts are similar in terms of maternal (Table S4) and baby characteristics (Table S5).

Table S4 Maternal characteristics of those in BB:0-2 compared to those in the BB:2-6

	**BB:0-2 N=1618**	**BB:2-6 N=1537**
**Minimisation variables at randomisation**		
**Gestational age at baseline** n(%)		
16 weeks or more	658 (40·7)	636 (41·4)
Less than 16 weeks	959 (59·3)	900 (58·6)
**Smoking status** n(%)		
No	860 (53·2)	822 (53·5)
Yes	758 (46·8)	715 (46·5)
**Language** n(%)		
English	1611 (99·6)	1530 (99·5)
Other	6 (0·4)	6 (0·4)
Missing	*1 (0·0)*	*0 (0·0)*

For peer review only - http://bmjopen.bmj.com/site/about/guidelines.xhtml

	BB:0-2 N=1618	BB:2-6 N=1537
**Trial arm** n(%)		
FNP	808 (49·9)	766 (49·8)
Usual care	810 (50·1)	771 (50·2)
**Age at recruitment (years) Median (25th to 75th centile)**	17·9 (16·9 to 18·8)	17·9 (16·9 to 18·8)
**Ethnicity** n(%)		
White background	1425 (88·1)	1355 (88·2)
Mixed background	89 (5·5)	82 (5·3)
Asian background	27 (1·7)	25 (1·6)
Black background	71 (4·4)	69 (4·5)
Chinese or Other background	6 (0·4)	6 (0·4)
**Relationship status with baby's father** n(%)		
Married	20 (1·2)	16 (1·0)
Separated	165 (10·2)	150 (10·0)
Closely involved/boyfriend	1222 (75·5)	1168 (76·0)
Just friends	211 (13·0)	203 (13·2)
**Live with father of baby** n(%)		
Yes	368 (22·7)	348 (22·6)
No	1112 (68·7)	1061 (69·0)
Missing	138 (8·5)	128 (8·3)
**NEET status[a]** n(%)		
Yes	663 (41·0)	634 (41·2)
No	717 (44·3)	678 (44·1)
Missing	*4 (0·2)*	*4 (0·3)*
Not applicable (aged 16 years or younger)	*234 (14·5)*	*221 (14·4)*
**Socio-economic status: Overall Index of Multiple Deprivation (IMD) Score[b]** Median (25th to 75th centile)	N=1606<br>38·2 (25·2 to 52·0)	N=1525<br>38·1 (25·0 to 52·1)
**Maternal health and wellbeing**		
**Generalized self-efficacy scale (score 10 to 40)[c]** Median (25th to 75th centile)	N=1592<br>30·0 (28·0 to 33·0)	N=1533<br>30·0 (28·0 to 33·0)
**Adaptive functioning: Difficulty in at least one basic skill** n(%)		
Yes	430 (26·6)	397 (25·8)
No	1185 (73·2)	1138 (74·0)
Missing	*3 (0·2)*	*2 (0·1)*
**Adaptive functioning: Had 3 or less life skills** n(%)		
Yes	434 (26·8)	403 (26·2)
No	1178 (72·8)	1129 (73·5)
Missing	*6 (0·4)*	*5 (0·3)*
**Adaptive functioning: At least one burden** n(%)		
Yes	476 (29·4)	451 (29·3)
No	1131 (69·9)	1075 (69·9)
Missing	*11 (0·7)*	*11 (0·7)*
**Smoking** (Participant self-reported) n(%)		
Ever smoked: Yes	1291 (79·8)	1227 (79·8)
No	327 (20·0)	310 (20·2)
Missing	*0 (0)*	*0 (0)*
**Child characteristics**	**BB:0-2 sample N=1510**	**BB:2-6 sample N=1547**
**Trial arm** n(%)		

For peer review only - http://bmjopen.bmj.com/site/about/guidelines.xhtml

	BB:0-2 N=1618	BB:2-6 N=1537
FNP	742 (49·1)	773 (50·0)
Usual Care	768 (50·9)	774 (50·0)
**Gender** n(%)		
Male	777 (51·5)	787 (50·9)
Female	732 (48·5)	758 (40·1)
Missing	1	72[d]
**Birthweight (grams) Mean (sd)**	3207·3 (599·6)	3219·6 (580·7)
Missing[c]		71
**Neonatal unit (NNU) admission** n(%)		
Yes	149 (10·0)	142 (9·2)
Missing from data collection	13	11
Missing	Not applicable	71[d]

[a] *Definition of NEET status: Not in education employment or training (applicable only to those whose age at end of previous academic year at time of baseline interview was >16)*
[b] *Higher IMD score indicated more deprivation. Mean IMD Score for England in 2010 was 21·67* [60]*;*
[c] *Higher score indicates higher level of self-efficacy*
[d] *Mothers were withdrawn in BB:0-2 but are included in BB:2-6*

Table S5 Child characteristics of those in the BB:0-2 study compared to those in the BB:2-6 study

	BB:0-2 sample N=1510	BB:2-6 sample N=1547
**Trial arm** n(%)		
FNP	742 (49·1)	773 (50·0)
Usual Care	768 (50·9)	774 (50·0)
**Gender** n(%)		
Male	777 (51·5)	787 (50·9)
Female	732 (48·5)	758 (40·1)
Missing	1	72[a]
**Birthweight (grams) Mean (sd)**	3207·3 (599·6)	3219·6 (580·7)
Missing[a]		71
**Neonatal unit (NNU) admission** n(%)		
Yes	149 (10·0)	142 (9·2)
Missing from data collection	13	11
Missing[a]	Not applicable	71*

[a] *Mothers were withdrawn in BB:0-2 but are included in BB:2-6*

For peer review only

VI. Descriptive secondary outcomes and additional Key Stage 1 results

Table S6 Child In Need referrals, Children's Social Services referrals and hospital admissions: descriptives

Outcome	FNP	Usual care (UC)
**Child in Need (CIN) Referrals**	***N=161***	***N=162***
Age at first CIN referral (days)		
Pre birth	<5	<5
Birth to <1 years	<5	<5
1 to <2 years	6 (3·7%)	6 (3·7%)
2 to <3 years	15 (9·4%)	28 (17·3%)
3 to <4 years	27 (16·9%)	23 (14·2%)
4 to <5 years	47 (29·4%)	39 (24·1%)
5 to <6 years	33 (20·6%)	41 (25·3%)
6 to <7 years	20 (12·5%)	20 (12·3%)
Missing	*1*	*0*
Unique* number of CIN referrals per child		
1 case	110 (68·3%)	110 (67·9%)
2 cases	42 (26·1%)	42 (25·9%)
3 or more cases	9 (5·5%)	10 (6·1%)
CIN cases closed at 31st March 2017	116 (72·0%)	110 (67·9%)
**Referral to Children's social care services (CSCS)**	***N=760***	***N=746***
Total referrals made N	339	354
Ratio referrals:children	1·65	1·70
Referrals made per child	***N=206***	***N=208***
1	159 (77·2%)	140 (67·3%)
2	34 (16·5%)	51 (24·5%)
3 to 5	13 (6·3%)	27 (8·2%)
**Unit=referral (referred to CSCS encompass any referral)**	***n=339***	***n=354***
Source of referral		
Individual	29 (8·8%)	34 (10·0%)
School/Education	150 (45·7%)	136 (40·0%)
Health services	114 (34·8%)	132 (38·8%)
Local Authority Services	35 (10·7%)	38 (11·2%)
Missing	*11*	*14*
Primary need of referrals		
Abuse or neglect	198 (58·9%)	205 (57·9%)
Family dysfunction	72 (21·4%)	88 (24·9%)
Family in acute stress	19 (5·7%)	21 (5·9%)
Low income/absent parenting/ socially unacceptable behaviour	11 (3·3%)	7 (2·0%)
Parental/Childs disability illness	14 (4·2%)	11 (3·1%)
Cases other than Child in Need	11 (2·3%)	12 (3·4%)
Not stated	11 (3·3%)	9 (2·5%)
Missing	*3*	*1*
Legal status of child looked after†	***N=17***	***N=23***
Interim care order	6 (35·3%)	8 (34·8%)

For peer review only - http://bmjopen.bmj.com/site/about/guidelines.xhtml

Outcome	FNP	Usual care (UC)
Full care order	5 (29·4%)	6 (26·1%)
Placement order granted	<5	<5
Single period of accommodation under section 20	<5	<5
**Injuries and Ingestions: Admission to hospital**	**N=760**	**N=759**
N unique admissions	109	119
Ratio admissions: children	1.22	1.20
Length of hospital stay (days)	0·5‡ (0·5 to 1·0)	0·5 (0·5 to 1·0)
	**N=760**	**N=746**
Death	<10	

Data are n (%), n/N (%), or median (25th to 75th centile) unless otherwise stated. <5 = numbers suppressed· FNP = Family Nurse Partnership Programme + Usual Care. * A unique referral is counted as a distinct referral date per child. † Legal status for most recent CLA episode (Of n=52 children with a recent CLA episode, n=12 cases have a missing status). ‡ 0·5 days indicates an admission and discharge on the same day (could be up to 1 day in hospital). Source: National Pupil Database, The Department for Education

Table S7 Emergency attendance to hospital by injury or ingestion, by trial arm

	**Trial arm, n (%)**	
**Injury / ingestion**	**FNP (N = 911)**	**Usual Care (N = 881)**
Laceration	136 (14.9)	158 (17.9)
Contusion/abrasion	90 (9.9)	96 (10.9)
Soft-tissue inflammation	63 (6.9)	64 (7.3)
Head injury	68 (29.4)	249 (28.3)
Dislocation	109 (12.0)	84 (9.5)
Sprain/muscle/tendon injury	35 (3.8)	33 (3.8)
Nerve/vascular injury	74 (8.1)	60 (6.8)
Burns and scalds/electric shock	48 (5.3)	37 (4.2)
Foreign body	40 (4.4) 4	6 (5.2)
Bites and stings	12 (1.3)	20 (2.3)
Poisoning	36 (4.0)	34 (3.9)

For peer review only - http://bmjopen.bmj.com/site/about/guidelines.xhtml

Table S8 Admissions by injury/ingestion type and trial arm

	Trial arm, n (%)	
**Diagnosis ICD-10 code**	**FNP**<br><br>**(N = 109)**	**Usual care**<br><br>**(N = 119)**
S00–S09 Injuries to the head	52 (47.7)	61 (51.3)
S20–S29 Injuries to the thorax	9 (8.3)	6 (5.0)
S30–S39 Injuries to the abdomen, lower back, lumbar spine and pelvis		
S40–S49 Injuries to the shoulder and upper arm		
S50–S59 Injuries to the elbow and forearm		
S60–S69 Injuries to the wrist and hand	8 (7.3)	5 (4.2)
S70–S79 Injuries to the hip and thigh	11 (10.1)	11 (9.2)
S80–S89 Injuries to the knee and lower leg		
S90–S99 Injuries to the ankle and foot		
T00–T07 Injuries involving multiple body regions	5 (4.6)	10 (8.4)
T08–T14 Injuries to unspecified part of trunk, limb or body region		
T20–T32 Burns and corrosions		
T15–T19 Effects of foreign body entering through natural orifice	5 (4.6)	8 (6.7)
T36–T50 Poisoning by drugs, medicaments and biological substances	11 (10.1)	8 (6.7)
T51–T65 Toxic effects of substances chiefly non-medicinal as to source (sting, alcohol, solvents etc.)	8 (7.3)	10 (8.4)
T66–T78 Other and unspecified effects of external causes		

For peer review only

For peer review only - http://bmjopen.bmj.com/site/about/guidelines.xhtml

Table S9. Hospital admissions for injuries and ingestions by trial arm and broken down by age categories

	**Intervention N=760**	**Usual care N=759**
No hospital admissions with an injury or an ingestion (0-6 years)	671 (88.3%)	660 (87.0%)
At least one hospital admission with an injury or an ingestion	89 (11.7%)	99 (13.0%)
By age at admission		
**Under 1 year**		
Children with no admission n(%)	744 (97.9%)	732 (96.4%)
Children with at least one admission n(%)	16 (2.1%)	27 (3.6%)
95% CI	1.3 to 3.3%	2.5 to 5.1%
Total number of admissions (different days)	20	29
Ratio of admissions: child	1.25	1.08
	n=20	n=29
Length of stay (days) Median (25th to 75th centiles)	0.75 (0.5 to 2)	0.5 (0.5 to 2)
**1 to under 2 years**		
Children with no admission n(%)	738 (97.1%)	725 (95.5%)
Children with at least one admission n(%)	21 (2.8%)	31 (4.1%)
95% CI	1.8 to 4.2%	2.9 to 5.7%
Total number of admissions (different days)	22	34
Ratio of admissions: child	1.05	1.10
	n=21*	n=34
Length of stay (days) Median (25th to 75th centiles)	0.5 (0.5 to 1)	0.5 (0.5 to 1)
**25 months to under 6 years**		
Children with no admission n(%)	699 (92.0%)	709 (93.4%)
Children with at least one admission n(%)	61 (8.0%)	50 (6.6%)
95% CI	6.3 to 10.2%	5.0 to 8.6%
Total number of admissions (different days)	67	56
Ratio of admissions: child	1.10	1.12
	n=65*	n=56
Length of stay (days) Median (25th to 75th centiles)	0.5 (0.5 to 1)	0.5 (0.5 to 1)

* Date of discharge missing – LOS cannot be calculated
LOS=0.5 days indicates an admission and discharge on the same day (could be up to 1 day in hospital).

For peer review only

Table S10 Child health, developmental and educational: descriptives

Outcome	FNP	Usual care	Absolute risk difference (FNP-Usual care) (95% CI)
**Early educational attendance**	**N=759**	**N=747**	
Children attending education* up to the age of 4 years			
Yes†	758 (99·7%)	747 (100·0%)	-0·3% (-1·0 to 0·3%)
Children attending school up to the age of:			
• 4 years†	753 (99·1%)	744 (99·6%)	-0·5% (-1·5 to 0·4%)
• 3 years	479 (63·0%)	482 (64·5%)	
• 2 years	137 (18·0%)	147 (19·7%)	
Earliest type of day care	***N=334***	***N=308***	
Private	217 (65·0%)	221 (71·8%)	
Voluntary	52 (15·6%)	57 (18·5%)	
Local Authority Day Nursery	36 (10·8%)	22 (7·1%)	
Other‡	29 (8·7%)	8 (2·6%)	
**School attendance**	**N=754**	**N=740**	
Number of academic sessions§	630 (264 to 638)	630 (262 to 638)	
Overall absence rate Median (25th to 75th centiles)	5·08 (2·69 to 8·73)	5·19 (2·73 to 8·41)	
Overall authorised absence rate Median (25th to 75th centiles)	3·50 (1·96 to 6·09)	3·79 (2·02 to 6·11)	
Overall unauthorised absence rate Median (25th to 75th centiles)	1·84 (0·76 to 4·34)	1·57 (0·63 to 3·88)	
**Early Years assessment**	**N=743**	**N=728**	
Achieving good level of development (GLD) ‖:			
• Communication and Language	642 (86·4%)	613 (84·2%)	2·2% (-1·4 to 5·8%)
• Physical Development	676 (91·0%)	643 (88·3%)	2·7% (-0·5 to 5·8%)

For peer review only

Outcome	FNP	Usual care	Absolute risk difference (FNP-Usual care) (95% CI)
• Personal, Social and Emotional Development	668 (89·9%)	634 (87·1%)	2·8% (-0·4 to 6·1%)
• Literacy	493 (66·4%)	458 (62·9%)	3·4% (-1·4 to 8·3%)
• Maths	545 (73·4%)	516 (70·9%)	2·5% (-2·1 to 7·0%)
Total point score¶			
• Score <34	325 (43·8%)	345 (47·4%)	
• Score = 34	187 (25·2%)	183 (25·1%)	
• Score >34	231 (31·1%)	200 (27·5%)	
**Key Stage 1 assessments**			
Writing academic year 2017/18	**N=242**	**N=245**	
Lower than expected	82 (33·9%)	81 (33·1%)	
Reaching at least the expected standard**	160 (66·1%)	164 (66·9%)	-0·8 (-9·2 to 7·5)
Expected standard	140 (57·9%)	132 (53·9%)	4·0 (-4·8 to 12·7)
Higher standard	20 (8·3%)	32 (13·1%)	-4·8 (-10·4 to 0·7)

Data are n (%), n/N (%), or median (25th to 75th centile) unless otherwise stated. FNP = Family Nurse Partnership Programme + Usual Care.

* School or an Ofsted registered Private, Voluntary or Independent (PVI) establishment;

† = Not estimable; ‡ Childminding Network, Childminder, Registered Independent School, Other; § Total number of sessions over the academic years available (2013 to Spring 2017) and all 6 terms per academic year. ‖ Children achieving a good level of development are those achieving at least the expected level within the prime and specific areas of learning; ¶ Total point score ranges from 17 to 51 with a higher score indicating a better level of development. ** Working at the expected standard and at a greater depth within the expected standard. PLASC (Autumn, Spring and Summer terms), AP, PRU Census, and EYC. If for any of the years no response was recorded then the assumption was that the child was not included in the denominator as either not present on Census day or not in school. Source: National Pupil Database, The Department for Education

For peer review only - http://bmjopen.bmj.com/site/about/guidelines.xhtml

Table S11 Children reaching the expected standard in KS1 assessments by trial arm and academic year

		Academic year 2016/17	Academic year 2017/18
Reading	FNP	302/498 (60.6%)	181/242 (74.8%)
	Usual care	263/487 (54.0%)	180/245 (73.5%)
Maths	FNP	280/498 (56.2%)	179/242 (74.0%)
	Usual care	263/487 (54.0%)	186/245 (75.9%)
Science	FNP	341/498 (68.5%)	196/242 (81.0%)
	Usual care	310/487 (63.7%)	203/245 (82.9%)

Source: National Pupil Database, The Department for Education

Figure S1 Distribution of children's month of birth by trial arm

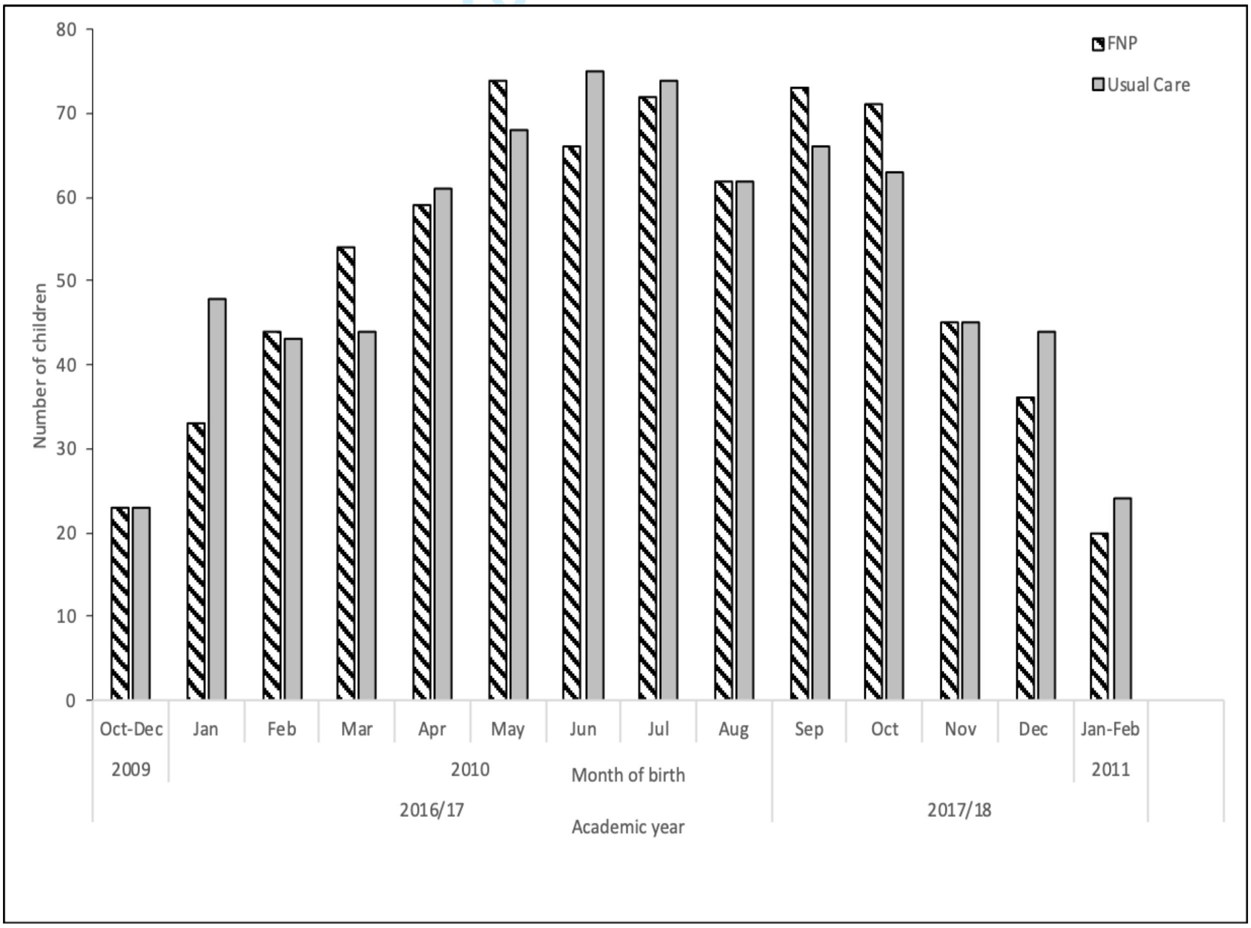

For peer review only - http://bmjopen.bmj.com/site/about/guidelines.xhtml

Table S12 Comparison of parameter estimates before and after adjustment for child's month[a] of birth

	Odds ratio[b] (95% CI), p-values	
	**Adjusted for site and minimisation variables[c]**	**Adjusted for site, minimisation variables[c] and month of birth**
**Key Stage 1 outcome**		
**Reading**		
Reaching at least the expected standard	1.23 (0.99 to 1.53), 0.051	1.26 (1.02 to 1.57), 0.035
Expected standard	*1.24 (0.99 to 1.56), 0.056*	*1.27 (1.01 to 1.59), 0.042*
Higher standard	*1.20 (0.88 to 1.65), 0.250*	*1.26 (0.91 to 1.75), 0.161*
**Maths**		
Reaching at least the expected standard	1.04 (0.84 to 1.28), 0.731	1.06 (0.85 to 1.31), 0.613
Expected standard	*1.06 (0.85 to 1.32), 0.611*	*1.07 (0.86 to 1.34), 0.527*
Higher standard	*0.93 (0.64 to 1.35), 0.711*	*0.93 (0.74 to 1.17), 0.522*
**Science**		
Reaching at least the expected standard	1.14 (0.91 to 1.43), 0.254	1.16 (0.92 to 1.47), 0.197
**Writing academic year 2017/18**		
Reaching at least the expected standard	1.24 (0.97 to 1.60), 0.090	1.26 (0.98 to 1.62), 0.076
Expected standard	*1.29 (1.00 to 1.68), 0.054*	*1.30 (1.00 to 1.69), 0.050*
Higher standard	*0.92 (0.51 to 1.64), 0.769*	*0.97 (0.54 to 1.75), 0.917*
**Early Years Assessment**		
Achieving GLD in all five areas of learning[d]	1.26 (1.03 to 1.55), 0.026	1.31 (1.05 to 1.62), 0.015
Achieving GLD in all 17 early learning goals	1.24 (1.01 to 1.52), 0.043	1.27 (1.03 to 1.58), 0.027
Total point score	0.65 (-0.11 to 1.41), 0.094	0.70 (-0.03 to 1.42), 0.060

a Quarter of birth runs from September to November, December to February, March to May, June to August; b Odds ratio represents the FNP arm compared to Usual care; c gestational age, smoking status at recruitment, and first or preferred language; d Children achieving a good level of development (GLD) are those achieving at least the expected level within the prime and specific areas of learning.

Source: National Pupil Database, The Department for Education

For peer review only

VII. Results of planned sub-group analysis

No differential effects due to maternal age, deprivation, participation in employment, education or training, or basic life skills were found in planned sub-group analyses for any of the objective maltreatment outcomes (Table S10 and Table S11). The beneficial impact of FNP on EYFSP total point score was mostly observed for children with mothers aged <16 years rather than those aged ≥16 at study entry but no other differences by study sub-group were found (Table S12).

The differential effects due to maternal age, deprivation, participation in employment, education or training, or basic life skills for KS1 reading, maths and science are displayed in Tables S13a to e and for writing in Table S14. At KS1, sex differences were large with girls outperforming boys in all four subject areas (Table S13a). The intervention effect was greater for boys than girls with a higher proportion reaching the expected standard in the FNP arm, particularly for reading and writing (and strengthened after adjusting for month of birth) and for the writing assessment (academic year 2016/17) this interaction reached statistical significance after adjusting for month of birth (post-hoc analysis). When examining how the intervention effects differed in mothers aged less than 16 years at recruitment compared to mothers aged 16 years or more in each KS1 assessment, differences were observed in maths (Table S13b) and writing (Table S14), with a larger effect seen in mothers aged less than 16 at recruitment, when compared to those aged 16 or more.

Table S13 Subgroup analyses for referrals to Children's social care services

N(%) referred	FNP	Usual care	Adjusted[a] OR (95% CI), p-value	Interaction p-value
**Child Gender**				
Boys	99 (26.3)	108 (27.7)	0.93 (0.65 to 1.33), 0.69	0.78
Girls	107 (27.9)	100 (28.1)	1.01 (0.69 to 1.48), 0.94	
**Maternal age at recruitment**				
<16 years	17 (34.7)	19 (34.6)	1.05 (0.44 to 2.46), 0.92	0.96
≥16 years	189 (26.6)	189 (27.4)	0.96 (0.72 to 1.30), 0.81	
**Adaptive functioning**				
No difficulty	140 (25.4)	149 (26.3)	0.99 (0.71 to 1.38), 0.96	0.67
Difficulty in basic skills	66 (31.7)	59 (33.0)	0.81 (0.59 to 1.41), 0.67	
**NEET status**				
EET	70 (20.3)	75 (23.1)	0.83 (0.54 to 1.29), 0.42	0.18
NEET	100 (32.5)	89 (29.2)	1.19 (0.80 to 1.78), 0.39	
**Deprivation quintile**				

For peer review only - http://bmjopen.bmj.com/site/about/guidelines.xhtml

1 – least deprivation	33 (20.4)	34 (23.9)	0.77 (0.44 to 1.36), 0.37	0.32
2	36 (23.8)	30 (20.5)	1.26 (0.61 to 2.60), 0.53	
3	42 (28.8)	43 (27.9)	0.96 (0.50 to 1.86), 0.91	
4	41 (31.1)	39 (25.3)	1.27 (0.69 to 2.31), 0.44	
5 – most deprivation	54 (33.3)	61 (42.4)	0.65 (0.35 to 1.22), 0.18	

[a] FNP compared to usual care. Analysis adjusted for stratification (site), minimisation variables (gestational age, smoking status at recruitment, and first or preferred language). N/EET – Not/In Education, Employment or Training (Source: National Pupil Database, The Department for Education)

Table S14 Subgroup analyses for child in need status

N(%) CIN	FNP	Usual care	Adjusted[a] OR (95% CI), p-value	Interaction p-value
**Gender**				
Boys	80 (21.3)	80 (20.5)	1.05 (0.72 to 1.53), 0.81	0.56
Girls	81 (21.1)	82 (23.0)	0.91 (0.60 to 1.38), 0.65	
**Maternal age at recruitment**				
<16 years	14 (28.6)	16 (29.1)	1.00 (0.41 to 2.43), 0.99	0.98
≥16 years	147 (20.7)	146 (21.1)	0.98 (0.72 to 1.33), 0.88	
**Adaptive functioning**				
No difficulty	106 (19.2)	113 (20.0)	0.99 (0.70 to 1.42), 0.98	0.76
Difficulty in basic skills	55 (26.4)	49 (27.4)	0.93 (0.59 to 1.47), 0.76	
**NEET**				
EET	52 (16.0)	52 (15.1)	0.93 (0.57 to 1.53), 0.78	0.42
NEET	81 (26.3)	72 (23.6)	1.18 (0.77 to 1.77), 0.46	
**Deprivation quintile**				
1 – least deprivation	24 (14.8)	22 (15.5)	0.92 (0.49 to 1.73), 0.79	0.62
2	30 (19.9)	22 (15.1)	1.53 (0.68 to 3.47), 0.31	
3	32 (21.9)	36 (23,4)	0.89 (0.52 to 1.54), 0.68	
4	32 (24.2)	34 (22.1)	1.15 (0.64 to 2.06), 0.64	
5 – most deprivation	43 (26.5)	48 (33.3)	0.72 (0.38 to 1.38), 0.32	

[a] FNP compared to usual care. Analysis adjusted for stratification (site), minimisation variables (gestational age, smoking status at recruitment, and first or preferred language). N/EET – Not/In Education, Employment or Training (Source: National Pupil Database, The Department for Education).

For peer review only - http://bmjopen.bmj.com/site/about/guidelines.xhtml

Table S15 Subgroup analyses for Early Years Assessment total point score

Mean (SD) total point score	FNP	Usual care	Adjusted[a] mean difference (95% CI), p-value	Interaction p-value
**Gender**				
Boys	N=369<br>30.57 (7.33)	N=383<br>29.84 (7.56)	0.75 (-0.31 to 1.82), 0.17	0.57
Girls	N=374<br>33.85 (6.79)	N=345<br>33.53 (7.22)	0.34 (-0.67 to 1.35), 0.51	
**Maternal age at recruitment**				
<16 years	N=45<br>32.44 (6.71)	N=52<br>28.91 (7.70)	3.65 (0.76 to 6.55), 0.013	0.046
≥16 years	N= 698<br>32.21 (7.28)	N= 676<br>31.80 (7.58)	0.42 (-0.36 to 1.21), 0.29	
**Adaptive functioning**				
No difficulty	N=540<br>32.99 (7.12)	N=552<br>32.11 (7.65)	0.88 (0.003 to 1.75), 0.049	0.50
Difficulty in basic skills	N=203<br>30.18 (7.20)	N=175<br>29.94 (7.33)	0.34 (-1.11 to 1.79), 0.65	
**NEET status**				
In EET	N=339<br>32.45 (7.12)	N=322<br>32.37 (7.68)	0.15 (-0.99 to 1.28), 0.80	0.95
NEET	N= 304<br>31.72 (7.57)	N=299<br>31.50 (7.43)	0.19 (-1.00 to 1.38), 0.76	
**Index of Multiple Deprivation quintiles**				
1 = least deprived	N=159<br>33.16 (7.44)	N=139<br>31.53 (8.24)	1.72 (-0.07 to 3.50), 0.060	ref
2	N=148<br>32.16 (6.97)	N=144<br>31.59 (7.05)	0.65 (-0.96 to 2.27), 0.43	0.84
3	N=146<br>32.16 (6.79)	N=151<br>32.59 (7.27)	-0.20 (-1.79 to 1.39), 0.80	0.21
4	N=128<br>30.95 (7.53)	N=148<br>31.11 (7.50)	-0.31 (-2.11 to 1.48), 0.73	0.85
5 = most deprived	N=156<br>32.41 (7.46)	N=140<br>31.03 (8.12)	1.45 (-0.33 to 3.22), 0.11	0.74

[a] FNP compared to usual care. Analysis adjusted for stratification (site), minimisation variables (gestational age, smoking status at recruitment, and first or preferred language). N/EET – Not/In Education, Employment or Training (Source: National Pupil Database, The Department for Education).

Table S16 Subgroup analyses for KS1 assessments reaching the expected standard (RES)

(a) Gender

N(%)	Boys		Girls	
	Not RES	RES	Not RES	RES
**Reading**				
FNP	152 (41.8)	212 (58.2)	105 (27.9)	271 (72.1)
Usual care	182 (47.3)	203 (52.7)	107 (30.8)	240 (69.2)
Adjusted[a] OR (95% CI), p-value	1.27 (0.95 to 1.69), 0.11		1.16 (0.84 to 1.59), 0.38	
Interaction p-value=0.68				

For peer review only - http://bmjopen.bmj.com/site/about/guidelines.xhtml

Adjusted[b] OR (95% CI), p-value	1.30 (0.96 to 1.75), 0.09		1.17 (0.84 to 1.62), 0.36	
Interaction p-value=0.68				
**Maths**				
FNP	149 (40.9)	215 (59.1)	132 (35.1)	244 (64.9)
Usual care	166 (43.1)	219 (56.9)	117 (33.7)	230 (66.3)
Adjusted[a] OR (95% CI), p-value	1.11 (0.83 to 1.49), 0.49		0.95 (0.69 to 1.29), 0.72*	
Interaction p-value=0.46				
Adjusted[b] OR (95% CI), p-value	1.14 (0.84 to 1.54), 0.40		0.94 (0.69 to 1.29), 0.71	
Interaction p-value=0.47				
**Science**				
FNP	120 (33.0)	244 (67.0)	83 (22.1)	293 (77.9)
Usual care	138 (35.8)	247 (64.2)	81 (23.3)	266 (76.7)
Adjusted[a] OR (95% CI), p-value	1.16 (0.86 to 1.58), 0.34		1.08 (0.76 to 1.53), 0.68	
Interaction p-value=0.77				
Adjusted[b] OR (95% CI), p-value	1.18 (0.87 to 1.63), 0.28		1.08 (0.76 to 1.55), 0.66	
Interaction p-value=0.72				

RES = Reaching the expected standard; [a] Analysis adjusted for stratification (site), minimisation variables (gestational age, smoking status at recruitment, and first or preferred language); [b] Analysis adjusted for stratification (site), minimisation variables (gestational age, smoking status at recruitment, and first or preferred language) and month of birth. (Source: National Pupil Database, The Department for Education).

(b) Maternal age at recruitment

N(%)	Maternal age<16 years		Maternal age≥16 years	
	**Not RES**	**RES**	**Not RES**	**RES**
**Reading**				
FNP	21 (44.7)	26 (55.3)	236 (34.1)	457 (65.9)
Usual care	29 (52.7)	26 (47.3)	260 (38.4)	417 (61.6)
Adjusted[a] OR (95% CI), p-value	1.21 (0.97 to 1.52), 0.09		1.49 (0.65 to 3.41), 0.34	
Interaction p-value=p=0.66				
Adjusted[b] OR (95% CI), p-value	1.50 (0.65 to 3.48), 0.34		1.25 (0.99 to 1.57), 0.07	
Interaction p-value=0.62				
**Maths**				
FNP	16 (34.0)	31 (66.0)	265 (38.2)	428 (61.8)
Usual care	32 (58.2)	23 (41.8)	251 (37.1)	426 (62.9)
Adjusted[a] OR (95% CI), p-value	3.15 (0.94 to 10.48), 0.06		0.96 (0.77 to 1.19), 0.69	
Interaction p-value=0.011				

For peer review only - http://bmjopen.bmj.com/site/about/guidelines.xhtml

Adjusted[b] OR (95% CI), p-value	3.23 (1.36 to 7.67), 0.008		0.98 (0.78 to 1.22), 0.83	
Interaction p-value=0.014				
**Science**				
FNP	18 (38.3)	29 (61.7)	185 (26.7)	508 (73.3)
Usual care	25 (45.4)	30 (54.6)	194 (28.7)	483 (71.3)
Adjusted[a] OR (95% CI), p-value	1.54 (0.53 to 4.47), 0.42		1.11 (0.88 to 1.41), 0.39	
Interaction p-value=0.56				
Adjusted[b] OR (95% CI), p-value	1.37 (0.59 to 3.19), 0.467		1.14 (0.89 to 1.45), 0.29	
Interaction p-value=0.69				

RES = Reaching the expected standard; [a] FNP compared to Usual care. Analysis adjusted for stratification (site), minimisation variables (gestational age, smoking status at recruitment, and first or preferred language); [b] Analysis adjusted for stratification (site), minimisation variables (gestational age, smoking status at recruitment, and first or preferred language) and month of birth. (Source: National Pupil Database, The Department for Education).

(c) Adaptive functioning

N(%)	No difficulty with basic skills		Difficulty with basic skills	
	Not RES	RES	Not RES	RES
**Reading**				
FNP	152 (30.5)	347 (69.5)	87 (42.9)	116 (57.1)
Usual care	188 (36.2)	332 (63.8)	84 (48.0)	91 (52.0)
Adjusted[a] OR (95% CI), p-value	1.29 (0.98 to 1.70), 0.07		1.23 (0.82 to 1.86), 0.32	
Interaction p-value=0.61				
Adjusted[b] OR (95% CI), p-value	1.29 (0.98 to 1.69), 0.07		1.25 (0.83 to 1.91), 0.29	
Interaction p-value=0.95				
**Maths**				
FNP	186 (34.6)	351 (65.4)	95 (46.8)	108 (53.2)
Usual care	192 (34.5)	364 (65.5)	91 (52.0)	84 (48.0)
Adjusted[a] OR (95% CI), p-value	1.01 (0.78 to 1.29), 0.97		1.27 (0.84 to 1.93), 0.26	
Interaction p-value=0.38				
Adjusted[b] OR (95% CI), p-value	1.01 (0.78 to 1.31), 0.93		1.32 (0.86 to 2.03), 0.20	
Interaction p-value=0.28				
**Science**				
FNP	130 (24.2)	407 (75.8)	73 (36.0)	130 (64.0)
Usual care	153 (27.5)	403 (72.5)	66 (37.7)	109 (62.3)
Adjusted[a] OR (95% CI), p-value	1.20 (0.92 to 1.58), 0.19		1.10 (0.71 to 1.69), 0.68	
Interaction p-value=0.71				

For peer review only - http://bmjopen.bmj.com/site/about/guidelines.xhtml

Adjusted[b] OR (95% CI), p-value	1.22 (0.93 to 1.62), 0.16	1.13 (0.73 to 1.74), 0.59
Interaction p-value=0.82		

RES = Reaching the expected standard; [a] FNP compared to Usual care. Analysis adjusted for stratification (site), minimisation variables (gestational age, smoking status at recruitment, and first or preferred language); [b] Analysis adjusted for stratification (site), minimisation variables (gestational age, smoking status at recruitment, and first or preferred language) and month of birth. (Source: National Pupil Database, The Department for Education).

(d) Not in Education, employment or training (NEET) status

N(%)	NEET		In EET	
	**Not RES**	**RES**	**Not RES**	**RES**
**Reading**				
FNP	105 (31.2)	232 (68.8)	109 (36.0)	194 (64.0)
Usual care	115 (35.8)	206 (61.2)	121 (40.5)	178 (59.5)
Adjusted[a] OR (95% CI), p-value	1.26 (0.91 to 1.75), 0.17		1.21 (0.87 to 1.69), 0.26	
Interaction p-value=0.86				
Adjusted[b] OR (95% CI), p-value	1.31 (0.94 to 1.83), 0.17		1.25 (0.89 to 1.76), 0.19	
Interaction p-value=0.83				
**Maths**				
FNP	122 (36.2)	215 (63.8)	122 (40.3)	181 (59.7)
Usual care	107 (33.3)	214 (66.7)	120 (40.1)	179 (59.9)
Adjusted[a] OR (95% CI), p-value	0.89 (0.65 to 1.23), 0.48		0.99 (0.72 to 1.38), 0.97	
Interaction p-value=0.64				
Adjusted[b] OR (95% CI), p-value	0.93 (0.66 to 1.29), 0.65		1.03 (0.73 to 1.42), 0.90	
Interaction p-value=0.68				
**Science**				
FNP	87 (25.8)	250 (74.2)	821 (27.1)	221 (72.9)
Usual care	78 (24.3)	243 (75.7)	98 (32.8)	201 (67.2)
Adjusted[a] OR (95% CI), p-value	0.93 (0.65 to 1.33), 0.69		1.31 (0.92 to 1.87), 0.14	
Interaction p-value=0.20				
Adjusted[b] OR (95% CI), p-value	0.96 (0.67 to 1.39), 0.84		1.36 (0.95 to 1.95), 0.10	
Interaction p-value=0.22				

RES = Reaching the expected standard; [a] FNP compared to Usual care. Analysis adjusted for stratification (site), minimisation variables (gestational age, smoking status at recruitment, and first or preferred language); [b] Analysis adjusted for stratification (site), minimisation variables (gestational age, smoking status at recruitment, and first or preferred language) and month of birth. N/EET – Not/In Education, Employment or Training (Source: National Pupil Database, The Department for Education).

For peer review only - http://bmjopen.bmj.com/site/about/guidelines.xhtml

(e) Index of Multiple Deprivation quintiles

	N(%)	Q1=least deprived		Q2		Q3		Q4		Q5=most deprived	
		**Not RES**	**RES**	**Not RES**	**RES**	**Not RES**	**RES**	**Not RES**	**RES**	**Not RES**	**RES**
**Reading**	FNP	49 (30.3)	113 (67.7)	53 (36.3)	93 (63.7)	49 (33.6)	97 (66.4)	48 (37.2)	81 (62.8)	57 (37.8)	94 (62.2)
	Usual care	54 (38.9)	85 (61.1)	64 (44.1)	81 (55.9)	48 (31.8)	103 (68.2)	64 (43.0)	85 (57.0)	56 (39.4)	86 (60.6)
	Adjusted[a] OR (95% CI), p-value	1.57 (0.88 to 2.77), 0.12		1.42 (0.88 to 2.29), 0.16		0.96 (0.59 to 1.57), 0.88		1.33 (0.81 to 2.18), 0.25		1.07 (0.60 to 1.89), 0.83	
	Adjusted[b] OR (95% CI), p-value	1.75 (1.00 to 3.07), 0.05		1.42 (0.85 to 2.37), 0.18		0.89 (0.53 to 1.48), 0.64		1.41 (0.86 to 2.34), 0.18		1.00 (0.57 to 1.75), 0.99	
	Interaction p-value=0.70										
	Interaction p-value=0.37										
Maths	FNP	56 (34.6)	106 (65.4)	57 (39.0)	89 (61.0)	50 (34.3)	96 (65.7)	55 (42.6)	74 (57.4)	62 (41.1)	89 (58.9)
	Usual care	52 (37.4)	87 (62.6)	60 (41.4)	85 (58.6)	56 (37.1)	95 (62.9)	60 (40.3)	89 (59.7)	54 (38.0)	88 (62.0)
	Adjusted[a] OR (95% CI), p-value	1.15 (0.72 to 1.85), 0.56		1.12 (0.69 to 1.82), 0.65		1.17 (0.69 to 1.97), 0.56		0.93 (0.57 to 1.52), 0.77		0.91 (0.56 to 1.47), 0.69	
	Adjusted[b] OR (95% CI), p-value	1.36 (0.82 to 2.24), 0.23		1.12 (0.68 to 1.82), 0.66		1.11 (0.65 to 1.90), 0.69		0.98 (0.60 to 1.62), 0.95		0.88 (0.54 to 1.44), 0.62	
	Interaction p-value=0.91										
	Interaction p-value=0.81										

For peer review only - http://bmjopen.bmj.com/site/about/guidelines.xhtml

	N(%)	Q1=least deprived		Q2		Q3		Q4		Q5=most deprived	
		Not RES	RES	Not RES	RES	Not RES	RES	Not RES	RES	Not RES	RES
Science	FNP	37 (22.8)	125 (77.2)	36 (24.7)	110 (75.3)	40 (27.4)	106 (72.6)	45 (34.9)	84 (65.1)	44 (29.1)	107 (70.9)
	Usual care	45 (32.4)	94 (67.6)	45 (31.0)	100 (69.0)	35 (23.2)	116 (76.8)	47 (31.5)	102 (68.5)	46 (32.4)	96 (67.6)
	Adjusted[a] OR (95% CI), p-value	1.68 (1.00 to 2.81), 0.048		1.39 (0.80 to 2.41), 0.25		0.82 (0.46 to 1.47), 0.51		0.89 (0.52 to 1.51), 0.65		1.24 (0.74 to 2.06), 0.42	
	Adjusted[b] OR (95% CI), p-value	1.94 (1.13 to 3.30), 0.015		1.40 (0.76 to 2.57), 0.28		0.78 (0.43 to 1.40), 0.41		0.92 (0.55 to 1.55), 0.76		1.16 (0.69 to 1.96), 0.57	
	Interaction p-value=0.25										
	Interaction p-value=0.11										

RES = Reaching the expected standard; a FNP compared to Usual care; [a] Analysis adjusted for stratification (site), minimisation variables (gestational age, smoking status at recruitment, and first or preferred language); [b] Analysis adjusted for stratification (site), minimisation variables (gestational age, smoking status at recruitment, and first or preferred language) and month of birth. (Source: National Pupil Database, The Department for Education).

For peer review only - http://bmjopen.bmj.com/site/about/guidelines.xhtml

Table S17 Subgroup analyses for reaching the expected standard in the writing KS1 assessment for 2016/17 academic year

N(%)	FNP	Usual care	Adjusted[a] OR (95% CI), p-value	Interaction p-value	Adjusted[b] OR (95% CI), p-value	Interaction p-value
**Gender**						
Boys	109 (42.9)	83 (31.8)	1.61 (1.13 to 2.32), 0.009	0.034	1.62 (1.13 to 2.33), 0.009	0.039
Girls	132 (54.1)	126 (55.8)	1.03 (0.76 to 1.39), 0.87		0.94 (0.65 to 1.37), 0.76	
**Maternal age at recruitment**						
<16 years	18 (54.6)	9 (22.5)	4.67 (1.47 to 14.84), 0.009	0.016	5.28 (1.49 to 18.73), 0.010	0.018
≥16 years	223 (48.0)	200 (44.7)	1.13 (0.87 to 1.47), 0.35		1.15 (0.89 to 1.50), 0.29	
**Adaptive functioning**						
No difficulty	189 (52.5)	174 (46.7)	1.26 (0.93 to 1.70), 0.14	0.72	1.26 (0.93 to 1.70), 0.14	0.67
Difficulty in basic skills	52 (37.7)	34 (30.1)	1.41 (0.77 to 2.58), 0.26		1.53 (0.81 to 2.90), 0.19	
**NEET**						
EET (no not EET)	109 (47.8)	119 (52.2)	0.82 (0.56 to 1.19), 0.29	0.019	0.82 (0.56 to 1.19), 0.29	0.028
NEET	105 (49.1)	77 (38.3)	1.56 (1.05 to 2.30), 0.027		1.56 (1.05 to 2.30), 0.027	
**Deprivation quintile**						
1 – least deprived	63 (53.9)	39 (42.9)	1.65 (0.90 to 3.05), 0.11	0.89	1.83 (0.95 to 3.51), 0.07	0.86
2	48 (46.2)	46 (44.7)	1.00 (0.52 to 1.95), 0.99		1.04 (0.53 to 2.03), 0.92	
3	46 (50.6)	49 (46.7)	1.20 (0.67 to 2.15), 0.53		1.22 (0.66 to 2.26), 0.53	
4	40 (45.5)	35 (38.5)	1.28 (0.70 to 2.35), 0.42		1.28 (0.69 to 2.35), 0.43	
5 – most deprived	42 (44.2)	39 (41.1)	1.16 (0.64 to 2.08), 0.629		1.17 (0.64 to 2.14), 0.602	

For peer review only - http://bmjopen.bmj.com/site/about/guidelines.xhtml

[a] FNP compared to usual care. Analysis adjusted for stratification (site), minimisation variables (gestational age, smoking status at recruitment, and first or preferred language); [b] Analysis additionally adjusted for month of birth. N/EET – Not/In Education, Employment or Training (Source: National Pupil Database, The Department for Education).

For peer review only

For peer review only - http://bmjopen.bmj.com/site/about/guidelines.xhtml

Domestic abuse and CIN status:

Domestic abuse experienced by the mother was assessed using the Composite Abuse Scale (CAS) in the BB:0-2 trial at 24 months follow-up (with a score 1 or greater indicating some sort of abuse). The CAS could only be administered if the interview was face-to-face (as opposed to by telephone) and only if the participant was alone (N=607). In the BB:2-6 sample, this could be examined in 595 participants (FNP:322, Usual Care:273) and the proportion of participants experiencing some form of abuse was comparable to that found in the BB:0-2 trial (FNP: 122 (27.9%) vs UC:113 (41.4%)). There was also a relationship found between CAS and CIN status with 49 (20.9%) participants who experienced some abuse having a child being in need versus 42 (11.8%) of those who did not experience abuse. Domestic abuse at 24 months was to be explored as a possible effect mediator but due to lack of intervention effect in the child's CIN status, the need to examine the proportion of effect mediated by experience of domestic abuse at 24 months was redundant.

Care-experienced mothers and CIN status:

In addition, care-experienced mothers at time of recruitment to the BB:0-2 trial was examined. This was defined from the CLA dataset. Sixty-one mothers were identified as having experienced a period of care, four of whom were identified as care-experienced only after their BB:0-2 recruitment date. Fifty-seven mothers were examined for their duration of care and the women randomised to receive FNP experienced on average nine months more care than the Usual care arm (median (25th to 75th centile) FNP: 40 (15.5 to 113.0) UC: 31 (7.0 to 69.5) months). After merging these care-experienced mothers into the children's dataset, 56 mothers remained; seven mothers were excluded from the subgroup analyses since their child did not have any NPD data. Children of these care-experienced mothers were at a higher risk of being defined as in need themselves but no interaction was observed between trial arm and mothers care status. The duration of the maternal care status as a possible mediator effect was also stated in the statistical analysis plan but similarly to domestic abuse, due to lack of intervention effect in child's CIN status, then the need to examine the proportion of effect mediated by length of mother's care experience was redundant.

VIII. Treatment efficacy

The analyses of the following outcomes were re-run to investigate the effect of FNP dosage (number of visits over all delivery phases) using complier averaged causal effects modelling by fitting a structural mean model. The programme stated maximum number of visits over the whole period of FNP is 64 (14 in the pregnancy phase, 28 in the infancy phase, 22 in the toddler phase).

For peer review only - http://bmjopen.bmj.com/site/about/guidelines.xhtml

Referrals to CSC services: Adjusting the analysis for receipt of full dosage of FNP visits, made no difference to the conclusion of the main analysis for referral to social services (efficacy per visit odds ratio (OR)=1.00, 95% CI=0.99 to 1.004, p=0.613).

CIN status: Adjusting the analysis for receipt of FNP made no difference to the conclusion of the primary outcome (efficacy per visit OR=1.00, 95% CI=0.99 to 1.004, p=0.613).

Early Years assessment total point score: Adjusting the analysis for receipt of FNP we observe a 0.02 increase in point score in the between-arm mean difference in early years total score (as the number of visits increase so does the score) however the confidence interval around this estimate is wide (-0.004 to 0.040). The estimated treatment efficacy for participants receiving all 64 visits was 1.15 (95% CI -0.26 to 2.56), p=0.106 showing no change to the overall conclusions compared to the primary analysis.

KS1 attainment: Adjusting the analysis for FNP receipt we observe an increase in the odds of an event occurring in the FNP arm compared UC arm for every unit increase in FNP visit received. For all areas of learning (reading, writing, maths and science), adjusting the analysis for FNP receipt strengthens the effect of FNP, but it is the reading and writing assessment where given the full dosage of 64 FNP visits the odds make a significant difference. For reading assessments, the odds of reaching the expected standard increases from OR=1.23 (95% CI=0.99 to 1.53), p=0.051 in the main analysis to 1.38 (1.07 to 1.77), p=0.015. The efficacy per visit for reading was 1.005 (1.001 to 1.009), p=0.015; maths: 1.001 (0.997 to 1.005), p=0.535; science: 1.004 (0.999 to 1.008), p=0.112; writing: 1.013 (1.09 to 1.017), p=0.001.

IX. Missing data

Since only a small number (n=40 (2.7%)) of children were not linked to any NPD dataset and not included in the primary outcome analyses, no multiple imputation was carried out.

For peer review only - http://bmjopen.bmj.com/site/about/guidelines.xhtml

X. Cost Consequence Analysis

Table S18 Cost-consequence balance sheet for the family-nurse based intervention for the four years between children's ages 2 and 6.

**<u>Mothers</u>**						
	Mean resource use per participant			Mean cost per participant (£)		
	FNP	Usual Care	Incremental (FNP-Usual Care)	FNP	Usual Care	Incremental (FNP-Usual Care)
**Health related resource use**						
Inpatient Attendances						
Overnight admissions (N)	3.04	2.99	0.05			
Overnight length of stay (N nights)	2.32	2.32	0.00	2,826.79	2,844.58	-17.79
Day Admittances	2.93	2.91	0.02	1,874.61	1,890.11	-15.50
Outpatient Attendances						
All Outpatient Attendances	23.29	22.61	0.68	2,244.62	2,189.32	55.30
Accident and Emergency						
A&E Visits (N)	5.19	4.93	0.26	411.34	392.03	19.31
Total Resource Use						
				7,357.36	7,316.04	41.32 p=0.393
**<u>Children</u>**						
	Mean resource use per participant			Mean cost per participant (£)		
	FNP	Usual Care	Incremental (FNP-Usual Care)	FNP	Usual Care	Incremental (FNP-Usual Care)
**Health related resource use**						
Inpatient Attendances - All						
Overnight admissions (N)	1.57	1.55	0.02			
Overnight length of stay (N nights)	3.57	3.39	0.18	1,962.95	2,033.71	-70.76
Day Admittances	0.98	0.97	0.01	744.94	724.90	20.04
Inpatient Attendances – All (Excluding Injuries and Ingestions)						
Overnight admissions (N)	1.51	1.49	0.02	1,883.44	1,951.15	-67.71
Overnight length of stay (N nights)	3.61	3.44	0.17			
Day Admittances	0.90	0.88	0.02	689.22	667.09	22.13
Inpatient Attendances – Injuries and Ingestions Only						
Overnight admissions (N)	0.06	0.06	0.00	79.51	82.55	-3.04

For peer review only

Overnight length of stay (N nights)	2.50	2.05	0.45			
Day Admittances	0.08	0.09	-0.01	55.72	57.81	-2.09
Outpatient Attendances						
All Outpatient Attendances	6.89	7.44	-0.55	673.24	727.79	-54.55
Accident and Emergency - All						
A&E Visits (N)	4.93	4.62	0.31	395.73	366.83	28.90
Accident and Emergency - All (Excluding Injuries and Ingestions)						
A&E Visits (N)	3.80	3.52	0.28	305.12	280.20	24.92
Accident and Emergency – Injuries and Ingestions Only						
A&E Visits (N)	1.12	1.10	0.02	75.09	69.91	5.18
Total Resource Use						
				3,778.13	3,853.22	-75.09 p=0.865
**Consequences**						
Child in Need status recorded at any time during the follow-up period.				OR: 0.98 (95%CI: 0.74 to 1.31) p=0.902		
Referral to Social Services				OR: 0.97 (0.74 to 1.28) p=0.829		
Child Protection registration (CPP)				OR: 1.04 (0.69 to 1.57) p=0.846		
Details of a CPP						
• Neglect				Reference		
• Emotional				OR: 2.77 (1.02 to 7.56) p=0.046		
• Physical				OR: 1.25 (0.32 to 4.88) p=0.743		
• Sexual and multiple				OR: 1.13 (0.33 to 3.86) p=0.848		
CIN categorisation						
• Abuse or Neglect				Reference		
• Family dysfunction				OR: 0.95 (0.54 to 1.69) p=0.868		
• Family in acute stress				OR: 1.47 (0.61 to 3.54) p=0.389		
• Low income, absent parenting, socially unacceptable behaviour				OR: 2.38 (0.86 to 6.57) p=0.093		
• Child/parent disability				OR: 1.47 (0.45 to 4.84) p=0.525		
Looked after status				OR: 0.90 (0.52 to 1.57) p=0.712		
Child looked after period of care (months)				OR: 0.75 (0.65 to 0.86) p<0.001		
DNA appointments						
• DNA at least once				OR: 1.00 (0.79 to 1.26) p=0.997		
Injuries and ingestions						
• Emergency attendance						
○ No				Reference		
○ Yes				OR: 1.17 (0.95 to 1.45) p=0.149		
• Admission to hospital						

For peer review only - http://bmjopen.bmj.com/site/about/guidelines.xhtml

○ No	Reference
○ Yes	OR: 0.87 (0.63 to 1.20) p=0.407
• Either hospital attendance and/or admission to hospital	
○ No	Reference
○ Yes	OR: 1.11 (0.89 to 1.37) p=0.351
Special Educational Needs	OR: 0.83 (0.67 to 1.03) p=0.097
Early Years assessment	
• Achieving good level of development in all five areas of learning	OR: 1.26 (1.03 to 1.55) p=0.026
• Achieving good level of development in all 17 early learning goals	OR: 1.24 (1.01 to 1.52) p=0.043
School attendance	
• Overall absences	
○ No absences	Reference
○ At least one absence	OR: 1.00 (0.47 to 2.12) p=0.998
• Overall unauthorised absences	
○ No absences	Reference
○ At least one absence	OR: 0.95 (0.76 to 1.18) p=0.620
• Overall authorised absences	
○ No absences	Reference
○ At least one absence	OR: 1.01 (0.58 to 1.75) p=0.984
KS1 attainment	
• Reading	
○ Lower than expected	Reference
○ Reaching the expected standard	OR: 1.23 (0.99 to 1.53) p=0.051
• Writing academic year 2016/17	
○ Lower than expected	Reference
○ Reaching the expected standard	OR: 1.24 (0.97 to 1.60) p=0.090
• Maths	
○ Lower than expected	Reference
○ Reaching the expected standard	OR: 1.04 (0.84 to 1.28) p=0.731
• Science	
○ Lower than expected	Reference
○ Reaching the expected standard	OR: 1.14 (0.91 to 1.43) p=0.254

For peer review only

The RECORD statement – checklist of items, extended from the STROBE statement, that should be reported in observational studies using routinely collected health data.

	Item No.	STROBE items	Location in manuscript where items are reported	RECORD items	Location in manuscript where items are reported
**Title and abstract**					
	1	(a) Indicate the study's design with a commonly used term in the title or the abstract (b) Provide in the abstract an informative and balanced summary of what was done and what was found	1a/b Abstract	RECORD 1.1: The type of data used should be specified in the title or abstract. When possible, the name of the databases used should be included.<br><br>RECORD 1.2: If applicable, the geographic region and timeframe within which the study took place should be reported in the title or abstract.<br><br>RECORD 1.3: If linkage between databases was conducted for the study, this should be clearly stated in the title or abstract.	1.1 Abstract<br>1.2 Abstract<br>1.3 Abstract
**Introduction**					
Background rationale	2	Explain the scientific background and rationale for the investigation being reported	Introduction		
Objectives	3	State specific objectives, including any prespecified hypotheses	Introduction > Objectives		
**Methods**					
Study Design	4	Present key elements of study design early in the paper	Methods> Study design, setting and participants		
Setting	5	Describe the setting, locations, and relevant dates, including periods of recruitment, exposure, follow-up, and data collection	Methods>Procedure and data sources		

For peer review only

Participants	6	*(a) Cohort study* - Give the eligibility criteria, and the sources and methods of selection of participants. Describe methods of follow-up<br>*Case-control study* - Give the eligibility criteria, and the sources and methods of case ascertainment and control selection. Give the rationale for the choice of cases and controls<br>*Cross-sectional study* - Give the eligibility criteria, and the sources and methods of selection of participants<br><br>*(b) Cohort study* - For matched studies, give matching criteria and number of exposed and unexposed<br>*Case-control study* - For matched studies, give matching criteria and the number of controls per case	6a Methods> Study design, setting and participants<br><br>6b NA	RECORD 6.1: The methods of study population selection (such as codes or algorithms used to identify subjects) should be listed in detail. If this is not possible, an explanation should be provided.<br><br>RECORD 6.2: Any validation studies of the codes or algorithms used to select the population should be referenced. If validation was conducted for this study and not published elsewhere, detailed methods and results should be provided.<br><br>RECORD 6.3: If the study involved linkage of databases, consider use of a flow diagram or other graphical display to demonstrate the data linkage process, including the number of individuals with linked data at each stage.	6.1 Methods> Study design, setting and participants<br><br>6.2 NA<br><br>6.3 Protocol paper cited, Figure 1
Variables	7	Clearly define all outcomes, exposures, predictors, potential confounders, and effect modifiers. Give diagnostic criteria, if applicable.	Methods> Study design, setting and participants, Outcomes; Appendix	RECORD 7.1: A complete list of codes and algorithms used to classify exposures, outcomes, confounders, and effect modifiers should be provided. If these cannot be reported, an explanation should be provided.	Methods> Study design, setting and participants, Outcomes
Data sources/ measurement	8	For each variable of interest, give sources of data and details of methods of assessment (measurement).<br>Describe comparability of assessment methods if there is more than one group	Methods> Study design, setting and participants, Outcomes; Appendix		

For peer review only

Bias	9	Describe any efforts to address potential sources of bias	Methods> Efforts to address sources of bias		
Study size	10	Explain how the study size was arrived at	Methods> Power calculation		
Quantitative variables	11	Explain how quantitative variables were handled in the analyses. If applicable, describe which groupings were chosen, and why	Methods>Statistical analysis		
Statistical methods	12	(a) Describe all statistical methods, including those used to control for confounding<br>(b) Describe any methods used to examine subgroups and interactions<br>(c) Explain how missing data were addressed<br>(d) *Cohort study* - If applicable, explain how loss to follow-up was addressed<br>*Case-control study* - If applicable, explain how matching of cases and controls was addressed<br>*Cross-sectional study* - If applicable, describe analytical methods taking account of sampling strategy<br>(e) Describe any sensitivity analyses	12a-c, e: Methods>Statistical analysis; Economic analysis<br>12d: NA		
Data access and cleaning methods		..		RECORD 12.1: Authors should describe the extent to which the investigators had access to the database population used to create the study population.	Methods> Study design, setting and participants, Outcomes; Appendix

For peer review only

				RECORD 12.2: Authors should provide information on the data cleaning methods used in the study.	
Linkage		..		RECORD 12.3: State whether the study included person-level, institutional-level, or other data linkage across two or more databases. The methods of linkage and methods of linkage quality evaluation should be provided.	Methods> Procedure and data sources
**Results**					
Participants	13	(a) Report the numbers of individuals at each stage of the study (*e.g.*, numbers potentially eligible, examined for eligibility, confirmed eligible, included in the study, completing follow-up, and analysed)<br>(b) Give reasons for non-participation at each stage.<br>(c) Consider use of a flow diagram	Table S3, Figure 1	RECORD 13.1: Describe in detail the selection of the persons included in the study (*i.e.,* study population selection) including filtering based on data quality, data availability and linkage. The selection of included persons can be described in the text and/or by means of the study flow diagram.	Figure 1
Descriptive data	14	(a) Give characteristics of study participants (*e.g.*, demographic, clinical, social) and information on exposures and potential confounders<br>(b) Indicate the number of participants with missing data for each variable of interest<br>(c) *Cohort study* - summarise follow-up time (*e.g.*, average and total amount)	14a: Table 1<br>14b: Table S3, Tables 2-4		
Outcome data	15	*Cohort study* - Report numbers of outcome events or summary measures over time<br>*Case-control study* - Report numbers in each exposure	Tables 2-4		

For peer review only

		category, or summary measures of exposure<br>*Cross-sectional study* - Report numbers of outcome events or summary measures			
Main results	16	(a) Give unadjusted estimates and, if applicable, confounder-adjusted estimates and their precision (e.g., 95% confidence interval). Make clear which confounders were adjusted for and why they were included<br>(b) Report category boundaries when continuous variables were categorized<br>(c) If relevant, consider translating estimates of relative risk into absolute risk for a meaningful time period	Tables 2-4		
Other analyses	17	Report other analyses done—e.g., analyses of subgroups and interactions, and sensitivity analyses	Appendix		
**Discussion**					
Key results	18	Summarise key results with reference to study objectives	Discussion> Principal findings		
Limitations	19	Discuss limitations of the study, taking into account sources of potential bias or imprecision. Discuss both direction and magnitude of any potential bias		RECORD 19.1: Discuss the implications of using data that were not created or collected to answer the specific research question(s). Include discussion of misclassification bias, unmeasured confounding, missing data, and changing eligibility over time, as they pertain to the study being reported.	Discussion> Strengths and weaknesses of this study, Strengths and weaknesses in comparison to other studies
Interpretation	20	Give a cautious overall interpretation of results considering objectives,			Discussion> Strengths and weaknesses of this study, Strengths

For peer review only

		limitations, multiplicity of analyses, results from similar studies, and other relevant evidence			and weaknesses in comparison to other studies
Generalisability	21	Discuss the generalisability (external validity) of the study results			Discussion> Strengths and weaknesses in comparison to other studies
**Other Information**					
Funding	22	Give the source of funding and the role of the funders for the present study and, if applicable, for the original study on which the present article is based			Described in Funding section
Accessibility of protocol, raw data, and programming code		..		RECORD 22.1: Authors should provide information on how to access any supplemental information such as the study protocol, raw data, or programming code.	Protocol paper cited in references; access to data described in Data sharing statement

*Reference: Benchimol EI, Smeeth L, Guttmann A, Harron K, Moher D, Petersen I, Sørensen HT, von Elm E, Langan SM, the RECORD Working Committee. The REporting of studies Conducted using Observational Routinely-collected health Data (RECORD) Statement. *PLoS Medicine* 2015; in press.

*Checklist is protected under Creative Commons Attribution (CC BY) license.

For peer review only

Title: A nurse-led home-visitation programme for first-time mothers in reducing maltreatment and improving child health and development (BB:2-6): longer-term outcomes from a randomised cohort using data-linkage

Authorship:

Michael Robling, [1,2] (Director of Population Health Trials), RoblingMR@cardiff.ac.uk
Fiona Lugg-Widger, [1] (Research Fellow), LuggFV@cardiff.ac.uk
Rebecca Cannings-John, [1] (Senior Research Fellow), CanningsRL@cardiff.ac.uk
Lianna Angel, [2] (Research Associate), AngelL@cardiff.ac.uk
Susan Channon, [1] (Director Research Design and Conduct Service) ChannonS2@cardiff.ac.uk
Deborah Fitzsimmons, [3] (Personal Chair, Public Health, Policy and Social Sciences) d.fitzsimmons@swansea.ac.uk
Kerenza Hood, [1] (Director of the Centre for Trials Research) HoodK1@cardiff.ac.uk
Joyce Kenkre, [4] (Professor of Primary Care) joyce.kenkre@southwales.ac.uk
Gwenllian Moody, [1] (Research Associate) MoodyG@cardiff.ac.uk
Eleri Owen-Jones, [1] (Research Associate) Owen-JonesCE@cardiff.ac.uk
Rhys Pockett, [3] (Senior Lecturer) r.d.pockett@swansea.ac.uk
Julia Sanders, [5] (Professor of Clinical Nursing and Midwifery) SandersJ3@cardiff.ac.uk
Jeremy Segrott, [1,2] (Senior lecturer) SegrottJ@cardiff.ac.uk
Thomas Slater, [6] (Senior lecturer) slaterTB1@cardiff.ac.uk

Affiliations:

1. Centre for Trials Research, Cardiff University, Cardiff, UK
2. DECIPHer, Cardiff University, UK
3. Swansea Centre for Health Economics, Swansea University, UK
4. Faculty of Life Sciences and Education, University of South Wales, UK
5. School of Healthcare Sciences, Cardiff University, UK
6. School of Social Sciences, Cardiff University, UK

Corresponding author:

Michael Robling, Director of Population Health Trials, RoblingMR@cardiff.ac.uk, Centre for Trials Research, College of Biomedical & Life Sciences, Cardiff University, 7th Floor, Neuadd Meirionnydd, Heath Park, Cardiff. CF14 4YS

Keywords: Home-visiting, maltreatment, child development, routine data, cohort

For peer review only

Abstract (300 words)

Objectives Measure effectiveness of Family Nurse Partnership (FNP) home-visiting programme in reducing maltreatment and improving maternal health and child health, developmental and educational outcomes; explore effect moderators, mediators; describe costs.

Design Follow-up of BB:0-2 trial cohort (ISRCTN:23019866) up to age 7 years in England using record linkage.

Participants 1,618 mothers aged 19 years or younger and their firstborn child(ren) recruited to BB:0-2 trial at less than 25 weeks gestation and not mandatorily withdrawn from trial or opted out. Intervention families were offered up to a maximum of 64 home visits by specially trained nurses from pregnancy until firstborn child was 2 years old, plus usually provided health and social care support. Comparator was usual care alone.

Outcome measures Primary outcome: state-verified child-in-need status recorded at any time during follow-up. Secondary outcomes: referral to social services, child protection registration (plan), child-in-need categorisation, looked-after status, recorded injuries and ingestions any time during follow-up, early childcare and educational attendance, school readiness and attainment at Key Stage 1 (KS1), healthcare costs.

Results Match rates for 1,547 eligible children (1517 singletons, 15 sets of twins) were 98.3% (NHS Digital) and 97.4% (National Pupil Database). There was no difference between study arms in the proportion of children being registered as in need (adjusted OR=0.98, 95% CI=0.74,1.31), or for any other measure of maltreatment. Children in the FNP arm were more likely to achieve a good level of development at reception age (school readiness) (adjusted OR=1.24, 95% CI= 1.01,1.52). After adjusting for birth month, children in FNP arm were more likely to reach the expected standard in reading at KS1 (adjusted OR=1.26, 95% CI=1.02,1.57). We found no trial arm differences for resource use and costs.

Conclusions FNP did not improve maltreatment or maternal outcomes. There was evidence of small advantages in school readiness and attainment at KS1.

Strengths and Limitations

- We used administrative records and novel data linkage to provide longer-term follow-up for a well characterised trial cohort
- This has maximised cohort retention and minimised bias
- We used data from a range of sectors including health, social care and education to assess the broad impact of the specialist home visiting programme

For peer review only

Manuscript (Word count is ~~5026~~5773)

Introduction

Protecting children from maltreatment and promoting their healthy development are UK policy priorities. The Family Nurse Partnership (FNP) is a preventative home-visiting programme for young women expecting their first child. FNP was developed in the USA as the Nurse Family Partnership (NFP) and subsequently adapted for implementation in England in 2007.

Three US trials have demonstrated programme improvements in prenatal health behaviours and birth outcomes, sensitive childcare, maternal life-course and child functioning.[1–3] A subgroup analysis of poor unmarried teenage mothers in the first US trial (n=54) found verified maltreatment by age 2 years in 19% of 32 control children and 4% of the 22 children in the group in receipt of NFP during both pregnancy and infancy (mean % difference: 0.15, 95% confidence interval (CI) of −0.01 to 0.31).[1] Additionally, there was a 56% relative reduction in emergency department encounters for injuries and ingestions during the second year of life. For children with state-verified maltreatment reported by age 4, children in the NFP group had fewer risks for harm than the control group between 25 and 50 months of life. A trial of VoorZorg, the Dutch adaptation of NFP, found a reduction in child-protecting agency reports by age three years for families in the home-visiting arm.[4,]

We evaluated short-term FNP programme outcomes to age 2 years in our Building Blocks trial (BB:0-2).[~~6~~5] We found no difference for four primary outcomes: maternal tobacco use in late pregnancy, birth weight of the baby, proportion of women with a second pregnancy within 24 months postpartum and emergency attendances and hospital admissions for the child within 24 months postpartum. There were some differences in favour of FNP for secondary child development outcomes including maternal-reported cognitive function and language at 24 months~~.~~.[5] As the previous US trials showed benefit for maltreatment outcomes increasingly after age four we sought to establish whether FNP moderates maltreatment outcomes over a medium-term period. Through access to administrative data, we aimed to determine impact across a range of child maltreatment outcomes and key indicators of neglect (e.g. injuries and ingestions). We also sought to assess impacts on other programme relevant developmental and maternal outcomes as well as costs.

Objectives

Primary: to determine programme effectiveness in reducing ~~objectively measured~~ maltreatment, when compared with usually provided health and social care alone. Secondary: to determine programme effectiveness in reducing maltreatment, medium-term programme outcomes such as

For peer review only - http://bmjopen.bmj.com/site/about/guidelines.xhtml

subsequent pregnancies, school readiness and educational outcomes, the impact of moderators of programme effect and the cost and consequences of the programme.

Methods

Study design, setting and participants

This study followed a cohort of mothers and children who had participated in the BB:0-2 trial for a further five years using administrative data only..[6,7] Data were retrieved from public sector providers and linked to the trial dataset using opt-out consent, following REC approval (14/WA10062) and s251 support (CAG 10-08(b)/2014). Children and mothers were followed up until the child reached Key Stage 1 teacher-based assessment (KS1) by which time most children were seven years old.

Building Blocks: 2-6 (BB:2-6) study participants were women and their first child (or twins, if relevant) who were not mandatorily withdrawn from the BB:0-2 trial; did not electively withdraw and/or did not opt-out of this follow-on study..[5-7] BB:0-2 trial participants were eligible if women were nulliparous, aged 19 years or younger, were recruited at less than 25 weeks' gestation, living within the catchment area of a local FNP team, able to provide consent and speak English. Women expecting multiple births and those with a previous pregnancy ending in miscarriage, stillbirth, or termination were eligible. Women planning to have their child adopted or to move beyond the FNP catchment area for more than 3 months were not eligible. [5-8] Trial participants had been randomly assigned to FNP or usual care, with randomisation stratified by site and minimised by gestation (<16 weeks vs ≥16 weeks), smoking (yes vs no), and preferred language of data collection (English vs non-English) and weighted towards minimising the imbalance in trial groups with probability 0.8.

Intervention and comparator (BB:0-2 Trial)

The FNP programme provides up to a maximum of 64 home visits from specially trained family nurses during pregnancy until the child is two years old addressing personal and environmental health, life course development, maternal role, family and friends and access to health and social services. For the 697 women randomised and enrolled to FNP in BB:0-2 and then retained in the BB:2-6 cohort, the average number (SD) of valid visits received by FNP clients by programme phase was: Pregnancy 9.74 (3.42), Infancy 18.7 (5.97) and Toddlerhood 13.28 (5.46) (Table S1). In the BB:0-2 trial, mean (SD) visit duration varied by delivery phase (Pregnancy: 79.14 mins (13.78), Infancy: 73.17 (11.61), Toddlerhood: 74.75 (13.50) which exceeded the programme target of 60 minutes.[87] All trial participants received usually provided health and social care services. Participants in the usual care (UC) arm received these services alone..[5,7-9] The number (SD) of midwifery contacts were recorded in the BB:0-2 trial and were for FNP clients 10.68 (5.25) and for women in UC arm 10.69 (5.34).[109] The number (SD) of home visits from specialist public health nurses (SPHN) reported by trial participants by 18 months postpartum was for FNP clients 4.70 (7.81) and for women in the UC

For peer review only

arm 5.01 (5.5). The average number (SD) of clinic contacts with SPHNs was 0.70 (2.29) for women in the FNP arm and 6.31 (7.07) for women in the control arm and 0.70 (2.29) in the FNP arm.

Procedure and data sources

The BB:0-2 trial consented 1618 eligible women between June 2009 and July 2010 and we concluded all BB:2-6 follow-up in May 2018. Maternal and child identifiers were sent to NHS Digital and the Department for Education (DfE) for matching with their databases. Maternal identifiers were also sent to the Department of Health and Social Care (DHSC) for matching with abortion statistics. Matching with NHS Digital and DHSC used a combination of NHS number, date of birth, postcode and sex. NHS Digital also provided a linked Hospital Episode Statistics (HES) - Office for National Statistics (ONS) extract for mortality data. Matching with the DfE used exact matching on first name and surname, date of birth and postcode (of both mother and child for social care data; all other datasets, just child). All matched data were sent to a third-party data safe haven (Secure Anonymised Information Linkage (SAIL) Databank) and linked by project identifiers to trial data and analysed via remote access..[10-12]

Outcomes

The primary outcome was Child in Need (CIN) status recorded at any time by a local department of children's social care services (CSCS) (and sourced from the NPD). This includes children assessed as unlikely to achieve or maintain a reasonable level of health or development, or whose health and development is likely to be significantly or further impaired, without the provision of services; or is a child who is disabled. Secondary outcomes were additional objectiveformally reported measures of maltreatment (referral to CSCS, child being on a Child Protection Plan, CIN categorisation, Looked after status (mother, child)); Associated measures of maltreatment (recorded injuries and ingestions, non-attendance rates for hospital appointments); Maternal outcomes (subsequent pregnancies); Child health, developmental and educational outcomes (special educational needs, early educational attendance and assessments (Early Years Foundation Stage Profile (EYFSP), KS1)); and health resource use. Study assessment domains, outcomes and principal data providers and analysis are described in the appendix (Table S2).

For peer review only - http://bmjopen.bmj.com/site/about/guidelines.xhtml

Power calculation

Our sample size was fixed by the cohort available from BB:0-2. However, for CIN status, while UK data on rates are not specific to the age-range of interest, the rate per 10,000 general population aged 5-9 years is 4.6% for local authorities covering BB:0-2 trial sites.[13] The rate of CIN status would be expected to be greater in the specific study sample. Therefore, we assumed a rate of 8%, hypothesised that FNP would reduce the presence of CIN in the first six years and assumed a difference of 4% would be important. To detect a difference of 4% (4% vs 8%) would require 602 children in each arm with 80% power and a two-sided 5% alpha level. With 1562 children available for follow-up (i.e. excluding mandatory withdrawals) and follow up through administrative records with 10% loss in tracking and linkage we would have 1405 participants, sufficient data to assess the primary outcome.

Efforts to address sources of bias

All maltreatment and child developmental outcomes were independently state-verified (social worker) reports or teacher-assessments respectively, recorded in administrative service data and abstracted from national data providers. By using administrative data and established linking fields we were able to minimise bias due to loss to follow-up. An a priori statistical analysis plan described primary, secondary (including sub-group) and sensitivity analyses, and any additional exploratory analysis is clearly highlighted.

Statistical analysis

Analysis was conducted on participants whose identifiers could be sent, linked and data released by the data providers. This excluded participants whose individual data could be used but could not be matched due to incorrect linking fields, other exclusions from health or education: e.g. private or home schooling and National opt-outs. Imbalance between the BB:0-2 and eligible BB:2-6 participants was quantified using descriptive statistics in baseline demographics, clinical and questionnaire data. We conducted all analyses on an intention-to-treat (ITT) basis with complete case population (with follow-up data). Therefore, participants remained in the BB:0-2 trial groups they were randomised to regardless of the intervention received. We used multilevel modelling to allow for clustering of effect within the 18 sites and family nurse level and both fitted as random effects. Where clustering was minimal at the family nurse level, results from the two-level model are presented. Parameter estimates are reported alongside a 95% CI and p-value. We adjusted for variables used in randomisation (smoking status, gestational age, language). We defined loss to follow up in this study as a child death or adoption, both of which we were able to determine. We excluded children from analyses of binary outcomes where follow-up was incomplete and no event was observed. With time-to-event analyses, children were censored at these events.

For peer review only - http://bmjopen.bmj.com/site/about/guidelines.xhtml

For peer review only

Primary comparative analysis examined whether the firstborn child(ren) ('BB:0-2') had ever been referred to CSCS and classed as a child in need at any point between birth and 6 years of age (appendix). For this binary outcome we used logistic multilevel modelling to investigate differences in the proportion defined as in need between the trial arms. We present the resulting estimate as an odds ratio. For binary and categorical outcomes, comparative analysis used logistic and multinomial modelling respectively with results presented as odds ratios (OR). For continuous outcomes we used linear regression and present mean differences. For count data we used Poisson regression modelling; where event distribution displayed signs of over dispersion a Negative Binomial regression model was used (or zero-inflated model if appropriate). Results are presented as incidence rate ratios (IRR). Time to event data used Cox regression modelling presented with hazard ratios (HR). To reduce problems with multiple comparisons, some outcomes were only assessed descriptively (for example, we prioritised and tested overall scores for a Good Level of Development in the Early Years Profile, rather than the individual areas of learning). Similarly, for outcomes that we expected to reflect a small number of events or higher levels of missingness we also took a descriptive approach. Nevertheless, we present all a priori comparative analyses in the main tables and all other descriptive and exploratory analyses in the supplementary materials.

KS1 data were received in two tranches from NPD, firstly children assessed in 2016/17 academic year (received in May 2018) and then younger children who were assessed in 2017/18 (received May 2019). Reading, maths and science were examined for the whole cohort over both academic years. However, following changes made within the 2017/18 writing assessment (such that judgements in 2018 are not directly comparable to those made using the previous interim frameworks in 2016/2017), writing was only statistically assessed using 2016/17 data and the data from 2017/18 were presented descriptively. Sensitivity analyses for primary and selected secondary outcomes (EYFSP, KS1, referral to CSCS) included adjusting for hypothesised confounders at baseline, dosage effects (i.e. number of visits) using complier average causal effects (CACE) modelling and sub-group analysis of potential effect moderators and mediators (maternal deprivation, adaptive functioning, NEET (not in education, employment or training) status at recruitment, maternal age at recruitment, child sex, maternal care status, duration of maternal care and domestic abuse self-reported at 24 months) as interaction terms in the main comparative models.

Results of all planned sensitivity analyses are presented in the appendix. An additional post hoc sensitivity analysis adjusted for the child's month of birth (categorised into quarter of birth) in the EYFSP and KS1 outcomes.

For peer review only - http://bmjopen.bmj.com/site/about/guidelines.xhtml

Economic Analysis

A cost consequences analysis of FNP over the full follow-up period (BB:0–2 and BB:2–6) took a secondary health care (UK NHS) perspective. The principal data source was Hospital Episode Statistics (HES) records (inpatient, outpatient, A&E), which were coded in Healthcare Resource Group (HRG) Grouper and matched to appropriate NHS Reference costs. Maternal and child resource use were costed separately and valued in £ sterling. Only direct healthcare costs were analysed. Indirect costs such as lost wages or childcare costs were not available within the routinely collected data. Where data were absent in HES records it was assumed no resource was incurred. No primary care data were available beyond 18 months postpartum. Costs were discounted back from year of event to baseline (2009/10) at an annual rate of 3.5% as described by Central Government's guidance on appraisal and evaluation~~.~~. [14]

Data management

Small numbers were handled according to SAIL Databank rules where any cell counts under five were suppressed in reporting. Where abortion data are presented, we handled in accordance with the data sharing agreement with DHSC where counts less than 9 were suppressed. Data were analysed using Stata v16 and IBM SPSS v23.0.

Patient and public involvement

Activities involved four groups of young people (CASCADE Voices, Our Place, FNP graduates and ALPHA). These activities contributed to three aspects of the study: data-linkage methods (e.g. to optimise acceptability of the proposed dissent model, use of data linkage and communication of these methods~~),~~)[15], study outcomes (by seeking views on the importance of the study outcomes) and dissemination of findings (including best way to present results, method of dissemination).

Results

The study population following the BB:0-2 trial tracing and matching process is shown in Figure 1. Five women were ineligible due to not meeting original trial entry criteria and 78 were mandatory withdrawals mainly due to fetal or infant death and adoption.[~~6~~5] The 110 women who electively withdrew during the trial were given the opportunity to opt-out from further data usage, 16 of whom removed consent for any further contact. Thus 94 women who had electively withdrawn from BB:0-2 were included, and their details sent for tracing alongside the remaining 1,452 mothers (1,546 mother-child dyads in total) to update contact details to improve matching. One dyad was subsequently removed as deceased and the remaining 1,545 contacted by letter, email or text of whom eight dissented from having their records linked. 1,537 mothers and 1,547 children were sent to the DfE and

For peer review only - http://bmjopen.bmj.com/site/about/guidelines.xhtml

NHS Digital for matching. These formed the population for the BB:2-6 study. Match rates for children were 98.3% (NHS Digital) and 97.4% (DfE).

Numbers matched to and returned for analysis differed by data source and population (Table S3). Owing to having discontinued their education, few mothers, compared with children, were matched to any DfE data set (19% vs. 97%, respectively); these events were based mainly on the CIN data set. For selected maternal and child baseline variables and delivery of FNP assessed against programme fidelity goals, the BB:2-6 sample appears broadly representative of the original BB:0-2 cohort (Tables S4 and S5). Baseline characteristics of the BB:2-6 mothers and children were well balanced between trial arms (Table 1).

Table 1 Socio-demographic characteristics of mother and babies at baseline by trial arm

	FNP N=766	Usual care N=771
**Maternal age at recruitment (years)**	17·9 (17·0 to 18·8)	17·9 (16·9 to 18·8)
**Ethnicity**		
White background	675 (88·1%)	680 (88·2%)
Mixed background	44 (5·7%)	38 (4·9%)
Asian background	15 (2·0%)	10 (1·3%)
Black background	29 (3·8%)	40 (5·2%)
Chinese or Other background	<5	<5
**Relationship status with baby's father**		
Married	6 (0·8%)	10 (1·3%)
Separated	72 (9·4%)	78 (10·1%)
Closely involved/boyfriend	582 (76·0%)	586 (76·0%)
Just friends	106 (13·8%)	97 (12·6%)
**NEET Status***		
Yes	315/764 (41·1%)	319/769 (41·4%)
No	345/764 (45·0%)	333/769 (43·2%)
**IMD Overall Score†**	38·1 (24·6 to 52·6)‡	38·1 (25·5 to 51·6)§
**Generalised self-efficacy scale (score 10 to 40)¶**	30·0 (28·0 to 33·0)‖	30·0 (27·0 to 32·0)**
**Adaptive functioning:**		
**Difficulty in at least one basic skill**		
Yes	213/765 (27·8%)	184/770 (23·9%)
No	552/765 (72·2%)	586/770 (76·1%)
**Had 3 or less life skills (out of 5)**		

For peer review only - http://bmjopen.bmj.com/site/about/guidelines.xhtml

	FNP N=766	Usual care N=771
Yes	185/763 (24·2%)	218/769 (28·3%)
No	578/763 (75·8%)	551/769 (71·7%)
**At least one burden**		
Yes	214/759 (28·2%)	237/767 (30·9%)
No	545/759 (71·8%)	530/767 (69·1%)
**Cigarette smoking participant self-reported**		
Ever smoked		
Yes	615 (80·3%)	612 (79·4%)
No	151 (19·7%)	159 (20·6%)
**Child characteristics**	**FNP N=773**	**Usual Care N=774**
**Sex**		
Male	381/773 (49·3%)	406/773 (52·5%)
Female	392/773 (50·7%)	367/773 (47·5%)
**Birthweight (grams)** Mean (SD)	3223·81 (606·0)††	3215·52 (555·56)‡‡
**NNU admission (direct or subsequent)**		
Yes	76/716 (10·6%)	66/749 (8·8%)
No	640/716 (89·4%)	683/749 (91·2%)

Data are n (%), n/N (%), or median (25th to 75th centile) unless otherwise stated. <5 = numbers suppressed. FNP = Family Nurse Partnership Programme + Usual Care. IMD = Index of Multiple Deprivation. SD= standard deviation. NNU = Neonatal unit. *Definition of NEET status: Not in education employment or training (applicable only to those whose age at end of previous academic year at time of baseline interview was >16). †Higher IMD score indicated more deprivation. Mean IMD Score for England in 2010 was 21.67 [Wilkinson DL, Sniehotta FF, Michie S. Targeting those in need: Baseline data from the first English National Health Service (NHS) health trainer service: *Psychology, health & medicine* 2011; **16**(6): 736-48], ‡N=760. §N=765. ¶ Higher score indicates higher level of self-efficacy. ‖N=764. **N=769. ††N=724. ‡‡N=752.

For the ~~primary outcome~~definition of a child in need~~, 277~~ referrals resulting in no further action (i.e. not classed as in need) were excluded ~~and 634 referrals remained associated with a CIN status.~~. Overall, 323 (21.5%) of children in the BB:2-6 cohort had at least one referral to CSCS which resulted as being classed as a child in need at any point between pregnancy (includes pre-birth CIN) and 6 years of age. No difference in rate of children in need was found between FNP (21.2%) and UC (21.7%) arms for the 1,506 children in the analysis (adjusted OR=0.98, 95% CI=0.74, 1.31) (Table 2). The parameter estimate was not affected when adjusting for the number of FNP visits received (efficacy per visit OR=1.00, 95% CI=0.99, 1.004). The number of children referred to CSC and assessed as not requiring additional support were FNP (n=45) and UC (n=46). There were no between arm differences for referral to CSCS, the age at which the first referral was made, the age at which the child was classified as in need and the proportion of children with a protection plan. A small number

For peer review only - http://bmjopen.bmj.com/site/about/guidelines.xhtml

of children were ever Looked After within the 6-year follow-up period, with no evidence of a difference between trial arms (Table 2). However, the UC arm experienced on average two more months in care compared to the FNP arm. The total number of child deaths across the full follow-up period was less than ten and not further reported by study arm, consistent with the non-disclosive data management policy. Data informing the disability outcome were not available for the years requested. In addition, no difference was found when examining age at first referral or CIN by study arm.

No between arm differences were found in children not taken to at least one outpatient appointment (did not attend rate), emergency attendances or hospital admissions for an injury or an ingestion (Table 2). Overall, 78% of mothers had a subsequent pregnancy (using inpatients, outpatients and abortions data) with no differences by arm (Table 3). Similarly, just over two thirds of the cohort had at least one registerable birth (using only admission data as a source) during the follow-up period with a comparable inter-birth interval. Descriptives for non-tested maltreatment outcomes are shown in Table S6. Descriptives for the number of emergency room attendances and hospital admissions for injuries or ingestions are shown for each trial arm in Tables S7-S8. Exploratory analyses of hospital admissions for injuries and ingestions by trial arm shown by age categories (Under 1 year, 1 to 2 years, 25 months to under 6 years) are shown in Table S9.

For peer review only

Table 2 Primary and secondary outcomes: ~~Objective~~CPS outcomes, healthcare encounters for injuries / ingestions and ~~associated measures of maltreatment~~out-patient non-attendance

Outcome	FNP	Usual care (UC)	Adjusted* parameter estimate (95% CI)	p-value	Absolute risk difference (FNP-UC) (95% CI)
**Child protection outcomes**	**N=760**	**N=746**			
Child in need (CIN) status at any time during the follow-up period					
No	599 (78·8%)	584 (78·3%)	Reference		
Yes	161 (21·2%)	162 (21·7%)	0·98† (0·74 to 1·31)	0·902	-0·5% (-0·5 to 0·4%)
Age at first CIN referral (days)					
Median (25th to 75th centile)	1602·5 (1153.0 to 1978·75)	1649·5 (1130·75 to 1980·25)	0·98‡ (0·79 to 1·23)	0·875	
Missing	*1*	*0*			
Unique§ number of CIN referrals per child	1 (1 to 2)	1 (1 to 2)	0·99‖ (0·82 to 1·19)	0·890	
CIN duration (days)	210 (70·5 to 571·25)	216 (72·75 to 503·25)	1·20‡ (0·90 to 1·59)	0·216	
CIN categorisation (primary need) of first case	***N=161***	***N=162***			
Abuse or neglect	92 (57·1%)	102 (63·0%)	Reference		
Family dysfunction	29 (18·0%)	34 (21·0%)	0·95¶ (0·54 to 1·69)	0·868	
Family in acute stress	13 (8·1%)	10 (6·2%)	1·47¶ (0·61 to 3·54)	0·389	

For peer review only - http://bmjopen.bmj.com/site/about/guidelines.xhtml

**Outcome**	**FNP**	**Usual care (UC)**	**Adjusted* parameter estimate (95% CI)**	**p-value**	**Absolute risk difference (FNP-UC) (95% CI)**
Low income, absent parenting, socially unacceptable behaviour, not stated	13 (8·1%)	6 (3·7%)	2·38‖ (0·86 to 6·57)	0·093	
Child/parent disability	7 (4·3%)	5 (3·1%)	1·47¶ (0·45 to 4·84)	0·525	
Cases other than CIN	7 (4·3%)	5 (3·1%)	1·64¶ (0·50 to 5·37)	0·417	
Referred to Children's social care services (CSCS)					
No	554 (72·9%)	538 (72·1%)	Reference		
Yes	206 (27·1%)	208 (27·9%)	0·97† (0·74 to 1·28)	0·829	-0·8% (-5·3 to 3·7%)
Age at first referral (days)<br>Median (25th to 75th centiles)	1541·5 (1272·75 to 1975·75)	1631·0 (1190·5 to 1984·75)	0·96‡ (0·79 to 1·17)	0·694	
Child Protection Plan					
No	708 (93·2%)	697 (93·4%)	Reference		
Yes	52 (6·8%)	49 (6·6%)	1·04† (0·69 to 1·57)	0·846	0·2% (-0·2 to 0·3%)
Category of child protection plan	***n=52***	***n=49***			
Neglect	17 (32·7%)	22 (44·9%)	Reference		
Emotional	22 (42·3%)	12 (24·5%)	2·77‖ (1·02 to 7·56)	0·046	
Physical	6 (11·5%)	6 (12·2%)	1·25‖ (0·32 to 4·88)	0·743	
Sexual and multiple**	7 (13·5%)	9 (18·4%)	1·13‖ (0·33 to 3·86)	0·848	

For peer review only - http://bmjopen.bmj.com/site/about/guidelines.xhtml

**Outcome**	**FNP**	**Usual care (UC)**	**Adjusted* parameter estimate (95% CI)**	**p-value**	**Absolute risk difference (FNP-UC) (95% CI)**
Looked after status††					
No	735 (96·7%)	719 (96·4%)	Reference		
Yes	25 (3·3%)	27 (3·6%)	0·90† (0·52 to 1·57)	0·712	-0·3% (-0·2 to 0·2%)
Child looked after period of care (months)	10·0 (4·5 to 37·5)	12·0 (6·0 to 33·0)	0·75‖ (0·65 to 0·86)	<0·001	
**Injuries and Ingestions**	**N=760**	**N=759**			
Emergency attendance‡‡					
None	317 (41·7%)	344 (45·3%)	Reference		
At least one	443 (58·3%)	415 (54·7%)	1·17† (0·95 to 1·45)	0·149	3·6% (-1·4 to 8·6%)
Number of attendances per child	2 (1 to 2)	2 (1 to 3)	1·09‖ (0·93 to 1·28)	0·281	
Admission to hospital					
None	671 (88·3%)	660 (87·0%)	Reference		
At least one	89 (11·7%)	99 (13·0%)	0·87† (0·63 to 1·20)	0·407	-1·3% (-4·7 to 2·0%)
Number of admissions per child	1 (1 to 1)	1 (1 to 1)	0·93‖ (0·67 to 1·29)	0·663	
Attendance and/or admission					
None	306 (40·3%)	324 (52·7%)	Reference		
At least one	454 (59·7%)	435 (57·3%)	1·11† (0·89 to 1·37)	0·351	2·4% (-2·5 to 7·4%)
DNA outpatient appointment	***N=580***	***N=577***			
Attended all appointments	290 (50·0%)	289 (50·1%)	Reference		

For peer review only - http://bmjopen.bmj.com/site/about/guidelines.xhtml

Outcome	FNP	Usual care (UC)	Adjusted* parameter estimate (95% CI)	p-value	Absolute risk difference (FNP-UC) (95% CI)
DNA at least once	290 (50·0%)	288 (49·9%)	1·00† (0·79 to 1·26)	0·997	0·09% (-5·6 to 5·8%)

Data are n (%), n/N (%), or median (25th to 75th centile) unless otherwise stated. FNP = Family Nurse Partnership Programme + Usual Care. * FNP compared to usual care. Analysis adjusted for stratification (site), minimisation variables (gestational age, smoking status at recruitment, and first or preferred language); † Odds ratio from logistic model; ‡ Hazard ratio from Cox model; § A unique referral is counted as a distinct referral date per child; ‖ Incidence rate ratio from Poisson model; ¶ Relative risk ratio from multinomial model; ‡‡Using diagnosis in any position; **Multiple indicates when more than one category of abuse is relevant to the child's current plan; †† We reflect here the terminology used at the time of the trial but note the more contemporary 'care experienced' as a more familiar and accepted term. Source: National Pupil Database, The Department for Education

For peer review only

Table 3 Secondary outcomes: Subsequent pregnancies and registerable birth

	FNP	Usual care	Adjusted* parameter estimate (95% CI)	p-value	Absolute risk difference (FNP-Usual care) (95% CI)
Subsequent pregnancy	***N=753***	***N=753***			
No	163 (21·7%)	163 (21·7%)	Reference		
Yes	590 (78·4%)	590 (78·4%)	1·00† (0·79 to 1·28)	0·984	0·0% (-4·2 to 4·2%)
Subsequent registerable birth	***N=752***	***N=749***			
No	276 (36·7%)	266 (35·5%)	Reference		
Yes	476 (63·3%)	483 (64·5%)	0·95† (0·77 to 1·18)	0·662	-1·2% (-6·0 to 3·7%)
One birth	326 (68·5)	331 (68·5)	0·95‡ (0·76 to 1·19)	0·655	
Two	124 (26·1)	121 (25·1)	0·99‡ (0·73 to 1·34)	0·955	
Three births or more	26 (5·5)	31 (6·4)	0·80‡ (0·46 to 1·39)	0·435	
Inter-birth interval between first and second child (days) Median (25th to 75th centiles)	1027 (590 to 1506·75)	1065 (665 to 1538)	0·99§ (0·88 to 1·13)	0·938	

Data are n (%), n/N (%), or median (25th to 75th centile) unless otherwise stated. FNP = Family Nurse Partnership Programme + Usual Care. * FNP compared to usual care. Analysis adjusted for stratification (site), minimisation variables (gestational age, smoking status at recruitment, and first or preferred language); † Odds ratio from logistic model; ‡ Relative risk ratio from multinomial model; § Hazard ratio from Cox model.

For peer review only

Table 4 Secondary outcomes: Child health, developmental and educational

Outcome	FNP	Usual care	Adjusted* odds ratio (95% CI)	p-value	Absolute risk difference (FNP-Usual care) (95% CI)
Special educational needs (SEN)† provision	**N=759**	**N=747**			
No	540 (71·1%)	502 (67·2%)	Reference		
Yes	219 (28·9%)	245 (32·8%)	0·83 (0·67 to 1·03)	0·097	-3·9% (-8·6 to 0·7%)
**Early educational attendance**	**N=759**	**N=747**			
Attending an Ofsted registered private, voluntary and independent establishment up to the age of 4 years	334 (43·9%)	308 (41·2%)	1·13 (0·91 to 1·40)	0·281	2·7% (-2·3 to 7·7%)
**School attendance**	**N=754**	**N=740**			
Overall absence					
No absences	14 (1·9%)	14 (1·9%)	Ref		
At least one absence	740 (98·1%)	726 (98·1%)	1·00 (0·47 to 2·12)	0·998	0·0% (-1·4 to 1·5%)
Overall authorised absence					
No absences	26 (3·4%)	26 (3·5%)	Ref		
At least one absence	728 (96·6%)	714 (96·5%)	1·01 (0·58 to 1·75)	0·984	0·1% (-1·8 to 2.8%)
Overall unauthorised absence					
No absences	256 (34·0%)	245 (33·1%)	Ref		
At least one absence	498 (66·0%)	495 (66·9%)	0·95 (0·76 to 1·18)	0·620	-0·8% (-5·6 to 3·9%)

For peer review only

For peer review only - http://bmjopen.bmj.com/site/about/guidelines.xhtml

**Outcome**	**FNP**	**Usual care**	**Adjusted* odds ratio (95% CI)**	**p-value**	**Absolute risk difference (FNP-Usual care) (95% CI)**
**Early Years assessment**	**N=743**	**N=728**			
**Achieving good level of development (GLD) ‡**					
Achieving GLD in all five areas of learning‖	431 (58·0%)	380 (52·2%)	1·26 (1·03 to 1·55)	0·026	5·8% (0·7 to 10·9%)
Achieving GLD in all 17 early learning goals	412 (55·5%)	365 (50·1%)	1·24 (1·01 to 1·52)	0·043	5·3% (0·2 to 10·4%)
**Total point score**§ Mean (SD)	32·22 (7·25)	31·59 (7·62)	0·65 ‖ (-0·11 to 1·41)	0·094	
**Key Stage 1 assessments**	**N=740**	**N=732**			
**Reading**					
Lower than expected	257 (34·7%)	289 (39·5%)	Reference		
Reaching at least the expected standard¶	483 (65·3%)	443 (60·5%)	1·23 (0·99 to 1·53)	0·051	4·8 (-0·2 to 9·7%)
Expected standard	*371 (50·1%)*	*337 (46·0%)*	*1·24 (0·99 to 1·56)*	*0·056*	*4·1 (-1·0 to 9·2%)*
Higher standard	*112 (15·1%)*	*106 (14·5%)*	*1·20 (0·88 to 1·65)*	*0·250*	*0·7 (-3·0 to 4·3%)*
**Maths**					
Lower than expected	281 (38·0%)	283 (38·7%)	Reference		
Reaching at least the expected standard¶	459 (62·0%)	449 (61·3%)	1·04 (0·84 to 1·28)	0·731	0·7 (-4·3 to 5·6%)
Expected standard	*392 (53·0%)*	*376 (51·4%)*	*1·06 (0·85 to 1·32)*	*0·611*	*1·6 (-3·5 to 6·7%)*
Higher standard	*67 (9·1%)*	*73 (10·0%)*	*0·93 (0·64 to 1·35)*	*0·711*	*-0·9 (-3·9 to 2·1%)*

For peer review only - http://bmjopen.bmj.com/site/about/guidelines.xhtml

Outcome	FNP	Usual care	Adjusted* odds ratio (95% CI)	p-value	Absolute risk difference (FNP-Usual care) (95% CI)
**Science**					
Lower than expected	203 (27·4%)	219 (29·9%)	Reference		
Reaching at least the expected standard**	537 (72·6%)	513 (70·1%)	1·14 (0·91 to 1·43)	0·254	2·5 (-2·1 to 7·1%)
**Writing academic year 2016/17**	**N=498**	**N=487**			
Lower than expected	257 (51·6%)	278 (57·1%)	Reference		
Reaching at least the expected standard¶	241 (48·4%)	209 (42·9%)	1·24 (0·97 to 1·60)	0·090	5·5 (-0·7 to 11·6%)
Expected standard	*218 (43·8%)*	*182 (37·4%)*	*1·29 (1·00 to 1·68)*	*0·054*	*6·4 (0·3 to 12·5%)*
Higher standard	*23 (4·6%)*	*27 (5·5%)*	*0·92 (0·51 to 1·64)*	*0·769*	*-0·9 (-3·8 to 1·9%)*

Data are n (%), n/N (%), or median (25th to 75th centile) unless otherwise stated. FNP = Family Nurse Partnership Programme + Usual Care. SD=standard deviation. *FNP compared to usual care. Analysis adjusted for stratification (site), minimisation variables (gestational age, smoking status at recruitment, and first or preferred language); † A child with SEN provision is recorded using the following codes (A = School Action, P = School Action Plus, E = Education, Health and Care Plan, S=Statement, K= SEN support) and the N code was used to indicate No SEN support in any of the following datasets between 2013 and 2017. PLASC (Autumn, Spring and Summer terms), AP, PRU Census, and EYC. If for any of the years no response was recorded then the assumption was that the child was not included in the denominator as either not present on Census day or not in school; ‡ Children achieving a good level of development are those achieving at least the expected level within the prime and specific areas of learning; §Total point score ranges from 17 to 51 with a higher score indicating a better level of development; ∥ Mean difference from linear model; ¶ Working at the expected standard and at a greater depth within the expected standard; **Working at a greater depth within the expected standard is not applicable in science. Source: National Pupil Database, The Department for Education

For peer review only - http://bmjopen.bmj.com/site/about/guidelines.xhtml

Evidence of positive programme impacts were found for some educational outcomes assessed at the end of the reception year and at KS1 by which time the child would be seven years old (Table 4). Children in the FNP arm were more likely to reach a good level of development across all five areas of learning by the end of the reception year (58.0%) than children in the UC arm (52.2%) and also to achieve a good level of development in all 17 early learning goals (FNP: 55.5%, UC: 50.1%). The small advantage for children of nurse-visited mothers was consistent across all five areas of learning although not tested statistically (Table ~~S7~~S10).

For KS1 reading, writing, maths and science assessments there was no evidence of a difference between arms in the proportion of children reaching at least the expected standard nor specifically in those working at the expected level or at a greater depth of knowledge (Table 4). What is observed from the writing assessment is the increase in the rates of children reaching at least the expected standard between the two academic years (Overall 45.7% in 2016/17 to 66.5% in 2017/18), reflective of all assessments across the two academic years in each arm (Table ~~S8~~S11). This is mainly explained by the distribution of births for the children for each academic year (Figure S1). Reflecting the recruitment period of the trial, children with KS1 results available in the academic year 2016/17 were born between October 2009 and August 2010 but skewed towards the summer born children (42% born between June and August 2010) whereas children appearing in the 2017/18 academic year were born between September 2010 and February 2011. There is a nationally observed relationship between rates of children reaching the expected standard and month of birth e.g. a 17% difference in reading for children born in August and September~~.~~.[16] Therefore, our 2017/18 cohort of children do not represent the whole academic year, but are biased towards those that are more likely to achieve. This indicates that the rates of children reaching the expected standard varies by their month of birth and is important as a moderator of programme effect. Importantly, the original trial allocation ensured study arms remained balanced with regards month of birth and the intervention had no impact on total weeks gestation.[~~6~~5] As a sensitivity analysis, the main analyses were adjusted for the child's month of birth (categorised into quarter of birth) and evidence of a between arm difference was found in reading assessments (Table ~~S9~~S12). In addition, the Early Years assessments were adjusted for child's month of birth, which strengthened the association found in Table 4.

Details of all planned sub-group analyses and further exploratory analyses are shown in the appendix (Tables ~~S10~~S13 to ~~S14~~S17).

Cost

There were negligible resource use and cost differences for both mothers (p=0.393) and children (p=0.865) between study arms (Table ~~S15~~S18). The adjusted incremental costs of programme delivery per women in BB:0-2 (£1,811) remain the key observed cost difference between study arms.

For peer review only - http://bmjopen.bmj.com/site/about/guidelines.xhtml

Discussion

Principal findings

In this data linkage study we found that adding FNP to usually provided health and social care provided no additional benefit for maltreatment outcomes including the numbers of children referred to CSCS, registered as in need of additional support, given a child protection plan, entering care, attending an emergency department or being admitted for an injury or ingestion, or duration of stay if admitted. While there was no difference between study arms in the small number of children in care, the UC arm experienced on average two more months in care compared to the FNP arm. The total number of child deaths found was less than ten and therefore disclosive and cannot be reported. High rates of children ever registered as in need in both study arms indicates substantial need amongst families. We found the programme led to more children reaching a good level of development at the end of school reception year and, when taking month of birth into account, improvements in reading at KS1. Writing scores improved as a result of FNP for boys, children of younger mothers and of mothers who were not in education, employment or training when first recruited to the study. We found no other difference between families who received FNP and those who did not. With no differences in secondary health resource use and costs for FNP, the programme can be considered cost-neutral compared to usual care in the medium-term (2-6 years).

Strengths and weaknesses of the study

The prioritisation of outcomes in our original trial attracted some debate..[17-19] However, the trial and the current follow-on study are both comprehensively reported, and preceded by a priori analysis plans providing transparency and guarding against undue post hoc interpretation..[6-8] Although a primary outcome of child being in need of additional support was incorporated into the study design, we adopted a multimethod multisource approach to look at the range of maltreatment outcomes in drawing our conclusions. The consistent pattern of results within the two principal outcome domains provides greater confidence in what we can conclude. While the study benefited from random allocation in the BB:0-2 trial, when assessing educational outcomes we additionally adjusted for month of birth. This was because at a national level, month of birth is strongly associated with educational outcomes and the distribution of participants recruited to the trial varied by month. ~~The potential to show benefit associated with additional support therefore differed by actual month of birth.~~

The available maltreatment data provides an essential overview of family experience as recorded in official records but may lack some depth which could have allowed a more nuanced understanding for children referred. The educational data are teacher-reported statutory tests but similarly provide less detailed insight into child functioning which research instruments may have provided. The lack of

For peer review only - http://bmjopen.bmj.com/site/about/guidelines.xhtml

detailed social care and any primary care resource use data limited the perspective of the economic analysis. However, using administrative data has enabled comprehensive cohort follow-up, with few cases lost for analysis preserving the benefits of the original trial randomisation. The data included are also objectively recorded rather than maternally self-reported, allow for easy comparison nationally and over time. Our approach has gathered more data at less cost than comparable prospective data collection and minimised family burden.

In our previous trial report of outcomes at 24 months postpartum, there were higher rates of safeguarding events recorded in GP notes for children in the FNP arm (n=64/469, 13.6%), compared to the usual care arm (n=38/476, 8.0%).[5] Additionally, more mothers in the FNP arm reported their child had ever been referred to social services (n=119/580, 20.5%) compared to mothers in the usual care arm (n=91/541, 16.8%). These finding can be indicative of surveillance bias or more accurate reporting by mothers in the FNP arm respectively. In the current study a tendency for children to be referred at lower thresholds of concern or closer involvement by attending FNP nurses may have been evidenced through a greater proportion of children referred to social services being assessed as requiring no additional support. However, we did not observe this in our cohort.

With no absolute external criterion for maltreatment, a reliance solely upon formal child protection reports may be limiting. In the US Elmira trial, a review of 42 children (intervention arm n=13, control arm n=29) referred to CPS between birth and their fourth year of life found few differences in the nature of maltreatment recorded. However, measures of the quality of home environment, parenting, child intellectual functioning and use of healthcare services between children's 25th and 50th months of life favoured families in the intervention arm.[20] It has been suggested that both parental and professional behaviour may lead to programme enrolled CPS reported children being at lower risk of harm than their counterparts in receipt of usual care alone. Thresholds for referral to social services may differ between specialist home-visiting nurses with high contact time, more in-depth relationship with clients and different approaches to risk and safety management and other health, social and educational care professionals.[21] CPS referrals for children in receipt of FNP at lower levels of apparent concern would make it harder to compare on such outcomes, particularly during the programme delivery period (ie up to the child's second birthday). However, subsequent progress of children through the child protection system and later occurring outcomes (for example, children being taken into care) may be less liable to such influences but in our sample such numbers are small.

Verified outcomes documented in administrative records contrast with maternal subjective self-reports for which there may be no potential external criterion (ie a child being referred to CPS is a verifiable event, whereas maternal well-being is not). Nevertheless, the presentation or referral of families and their assessment against legally defined statuses such as being a Child in Need requires

For peer review only - http://bmjopen.bmj.com/site/about/guidelines.xhtml

professional judgement. It has been suggested that surveillance bias is not likely to be problematic for assessing maltreatment using verified service data (i.e. by inflating the numbers of children being reported when in receipt of home-visiting).[22-23] As others have found this could be higher when clients are actively engaged with services and as noted above, the potential for such bias remains.[24]

It has been proposed that other documented evidence such as hospital admission due to injury and ingestions and in particular duration of subsequent stay may provide a better indicator of maltreatment severity. In the follow-up of the US Memphis trial cohort at two years of the child's life, children in the intervention arm admitted following an injury or ingestion spent fewer days in hospital than their counterparts in the control arm. In the US Elmira trial, no difference between trial arms were found in the number of days hospitalised during the 25- to 50-month period of follow-up after adjustments were made for one outlier (in the intervention arm) assessed as being not relevant to parental caregiving.[25] We explored length of hospital stay following admission for an injury and ingestion both overall and for time periods representing periods of increasing of child mobility (ie under 1 year of age, 1 to under 2 years of age and 25 months to under 6 years of age). These were similar in each case but factors other than severity of clinical need may influence how long children remain in hospital. Wide differences exist between countries for hospital stay duration following injuries, ingestions and other consequences of external causation (when assessed across adult and paediatric populations).[26] One observational UK study of predictors of length of stay amongst paediatric inpatients (all causes) shows the contribution of ,health service factors in addition to clinical and social factors.[27] Interestingly, a large US study amongst paediatric inpatients found that non-accidental trauma compared to accidental trauma was a strong predictor of having a prolonged hospital stay even after adjusting for injury severity.[28]Therefore, length of hospital stay may be indicative of maltreatment, severity of clinical presentation, health service factors and possibly social factors. Further clarifying the factors that independently drive length of hospital stay and the validity of this outcome as a measure of maltreatment will be valuable.

Potential sources of bias

As an open label study, it is possible that service professionals may have attempted to compensate for the lack of specialist support from FNP for participants in the usual care arm. However, it should be noted that enhanced care would have been expected as part of usual practice for teenage mothers, for example, through teenage pregnancy midwives. The number of midwifery contacts were recorded in the trial and found to be equivalent between FNP and UC arms.[109] The number of home visits from specialist public health nurses (SPHN) reported by trial participants by 18 months was also equivalent between study arms. As would have been expected, the number of clinic contacts with SPHNs was greater for women in the control arm than in the FNP arm. In contrast, women allocated to the intervention arm would have received an average of just over 40 home visits from a family nurse

For peer review only

For peer review only - http://bmjopen.bmj.com/site/about/guidelines.xhtml

before graduating from the programme. Overall, this suggests a common core of usual care provided across both trial arms, a higher level of SPHN input to the usual care arm reflecting in part the responsibility of delivering the Healthy Child Programme (which was formally allocated to FNP nurses rather than SPHNs for their clients). The capacity of SPHNs to provide additional support will also be limited by their caseload. The maximum caseload currently recommended by the Institute of Health Visiting is one practitioner per 250 children. However, in 2015, 28% of surveyed practitioners in England reported caseloads in excess of 400 children and 12% reported between 500 and 1000+ families.~~[21]~~[29]

~~In categorising our outcomes we delineated between those which were objective indicators of maltreatment and other markers. This was to recognise those outcomes assessed independently of maternal report or presentation by third party agents - specifically local departments of children social services. However, both the presentation or referral of families and their assessment against legally defined statuses such as being a Child in Need still involves interpretation and remains a potential source of bias. It has been suggested that surveillance bias is not likely to be problematic for assessing maltreatment using verified service data (i.e. by inflating the numbers of children being reported when in receipt of home-visiting).[22-23] As others have found this could be higher when clients are actively engaged with services; the potential for such bias still needs to be acknowledged.[24]~~

Comparison with other studies

The strongest evidence for the programme preventing maltreatment remains from the original US Elmira trial. While the programme reduced the number of substantiated reports of abuse and neglect in the first 15 years of the child's life, the advantage emerged after age four years~~.~~.[30] We found differences in duration spent in care for looked after children, but the numbers involved are small and across the range of maltreatment outcomes the pattern is of no overall group difference. The programme was no more effective in tested sub-groups. It is worth recognising differences in the population of clients in receipt of the programme across the different study populations, their social circumstances (which also may have changed over time) and how that may determine potential to benefit from FNP.~~[10]~~[9] We have not directly explored differences in social care systems between England and the US but these may vary the underlying risk of maltreatment and its likelihood of detection~~.~~.[31]

School entry reading and maths is of important predictive value for later school achievement~~.~~.[32] Early childhood educational investment programmes typically show a modest impact upon cognitive and achievement scores (~~eg~~e.g. weighted average of 0.21 standard deviations) with some decline in observed impact being attributed to general improvements in both school and home environments~~.~~.[33,34] The question of comparator is therefore of particular relevance. In this study, a

For peer review only - http://bmjopen.bmj.com/site/about/guidelines.xhtml

range of state and third sector funded services (including midwifery and health visiting) would have been available as usual care, possibly limiting the incremental benefit possible from the family nurse partnership programme.[109] Nevertheless, as noted above women allocated to FNP received substantial input from family nurses, albeit fewer than possible according to the maximum fidelity targets at the time of the trial.

Home visiting interventions, which aim to impact upon a range of outcomes, have shown similarly modest effects on child cognitive outcomes (ege.g. mean Effect Size 0.25, 96% CI 0.11, 0.38) with considerable variability due to programme composition (ege.g. greater effects with programmes addressing parental responsiveness, sensitivity to care and nurturing and rehearsal and role-playing).[3135] Our BB:0-2 study found benefits at age two years for children in the FNP arm for both language and developmental outcomes but these outcomes were maternally reported and potentially subject to reporting bias in the open trial..[8] This new objectively obtained medium-term evidence for programme impact upon developmental outcomes adds to that picture, although the size of effect remains small. Similar medium-term programme impact (to age six years old) on developmental outcomes (eg, intellectual functioning and receptive language scores) was reported from the Memphis trial.[3236] A reanalysis of those data found cognitive benefits at age 6 years attributed to both programme induced improvements in maternal traits and family life investments at age 2.[3337]

Unanswered questions and future research

Given small but positive findings for developmental outcomes, tracking the cohort through to subsequent school years is warranted. As social care data are equally accessible via the NPD there is value in observing the trajectory for maltreatment outcomes given that the principal maltreatment findings from the US Elmira trial are located in the 15-year follow-up. Linkage to data in other sectors such as welfare would provide a greater understanding of broader impact for families. Such work should be driven by the programme's logic model, existing evidence for long-term programme impact, informed by programme evolution in England and supplemented by work to understand what may explain differing programme benefits across national settings.[26,3430,38]

Conclusions

There are no evident programme benefits for maltreatment outcomes by age six but the FNP generates higher rates of child attainment at the end of the reception year and at KS1. The adapted programme remains locally commissioned and delivered in England.[34,3538,39] Local needs and priorities may determine the weight attached to these different sets of outcomes.

For peer review only - http://bmjopen.bmj.com/site/about/guidelines.xhtml

Acknowledgements

The Centre for Trials Research receives funding from Health and Care Research Wales and Cancer Research UK. The work was undertaken with the support of The Centre for the Development and Evaluation of Complex Interventions for Public Health Improvement (DECIPHer), a UKCRC Public Health Research Centre of Excellence. Joint funding (MR/KO232331/1) from the British Heart Foundation, Cancer Research UK, Economic and Social Research Council, Medical Research Council, the Welsh Government and the Wellcome Trust, under the auspices of the UK Clinical Research Collaboration, is gratefully acknowledged.

Data re-use statement: Copyright © (2019), the Health and Social Care Information Centre. Re-used with the permission of the Health and Social Care Information Centre. All rights reserved.

We would like to thank all members of CASCADE voices, ALPHA and Our Place for contributing to the study including those who co-ordinated access to these individuals. Thanks are due to the FNP teams in Bristol, Bath and Wiltshire who helped us to make contact with FNP clients and graduates during our PPI work. We would also like to acknowledge the contribution of members of the study steering committee - Ann Louise Kinmonth (Chair), David Low, Gordon Taylor, Nina Biehal, Rachel Tonkin, Stavros Petrou, and Silvia van den Heijkant.

This work uses data provided by patients and collected by the NHS as part of their care and support as well as data from schools and local authorities. Using patient and other public data is vital to improve health and care for everyone. There is huge potential to make better use of information from people's patient records, to understand more about disease, develop new treatments, monitor safety, and plan NHS services. Patient and other public data should be kept safe and secure, to protect everyone's privacy, and it's important that there are safeguards to make sure that it is stored and used responsibly. Everyone should be able to find out about how patient data is used. #datasaveslives You can find out more about the background to this citation here: https://understandingpatientdata.org.uk/data-citation. [To note. This has been adapted to include other data sources not made available from the NHS such as education and social services data].

Footnotes

Author contributions: MR was the chief investigator and acts as guarantor for the study. MR, FLW, RCJ, LA, SC, DF, KH, JK, GM, EOJ, RP, JSa, JSe, TS contributed to study design. FLW was the study manager. RCJ was the lead statistician. DF supervised the health economics. MR, FLW, RCJ, LA, SC, DF, KH, JK, GM, EOJ, RP, JSa, JSe, TS were responsible for study management. GM and LA were the data managers. JK and JS led for public involvement. GM and LA cleaned the data. RCJ designed and conducted the analysis. RP conducted the economic analysis. MR, JSa, RCJ, SC, KH,

For peer review only - http://bmjopen.bmj.com/site/about/guidelines.xhtml

JK, EOJ, JSe were co-applicants on the original funding application. MR, FLW, RCJ, LA, SC, DF, KH, JK, GM, EOJ, RP, JSa, JSe, TS interpreted the results of the analysis. RC-J and RP were responsible for producing the tables. MR, FLW, RC-J and RP drafted the study report. MR, FLW, RCJ, LA, SC, DF, KH, JK, GM, EOJ, RP, JSa, JSe, TS reviewed manuscript drafts, revised for important intellectual content and approved the final version.

Funding: This project was funded by the National Institute for Health Research Public Health Research (NIHR PHR) Programme (reference:11/3002/11). The views and opinions expressed therein are those of the authors and do not necessarily reflect those of the NIHR PHR Programme or the Department of Health. The funders had no role in considering the study design or in the collection, analysis, interpretation of data, writing of the report, or decision to submit the article for publication.

Competing interests: All authors have completed the ICMJE uniform disclosure form at www.icmje.org/coi_disclosure.pdf and declare: all authors declare financial support from NIHR PHR for the submitted work; no financial relationships with any organisations that might have an interest in the submitted work in the previous three years; no other relationships or activities that could appear to have influenced the submitted work.

Ethical approval: Ethical approval for this study was granted by the Health Research Authority research ethics committee (Wales REC 3; REC reference 14/WA10062) and section 251 support was provided by the Confidentiality Advisory Group (CAG reference CAG 10-08(b)/2014).

Data sharing: Anyone wishing to access the linked datasets for research purposes should apply via the CAG to the Health Research Authority to access patient identifiable data without consent and then to the NHS Digital, National Pupil Database and the abortion statistics – Department for Health and Social Care. In the first instance, enquiries about access to the data should be addressed to Professor Mike Robling, Centre for Trials Research, Cardiff University.

The lead author (MR) affirms that the manuscript is an honest, accurate, and transparent account of the study being reported; that no important aspects of the study have been omitted; and that any discrepancies from the study as planned (and, if relevant, registered) have been explained.

Dissemination to participants and related public communities: The findings from the BB:2-6 study, including those from this paper will be disseminated directly to participants where valid contact details remain available, via social media and conference presentations. This will include the use of lay-informed infographic materials. Summary results will also be made available on the study webpage [https://evidencinghomevisiting.co.uk/].

For peer review only

References

1. Olds DL, Henderson CR, Chamberlin R, Tatelbaum R. Preventing Child Abuse and Neglect: A Randomized Trial of Nurse Home Visitation. Pediatrics. 1986;78(1) [accessed 2017 May 5]. Available from: http://pediatrics.aappublications.org/content/pediatrics/78/1/65.full.pdf

2. Kitzman H, Olds DL, Henderson CR, Hanks C, Cole R, Tatelbaum R, et al. Effect of Prenatal and Infancy Home Visitation by Nurses on Pregnancy Outcomes, Childhood Injuries, and Repeated Childbearing. JAMA. 1997;278(8):644 [accessed 2017 May 5]. Available from: http://jama.jamanetwork.com/article.aspx?doi=10.1001/jama.1997.03550080054039

3. Olds DL, Robinson J, O'brien R, Luckey DW, Pettitt LM, Henderson CR, et al. Home Visiting by Paraprofessionals and by Nurses: A Randomized, Controlled Trial. Pediatrics. 2002;110(3) [accessed 2017 May 5]. Available from: http://pediatrics.aappublications.org/content/110/3/486.short

4. Mejdoubi J, van den Heijkant ~~SCCM~~SC, van Leerdam ~~FJM~~FJ, Heymans MW, Crijnen A, Hirasing RA. The effect of VoorZorg, the Dutch nurse~~-~~–family partnership, on child maltreatment and development: a randomized controlled trial. PLOS ONE~~.~~ 2015;10~~(4).~~:e0120182. https://doi.org/10.1371/journal.pone.0120182

5~~. Olds DL, Kitzman H, Anson E, Smith JA, Knudtson MD, Miller T, et al. Prenatal and infancy nurse home visiting effects on mothers: 18-year follow-up of a randomized trial. Pediatrics. 2019;144(6) [accessed 2020 Jul 23]. Available from: https://pediatrics.aappublications.org/content/144/6/e20183889~~

~~6~~. Robling M, Bekkers MJ, Bell K, Butler CC, Cannings-John R, Channon S, et al. Effectiveness of a nurse-led intensive home-visitation programme for first-time teenage mothers (Building Blocks): A pragmatic randomised controlled trial. Lancet. 2016;387:146–55. Available from: http://www.thelancet.com/journals/lancet/article/PIIS0140-6736(15)00392-X/fulltext

~~7~~6. Lugg-Widger F, Cannings-John R, Channon Sue, Fitzsimmons D, Hood K, Jones K, et al. Assessing the medium-term impact of a home-visiting programme on child maltreatment in England: protocol for a routine data linkage study. BMJ Open. 2017;7(e015728) [accessed 2017 Jul 17]. Available from: http://bmjopen.bmj.com/content/bmjopen/7/7/e015728.full.pdf

~~8~~7. Robling M, Bekkers MJ, Bell K, Butler CC, Cannings-John R, Channon S, et al. The Building Blocks Trial (Main report). 2014. Available from: https://www.cardiff.ac.uk/__data/assets/pdf_file/0009/504729/Building-Blocks-Full-Study-Report.pdf

~~9~~8. Owen-Jones E, Bekkers M-J, Butler CC, Cannings-John R, Channon S, Hood K, et al. The effectiveness and cost-effectiveness of the Family Nurse Partnership home visiting programme for first time teenage mothers in England: a protocol for the Building Blocks randomised controlled trial. BMC Pediatr. 2013;13:1.

~~10~~9. Robling M, Cannings-John R, Channon S, Hood K, Moody G, Poole R, et al. What is usual care for teenagers expecting their first child in England? A process evaluation using key informant mapping and participant survey as part of the Building Blocks randomised controlled trial of specialist home visiting. BMJ Open. 2018;8(5).

~~11~~10. Lyons RA, Jones KH, John G, Brooks CJ, Verplancke J-P, Ford D V, et al. The SAIL databank: linking multiple health and social care datasets. BMC Med Inform Decis Mak. 2009;9(9) [accessed 2017 May 12]. Available from: http://www.biomedcentral.com/1472-6947/9/3

For peer review only

~~12~~11. Jones KH, Ford D V., Jones C, Dsilva R, Thompson S, Brooks CJ, et al. A case study of the Secure Anonymous Information Linkage (SAIL) Gateway: A privacy-protecting remote access system for health-related research and evaluation. J Biomed Inform. 2014;50:196–204 [accessed 2017 May 12]. Available from: http://www.sciencedirect.com/science/article/pii/S1532046414000045

~~13~~12. Jones KH, Ford DV, Thompson S, Lyons R. A Profile of the SAIL Databank on the UK Secure Research Platform. Int J Popul Data Sci. 2019;4(2) [accessed 2020 Jun 30]. Available from: https://ijpds.org/article/view/1134

~~14~~13. Department for Education. Children In Need in England, including their characteristics and further information on children who were the subject of a child protection plan (2009-10 Children in Need census, Final. 2010.

~~15~~14. HM Treasury. The Green Book, Central Government Guidance on Appraisal and Evaluation. 2020. [accessed 2020 Dec 7]. Available from: https://assets.publishing.service.gov.uk/government/uploads/system/uploads/attachment_data/file/938046/The_Green_Book_2020.pdf

~~16~~15. Lugg-widger F, Cannings-john R, Angel L, Moody G, Segrott J, Kenkre J, et al. Assessing the impact of specialist home visiting upon maltreatment in England : a feasibility study of data linkage from a public health trial to routine health and social care data. Pilot feasibility Stud. 2018;4(1):98.

~~17~~16. Department for Education. Phonics screening check and ~~Kay~~Key stage 1 assessments in England, 2016 (SFR 42/2016). London: Department for Education; 2016.

~~18~~17. Olds D. Building evidence to improve maternal and child health. Vol. 387, The Lancet. Lancet Publishing Group; 2016. p. 105–7 [accessed 2020 Jul 23]. Available from: http://dx.doi.org/10.1016/S0140-6736

~~19~~18. Barlow J, Barnes J, Sylva K, Fonagy P, Fearon P. Questioning the outcome of the Building Blocks trial. Vol. 387, The Lancet. Lancet Publishing Group; 2016. p. 1615–6 [accessed 2020 Jul 23]. Available from: http://www.dh.gov.uk/

~~20.~~19. Robling M, Torgerson D, Pickett K, Channon S, Sanders J. Questioning the outcome of the Building Blocks trial - Authors' reply. Vol. 387, The Lancet. Lancet Publishing Group; 2016. p. 1616–7 [accessed 2020 Jul 23]. Available from: http://www.dh.gov.uk/~~http://www.dh.gov.uk/~~

20. Olds D, Henderson CR, Kitzman H, Cole R. Effects of prenatal and infancy nurse home visitation on surveillance of child maltreatment. Pediatrics 1995; 95; 365-372.

21. Williams, VN, Ayele, R, Shimasaki, S, Tung, GJ, Olds, D. "Risk assessment practices among home visiting nurses and child protection caseworkers in Colorado, United States: A qualitative investigation." Health & Social Care in the Community. 2019;27(5): 1344-52. doi.org/10.1111/hsc.12773

22. Drake B, Jonson-Reid M, Kim H. Surveillance bias in child maltreatment: a tempest in a teapot. Int J Environ Res Public Health 2017;14:E971. https://doi.org/10.3390/ijerph140909712~~25~~

23. Chaffin M, Bard D. Impact of intervention surveillance bias on analyses of child welfare report outcomes. Child Maltreat 2006;11:301–12. https://doi.org/10.1177/107755950629

24. Zielinski DS, Eckenrode J, Olds DL. Nurse home visitation and the prevention of child maltreatment: Impact on the timing of official reports. Dev Psychopathol. 2009;21(2):441–53 [accessed 2019 Aug 2]. Available from:

For peer review only

https://www.cambridge.org/core/product/identifier/S0954579409000248/type/journal_articlehttps://www.cambridge.org/core/product/identifier/S0954579409000248/type/journal_article

25. Olds DL, Henderson Jr CR, Kitzman H. Does prenatal and infancy nurse home visitation have enduring effects on qualities of parental caregiving and child health at 25 to 50 months of Life? Pediatrics 1994; 93; 89-98.

26. https://ec.europa.eu/eurostat/statistics-explained/index.php?title=Hospital_discharges_and_length_of_stay_statistics#Average_length_of_hospital_stay_for_in-patients Date accessed: 30.7.21

27. Heys M, Rajan M, Blair M. Length of paediatric inpatient stay, socio-economic status and hospital configuration: a retrospective cohort study. BMC Health Services Research 2017 17: 274 DOI 10.1186/s12913-017-2171-x

28. Livingston JK, Grigorian A, Kuza CM, Lekawa M, Bernal N, Allen A, Nahmais J. Non-accidental trauma increases length of stay and mortality in pediatric trauma. Pediatric Surgery International (2019) 35:779–784 doi.org/10.1007/s00383-019-04482-5

29. Institute of Health Visiting. Health Visiting in England: State of Health Visiting in England: Results from a survey of 1040 practising health visitors. 2020 [accessed 2021 May 21]. Available from: https://www.activematters.org/wp-content/uploads/2020/03/State_of_Health_Visiting_survey.pdf26

30. Olds DL, Eckenrode J, Henderson CR, Kitzman H, Powers J, Cole R, et al. Long-term Effects of Home Visitation on Maternal Life Course and Child Abuse and Neglect Fifteen-Year Follow-up of a Randomized Trial. JAMA. 1997;278(8):637–43.

27. 31. Moody G, Cannings-John R, Hood K, Kemp A, Robling M. Establishing the international prevalence of self-reported child maltreatment: A systematic review by maltreatment type and gender. Vol. 18, BMC Public Health. BioMed Central Ltd.; 2018. p. 1164 [accessed 2020 Jul 23]. Available from: https://bmcpublichealth.biomedcentral.com/articles/10.1186/s12889-018-6044-y

28. 32. Duncan GJ, Dowsett CJ, Claessens A, Magnuson K, Huston AC, Klebanov P, et al. School Readiness and Later Achievement. Dev Psychol. 2007;43(6):1428–46.

2933. Duncan G, Magnusson K. Early childhood interventions for low-income children. Focus (Madison). 2014;31.

3034. Duncan G, Magnuson K. Investing in Preschool Programs. J Econ Perspect. 2013;27(2):109–31.

3135. Filene JH, Kaminski JW, Valle LA, Cachat P. Components associated with home visiting program outcomes: A meta-analysis. Pediatrics. 2013;132(SUPPL.2):S100–9 [accessed 2021 Jan 8]. Available from: www.pediatrics.org/cgi/doi/10.1542/peds.2013-1021H

3236. Olds DL, Kitzman H, Cole R, Robinson J, Sidora K, Luckey DW, et al. Effects of Nurse Home-Visiting on Maternal Life Course and Child Development: Age 6 Follow-Up Results of a Randomized Trial. Pediatrics. 2004;114(6):1550–9. Available from: http://pediatrics.aappublications.org/cgi/doi/10.1542/peds.2004-0962

3337. Heckman JJ, Holland ML, Makino KK, Pinto R, Rosales-Rueda M. An Analysis of the Memphis Nurse-Family Partnership Program. NBER Work Pap. 2017;23610. Available from: http://www.nber.org/papers/w23610%0Ahttp://www.nber.org/papers/w23610

For peer review only - http://bmjopen.bmj.com/site/about/guidelines.xhtml

~~34~~38. Family Nurse Partnership National Unit & Dartington Service Design Lab. FNP ADAPT: Using evidence, pragmatism and collaboration to change the FNP programme in England. London, UK; 2020. Available from: www.fnp.nhs.uk

~~35~~39. The Family Nurse Partnership |. [accessed 2020 Jul 23]. Available from: https://www.fnp.nhs.uk/

For peer review only

Figure Legend

Figure 1 Participant flow chart

Describing follow-up within the BB0:0-2 trial and formation of the BB:2-6 study cohort. MW=Mandatory withdrawals; EW=elective withdrawals; M=Mother; C=Child, FNP=Family Nurse Partnership (trial arm); UC=Usual care (trial arm); BB:0-2=Building Blocks Randomised Controlled Trial; BB:2-6=Building Blocks Cohort Study; NPD=National Pupil Database

For peer review only

BMJ Open

A nurse-led home-visitation programme for first-time mothers in reducing maltreatment and improving child health and development (BB:2-6): longer-term outcomes from a randomised cohort using data-linkage.

Journal:	*BMJ Open*
Manuscript ID	bmjopen-2021-049960.R3
Article Type:	Original research
Date Submitted by the Author:	08-Nov-2021
Complete List of Authors:	Robling, Michael; Cardiff University, Centre for Trials Research; DECIPHer, Cardiff University School of Social Sciences<br>Lugg-Widger, Fiona; Cardiff University, Centre for Trials Research<br>Cannings-John, Rebecca; Cardiff University Centre for Trials Research,<br>Angel, Lianna; Cardiff University, Centre for Trials Research<br>Channon, Sue; Cardiff University, Centre for Trials Research<br>Fitzsimmons, Deborah; Swansea University, College of Human and Health Sciences<br>Hood, Kerenza; Cardiff University,<br>Kenkre, Joyce; University of South Wales, Faculty of Life Sciences and Education<br>Moody, Gwenllian; Cardiff University, Centre for Trials Research<br>Owen-Jones, Eleri; Cardiff University, Centre for Trials Research<br>Pockett, Rhys; Swansea University, Swansea Centre for Health Economics<br>Sanders, Julia; Cardiff University, School of Healthcare Sciences<br>Segrott, Jeremy; Cardiff University, Centre for Trials Research; Cardiff University, DECIPHer Centre, Cardiff School of Social Sciences<br>Slater, Thomas; Cardiff University, School of Social Sciences
**Primary Subject Heading**:	Public health
Secondary Subject Heading:	Nursing
Keywords:	PUBLIC HEALTH, Child protection < PAEDIATRICS, PREVENTIVE MEDICINE

SCHOLARONE™
Manuscripts

I, the Submitting Author has the right to grant and does grant on behalf of all authors of the Work (as defined in the below author licence), an exclusive licence and/or a non-exclusive licence for contributions from authors who are: i) UK Crown employees; ii) where BMJ has agreed a CC-BY licence shall apply, and/or iii) in accordance with the terms applicable for US Federal Government officers or employees acting as part of their official duties; on a worldwide, perpetual, irrevocable, royalty-free basis to BMJ Publishing Group Ltd ("BMJ") its licensees and where the relevant Journal is co-owned by BMJ to the co-owners of the Journal, to publish the Work in this journal and any other BMJ products and to exploit all rights, as set out in our licence.

The Submitting Author accepts and understands that any supply made under these terms is made by BMJ to the Submitting Author unless you are acting as an employee on behalf of your employer or a postgraduate student of an affiliated institution which is paying any applicable article publishing charge ("APC") for Open Access articles. Where the Submitting Author wishes to make the Work available on an Open Access basis (and intends to pay the relevant APC), the terms of reuse of such Open Access shall be governed by a Creative Commons licence – details of these licences and which Creative Commons licence will apply to this Work are set out in our licence referred to above.

Other than as permitted in any relevant BMJ Author's Self Archiving Policies, I confirm this Work has not been accepted for publication elsewhere, is not being considered for publication elsewhere and does not duplicate material already published. I confirm all authors consent to publication of this Work and authorise the granting of this licence.

For peer review only

Title: A nurse-led home-visitation programme for first-time mothers in reducing maltreatment and improving child health and development (BB:2-6): longer-term outcomes from a randomised cohort using data-linkage

Authorship:

Michael Robling, [1,2] (Director of Population Health Trials), RoblingMR@cardiff.ac.uk

Fiona Lugg-Widger, [1] (Research Fellow), LuggFV@cardiff.ac.uk

Rebecca Cannings-John, [1] (Senior Research Fellow), CanningsRL@cardiff.ac.uk

Lianna Angel, [2] (Research Associate), AngelL@cardiff.ac.uk

Susan Channon, [1] (Director Research Design and Conduct Service) ChannonS2@cardiff.ac.uk

Deborah Fitzsimmons, [3] (Personal Chair, Public Health, Policy and Social Sciences) d.fitzsimmons@swansea.ac.uk

Kerenza Hood, [1] (Director of the Centre for Trials Research) HoodK1@cardiff.ac.uk

Joyce Kenkre, [4] (Professor of Primary Care) joyce.kenkre@southwales.ac.uk

Gwenllian Moody, [1] (Research Associate) MoodyG@cardiff.ac.uk

Eleri Owen-Jones, [1] (Research Associate) Owen-JonesCE@cardiff.ac.uk

Rhys Pockett, [3] (Senior Lecturer) r.d.pockett@swansea.ac.uk

Julia Sanders, [5] (Professor of Clinical Nursing and Midwifery) SandersJ3@cardiff.ac.uk

Jeremy Segrott, [1,2] (Senior lecturer) SegrottJ@cardiff.ac.uk

Thomas Slater, [6] (Senior lecturer) slaterTB1@cardiff.ac.uk

Affiliations:

1. Centre for Trials Research, Cardiff University, Cardiff, UK
2. DECIPHer, Cardiff University, UK
3. Swansea Centre for Health Economics, Swansea University, UK
4. Faculty of Life Sciences and Education, University of South Wales, UK
5. School of Healthcare Sciences, Cardiff University, UK
6. School of Social Sciences, Cardiff University, UK

Corresponding author:

Michael Robling, Director of Population Health Trials, RoblingMR@cardiff.ac.uk, Centre for Trials Research, College of Biomedical & Life Sciences, Cardiff University, 7th Floor, Neuadd Meirionnydd, Heath Park, Cardiff. CF14 4YS

Keywords: Home-visiting, maltreatment, child development, routine data, cohort

For peer review only

Abstract (300 words)

Objectives Measure effectiveness of Family Nurse Partnership (FNP) home-visiting programme in reducing maltreatment and improving maternal health and child health, developmental and educational outcomes; explore effect moderators, mediators; describe costs.

Design Follow-up of BB:0-2 trial cohort (ISRCTN:23019866) up to age 7 years in England using record linkage.

Participants 1,618 mothers aged 19 years or younger and their firstborn child(ren) recruited to BB:0-2 trial at less than 25 weeks gestation and not mandatorily withdrawn from trial or opted out. Intervention families were offered up to a maximum of 64 home visits by specially trained nurses from pregnancy until firstborn child was 2 years old, plus usually provided health and social care support. Comparator was usual care alone.

Outcome measures Primary outcome: state-verified child-in-need status recorded at any time during follow-up. Secondary outcomes: referral to social services, child protection registration (plan), child-in-need categorisation, looked-after status, recorded injuries and ingestions any time during follow-up, early childcare and educational attendance, school readiness and attainment at Key Stage 1 (KS1), healthcare costs.

Results Match rates for 1,547 eligible children (1517 singletons, 15 sets of twins) were 98.3% (NHS Digital) and 97.4% (National Pupil Database). There was no difference between study arms in the proportion of children being registered as in need (adjusted OR=0.98, 95% CI=0.74,1.31), or for any other measure of maltreatment. Children in the FNP arm were more likely to achieve a good level of development at reception age (school readiness) (adjusted OR=1.24, 95% CI= 1.01,1.52). After adjusting for birth month, children in FNP arm were more likely to reach the expected standard in reading at KS1 (adjusted OR=1.26, 95% CI=1.02,1.57). We found no trial arm differences for resource use and costs.

Conclusions FNP did not improve maltreatment or maternal outcomes. There was evidence of small advantages in school readiness and attainment at KS1.

Strengths and Limitations

- We used administrative records and novel data linkage to provide longer-term follow-up for a well characterised trial cohort
- This has maximised cohort retention and minimised bias
- We used data from a range of sectors including health, social care and education to assess the broad impact of the specialist home visiting programme

For peer review only

Manuscript (Word count is 6631)

Introduction

Protecting children from maltreatment and promoting their healthy development are UK policy priorities. The Family Nurse Partnership (FNP) is a preventative home-visiting programme for young women expecting their first child. FNP was developed in the USA as the Nurse Family Partnership (NFP) and subsequently adapted for implementation in England in 2007.

Three US trials have demonstrated programme improvements in prenatal health behaviours and birth outcomes, sensitive childcare, maternal life-course and child functioning.[1–3] A subgroup analysis of poor unmarried teenage mothers in the first US trial (n=54) found verified maltreatment by age 2 years in 19% of 32 control children and 4% of the 22 children in the group in receipt of NFP during both pregnancy and infancy (mean % difference: 0.15, 95% confidence interval (CI) of −0.01 to 0.31).[1] Following up the same trial cohort, adjusted rates of verified state reports of child abuse and neglect perpetrated by mothers were lower in the group visited by nurses in pregnancy and infancy (0.29) when compared to the women in the control group (0.54) by 15 years (p<0.001).[4] Additionally, there was a 56% relative reduction in emergency department encounters for injuries and ingestions during the second year of life. In the Memphis trial of NFP, the number days in hospital following an injury or ingestion in the first two years of life (a possible indicator of maltreatment) was lower for children of mothers visited by nurses in pregnancy and infancy than children of mothers in the control group.[2] For children with state-verified maltreatment reported by age 4, children in the NFP group had fewer risks for harm than the control group between 25 and 50 months of life. A trial of VoorZorg, the Dutch adaptation of NFP, found a reduction in child-protecting agency reports by age three years for families in the home-visiting arm.[5,]

We evaluated short-term FNP programme outcomes to age 2 years in our Building Blocks trial (BB:0-2).[6] We found no difference for four primary outcomes: maternal tobacco use in late pregnancy, birth weight of the baby, proportion of women with a second pregnancy within 24 months postpartum and emergency attendances and hospital admissions for the child within 24 months postpartum. There were some differences in favour of FNP for secondary child development outcomes including maternal-reported cognitive function and language at 24 months.[6] As the previous US trials showed benefit for maltreatment outcomes increasingly after age four we sought to establish whether FNP moderates maltreatment outcomes over a medium-term period. Through access to administrative data, we aimed to determine impact across a range of child maltreatment outcomes and key indicators of

For peer review only - http://bmjopen.bmj.com/site/about/guidelines.xhtml

neglect (e.g. injuries and ingestions). We also sought to assess impacts on other programme relevant developmental and maternal outcomes as well as costs.

Objectives

Primary: to determine programme effectiveness in reducing maltreatment, when compared with usually provided health and social care alone. Secondary: to determine programme effectiveness in reducing maltreatment, medium-term programme outcomes such as subsequent pregnancies, school readiness and educational outcomes, the impact of moderators of programme effect and the cost and consequences of the programme.

Methods

Study design, setting and participants

This study followed a cohort of mothers and children who had participated in the BB:0-2 trial for a further five years using administrative data only.[7,8] Data were retrieved from public sector providers and linked to the trial dataset using opt-out consent, following REC approval (14/WA10062) and s251 support (CAG 10-08(b)/2014). Children and mothers were followed up until the child reached Key Stage 1 teacher-based assessment (KS1) by which time most children were seven years old.

Building Blocks: 2-6 (BB:2-6) study participants were women and their first child (or twins, if relevant) who were not mandatorily withdrawn from the BB:0-2 trial; did not electively withdraw and/or did not opt-out of this follow-on study.[6-8] BB:0-2 trial participants were eligible if women were nulliparous, aged 19 years or younger, were recruited at less than 25 weeks' gestation, living within the catchment area of a local FNP team, able to provide consent and speak English. Women expecting multiple births and those with a previous pregnancy ending in miscarriage, stillbirth, or termination were eligible. Women planning to have their child adopted or to move beyond the FNP catchment area for more than 3 months were not eligible. [6-9] Trial participants had been randomly assigned to FNP or usual care, with randomisation stratified by site and minimised by gestation (<16 weeks vs ≥16 weeks), smoking (yes vs no), and preferred language of data collection (English vs non- English) and weighted towards minimising the imbalance in trial groups with probability 0.8.

Intervention and comparator (BB:0-2 Trial)

The FNP programme provides up to a maximum of 64 home visits from specially trained family nurses during pregnancy until the child is two years old addressing personal and environmental health, life course development, maternal role, family and friends and access to health and social services. For the 697 women randomised and enrolled to FNP in BB:0-2 and then retained in the BB:2-6 cohort, the average number (SD) of valid visits reported by FNs as being received by FNP clients by programme phase was: Pregnancy 9.74 (3.42), Infancy 18.7 (5.97) and Toddlerhood 13.28 (5.46) (Table S1). In the BB:0-2 trial, mean (SD) visit duration varied by delivery phase (Pregnancy: 79.14

For peer review only - http://bmjopen.bmj.com/site/about/guidelines.xhtml

mins (13.78), Infancy: 73.17 (11.61), Toddlerhood: 74.75 (13.50) which exceeded the programme target of 60 minutes.[8] This compares to a duration of 75-90 mins (undifferentiated across the three programme delivery phases) reported for the Elmira and Memphis trials.[10] All trial participants received usually provided health and social care services. Participants in the usual care (UC) arm received these services alone.[6,8,11] The number (SD) of midwifery contacts were recorded in the BB:0-2 trial and were for FNP clients 10.68 (5.25) and for women in UC arm 10.69 (5.34).[11] The number (SD) of home visits from specialist public health nurses (SPHN) reported by trial participants by 18 months postpartum was for FNP clients 4.70 (7.81) and for women in the UC arm 5.01 (5.5). The average number (SD) of clinic contacts with SPHNs was 0.70 (2.29) for women in the FNP arm and 6.31 (7.07) for women in the control arm.

Procedure and data sources

The BB:0-2 trial consented 1618 eligible women between June 2009 and July 2010 and we concluded all BB:2-6 follow-up in May 2018. Maternal and child identifiers were sent to NHS Digital and the Department for Education (DfE) for matching with their databases. Maternal identifiers were also sent to the Department of Health and Social Care (DHSC) for matching with abortion statistics. Matching with NHS Digital and DHSC used a combination of NHS number, date of birth, postcode and sex. NHS Digital also provided a linked Hospital Episode Statistics (HES) - Office for National Statistics (ONS) extract for mortality data. Matching with the DfE used exact matching on first name and surname, date of birth and postcode (of both mother and child for social care data; all other datasets, just child). All matched data were sent to a third-party data safe haven (Secure Anonymised Information Linkage (SAIL) Databank) and linked by project identifiers to trial data and analysed via remote access.[12-14]

Outcomes

The primary outcome was Child in Need (CIN) status recorded at any time by a local department of children's social care services (CSCS) (and sourced from the NPD). This includes children assessed as unlikely to achieve or maintain a reasonable level of health or development, or whose health and development is likely to be significantly or further impaired, without the provision of services; or is a child who is disabled. Secondary outcomes were additional formally reported measures of maltreatment (referral to CSCS, child being on a Child Protection Plan, CIN categorisation, Looked after status (mother, child)); Associated measures of maltreatment (recorded injuries and ingestions, non-attendance rates for hospital appointments); Maternal outcomes (subsequent pregnancies); Child health, developmental and educational outcomes (special educational needs, early educational attendance and assessments (Early Years Foundation Stage Profile (EYFSP), KS1)); and health

For peer review only - http://bmjopen.bmj.com/site/about/guidelines.xhtml

resource use. Study assessment domains, outcomes and principal data providers and analysis are described in the appendix (Table S2).

Power calculation

Our sample size was fixed by the cohort available from BB:0-2. However, for CIN status, while UK data on rates are not specific to the age-range of interest, the rate per 10,000 general population aged 5-9 years is 4.6% for local authorities covering BB:0-2 trial sites.[15] The rate of CIN status would be expected to be greater in the specific study sample. Therefore, we assumed a rate of 8%, hypothesised that FNP would reduce the presence of CIN in the first six years and assumed a difference of 4% would be important. To detect a difference of 4% (4% vs 8%) would require 602 children in each arm with 80% power and a two-sided 5% alpha level. With 1562 children available for follow-up (i.e. excluding mandatory withdrawals) and follow up through administrative records with 10% loss in tracking and linkage we would have 1405 participants, sufficient data to assess the primary outcome.

Efforts to address sources of bias

All maltreatment and child developmental outcomes were independently state-verified (social worker) reports or teacher-assessments respectively, recorded in administrative service data and abstracted from national data providers. By using administrative data and established linking fields we were able to minimise bias due to loss to follow-up. An a priori statistical analysis plan described primary, secondary (including sub-group) and sensitivity analyses, and any additional exploratory analysis is clearly highlighted.

Statistical analysis

Analysis was conducted on participants whose identifiers could be sent, linked and data released by the data providers. This excluded participants whose individual data could be used but could not be matched due to incorrect linking fields, other exclusions from health or education: e.g. private or home schooling and National opt-outs. Imbalance between the BB:0-2 and eligible BB:2-6 participants was quantified using descriptive statistics in baseline demographics, clinical and questionnaire data. We conducted all analyses on an intention-to-treat (ITT) basis with complete case population (with follow-up data). Therefore, participants remained in the BB:0-2 trial groups they were randomised to regardless of the intervention received. We used multilevel modelling to allow for clustering of effect within the 18 sites and family nurse level and both fitted as random effects. Where clustering was minimal at the family nurse level, results from the two-level model are presented. Parameter estimates are reported alongside a 95% CI and p-value. We adjusted for variables used in randomisation (smoking status, gestational age, language). We defined loss to follow up in this study as a child death or adoption, both of which we were able to determine. We excluded children from

For peer review only - http://bmjopen.bmj.com/site/about/guidelines.xhtml

analyses of binary outcomes where follow-up was incomplete and no event was observed. With time-to-event analyses, children were censored at these events.

Primary comparative analysis examined whether the firstborn child(ren) ('BB:0-2') had ever been referred to CSCS and classed as a child in need at any point between birth and 6 years of age (appendix). For this binary outcome we used logistic multilevel modelling to investigate differences in the proportion defined as in need between the trial arms. We present the resulting estimate as an odds ratio. For binary and categorical outcomes, comparative analysis used logistic and multinomial modelling respectively with results presented as odds ratios (OR). For continuous outcomes we used linear regression and present mean differences. For count data we used Poisson regression modelling; where event distribution displayed signs of over dispersion a Negative Binomial regression model was used (or zero-inflated model if appropriate). Results are presented as incidence rate ratios (IRR). Time to event data used Cox regression modelling presented with hazard ratios (HR). To reduce problems with multiple comparisons, some outcomes were only assessed descriptively (for example, we prioritised and tested overall scores for a Good Level of Development in the Early Years Profile, rather than the individual areas of learning). Similarly, for outcomes that we expected to reflect a small number of events or higher levels of missingness we also took a descriptive approach. Nevertheless, we present all a priori comparative analyses in the main tables and all other descriptive and exploratory analyses in the supplementary materials.

KS1 data were received in two tranches from NPD, firstly children assessed in 2016/17 academic year (received in May 2018) and then younger children who were assessed in 2017/18 (received May 2019). Reading, maths and science were examined for the whole cohort over both academic years. However, following changes made within the 2017/18 writing assessment (such that judgements in 2018 are not directly comparable to those made using the previous interim frameworks in 2016/2017), writing was only statistically assessed using 2016/17 data and the data from 2017/18 were presented descriptively. Sensitivity analyses for primary and selected secondary outcomes (EYFSP, KS1, referral to CSCS) included adjusting for hypothesised confounders at baseline, dosage effects (i.e. number of visits) using complier average causal effects (CACE) modelling and sub-group analysis of potential effect moderators and mediators (maternal deprivation, adaptive functioning, NEET (not in education, employment or training) status at recruitment, maternal age at recruitment, child sex, maternal care status, duration of maternal care and domestic abuse self-reported at 24 months) as interaction terms in the main comparative models.

Results of all planned sensitivity analyses are presented in the appendix. An additional post hoc sensitivity analysis adjusted for the child's month of birth (categorised into quarter of birth) in the EYFSP and KS1 outcomes.

For peer review only - http://bmjopen.bmj.com/site/about/guidelines.xhtml

Economic Analysis

A cost consequences analysis of FNP over the full follow-up period (BB:0–2 and BB:2–6) took a secondary health care (UK NHS) perspective. The principal data source was Hospital Episode Statistics (HES) records (inpatient, outpatient, A&E), which were coded in Healthcare Resource Group (HRG) Grouper and matched to appropriate NHS Reference costs. Maternal and child resource use were costed separately and valued in £ sterling. Only direct healthcare costs were analysed. Indirect costs such as lost wages or childcare costs were not available within the routinely collected data. Where data were absent in HES records it was assumed no resource was incurred. No primary care data were available beyond 18 months postpartum. Costs were discounted back from year of event to baseline (2009/10) at an annual rate of 3.5% as described by Central Government's guidance on appraisal and evaluation. [16]

Data management

Small numbers were handled according to SAIL Databank rules where any cell counts under five were suppressed in reporting. Where abortion data are presented, we handled in accordance with the data sharing agreement with DHSC where counts less than 9 were suppressed. Data were analysed using Stata v16 and IBM SPSS v23.0.

Patient and public involvement

Activities involved four groups of young people (CASCADE Voices, Our Place, FNP graduates and ALPHA). These activities contributed to three aspects of the study: data-linkage methods (e.g. to optimise acceptability of the proposed dissent model, use of data linkage and communication of these methods),[17] study outcomes (by seeking views on the importance of the study outcomes) and dissemination of findings (including best way to present results, method of dissemination).

Results

The study population following the BB:0-2 trial tracing and matching process is shown in Figure 1. Five women were ineligible due to not meeting original trial entry criteria and 78 were mandatory withdrawals mainly due to fetal or infant death and adoption.[6] The 110 women who electively withdrew during the trial were given the opportunity to opt-out from further data usage, 16 of whom removed consent for any further contact. Thus 94 women who had electively withdrawn from BB:0-2 were included, and their details sent for tracing alongside the remaining 1,452 mothers (1,546 mother-child dyads in total) to update contact details to improve matching. One dyad was subsequently removed as deceased and the remaining 1,545 contacted by letter, email or text of whom eight dissented from having their records linked. 1,537 mothers and 1,547 children were sent to the DfE and

For peer review only - http://bmjopen.bmj.com/site/about/guidelines.xhtml

NHS Digital for matching. These formed the population for the BB:2-6 study. Match rates for children were 98.3% (NHS Digital) and 97.4% (DfE).

Numbers matched to and returned for analysis differed by data source and population (Table S3). Owing to having discontinued their education, few mothers, compared with children, were matched to any DfE data set (19% vs. 97%, respectively); these events were based mainly on the CIN data set. For selected maternal and child baseline variables and delivery of FNP assessed against programme fidelity goals, the BB:2-6 sample appears broadly representative of the original BB:0-2 cohort (Tables S4 and S5). Baseline characteristics of the BB:2-6 mothers and children were well balanced between trial arms (Table 1).

Table 1 Socio-demographic characteristics of mother and babies at baseline by trial arm

	FNP N=766	Usual care N=771
**Maternal age at recruitment (years)**	17·9 (17·0 to 18·8)	17·9 (16·9 to 18·8)
**Ethnicity**		
White background	675 (88·1%)	680 (88·2%)
Mixed background	44 (5·7%)	38 (4·9%)
Asian background	15 (2·0%)	10 (1·3%)
Black background	29 (3·8%)	40 (5·2%)
Chinese or Other background	<5	<5
**Relationship status with baby's father**		
Married	6 (0·8%)	10 (1·3%)
Separated	72 (9·4%)	78 (10·1%)
Closely involved/boyfriend	582 (76·0%)	586 (76·0%)
Just friends	106 (13·8%)	97 (12·6%)
**NEET Status***		
Yes	315/764 (41·1%)	319/769 (41·4%)
No	345/764 (45·0%)	333/769 (43·2%)
**IMD Overall Score†**	38·1 (24·6 to 52·6)‡	38·1 (25·5 to 51·6)§
**Generalised self-efficacy scale (score 10 to 40)¶**	30·0 (28·0 to 33·0)‖	30·0 (27·0 to 32·0)**
**Adaptive functioning:**		
**Difficulty in at least one basic skill**		
Yes	213/765 (27·8%)	184/770 (23·9%)
No	552/765 (72·2%)	586/770 (76·1%)
**Had 3 or less life skills (out of 5)**		

For peer review only

	FNP N=766	Usual care N=771
Yes	185/763 (24·2%)	218/769 (28·3%)
No	578/763 (75·8%)	551/769 (71·7%)
**At least one burden**		
Yes	214/759 (28·2%)	237/767 (30·9%)
No	545/759 (71·8%)	530/767 (69·1%)
**Cigarette smoking participant self-reported**		
Ever smoked		
Yes	615 (80·3%)	612 (79·4%)
No	151 (19·7%)	159 (20·6%)
**Child characteristics**	**FNP N=773**	**Usual Care N=774**
**Sex**		
Male	381/773 (49·3%)	406/773 (52·5%)
Female	392/773 (50·7%)	367/773 (47·5%)
**Birthweight (grams)** Mean (SD)	3223·81 (606·0)††	3215·52 (555·56)‡‡
**NNU admission (direct or subsequent)**		
Yes	76/716 (10·6%)	66/749 (8·8%)
No	640/716 (89·4%)	683/749 (91·2%)

Data are n (%), n/N (%), or median (25th to 75th centile) unless otherwise stated. <5 = numbers suppressed. FNP = Family Nurse Partnership Programme + Usual Care. IMD = Index of Multiple Deprivation. SD= standard deviation. NNU = Neonatal unit. *Definition of NEET status: Not in education employment or training (applicable only to those whose age at end of previous academic year at time of baseline interview was >16). †Higher IMD score indicated more deprivation. Mean IMD Score for England in 2010 was 21.67 [Wilkinson DL, Sniehotta FF, Michie S. Targeting those in need: Baseline data from the first English National Health Service (NHS) health trainer service: *Psychology, health & medicine* 2011; **16**(6): 736-48], ‡N=760. §N=765. ¶ Higher score indicates higher level of self-efficacy. ‖N=764. **N=769. ††N=724. ‡‡N=752.

For the definition of a child in need referrals resulting in no further action (i.e. not classed as in need) were excluded. Overall, 323 (21.5%) of children in the BB:2-6 cohort had at least one referral to CSCS which resulted as being classed as a child in need at any point between pregnancy (includes pre-birth CIN) and 6 years of age. No difference in rate of children in need was found between FNP (21.2%) and UC (21.7%) arms for the 1,506 children in the analysis (adjusted OR=0.98, 95% CI=0.74, 1.31) (Table 2). The parameter estimate was not affected when adjusting for the number of FNP visits received (efficacy per visit OR=1.00, 95% CI=0.99, 1.004). The number of children referred to CSC and assessed as not requiring additional support were FNP (n=45) and UC (n=46). There were no between arm differences for referral to CSCS, the age at which the first referral was made, the age at which the child was classified as in need and the proportion of children with a protection plan. A small number of children were ever Looked After within the 6-year follow-up

For peer review only - http://bmjopen.bmj.com/site/about/guidelines.xhtml

period, with no evidence of a difference between trial arms (Table 2). However, the UC arm experienced on average two more months in care compared to the FNP arm. The total number of child deaths across the full follow-up period was less than ten and not further reported by study arm, consistent with the non-disclosive data management policy. Data informing the disability outcome were not available for the years requested. In addition, no difference was found when examining age at first referral or CIN by study arm.

No between arm differences were found in children not taken to at least one outpatient appointment (did not attend rate), emergency attendances or hospital admissions for an injury or an ingestion (Table 2). Overall, 78% of mothers had a subsequent pregnancy (using inpatients, outpatients and abortions data) with no differences by arm (Table 3). Similarly, just over two thirds of the cohort had at least one registerable birth (using only admission data as a source) during the follow-up period with a comparable inter-birth interval. Descriptives for non-tested maltreatment outcomes are shown in Table S6. Descriptives for the number of emergency room attendances and hospital admissions for injuries or ingestions are shown for each trial arm in Tables S7-S8. Exploratory analysis found no differences in length of hospital stay between trial arms following admission for an injury or ingestion for children aged under 1 year old or for children aged between 1 year old and under 2 years old (Table S8). Exploratory analyses of hospital admissions for injuries and ingestions by trial arm shown by age categories (Under 1 year, 1 to 2 years, 25 months to under 6 years) are shown in Table S9.

For peer review only

Table 2 Primary and secondary outcomes: CPS outcomes, healthcare encounters for injuries / ingestions and out-patient non-attendance

Outcome	FNP	Usual care (UC)	Adjusted* parameter estimate (95% CI)	p-value	Absolute risk difference (FNP-UC) (95% CI)
**Child protection outcomes**	**N=760**	**N=746**			
Child in need (CIN) status at any time during the follow-up period					
No	599 (78·8%)	584 (78·3%)	Reference		
Yes	161 (21·2%)	162 (21·7%)	0·98† (0·74 to 1·31)	0·902	-0·5% (-0·5 to 0·4%)
Age at first CIN referral (days)					
Median (25th to 75th centile)	1602·5 (1153.0 to 1978·75)	1649·5 (1130·75 to 1980·25)	0·98‡ (0·79 to 1·23)	0·875	
Missing	*1*	*0*			
Unique§ number of CIN referrals per child	1 (1 to 2)	1 (1 to 2)	0·99‖ (0·82 to 1·19)	0·890	
CIN duration (days)	210 (70·5 to 571·25)	216 (72·75 to 503·25)	1·20‡ (0·90 to 1·59)	0·216	
CIN categorisation (primary need) of first case	***N=161***	***N=162***			
Abuse or neglect	92 (57·1%)	102 (63·0%)	Reference		
Family dysfunction	29 (18·0%)	34 (21·0%)	0·95¶ (0·54 to 1·69)	0·868	
Family in acute stress	13 (8·1%)	10 (6·2%)	1·47¶ (0·61 to 3·54)	0·389	

For peer review only - http://bmjopen.bmj.com/site/about/guidelines.xhtml

Outcome	FNP	Usual care (UC)	Adjusted* parameter estimate (95% CI)	p-value	Absolute risk difference (FNP-UC) (95% CI)
Low income, absent parenting, socially unacceptable behaviour, not stated	13 (8·1%)	6 (3·7%)	2·38‖ (0·86 to 6·57)	0·093	
Child/parent disability	7 (4·3%)	5 (3·1%)	1·47¶ (0·45 to 4·84)	0·525	
Cases other than CIN	7 (4·3%)	5 (3·1%)	1·64¶ (0·50 to 5·37)	0·417	
Referred to Children's social care services (CSCS)					
No	554 (72·9%)	538 (72·1%)	Reference		
Yes	206 (27·1%)	208 (27·9%)	0·97† (0·74 to 1·28)	0·829	-0·8% (-5·3 to 3·7%)
Age at first referral (days)<br>Median (25th to 75th centiles)	1541·5 (1272·75 to 1975·75)	1631·0 (1190·5 to 1984·75)	0·96‡ (0·79 to 1·17)	0·694	
Child Protection Plan					
No	708 (93·2%)	697 (93·4%)	Reference		
Yes	52 (6·8%)	49 (6·6%)	1·04† (0·69 to 1·57)	0·846	0·2% (-0·2 to 0·3%)
Category of child protection plan	***n=52***	***n=49***			
Neglect	17 (32·7%)	22 (44·9%)	Reference		
Emotional	22 (42·3%)	12 (24·5%)	2·77‖ (1·02 to 7·56)	0·046	
Physical	6 (11·5%)	6 (12·2%)	1·25‖ (0·32 to 4·88)	0·743	
Sexual and multiple**	7 (13·5%)	9 (18·4%)	1·13‖ (0·33 to 3·86)	0·848	

For peer review only - http://bmjopen.bmj.com/site/about/guidelines.xhtml

Outcome	FNP	Usual care (UC)	Adjusted* parameter estimate (95% CI)	p-value	Absolute risk difference (FNP-UC) (95% CI)
Looked after status††					
No	735 (96·7%)	719 (96·4%)	Reference		
Yes	25 (3·3%)	27 (3·6%)	0·90† (0·52 to 1·57)	0·712	-0·3% (-0·2 to 0·2%)
Child looked after period of care (months)	10·0 (4·5 to 37·5)	12·0 (6·0 to 33·0)	0·75‖ (0·65 to 0·86)	<0·001	
**Injuries and Ingestions**	**N=760**	**N=759**			
Emergency attendance					
None	317 (41·7%)	344 (45·3%)	Reference		
At least one	443 (58·3%)	415 (54·7%)	1·17† (0·95 to 1·45)	0·149	3·6% (-1·4 to 8·6%)
Number of attendances per child	2 (1 to 2)	2 (1 to 3)	1·09‖ (0·93 to 1·28)	0·281	
Admission to hospital					
None	671 (88·3%)	660 (87·0%)	Reference		
At least one	89 (11·7%)	99 (13·0%)	0·87† (0·63 to 1·20)	0·407	-1·3% (-4·7 to 2·0%)
Unique admissions	109	119			
Ratio of admissions to children	1.22	1.20			
Number of admissions per child, median (25th to 75th centile)	1 (1 to 1)	1 (1 to 1)	0·93‖ (0·67 to 1·29)	0·663	
Length of stay (days) ‡‡	N=106	119			
Median (25th to 75th centile) days	0.5 (0.5 to 1.0)	0.5 (0.5 to 1.0)			

For peer review only - http://bmjopen.bmj.com/site/about/guidelines.xhtml

Outcome	FNP	Usual care (UC)	Adjusted* parameter estimate (95% CI)	p-value	Absolute risk difference (FNP-UC) (95% CI)
Hospital attendance and/or admission					
None	306 (40·3%)	324 (52·7%)	Reference		
At least one	454 (59·7%)	435 (57·3%)	1·11† (0·89 to 1·37)	0·351	2·4% (-2·5 to 7·4%)
DNA outpatient appointment	***N=580***	***N=577***			
Attended all appointments	290 (50·0%)	289 (50·1%)	Reference		
DNA at least once	290 (50·0%)	288 (49·9%)	1·00† (0·79 to 1·26)	0·997	0·09% (-5·6 to 5·8%)

Data are n (%), n/N (%), or median (25th to 75th centile) unless otherwise stated. FNP = Family Nurse Partnership Programme + Usual Care. * FNP compared to usual care. Analysis adjusted for stratification (site), minimisation variables (gestational age, smoking status at recruitment, and first or preferred language); † Odds ratio from logistic model; ‡ Hazard ratio from Cox model; § A unique referral is counted as a distinct referral date per child; ‖ Incidence rate ratio from Poisson model; ¶ Relative risk ratio from multinomial model; ‡‡Using diagnosis in any position; **Multiple indicates when more than one category of abuse is relevant to the child's current plan; †† We reflect here the terminology used at the time of the trial but note the more contemporary 'care experienced' as a more familiar and accepted term ‡‡ 0.5 days indicates an admission and discharge on the same day (could be up to 1 day in hospital). Data available from NHS Digital which show an admission and discharge one day apart may not equate to one full day in hospital (ie the child may have been in hospital for only a few hours if the admission spanned midnight). Source: National Pupil Database, The Department for Education

For peer review only - http://bmjopen.bmj.com/site/about/guidelines.xhtml

Table 3 Secondary outcomes: Subsequent pregnancies and registerable birth

	FNP	Usual care	Adjusted* parameter estimate (95% CI)	p-value	Absolute risk difference (FNP-Usual care) (95% CI)
Subsequent pregnancy	*N=753*	*N=753*			
No	163 (21·7%)	163 (21·7%)	Reference		
Yes	590 (78·4%)	590 (78·4%)	1·00† (0·79 to 1·28)	0·984	0·0% (-4·2 to 4·2%)
Subsequent registerable birth	*N=752*	*N=749*			
No	276 (36·7%)	266 (35·5%)	Reference		
Yes	476 (63·3%)	483 (64·5%)	0·95† (0·77 to 1·18)	0·662	-1·2% (-6·0 to 3·7%)
One birth	326 (68·5)	331 (68·5)	0·95‡ (0·76 to 1·19)	0·655	
Two	124 (26·1)	121 (25·1)	0·99‡ (0·73 to 1·34)	0·955	
Three births or more	26 (5·5)	31 (6·4)	0·80‡ (0·46 to 1·39)	0·435	
Inter-birth interval between first and second child (days) Median (25th to 75th centiles)	1027 (590 to 1506·75)	1065 (665 to 1538)	0·99§ (0·88 to 1·13)	0·938	

Data are n (%), n/N (%), or median (25th to 75th centile) unless otherwise stated. FNP = Family Nurse Partnership Programme + Usual Care. * FNP compared to usual care. Analysis adjusted for stratification (site), minimisation variables (gestational age, smoking status at recruitment, and first or preferred language); † Odds ratio from logistic model; ‡ Relative risk ratio from multinomial model; § Hazard ratio from Cox model.

For peer review only - http://bmjopen.bmj.com/site/about/guidelines.xhtml

Table 4 Secondary outcomes: Child health, developmental and educational

Outcome	FNP	Usual care	Adjusted* odds ratio (95% CI)	p-value	Absolute risk difference (FNP-Usual care) (95% CI)
Special educational needs (SEN)† provision	**N=759**	**N=747**			
No	540 (71·1%)	502 (67·2%)	Reference		
Yes	219 (28·9%)	245 (32·8%)	0·83 (0·67 to 1·03)	0·097	-3·9% (-8·6 to 0·7%)
**Early educational attendance**	**N=759**	**N=747**			
Attending an Ofsted registered private, voluntary and independent establishment up to the age of 4 years	334 (43·9%)	308 (41·2%)	1·13 (0·91 to 1·40)	0·281	2·7% (-2·3 to 7·7%)
**School attendance**	**N=754**	**N=740**			
Overall absence					
No absences	14 (1·9%)	14 (1·9%)	Ref		
At least one absence	740 (98·1%)	726 (98·1%)	1·00 (0·47 to 2·12)	0·998	0·0% (-1·4 to 1·5%)
Overall authorised absence					
No absences	26 (3·4%)	26 (3·5%)	Ref		
At least one absence	728 (96·6%)	714 (96·5%)	1·01 (0·58 to 1·75)	0·984	0·1% (-1·8 to 2.8%)
Overall unauthorised absence					
No absences	256 (34·0%)	245 (33·1%)	Ref		
At least one absence	498 (66·0%)	495 (66·9%)	0·95 (0·76 to 1·18)	0·620	-0·8% (-5·6 to 3·9%)

For peer review only - http://bmjopen.bmj.com/site/about/guidelines.xhtml

**Outcome**	**FNP**	**Usual care**	**Adjusted* odds ratio (95% CI)**	**p-value**	**Absolute risk difference (FNP-Usual care) (95% CI)**
**Early Years assessment**	**N=743**	**N=728**			
**Achieving good level of development (GLD) ‡**					
Achieving GLD in all five areas of learning‖	431 (58·0%)	380 (52·2%)	1·26 (1·03 to 1·55)	0·026	5·8% (0·7 to 10·9%)
Achieving GLD in all 17 early learning goals	412 (55·5%)	365 (50·1%)	1·24 (1·01 to 1·52)	0·043	5·3% (0·2 to 10·4%)
**Total point score§** Mean (SD)	32·22 (7·25)	31·59 (7·62)	0·65 ‖ (-0·11 to 1·41)	0·094	
**Key Stage 1 assessments**	**N=740**	**N=732**			
**Reading**					
Lower than expected	257 (34·7%)	289 (39·5%)	Reference		
Reaching at least the expected standard¶	483 (65·3%)	443 (60·5%)	1·23 (0·99 to 1·53)	0·051	4·8 (-0·2 to 9·7%)
Expected standard	*371 (50·1%)*	*337 (46·0%)*	*1·24 (0·99 to 1·56)*	*0·056*	*4·1 (-1·0 to 9·2%)*
Higher standard	*112 (15·1%)*	*106 (14·5%)*	*1·20 (0·88 to 1·65)*	*0·250*	*0·7 (-3·0 to 4·3%)*
**Maths**					
Lower than expected	281 (38·0%)	283 (38·7%)	Reference		
Reaching at least the expected standard¶	459 (62·0%)	449 (61·3%)	1·04 (0·84 to 1·28)	0·731	0·7 (-4·3 to 5·6%)
Expected standard	*392 (53·0%)*	*376 (51·4%)*	*1·06 (0·85 to 1·32)*	*0·611*	*1·6 (-3·5 to 6·7%)*
Higher standard	*67 (9·1%)*	*73 (10·0%)*	*0·93 (0·64 to 1·35)*	*0·711*	*-0·9 (-3·9 to 2·1%)*

For peer review only - http://bmjopen.bmj.com/site/about/guidelines.xhtml

Outcome	FNP	Usual care	Adjusted* odds ratio (95% CI)	p-value	Absolute risk difference (FNP-Usual care) (95% CI)
**Science**					
Lower than expected	203 (27·4%)	219 (29·9%)	Reference		
Reaching at least the expected standard**	537 (72·6%)	513 (70·1%)	1·14 (0·91 to 1·43)	0·254	2·5 (-2·1 to 7·1%)
**Writing academic year 2016/17**	**N=498**	**N=487**			
Lower than expected	257 (51·6%)	278 (57·1%)	Reference		
Reaching at least the expected standard¶	241 (48·4%)	209 (42·9%)	1·24 (0·97 to 1·60)	0·090	5·5 (-0·7 to 11·6%)
Expected standard	*218 (43·8%)*	*182 (37·4%)*	*1·29 (1·00 to 1·68)*	*0·054*	*6·4 (0·3 to 12·5%)*
Higher standard	*23 (4·6%)*	*27 (5·5%)*	*0·92 (0·51 to 1·64)*	*0·769*	*-0·9 (-3·8 to 1·9%)*

Data are n (%), n/N (%), or median (25th to 75th centile) unless otherwise stated. FNP = Family Nurse Partnership Programme + Usual Care. SD=standard deviation. *FNP compared to usual care. Analysis adjusted for stratification (site), minimisation variables (gestational age, smoking status at recruitment, and first or preferred language); † A child with SEN provision is recorded using the following codes (A = School Action, P = School Action Plus, E = Education, Health and Care Plan, S=Statement, K= SEN support) and the N code was used to indicate No SEN support in any of the following datasets between 2013 and 2017. PLASC (Autumn, Spring and Summer terms), AP, PRU Census, and EYC. If for any of the years no response was recorded then the assumption was that the child was not included in the denominator as either not present on Census day or not in school; ‡ Children achieving a good level of development are those achieving at least the expected level within the prime and specific areas of learning; §Total point score ranges from 17 to 51 with a higher score indicating a better level of development; ‖ Mean difference from linear model; ¶ Working at the expected standard and at a greater depth within the expected standard; **Working at a greater depth within the expected standard is not applicable in science. Source: National Pupil Database, The Department for Education

For peer review only - http://bmjopen.bmj.com/site/about/guidelines.xhtml

Evidence of positive programme impacts were found for some educational outcomes assessed at the end of the reception year and at KS1 by which time the child would be seven years old (Table 4). Children in the FNP arm were more likely to reach a good level of development across all five areas of learning by the end of the reception year (58.0%) than children in the UC arm (52.2%) and also to achieve a good level of development in all 17 early learning goals (FNP: 55.5%, UC: 50.1%). The small advantage for children of nurse-visited mothers was consistent across all five areas of learning although not tested statistically (Table S10).

For KS1 reading, writing, maths and science assessments there was no evidence of a difference between arms in the proportion of children reaching at least the expected standard nor specifically in those working at the expected level or at a greater depth of knowledge (Table 4). What is observed from the writing assessment is the increase in the rates of children reaching at least the expected standard between the two academic years (Overall 45.7% in 2016/17 to 66.5% in 2017/18), reflective of all assessments across the two academic years in each arm (Table S11). This is mainly explained by the distribution of births for the children for each academic year (Figure S1). Reflecting the recruitment period of the trial, children with KS1 results available in the academic year 2016/17 were born between October 2009 and August 2010 but skewed towards the summer born children (42% born between June and August 2010) whereas children appearing in the 2017/18 academic year were born between September 2010 and February 2011. There is a nationally observed relationship between rates of children reaching the expected standard and month of birth e.g. a 17% difference in reading for children born in August and September.[18] Therefore, our 2017/18 cohort of children do not represent the whole academic year, but are biased towards those that are more likely to achieve. This indicates that the rates of children reaching the expected standard varies by their month of birth and is important as a moderator of programme effect. Importantly, the original trial allocation ensured study arms remained balanced with regards month of birth and the intervention had no impact on total weeks gestation.[6] As a sensitivity analysis, the main analyses were adjusted for the child's month of birth (categorised into quarter of birth) and evidence of a between arm difference was found in reading assessments (Table S12). In addition, the Early Years assessments were adjusted for child's month of birth, which strengthened the association found in Table 4.

Details of all planned sub-group analyses and further exploratory analyses are shown in the appendix (Tables S13 to S18). The latter included exploration of potential surveillance bias for all children assessed as a Child in Need by age 4 years old. This explored whether children in the FNP arm may have been referred and assessed as in need at lower levels of concern than children in the Usual Care arm based upon maternal baseline characteristics (Table S18). Mothers in the Usual care arm were more likely to have not been in education, employment or training than mothers in the FNP arm but there were no other differences at baseline. The two groups of children assessed as in need were also no different on subsequent measures of school readiness.

For peer review only - http://bmjopen.bmj.com/site/about/guidelines.xhtml

Cost

There were negligible resource use and cost differences for both mothers (p=0.393) and children (p=0.865) between study arms (Table S19). The adjusted incremental costs of programme delivery per women in BB:0-2 (£1,811) remain the key observed cost difference between study arms.

Discussion

Principal findings

In this data linkage study we found that adding FNP to usually provided health and social care provided no additional benefit for maltreatment outcomes including the numbers of children referred to CSCS, registered as in need of additional support, given a child protection plan, entering care, attending an emergency department or being admitted for an injury or ingestion, or duration of stay if admitted. While there was no difference between study arms in the small number of children in care, the UC arm experienced on average two more months in care compared to the FNP arm. The total number of child deaths found was less than ten and therefore disclosive and cannot be reported. High rates of children ever registered as in need in both study arms indicates substantial need amongst families. We found the programme led to more children reaching a good level of development at the end of school reception year and, when taking month of birth into account, improvements in reading at KS1. Writing scores improved as a result of FNP for boys, children of younger mothers and of mothers who were not in education, employment or training when first recruited to the study. We found no other difference between families who received FNP and those who did not. With no differences in secondary health resource use and costs for FNP, the programme can be considered cost-neutral compared to usual care in the medium-term (2-6 years).

Strengths and weaknesses of the study

The prioritisation of outcomes in our original trial attracted some debate.[19-21] However, the trial and the current follow-on study are both comprehensively reported, and preceded by a priori analysis plans providing transparency and guarding against undue post hoc interpretation.[7-9] Although a primary outcome of child being in need of additional support was incorporated into the study design, we adopted a multimethod multisource approach to look at the range of maltreatment outcomes in drawing our conclusions. The consistent pattern of results within the two principal outcome domains provides greater confidence in what we can conclude. While the study benefited from random allocation in the BB:0-2 trial, when assessing educational outcomes we additionally adjusted for month of birth. This was because at a national level, month of birth is strongly associated with educational outcomes and the distribution of participants recruited to the trial varied by month.

For peer review only - http://bmjopen.bmj.com/site/about/guidelines.xhtml

The available maltreatment data provides an essential overview of family experience as recorded in official records but may lack some depth which could have allowed a more nuanced understanding for children referred. The educational data are teacher-reported statutory tests but similarly provide less detailed insight into child functioning which research instruments may have provided. The lack of detailed social care and any primary care resource use data limited the perspective of the economic analysis. However, using administrative data has enabled comprehensive cohort follow-up, with few cases lost for analysis preserving the benefits of the original trial randomisation. The data included are also objectively recorded rather than maternally self-reported, allow for easy comparison nationally and over time. Our approach has gathered more data at less cost than comparable prospective data collection and minimised family burden.

In our previous trial report of outcomes at 24 months postpartum, there were higher rates of safeguarding events recorded in GP notes for children in the FNP arm (n=64/469, 13.6%), compared to the usual care arm (n=38/476, 8.0%).[6] Additionally, more mothers in the FNP arm reported their child had ever been referred to social services (n=119/580, 20.5%) compared to mothers in the usual care arm (n=91/541, 16.8%). These finding can be indicative of surveillance bias or more accurate reporting by mothers in the FNP arm respectively. In the current study a tendency for children to be referred at lower thresholds of concern or closer involvement by attending FNP nurses may have been evidenced through a greater proportion of children referred to social services being assessed as requiring no additional support. However, we did not observe this in our cohort.

With no absolute external criterion for maltreatment, a reliance solely upon formal child protection reports may be limiting. In the US Elmira trial, a review of 42 children (intervention arm n=13, control arm n=29) referred to CPS between birth and their fourth year of life found few differences in the nature of maltreatment recorded. However, measures of the quality of home environment, parenting, child intellectual functioning and use of healthcare services between children's 25th and 50th months of life favoured families in the intervention arm.[22] It has been suggested that both parental and professional behaviour may lead to programme enrolled CPS reported children being at lower risk of harm than their counterparts in receipt of usual care alone. Thresholds for referral to social services may differ between specialist home-visiting nurses with high contact time, more in-depth relationship with clients and different approaches to risk and safety management and other health, social and educational care professionals.[23] For children referred to CSCS and assessed as a Child In Need, we explored whether the trial arms differed on characteristics that may suggest a lower threshold of concern for those in the FNP arm. Our comparative analysis was limited principally to baseline maternal characteristics to reduce the risk of interpreting outcomes subsequent to the Child In Need assessment as evidence of contemporary child concerns. The analysis did not support a hypothesis of surveillance bias, with the only difference being a higher level of educational, employment and

For peer review only - http://bmjopen.bmj.com/site/about/guidelines.xhtml

training inactivity amongst mothers in the Usual Care arm. While this differs to the findings in the Elmira trial, it does not rule out the possibility that such mechanisms may have been operating.[22] CPS referrals for children in receipt of FNP at lower levels of apparent concern would make it harder to compare on such outcomes, particularly during the programme delivery period (ie up to the child's second birthday). However, subsequent progress of children through the child protection system and later occurring outcomes (for example, children being taken into care) may be less liable to such influences but in our sample such numbers are small.

Verified outcomes documented in administrative records contrast with maternal subjective self-reports for which there may be no potential external criterion (ie a child being referred to CPS is a verifiable event, whereas maternal well-being is not). Nevertheless, the presentation or referral of families and their assessment against legally defined statuses such as being a Child in Need requires professional judgement. It has been suggested that surveillance bias is not likely to be problematic for assessing maltreatment using verified service data (i.e. by inflating the numbers of children being reported when in receipt of home-visiting).[24,25] As others have found this could be higher when clients are actively engaged with services and as noted above, the potential for such bias remains.[26]

It has been proposed that other documented evidence such as hospital admission due to injury and ingestions and in particular duration of subsequent stay may provide a better indicator of maltreatment severity. In the follow-up of the US Memphis trial cohort at two years of the child's life, children in the intervention arm admitted following an injury or ingestion spent fewer days in hospital than their counterparts in the control arm. In the US Elmira trial, no difference between trial arms were found in the number of days hospitalised during the 25- to 50-month period of follow-up after adjustments were made for one outlier (in the intervention arm) assessed as being not relevant to parental caregiving.[27] We explored length of hospital stay following admission for an injury and ingestion both overall and for time periods representing periods of increasing of child mobility (ie under 1 year of age, 1 to under 2 years of age and 25 months to under 6 years of age). These were similar in each case but factors other than severity of clinical need may influence how long children remain in hospital. Wide differences exist between countries for hospital stay duration following injuries, ingestions and other consequences of external causation (when assessed across adult and paediatric populations).[28] One observational UK study of predictors of length of stay amongst paediatric inpatients (all causes) shows the contribution of health service factors in addition to clinical and social factors.[29] Interestingly, a large US study amongst paediatric inpatients found that non-accidental trauma compared to accidental trauma was a strong predictor of having a prolonged hospital stay even after adjusting for injury severity.[30] Therefore, length of hospital stay may be indicative of maltreatment, severity of clinical presentation, health service factors and possibly social factors. Further clarifying

For peer review only

the factors that independently drive length of hospital stay and the validity of this outcome as a measure of maltreatment will be valuable.

Potential sources of bias

As an open label study, it is possible that service professionals may have attempted to compensate for the lack of specialist support from FNP for participants in the usual care arm. However, it should be noted that enhanced care would have been expected as part of usual practice for teenage mothers, for example, through teenage pregnancy midwives. The number of midwifery contacts were recorded in the trial and found to be equivalent between FNP and UC arms.[11] The number of home visits from specialist public health nurses (SPHN) reported by trial participants by 18 months was also equivalent between study arms. As would have been expected, the number of clinic contacts with SPHNs was greater for women in the control arm than in the FNP arm. In contrast, women allocated to the intervention arm would have received an average of just over 40 home visits from a family nurse before graduating from the programme. Overall, this suggests a common core of usual care provided across both trial arms, a higher level of SPHN input to the usual care arm reflecting in part the responsibility of delivering the Healthy Child Programme (which was formally allocated to FNP nurses rather than SPHNs for their clients). The capacity of SPHNs to provide additional support will also be limited by their caseload. The maximum caseload currently recommended by the Institute of Health Visiting is one practitioner per 250 children. However, in 2015, 28% of surveyed practitioners in England reported caseloads in excess of 400 children and 12% reported between 500 and 1000+ families.[31]

The potential for the open label nature of the study to introduce bias through the actions of SPHNs is an important question and can be viewed through the lens of contemporary behavioural change frameworks such as COM-B.[32] This approach looks at three essential conditions required for a change to occur (eg in this instance a SPHN acting to provide enhanced support to a mother) - Capability, Opportunity and Motivation. Firstly, do SPHNs have the skills, training, model of delivery and supportive management structure (ie Capability) to be provide an enhanced service? Many SPHNs would have had similar professional backgrounds and skills to their FNP colleagues, and some expressed similar practice aspirations to those they perceived amongst family nurses. In contrast, most FNP nurses would have been selectively recruited from existing SPHNs and received additional programme training, supervision and a distinct programme delivery model. While the capabilities of SPHNs and FNP nurses certainly overlapped, they were unlikely to be equivalent at the time of the trial. Second, as noted above, the opportunity for SPHNs to deliver additional support would have been limited by their caseload. This was evidenced by the testimony of SPHNs in the trial's process evaluation and reported caseloads of up to 600 women at two trial sites.[8] The reduction to an individual SPHN's own caseload associated with an FNP nurse enrolling up to her caseload maximum

For peer review only - http://bmjopen.bmj.com/site/about/guidelines.xhtml

of 25 clients would appear to offer limited scope to substantially increase the opportunity to then enhance provision. Finally, would SPHNs be motivated to enhance service provision either in general (eg to all clients who may be seen as likely to benefit from additional support) or specifically to participants allocated to the trial's control arm? In our trial's process evaluation, several SPHNs reported a lack of awareness about whether their clients were in the trial and in some cases a misperception that they shouldn't know either. SPHNs with the potential to provide additional care and to do so specifically for women they knew to be enrolled in the trial and allocated to the control arm (and not say to other women with similar needs unwilling to participate in the trial or who were not identified to do so) would require considerable motivation. To do so would also represent a wilful attempt to undermine the requirements of a trial supported by their own departments. For this to occur consistently for a large number of SPHNs both within and across the 18 trial sites at a level that may start to impact upon outcomes seems less likely. Given the 'fire-fighting' described by SPHNs in delivering care as usual to their large caseloads, the additional motivation to target additional support to women known to be in the trial seems more a theoretical rather than a practical risk.

Comparison with other studies

The strongest evidence for the programme preventing maltreatment remains from the original US Elmira trial. While the programme reduced the number of substantiated reports of abuse and neglect in the first 15 years of the child's life, the advantage emerged after age four years.[4] We found differences in duration spent in care for looked after children, but the numbers involved are small and across the range of maltreatment outcomes the pattern is of no overall group difference. The programme was no more effective in tested sub-groups. It is worth recognising differences in the population of clients in receipt of the programme across the different study populations, their social circumstances (which also may have changed over time) and how that may determine potential to benefit from FNP.[11] We have not directly explored differences in social care systems between England and the US but these may vary the underlying risk of maltreatment and its likelihood of detection.[33]

School entry reading and maths is of important predictive value for later school achievement.[34] Early childhood educational investment programmes typically show a modest impact upon cognitive and achievement scores (e.g. weighted average of 0.21 standard deviations) with some decline in observed impact being attributed to general improvements in both school and home environments.[35,36] The question of comparator is therefore of particular relevance. In this study, a range of state and third sector funded services (including midwifery and health visiting) would have been available as usual care, possibly limiting the incremental benefit possible from the family nurse partnership programme.[11] Nevertheless, as noted above women allocated to FNP received substantial input from

For peer review only - http://bmjopen.bmj.com/site/about/guidelines.xhtml

family nurses, albeit fewer than possible according to the maximum fidelity targets at the time of the trial.

Home visiting interventions, which aim to impact upon a range of outcomes, have shown similarly modest effects on child cognitive outcomes (e.g. mean Effect Size 0.25, 96% CI 0.11, 0.38) with considerable variability due to programme composition (e.g. greater effects with programmes addressing parental responsiveness, sensitivity to care and nurturing and rehearsal and role-playing).[37] Our BB:0-2 study found benefits at age two years for children in the FNP arm for both language and developmental outcomes but these outcomes were maternally reported and potentially subject to reporting bias in the open trial.[9] This new medium-term evidence for programme impact upon developmental outcomes adds to that picture, although the size of effect remains small. Similar medium-term programme impact (to age six years old) on developmental outcomes (eg, intellectual functioning and receptive language scores) was reported from the Memphis trial.[38] A reanalysis of those data found cognitive benefits at age 6 years attributed to both programme induced improvements in maternal traits and family life investments at age 2.[39]

Unanswered questions and future research

Given small but positive findings for developmental outcomes, tracking the cohort through to subsequent school years is warranted. As social care data are equally accessible via the NPD there is value in observing the trajectory for maltreatment outcomes given that the principal maltreatment findings from the US Elmira trial are located in the 15-year follow-up. Linkage to data in other sectors such as welfare would provide a greater understanding of broader impact for families. Such work should be driven by the programme's logic model, existing evidence for long-term programme impact, informed by programme evolution in England and supplemented by work to understand what may explain differing programme benefits across national settings.[4,40]

Conclusions

There are no evident programme benefits for maltreatment outcomes by age six but the FNP generates higher rates of child attainment at the end of the reception year and at KS1. The adapted programme remains locally commissioned and delivered in England.[40,41] Local needs and priorities may determine the weight attached to these different sets of outcomes.

Acknowledgements

The Centre for Trials Research receives funding from Health and Care Research Wales and Cancer Research UK. The work was undertaken with the support of The Centre for the Development and Evaluation of Complex Interventions for Public Health Improvement (DECIPHer), a UKCRC Public Health Research Centre of Excellence. Joint funding (MR/KO232331/1) from the British Heart

For peer review only - http://bmjopen.bmj.com/site/about/guidelines.xhtml

Foundation, Cancer Research UK, Economic and Social Research Council, Medical Research Council, the Welsh Government and the Wellcome Trust, under the auspices of the UK Clinical Research Collaboration, is gratefully acknowledged.

Data re-use statement: Copyright © (2019), the Health and Social Care Information Centre. Re-used with the permission of the Health and Social Care Information Centre. All rights reserved.

We would like to thank all members of CASCADE voices, ALPHA and Our Place for contributing to the study including those who co-ordinated access to these individuals. Thanks are due to the FNP teams in Bristol, Bath and Wiltshire who helped us to make contact with FNP clients and graduates during our PPI work. We would also like to acknowledge the contribution of members of the study steering committee - Ann Louise Kinmonth (Chair), David Low, Gordon Taylor, Nina Biehal, Rachel Tonkin, Stavros Petrou, and Silvia van den Heijkant.
This work uses data provided by patients and collected by the NHS as part of their care and support as well as data from schools and local authorities. Using patient and other public data is vital to improve health and care for everyone. There is huge potential to make better use of information from people's patient records, to understand more about disease, develop new treatments, monitor safety, and plan NHS services. Patient and other public data should be kept safe and secure, to protect everyone's privacy, and it's important that there are safeguards to make sure that it is stored and used responsibly. Everyone should be able to find out about how patient data is used. #datasaveslives You can find out more about the background to this citation here: https://understandingpatientdata.org.uk/data-citation. [To note. This has been adapted to include other data sources not made available from the NHS such as education and social services data].

Footnotes

Author contributions: MR was the chief investigator and acts as guarantor for the study. MR, FLW, RCJ, LA, SC, DF, KH, JK, GM, EOJ, RP, JSa, JSe, TS contributed to study design. FLW was the study manager. RCJ was the lead statistician. DF supervised the health economics. MR, FLW, RCJ, LA, SC, DF, KH, JK, GM, EOJ, RP, JSa, JSe, TS were responsible for study management. GM and LA were the data managers. JK and JS led for public involvement. GM and LA cleaned the data. RCJ designed and conducted the analysis. RP conducted the economic analysis. MR, JSa, RCJ, SC, KH, JK, EOJ, JSe were co-applicants on the original funding application. MR, FLW, RCJ, LA, SC, DF, KH, JK, GM, EOJ, RP, JSa, JSe, TS interpreted the results of the analysis. RC-J and RP were responsible for producing the tables. MR, FLW, RC-J and RP drafted the study report. MR, FLW, RCJ, LA, SC, DF, KH, JK, GM, EOJ, RP, JSa, JSe, TS reviewed manuscript drafts, revised for important intellectual content and approved the final version.

For peer review only - http://bmjopen.bmj.com/site/about/guidelines.xhtml

Funding: This project was funded by the National Institute for Health Research Public Health Research (NIHR PHR) Programme (reference:11/3002/11). The views and opinions expressed therein are those of the authors and do not necessarily reflect those of the NIHR PHR Programme or the Department of Health. The funders had no role in considering the study design or in the collection, analysis, interpretation of data, writing of the report, or decision to submit the article for publication.

Competing interests: All authors have completed the ICMJE uniform disclosure form at www.icmje.org/coi_disclosure.pdf and declare: all authors declare financial support from NIHR PHR for the submitted work; no financial relationships with any organisations that might have an interest in the submitted work in the previous three years; no other relationships or activities that could appear to have influenced the submitted work.

Ethical approval: Ethical approval for this study was granted by the Health Research Authority research ethics committee (Wales REC 3; REC reference 14/WA10062) and section 251 support was provided by the Confidentiality Advisory Group (CAG reference CAG 10-08(b)/2014).

Data sharing: Anyone wishing to access the linked datasets for research purposes should apply via the CAG to the Health Research Authority to access patient identifiable data without consent and then to the NHS Digital, National Pupil Database and the abortion statistics – Department for Health and Social Care. In the first instance, enquiries about access to the data should be addressed to Professor Mike Robling, Centre for Trials Research, Cardiff University.

The lead author (MR) affirms that the manuscript is an honest, accurate, and transparent account of the study being reported; that no important aspects of the study have been omitted; and that any discrepancies from the study as planned (and, if relevant, registered) have been explained.

Dissemination to participants and related public communities: The findings from the BB:2-6 study, including those from this paper will be disseminated directly to participants where valid contact details remain available, via social media and conference presentations. This will include the use of lay-informed infographic materials. Summary results will also be made available on the study webpage [https://evidencinghomevisiting.co.uk/].

References

1. Olds DL, Henderson CR, Chamberlin R, Tatelbaum R. Preventing Child Abuse and Neglect: A Randomized Trial of Nurse Home Visitation. Pediatrics. 1986;78(1). Available from: http://pediatrics.aappublications.org/content/pediatrics/78/1/65.full.pdf

For peer review only

2. Kitzman H, Olds DL, Henderson CR, Hanks C, Cole R, Tatelbaum R, et al. Effect of Prenatal and Infancy Home Visitation by Nurses on Pregnancy Outcomes, Childhood Injuries, and Repeated Childbearing. JAMA. 1997;278(8):644. Available from: http://jama.jamanetwork.com/article.aspx?doi=10.1001/jama.1997.03550080054039

3. Olds DL, Robinson J, O'brien R, Luckey DW, Pettitt LM, Henderson CR, et al. Home Visiting by Paraprofessionals and by Nurses: A Randomized, Controlled Trial. Pediatrics. 2002;110(3). DOI: 10.1542/peds.110.3.486 Available from: http://pediatrics.aappublications.org/content/110/3/486.short

4. Olds DL, Eckenrode J, Henderson CR, Kitzman H, Powers J, Cole R, et al. Long-term Effects of Home Visitation on Maternal Life Course and Child Abuse and Neglect Fifteen-Year Follow-up of a Randomized Trial. JAMA. 1997;278(8):637–43. DOI: 10.1001/jama.1997.03550080047038

5. Mejdoubi J, van den Heijkant SC, van Leerdam FJ, Heymans MW, Crijnen A, Hirasing RA. The effect of VoorZorg, the Dutch nurse–family partnership, on child maltreatment and development: a randomized controlled trial. PLOS ONE 2015;10:e0120182. https://doi.org/10.1371/journal.pone.0120182

6. Robling M, Bekkers MJ, Bell K, Butler CC, Cannings-John R, Channon S, et al. Effectiveness of a nurse-led intensive home-visitation programme for first-time teenage mothers (Building Blocks): A pragmatic randomised controlled trial. Lancet. 2016;387:146–55. Available from: http://www.thelancet.com/journals/lancet/article/PIIS0140-6736(15)00392-X/fulltext

7. Lugg-Widger F, Cannings-John R, Channon Sue, Fitzsimmons D, Hood K, Jones K, et al. Assessing the medium-term impact of a home-visiting programme on child maltreatment in England: protocol for a routine data linkage study. BMJ Open. 2017;7(e015728) . Available from: http://bmjopen.bmj.com/content/bmjopen/7/7/e015728.full.pdf

8. Robling M, Bekkers MJ, Bell K, Butler CC, Cannings-John R, Channon S, et al. The Building Blocks Trial (Main report). 2014. [cited 2017 Jul 17] Available from: https://www.cardiff.ac.uk/__data/assets/pdf_file/0009/504729/Building-Blocks-Full-Study-Report.pdf

9. Owen-Jones E, Bekkers M-J, Butler CC, Cannings-John R, Channon S, Hood K, et al. The effectiveness and cost-effectiveness of the Family Nurse Partnership home visiting programme for first time teenage mothers in England: a protocol for the Building Blocks randomised controlled trial. BMC Pediatr. 2013;13:1. DOI: 10.1186/1471-2431-13-114

10. Olds D. Prenatal and Infancy Home Visiting by Nurses: From Randomized Trials to Community Replication. Prevention Science, Vol 3, No 3 2002 DOI: 10.1023/a:1019990432161

11. Robling M, Cannings-John R, Channon S, Hood K, Moody G, Poole R, et al. What is usual care for teenagers expecting their first child in England? A process evaluation using key informant mapping and participant survey as part of the Building Blocks randomised controlled trial of specialist home visiting. BMJ Open. 2018;8(5). doi:10.1136/bmjopen-2017-020152

12. Lyons RA, Jones KH, John G, Brooks CJ, Verplancke J-P, Ford D V, et al. The SAIL databank: linking multiple health and social care datasets. BMC Med Inform Decis Mak. 2009;9(9) https://doi.org/10.1186/1472-6947-9-3

13. Jones KH, Ford D V., Jones C, Dsilva R, Thompson S, Brooks CJ, et al. A case study of the Secure Anonymous Information Linkage (SAIL) Gateway: A privacy-protecting remote access system for health-related research and evaluation. J Biomed Inform. 2014;50:196–204 https://doi.org/10.1016/j.jbi.2014.01.003

For peer review only - http://bmjopen.bmj.com/site/about/guidelines.xhtml

14. Jones KH, Ford DV, Thompson S, Lyons R. A Profile of the SAIL Databank on the UK Secure Research Platform. Int J Popul Data Sci. 2019;4(2)https://doi.org/10.23889/ijpds.v4i2.1134

15. Department for Education. Children In Need in England, including their characteristics and further information on children who were the subject of a child protection plan (2009-10 Children in Need census, Final. 2010. [cited 2020 Jun 30] Available from: https://www.gov.uk/government/statistics/children-in-need-in-england-including-their-characteristics-and-further-information-on-children-who-were-the-subject-of-a-child-protection-plan-2009

16. HM Treasury. The Green Book, Central Government Guidance on Appraisal and Evaluation. 2020. [cited 2020 Dec 7]. Available from: https://assets.publishing.service.gov.uk/government/uploads/system/uploads/attachment_data/file/938046/The_Green_Book_2020.pdf

17. Lugg-Widger F, Cannings-john R, Angel L, Moody G, Segrott J, Kenkre J, et al. Assessing the impact of specialist home visiting upon maltreatment in England : a feasibility study of data linkage from a public health trial to routine health and social care data. Pilot feasibility Stud. 2018;4(1):98. https://doi.org/10.1186/s40814-018-0294-4

18. Department for Education. Phonics screening check and Key stage 1 assessments in England, 2016 (SFR 42/2016). London: Department for Education; 2016. [cited 2020 Dec 7]. Available from: https://assets.publishing.service.gov.uk/government/uploads/system/uploads/attachment_data/file/577806/SFR42_Phonics_KS1_2016.pdf

19. Olds D. Building evidence to improve maternal and child health. Vol. 387, The Lancet. Lancet Publishing Group; 2016. p. 105–7 [cited 2020 Jul 23]. Available from: http://dx.doi.org/10.1016/S0140-6736

20. Barlow J, Barnes J, Sylva K, Fonagy P, Fearon P. Questioning the outcome of the Building Blocks trial. Vol. 387, The Lancet. Lancet Publishing Group; 2016. p. 1615–6 [cited 2020 Jul 23]. Available from: http://www.dh.gov.uk/

21. Robling M, Torgerson D, Pickett K, Channon S, Sanders J. Questioning the outcome of the Building Blocks trial - Authors' reply. Vol. 387, The Lancet. Lancet Publishing Group; 2016. p. 1616–7 [cited 2020 Jul 23]. Available from: http://www.dh.gov.uk/

22. Olds D, Henderson CR, Kitzman H, Cole R. Effects of prenatal and infancy nurse home visitation on surveillance of child maltreatment. Pediatrics 1995; 95; 365-372.

23. Williams, VN, Ayele, R, Shimasaki, S, Tung, GJ, Olds, D. "Risk assessment practices among home visiting nurses and child protection caseworkers in Colorado, United States: A qualitative investigation." Health & Social Care in the Community. 2019;27(5): 1344-52. doi.org/10.1111/hsc.12773

24. Drake B, Jonson-Reid M, Kim H. Surveillance bias in child maltreatment: a tempest in a teapot. Int J Environ Res Public Health 2017;14:E971. https://doi.org/10.3390/ijerph14090971

25. Chaffin M, Bard D. Impact of intervention surveillance bias on analyses of child welfare report outcomes. Child Maltreat 2006;11:301–12. https://doi.org/10.1177/107755950629

26. Zielinski DS, Eckenrode J, Olds DL. Nurse home visitation and the prevention of child maltreatment: Impact on the timing of official reports. Dev Psychopathol. 2009;21(2):441–53 [cited 2019 Aug 2]. Available from: https://www.cambridge.org/core/product/identifier/S0954579409000248/type/journal_article

For peer review only

27. Olds DL, Henderson Jr CR, Kitzman H. Does prenatal and infancy nurse home visitation have enduring effects on qualities of parental caregiving and child health at 25 to 50 months of Life? Pediatrics 1994; 93; 89-98.

28. Hospital discharges and length of stay statistics. [cited 2021 Jul 30] Available from: https://ec.europa.eu/eurostat/statistics-explained/index.php?title=Hospital_discharges_and_length_of_stay_statistics#Average_length_of_hospital_stay_for_in-patients

29. Heys M, Rajan M, Blair M. Length of paediatric inpatient stay, socio-economic status and hospital configuration: a retrospective cohort study. BMC Health Services Research 2017 17: 274 DOI 10.1186/s12913-017-2171-x

30. Livingston JK, Grigorian A, Kuza CM, Lekawa M, Bernal N, Allen A, Nahmais J. Non‑accidental trauma increases length of stay and mortality in pediatric trauma. Pediatric Surgery International (2019) 35:779–784 doi.org/10.1007/s00383-019-04482-5

31. Institute of Health Visiting. Health Visiting in England: State of Health Visiting in England: Results from a survey of 1040 practising health visitors. 2020 [cited 2021 May 21]. Available from: https://www.activematters.org/wp-content/uploads/2020/03/State_of_Health_Visiting_survey.pdf

32. Michie S, van Stralen MM, West R. The behaviour change wheel: A new method for characterising and designing behaviour change interventions. Implementation Science 2011; 6: 42 doi: 10.1186/1748-5908-6-42

33. Moody G, Cannings-John R, Hood K, Kemp A, Robling M. Establishing the international prevalence of self-reported child maltreatment: A systematic review by maltreatment type and gender. Vol. 18, BMC Public Health. BioMed Central Ltd.; 2018. p. 1164 [cited 2020 Jul 23]. Available from: https://bmcpublichealth.biomedcentral.com/articles/10.1186/s12889-018-6044-y

34. Duncan GJ, Dowsett CJ, Claessens A, Magnuson K, Huston AC, Klebanov P, et al. School Readiness and Later Achievement. Dev Psychol. 2007;43(6):1428–46.

35. Duncan G, Magnusson K. Early childhood interventions for low-income children. Focus (Madison). 2014;31.

36. Duncan G, Magnuson K. Investing in Preschool Programs. J Econ Perspect. 2013;27(2):109–31.

37. Filene JH, Kaminski JW, Valle LA, Cachat P. Components associated with home visiting program outcomes: A meta-analysis. Pediatrics. 2013;132(SUPPL.2):S100–9 [cited 2021 Jan 8]. Available from: www.pediatrics.org/cgi/doi/10.1542/peds.2013-1021H

38. Olds DL, Kitzman H, Cole R, Robinson J, Sidora K, Luckey DW, et al. Effects of Nurse Home-Visiting on Maternal Life Course and Child Development: Age 6 Follow-Up Results of a Randomized Trial. Pediatrics. 2004;114(6):1550–9. Available from: http://pediatrics.aappublications.org/cgi/doi/10.1542/peds.2004-0962

39. Heckman JJ, Holland ML, Makino KK, Pinto R, Rosales-Rueda M. An Analysis of the Memphis Nurse-Family Partnership Program. NBER Work Pap. 2017;23610. Available from: http://www.nber.org/papers/w23610%0Ahttp://www.nber.org/papers/w23610

40. Family Nurse Partnership National Unit & Dartington Service Design Lab. FNP ADAPT: Using evidence, pragmatism and collaboration to change the FNP programme in England. London, UK; 2020. Available from: www.fnp.nhs.uk

41. The Family Nurse Partnership |. [cited 2020 Jul 23]. Available from: https://www.fnp.nhs.uk/

For peer review only - http://bmjopen.bmj.com/site/about/guidelines.xhtml

For peer review only

Figure Legend

Figure 1 Participant flow chart

Describing follow-up within the BB0:0-2 trial and formation of the BB:2-6 study cohort. MW=Mandatory withdrawals; EW=elective withdrawals; M=Mother; C=Child, FNP=Family Nurse Partnership (trial arm); UC=Usual care (trial arm); BB:0-2=Building Blocks Randomised Controlled Trial; BB:2-6=Building Blocks Cohort Study; NPD=National Pupil Database

For peer review only

Figure 1 Flow chart

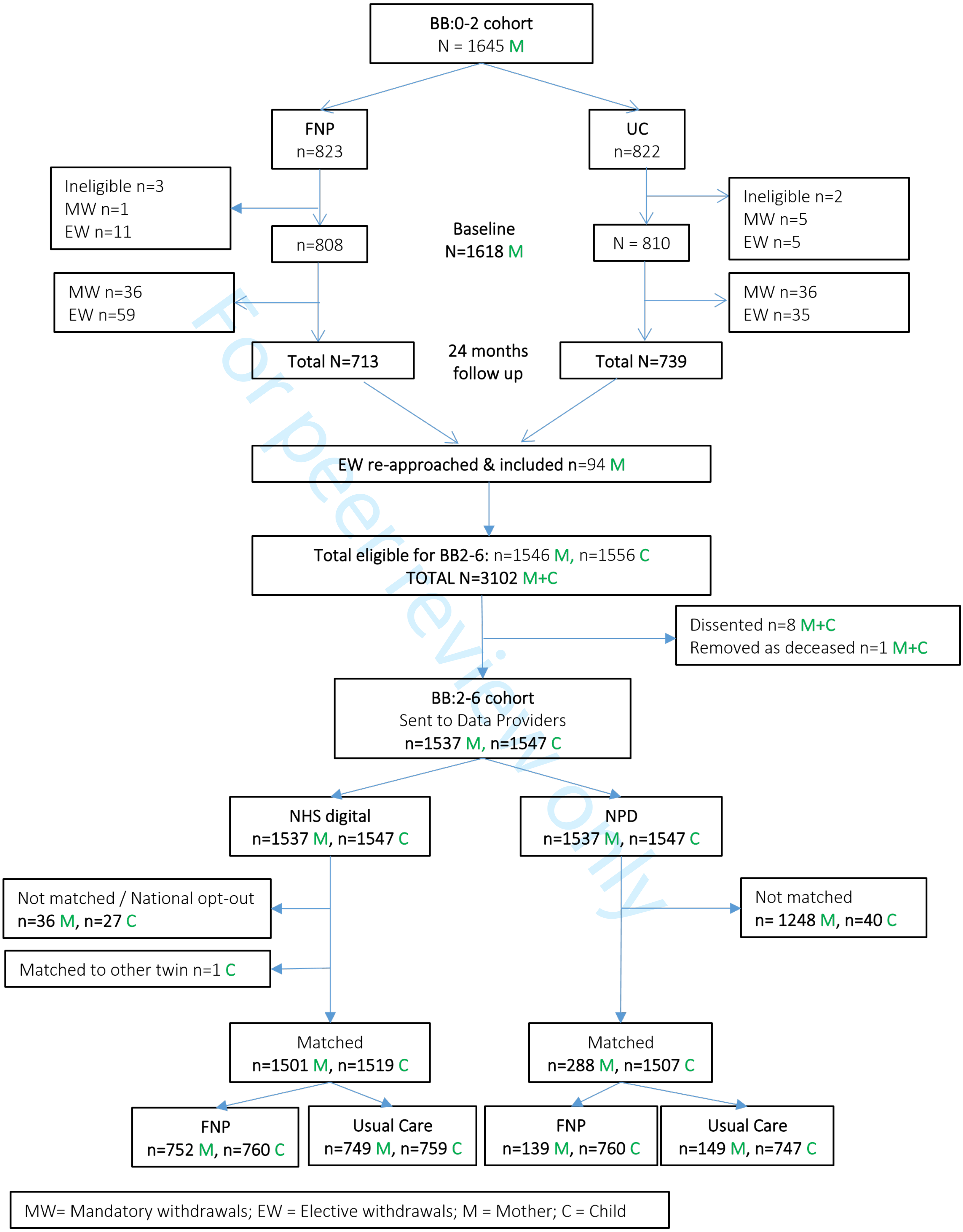

Supplementary appendix

I. Number of valid FNP visits received
- Table S1 BB:2-6 study population data: Valid FNP visits per phase

II. Study assessment domain, outcomes, principal data providers and data obtained
- Table S2 BB:2-6 Study assessment domains, key and exploratory outcomes and principal data providers

III. Data accrued from providers
- Table S3 Numbers (%) of mothers and children with data returned by dataset

IV. Further details of Child in Need outcome

V. Representativeness of study sample
- Table S4 Maternal characteristics of those in BB:0-2 compared to those in the BB:2-6
- Table S5 Child characteristics of those in the BB:0-2 study compared to those in the BB:2-6 study

VI. Descriptive secondary outcomes and additional Key Stage 1 results

- Table S6 Child In Need referrals, Children's Social Services referrals and hospital admissions: descriptives
- Table S7 Emergency attendance to hospital by injury or ingestion, by trial arm
- Table S8 Admissions by injury/ingestion type and trial arm
- Table S9 Hospital admissions for injuries and ingestions by trial arm and broken down by age categories
- Table S10 Child health, developmental and educational: descriptives
- Table S11 Children reaching the expected standard in KS1 assessments by trial arm and academic year
- Figure S1 Distribution of children's month of birth by trial arm
- Table S12 Comparison of parameter estimates before and after adjustment for child's month of birth

VII. Results of planned sub-group analysis
- Table S13 Subgroup analyses for referrals to Children's social care services
- Table S14 Subgroup analyses for child in need status
- Table S15 Predefined subgroup analyses on Early Years Assessment total point score
- Table S16 Subgroup analyses on KS1 assessments reaching the expected standard (RES)
- Table S17 Subgroup analyses on reaching the expected standard in the writing KS1 assessment for 2016/17 academic year

VIII. Treatment efficacy

IX. Exploration of surveillance bias upon Child In Need outcome assessment

- Table S18 Baseline maternal characteristics, school readiness outcomes for children referred to CSC and classed as a Child In Need, before the age of 4 years old by intervention arm.

X. Missing data

XI. Cost Consequence Analysis

For peer review only

- Table S19 Cost-consequence balance sheet for the family-nurse based intervention for the four years between children's ages 2 and 6.

For peer review only

For peer review only - http://bmjopen.bmj.com/site/about/guidelines.xhtml

I. Number of valid FNP visits received

Of the 766 women in the FNP arm in the BB:2-6 cohort, 69 did not enrol for FNP. For the remaining 697 who were randomised and enrolled, 695 had received at least one valid visit and two did not receive any valid visits across the three phases (pregnancy, infancy and toddlerhood). The descriptives in Table S1 are based on 697 women. A greater proportion of women received no visits during the infancy and toddler phases (40 (5.7%) and 98 (14.1%) respectively) compared to the pregnancy phase (6 (0.7%)). This is similar to that reported in the BB:0-2 cohort with comparable median valid visits and proportions reaching the fidelity goals (see Lancet Supplementary appendix Table S11.3).[1]

Table S1 BB:2-6 study population data: Valid FNP visits per phase

	**Pregnancy**	**Infancy**	**Toddlerhood**
N women randomised and enrolled to FNP	697	697	697
Women not receiving any valid visits n(%)	6 (0·7)	40 (5·7)	98 (14·1)
Women receiving at least one valid visit n(%)	691 (99·3)	657 (94·3)	599 (85·9)
Valid visits received			
Median (25th to 75th centile)	10 (8 to 12)	19 (15 to 22)	13 (10 to 16)
Mean (SD)	9·74 (3·42)	18·7 (5·97)	13·28 (5·46)
Reaching fidelity goal of: n(%)			
80%+	401/697 (57·5)	-	-
65%+	-	364/697 (52·2)	-
60%+	-	-	297/697 (42·6)

[1] Robling M, Bekkers MJ, Bell K, Butler CC, Cannings-John R, Channon S, et al. Effectiveness of a nurse-led intensive home-visitation programme for first-time teenage mothers (Building Blocks): A pragmatic randomised controlled trial. Lancet. 2016;387:146–55. [accessed 2019 Nov 12]. Available from: http://www.thelancet.com/journals/lancet/article/PIIS0140-6736(15)00392-X/fulltext

For peer review only - http://bmjopen.bmj.com/site/about/guidelines.xhtml

II. Study assessment domain, outcomes and principal data providers

Table S2 BB:2-6 Study assessment domains, key and exploratory outcomes and principal data providers

Domains	Outcomes	Maternal	Child	HES	ONS	NPD	Data source	Analysis
Primary: Child in Need status recorded at any time during the follow-up period.	CIN status		✓			✓	CIN	LO
	Exploratory:<br>*Age at first CIN referral*<br>*Number of CIN referrals per child*		 ✓<br>✓			 ✓<br>✓	 CIN<br>CIN	 *SURV*<br>*PO*
Secondary:<br>(i) Objective measures of maltreatment	CIN categorisation<br>CIN duration		✓<br>✓			✓<br>✓	CIN<br>CIN	MNOM<br>SURV
	Referral to Children's social care services		✓			✓	CIN	LO
	Exploratory: Age at first referral		✓			✓	CIN	SURV
	Child Protection Plan<br>Child Protection Plan categorisation		✓<br>✓			✓<br>✓	CIN<br>CIN	LO<br>MNOM
	Looked after status<br>CLA period of care<br>Legal status of CLA		✓<br>✓<br>✓			✓<br>✓<br>✓	CLA<br>CLA<br>CLA	LO<br>PO<br>D
	Cause of death		✓		✓		ONS- deaths	D
(ii) Associated measures of maltreatment	Injuries and ingestions:<br>Emergency attendance<br>Admission to hospital<br>Attendance and/or admission<br>DNA appointments		 ✓<br>✓<br>✓<br>✓	 ✓<br>✓<br>✓<br>✓			 A&E<br>IP<br>A&E, IP<br>OP	 LO<br>LO<br>LO<br>LO
	Exploratory:							

For peer review only

**Domains**	**Outcomes**	**Maternal**	**Child**	**HES**	**ONS**	**NPD**	**Data source**	**Analysis**
	Number of admissions per child		✓	✓			IP	PO
	Length of hospital admission stay		✓	✓			IP	D
(iii) Maternal outcomes	Subsequent pregnancy	✓		✓			OP, IP, Ab	LO
	Exploratory:							
	Number of births	✓		✓			IP	PO
	Inter-birth interval between first and second child	✓		✓			IP	SURV
(iv) Costs	Health and Social Care resource use	✓	✓	✓		✓	OP, IP, A&E matched with NHS reference costs	D
(v) Child health, developmental and educational outcomes	Special educational needs		✓				PLASC, AP, PRU Census, EYC	LO
	provision		✓	✓		✓		
	Disability		✓			✓	NA	NA
	Early education attendance		✓			✓	PLASC, EYC	LO
	School attendance		✓			✓	Absence	LO
	Early Years assessment		✓			✓	EYFSP	LIN, LO
	KS1 attainment					✓	KS1	LO
	Exploratory:							
	Early education attendance by School or an Ofsted registered PVI establishment		✓			✓	PLASC, EYC	D
	Earliest type of day care		✓			✓	EYC	D

Ab - Abortions; A&E – Accident and Emergency; AP – Alternative Provision; CIN – Child in Need; CLA – Child Looked After; D – Descriptive; DNA – Did not attend; EYC – Early Years Census; EYFSP –Early years foundation stage profile; FNP – Family Nurse Partnership; HES – Hospital Episode Statistics; IP - Inpatients; KS1 – Key Stage 1; LIN – Linear regression; LO – Logistic; MNOM - multinomial NA - Not available; NPD – National Pupil Database; ONS – Office for National Statistics; OP – Outpatients; PLASC – Pupil Level and School Census; PO-Poisson; PRU – Pupil Referral Unit Census.; PVI – Private, Voluntary or Independent (PVI) establishment; SURV – Survival.

For peer review only - http://bmjopen.bmj.com/site/about/guidelines.xhtml

III. Data accrued from providers

Table S3 Numbers (%) of mothers and children with data returned by dataset

	**Mothers**	**Children**
**Sent for matching to DfE and NHSD**	**N=1537**	**N=1547**
**Department for Education (DfE)**		
Not matched	1248 (81·2)	40 (2·6)
Match to any DfE dataset	289 (18·8)	1507 (97·4)
Pupil Level Annual School Census (PLASC)	<5	1498 (96·8)
Child in Need (CIN)	278 (18·1)	414 (26·8)
Children looked after (CLA)	61 (4·0)	52 (3·4)
Early years foundation stage profile (EYFSP)	N/A	1476 (95·4)
Key Stage one (KS1)	N/A	1472 (95·2)
Early Years Census (EYC)	N/A	642 (41·5)
Pupil referral unit (PRU) Census	<5	0
Alternative Provision (AP)	<5	<5
Absence	<5	1494 (96·6)
**NHS Digital (NHSD)**		
Not matched	36 (2·3)	27 (1·7)
Duplicate match	0	1 (0·06)
Matched to any NHSD dataset	1501 (97·7)	1519 (98·3)
Accident and Emergency (A&E)	1360 (88·5)	1392 (90·0)
Admitted Patient Care (Inpatients) (APC)	1498 (97·5)	1507 (97·4)
Outpatient (OP)	1495 (97·3)	1189 (76·9)
**Sent for matching to Department of Health and Social Care (DHSC)**	**N=1532**	**0**
Abortions dataset	1532 (100)	NA

For peer review only - http://bmjopen.bmj.com/site/about/guidelines.xhtml

IV. Further details of Child in Need outcome.

In our primary comparative analysis we examined whether the firstborn ('BB:0-2 child') had ever been referred to social services and classed as a child in need at any point between birth and 6 years of age. There is no flag in the Child In Need dataset to identify whether a child is in need after they have been referred to social services. Therefore, CIN was defined (as per DfE methodology)[1] as all children referred to social services, excluding cases where:

- the referral is flagged as being a referral resulting in no further action; or
- the only activity recorded is an assessment and the reason for the closure of the case being that case was closed after assessment with no further action.

1. Department for Education. Characteristics of children in need in England: 2014 to 2015. 2015. [accessed 2019 Nov 12]. Available from: https://www.gov.uk/government/uploads/system/uploads/attachment_data/file/469740/SFR41-2015_Methodology.pdf

V. Representativeness of study sample

Scope and purpose: We assessed whether the study sample for BB:2-6 were similar to women recruited to the original BB:0-2 trial.
Conclusion: The BB:2-6 and BB:0-2 cohorts are similar in terms of maternal (Table S4) and baby characteristics (Table S5).

Table S4 Maternal characteristics of those in BB:0-2 compared to those in the BB:2-6

	BB:0-2 N=1618	BB:2-6 N=1537
**Minimisation variables at randomisation**		
**Gestational age at baseline** n(%)		
16 weeks or more	658 (40·7)	636 (41·4)
Less than 16 weeks	959 (59·3)	900 (58·6)
**Smoking status** n(%)		
No	860 (53·2)	822 (53·5)
Yes	758 (46·8)	715 (46·5)
**Language** n(%)		
English	1611 (99·6)	1530 (99·5)
Other	6 (0·4)	6 (0·4)
Missing	*1 (0·0)*	*0 (0·0)*

For peer review only - http://bmjopen.bmj.com/site/about/guidelines.xhtml

	BB:0-2 N=1618	BB:2-6 N=1537
**Trial arm** n(%)		
FNP	808 (49·9)	766 (49·8)
Usual care	810 (50·1)	771 (50·2)
**Age at recruitment (years) Median (25th to 75th centile)**	17·9 (16·9 to 18·8)	17·9 (16·9 to 18·8)
**Ethnicity** n(%)		
White background	1425 (88·1)	1355 (88·2)
Mixed background	89 (5·5)	82 (5·3)
Asian background	27 (1·7)	25 (1·6)
Black background	71 (4·4)	69 (4·5)
Chinese or Other background	6 (0·4)	6 (0·4)
**Relationship status with baby's father** n(%)		
Married	20 (1·2)	16 (1·0)
Separated	165 (10·2)	150 (10·0)
Closely involved/boyfriend	1222 (75·5)	1168 (76·0)
Just friends	211 (13·0)	203 (13·2)
**Live with father of baby** n(%)		
Yes	368 (22·7)	348 (22·6)
No	1112 (68·7)	1061 (69·0)
Missing	138 (8·5)	128 (8·3)
**NEET status**[a] n(%)		
Yes	663 (41·0)	634 (41·2)
No	717 (44·3)	678 (44·1)
Missing	*4 (0·2)*	*4 (0·3)*
Not applicable (aged 16 years or younger)	*234 (14·5)*	*221 (14·4)*
**Socio-economic status: Overall Index of Multiple Deprivation (IMD) Score**[b] Median (25th to 75th centile)	N=1606<br>38·2 (25·2 to 52·0)	N=1525<br>38·1 (25·0 to 52·1)
**Maternal health and wellbeing**		
**Generalized self-efficacy scale (score 10 to 40)**[c] Median (25th to 75th centile)	N=1592<br>30·0 (28·0 to 33·0)	N=1533<br>30·0 (28·0 to 33·0)
**Adaptive functioning: Difficulty in at least one basic skill** n(%)		
Yes	430 (26·6)	397 (25·8)
No	1185 (73·2)	1138 (74·0)
Missing	*3 (0·2)*	*2 (0·1)*
**Adaptive functioning: Had 3 or less life skills** n(%)		
Yes	434 (26·8)	403 (26·2)
No	1178 (72·8)	1129 (73·5)
Missing	*6 (0·4)*	*5 (0·3)*
**Adaptive functioning: At least one burden** n(%)		
Yes	476 (29·4)	451 (29·3)
No	1131 (69·9)	1075 (69·9)
Missing	*11 (0·7)*	*11 (0·7)*
**Smoking** (Participant self-reported) n(%)		
Ever smoked: Yes	1291 (79·8)	1227 (79·8)
No	327 (20·0)	310 (20·2)
Missing	*0 (0)*	*0 (0)*
**Child characteristics**	**BB:0-2 sample N=1510**	**BB:2-6 sample N=1547**
**Trial arm** n(%)		

For peer review only - http://bmjopen.bmj.com/site/about/guidelines.xhtml

	BB:0-2 N=1618	BB:2-6 N=1537
FNP	742 (49·1)	773 (50·0)
Usual Care	768 (50·9)	774 (50·0)
**Gender** n(%)		
Male	777 (51·5)	787 (50·9)
Female	732 (48·5)	758 (40·1)
Missing	1	72[d]
**Birthweight (grams) Mean (sd)**	3207·3 (599·6)	3219·6 (580·7)
Missing[c]		71
**Neonatal unit (NNU) admission** n(%)		
Yes	149 (10·0)	142 (9·2)
Missing from data collection	13	11
Missing	Not applicable	71[d]

[a] Definition of NEET status: Not in education employment or training (applicable only to those whose age at end of previous academic year at time of baseline interview was >16)
[b] Higher IMD score indicated more deprivation. Mean IMD Score for England in 2010 was 21·67 [60];
[c] Higher score indicates higher level of self-efficacy
[d] Mothers were withdrawn in BB:0-2 but are included in BB:2-6

Table S5 Child characteristics of those in the BB:0-2 study compared to those in the BB:2-6 study

	BB:0-2 sample N=1510	BB:2-6 sample N=1547
**Trial arm** n(%)		
FNP	742 (49·1)	773 (50·0)
Usual Care	768 (50·9)	774 (50·0)
**Gender** n(%)		
Male	777 (51·5)	787 (50·9)
Female	732 (48·5)	758 (40·1)
Missing	1	72[a]
**Birthweight (grams) Mean (sd)**	3207·3 (599·6)	3219·6 (580·7)
Missing[a]		71
**Neonatal unit (NNU) admission** n(%)		
Yes	149 (10·0)	142 (9·2)
Missing from data collection	13	11
Missing[a]	Not applicable	71*

[a] Mothers were withdrawn in BB:0-2 but are included in BB:2-6

For peer review only - http://bmjopen.bmj.com/site/about/guidelines.xhtml

VI. Descriptive secondary outcomes and additional Key Stage 1 results

Table S6 Child In Need referrals, Children's Social Services referrals and hospital admissions: descriptives

Outcome	FNP	Usual care (UC)
**Child in Need (CIN) Referrals**	***N=161***	***N=162***
Age at first CIN referral (days)		
Pre birth to < 1year	12 (7.5%)	5 (3.1%)
1 to <2 years	6 (3·7%)	6 (3·7%)
2 to <3 years	15 (9·4%)	28 (17·3%)
3 to <4 years	27 (16·9%)	23 (14·2%)
4 to <5 years	47 (29·4%)	39 (24·1%)
5 to <6 years	33 (20·6%)	41 (25·3%)
6 to <7 years	20 (12·5%)	20 (12·3%)
Missing	*1*	*0*
Unique* number of CIN referrals per child		
1 case	110 (68·3%)	110 (67·9%)
2 cases	42 (26·1%)	42 (25·9%)
3 or more cases	9 (5·5%)	10 (6·1%)
CIN cases closed at 31st March 2017	116 (72·0%)	110 (67·9%)
**Referral to Children's social care services (CSCS)**	***N=760***	***N=746***
Total referrals made N	339	354
Ratio referrals:children	1·65	1·70
Referrals made per child	***N=206***	***N=208***
1	159 (77·2%)	140 (67·3%)
2	34 (16·5%)	51 (24·5%)
3 to 5	13 (6·3%)	27 (8·2%)
**Unit=referral (referred to CSCS encompass any referral)**	***n=339***	***n=354***
Source of referral		
Individual	29 (8·8%)	34 (10·0%)
School/Education	150 (45·7%)	136 (40·0%)
Health services	114 (34·8%)	132 (38·8%)
Local Authority Services	35 (10·7%)	38 (11·2%)
Missing	*11*	*14*
Primary need of referrals		
Abuse or neglect	198 (58·9%)	205 (57·9%)
Family dysfunction	72 (21·4%)	88 (24·9%)
Family in acute stress	19 (5·7%)	21 (5·9%)
Low income/absent parenting/ socially unacceptable behaviour	11 (3·3%)	7 (2·0%)
Parental/Childs disability illness	14 (4·2%)	11 (3·1%)
Cases other than Child in Need	11 (2·3%)	12 (3·4%)
Not stated	11 (3·3%)	9 (2·5%)
Missing	*3*	*1*
Legal status of child looked after†	***N=17***	***N=23***
Interim care order	6 (35·3%)	8 (34·8%)
Full care order	5 (29·4%)	6 (26·1%)

For peer review only - http://bmjopen.bmj.com/site/about/guidelines.xhtml

Outcome	FNP	Usual care (UC)
Placement order granted	<5	<5
Single period of accommodation under section 20	<5	<5
**Injuries and Ingestions: Admission to hospital**	**N=760**	**N=759**
N unique admissions	109	119
Ratio admissions: children	1.22	1.20
Length of hospital stay (days)	0·5‡ (0·5 to 1·0)	0·5 (0·5 to 1·0)
	**N=760**	**N=746**
Death	<10	

Data are n (%), n/N (%), or median (25th to 75th centile) unless otherwise stated. <5 = numbers suppressed· FNP = Family Nurse Partnership Programme + Usual Care. * A unique referral is counted as a distinct referral date per child. † Legal status for most recent CLA episode (Of n=52 children with a recent CLA episode, n=12 cases have a missing status). ‡ 0·5 days indicates an admission and discharge on the same day (could be up to 1 day in hospital). Source: National Pupil Database, The Department for Education

For peer review only

Table S7 Emergency attendance to hospital by injury or ingestion, by trial arm

	Trial arm, n (%)	
**Injury / ingestion**	**FNP (N = 911)**	**Usual Care (N = 881)**
Laceration	136 (14.9)	158 (17.9)
Contusion/abrasion	90 (9.9)	96 (10.9)
Soft-tissue inflammation	63 (6.9)	64 (7.3)
Head injury	68 (29.4)	249 (28.3)
Dislocation	109 (12.0)	84 (9.5)
Sprain/muscle/tendon injury	35 (3.8)	33 (3.8)
Nerve/vascular injury	74 (8.1)	60 (6.8)
Burns and scalds/electric shock	48 (5.3)	37 (4.2)
Foreign body	40 (4.4) 4	6 (5.2)
Bites and stings	12 (1.3)	20 (2.3)
Poisoning	36 (4.0)	34 (3.9)

For peer review only - http://bmjopen.bmj.com/site/about/guidelines.xhtml

Table S8 Admissions by injury/ingestion type and trial arm: For children of all ages (0-6 years) and for children aged 0-2 years old

	Trial arm, n (%)			
	**All children**		**Children aged 0-2 years***	
**Diagnosis ICD-10 code**	**FNP**<br><br>**(n=109)**	**Usual care**<br><br>**(n=119)**	**FNP**<br><br>**(n=42)**	**Usual Care**<br><br>**(n=63)**
S00–S09 Injuries to the head	52 (47.7)	61 (51.3)	39 (61.9)	27 (64.3)
S20–S29 Injuries to the thorax	9 (8.3)	6 (5.0)		
S30–S39 Injuries to the abdomen, lower back, lumbar spine and pelvis				
S40–S49 Injuries to the shoulder and upper arm			7 (16.7)	7 (11.1)
S50–S59 Injuries to the elbow and forearm				
S60–S69 Injuries to the wrist and hand	8 (7.3)	5 (4.2)		
S70–S79 Injuries to the hip and thigh	11 (10.1)	11 (9.2)		
S80–S89 Injuries to the knee and lower leg				
S90–S99 Injuries to the ankle and foot				
T00–T07 Injuries involving multiple body regions	5 (4.6)	10 (8.4)	15 (35.7)	7 (11.1)
T08–T14 Injuries to unspecified part of trunk, limb or body region				
T20–T32 Burns and corrosions				
T15–T19 Effects of foreign body entering through natural orifice	5 (4.6)	8 (6.7)		
T36–T50 Poisoning by drugs, medicaments and biological substances	11 (10.1)	8 (6.7)		
T51–T65 Toxic effects of substances chiefly non-medicinal as to source (sting, alcohol, solvents etc.)	8 (7.3)	10 (8.4)		
T66–T78 Other and unspecified effects of external causes				

* We undertook further exploratory analyses of length of stay following admission for:
(i) children aged under 1 year old: The median (25th to 75th quartile) length of stay (in days) was, for the n=20 children in FNP group 0.75 (0.5 to 2) and for the n=29 children in the UC group 0.5 (0.5 to 2) with no difference observed (Incidence rate ratio (IRR) 0.68, 95% CI 0.24 to 1.98, Ref = UC).
(ii) children aged between 1 and under 2 years old: The median (25th to 75th centile) length of stay (in days) was, for the n=21 admissions in FNP group, 0.5 (0.5 to 1) and for the n=34 admissions in UC group 0.5 (0.5 to 1) with no observed difference (IRR 0.36, 95% CI 0.05 to 2.43, Ref = UC).

For peer review only

Table S9. Hospital admissions for injuries and ingestions by trial arm and broken down by age categories

	**FNP N=760**	**Usual care N=759**
No hospital admissions with an injury or an ingestion (0-6 years)	671 (88.3%)	660 (87.0%)
At least one hospital admission with an injury or an ingestion	89 (11.7%)	99 (13.0%)
By age at admission:		
**Under 1 year**		
Children with no admission n(%)	744 (97.9%)	732 (96.4%)
Children with at least one admission n(%)	16 (2.1%)	27 (3.6%)
95% CI	1.3 to 3.3%	2.5 to 5.1%
Total number of admissions (different days)	20	29
Ratio of admissions: child	1.25	1.08
	n=20	n=29
Length of stay (days) Median (25th to 75th centiles)	0.75 (0.5 to 2)	0.5 (0.5 to 2)
**1 to under 2 years**		
Children with no admission n(%)	738 (97.1%)	725 (95.5%)
Children with at least one admission n(%)	21 (2.8%)	31 (4.1%)
95% CI	1.8 to 4.2%	2.9 to 5.7%
Total number of admissions (different days)	22	34
Ratio of admissions: child	1.05	1.10
	n=21*	n=34
Length of stay (days) Median (25th to 75th centiles)	0.5 (0.5 to 1)	0.5 (0.5 to 1)
**25 months to under 6 years**		
Children with no admission n(%)	699 (92.0%)	709 (93.4%)
Children with at least one admission n(%)	61 (8.0%)	50 (6.6%)
95% CI	6.3 to 10.2%	5.0 to 8.6%
Total number of admissions (different days)	67	56
Ratio of admissions: child	1.10	1.12
	n=65*	n=56
Length of stay (days) Median (25th to 75th centiles)	0.5 (0.5 to 1)	0.5 (0.5 to 1)

* Date of discharge missing – LOS cannot be calculated

LOS=0.5 days indicates an admission and discharge on the same day (could be up to 1 day in hospital).

For peer review only - http://bmjopen.bmj.com/site/about/guidelines.xhtml

Table S10 Child health, developmental and educational: descriptives

Outcome	FNP	Usual care	Absolute risk difference (FNP-Usual care) (95% CI)
**Early educational attendance**	**N=759**	**N=747**	
Children attending education* up to the age of 4 years			
Yes†	758 (99·7%)	747 (100·0%)	-0·3% (-1·0 to 0·3%)
Children attending school up to the age of:			
• 4 years†	753 (99·1%)	744 (99·6%)	-0·5% (-1·5 to 0·4%)
• 3 years	479 (63·0%)	482 (64·5%)	
• 2 years	137 (18·0%)	147 (19·7%)	
Earliest type of day care	***N=334***	***N=308***	
Private	217 (65·0%)	221 (71·8%)	
Voluntary	52 (15·6%)	57 (18·5%)	
Local Authority Day Nursery	36 (10·8%)	22 (7·1%)	
Other‡	29 (8·7%)	8 (2·6%)	
**School attendance**	**N=754**	**N=740**	
Number of academic sessions§	630 (264 to 638)	630 (262 to 638)	
Overall absence rate Median (25th to 75th centiles)	5·08 (2·69 to 8·73)	5·19 (2·73 to 8·41)	
Overall authorised absence rate Median (25th to 75th centiles)	3·50 (1·96 to 6·09)	3·79 (2·02 to 6·11)	
Overall unauthorised absence rate Median (25th to 75th centiles)	1·84 (0·76 to 4·34)	1·57 (0·63 to 3·88)	
**Early Years assessment**	**N=743**	**N=728**	
Achieving good level of development (GLD) ‖:			
• Communication and Language	642 (86·4%)	613 (84·2%)	2·2% (-1·4 to 5·8%)
• Physical Development	676 (91·0%)	643 (88·3%)	2·7% (-0·5 to 5·8%)

For peer review only - http://bmjopen.bmj.com/site/about/guidelines.xhtml

Outcome	FNP	Usual care	Absolute risk difference (FNP-Usual care) (95% CI)
• Personal, Social and Emotional Development	668 (89·9%)	634 (87·1%)	2·8% (-0·4 to 6·1%)
• Literacy	493 (66·4%)	458 (62·9%)	3·4% (-1·4 to 8·3%)
• Maths	545 (73·4%)	516 (70·9%)	2·5% (-2·1 to 7·0%)
Total point score¶			
• Score <34	325 (43·8%)	345 (47·4%)	
• Score = 34	187 (25·2%)	183 (25·1%)	
• Score >34	231 (31·1%)	200 (27·5%)	
**Key Stage 1 assessments**			
Writing academic year 2017/18	**N=242**	**N=245**	
Lower than expected	82 (33·9%)	81 (33·1%)	
Reaching at least the expected standard**	160 (66·1%)	164 (66·9%)	-0·8 (-9·2 to 7·5)
Expected standard	140 (57·9%)	132 (53·9%)	4·0 (-4·8 to 12·7)
Higher standard	20 (8·3%)	32 (13·1%)	-4·8 (-10·4 to 0·7)

Data are n (%), n/N (%), or median (25th to 75th centile) unless otherwise stated. FNP = Family Nurse Partnership Programme + Usual Care.
* School or an Ofsted registered Private, Voluntary or Independent (PVI) establishment;
† = Not estimable; ‡ Childminding Network, Childminder, Registered Independent School, Other; § Total number of sessions over the academic years available (2013 to Spring 2017) and all 6 terms per academic year. ‖ Children achieving a good level of development are those achieving at least the expected level within the prime and specific areas of learning; ¶ Total point score ranges from 17 to 51 with a higher score indicating a better level of development. ** Working at the expected standard and at a greater depth within the expected standard. PLASC (Autumn, Spring and Summer terms), AP, PRU Census, and EYC. If for any of the years no response was recorded then the assumption was that the child was not included in the denominator as either not present on Census day or not in school. Source: National Pupil Database, The Department for Education

For peer review only - http://bmjopen.bmj.com/site/about/guidelines.xhtml

Table S11 Children reaching the expected standard in KS1 assessments by trial arm and academic year

		Academic year 2016/17	Academic year 2017/18
Reading	FNP	302/498 (60.6%)	181/242 (74.8%)
	Usual care	263/487 (54.0%)	180/245 (73.5%)
Maths	FNP	280/498 (56.2%)	179/242 (74.0%)
	Usual care	263/487 (54.0%)	186/245 (75.9%)
Science	FNP	341/498 (68.5%)	196/242 (81.0%)
	Usual care	310/487 (63.7%)	203/245 (82.9%)

Source: National Pupil Database, The Department for Education

Figure S1 Distribution of children's month of birth by trial arm

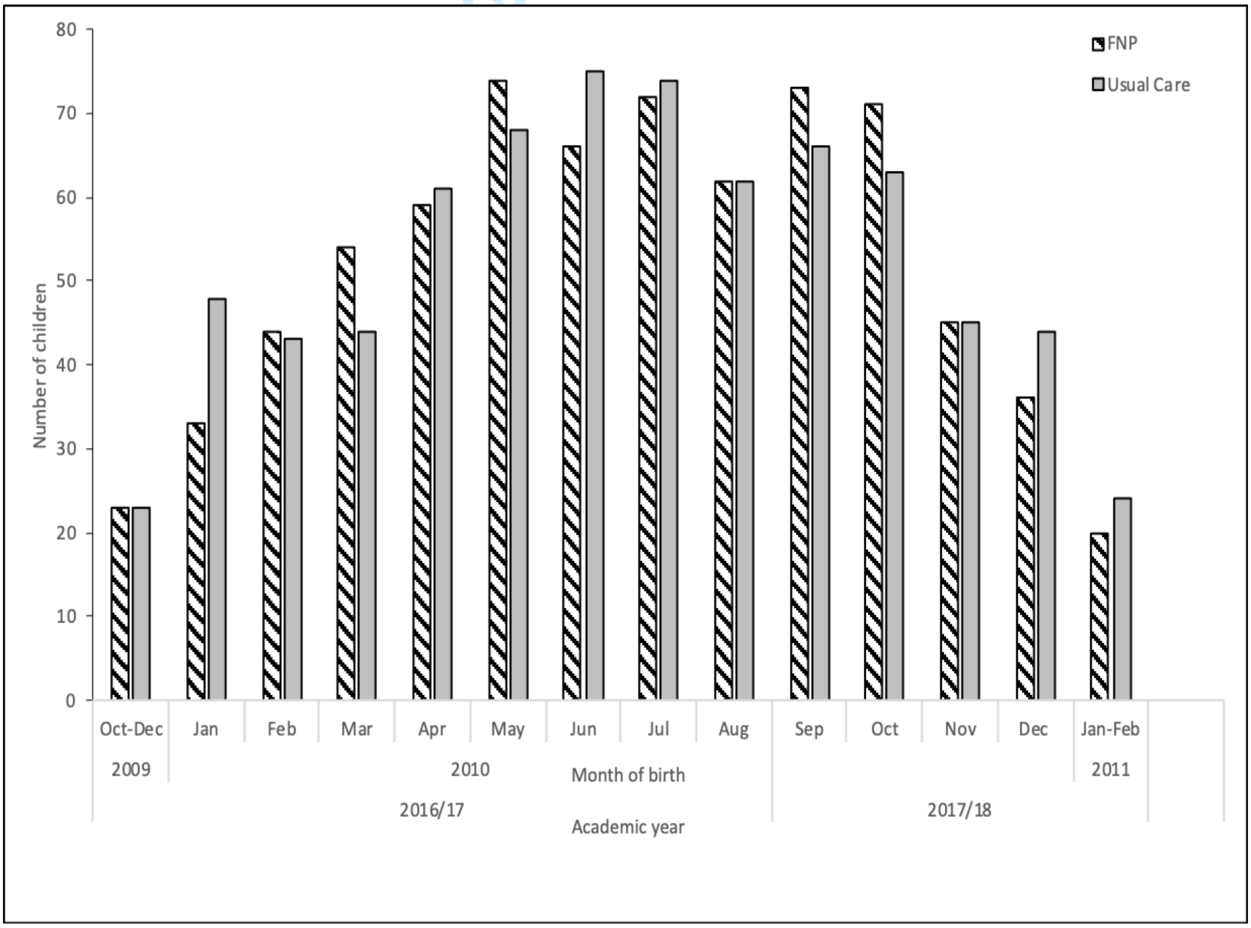

For peer review only - http://bmjopen.bmj.com/site/about/guidelines.xhtml

Table S12 Comparison of parameter estimates before and after adjustment for child's month[a] of birth

	Odds ratio[b] (95% CI), p-values	
	Adjusted for site and minimisation variables[c]	Adjusted for site, minimisation variables[c] and month of birth
**Key Stage 1 outcome**		
**Reading**		
Reaching at least the expected standard	1.23 (0.99 to 1.53), 0.051	1.26 (1.02 to 1.57), 0.035
Expected standard	*1.24 (0.99 to 1.56), 0.056*	*1.27 (1.01 to 1.59), 0.042*
Higher standard	*1.20 (0.88 to 1.65), 0.250*	*1.26 (0.91 to 1.75), 0.161*
**Maths**		
Reaching at least the expected standard	1.04 (0.84 to 1.28), 0.731	1.06 (0.85 to 1.31), 0.613
Expected standard	*1.06 (0.85 to 1.32), 0.611*	*1.07 (0.86 to 1.34), 0.527*
Higher standard	*0.93 (0.64 to 1.35), 0.711*	*0.93 (0.74 to 1.17), 0.522*
**Science**		
Reaching at least the expected standard	1.14 (0.91 to 1.43), 0.254	1.16 (0.92 to 1.47), 0.197
**Writing academic year 2017/18**		
Reaching at least the expected standard	1.24 (0.97 to 1.60), 0.090	1.26 (0.98 to 1.62), 0.076
Expected standard	*1.29 (1.00 to 1.68), 0.054*	*1.30 (1.00 to 1.69), 0.050*
Higher standard	*0.92 (0.51 to 1.64), 0.769*	*0.97 (0.54 to 1.75), 0.917*
**Early Years Assessment**		
Achieving GLD in all five areas of learning[d]	1.26 (1.03 to 1.55), 0.026	1.31 (1.05 to 1.62), 0.015
Achieving GLD in all 17 early learning goals	1.24 (1.01 to 1.52), 0.043	1.27 (1.03 to 1.58), 0.027
Total point score	0.65 (-0.11 to 1.41), 0.094	0.70 (-0.03 to 1.42), 0.060

a Quarter of birth runs from September to November, December to February, March to May, June to August; b Odds ratio represents the FNP arm compared to Usual care; c gestational age, smoking status at recruitment, and first or preferred language; d Children achieving a good level of development (GLD) are those achieving at least the expected level within the prime and specific areas of learning.

Source: National Pupil Database, The Department for Education

For peer review only

VII. Results of planned sub-group analysis

No differential effects due to maternal age, deprivation, participation in employment, education or training, or basic life skills were found in planned sub-group analyses for any of the objective maltreatment outcomes (Table S10 and Table S11). The beneficial impact of FNP on EYFSP total point score was mostly observed for children with mothers aged <16 years rather than those aged ≥16 at study entry but no other differences by study sub-group were found (Table S12).

The differential effects due to maternal age, deprivation, participation in employment, education or training, or basic life skills for KS1 reading, maths and science are displayed in Tables S13a to e and for writing in Table S14. At KS1, sex differences were large with girls outperforming boys in all four subject areas (Table S13a). The intervention effect was greater for boys than girls with a higher proportion reaching the expected standard in the FNP arm, particularly for reading and writing (and strengthened after adjusting for month of birth) and for the writing assessment (academic year 2016/17) this interaction reached statistical significance after adjusting for month of birth (post-hoc analysis). When examining how the intervention effects differed in mothers aged less than 16 years at recruitment compared to mothers aged 16 years or more in each KS1 assessment, differences were observed in maths (Table S13b) and writing (Table S14), with a larger effect seen in mothers aged less than 16 at recruitment, when compared to those aged 16 or more.

Table S13 Subgroup analyses for referrals to Children's social care services

N(%) referred	FNP	Usual care	Adjusted[a] OR (95% CI), p-value	Interaction p-value
**Child Gender**				
Boys	99 (26.3)	108 (27.7)	0.93 (0.65 to 1.33), 0.69	0.78
Girls	107 (27.9)	100 (28.1)	1.01 (0.69 to 1.48), 0.94	
**Maternal age at recruitment**				
<16 years	17 (34.7)	19 (34.6)	1.05 (0.44 to 2.46), 0.92	0.96
≥16 years	189 (26.6)	189 (27.4)	0.96 (0.72 to 1.30), 0.81	
**Adaptive functioning**				
No difficulty	140 (25.4)	149 (26.3)	0.99 (0.71 to 1.38), 0.96	0.67
Difficulty in basic skills	66 (31.7)	59 (33.0)	0.81 (0.59 to 1.41), 0.67	
**NEET status**				
EET	70 (20.3)	75 (23.1)	0.83 (0.54 to 1.29), 0.42	0.18
NEET	100 (32.5)	89 (29.2)	1.19 (0.80 to 1.78), 0.39	
**Deprivation quintile**				

For peer review only - http://bmjopen.bmj.com/site/about/guidelines.xhtml

1 – least deprivation	33 (20.4)	34 (23.9)	0.77 (0.44 to 1.36), 0.37	0.32
2	36 (23.8)	30 (20.5)	1.26 (0.61 to 2.60), 0.53	
3	42 (28.8)	43 (27.9)	0.96 (0.50 to 1.86), 0.91	
4	41 (31.1)	39 (25.3)	1.27 (0.69 to 2.31), 0.44	
5 – most deprivation	54 (33.3)	61 (42.4)	0.65 (0.35 to 1.22), 0.18	

[a] FNP compared to usual care. Analysis adjusted for stratification (site), minimisation variables (gestational age, smoking status at recruitment, and first or preferred language). N/EET – Not/In Education, Employment or Training (Source: National Pupil Database, The Department for Education)

Table S14 Subgroup analyses for child in need status

N(%) CIN	FNP	Usual care	Adjusted[a] OR (95% CI), p-value	Interaction p-value
**Gender**				
Boys	80 (21.3)	80 (20.5)	1.05 (0.72 to 1.53), 0.81	0.56
Girls	81 (21.1)	82 (23.0)	0.91 (0.60 to 1.38), 0.65	
**Maternal age at recruitment**				
<16 years	14 (28.6)	16 (29.1)	1.00 (0.41 to 2.43), 0.99	0.98
≥16 years	147 (20.7)	146 (21.1)	0.98 (0.72 to 1.33), 0.88	
**Adaptive functioning**				
No difficulty	106 (19.2)	113 (20.0)	0.99 (0.70 to 1.42), 0.98	0.76
Difficulty in basic skills	55 (26.4)	49 (27.4)	0.93 (0.59 to 1.47), 0.76	
**NEET**				
EET	52 (16.0)	52 (15.1)	0.93 (0.57 to 1.53), 0.78	0.42
NEET	81 (26.3)	72 (23.6)	1.18 (0.77 to 1.77), 0.46	
**Deprivation quintile**				
1 – least deprivation	24 (14.8)	22 (15.5)	0.92 (0.49 to 1.73), 0.79	0.62
2	30 (19.9)	22 (15.1)	1.53 (0.68 to 3.47), 0.31	
3	32 (21.9)	36 (23,4)	0.89 (0.52 to 1.54), 0.68	
4	32 (24.2)	34 (22.1)	1.15 (0.64 to 2.06), 0.64	
5 – most deprivation	43 (26.5)	48 (33.3)	0.72 (0.38 to 1.38), 0.32	

[a] FNP compared to usual care. Analysis adjusted for stratification (site), minimisation variables (gestational age, smoking status at recruitment, and first or preferred language). N/EET – Not/In Education, Employment or Training (Source: National Pupil Database, The Department for Education).

For peer review only - http://bmjopen.bmj.com/site/about/guidelines.xhtml

Table S15 Subgroup analyses for Early Years Assessment total point score

Mean (SD) total point score	FNP	Usual care	Adjusted[a] mean difference (95% CI), p-value	Interaction p-value
**Gender**				
Boys	N=369<br>30.57 (7.33)	N=383<br>29.84 (7.56)	0.75 (-0.31 to 1.82), 0.17	0.57
Girls	N=374<br>33.85 (6.79)	N=345<br>33.53 (7.22)	0.34 (-0.67 to 1.35), 0.51	
**Maternal age at recruitment**				
<16 years	N=45<br>32.44 (6.71)	N=52<br>28.91 (7.70)	3.65 (0.76 to 6.55), 0.013	0.046
≥16 years	N= 698<br>32.21 (7.28)	N= 676<br>31.80 (7.58)	0.42 (-0.36 to 1.21), 0.29	
**Adaptive functioning**				
No difficulty	N=540<br>32.99 (7.12)	N=552<br>32.11 (7.65)	0.88 (0.003 to 1.75), 0.049	0.50
Difficulty in basic skills	N=203<br>30.18 (7.20)	N=175<br>29.94 (7.33)	0.34 (-1.11 to 1.79), 0.65	
**NEET status**				
In EET	N=339<br>32.45 (7.12)	N=322<br>32.37 (7.68)	0.15 (-0.99 to 1.28), 0.80	0.95
NEET	N= 304<br>31.72 (7.57)	N=299<br>31.50 (7.43)	0.19 (-1.00 to 1.38), 0.76	
**Index of Multiple Deprivation quintiles**				
1 = least deprived	N=159<br>33.16 (7.44)	N=139<br>31.53 (8.24)	1.72 (-0.07 to 3.50), 0.060	ref
2	N=148<br>32.16 (6.97)	N=144<br>31.59 (7.05)	0.65 (-0.96 to 2.27), 0.43	0.84
3	N=146<br>32.16 (6.79)	N=151<br>32.59 (7.27)	-0.20 (-1.79 to 1.39), 0.80	0.21
4	N=128<br>30.95 (7.53)	N=148<br>31.11 (7.50)	-0.31 (-2.11 to 1.48), 0.73	0.85
5 = most deprived	N=156<br>32.41 (7.46)	N=140<br>31.03 (8.12)	1.45 (-0.33 to 3.22), 0.11	0.74

[a] FNP compared to usual care. Analysis adjusted for stratification (site), minimisation variables (gestational age, smoking status at recruitment, and first or preferred language). N/EET – Not/In Education, Employment or Training (Source: National Pupil Database, The Department for Education).

For peer review only

Table S16 Subgroup analyses for KS1 assessments reaching the expected standard (RES)

(a) Gender

N(%)	Boys		Girls	
	**Not RES**	**RES**	**Not RES**	**RES**
**Reading**				
FNP	152 (41.8)	212 (58.2)	105 (27.9)	271 (72.1)
Usual care	182 (47.3)	203 (52.7)	107 (30.8)	240 (69.2)
Adjusted[a] OR (95% CI), p-value	1.27 (0.95 to 1.69), 0.11		1.16 (0.84 to 1.59), 0.38	
Interaction p-value=0.68				

Adjusted[b] OR (95% CI), p-value	1.30 (0.96 to 1.75), 0.09		1.17 (0.84 to 1.62), 0.36	
Interaction p-value=0.68				
**Maths**				
FNP	149 (40.9)	215 (59.1)	132 (35.1)	244 (64.9)
Usual care	166 (43.1)	219 (56.9)	117 (33.7)	230 (66.3)
Adjusted[a] OR (95% CI), p-value	1.11 (0.83 to 1.49), 0.49		0.95 (0.69 to 1.29), 0.72*	
Interaction p-value=0.46				
Adjusted[b] OR (95% CI), p-value	1.14 (0.84 to 1.54), 0.40		0.94 (0.69 to 1.29), 0.71	
Interaction p-value=0.47				
**Science**				
FNP	120 (33.0)	244 (67.0)	83 (22.1)	293 (77.9)
Usual care	138 (35.8)	247 (64.2)	81 (23.3)	266 (76.7)
Adjusted[a] OR (95% CI), p-value	1.16 (0.86 to 1.58), 0.34		1.08 (0.76 to 1.53), 0.68	
Interaction p-value=0.77				
Adjusted[b] OR (95% CI), p-value	1.18 (0.87 to 1.63), 0.28		1.08 (0.76 to 1.55), 0.66	
Interaction p-value=0.72				

RES = Reaching the expected standard; [a] Analysis adjusted for stratification (site), minimisation variables (gestational age, smoking status at recruitment, and first or preferred language); [b] Analysis adjusted for stratification (site), minimisation variables (gestational age, smoking status at recruitment, and first or preferred language) and month of birth. (Source: National Pupil Database, The Department for Education).

(b) Maternal age at recruitment

N(%)	Maternal age<16 years		Maternal age≥16 years	
	**Not RES**	**RES**	**Not RES**	**RES**
**Reading**				
FNP	21 (44.7)	26 (55.3)	236 (34.1)	457 (65.9)
Usual care	29 (52.7)	26 (47.3)	260 (38.4)	417 (61.6)
Adjusted[a] OR (95% CI), p-value	1.21 (0.97 to 1.52), 0.09		1.49 (0.65 to 3.41), 0.34	
Interaction p-value=p=0.66				
Adjusted[b] OR (95% CI), p-value	1.50 (0.65 to 3.48), 0.34		1.25 (0.99 to 1.57), 0.07	
Interaction p-value=0.62				
**Maths**				
FNP	16 (34.0)	31 (66.0)	265 (38.2)	428 (61.8)
Usual care	32 (58.2)	23 (41.8)	251 (37.1)	426 (62.9)
Adjusted[a] OR (95% CI), p-value	3.15 (0.94 to 10.48), 0.06		0.96 (0.77 to 1.19), 0.69	
Interaction p-value=0.011				

For peer review only - http://bmjopen.bmj.com/site/about/guidelines.xhtml

Adjusted[b] OR (95% CI), p-value	3.23 (1.36 to 7.67), 0.008		0.98 (0.78 to 1.22), 0.83	
Interaction p-value=0.014				
**Science**				
FNP	18 (38.3)	29 (61.7)	185 (26.7)	508 (73.3)
Usual care	25 (45.4)	30 (54.6)	194 (28.7)	483 (71.3)
Adjusted[a] OR (95% CI), p-value	1.54 (0.53 to 4.47), 0.42		1.11 (0.88 to 1.41), 0.39	
Interaction p-value=0.56				
Adjusted[b] OR (95% CI), p-value	1.37 (0.59 to 3.19), 0.467		1.14 (0.89 to 1.45), 0.29	
Interaction p-value=0.69				

RES = Reaching the expected standard; [a] FNP compared to Usual care. Analysis adjusted for stratification (site), minimisation variables (gestational age, smoking status at recruitment, and first or preferred language); [b] Analysis adjusted for stratification (site), minimisation variables (gestational age, smoking status at recruitment, and first or preferred language) and month of birth. (Source: National Pupil Database, The Department for Education).

(c) Adaptive functioning

N(%)	No difficulty with basic skills		Difficulty with basic skills	
	**Not RES**	**RES**	**Not RES**	**RES**
**Reading**				
FNP	152 (30.5)	347 (69.5)	87 (42.9)	116 (57.1)
Usual care	188 (36.2)	332 (63.8)	84 (48.0)	91 (52.0)
Adjusted[a] OR (95% CI), p-value	1.29 (0.98 to 1.70), 0.07		1.23 (0.82 to 1.86), 0.32	
Interaction p-value=0.61				
Adjusted[b] OR (95% CI), p-value	1.29 (0.98 to 1.69), 0.07		1.25 (0.83 to 1.91), 0.29	
Interaction p-value=0.95				
**Maths**				
FNP	186 (34.6)	351 (65.4)	95 (46.8)	108 (53.2)
Usual care	192 (34.5)	364 (65.5)	91 (52.0)	84 (48.0)
Adjusted[a] OR (95% CI), p-value	1.01 (0.78 to 1.29), 0.97		1.27 (0.84 to 1.93), 0.26	
Interaction p-value=0.38				
Adjusted[b] OR (95% CI), p-value	1.01 (0.78 to 1.31), 0.93		1.32 (0.86 to 2.03), 0.20	
Interaction p-value=0.28				
**Science**				
FNP	130 (24.2)	407 (75.8)	73 (36.0)	130 (64.0)
Usual care	153 (27.5)	403 (72.5)	66 (37.7)	109 (62.3)
Adjusted[a] OR (95% CI), p-value	1.20 (0.92 to 1.58), 0.19		1.10 (0.71 to 1.69), 0.68	
Interaction p-value=0.71				

For peer review only - http://bmjopen.bmj.com/site/about/guidelines.xhtml

Adjusted[b] OR (95% CI), p-value	1.22 (0.93 to 1.62), 0.16	1.13 (0.73 to 1.74), 0.59
Interaction p-value=0.82		

RES = Reaching the expected standard; [a] FNP compared to Usual care. Analysis adjusted for stratification (site), minimisation variables (gestational age, smoking status at recruitment, and first or preferred language); [b] Analysis adjusted for stratification (site), minimisation variables (gestational age, smoking status at recruitment, and first or preferred language) and month of birth. (Source: National Pupil Database, The Department for Education).

(d) Not in Education, employment or training (NEET) status

N(%)	NEET		In EET	
	**Not RES**	**RES**	**Not RES**	**RES**
**Reading**				
FNP	105 (31.2)	232 (68.8)	109 (36.0)	194 (64.0)
Usual care	115 (35.8)	206 (61.2)	121 (40.5)	178 (59.5)
Adjusted[a] OR (95% CI), p-value	1.26 (0.91 to 1.75), 0.17		1.21 (0.87 to 1.69), 0.26	
Interaction p-value=0.86				
Adjusted[b] OR (95% CI), p-value	1.31 (0.94 to 1.83), 0.17		1.25 (0.89 to 1.76), 0.19	
Interaction p-value=0.83				
**Maths**				
FNP	122 (36.2)	215 (63.8)	122 (40.3)	181 (59.7)
Usual care	107 (33.3)	214 (66.7)	120 (40.1)	179 (59.9)
Adjusted[a] OR (95% CI), p-value	0.89 (0.65 to 1.23), 0.48		0.99 (0.72 to 1.38), 0.97	
Interaction p-value=0.64				
Adjusted[b] OR (95% CI), p-value	0.93 (0.66 to 1.29), 0.65		1.03 (0.73 to 1.42), 0.90	
Interaction p-value=0.68				
**Science**				
FNP	87 (25.8)	250 (74.2)	821 (27.1)	221 (72.9)
Usual care	78 (24.3)	243 (75.7)	98 (32.8)	201 (67.2)
Adjusted[a] OR (95% CI), p-value	0.93 (0.65 to 1.33), 0.69		1.31 (0.92 to 1.87), 0.14	
Interaction p-value=0.20				
Adjusted[b] OR (95% CI), p-value	0.96 (0.67 to 1.39), 0.84		1.36 (0.95 to 1.95), 0.10	
Interaction p-value=0.22				

RES = Reaching the expected standard; [a] FNP compared to Usual care. Analysis adjusted for stratification (site), minimisation variables (gestational age, smoking status at recruitment, and first or preferred language); [b] Analysis adjusted for stratification (site), minimisation variables (gestational age, smoking status at recruitment, and first or preferred language) and month of birth. N/EET – Not/In Education, Employment or Training (Source: National Pupil Database, The Department for Education).

For peer review only - http://bmjopen.bmj.com/site/about/guidelines.xhtml

(e) Index of Multiple Deprivation quintiles

	N(%)	Q1=least deprived		Q2		Q3		Q4		Q5=most deprived	
		Not RES	RES	Not RES	RES	Not RES	RES	Not RES	RES	Not RES	RES
Reading	FNP	49 (30.3)	113 (67.7)	53 (36.3)	93 (63.7)	49 (33.6)	97 (66.4)	48 (37.2)	81 (62.8)	57 (37.8)	94 (62.2)
	Usual care	54 (38.9)	85 (61.1)	64 (44.1)	81 (55.9)	48 (31.8)	103 (68.2)	64 (43.0)	85 (57.0)	56 (39.4)	86 (60.6)
	Adjusted[a] OR (95% CI), p-value	1.57 (0.88 to 2.77), 0.12		1.42 (0.88 to 2.29), 0.16		0.96 (0.59 to 1.57), 0.88		1.33 (0.81 to 2.18), 0.25		1.07 (0.60 to 1.89), 0.83	
	Adjusted[b] OR (95% CI), p-value	1.75 (1.00 to 3.07), 0.05		1.42 (0.85 to 2.37), 0.18		0.89 (0.53 to 1.48), 0.64		1.41 (0.86 to 2.34), 0.18		1.00 (0.57 to 1.75), 0.99	
	Interaction p-value=0.70										
	Interaction p-value=0.37										
Maths	FNP	56 (34.6)	106 (65.4)	57 (39.0)	89 (61.0)	50 (34.3)	96 (65.7)	55 (42.6)	74 (57.4)	62 (41.1)	89 (58.9)
	Usual care	52 (37.4)	87 (62.6)	60 (41.4)	85 (58.6)	56 (37.1)	95 (62.9)	60 (40.3)	89 (59.7)	54 (38.0)	88 (62.0)
	Adjusted[a] OR (95% CI), p-value	1.15 (0.72 to 1.85), 0.56		1.12 (0.69 to 1.82), 0.65		1.17 (0.69 to 1.97), 0.56		0.93 (0.57 to 1.52), 0.77		0.91 (0.56 to 1.47), 0.69	
	Adjusted[b] OR (95% CI), p-value	1.36 (0.82 to 2.24), 0.23		1.12 (0.68 to 1.82), 0.66		1.11 (0.65 to 1.90), 0.69		0.98 (0.60 to 1.62), 0.95		0.88 (0.54 to 1.44), 0.62	
	Interaction p-value=0.91										
	Interaction p-value=0.81										

For peer review only - http://bmjopen.bmj.com/site/about/guidelines.xhtml

	N(%)	Q1=least deprived		Q2		Q3		Q4		Q5=most deprived	
		Not RES	RES	Not RES	RES	Not RES	RES	Not RES	RES	Not RES	RES
Science	FNP	37 (22.8)	125 (77.2)	36 (24.7)	110 (75.3)	40 (27.4)	106 (72.6)	45 (34.9)	84 (65.1)	44 (29.1)	107 (70.9)
	Usual care	45 (32.4)	94 (67.6)	45 (31.0)	100 (69.0)	35 (23.2)	116 (76.8)	47 (31.5)	102 (68.5)	46 (32.4)	96 (67.6)
	Adjusted[a] OR (95% CI), p-value	1.68 (1.00 to 2.81), 0.048		1.39 (0.80 to 2.41), 0.25		0.82 (0.46 to 1.47), 0.51		0.89 (0.52 to 1.51), 0.65		1.24 (0.74 to 2.06), 0.42	
	Adjusted[b] OR (95% CI), p-value	1.94 (1.13 to 3.30), 0.015		1.40 (0.76 to 2.57), 0.28		0.78 (0.43 to 1.40), 0.41		0.92 (0.55 to 1.55), 0.76		1.16 (0.69 to 1.96), 0.57	
	Interaction p-value=0.25										
	Interaction p-value=0.11										

RES = Reaching the expected standard; a FNP compared to Usual care; [a] Analysis adjusted for stratification (site), minimisation variables (gestational age, smoking status at recruitment, and first or preferred language); [b] Analysis adjusted for stratification (site), minimisation variables (gestational age, smoking status at recruitment, and first or preferred language) and month of birth. (Source: National Pupil Database, The Department for Education).

For peer review only - http://bmjopen.bmj.com/site/about/guidelines.xhtml

Table S17 Subgroup analyses for reaching the expected standard in the writing KS1 assessment for 2016/17 academic year

N(%)	FNP	Usual care	Adjusted[a] OR (95% CI), p-value	Interaction p-value	Adjusted[b] OR (95% CI), p-value	Interaction p-value
**Gender**						
Boys	109 (42.9)	83 (31.8)	1.61 (1.13 to 2.32), 0.009	0.034	1.62 (1.13 to 2.33), 0.009	0.039
Girls	132 (54.1)	126 (55.8)	1.03 (0.76 to 1.39), 0.87		0.94 (0.65 to 1.37), 0.76	
**Maternal age at recruitment**						
<16 years	18 (54.6)	9 (22.5)	4.67 (1.47 to 14.84), 0.009	0.016	5.28 (1.49 to 18.73), 0.010	0.018
≥16 years	223 (48.0)	200 (44.7)	1.13 (0.87 to 1.47), 0.35		1.15 (0.89 to 1.50), 0.29	
**Adaptive functioning**						
No difficulty	189 (52.5)	174 (46.7)	1.26 (0.93 to 1.70), 0.14	0.72	1.26 (0.93 to 1.70), 0.14	0.67
Difficulty in basic skills	52 (37.7)	34 (30.1)	1.41 (0.77 to 2.58), 0.26		1.53 (0.81 to 2.90), 0.19	
**NEET**						
EET (no not EET)	109 (47.8)	119 (52.2)	0.82 (0.56 to 1.19), 0.29	0.019	0.82 (0.56 to 1.19), 0.29	0.028
NEET	105 (49.1)	77 (38.3)	1.56 (1.05 to 2.30), 0.027		1.56 (1.05 to 2.30), 0.027	
**Deprivation quintile**						
1 – least deprived	63 (53.9)	39 (42.9)	1.65 (0.90 to 3.05), 0.11	0.89	1.83 (0.95 to 3.51), 0.07	0.86
2	48 (46.2)	46 (44.7)	1.00 (0.52 to 1.95), 0.99		1.04 (0.53 to 2.03), 0.92	
3	46 (50.6)	49 (46.7)	1.20 (0.67 to 2.15), 0.53		1.22 (0.66 to 2.26), 0.53	
4	40 (45.5)	35 (38.5)	1.28 (0.70 to 2.35), 0.42		1.28 (0.69 to 2.35), 0.43	
5 – most deprived	42 (44.2)	39 (41.1)	1.16 (0.64 to 2.08), 0.629		1.17 (0.64 to 2.14), 0.602	

For peer review only - http://bmjopen.bmj.com/site/about/guidelines.xhtml

[a] FNP compared to usual care. Analysis adjusted for stratification (site), minimisation variables (gestational age, smoking status at recruitment, and first or preferred language); [b] Analysis additionally adjusted for month of birth. N/EET – Not/In Education, Employment or Training (Source: National Pupil Database, The Department for Education).

For peer review only

Domestic abuse and CIN status:

Domestic abuse experienced by the mother was assessed using the Composite Abuse Scale (CAS) in the BB:0-2 trial at 24 months follow-up (with a score 1 or greater indicating some sort of abuse). The CAS could only be administered if the interview was face-to-face (as opposed to by telephone) and only if the participant was alone (N=607). In the BB:2-6 sample, this could be examined in 595 participants (FNP:322, Usual Care:273) and the proportion of participants experiencing some form of abuse was comparable to that found in the BB:0-2 trial (FNP: 122 (27.9%) vs UC:113 (41.4%)). There was also a relationship found between CAS and CIN status with 49 (20.9%) participants who experienced some abuse having a child being in need versus 42 (11.8%) of those who did not experience abuse. Domestic abuse at 24 months was to be explored as a possible effect mediator but due to lack of intervention effect in the child's CIN status, the need to examine the proportion of effect mediated by experience of domestic abuse at 24 months was redundant.

Care-experienced mothers and CIN status:

In addition, care-experienced mothers at time of recruitment to the BB:0-2 trial was examined. This was defined from the CLA dataset. Sixty-one mothers were identified as having experienced a period of care, four of whom were identified as care-experienced only after their BB:0-2 recruitment date. Fifty-seven mothers were examined for their duration of care and the women randomised to receive FNP experienced on average nine months more care than the Usual care arm (median (25th to 75th centile) FNP: 40 (15.5 to 113.0) UC: 31 (7.0 to 69.5) months). After merging these care-experienced mothers into the children's dataset, 56 mothers remained; seven mothers were excluded from the subgroup analyses since their child did not have any NPD data. Children of these care-experienced mothers were at a higher risk of being defined as in need themselves but no interaction was observed between trial arm and mothers care status. The duration of the maternal care status as a possible mediator effect was also stated in the statistical analysis plan but similarly to domestic abuse, due to lack of intervention effect in child's CIN status, then the need to examine the proportion of effect mediated by length of mother's care experience was redundant.

VIII. Treatment efficacy

The analyses of the following outcomes were re-run to investigate the effect of FNP dosage (number of visits over all delivery phases) using complier averaged causal effects modelling by fitting a structural mean model. The programme stated maximum number of visits over the whole period of FNP is 64 (14 in the pregnancy phase, 28 in the infancy phase, 22 in the toddler phase).

For peer review only - http://bmjopen.bmj.com/site/about/guidelines.xhtml

Referrals to CSC services: Adjusting the analysis for receipt of full dosage of FNP visits, made no difference to the conclusion of the main analysis for referral to social services (efficacy per visit odds ratio (OR)=1.00, 95% CI=0.99 to 1.004, p=0.613).

CIN status: Adjusting the analysis for receipt of FNP made no difference to the conclusion of the primary outcome (efficacy per visit OR=1.00, 95% CI=0.99 to 1.004, p=0.613).

Early Years assessment total point score: Adjusting the analysis for receipt of FNP we observe a 0.02 increase in point score in the between-arm mean difference in early years total score (as the number of visits increase so does the score) however the confidence interval around this estimate is wide (-0.004 to 0.040). The estimated treatment efficacy for participants receiving all 64 visits was 1.15 (95% CI -0.26 to 2.56), p=0.106 showing no change to the overall conclusions compared to the primary analysis.

KS1 attainment: Adjusting the analysis for FNP receipt we observe an increase in the odds of an event occurring in the FNP arm compared UC arm for every unit increase in FNP visit received. For all areas of learning (reading, writing, maths and science), adjusting the analysis for FNP receipt strengthens the effect of FNP, but it is the reading and writing assessment where given the full dosage of 64 FNP visits the odds make a significant difference. For reading assessments, the odds of reaching the expected standard increases from OR=1.23 (95% CI=0.99 to 1.53), p=0.051 in the main analysis to 1.38 (1.07 to 1.77), p=0.015. The efficacy per visit for reading was 1.005 (1.001 to 1.009), p=0.015; maths: 1.001 (0.997 to 1.005), p=0.535; science: 1.004 (0.999 to 1.008), p=0.112; writing: 1.013 (1.09 to 1.017), p=0.001.

For peer review only

IX. Exploration of surveillance bias upon Child In Need outcome assessment

Table S18 Baseline maternal characteristics, school readiness outcomes for children referred to CSC and classed as a Child In Need, before the age of 4 years old by intervention arm.

	**FNP N=60**	**Usual Care N=62**	**OR (95% CI)**
Maternal age (years) at recruitment *Median (25th to 75th centiles)*	17.7 (16.9 to 18.3)	17.8 (16.7 to 18.7)	0.97 (0.73 to 1.27)
Not in Education, Employment or Training at recruitment *N(%)*			
Education, Employment or Training	21 (42.0)	11 (22.0)	
Not in Education, Employment or Training	30 (58.0)	39 (78.0)	2.45 (1.04 to 5.93)
Not applicable (<16 years at interview)/ missing	9	12	

For peer review only - http://bmjopen.bmj.com/site/about/guidelines.xhtml

Ethnicity *N(%)*	Numbers too small		
Deprivation score[1] *Median (25th to 75th centiles)*	43.0 (25.0 to 56.1)	45.0 (31.8 to 62.7)	0.99 (0.98 to 1.01)
Adaptive functioning			
Had 3 or less life skills			
No	38 (63.3)	43 (69.4)	
Yes	22 (36.7)	19 (30.6)	1.41 (0.67 to 2.98)
Difficulty in at least one basic skills *N(%)*			
No	37 (61.7)	43 (69.4)	
Yes	23 (38.3)	19 (30.6)	1.31 (0.62 to 2.8)
At least one burden *N(%)*			
No	39 (65.0)	42 (68.9)	
Yes	21 (35.0)	19 (31.1)	1.19 (0.56 to 2.54)
Social support			
Maximum social support N(%) (score=100)	10 (16.9)	9 (14.5)	1.20 (0.45 to 3.21)
Relationship quality *(score 7 to 35)[2]*	28 (25 to 30)	30 (25 to 32)	0.97 (0.89 to 1.05)
Self-efficacy *(score 10 to 40)[3]* *Median (25th to 75th centiles)*	30 (28 to 32)	30 (27.25 to 31)	1.05 (0.97 to 1.15)
Family resource *(score 4 to 20)[4]* *Mean (SD)*	12.6 (4.2)	12.7 (3.9)	0.91 (0.64 to 1.30)
CRAFFT *(score 0 to 6)[5]* *Median (25th to 75th centiles)*	1 (0 to 2)	1 (0 to 2)	1.07 (0.85 to 1.35)
**Early Years**			
GLD in all 5 areas of learning *N(%)*	26 (44.1)	22 (37.9)	1.29 (0.62 to 2.70)
GLD in all 17 early learning goals *N(%)*	26 (44.1)	20 (34.5)	1.50 (0.71 to 3.16)
Total point score over all 17 early learning goals *Median (25th to 75th centiles)*	32 (22 to 34)	30.5 (21 to 34)	Mean difference = 1.66 (-1.14 to 4.45)

[1] Higher score indicates more deprived, [2] Higher score indicates a better relationship quality, [3] Higher score indicates higher level of self-efficacy, [4] Higher score indicates higher level of family resource, [5] Greater risk of problems related to drugs and alcohol

For peer review only

X. Missing data

Since only a small number (n=40 (2.7%)) of children were not linked to any NPD dataset and not included in the primary outcome analyses, no multiple imputation was carried out.

XI. Cost Consequence Analysis

Table S19 Cost-consequence balance sheet for the family-nurse based intervention for the four years between children's ages 2 and 6.

**Mothers**						
	Mean resource use per participant			Mean cost per participant (£)		
	FNP	Usual Care	Incremental (FNP-Usual Care)	FNP	Usual Care	Incremental (FNP-Usual Care)
**Health related resource use**						
Inpatient Attendances						
Overnight admissions (N)	3.04	2.99	0.05			
Overnight length of stay (N nights)	2.32	2.32	0.00	2,826.79	2,844.58	-17.79
Day Admittances	2.93	2.91	0.02	1,874.61	1,890.11	-15.50
Outpatient Attendances						
All Outpatient Attendances	23.29	22.61	0.68	2,244.62	2,189.32	55.30
Accident and Emergency						
A&E Visits (N)	5.19	4.93	0.26	411.34	392.03	19.31
Total Resource Use						
				7,357.36	7,316.04	41.32 p=0.393
**Children**						
	Mean resource use per participant			Mean cost per participant (£)		
	FNP	Usual Care	Incremental (FNP-Usual Care)	FNP	Usual Care	Incremental (FNP-Usual Care)
**Health related resource use**						
Inpatient Attendances - All						
Overnight admissions (N)	1.57	1.55	0.02			
Overnight length of stay (N nights)	3.57	3.39	0.18	1,962.95	2,033.71	-70.76
Day Admittances	0.98	0.97	0.01	744.94	724.90	20.04
Inpatient Attendances – All (Excluding Injuries and Ingestions)						
Overnight admissions (N)	1.51	1.49	0.02	1,883.44	1,951.15	-67.71
Overnight length of stay (N nights)	3.61	3.44	0.17			
Day Admittances	0.90	0.88	0.02	689.22	667.09	22.13

For peer review only - http://bmjopen.bmj.com/site/about/guidelines.xhtml

Inpatient Attendances – Injuries and Ingestions Only						
Overnight admissions (N)	0.06	0.06	0.00			
Overnight length of stay (N nights)	2.50	2.05	0.45	79.51	82.55	-3.04
Day Admittances	0.08	0.09	-0.01	55.72	57.81	-2.09
Outpatient Attendances						
All Outpatient Attendances	6.89	7.44	-0.55	673.24	727.79	-54.55
Accident and Emergency - All						
A&E Visits (N)	4.93	4.62	0.31	395.73	366.83	28.90
Accident and Emergency - All (Excluding Injuries and Ingestions)						
A&E Visits (N)	3.80	3.52	0.28	305.12	280.20	24.92
Accident and Emergency – Injuries and Ingestions Only						
A&E Visits (N)	1.12	1.10	0.02	75.09	69.91	5.18
Total Resource Use						
				3,778.13	3,853.22	-75.09 p=0.865
**Consequences**						
Child in Need status recorded at any time during the follow-up period.						OR: 0.98 (95%CI: 0.74 to 1.31) p=0.902
Referral to Social Services						OR: 0.97 (0.74 to 1.28) p=0.829
Child Protection registration (CPP)						OR: 1.04 (0.69 to 1.57) p=0.846
Details of a CPP						
• Neglect						Reference
• Emotional						OR: 2.77 (1.02 to 7.56) p=0.046
• Physical						OR: 1.25 (0.32 to 4.88) p=0.743
• Sexual and multiple						OR: 1.13 (0.33 to 3.86) p=0.848
CIN categorisation						
• Abuse or Neglect						Reference
• Family dysfunction						OR: 0.95 (0.54 to 1.69) p=0.868
• Family in acute stress						OR: 1.47 (0.61 to 3.54) p=0.389
• Low income, absent parenting, socially unacceptable behaviour						OR: 2.38 (0.86 to 6.57) p=0.093
• Child/parent disability						OR: 1.47 (0.45 to 4.84) p=0.525
Looked after status						OR: 0.90 (0.52 to 1.57) p=0.712
Child looked after period of care (months)						OR: 0.75 (0.65 to 0.86) p<0.001
DNA appointments						
• DNA at least once						OR: 1.00 (0.79 to 1.26) p=0.997
Injuries and ingestions						
• Emergency attendance						
○ No						Reference

For peer review only

For peer review only - http://bmjopen.bmj.com/site/about/guidelines.xhtml

○ Yes	OR: 1.17 (0.95 to 1.45) p=0.149
• Admission to hospital	
○ No	Reference
○ Yes	OR: 0.87 (0.63 to 1.20) p=0.407
• Either hospital attendance and/or admission to hospital	
○ No	Reference
○ Yes	OR: 1.11 (0.89 to 1.37) p=0.351
Special Educational Needs	OR: 0.83 (0.67 to 1.03) p=0.097
Early Years assessment	
• Achieving good level of development in all five areas of learning	OR: 1.26 (1.03 to 1.55) p=0.026
• Achieving good level of development in all 17 early learning goals	OR: 1.24 (1.01 to 1.52) p=0.043
School attendance	
• Overall absences	
○ No absences	Reference
○ At least one absence	OR: 1.00 (0.47 to 2.12) p=0.998
• Overall unauthorised absences	
○ No absences	Reference
○ At least one absence	OR: 0.95 (0.76 to 1.18) p=0.620
• Overall authorised absences	
○ No absences	Reference
○ At least one absence	OR: 1.01 (0.58 to 1.75) p=0.984
KS1 attainment	
• Reading	
○ Lower than expected	Reference
○ Reaching the expected standard	OR: 1.23 (0.99 to 1.53) p=0.051
• Writing academic year 2016/17	
○ Lower than expected	Reference
○ Reaching the expected standard	OR: 1.24 (0.97 to 1.60) p=0.090
• Maths	
○ Lower than expected	Reference
○ Reaching the expected standard	OR: 1.04 (0.84 to 1.28) p=0.731
• Science	
○ Lower than expected	Reference
○ Reaching the expected standard	OR: 1.14 (0.91 to 1.43) p=0.254

For peer review only

The RECORD statement – checklist of items, extended from the STROBE statement, that should be reported in observational studies using routinely collected health data.

	Item No.	STROBE items	Location in manuscript where items are reported	RECORD items	Location in manuscript where items are reported
**Title and abstract**					
	1	(a) Indicate the study's design with a commonly used term in the title or the abstract (b) Provide in the abstract an informative and balanced summary of what was done and what was found	1a/b Abstract	RECORD 1.1: The type of data used should be specified in the title or abstract. When possible, the name of the databases used should be included.<br><br>RECORD 1.2: If applicable, the geographic region and timeframe within which the study took place should be reported in the title or abstract.<br><br>RECORD 1.3: If linkage between databases was conducted for the study, this should be clearly stated in the title or abstract.	1.1 Abstract<br>1.2 Abstract<br>1.3 Abstract
**Introduction**					
Background rationale	2	Explain the scientific background and rationale for the investigation being reported	Introduction		
Objectives	3	State specific objectives, including any prespecified hypotheses	Introduction > Objectives		
**Methods**					
Study Design	4	Present key elements of study design early in the paper	Methods> Study design, setting and participants		
Setting	5	Describe the setting, locations, and relevant dates, including periods of recruitment, exposure, follow-up, and data collection	Methods>Procedure and data sources		

For peer review only

Participants	6	*(a) Cohort study* - Give the eligibility criteria, and the sources and methods of selection of participants. Describe methods of follow-up<br>*Case-control study* - Give the eligibility criteria, and the sources and methods of case ascertainment and control selection. Give the rationale for the choice of cases and controls<br>*Cross-sectional study* - Give the eligibility criteria, and the sources and methods of selection of participants<br><br>*(b) Cohort study* - For matched studies, give matching criteria and number of exposed and unexposed<br>*Case-control study* - For matched studies, give matching criteria and the number of controls per case	6a Methods> Study design, setting and participants<br><br>6b NA	RECORD 6.1: The methods of study population selection (such as codes or algorithms used to identify subjects) should be listed in detail. If this is not possible, an explanation should be provided.<br><br>RECORD 6.2: Any validation studies of the codes or algorithms used to select the population should be referenced. If validation was conducted for this study and not published elsewhere, detailed methods and results should be provided.<br><br>RECORD 6.3: If the study involved linkage of databases, consider use of a flow diagram or other graphical display to demonstrate the data linkage process, including the number of individuals with linked data at each stage.	6.1 Methods> Study design, setting and participants<br><br>6.2 NA<br><br>6.3 Protocol paper cited, Figure 1
Variables	7	Clearly define all outcomes, exposures, predictors, potential confounders, and effect modifiers. Give diagnostic criteria, if applicable.	Methods> Study design, setting and participants, Outcomes; Appendix	RECORD 7.1: A complete list of codes and algorithms used to classify exposures, outcomes, confounders, and effect modifiers should be provided. If these cannot be reported, an explanation should be provided.	Methods> Study design, setting and participants, Outcomes
Data sources/ measurement	8	For each variable of interest, give sources of data and details of methods of assessment (measurement).<br>Describe comparability of assessment methods if there is more than one group	Methods> Study design, setting and participants, Outcomes; Appendix		

For peer review only

Bias	9	Describe any efforts to address potential sources of bias	Methods> Efforts to address sources of bias		
Study size	10	Explain how the study size was arrived at	Methods> Power calculation		
Quantitative variables	11	Explain how quantitative variables were handled in the analyses. If applicable, describe which groupings were chosen, and why	Methods>Statistical analysis		
Statistical methods	12	(a) Describe all statistical methods, including those used to control for confounding<br>(b) Describe any methods used to examine subgroups and interactions<br>(c) Explain how missing data were addressed<br>(d) *Cohort study* - If applicable, explain how loss to follow-up was addressed<br>*Case-control study* - If applicable, explain how matching of cases and controls was addressed<br>*Cross-sectional study* - If applicable, describe analytical methods taking account of sampling strategy<br>(e) Describe any sensitivity analyses	12a-c, e: Methods>Statistical analysis; Economic analysis<br>12d: NA		
Data access and cleaning methods		..		RECORD 12.1: Authors should describe the extent to which the investigators had access to the database population used to create the study population.	Methods> Study design, setting and participants, Outcomes; Appendix

For peer review only

				RECORD 12.2: Authors should provide information on the data cleaning methods used in the study.	
Linkage		..		RECORD 12.3: State whether the study included person-level, institutional-level, or other data linkage across two or more databases. The methods of linkage and methods of linkage quality evaluation should be provided.	Methods> Procedure and data sources
**Results**					
Participants	13	(a) Report the numbers of individuals at each stage of the study (*e.g.*, numbers potentially eligible, examined for eligibility, confirmed eligible, included in the study, completing follow-up, and analysed)<br>(b) Give reasons for non-participation at each stage.<br>(c) Consider use of a flow diagram	Table S3, Figure 1	RECORD 13.1: Describe in detail the selection of the persons included in the study (*i.e.,* study population selection) including filtering based on data quality, data availability and linkage. The selection of included persons can be described in the text and/or by means of the study flow diagram.	Figure 1
Descriptive data	14	(a) Give characteristics of study participants (*e.g.*, demographic, clinical, social) and information on exposures and potential confounders<br>(b) Indicate the number of participants with missing data for each variable of interest<br>(c) *Cohort study* - summarise follow-up time (*e.g.*, average and total amount)	14a: Table 1<br>14b: Table S3, Tables 2-4		
Outcome data	15	*Cohort study* - Report numbers of outcome events or summary measures over time<br>*Case-control study* - Report numbers in each exposure	Tables 2-4		

For peer review only

		category, or summary measures of exposure<br>*Cross-sectional study* - Report numbers of outcome events or summary measures			
Main results	16	(a) Give unadjusted estimates and, if applicable, confounder-adjusted estimates and their precision (e.g., 95% confidence interval). Make clear which confounders were adjusted for and why they were included<br>(b) Report category boundaries when continuous variables were categorized<br>(c) If relevant, consider translating estimates of relative risk into absolute risk for a meaningful time period	Tables 2-4		
Other analyses	17	Report other analyses done—e.g., analyses of subgroups and interactions, and sensitivity analyses	Appendix		
**Discussion**					
Key results	18	Summarise key results with reference to study objectives	Discussion> Principal findings		
Limitations	19	Discuss limitations of the study, taking into account sources of potential bias or imprecision. Discuss both direction and magnitude of any potential bias		RECORD 19.1: Discuss the implications of using data that were not created or collected to answer the specific research question(s). Include discussion of misclassification bias, unmeasured confounding, missing data, and changing eligibility over time, as they pertain to the study being reported.	Discussion> Strengths and weaknesses of this study, Strengths and weaknesses in comparison to other studies
Interpretation	20	Give a cautious overall interpretation of results considering objectives,			Discussion> Strengths and weaknesses of this study, Strengths

For peer review only

		limitations, multiplicity of analyses, results from similar studies, and other relevant evidence			and weaknesses in comparison to other studies
Generalisability	21	Discuss the generalisability (external validity) of the study results			Discussion> Strengths and weaknesses in comparison to other studies
**Other Information**					
Funding	22	Give the source of funding and the role of the funders for the present study and, if applicable, for the original study on which the present article is based			Described in Funding section
Accessibility of protocol, raw data, and programming code		..		RECORD 22.1: Authors should provide information on how to access any supplemental information such as the study protocol, raw data, or programming code.	Protocol paper cited in references; access to data described in Data sharing statement

*Reference: Benchimol EI, Smeeth L, Guttmann A, Harron K, Moher D, Petersen I, Sørensen HT, von Elm E, Langan SM, the RECORD Working Committee. The REporting of studies Conducted using Observational Routinely-collected health Data (RECORD) Statement. *PLoS Medicine* 2015; in press.

*Checklist is protected under Creative Commons Attribution (CC BY) license.

For peer review only

BMJ Open

A nurse-led home-visitation programme for first-time mothers in reducing maltreatment and improving child health and development (BB:2-6): longer-term outcomes from a randomised cohort using data-linkage.

Journal:	*BMJ Open*
Manuscript ID	bmjopen-2021-049960.R4
Article Type:	Original research
Date Submitted by the Author:	14-Jan-2022
Complete List of Authors:	Robling, Michael; Cardiff University, Centre for Trials Research; DECIPHer, Cardiff University School of Social Sciences<br>Lugg-Widger, Fiona; Cardiff University, Centre for Trials Research<br>Cannings-John, Rebecca; Cardiff University Centre for Trials Research,<br>Angel, Lianna; Cardiff University, Centre for Trials Research<br>Channon, Sue; Cardiff University, Centre for Trials Research<br>Fitzsimmons, Deborah; Swansea University, College of Human and Health Sciences<br>Hood, Kerenza; Cardiff University,<br>Kenkre, Joyce; University of South Wales, Faculty of Life Sciences and Education<br>Moody, Gwenllian; Cardiff University, Centre for Trials Research<br>Owen-Jones, Eleri; Cardiff University, Centre for Trials Research<br>Pockett, Rhys; Swansea University, Swansea Centre for Health Economics<br>Sanders, Julia; Cardiff University, School of Healthcare Sciences<br>Segrott, Jeremy; Cardiff University, Centre for Trials Research; Cardiff University, DECIPHer Centre, Cardiff School of Social Sciences<br>Slater, Thomas; Cardiff University, School of Social Sciences
**Primary Subject Heading**:	Public health
Secondary Subject Heading:	Nursing
Keywords:	PUBLIC HEALTH, Child protection < PAEDIATRICS, PREVENTIVE MEDICINE

SCHOLARONE™
Manuscripts

I, the Submitting Author has the right to grant and does grant on behalf of all authors of the Work (as defined in the below author licence), an exclusive licence and/or a non-exclusive licence for contributions from authors who are: i) UK Crown employees; ii) where BMJ has agreed a CC-BY licence shall apply, and/or iii) in accordance with the terms applicable for US Federal Government officers or employees acting as part of their official duties; on a worldwide, perpetual, irrevocable, royalty-free basis to BMJ Publishing Group Ltd ("BMJ") its licensees and where the relevant Journal is co-owned by BMJ to the co-owners of the Journal, to publish the Work in this journal and any other BMJ products and to exploit all rights, as set out in our licence.

The Submitting Author accepts and understands that any supply made under these terms is made by BMJ to the Submitting Author unless you are acting as an employee on behalf of your employer or a postgraduate student of an affiliated institution which is paying any applicable article publishing charge ("APC") for Open Access articles. Where the Submitting Author wishes to make the Work available on an Open Access basis (and intends to pay the relevant APC), the terms of reuse of such Open Access shall be governed by a Creative Commons licence – details of these licences and which Creative Commons licence will apply to this Work are set out in our licence referred to above.

Other than as permitted in any relevant BMJ Author's Self Archiving Policies, I confirm this Work has not been accepted for publication elsewhere, is not being considered for publication elsewhere and does not duplicate material already published. I confirm all authors consent to publication of this Work and authorise the granting of this licence.

For peer review only

Title: A nurse-led home-visitation programme for first-time mothers in reducing maltreatment and improving child health and development (BB:2-6): longer-term outcomes from a randomised cohort using data-linkage

Authorship:

Michael Robling, [1,2] (Director of Population Health Trials), RoblingMR@cardiff.ac.uk
Fiona Lugg-Widger, [1] (Research Fellow), LuggFV@cardiff.ac.uk
Rebecca Cannings-John, [1] (Senior Research Fellow), CanningsRL@cardiff.ac.uk
Lianna Angel, [2] (Research Associate), AngelL@cardiff.ac.uk
Susan Channon, [1] (Director Research Design and Conduct Service) ChannonS2@cardiff.ac.uk
Deborah Fitzsimmons, [3] (Personal Chair, Public Health, Policy and Social Sciences) d.fitzsimmons@swansea.ac.uk
Kerenza Hood, [1] (Director of the Centre for Trials Research) HoodK1@cardiff.ac.uk
Joyce Kenkre, [4] (Professor of Primary Care) joyce.kenkre@southwales.ac.uk
Gwenllian Moody, [1] (Research Associate) MoodyG@cardiff.ac.uk
Eleri Owen-Jones, [1] (Research Associate) Owen-JonesCE@cardiff.ac.uk
Rhys Pockett, [3] (Senior Lecturer) r.d.pockett@swansea.ac.uk
Julia Sanders, [5] (Professor of Clinical Nursing and Midwifery) SandersJ3@cardiff.ac.uk
Jeremy Segrott, [1,2] (Senior lecturer) SegrottJ@cardiff.ac.uk
Thomas Slater, [6] (Senior lecturer) slaterTB1@cardiff.ac.uk

Affiliations:

1. Centre for Trials Research, Cardiff University, Cardiff, UK
2. DECIPHer, Cardiff University, UK
3. Swansea Centre for Health Economics, Swansea University, UK
4. Faculty of Life Sciences and Education, University of South Wales, UK
5. School of Healthcare Sciences, Cardiff University, UK
6. School of Social Sciences, Cardiff University, UK

Corresponding author:

Michael Robling, Director of Population Health Trials, RoblingMR@cardiff.ac.uk, Centre for Trials Research, College of Biomedical & Life Sciences, Cardiff University, 7th Floor, Neuadd Meirionnydd, Heath Park, Cardiff. CF14 4YS

Keywords: Home-visiting, maltreatment, child development, routine data, cohort

For peer review only

Abstract (300 words)

Objectives Measure effectiveness of Family Nurse Partnership (FNP) home-visiting programme in reducing maltreatment and improving maternal health and child health, developmental and educational outcomes; explore effect moderators, mediators; describe costs.

Design Follow-up of BB:0-2 trial cohort (ISRCTN:23019866) up to age 7 years in England using record linkage.

Participants 1,618 mothers aged 19 years or younger and their firstborn child(ren) recruited to BB:0-2 trial at less than 25 weeks gestation and not mandatorily withdrawn from trial or opted out. Intervention families were offered up to a maximum of 64 home visits by specially trained nurses from pregnancy until firstborn child was 2 years old, plus usually provided health and social care support. Comparator was usual care alone.

Outcome measures Primary outcome: state-verified child-in-need status recorded at any time during follow-up. Secondary outcomes: referral to social services, child protection registration (plan), child-in-need categorisation, looked-after status, recorded injuries and ingestions any time during follow-up, early childcare and educational attendance, school readiness and attainment at Key Stage 1 (KS1), healthcare costs.

Results Match rates for 1,547 eligible children (1517 singletons, 15 sets of twins) were 98.3% (NHS Digital) and 97.4% (National Pupil Database). There was no difference between study arms in the proportion of children being registered as in need (adjusted OR=0.98, 95% CI=0.74,1.31), or for any other measure of maltreatment. Children in the FNP arm were more likely to achieve a good level of development at reception age (school readiness) (adjusted OR=1.24, 95% CI= 1.01,1.52). After adjusting for birth month, children in FNP arm were more likely to reach the expected standard in reading at KS1 (adjusted OR=1.26, 95% CI=1.02,1.57). We found no trial arm differences for resource use and costs.

Conclusions FNP did not improve maltreatment or maternal outcomes. There was evidence of small advantages in school readiness and attainment at KS1.

Strengths and Limitations

- We used administrative records and novel data linkage to provide longer-term follow-up for a well characterised trial cohort
- This has maximised cohort retention and minimised response bias
- Prospectively collected trial data on resource use (e.g. participant-reported contact with health visitors) and FNP service records (e.g. nurse home visits) provide an indication of support

For peer review only

received although the former lacks some granularity. We map out how this informs the comparison of novel intervention with progressive universal usual care.

- We used data from a range of sectors including health, social care and education to assess the broad impact of the specialist home visiting programme.
- The nature of data recorded in service records place restrictions on its utility for some analyses.

Manuscript (Word count is 6631)

For peer review only

Introduction

It is a UK policy priority to protect children from maltreatment and to promote their healthy development. The Family Nurse Partnership (FNP) is a preventative home-visiting programme for young women expecting their first child. FNP was developed in the USA as the Nurse Family Partnership (NFP) and subsequently adapted for implementation in England in 2007.

Three US trials have demonstrated programme improvements in prenatal health behaviours and birth outcomes, sensitive childcare, maternal life-course and child functioning.[1–3] A subgroup analysis of poor unmarried teenage mothers in the first US trial (n=54) found verified maltreatment by age 2 years in 19% of 32 control children and 4% of the 22 children in the group in receipt of NFP during both pregnancy and infancy (mean % difference: 0.15, 95% confidence interval (CI) of −0.01 to 0.31).[1] Following up the same trial cohort, adjusted rates of verified state reports of child abuse and neglect perpetrated by mothers were lower in the group visited by nurses in pregnancy and infancy (0.29) when compared to the women in the control group (0.54) by 15 years (p<0.001).[4] Additionally, there was a 56% relative reduction in emergency department encounters for injuries and ingestions during the second year of life. In the Memphis trial of NFP, the number days in hospital following an injury or ingestion in the first two years of life (a possible indicator of maltreatment) was lower for children of mothers visited by nurses in pregnancy and infancy than children of mothers in the control group.[2] For children with state-verified maltreatment reported by age 4, children in the NFP group had fewer risks for harm than the control group between 25 and 50 months of life. A trial of VoorZorg, the Dutch adaptation of NFP, found a reduction in child-protecting agency reports by age three years for families in the home-visiting arm.[5,]

We evaluated short-term FNP programme outcomes to age 2 years in our Building Blocks trial (BB:0-2).[6] We found no difference for four primary outcomes: maternal tobacco use in late pregnancy, birth weight of the baby, proportion of women with a second pregnancy within 24 months postpartum and emergency attendances and hospital admissions for the child within 24 months postpartum. There

For peer review only - http://bmjopen.bmj.com/site/about/guidelines.xhtml

were some differences in favour of FNP for secondary child development outcomes including maternal-reported cognitive function and language at 24 months.[6] As the previous US trials showed benefit for maltreatment outcomes increasingly after age four we sought to establish whether FNP moderates maltreatment outcomes over a medium-term period. Through access to administrative data, we aimed to determine impact across a range of child maltreatment outcomes and key indicators of neglect (e.g. injuries and ingestions). We also sought to assess impacts on other programme relevant developmental and maternal outcomes as well as costs.

Objectives

Primary: to determine the effectiveness of the FNP programme when added to usually provided health and social care in reducing maltreatment, when compared with usually provided health and social care alone. Secondary: to determine programme effectiveness in reducing maltreatment, medium-term programme outcomes such as subsequent pregnancies, school readiness and educational outcomes, the impact of moderators of programme effect and the cost and consequences of the programme.

Methods

Study design, setting and participants

This study followed a cohort of mothers and children who had participated in the BB:0-2 trial for a further five years using administrative data only.[7,8] Data were retrieved from public sector providers and linked to the trial dataset using opt-out consent, following REC approval (14/WA10062) and s251 support (CAG 10-08(b)/2014). Children and mothers were followed up until the child reached Key Stage 1 teacher-based assessment (KS1) by which time most children were seven years old.

Building Blocks: 2-6 (BB:2-6) study participants were women and their first child (or twins, if relevant) who were not mandatorily withdrawn from the BB:0-2 trial; did not electively withdraw and/or did not opt-out of this follow-on study.[6-8] BB:0-2 trial participants were eligible if women were nulliparous, aged 19 years or younger, were recruited at less than 25 weeks' gestation, living within the catchment area of a local FNP team, able to provide consent and speak English. Women expecting multiple births and those with a previous pregnancy ending in miscarriage, stillbirth, or termination were eligible. Women planning to have their child adopted or to move beyond the FNP catchment area for more than 3 months were not eligible. [6-9] Trial participants had been randomly assigned to FNP or usual care, with randomisation stratified by site and minimised by gestation (<16 weeks vs ≥16 weeks), smoking (yes vs no), and preferred language of data collection (English vs non- English) and weighted towards minimising the imbalance in trial groups with probability 0.8.

For peer review only - http://bmjopen.bmj.com/site/about/guidelines.xhtml

Intervention and comparator (BB:0-2 Trial)

The FNP programme provides up to a maximum of 64 home visits from specially trained family nurses during pregnancy until the child is two years old addressing personal and environmental health, life course development, maternal role, family and friends and access to health and social services. For the 697 women randomised and enrolled to FNP in BB:0-2 and then retained in the BB:2-6 cohort, the average number (SD) of valid visits reported by FNs as being received by FNP clients by programme phase was: Pregnancy 9.74 (3.42), Infancy 18.7 (5.97) and Toddlerhood 13.28 (5.46) (Table S1). In the BB:0-2 trial, mean (SD) visit duration varied by delivery phase (Pregnancy: 79.14 mins (13.78), Infancy: 73.17 (11.61), Toddlerhood: 74.75 (13.50) which exceeded the programme target of 60 minutes.[8] This compares to a duration of 75-90 mins (undifferentiated across the three programme delivery phases) reported for the Elmira and Memphis trials.[10] All trial participants received usually provided health and social care services. Participants in the usual care (UC) arm received these services alone.[6,8,11] The number (SD) of midwifery contacts were recorded in the BB:0-2 trial and were for FNP clients 10.68 (5.25) and for women in UC arm 10.69 (5.34).[11] The number (SD) of home visits from specialist public health nurses (SPHN) reported by trial participants by 18 months postpartum was for FNP clients 4.70 (7.81) and for women in the UC arm 5.01 (5.5). The average number (SD) of clinic contacts with SPHNs was 0.70 (2.29) for women in the FNP arm and 6.31 (7.07) for women in the control arm.

Procedure and data sources

The BB:0-2 trial consented 1618 eligible women between June 2009 and July 2010 and we concluded all BB:2-6 follow-up in May 2018. Maternal and child identifiers were sent to NHS Digital and the Department for Education (DfE) for matching with their databases. Maternal identifiers were also sent to the Department of Health and Social Care (DHSC) for matching with abortion statistics. Matching with NHS Digital and DHSC used a combination of NHS number, date of birth, postcode and sex. NHS Digital also provided a linked Hospital Episode Statistics (HES) - Office for National Statistics (ONS) extract for mortality data. The process of matching for DfE records involved exact matching on first name and surname, date of birth and postcode. For social care data this was for both mother and child. For all other DfE datasets, this was just for the child. All matched data were sent to a third-party data safe haven (Secure Anonymised Information Linkage (SAIL) Databank) and linked by project identifiers to trial data and analysed via remote access.[12-14]

Outcomes

The primary outcome was Child in Need (CIN) status recorded at any time by a local department of children's social care services (CSCS) (and sourced from the NPD). This includes children assessed as unlikely to achieve or maintain a reasonable level of health or development, or whose health and

For peer review only - http://bmjopen.bmj.com/site/about/guidelines.xhtml

development is likely to be significantly or further impaired, without the provision of services; or is a child who is disabled. Secondary outcomes were additional formally reported measures of maltreatment (referral to CSCS, child being on a Child Protection Plan, CIN categorisation, Looked after status (mother, child)); Associated measures of maltreatment (recorded injuries and ingestions, non-attendance rates for hospital appointments); Maternal outcomes (subsequent pregnancies); Child health, developmental and educational outcomes (special educational needs, early educational attendance and assessments (Early Years Foundation Stage Profile (EYFSP), KS1)); and health resource use. Study assessment domains, outcomes and principal data providers and analysis are described in the appendix (Table S2).

Power calculation

Our sample size was fixed by the cohort available from BB:0-2. However, for CIN status, while UK data on rates are not specific to the age-range of interest, the rate per 10,000 general population aged 5-9 years is 4.6% for local authorities covering BB:0-2 trial sites.[15] The rate of CIN status would be expected to be greater in the specific study sample. Therefore, we assumed a rate of 8%, hypothesised that FNP would reduce the presence of CIN in the first six years and assumed a difference of 4% would be important. To detect a difference of 4% (4% vs 8%) would require 602 children in each arm with 80% power and a two-sided 5% alpha level. With 1562 children available for follow-up (i.e. excluding mandatory withdrawals) and follow up through administrative records with 10% loss in tracking and linkage we would have 1405 participants, sufficient data to assess the primary outcome.

Efforts to address sources of bias

All maltreatment and child developmental outcomes were independently state-verified (social worker) reports or teacher-assessments respectively, recorded in administrative service data and abstracted from national data providers. By using administrative data and established linking fields we were able to minimise bias due to loss to follow-up. An a priori statistical analysis plan described primary, secondary (including sub-group) and sensitivity analyses, and any additional exploratory analysis is clearly highlighted.

Statistical analysis

Our analysis involved participants whose identifiers could be sent, linked and records released by the respective data providers. This excluded participants whose individual data could be used but could not be matched due to incorrect linking fields, other exclusions from health or education: e.g. private or home schooling and National opt-outs. Imbalance between the BB:0-2 and eligible BB:2-6 participants was quantified using descriptive statistics in baseline demographics, clinical and questionnaire data. We conducted all analyses on an intention-to-treat (ITT) basis with complete case

For peer review only

population (with follow-up data). Therefore, participants remained in the BB:0-2 trial groups they were randomised to regardless of the intervention received. We used multilevel modelling to allow for clustering of effect within the 18 sites and family nurse level and both fitted as random effects. Where clustering was minimal at the family nurse level, results from the two-level model are presented. Parameter estimates are reported alongside a 95% CI and p-value. We adjusted for variables used in randomisation (smoking status, gestational age, language). We defined loss to follow up as a child death or adoption. We were able to determine both of these outcomes. We excluded children from analyses of binary outcomes where follow-up was incomplete and no event was observed. With time-to-event analyses, children were censored at these events.

Primary comparative analysis examined whether the firstborn child(ren) ('BB:0-2') had ever been referred to CSCS and classed as a child in need at any point between birth and 6 years of age (appendix). We used logistic multilevel modelling to determine differences in the proportion defined as in need between the two trial arms. We present the resulting estimate as an odds ratio. For binary and categorical outcomes, comparative analysis used logistic and multinomial modelling respectively with results presented as odds ratios (OR). For continuous outcomes we used linear regression and present mean differences. For count data we used Poisson regression modelling; where event distribution displayed signs of over dispersion a Negative Binomial regression model was used (or zero-inflated model if appropriate). Results are presented as incidence rate ratios (IRR). Time to event data used Cox regression modelling presented with hazard ratios (HR). To reduce problems with multiple comparisons, some outcomes were only assessed descriptively (for example, we prioritised and tested overall scores for a Good Level of Development in the Early Years Profile, rather than the individual areas of learning). Similarly, for outcomes that we expected to reflect a small number of events or higher levels of missingness we also took a descriptive approach. Nevertheless, we present all a priori comparative analyses in the main tables and all other descriptive and exploratory analyses in the supplementary materials.

We received KS1 data in two waves from NPD. The first wave was for children assessed in the 2016/17 academic year (received in May 2018). The second wave was for younger children, assessed in 2017/18 (received in May 2019). Reading, maths and science were examined for the whole cohort over both academic years. However, the 2017/18 writing assessment was changed with the consequence that judgements in 2018 are not directly comparable to judgements made using the previous interim frameworks (2016/2017). Therefore, we only assessed statistically writing using 2016/17 data. We presented the data from 2017/18 descriptively. Sensitivity analyses for primary and selected secondary outcomes (EYFSP, KS1, referral to CSCS) included adjusting for hypothesised confounders at baseline, dosage effects (i.e. number of visits) using complier average causal effects (CACE) modelling and sub-group analysis of potential effect moderators and mediators (maternal

deprivation, adaptive functioning, NEET (not in education, employment or training) status at recruitment, maternal age at recruitment, child sex, maternal care status, duration of maternal care and domestic abuse self-reported at 24 months) as interaction terms in the main comparative models.

Results of all planned sensitivity analyses are presented in the appendix. An additional post hoc sensitivity analysis adjusted for the child's month of birth (categorised into quarter of birth) in the EYFSP and KS1 outcomes.

Economic Analysis

A cost consequences analysis of FNP over the full follow-up period (BB:0–2 and BB:2–6) took a secondary health care (UK NHS) perspective. The principal data source was Hospital Episode Statistics (HES) records (inpatient, outpatient, A&E), which were coded in Healthcare Resource Group (HRG) Grouper and matched to appropriate NHS Reference costs. Maternal and child resource use were costed separately and valued in £ sterling. Only direct healthcare costs were analysed. Indirect costs such as lost wages or childcare costs were not available within the routinely collected data. Where data were absent in HES records it was assumed no resource was incurred. No primary care data were available beyond 18 months postpartum. Costs were discounted back from year of event to baseline (2009/10) at an annual rate of 3.5% as described by Central Government's guidance on appraisal and evaluation. [16]

Data management

Small numbers were handled according to SAIL Databank rules where any cell counts under five were suppressed in reporting. Where abortion data are presented, we handled in accordance with the data sharing agreement with DHSC where counts less than 9 were suppressed. Data were analysed using Stata v16 and IBM SPSS v23.0.

Patient and public involvement

Activities involved four groups of young people (CASCADE Voices, Our Place, FNP graduates and ALPHA). These activities contributed to three aspects of the study: data-linkage methods (e.g. to optimise acceptability of the proposed dissent model, use of data linkage and communication of these methods),[17] study outcomes (by seeking views on the importance of the study outcomes) and dissemination of findings (including best way to present results, method of dissemination).

Results

The study population following the BB:0-2 trial tracing and matching process is shown in Figure 1. Five women were ineligible due to not meeting original trial entry criteria and 78 were mandatory

For peer review only - http://bmjopen.bmj.com/site/about/guidelines.xhtml

withdrawals mainly due to fetal or infant death and adoption.[6] The 110 women who electively withdrew during the trial were given the opportunity to opt-out from further data usage, 16 of whom removed consent for any further contact. Therefore, we sent the details for these 94 women for tracing alongside the remaining 1,452 mothers (a total of 1,546 mother-child dyads) to update their contact details and thereby improve matching. One dyad was subsequently removed as deceased and the remaining 1,545 contacted by letter, email or text of whom eight dissented from having their records linked. 1,537 mothers and 1,547 children were sent to the DfE and NHS Digital for matching. These formed the population for the BB:2-6 study. Match rates for children were 98.3% (NHS Digital) and 97.4% (DfE).

Numbers matched to and returned for analysis differed by data source and population (Table S3). Few mothers, compared with children, were matched to any DfE data set (19% vs. 97%, respectively) due to having ceased their education. These events were based mainly on CIN data. For selected maternal and child baseline variables and delivery of FNP assessed against programme fidelity goals, the BB:2-6 sample appears broadly representative of the original BB:0-2 cohort (Tables S4 and S5). Baseline characteristics of the BB:2-6 mothers and children were well balanced between trial arms (Table 1).

Table 1 Socio-demographic characteristics of mother and babies at baseline by trial arm

	**FNP N=766**	**Usual care N=771**
**Maternal age at recruitment (years)**	17·9 (17·0 to 18·8)	17·9 (16·9 to 18·8)
**Ethnicity**		
White background	675 (88·1%)	680 (88·2%)
Mixed background	44 (5·7%)	38 (4·9%)
Asian background	15 (2·0%)	10 (1·3%)
Black background	29 (3·8%)	40 (5·2%)
Chinese or Other background	<5	<5
**Relationship status with baby's father**		
Married	6 (0·8%)	10 (1·3%)
Separated	72 (9·4%)	78 (10·1%)
Closely involved/boyfriend	582 (76·0%)	586 (76·0%)
Just friends	106 (13·8%)	97 (12·6%)
**NEET Status***		
Yes	315/764 (41·1%)	319/769 (41·4%)
No	345/764 (45·0%)	333/769 (43·2%)

For peer review only - http://bmjopen.bmj.com/site/about/guidelines.xhtml

	FNP N=766	Usual care N=771
**IMD Overall Score†**	38·1 (24·6 to 52·6)‡	38·1 (25·5 to 51·6)§
**Generalised self-efficacy scale (score 10 to 40)¶**	30·0 (28·0 to 33·0)‖	30·0 (27·0 to 32·0)**
**Adaptive functioning:**		
**Difficulty in at least one basic skill**		
Yes	213/765 (27·8%)	184/770 (23·9%)
No	552/765 (72·2%)	586/770 (76·1%)
**Had 3 or less life skills (out of 5)**		
Yes	185/763 (24·2%)	218/769 (28·3%)
No	578/763 (75·8%)	551/769 (71·7%)
**At least one burden**		
Yes	214/759 (28·2%)	237/767 (30·9%)
No	545/759 (71·8%)	530/767 (69·1%)
**Cigarette smoking participant self-reported**		
Ever smoked		
Yes	615 (80·3%)	612 (79·4%)
No	151 (19·7%)	159 (20·6%)
**Child characteristics**	**FNP N=773**	**Usual Care N=774**
**Sex**		
Male	381/773 (49·3%)	406/773 (52·5%)
Female	392/773 (50·7%)	367/773 (47·5%)
**Birthweight (grams)** Mean (SD)	3223·81 (606·0)††	3215·52 (555·56)‡‡
**NNU admission (direct or subsequent)**		
Yes	76/716 (10·6%)	66/749 (8·8%)
No	640/716 (89·4%)	683/749 (91·2%)

Data are n (%), n/N (%), or median (25th to 75th centile) unless otherwise stated. <5 = numbers suppressed. FNP = Family Nurse Partnership Programme + Usual Care. IMD = Index of Multiple Deprivation. SD= standard deviation. NNU = Neonatal unit. *Definition of NEET status: Not in education employment or training (applicable only to those whose age at end of previous academic year at time of baseline interview was >16). †Higher IMD score indicated more deprivation. Mean IMD Score for England in 2010 was 21.67 [Wilkinson DL, Sniehotta FF, Michie S. Targeting those in need: Baseline data from the first English National Health Service (NHS) health trainer service: *Psychology, health & medicine* 2011; **16**(6): 736-48], ‡N=760. §N=765. ¶ Higher score indicates higher level of self-efficacy. ‖N=764. **N=769. ††N=724. ‡‡N=752.

We excluded referrals resulting in no further action (i.e. children not classed as in need) for the definition of a child in need. Overall, 323 (21.5%) of children in the BB:2-6 cohort had at least one referral to CSCS which resulted as being classed as a child in need at any point between pregnancy (includes pre-birth CIN) and 6 years of age. No difference in rate of children in need was found between FNP (21.2%) and UC (21.7%) arms for the 1,506 children in the analysis (adjusted OR=0.98,

For peer review only

95% CI=0.74, 1.31) (Table 2). The parameter estimate was not affected when adjusting for the number of FNP visits received (efficacy per visit OR=1.00, 95% CI=0.99, 1.004). The number of children referred to CSC and assessed as not requiring additional support were FNP (n=45) and UC (n=46). There were no between arm differences for referral to CSCS, the age at which the first referral was made, the age at which the child was classified as in need and the proportion of children with a protection plan. A small number of children were ever Looked After within the 6-year follow-up period, with no evidence of a difference between trial arms (Table 2). However, the UC arm experienced on average two more months in care compared to the FNP arm. The total number of child deaths across the full follow-up period was less than ten and not further reported by study arm, consistent with the non-disclosive data management policy. Data informing the disability outcome were not available for the years requested. In addition, no difference was found when examining age at first referral or CIN by study arm.

No between arm differences were found in children not taken to at least one outpatient appointment (did not attend rate), emergency attendances or hospital admissions for an injury or an ingestion (Table 2). Overall, 78% of mothers had a subsequent pregnancy (using inpatients, outpatients and abortions data) with no differences by arm (Table 3). Similarly, just over two thirds of the cohort had at least one registerable birth (using only admission data as a source) during the follow-up period with a comparable inter-birth interval. Descriptives for non-tested maltreatment outcomes are shown in Table S6. Descriptives for the number of emergency room attendances and hospital admissions for injuries or ingestions are shown for each trial arm in Tables S7-S8. Exploratory analysis found no differences in length of hospital stay between trial arms following admission for an injury or ingestion for children aged under 1 year old or for children aged between 1 year old and under 2 years old (Table S8). Exploratory analyses of hospital admissions for injuries and ingestions by trial arm shown by age categories (Under 1 year, 1 to 2 years, 25 months to under 6 years) are shown in Table S9.

For peer review only

Table 2 Primary and secondary outcomes: CPS outcomes, healthcare encounters for injuries / ingestions and out-patient non-attendance

**Outcome**	**FNP**	**Usual care (UC)**	**Adjusted* parameter estimate (95% CI)**	**p-value**	**Absolute risk difference (FNP-UC) (95% CI)**
**Child protection outcomes**	**N=760**	**N=746**			
Child in need (CIN) status at any time during the follow-up period					
No	599 (78·8%)	584 (78·3%)	Reference		
Yes	161 (21·2%)	162 (21·7%)	0·98† (0·74 to 1·31)	0·902	-0·5% (-0·5 to 0·4%)
Age at first CIN referral (days)					
Median (25th to 75th centile)	1602·5 (1153.0 to 1978·75)	1649·5 (1130·75 to 1980·25)	0·98‡ (0·79 to 1·23)	0·875	
Missing	*1*	*0*			
Unique§ number of CIN referrals per child	1 (1 to 2)	1 (1 to 2)	0·99‖ (0·82 to 1·19)	0·890	
CIN duration (days)	210 (70·5 to 571·25)	216 (72·75 to 503·25)	1·20‡ (0·90 to 1·59)	0·216	
CIN categorisation (primary need) of first case	***N=161***	***N=162***			
Abuse or neglect	92 (57·1%)	102 (63·0%)	Reference		
Family dysfunction	29 (18·0%)	34 (21·0%)	0·95¶ (0·54 to 1·69)	0·868	
Family in acute stress	13 (8·1%)	10 (6·2%)	1·47¶ (0·61 to 3·54)	0·389	

For peer review only - http://bmjopen.bmj.com/site/about/guidelines.xhtml

Outcome	FNP	Usual care (UC)	Adjusted* parameter estimate (95% CI)	p-value	Absolute risk difference (FNP-UC) (95% CI)
Low income, absent parenting, socially unacceptable behaviour, not stated	13 (8·1%)	6 (3·7%)	2·38‖ (0·86 to 6·57)	0·093	
Child/parent disability	7 (4·3%)	5 (3·1%)	1·47¶ (0·45 to 4·84)	0·525	
Cases other than CIN	7 (4·3%)	5 (3·1%)	1·64¶ (0·50 to 5·37)	0·417	
Referred to Children's social care services (CSCS)					
No	554 (72·9%)	538 (72·1%)	Reference		
Yes	206 (27·1%)	208 (27·9%)	0·97† (0·74 to 1·28)	0·829	-0·8% (-5·3 to 3·7%)
Age at first referral (days)<br>Median (25th to 75th centiles)	1541·5 (1272·75 to 1975·75)	1631·0 (1190·5 to 1984·75)	0·96‡ (0·79 to 1·17)	0·694	
Child Protection Plan					
No	708 (93·2%)	697 (93·4%)	Reference		
Yes	52 (6·8%)	49 (6·6%)	1·04† (0·69 to 1·57)	0·846	0·2% (-0·2 to 0·3%)
Category of child protection plan	***n=52***	***n=49***			
Neglect	17 (32·7%)	22 (44·9%)	Reference		
Emotional	22 (42·3%)	12 (24·5%)	2·77‖ (1·02 to 7·56)	0·046	
Physical	6 (11·5%)	6 (12·2%)	1·25‖ (0·32 to 4·88)	0·743	
Sexual and multiple**	7 (13·5%)	9 (18·4%)	1·13‖ (0·33 to 3·86)	0·848	

For peer review only - http://bmjopen.bmj.com/site/about/guidelines.xhtml

Outcome	FNP	Usual care (UC)	Adjusted* parameter estimate (95% CI)	p-value	Absolute risk difference (FNP-UC) (95% CI)
Looked after status††					
No	735 (96·7%)	719 (96·4%)	Reference		
Yes	25 (3·3%)	27 (3·6%)	0·90† (0·52 to 1·57)	0·712	-0·3% (-0·2 to 0·2%)
Child looked after period of care (months)	10·0 (4·5 to 37·5)	12·0 (6·0 to 33·0)	0·75‖ (0·65 to 0·86)	<0·001	
**Injuries and Ingestions**	**N=760**	**N=759**			
Emergency attendance					
None	317 (41·7%)	344 (45·3%)	Reference		
At least one	443 (58·3%)	415 (54·7%)	1·17† (0·95 to 1·45)	0·149	3·6% (-1·4 to 8·6%)
Number of attendances per child	2 (1 to 2)	2 (1 to 3)	1·09‖ (0·93 to 1·28)	0·281	
Admission to hospital					
None	671 (88·3%)	660 (87·0%)	Reference		
At least one	89 (11·7%)	99 (13·0%)	0·87† (0·63 to 1·20)	0·407	-1·3% (-4·7 to 2·0%)
Unique admissions	109	119			
Ratio of admissions to children	1.22	1.20			
Number of admissions per child, median (25th to 75th centile)	1 (1 to 1)	1 (1 to 1)	0·93‖ (0·67 to 1·29)	0·663	
Length of stay (days) ‡‡	N=106	119			
Median (25th to 75th centile) days	0.5 (0.5 to 1.0)	0.5 (0.5 to 1.0)			

For peer review only - http://bmjopen.bmj.com/site/about/guidelines.xhtml

Outcome	FNP	Usual care (UC)	Adjusted* parameter estimate (95% CI)	p-value	Absolute risk difference (FNP-UC) (95% CI)
Hospital attendance and/or admission					
None	306 (40·3%)	324 (52·7%)	Reference		
At least one	454 (59·7%)	435 (57·3%)	1·11† (0·89 to 1·37)	0·351	2·4% (-2·5 to 7·4%)
DNA outpatient appointment	***N=580***	***N=577***			
Attended all appointments	290 (50·0%)	289 (50·1%)	Reference		
DNA at least once	290 (50·0%)	288 (49·9%)	1·00† (0·79 to 1·26)	0·997	0·09% (-5·6 to 5·8%)

Data are n (%), n/N (%), or median (25th to 75th centile) unless otherwise stated. FNP = Family Nurse Partnership Programme + Usual Care. * FNP compared to usual care. Analysis adjusted for stratification (site), minimisation variables (gestational age, smoking status at recruitment, and first or preferred language); † Odds ratio from logistic model; ‡ Hazard ratio from Cox model; § A unique referral is counted as a distinct referral date per child; ‖ Incidence rate ratio from Poisson model; ¶ Relative risk ratio from multinomial model; ‡‡Using diagnosis in any position; **Multiple indicates when more than one category of abuse is relevant to the child's current plan; †† We reflect here the terminology used at the time of the trial but note the more contemporary 'care experienced' as a more familiar and accepted term ‡‡ 0.5 days indicates an admission and discharge on the same day (could be up to 1 day in hospital). Data available from NHS Digital which show an admission and discharge one day apart may not equate to one full day in hospital (ie the child may have been in hospital for only a few hours if the admission spanned midnight). Source: National Pupil Database, The Department for Education

For peer review only

Table 3 Secondary outcomes: Subsequent pregnancies and registerable birth

	FNP	Usual care	Adjusted* parameter estimate (95% CI)	p-value	Absolute risk difference (FNP-Usual care) (95% CI)
Subsequent pregnancy	*N=753*	*N=753*			
No	163 (21·7%)	163 (21·7%)	Reference		
Yes	590 (78·4%)	590 (78·4%)	1·00† (0·79 to 1·28)	0·984	0·0% (-4·2 to 4·2%)
Subsequent registerable birth	*N=752*	*N=749*			
No	276 (36·7%)	266 (35·5%)	Reference		
Yes	476 (63·3%)	483 (64·5%)	0·95† (0·77 to 1·18)	0·662	-1·2% (-6·0 to 3·7%)
One birth	326 (68·5)	331 (68·5)	0·95‡ (0·76 to 1·19)	0·655	
Two	124 (26·1)	121 (25·1)	0·99‡ (0·73 to 1·34)	0·955	
Three births or more	26 (5·5)	31 (6·4)	0·80‡ (0·46 to 1·39)	0·435	
Inter-birth interval between first and second child (days)<br>Median (25th to 75th centiles)	1027 (590 to 1506·75)	1065 (665 to 1538)	0·99§ (0·88 to 1·13)	0·938	

Data are n (%), n/N (%), or median (25th to 75th centile) unless otherwise stated. FNP = Family Nurse Partnership Programme + Usual Care. * FNP compared to usual care. Analysis adjusted for stratification (site), minimisation variables (gestational age, smoking status at recruitment, and first or preferred language); † Odds ratio from logistic model; ‡ Relative risk ratio from multinomial model; § Hazard ratio from Cox model.

For peer review only - http://bmjopen.bmj.com/site/about/guidelines.xhtml

Table 4 Secondary outcomes: Child health, developmental and educational

Outcome	FNP	Usual care	Adjusted* odds ratio (95% CI)	p-value	Absolute risk difference (FNP-Usual care) (95% CI)
Special educational needs (SEN)† provision	**N=759**	**N=747**			
No	540 (71·1%)	502 (67·2%)	Reference		
Yes	219 (28·9%)	245 (32·8%)	0·83 (0·67 to 1·03)	0·097	-3·9% (-8·6 to 0·7%)
**Early educational attendance**	**N=759**	**N=747**			
Attending an Ofsted registered private, voluntary and independent establishment up to the age of 4 years	334 (43·9%)	308 (41·2%)	1·13 (0·91 to 1·40)	0·281	2·7% (-2·3 to 7·7%)
**School attendance**	**N=754**	**N=740**			
Overall absence					
No absences	14 (1·9%)	14 (1·9%)	Ref		
At least one absence	740 (98·1%)	726 (98·1%)	1·00 (0·47 to 2·12)	0·998	0·0% (-1·4 to 1·5%)
Overall authorised absence					
No absences	26 (3·4%)	26 (3·5%)	Ref		
At least one absence	728 (96·6%)	714 (96·5%)	1·01 (0·58 to 1·75)	0·984	0·1% (-1·8 to 2.8%)
Overall unauthorised absence					
No absences	256 (34·0%)	245 (33·1%)	Ref		
At least one absence	498 (66·0%)	495 (66·9%)	0·95 (0·76 to 1·18)	0·620	-0·8% (-5·6 to 3·9%)

For peer review only - http://bmjopen.bmj.com/site/about/guidelines.xhtml

**Outcome**	**FNP**	**Usual care**	**Adjusted* odds ratio (95% CI)**	**p-value**	**Absolute risk difference (FNP-Usual care) (95% CI)**
**Early Years assessment**	**N=743**	**N=728**			
**Achieving good level of development (GLD) ‡**					
Achieving GLD in all five areas of learning‖	431 (58·0%)	380 (52·2%)	1·26 (1·03 to 1·55)	0·026	5·8% (0·7 to 10·9%)
Achieving GLD in all 17 early learning goals	412 (55·5%)	365 (50·1%)	1·24 (1·01 to 1·52)	0·043	5·3% (0·2 to 10·4%)
**Total point score§** Mean (SD)	32·22 (7·25)	31·59 (7·62)	0·65 ‖ (-0·11 to 1·41)	0·094	
**Key Stage 1 assessments**	**N=740**	**N=732**			
**Reading**					
Lower than expected	257 (34·7%)	289 (39·5%)	Reference		
Reaching at least the expected standard¶	483 (65·3%)	443 (60·5%)	1·23 (0·99 to 1·53)	0·051	4·8 (-0·2 to 9·7%)
Expected standard	*371 (50·1%)*	*337 (46·0%)*	*1·24 (0·99 to 1·56)*	*0·056*	*4·1 (-1·0 to 9·2%)*
Higher standard	*112 (15·1%)*	*106 (14·5%)*	*1·20 (0·88 to 1·65)*	*0·250*	*0·7 (-3·0 to 4·3%)*
**Maths**					
Lower than expected	281 (38·0%)	283 (38·7%)	Reference		
Reaching at least the expected standard¶	459 (62·0%)	449 (61·3%)	1·04 (0·84 to 1·28)	0·731	0·7 (-4·3 to 5·6%)
Expected standard	*392 (53·0%)*	*376 (51·4%)*	*1·06 (0·85 to 1·32)*	*0·611*	*1·6 (-3·5 to 6·7%)*
Higher standard	*67 (9·1%)*	*73 (10·0%)*	*0·93 (0·64 to 1·35)*	*0·711*	*-0·9 (-3·9 to 2·1%)*

For peer review only - http://bmjopen.bmj.com/site/about/guidelines.xhtml

Outcome	FNP	Usual care	Adjusted* odds ratio (95% CI)	p-value	Absolute risk difference (FNP-Usual care) (95% CI)
**Science**					
Lower than expected	203 (27·4%)	219 (29·9%)	Reference		
Reaching at least the expected standard**	537 (72·6%)	513 (70·1%)	1·14 (0·91 to 1·43)	0·254	2·5 (-2·1 to 7·1%)
**Writing academic year 2016/17**	**N=498**	**N=487**			
Lower than expected	257 (51·6%)	278 (57·1%)	Reference		
Reaching at least the expected standard¶	241 (48·4%)	209 (42·9%)	1·24 (0·97 to 1·60)	0·090	5·5 (-0·7 to 11·6%)
Expected standard	*218 (43·8%)*	*182 (37·4%)*	*1·29 (1·00 to 1·68)*	*0·054*	*6·4 (0·3 to 12·5%)*
Higher standard	*23 (4·6%)*	*27 (5·5%)*	*0·92 (0·51 to 1·64)*	*0·769*	*-0·9 (-3·8 to 1·9%)*

Data are n (%), n/N (%), or median (25th to 75th centile) unless otherwise stated. FNP = Family Nurse Partnership Programme + Usual Care. SD=standard deviation. *FNP compared to usual care. Analysis adjusted for stratification (site), minimisation variables (gestational age, smoking status at recruitment, and first or preferred language); † A child with SEN provision is recorded using the following codes (A = School Action, P = School Action Plus, E = Education, Health and Care Plan, S=Statement, K= SEN support) and the N code was used to indicate No SEN support in any of the following datasets between 2013 and 2017. PLASC (Autumn, Spring and Summer terms), AP, PRU Census, and EYC. If for any of the years no response was recorded then the assumption was that the child was not included in the denominator as either not present on Census day or not in school; ‡ Children achieving a good level of development are those achieving at least the expected level within the prime and specific areas of learning; §Total point score ranges from 17 to 51 with a higher score indicating a better level of development; ‖ Mean difference from linear model; ¶ Working at the expected standard and at a greater depth within the expected standard; **Working at a greater depth within the expected standard is not applicable in science. Source: National Pupil Database, The Department for Education

For peer review only - http://bmjopen.bmj.com/site/about/guidelines.xhtml

Evidence of positive programme impacts were found for some educational outcomes assessed at the end of the reception year and at KS1 by which time the child would be seven years old (Table 4). Children in the FNP arm were more likely to reach a good level of development across all five areas of learning by the end of the reception year (58.0%) than children in the UC arm (52.2%) and also to achieve a good level of development in all 17 early learning goals (FNP: 55.5%, UC: 50.1%). The small advantage for children of nurse-visited mothers was consistent across all five areas of learning although not tested statistically (Table S10).

For KS1 reading, writing, maths and science assessments there was no evidence of a difference between arms in the proportion of children reaching at least the expected standard nor specifically in those working at the expected level or at a greater depth of knowledge (Table 4). What is observed from the writing assessment is the increase in the rates of children reaching at least the expected standard between the two academic years (Overall 45.7% in 2016/17 to 66.5% in 2017/18), reflective of all assessments across the two academic years in each arm (Table S11). This is mainly explained by the distribution of births for the children for each academic year (Figure S1). Children with KS1 results for the academic year 2016/17 were born between October 2009 and August 2010. However, there was a skew towards summer-born children (i.e. 42% were born between June and August 2010). Children with KS1 results for the academic year 2017/18 were born between September 2010 and February 2011. Nationally, there is a relationship between rates of children reaching the expected standard and month of birth (e.g. there is a 17% difference in reading for children born in August and September).[18] Therefore, the 2017/18 cohort of children are not fully representative of the whole academic year and are biased towards children who are more likely to achieve. This indicates that the rates of children reaching the expected standard varies by their month of birth and is important as a moderator of programme effect. Importantly, the original trial allocation ensured study arms remained balanced with regards month of birth and the intervention had no impact on total weeks gestation.[6] As a sensitivity analysis, the main analyses were adjusted for the child's month of birth (categorised into quarter of birth) and evidence of a between arm difference was found in reading assessments (Table S12). In addition, the Early Years assessments were adjusted for child's month of birth, which strengthened the association found in Table 4.

Details of all planned sub-group analyses and further exploratory analyses are shown in the appendix (Tables S13 to S18). The latter included exploration of potential surveillance bias for all children assessed as a Child in Need by age 4 years old. This explored whether children in the FNP arm may have been referred and assessed as in need at lower levels of concern than children in the Usual Care arm based upon maternal baseline characteristics (Table S18). Mothers in the Usual care arm were more likely to have not been in education, employment or training than mothers in the FNP arm but there were no other differences at baseline. The two groups of children assessed as in need were also no different on subsequent measures of school readiness.

For peer review only - http://bmjopen.bmj.com/site/about/guidelines.xhtml

Cost

There were negligible resource use and cost differences for both mothers (p=0.393) and children (p=0.865) between study arms (Table S19). The adjusted incremental costs of programme delivery per women in BB:0-2 (£1,811) remain the key observed cost difference between study arms.

Discussion

Principal findings

In this data linkage study we found that adding FNP to usually provided health and social care provided no additional benefit for maltreatment outcomes including the numbers of children referred to CSCS, registered as in need of additional support, given a child protection plan, entering care, attending an emergency department or being admitted for an injury or ingestion, or duration of stay if admitted. While there was no difference between study arms in the small number of children in care, the UC arm experienced on average two more months in care compared to the FNP arm. The total number of child deaths found was less than ten and therefore disclosive and cannot be reported. High rates of children ever registered as in need in both study arms indicates substantial need amongst families. We found the programme led to more children reaching a good level of development at the end of school reception year and, when taking month of birth into account, improvements in reading at KS1. Writing scores improved as a result of FNP for boys, for children of younger mothers and for children of mothers who were not in education, employment or training (NEET) when recruited to the trial. We found no other difference between families who received FNP and those who did not. With no differences in secondary health resource use and costs for FNP, the programme can be considered cost-neutral compared to usual care in the medium-term (2-6 years).

Strengths and weaknesses of the study

The prioritisation of outcomes in our original trial attracted some debate.[19-21] However, the trial and the current follow-on study are both comprehensively reported, and preceded by a priori analysis plans providing transparency and guarding against undue post hoc interpretation.[7-9] Although a primary outcome of child being in need of additional support was incorporated into the study design, we adopted a multimethod multisource approach to look at the range of maltreatment outcomes in drawing our conclusions. The consistent pattern of results within the two principal outcome domains provides greater confidence in what we can conclude. While the study benefited from random allocation in the BB:0-2 trial, when assessing educational outcomes we additionally adjusted for month of birth. This was because at a national level, month of birth is strongly associated with educational outcomes and the distribution of participants recruited to the trial varied by month.

For peer review only

The available maltreatment data provides an essential overview of family experience as recorded in official records but may lack some depth which could have allowed a more nuanced understanding for children referred. The educational data are teacher-reported statutory tests but similarly provide less detailed insight into child functioning which research instruments may have provided. The lack of detailed social care and any primary care resource use data limited the perspective of the economic analysis. However, using administrative data has enabled comprehensive cohort follow-up, with few cases lost for analysis preserving the benefits of the original trial randomisation. The data included are also objectively recorded rather than maternally self-reported, allow for easy comparison nationally and over time. Our approach has gathered more data at less cost than comparable prospective data collection and minimised family burden.

In our previous trial report of outcomes at 24 months postpartum, there were higher rates of safeguarding events recorded in GP notes for children in the FNP arm (n=64/469, 13.6%), compared to the usual care arm (n=38/476, 8.0%).[6] Additionally, more mothers in the FNP arm reported their child had ever been referred to social services (n=119/580, 20.5%) compared to mothers in the usual care arm (n=91/541, 16.8%). These finding can be indicative of surveillance bias or more accurate reporting by mothers in the FNP arm respectively. In the current study a tendency for children to be referred at lower thresholds of concern or closer involvement by attending FNP nurses may have been evidenced through a greater proportion of children referred to social services being assessed as requiring no additional support. However, we did not observe this in our cohort.

With no absolute external criterion for maltreatment, a reliance solely upon formal child protection reports may be limiting. In the US Elmira trial, a review of 42 children (intervention arm n=13, control arm n=29) referred to CPS between birth and their fourth year of life found few differences in the nature of maltreatment recorded. However, measures of the quality of home environment, parenting, child intellectual functioning and use of healthcare services between children's 25th and 50th months of life favoured families in the intervention arm.[22] It has been suggested that both parental and professional behaviour may lead to programme enrolled CPS reported children being at lower risk of harm than their counterparts in receipt of usual care alone. Thresholds for referral to social services may differ between specialist home-visiting nurses with high contact time, more in-depth relationship with clients and different approaches to risk and safety management and other health, social and educational care professionals.[23] For children referred to CSCS and assessed as a Child In Need, we explored whether the trial arms differed on characteristics that may suggest a lower threshold of concern for those in the FNP arm. Our comparative analysis was limited principally to baseline maternal characteristics to reduce the risk of interpreting outcomes subsequent to the Child In Need assessment as evidence of contemporary child concerns. This exploratory analysis found a higher rate of educational, employment and training inactivity at trial baseline amongst mothers in the Usual

For peer review only - http://bmjopen.bmj.com/site/about/guidelines.xhtml

Care arm compared to the FNP trial arm. While no other differences were found, this is consistent to the findings in the Elmira trial.[22] CPS referrals for children in receipt of FNP at lower levels of apparent concern would make it harder to compare on such outcomes, particularly during the programme delivery period (ie up to the child's second birthday). However, subsequent progress of children through the child protection system and later occurring outcomes (for example, children being taken into care) may be less liable to such influences but in our sample such numbers are small.

Verified outcomes documented in administrative records contrast with maternal subjective self-reports for which there may be no potential external criterion (ie a child being referred to CPS is a verifiable event, whereas maternal well-being is not). Nevertheless, the presentation or referral of families and their assessment against legally defined statuses such as being a Child in Need requires professional judgement. It has been suggested that surveillance bias is not likely to be problematic for assessing maltreatment using verified service data (i.e. by inflating the numbers of children being reported when in receipt of home-visiting).[24,25] As others have found this could be higher when clients are actively engaged with services and as noted above, the potential for such bias remains.[26]

It has been proposed that other documented evidence such as hospital admission due to injury and ingestions and in particular duration of subsequent stay may provide a better indicator of maltreatment severity. In the follow-up of the US Memphis trial cohort at two years of the child's life, children in the intervention arm admitted following an injury or ingestion spent fewer days in hospital than their counterparts in the control arm. In the US Elmira trial, no difference between trial arms were found in the number of days hospitalised during the 25- to 50-month period of follow-up after adjustments were made for one outlier (in the intervention arm) assessed as being not relevant to parental caregiving.[27] We explored length of hospital stay following admission for an injury and ingestion both overall and for time periods representing periods of increasing of child mobility (ie under 1 year of age, 1 to under 2 years of age and 25 months to under 6 years of age). These were similar in each case but factors other than severity of clinical need may influence how long children remain in hospital. Wide differences exist between countries for hospital stay duration following injuries, ingestions and other consequences of external causation (when assessed across adult and paediatric populations).[28] One observational UK study of predictors of length of stay amongst paediatric inpatients (all causes) shows the contribution of health service factors in addition to clinical and social factors.[29] Interestingly, a large US study amongst paediatric inpatients found that non-accidental trauma compared to accidental trauma was a strong predictor of having a prolonged hospital stay even after adjusting for injury severity.[30] Therefore, length of hospital stay may be indicative of maltreatment, severity of clinical presentation, health service factors and possibly social factors. Further clarifying the factors that independently drive length of hospital stay and the validity of this outcome as a measure of maltreatment will be valuable.

For peer review only - http://bmjopen.bmj.com/site/about/guidelines.xhtml

Potential sources of bias

The comparisons being made in this study are principally between women in receipt of visits from family nurses, in addition to usually provided health and social care and women receiving usually provided care only. In our previous full trial report, we summarised participant-reported contacts with a wide range of health and social care services up to 18 months post-partum. In addition, the within-trial economic analysis involved imputing values for all resource domains to cover the final six months of the trial period (ie up to 24 months postpartum) and which assumed equivalent resource use at 24 months as reported at 18 months.[8] This included resource use (ie contacts) related to specialist public health nurses (SPHN). The full trial report includes descriptions of both reported and imputed values; however, it should be noted that the concurrently published journal article only included the higher values imputed for the purpose of the economic analysis.[6] As the schedule for delivery of the universal elements of the healthy child Programme (HCP) specifically by SPHNs indicates a decreasing frequency of contact over time (and none from 1 year until by 2-2.5 years) this is likely to be a conservative (overestimate) of actual provision.[31]

As an open label study, it is possible that service professionals may have attempted to compensate for the lack of specialist support from FNP for participants in the usual care arm. However, it should be noted that enhanced care would have been expected as part of usual practice for teenage mothers, for example, through specialist teenage pregnancy midwives who were employed at 14 of the 18 trial sites, with lower caseloads and an emphasis on home-based antenatal care, promotion of healthy relationships and positive parenting. For example, the specification for children's public health services (from pregnancy to age 5) indicates a schedule of universal elements for the Healthy Child Programme, including both universal and progressive elements where young mothers are not under the care of family nurses.[31] The number of midwifery contacts were recorded in the trial and found to be equivalent between FNP and UC arms.[11] The number of home visits from SPHNs reported by trial participants by 18 months was also equivalent between study arms. As would have been expected, the number of clinic contacts with SPHNs was greater for women in the control arm than in the FNP arm. In contrast, women allocated to the intervention arm would have received an average of just over 40 home visits from a family nurse before graduating from the programme. Overall, this suggests a common core of usual care provided across both trial arms, a higher level of SPHN input to the usual care arm reflecting in part the responsibility of delivering the Healthy Child Programme (which was formally allocated to FNP nurses rather than SPHNs for their clients). The capacity of SPHNs to provide additional support will also be limited by their caseload. The maximum caseload currently recommended by the Institute of Health Visiting is one practitioner per 250 children. However, in 2015, 28% of surveyed practitioners in England reported caseloads in excess of 400 children and 12% reported between 500 and 1000+ families.[32] Providing additional support only to mothers with

For peer review only - http://bmjopen.bmj.com/site/about/guidelines.xhtml

apparent need rather than all women on each SPHN's caseload may be considered at least more feasible. If so, and if this actually approached an optimal level of support driven by women's needs as per the progressive universal model then our trial comparison of FNP would still be against best usual care. There are limitations in the data captured on routine health service usage (see relevant excerpts of CRF data for postpartum data collection in Appendix XII). For example, SPHN contact content and duration were not captured. This was due to pragmatic and ethical considerations (eg to not increasingly burden participants for what were already lengthy telephone data collection schedules). Similarly, reported contacts between women in the trial intervention trial and SPHNs would have been worthy of further investigation. It's possible that some reports could in fact have been related to family nurses rather than SPHNs, although perhaps less likely for women engaged in a constructive on-going relationship with a named family nurse. Furthermore, women in the intervention arm who either never engaged with FNP or may have dropped scheduled contact with their family nurse would be expected to have had on-going contact with a SPHN.

The potential for the open label nature of the study to introduce bias through the actions of SPHNs is an important question and can be viewed through the lens of contemporary behavioural change frameworks such as COM-B.[33] This approach looks at three essential conditions required for a change to occur (eg in this instance a SPHN acting to provide enhanced support to a mother) - Capability, Opportunity and Motivation. Firstly, do SPHNs have the skills, training, model of delivery and supportive management structure (ie Capability) to be provide an enhanced service? Many SPHNs would have had similar professional backgrounds and skills to their FNP colleagues, and some expressed similar practice aspirations to those they perceived amongst family nurses. In contrast, most FNP nurses would have been selectively recruited from existing SPHNs and received additional programme training, supervision and a distinct programme delivery model. While the capabilities of SPHNs and FNP nurses certainly overlapped, they were unlikely to be equivalent at the time of the trial. Second, as noted above, the opportunity for SPHNs to deliver additional support would have been limited by their caseload. This was evidenced by the testimony of SPHNs in the trial's process evaluation and reported caseloads of up to 600 women at two trial sites.[8] The reduction to an individual SPHN's own caseload associated with an FNP nurse enrolling up to her caseload maximum of 25 clients would appear to offer limited scope to substantially increase the opportunity to then enhance provision. Finally, would SPHNs be motivated to enhance service provision either in general (eg to all clients who may be seen as likely to benefit from additional support) or specifically to participants allocated to the trial's control arm? In our trial's process evaluation, several SPHNs reported a lack of awareness about whether their clients were in the trial and in some cases a misperception that they shouldn't know either. SPHNs with the potential to provide additional care and to do so specifically for women they knew to be enrolled in the trial and allocated to the control arm (and not say to other women with similar needs unwilling to participate in the trial or who were

For peer review only

not identified to do so) would require considerable motivation. To do so would also represent a wilful attempt to undermine the requirements of a trial supported by their own departments. For this to occur consistently for a large number of SPHNs both within and across the 18 trial sites at a level that may start to impact upon outcomes seems less likely. Given the 'fire-fighting' described by SPHNs in delivering care as usual to their large caseloads, the additional motivation to target additional support to women known to be in the trial seems more a theoretical rather than a practical risk.

Comparison with other studies

The strongest evidence for the programme preventing maltreatment remains from the original US Elmira trial. While the programme reduced the number of substantiated reports of abuse and neglect in the first 15 years of the child's life, the advantage emerged after age four years.[4] We found differences in duration spent in care for looked after children, but the numbers involved are small and across the range of maltreatment outcomes the pattern is of no overall group difference. The programme was no more effective in tested sub-groups. It is worth recognising differences in the population of clients in receipt of the programme across the different study populations, their social circumstances (which also may have changed over time) and how that may determine potential to benefit from FNP.[11] We have not directly explored differences in social care systems between England and the US but these may vary the underlying risk of maltreatment and its likelihood of detection.[34]

School entry reading and maths is of important predictive value for later school achievement.[35] Early childhood educational investment programmes typically show a modest impact upon cognitive and achievement scores (e.g. weighted average of 0.21 standard deviations) with some decline in observed impact being attributed to general improvements in both school and home environments.[36,37] The question of comparator is therefore of particular relevance. In this study, a range of state and third sector funded services (including midwifery and health visiting) would have been available as usual care, possibly limiting the incremental benefit possible from the family nurse partnership programme.[11] Nevertheless, as noted above women allocated to FNP received substantial input from family nurses, albeit fewer than possible according to the maximum fidelity targets at the time of the trial.

Home visiting interventions, which aim to impact upon a range of outcomes, have shown similarly modest effects on child cognitive outcomes (e.g. mean Effect Size 0.25, 96% CI 0.11, 0.38) with considerable variability due to programme composition (e.g. greater effects with programmes addressing parental responsiveness, sensitivity to care and nurturing and rehearsal and role-playing).[38] Our BB:0-2 study found benefits at age two years for children in the FNP arm for both language and developmental outcomes but these outcomes were maternally reported and potentially subject to

For peer review only - http://bmjopen.bmj.com/site/about/guidelines.xhtml

reporting bias in the open trial.[9] This new medium-term evidence for programme impact upon developmental outcomes adds to that picture, although the size of effect remains small. Similar medium-term programme impact (to age six years old) on developmental outcomes (eg, intellectual functioning and receptive language scores) was reported from the Memphis trial.[39] A reanalysis of those data found cognitive benefits at age 6 years attributed to both programme induced improvements in maternal traits and family life investments at age 2.[40]

Unanswered questions and future research

Given small but positive findings for developmental outcomes, tracking the cohort through to subsequent school years is warranted. As social care data are equally accessible via the NPD there is value in observing the trajectory for maltreatment outcomes given that the principal maltreatment findings from the US Elmira trial are located in the 15-year follow-up. Linkage to data in other sectors such as welfare would provide a greater understanding of broader impact for families. Such work should be driven by the programme's logic model, existing evidence for long-term programme impact, informed by programme evolution in England and supplemented by work to understand what may explain differing programme benefits across national settings.[4, 41]

Conclusions

There are no evident programme benefits for maltreatment outcomes by age six but the FNP generates higher rates of child attainment at the end of the reception year and at KS1. The adapted programme remains locally commissioned and delivered in England.[41,42] Local needs and priorities may determine the weight attached to these different sets of outcomes.

Acknowledgements

The Centre for Trials Research receives funding from Health and Care Research Wales and Cancer Research UK. The work was undertaken with the support of The Centre for the Development and Evaluation of Complex Interventions for Public Health Improvement (DECIPHer), a UKCRC Public Health Research Centre of Excellence. Joint funding (MR/KO232331/1) from the British Heart Foundation, Cancer Research UK, Economic and Social Research Council, Medical Research Council, the Welsh Government and the Wellcome Trust, under the auspices of the UK Clinical Research Collaboration, is gratefully acknowledged.

Data re-use statement: Copyright © (2019), the Health and Social Care Information Centre. Re-used with the permission of the Health and Social Care Information Centre. All rights reserved.

We would like to thank all members of CASCADE voices, ALPHA and Our Place for contributing to the study including those who co-ordinated access to these individuals. Thanks are due to the FNP

For peer review only - http://bmjopen.bmj.com/site/about/guidelines.xhtml

teams in Bristol, Bath and Wiltshire who helped us to make contact with FNP clients and graduates during our PPI work. We would also like to acknowledge the contribution of members of the study steering committee - Ann Louise Kinmonth (Chair), David Low, Gordon Taylor, Nina Biehal, Rachel Tonkin, Stavros Petrou, and Silvia van den Heijkant.

This work uses data provided by patients and collected by the NHS as part of their care and support as well as data from schools and local authorities. Using patient and other public data is vital to improve health and care for everyone. There is huge potential to make better use of information from people's patient records, to understand more about disease, develop new treatments, monitor safety, and plan NHS services. Patient and other public data should be kept safe and secure, to protect everyone's privacy, and it's important that there are safeguards to make sure that it is stored and used responsibly. Everyone should be able to find out about how patient data is used. #datasaveslives You can find out more about the background to this citation here: https://understandingpatientdata.org.uk/data-citation. [To note. This has been adapted to include other data sources not made available from the NHS such as education and social services data].

Footnotes

Author contributions: MR was the chief investigator and acts as guarantor for the study. MR, FLW, RCJ, LA, SC, DF, KH, JK, GM, EOJ, RP, JSa, JSe, TS contributed to study design. FLW was the study manager. RCJ was the lead statistician. DF supervised the health economics. MR, FLW, RCJ, LA, SC, DF, KH, JK, GM, EOJ, RP, JSa, JSe, TS were responsible for study management. GM and LA were the data managers. JK and JS led for public involvement. GM and LA cleaned the data. RCJ designed and conducted the analysis. RP conducted the economic analysis. MR, JSa, RCJ, SC, KH, JK, EOJ, JSe were co-applicants on the original funding application. MR, FLW, RCJ, LA, SC, DF, KH, JK, GM, EOJ, RP, JSa, JSe, TS interpreted the results of the analysis. RC-J and RP were responsible for producing the tables. MR, FLW, RC-J and RP drafted the study report. MR, FLW, RCJ, LA, SC, DF, KH, JK, GM, EOJ, RP, JSa, JSe, TS reviewed manuscript drafts, revised for important intellectual content and approved the final version.

Funding: This project was funded by the National Institute for Health Research Public Health Research (NIHR PHR) Programme (reference:11/3002/11). The views and opinions expressed therein are those of the authors and do not necessarily reflect those of the NIHR PHR Programme or the Department of Health. The funders had no role in considering the study design or in the collection, analysis, interpretation of data, writing of the report, or decision to submit the article for publication.

Competing interests: All authors have completed the ICMJE uniform disclosure form at www.icmje.org/coi_disclosure.pdf and declare: all authors declare financial support from NIHR PHR for the submitted work; no financial relationships with any organisations that might have an interest in

For peer review only

the submitted work in the previous three years; no other relationships or activities that could appear to have influenced the submitted work.

Ethical approval: Ethical approval for this study was granted by the Health Research Authority research ethics committee (Wales REC 3; REC reference 14/WA10062) and section 251 support was provided by the Confidentiality Advisory Group (CAG reference CAG 10-08(b)/2014).

Data sharing: Anyone wishing to access the linked datasets for research purposes should apply via the CAG to the Health Research Authority to access patient identifiable data without consent and then to the NHS Digital, National Pupil Database and the abortion statistics – Department for Health and Social Care. In the first instance, enquiries about access to the data should be addressed to Professor Mike Robling, Centre for Trials Research, Cardiff University.

The lead author (MR) affirms that the manuscript is an honest, accurate, and transparent account of the study being reported; that no important aspects of the study have been omitted; and that any discrepancies from the study as planned (and, if relevant, registered) have been explained.

Dissemination to participants and related public communities: The findings from the BB:2-6 study, including those from this paper will be disseminated directly to participants where valid contact details remain available, via social media and conference presentations. This will include the use of lay-informed infographic materials. Summary results will also be made available on the study webpage [https://evidencinghomevisiting.co.uk/].

References

1. Olds DL, Henderson CR, Chamberlin R, Tatelbaum R. Preventing Child Abuse and Neglect: A Randomized Trial of Nurse Home Visitation. Pediatrics. 1986;78(1). Available from: http://pediatrics.aappublications.org/content/pediatrics/78/1/65.full.pdf

2. Kitzman H, Olds DL, Henderson CR, Hanks C, Cole R, Tatelbaum R, et al. Effect of Prenatal and Infancy Home Visitation by Nurses on Pregnancy Outcomes, Childhood Injuries, and Repeated Childbearing. JAMA. 1997;278(8):644. Available from: http://jama.jamanetwork.com/article.aspx?doi=10.1001/jama.1997.03550080054039

3. Olds DL, Robinson J, O'brien R, Luckey DW, Pettitt LM, Henderson CR, et al. Home Visiting by Paraprofessionals and by Nurses: A Randomized, Controlled Trial. Pediatrics. 2002;110(3). DOI: 10.1542/peds.110.3.486 Available from: http://pediatrics.aappublications.org/content/110/3/486.short

4. Olds DL, Eckenrode J, Henderson CR, Kitzman H, Powers J, Cole R, et al. Long-term Effects of Home Visitation on Maternal Life Course and Child Abuse and Neglect Fifteen-Year Follow-up of a Randomized Trial. JAMA. 1997;278(8):637–43. DOI: 10.1001/jama.1997.03550080047038

For peer review only

5. Mejdoubi J, van den Heijkant SC, van Leerdam FJ, Heymans MW, Crijnen A, Hirasing RA. The effect of VoorZorg, the Dutch nurse–family partnership, on child maltreatment and development: a randomized controlled trial. PLOS ONE 2015;10:e0120182. https://doi.org/10.1371/journal.pone.0120182

6. Robling M, Bekkers MJ, Bell K, Butler CC, Cannings-John R, Channon S, et al. Effectiveness of a nurse-led intensive home-visitation programme for first-time teenage mothers (Building Blocks): A pragmatic randomised controlled trial. Lancet. 2016;387:146–55. Available from: http://www.thelancet.com/journals/lancet/article/PIIS0140-6736(15)00392-X/fulltext

7. Lugg-Widger F, Cannings-John R, Channon Sue, Fitzsimmons D, Hood K, Jones K, et al. Assessing the medium-term impact of a home-visiting programme on child maltreatment in England: protocol for a routine data linkage study. BMJ Open. 2017;7(e015728) . Available from: http://bmjopen.bmj.com/content/bmjopen/7/7/e015728.full.pdf

8. Robling M, Bekkers MJ, Bell K, Butler CC, Cannings-John R, Channon S, et al. The Building Blocks Trial (Main report). 2014. [cited 2017 Jul 17] Available from: https://www.cardiff.ac.uk/__data/assets/pdf_file/0009/504729/Building-Blocks-Full-Study-Report.pdf

9. Owen-Jones E, Bekkers M-J, Butler CC, Cannings-John R, Channon S, Hood K, et al. The effectiveness and cost-effectiveness of the Family Nurse Partnership home visiting programme for first time teenage mothers in England: a protocol for the Building Blocks randomised controlled trial. BMC Pediatr. 2013;13:1. DOI: 10.1186/1471-2431-13-114

10. Olds D. Prenatal and Infancy Home Visiting by Nurses: From Randomized Trials to Community Replication. Prevention Science, Vol 3, No 3 2002 DOI: 10.1023/a:1019990432161

11. Robling M, Cannings-John R, Channon S, Hood K, Moody G, Poole R, et al. What is usual care for teenagers expecting their first child in England? A process evaluation using key informant mapping and participant survey as part of the Building Blocks randomised controlled trial of specialist home visiting. BMJ Open. 2018;8(5). doi:10.1136/bmjopen-2017-020152

12. Lyons RA, Jones KH, John G, Brooks CJ, Verplancke J-P, Ford D V, et al. The SAIL databank: linking multiple health and social care datasets. BMC Med Inform Decis Mak. 2009;9(9) https://doi.org/10.1186/1472-6947-9-3

13. Jones KH, Ford D V., Jones C, Dsilva R, Thompson S, Brooks CJ, et al. A case study of the Secure Anonymous Information Linkage (SAIL) Gateway: A privacy-protecting remote access system for health-related research and evaluation. J Biomed Inform. 2014;50:196–204 https://doi.org/10.1016/j.jbi.2014.01.003

14. Jones KH, Ford DV, Thompson S, Lyons R. A Profile of the SAIL Databank on the UK Secure Research Platform. Int J Popul Data Sci. 2019;4(2)https://doi.org/10.23889/ijpds.v4i2.1134

15. Department for Education. Children In Need in England, including their characteristics and further information on children who were the subject of a child protection plan (2009-10 Children in Need census, Final. 2010. [cited 2020 Jun 30] Available from: https://www.gov.uk/government/statistics/children-in-need-in-england-including-their-characteristics-and-further-information-on-children-who-were-the-subject-of-a-child-protection-plan-2009

16. HM Treasury. The Green Book, Central Government Guidance on Appraisal and Evaluation. 2020. [cited 2020 Dec 7]. Available from: https://assets.publishing.service.gov.uk/government/uploads/system/uploads/attachment_data/file/938046/The_Green_Book_2020.pdf

For peer review only

17. Lugg-Widger F, Cannings-john R, Angel L, Moody G, Segrott J, Kenkre J, et al. Assessing the impact of specialist home visiting upon maltreatment in England : a feasibility study of data linkage from a public health trial to routine health and social care data. Pilot feasibility Stud. 2018;4(1):98. https://doi.org/10.1186/s40814-018-0294-4

18. Department for Education. Phonics screening check and Key stage 1 assessments in England, 2016 (SFR 42/2016). London: Department for Education; 2016. [cited 2020 Dec 7]. Available from: https://assets.publishing.service.gov.uk/government/uploads/system/uploads/attachment_data/file/577806/SFR42_Phonics_KS1_2016.pdf

19. Olds D. Building evidence to improve maternal and child health. Vol. 387, The Lancet. Lancet Publishing Group; 2016. p. 105–7 [cited 2020 Jul 23]. Available from: http://dx.doi.org/10.1016/S0140-6736

20. Barlow J, Barnes J, Sylva K, Fonagy P, Fearon P. Questioning the outcome of the Building Blocks trial. Vol. 387, The Lancet. Lancet Publishing Group; 2016. p. 1615–6 [cited 2020 Jul 23]. Available from: http://www.dh.gov.uk/

21. Robling M, Torgerson D, Pickett K, Channon S, Sanders J. Questioning the outcome of the Building Blocks trial - Authors' reply. Vol. 387, The Lancet. Lancet Publishing Group; 2016. p. 1616–7 [cited 2020 Jul 23]. Available from: http://www.dh.gov.uk/

22. Olds D, Henderson CR, Kitzman H, Cole R. Effects of prenatal and infancy nurse home visitation on surveillance of child maltreatment. Pediatrics 1995; 95; 365-372.

23. Williams, VN, Ayele, R, Shimasaki, S, Tung, GJ, Olds, D. "Risk assessment practices among home visiting nurses and child protection caseworkers in Colorado, United States: A qualitative investigation." Health & Social Care in the Community. 2019;27(5): 1344-52. doi.org/10.1111/hsc.12773

24. Drake B, Jonson-Reid M, Kim H. Surveillance bias in child maltreatment: a tempest in a teapot. Int J Environ Res Public Health 2017;14:E971. https://doi.org/10.3390/ijerph14090971

25. Chaffin M, Bard D. Impact of intervention surveillance bias on analyses of child welfare report outcomes. Child Maltreat 2006;11:301–12. https://doi.org/10.1177/107755950629

26. Zielinski DS, Eckenrode J, Olds DL. Nurse home visitation and the prevention of child maltreatment: Impact on the timing of official reports. Dev Psychopathol. 2009;21(2):441–53 [cited 2019 Aug 2]. Available from: https://www.cambridge.org/core/product/identifier/S0954579409000248/type/journal_article

27. Olds DL, Henderson Jr CR, Kitzman H. Does prenatal and infancy nurse home visitation have enduring effects on qualities of parental caregiving and child health at 25 to 50 months of Life? Pediatrics 1994; 93; 89-98.

28. Hospital discharges and length of stay statistics. [cited 2021 Jul 30] Available from: https://ec.europa.eu/eurostat/statistics-explained/index.php?title=Hospital_discharges_and_length_of_stay_statistics#Average_length_of_hospital_stay_for_in-patients

29. Heys M, Rajan M, Blair M. Length of paediatric inpatient stay, socio-economic status and hospital configuration: a retrospective cohort study. BMC Health Services Research 2017 17: 274 DOI 10.1186/s12913-017-2171-x

30. Livingston JK, Grigorian A, Kuza CM, Lekawa M, Bernal N, Allen A, Nahmais J. Non-accidental trauma increases length of stay and mortality in pediatric trauma. Pediatric Surgery International (2019) 35:779–784 doi.org/10.1007/s00383-019-04482-5

For peer review only - http://bmjopen.bmj.com/site/about/guidelines.xhtml

31. Department of Health. Public health functions to be exercised by NHS England Service specification No.27 Children's public health services (from pregnancy to age 5). 2013 [cited 2022 Jan 12]. Available from https://assets.publishing.service.gov.uk/government/uploads/system/uploads/attachment_data/file/192978/27_Children_s_Public_Health_Services__pregnancy_to_5__VARIATION_130422_-_NA.pdf

32. Institute of Health Visiting. Health Visiting in England: State of Health Visiting in England: Results from a survey of 1040 practising health visitors. 2020 [cited 2021 May 21]. Available from: https://www.activematters.org/wp-content/uploads/2020/03/State_of_Health_Visiting_survey.pdf

33. Michie S, van Stralen MM, West R. The behaviour change wheel: A new method for characterising and designing behaviour change interventions. Implementation Science 2011; 6: 42 doi: 10.1186/1748-5908-6-42

34. Moody G, Cannings-John R, Hood K, Kemp A, Robling M. Establishing the international prevalence of self-reported child maltreatment: A systematic review by maltreatment type and gender. Vol. 18, BMC Public Health. BioMed Central Ltd.; 2018. p. 1164 [cited 2020 Jul 23]. Available from: https://bmcpublichealth.biomedcentral.com/articles/10.1186/s12889-018-6044-y

35. Duncan GJ, Dowsett CJ, Claessens A, Magnuson K, Huston AC, Klebanov P, et al. School Readiness and Later Achievement. Dev Psychol. 2007;43(6):1428–46.

36. Duncan G, Magnusson K. Early childhood interventions for low-income children. Focus (Madison). 2014;31.

37. Duncan G, Magnuson K. Investing in Preschool Programs. J Econ Perspect. 2013;27(2):109–31.

38. Filene JH, Kaminski JW, Valle LA, Cachat P. Components associated with home visiting program outcomes: A meta-analysis. Pediatrics. 2013;132(SUPPL.2):S100–9 [cited 2021 Jan 8]. Available from: www.pediatrics.org/cgi/doi/10.1542/peds.2013-1021H

39. Olds DL, Kitzman H, Cole R, Robinson J, Sidora K, Luckey DW, et al. Effects of Nurse Home-Visiting on Maternal Life Course and Child Development: Age 6 Follow-Up Results of a Randomized Trial. Pediatrics. 2004;114(6):1550–9. Available from: http://pediatrics.aappublications.org/cgi/doi/10.1542/peds.2004-0962

40. Heckman JJ, Holland ML, Makino KK, Pinto R, Rosales-Rueda M. An Analysis of the Memphis Nurse-Family Partnership Program. NBER Work Pap. 2017;23610. Available from: http://www.nber.org/papers/w23610%0Ahttp://www.nber.org/papers/w23610

41. Family Nurse Partnership National Unit & Dartington Service Design Lab. FNP ADAPT: Using evidence, pragmatism and collaboration to change the FNP programme in England. London, UK; 2020. Available from: www.fnp.nhs.uk

42. The Family Nurse Partnership |. [cited 2020 Jul 23]. Available from: https://www.fnp.nhs.uk/

For peer review only - http://bmjopen.bmj.com/site/about/guidelines.xhtml

Figure Legend

Figure 1 Participant flow chart

Describing follow-up within the BB0:0-2 trial and formation of the BB:2-6 study cohort. MW=Mandatory withdrawals; EW=elective withdrawals; M=Mother; C=Child, FNP=Family Nurse Partnership (trial arm); UC=Usual care (trial arm); BB:0-2=Building Blocks Randomised Controlled Trial; BB:2-6=Building Blocks Cohort Study; NPD=National Pupil Database

For peer review only

Figure 1 Flow chart

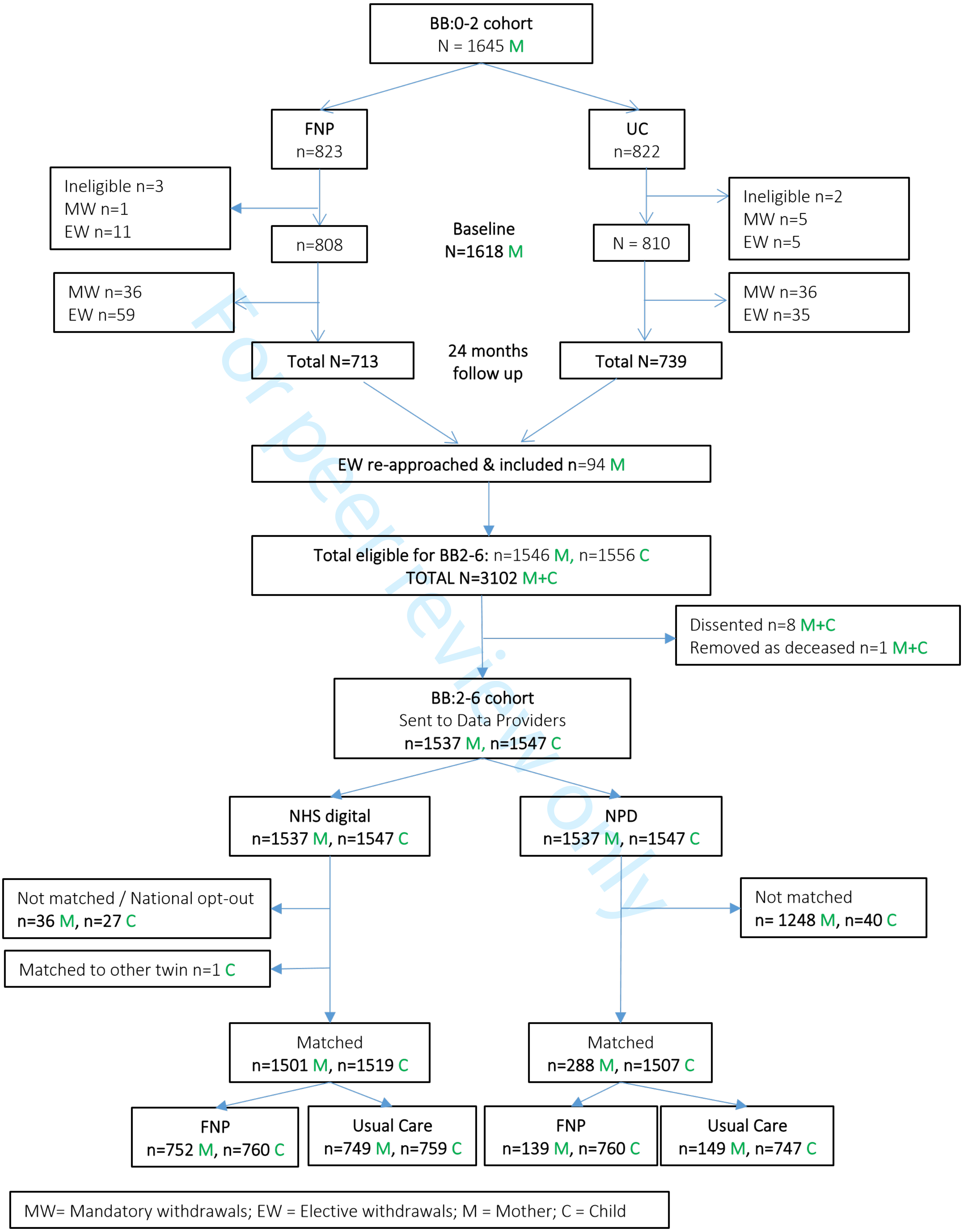

For peer review only

Supplementary appendix

I. Number of valid FNP visits received

- Table S1 BB:2-6 study population data: Valid FNP visits per phase

II. Study assessment domain, outcomes, principal data providers and data obtained

- Table S2 BB:2-6 Study assessment domains, key and exploratory outcomes and principal data providers

III. Data accrued from providers

- Table S3 Numbers (%) of mothers and children with data returned by dataset

IV. Further details of Child in Need outcome

V. Representativeness of study sample

- Table S4 Maternal characteristics of those in BB:0-2 compared to those in the BB:2-6
- Table S5 Child characteristics of those in the BB:0-2 study compared to those in the BB:2-6 study

VI. Descriptive secondary outcomes and additional Key Stage 1 results

- Table S6 Child In Need referrals, Children's Social Services referrals and hospital admissions: descriptives
- Table S7 Emergency attendance to hospital by injury or ingestion, by trial arm
- Table S8 Admissions by injury/ingestion type and trial arm
- Table S9 Hospital admissions for injuries and ingestions by trial arm and broken down by age categories
- Table S10 Child health, developmental and educational: descriptives
- Table S11 Children reaching the expected standard in KS1 assessments by trial arm and academic year
- Figure S1 Distribution of children's month of birth by trial arm
- Table S12 Comparison of parameter estimates before and after adjustment for child's month of birth

VII. Results of planned sub-group analysis

- Table S13 Subgroup analyses for referrals to Children's social care services
- Table S14 Subgroup analyses for child in need status
- Table S15 Predefined subgroup analyses on Early Years Assessment total point score
- Table S16 Subgroup analyses on KS1 assessments reaching the expected standard (RES)
- Table S17 Subgroup analyses on reaching the expected standard in the writing KS1 assessment for 2016/17 academic year

VIII. Treatment efficacy

IX. Exploration of surveillance bias upon Child In Need outcome assessment

- Table S18 Baseline maternal characteristics, school readiness outcomes for children referred to CSC and classed as a Child In Need, before the age of 4 years old by intervention arm.

X. Missing data

XI. Cost Consequence Analysis

For peer review only - http://bmjopen.bmj.com/site/about/guidelines.xhtml
For peer review only

- Table S19 Cost-consequence balance sheet for the family-nurse based intervention for the four years between children's ages 2 and 6.

XII. Postpartum resource use data collected for SPHN contacts

For peer review only

For peer review only - http://bmjopen.bmj.com/site/about/guidelines.xhtml

I. Number of valid FNP visits received

Of the 766 women in the FNP arm in the BB:2-6 cohort, 69 did not enrol for FNP. For the remaining 697 who were randomised and enrolled, 695 had received at least one valid visit and two did not receive any valid visits across the three phases (pregnancy, infancy and toddlerhood). The descriptives in Table S1 are based on 697 women. A greater proportion of women received no visits during the infancy and toddler phases (40 (5.7%) and 98 (14.1%) respectively) compared to the pregnancy phase (6 (0.7%)). This is similar to that reported in the BB:0-2 cohort with comparable median valid visits and proportions reaching the fidelity goals (see Lancet Supplementary appendix Table S11.3).[1]

Table S1 BB:2-6 study population data: Valid FNP visits per phase

	**Pregnancy**	**Infancy**	**Toddlerhood**
N women randomised and enrolled to FNP	697	697	697
Women not receiving any valid visits n(%)	6 (0·7)	40 (5·7)	98 (14·1)
Women receiving at least one valid visit n(%)	691 (99·3)	657 (94·3)	599 (85·9)
Valid visits received			
Median (25th to 75th centile)	10 (8 to 12)	19 (15 to 22)	13 (10 to 16)
Mean (SD)	9·74 (3·42)	18·7 (5·97)	13·28 (5·46)
Reaching fidelity goal of: n(%)			
80%+	401/697 (57·5)	-	-
65%+	-	364/697 (52·2)	-
60%+	-	-	297/697 (42·6)

[1] Robling M, Bekkers MJ, Bell K, Butler CC, Cannings-John R, Channon S, et al. Effectiveness of a nurse-led intensive home-visitation programme for first-time teenage mothers (Building Blocks): A pragmatic randomised controlled trial. Lancet. 2016;387:146–55. [accessed 2019 Nov 12]. Available from: http://www.thelancet.com/journals/lancet/article/PIIS0140-6736(15)00392-X/fulltext

For peer review only - http://bmjopen.bmj.com/site/about/guidelines.xhtml

II. Study assessment domain, outcomes and principal data providers

Table S2 BB:2-6 Study assessment domains, key and exploratory outcomes and principal data providers

Domains	Outcomes	Maternal	Child	HES	ONS	NPD	Data source	Analysis
Primary: Child in Need status recorded at any time during the follow-up period.	CIN status		✓			✓	CIN	LO
	Exploratory:							
	Age at first CIN referral		✓			✓	CIN	*SURV*
	Number of CIN referrals per child		✓			✓	CIN	*PO*
Secondary: (i) Objective measures of maltreatment	CIN categorisation		✓			✓	CIN	MNOM
	CIN duration		✓			✓	CIN	SURV
	Referral to Children's social care services		✓			✓	CIN	LO
	Exploratory: Age at first referral		✓			✓	CIN	SURV
	Child Protection Plan		✓			✓	CIN	LO
	Child Protection Plan categorisation		✓			✓	CIN	MNOM
	Looked after status		✓			✓	CLA	LO
	CLA period of care		✓			✓	CLA	PO
	Legal status of CLA		✓			✓	CLA	D
	Cause of death		✓		✓		ONS- deaths	D
(ii) Associated measures of maltreatment	Injuries and ingestions:							
	Emergency attendance		✓	✓			A&E	LO
	Admission to hospital		✓	✓			IP	LO
	Attendance and/or admission		✓	✓			A&E, IP	LO
	DNA appointments		✓	✓			OP	LO
	Exploratory:							

For peer review only

Domains	Outcomes	Maternal	Child	HES	ONS	NPD	Data source	Analysis
	Number of admissions per child		✓	✓			IP	PO
	Length of hospital admission stay		✓	✓			IP	D
(iii) Maternal outcomes	Subsequent pregnancy	✓		✓			OP, IP, Ab	LO
	Exploratory:							
	Number of births	✓		✓			IP	PO
	Inter-birth interval between first and second child	✓		✓			IP	SURV
(iv) Costs	Health and Social Care resource use	✓	✓	✓		✓	OP, IP, A&E matched with NHS reference costs	D
(v) Child health, developmental and educational outcomes	Special educational needs provision		✓<br>✓	✓		✓	PLASC, AP, PRU Census, EYC	LO
	Disability		✓			✓	NA	NA
	Early education attendance		✓			✓	PLASC, EYC	LO
	School attendance		✓			✓	Absence	LO
	Early Years assessment		✓			✓	EYFSP	LIN, LO
	KS1 attainment					✓	KS1	LO
	Exploratory:							
	Early education attendance by School or an Ofsted registered PVI establishment		✓			✓	PLASC, EYC	D
	Earliest type of day care		✓			✓	EYC	D

Ab - Abortions; A&E – Accident and Emergency; AP – Alternative Provision; CIN – Child in Need; CLA – Child Looked After; D – Descriptive; DNA – Did not attend; EYC – Early Years Census; EYFSP –Early years foundation stage profile; FNP – Family Nurse Partnership; HES – Hospital Episode Statistics; IP - Inpatients; KS1 – Key Stage 1; LIN – Linear regression; LO – Logistic; MNOM - multinomial NA - Not available; NPD – National Pupil Database; ONS – Office for National Statistics; OP – Outpatients; PLASC – Pupil Level and School Census; PO-Poisson; PRU – Pupil Referral Unit Census.; PVI – Private, Voluntary or Independent (PVI) establishment; SURV – Survival.

For peer review only - http://bmjopen.bmj.com/site/about/guidelines.xhtml

III. Data accrued from providers

Table S3 Numbers (%) of mothers and children with data returned by dataset

	Mothers	Children
**Sent for matching to DfE and NHSD**	**N=1537**	**N=1547**
**Department for Education (DfE)**		
Not matched	1248 (81·2)	40 (2·6)
Match to any DfE dataset	289 (18·8)	1507 (97·4)
Pupil Level Annual School Census (PLASC)	<5	1498 (96·8)
Child in Need (CIN)	278 (18·1)	414 (26·8)
Children looked after (CLA)	61 (4·0)	52 (3·4)
Early years foundation stage profile (EYFSP)	N/A	1476 (95·4)
Key Stage one (KS1)	N/A	1472 (95·2)
Early Years Census (EYC)	N/A	642 (41·5)
Pupil referral unit (PRU) Census	<5	0
Alternative Provision (AP)	<5	<5
Absence	<5	1494 (96·6)
**NHS Digital (NHSD)**		
Not matched	36 (2·3)	27 (1·7)
Duplicate match	0	1 (0·06)
Matched to any NHSD dataset	1501 (97·7)	1519 (98·3)
Accident and Emergency (A&E)	1360 (88·5)	1392 (90·0)
Admitted Patient Care (Inpatients) (APC)	1498 (97·5)	1507 (97·4)
Outpatient (OP)	1495 (97·3)	1189 (76·9)
**Sent for matching to Department of Health and Social Care (DHSC)**	**N=1532**	**0**
Abortions dataset	1532 (100)	NA

For peer review only - http://bmjopen.bmj.com/site/about/guidelines.xhtml

IV. Further details of Child in Need outcome.

In our primary comparative analysis we examined whether the firstborn ('BB:0-2 child') had ever been referred to social services and classed as a child in need at any point between birth and 6 years of age. There is no flag in the Child In Need dataset to identify whether a child is in need after they have been referred to social services. Therefore, CIN was defined (as per DfE methodology)[1] as all children referred to social services, excluding cases where:

- the referral is flagged as being a referral resulting in no further action; or
- the only activity recorded is an assessment and the reason for the closure of the case being that case was closed after assessment with no further action.

---

1. Department for Education. Characteristics of children in need in England: 2014 to 2015. 2015. [accessed 2019 Nov 12]. Available from: https://www.gov.uk/government/uploads/system/uploads/attachment_data/file/469740/SFR41-2015_Methodology.pdf

V. Representativeness of study sample

Scope and purpose: We assessed whether the study sample for BB:2-6 were similar to women recruited to the original BB:0-2 trial.

Conclusion: The BB:2-6 and BB:0-2 cohorts are similar in terms of maternal (Table S4) and baby characteristics (Table S5).

Table S4 Maternal characteristics of those in BB:0-2 compared to those in the BB:2-6

	**BB:0-2 N=1618**	**BB:2-6 N=1537**
**Minimisation variables at randomisation**		
**Gestational age at baseline** n(%)		
16 weeks or more	658 (40·7)	636 (41·4)
Less than 16 weeks	959 (59·3)	900 (58·6)
**Smoking status** n(%)		
No	860 (53·2)	822 (53·5)
Yes	758 (46·8)	715 (46·5)
**Language** n(%)		
English	1611 (99·6)	1530 (99·5)
Other	6 (0·4)	6 (0·4)
Missing	*1 (0·0)*	*0 (0·0)*

For peer review only - http://bmjopen.bmj.com/site/about/guidelines.xhtml

	BB:0-2 N=1618	BB:2-6 N=1537
**Trial arm** n(%)		
FNP	808 (49·9)	766 (49·8)
Usual care	810 (50·1)	771 (50·2)
**Age at recruitment (years) Median (25th to 75th centile)**	17·9 (16·9 to 18·8)	17·9 (16·9 to 18·8)
**Ethnicity** n(%)		
White background	1425 (88·1)	1355 (88·2)
Mixed background	89 (5·5)	82 (5·3)
Asian background	27 (1·7)	25 (1·6)
Black background	71 (4·4)	69 (4·5)
Chinese or Other background	6 (0·4)	6 (0·4)
**Relationship status with baby's father** n(%)		
Married	20 (1·2)	16 (1·0)
Separated	165 (10·2)	150 (10·0)
Closely involved/boyfriend	1222 (75·5)	1168 (76·0)
Just friends	211 (13·0)	203 (13·2)
**Live with father of baby** n(%)		
Yes	368 (22·7)	348 (22·6)
No	1112 (68·7)	1061 (69·0)
Missing	138 (8·5)	128 (8·3)
**NEET status**[a] n(%)		
Yes	663 (41·0)	634 (41·2)
No	717 (44·3)	678 (44·1)
Missing	*4 (0·2)*	*4 (0·3)*
Not applicable (aged 16 years or younger)	*234 (14·5)*	*221 (14·4)*
**Socio-economic status: Overall Index of Multiple Deprivation (IMD) Score**[b] Median (25th to 75th centile)	N=1606 38·2 (25·2 to 52·0)	N=1525 38·1 (25·0 to 52·1)
**Maternal health and wellbeing**		
**Generalized self-efficacy scale (score 10 to 40)**[c] Median (25th to 75th centile)	N=1592 30·0 (28·0 to 33·0)	N=1533 30·0 (28·0 to 33·0)
**Adaptive functioning: Difficulty in at least one basic skill** n(%)		
Yes	430 (26·6)	397 (25·8)
No	1185 (73·2)	1138 (74·0)
Missing	*3 (0·2)*	*2 (0·1)*
**Adaptive functioning: Had 3 or less life skills** n(%)		
Yes	434 (26·8)	403 (26·2)
No	1178 (72·8)	1129 (73·5)
Missing	*6 (0·4)*	*5 (0·3)*
**Adaptive functioning: At least one burden** n(%)		
Yes	476 (29·4)	451 (29·3)
No	1131 (69·9)	1075 (69·9)
Missing	*11 (0·7)*	*11 (0·7)*
**Smoking** (Participant self-reported) n(%)		
Ever smoked: Yes	1291 (79·8)	1227 (79·8)
No	327 (20·0)	310 (20·2)
Missing	*0 (0)*	*0 (0)*
**Child characteristics**	**BB:0-2 sample N=1510**	**BB:2-6 sample N=1547**
**Trial arm** n(%)		

For peer review only - http://bmjopen.bmj.com/site/about/guidelines.xhtml

	**BB:0-2 N=1618**	**BB:2-6 N=1537**
FNP	742 (49·1)	773 (50·0)
Usual Care	768 (50·9)	774 (50·0)
**Gender** n(%)		
Male	777 (51·5)	787 (50·9)
Female	732 (48·5)	758 (40·1)
Missing	1	72[d]
**Birthweight (grams) Mean (sd)**	3207·3 (599·6)	3219·6 (580·7)
Missing[c]		71
**Neonatal unit (NNU) admission** n(%)		
Yes	149 (10·0)	142 (9·2)
Missing from data collection	13	11
Missing	Not applicable	71[d]

[a] *Definition of NEET status: Not in education employment or training (applicable only to those whose age at end of previous academic year at time of baseline interview was >16)*
[b] *Higher IMD score indicated more deprivation. Mean IMD Score for England in 2010 was 21·67 [60];*
[c] *Higher score indicates higher level of self-efficacy*
[d] *Mothers were withdrawn in BB:0-2 but are included in BB:2-6*

Table S5 Child characteristics of those in the BB:0-2 study compared to those in the BB:2-6 study

	**BB:0-2 sample N=1510**	**BB:2-6 sample N=1547**
**Trial arm** n(%)		
FNP	742 (49·1)	773 (50·0)
Usual Care	768 (50·9)	774 (50·0)
**Gender** n(%)		
Male	777 (51·5)	787 (50·9)
Female	732 (48·5)	758 (40·1)
Missing	1	72[a]
**Birthweight (grams) Mean (sd)**	3207·3 (599·6)	3219·6 (580·7)
Missing[a]		71
**Neonatal unit (NNU) admission** n(%)		
Yes	149 (10·0)	142 (9·2)
Missing from data collection	13	11
Missing[a]	Not applicable	71*

[a] *Mothers were withdrawn in BB:0-2 but are included in BB:2-6*

For peer review only - http://bmjopen.bmj.com/site/about/guidelines.xhtml

VI. Descriptive secondary outcomes and additional Key Stage 1 results

Table S6 Child In Need referrals, Children's Social Services referrals and hospital admissions: descriptives

Outcome	FNP	Usual care (UC)
**Child in Need (CIN) Referrals**	***N=161***	***N=162***
Age at first CIN referral (days)		
Pre birth to < 1year	12 (7.5%)	5 (3.1%)
1 to <2 years	6 (3·7%)	6 (3·7%)
2 to <3 years	15 (9·4%)	28 (17·3%)
3 to <4 years	27 (16·9%)	23 (14·2%)
4 to <5 years	47 (29·4%)	39 (24·1%)
5 to <6 years	33 (20·6%)	41 (25·3%)
6 to <7 years	20 (12·5%)	20 (12·3%)
Missing	*1*	*0*
Unique* number of CIN referrals per child		
1 case	110 (68·3%)	110 (67·9%)
2 cases	42 (26·1%)	42 (25·9%)
3 or more cases	9 (5·5%)	10 (6·1%)
CIN cases closed at 31st March 2017	116 (72·0%)	110 (67·9%)
**Referral to Children's social care services (CSCS)**	***N=760***	***N=746***
Total referrals made N	339	354
Ratio referrals:children	1·65	1·70
Referrals made per child	***N=206***	***N=208***
1	159 (77·2%)	140 (67·3%)
2	34 (16·5%)	51 (24·5%)
3 to 5	13 (6·3%)	27 (8·2%)
**Unit=referral (referred to CSCS encompass any referral)**	***n=339***	***n=354***
Source of referral		
Individual	29 (8·8%)	34 (10·0%)
School/Education	150 (45·7%)	136 (40·0%)
Health services	114 (34·8%)	132 (38·8%)
Local Authority Services	35 (10·7%)	38 (11·2%)
Missing	*11*	*14*
Primary need of referrals		
Abuse or neglect	198 (58·9%)	205 (57·9%)
Family dysfunction	72 (21·4%)	88 (24·9%)
Family in acute stress	19 (5·7%)	21 (5·9%)
Low income/absent parenting/ socially unacceptable behaviour	11 (3·3%)	7 (2·0%)
Parental/Childs disability illness	14 (4·2%)	11 (3·1%)
Cases other than Child in Need	11 (2·3%)	12 (3·4%)
Not stated	11 (3·3%)	9 (2·5%)
Missing	*3*	*1*
Legal status of child looked after†	***N=17***	***N=23***
Interim care order	6 (35·3%)	8 (34·8%)
Full care order	5 (29·4%)	6 (26·1%)

For peer review only - http://bmjopen.bmj.com/site/about/guidelines.xhtml

Outcome	FNP	Usual care (UC)
Placement order granted	<5	<5
Single period of accommodation under section 20	<5	<5
**Injuries and Ingestions: Admission to hospital**	**N=760**	**N=759**
N unique admissions	109	119
Ratio admissions: children	1.22	1.20
Length of hospital stay (days)	0·5‡ (0·5 to 1·0)	0·5 (0·5 to 1·0)
	**N=760**	**N=746**
Death	<10	

Data are n (%), n/N (%), or median (25th to 75th centile) unless otherwise stated. <5 = numbers suppressed· FNP = Family Nurse Partnership Programme + Usual Care. * A unique referral is counted as a distinct referral date per child. † Legal status for most recent CLA episode (Of n=52 children with a recent CLA episode, n=12 cases have a missing status). ‡ 0·5 days indicates an admission and discharge on the same day (could be up to 1 day in hospital). Source: National Pupil Database, The Department for Education

For peer review only

Table S7 Emergency attendance to hospital by injury or ingestion, by trial arm

	**Trial arm, n (%)**	
**Injury / ingestion**	**FNP (N = 911)**	**Usual Care (N = 881)**
Laceration	136 (14.9)	158 (17.9)
Contusion/abrasion	90 (9.9)	96 (10.9)
Soft-tissue inflammation	63 (6.9)	64 (7.3)
Head injury	68 (29.4)	249 (28.3)
Dislocation	109 (12.0)	84 (9.5)
Sprain/muscle/tendon injury	35 (3.8)	33 (3.8)
Nerve/vascular injury	74 (8.1)	60 (6.8)
Burns and scalds/electric shock	48 (5.3)	37 (4.2)
Foreign body	40 (4.4) 4	6 (5.2)
Bites and stings	12 (1.3)	20 (2.3)
Poisoning	36 (4.0)	34 (3.9)

For peer review only - http://bmjopen.bmj.com/site/about/guidelines.xhtml

Table S8 **Admissions by injury/ingestion type and trial arm: For children of all ages (0-6 years) and for children aged 0-2 years old**

	**Trial arm, n (%)**			
	**All children**		**Children aged 0-2 years***	
**Diagnosis ICD-10 code**	**FNP**<br><br>**(n=109)**	**Usual care**<br><br>**(n=119)**	**FNP**<br><br>**(n=42)**	**Usual Care**<br><br>**(n=63)**
S00–S09 Injuries to the head	52 (47.7)	61 (51.3)	39 (61.9)	27 (64.3)
S20–S29 Injuries to the thorax	9 (8.3)	6 (5.0)		
S30–S39 Injuries to the abdomen, lower back, lumbar spine and pelvis				
S40–S49 Injuries to the shoulder and upper arm			7 (16.7)	7 (11.1)
S50–S59 Injuries to the elbow and forearm				
S60–S69 Injuries to the wrist and hand	8 (7.3)	5 (4.2)		
S70–S79 Injuries to the hip and thigh	11 (10.1)	11 (9.2)		
S80–S89 Injuries to the knee and lower leg				
S90–S99 Injuries to the ankle and foot				
T00–T07 Injuries involving multiple body regions	5 (4.6)	10 (8.4)	15 (35.7)	7 (11.1)
T08–T14 Injuries to unspecified part of trunk, limb or body region				
T20–T32 Burns and corrosions				
T15–T19 Effects of foreign body entering through natural orifice	5 (4.6)	8 (6.7)		
T36–T50 Poisoning by drugs, medicaments and biological substances	11 (10.1)	8 (6.7)		
T51–T65 Toxic effects of substances chiefly non-medicinal as to source (sting, alcohol, solvents etc.)	8 (7.3)	10 (8.4)		
T66–T78 Other and unspecified effects of external causes				

* We undertook further exploratory analyses of length of stay following admission for:
(i) children aged under 1 year old: The median (25th to 75th quartile) length of stay (in days) was, for the n=20 children in FNP group 0.75 (0.5 to 2) and for the n=29 children in the UC group 0.5 (0.5 to 2) with no difference observed (Incidence rate ratio (IRR) 0.68, 95% CI 0.24 to 1.98, Ref = UC).
(ii) children aged between 1 and under 2 years old: The median (25th to 75th centile) length of stay (in days) was, for the n=21 admissions in FNP group, 0.5 (0.5 to 1) and for the n=34 admissions in UC group 0.5 (0.5 to 1) with no observed difference (IRR 0.36, 95% CI 0.05 to 2.43, Ref = UC).

For peer review only

Table S9. Hospital admissions for injuries and ingestions by trial arm and broken down by age categories

	**FNP N=760**	**Usual care N=759**
No hospital admissions with an injury or an ingestion (0-6 years)	671 (88.3%)	660 (87.0%)
At least one hospital admission with an injury or an ingestion	89 (11.7%)	99 (13.0%)
By age at admission:		
**Under 1 year**		
Children with no admission n(%)	744 (97.9%)	732 (96.4%)
Children with at least one admission n(%)	16 (2.1%)	27 (3.6%)
95% CI	1.3 to 3.3%	2.5 to 5.1%
Total number of admissions (different days)	20	29
Ratio of admissions: child	1.25	1.08
	n=20	n=29
Length of stay (days) Median (25th to 75th centiles)	0.75 (0.5 to 2)	0.5 (0.5 to 2)
Same day discharge (admission = discharge date)	10 (50%)	15 (51.7%)
Discharge after 1 day or more	10 (50%)	14 (48.3%)
**1 to under 2 years**		
Children with no admission n(%)	738 (97.1%)	725 (95.5%)
Children with at least one admission n(%)	21 (2.8%)	31 (4.1%)
95% CI	1.8 to 4.2%	2.9 to 5.7%
Total number of admissions (different days)	22	34
Ratio of admissions: child	1.05	1.10
	n=21*	n=34
Length of stay (days) Median (25th to 75th centiles)	0.5 (0.5 to 1)	0.5 (0.5 to 1)
Same day discharge (admission = discharge date)	14 (66.7%)	23 (67.6%)
Discharge after 1 day or more	7 (33.3%)	11 (32.4%)
**25 months to under 6 years**		
Children with no admission n(%)	699 (92.0%)	709 (93.4%)
Children with at least one admission n(%)	61 (8.0%)	50 (6.6%)
95% CI	6.3 to 10.2%	5.0 to 8.6%
Total number of admissions (different days)	67	56
Ratio of admissions: child	1.10	1.12
	n=65*	n=56
Length of stay (days) Median (25th to 75th centiles)	0.5 (0.5 to 1)	0.5 (0.5 to 1)
Same day discharge (admission = discharge date)	38 (58.4%)	29 (51.8%)
Discharge after 1 day	18 (27.6%)	17 (30.4%)
Discharge after 2 days or more	11 (16.9%)	10 (17.9%)

* Date of discharge missing – LOS cannot be calculated

LOS=0.5 days indicates an admission and discharge on the same day (could be up to 1 day in hospital).

For peer review only - http://bmjopen.bmj.com/site/about/guidelines.xhtml

Table S10 Child health, developmental and educational: descriptives

Outcome	FNP	Usual care	Absolute risk difference (FNP-Usual care) (95% CI)
**Early educational attendance**	**N=759**	**N=747**	
Children attending education* up to the age of 4 years			
Yes†	758 (99·7%)	747 (100·0%)	-0·3% (-1·0 to 0·3%)
Children attending school up to the age of:			
• 4 years†	753 (99·1%)	744 (99·6%)	-0·5% (-1·5 to 0·4%)
• 3 years	479 (63·0%)	482 (64·5%)	
• 2 years	137 (18·0%)	147 (19·7%)	
Earliest type of day care	***N=334***	***N=308***	
Private	217 (65·0%)	221 (71·8%)	
Voluntary	52 (15·6%)	57 (18·5%)	
Local Authority Day Nursery	36 (10·8%)	22 (7·1%)	
Other‡	29 (8·7%)	8 (2·6%)	
**School attendance**	**N=754**	**N=740**	
Number of academic sessions§	630 (264 to 638)	630 (262 to 638)	
Overall absence rate Median (25th to 75th centiles)	5·08 (2·69 to 8·73)	5·19 (2·73 to 8·41)	
Overall authorised absence rate Median (25th to 75th centiles)	3·50 (1·96 to 6·09)	3·79 (2·02 to 6·11)	
Overall unauthorised absence rate Median (25th to 75th centiles)	1·84 (0·76 to 4·34)	1·57 (0·63 to 3·88)	
**Early Years assessment**	**N=743**	**N=728**	
Achieving good level of development (GLD) ‖:			
• Communication and Language	642 (86·4%)	613 (84·2%)	2·2% (-1·4 to 5·8%)
• Physical Development	676 (91·0%)	643 (88·3%)	2·7% (-0·5 to 5·8%)

For peer review only - http://bmjopen.bmj.com/site/about/guidelines.xhtml

Outcome	FNP	Usual care	Absolute risk difference (FNP-Usual care) (95% CI)
• Personal, Social and Emotional Development	668 (89·9%)	634 (87·1%)	2·8% (-0·4 to 6·1%)
• Literacy	493 (66·4%)	458 (62·9%)	3·4% (-1·4 to 8·3%)
• Maths	545 (73·4%)	516 (70·9%)	2·5% (-2·1 to 7·0%)
Total point score¶			
• Score <34	325 (43·8%)	345 (47·4%)	
• Score = 34	187 (25·2%)	183 (25·1%)	
• Score >34	231 (31·1%)	200 (27·5%)	
**Key Stage 1 assessments**			
Writing academic year 2017/18	**N=242**	**N=245**	
Lower than expected	82 (33·9%)	81 (33·1%)	
Reaching at least the expected standard**	160 (66·1%)	164 (66·9%)	-0·8 (-9·2 to 7·5)
Expected standard	140 (57·9%)	132 (53·9%)	4·0 (-4·8 to 12·7)
Higher standard	20 (8·3%)	32 (13·1%)	-4·8 (-10·4 to 0·7)

Data are n (%), n/N (%), or median (25th to 75th centile) unless otherwise stated. FNP = Family Nurse Partnership Programme + Usual Care.

* School or an Ofsted registered Private, Voluntary or Independent (PVI) establishment;

† = Not estimable; ‡ Childminding Network, Childminder, Registered Independent School, Other; § Total number of sessions over the academic years available (2013 to Spring 2017) and all 6 terms per academic year. ‖ Children achieving a good level of development are those achieving at least the expected level within the prime and specific areas of learning; ¶ Total point score ranges from 17 to 51 with a higher score indicating a better level of development. ** Working at the expected standard and at a greater depth within the expected standard. PLASC (Autumn, Spring and Summer terms), AP, PRU Census, and EYC. If for any of the years no response was recorded then the assumption was that the child was not included in the denominator as either not present on Census day or not in school. Source: National Pupil Database, The Department for Education

For peer review only - http://bmjopen.bmj.com/site/about/guidelines.xhtml

Table S11 Children reaching the expected standard in KS1 assessments by trial arm and academic year

		Academic year 2016/17	Academic year 2017/18
Reading	FNP	302/498 (60.6%)	181/242 (74.8%)
	Usual care	263/487 (54.0%)	180/245 (73.5%)
Maths	FNP	280/498 (56.2%)	179/242 (74.0%)
	Usual care	263/487 (54.0%)	186/245 (75.9%)
Science	FNP	341/498 (68.5%)	196/242 (81.0%)
	Usual care	310/487 (63.7%)	203/245 (82.9%)

Source: National Pupil Database, The Department for Education

Figure S1 Distribution of children's month of birth by trial arm

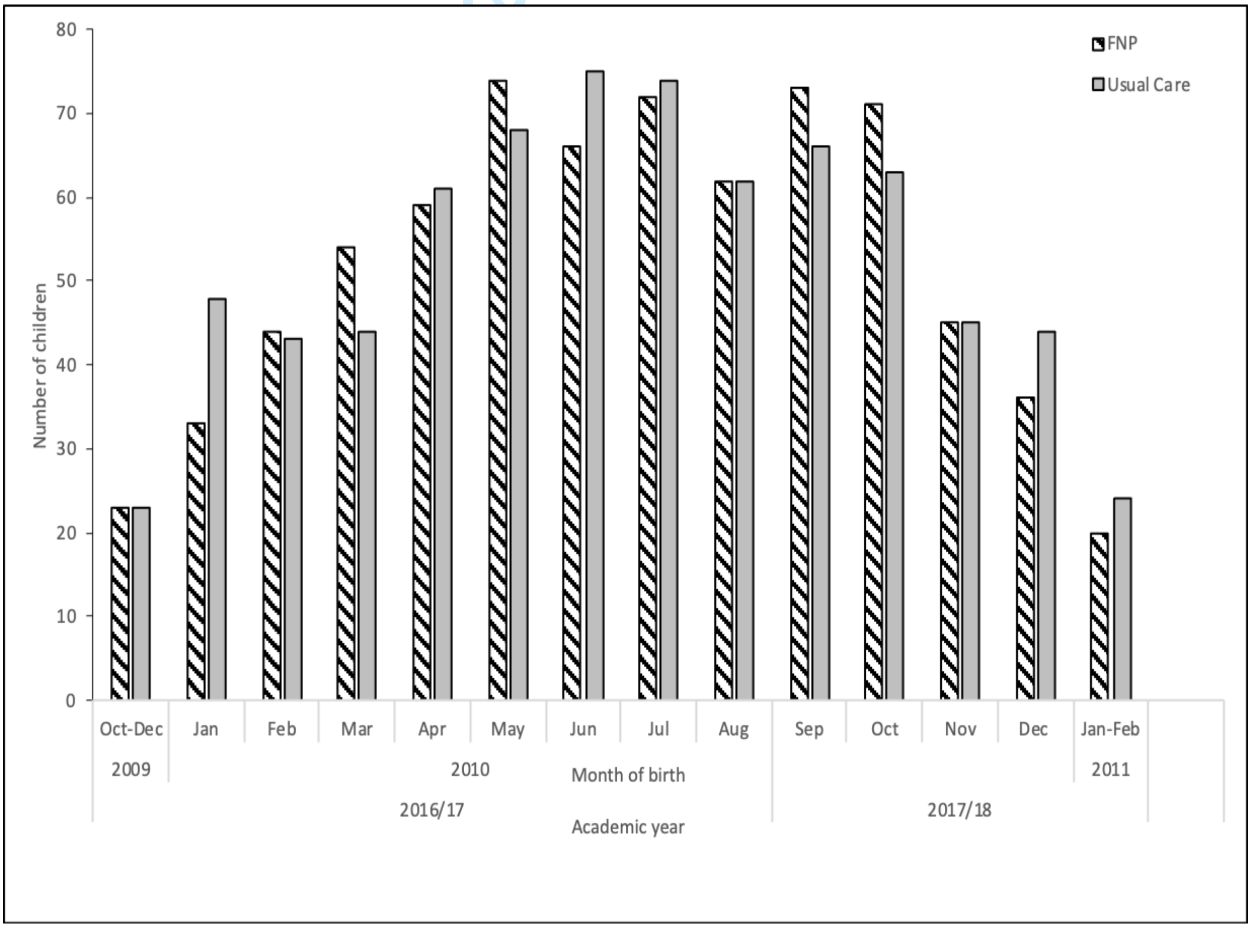

For peer review only - http://bmjopen.bmj.com/site/about/guidelines.xhtml

Table S12 Comparison of parameter estimates before and after adjustment for child's month[a] of birth

	Odds ratio[b] (95% CI), p-values	
	Adjusted for site and minimisation variables[c]	Adjusted for site, minimisation variables[c] and month of birth
**Key Stage 1 outcome**		
**Reading**		
Reaching at least the expected standard	1.23 (0.99 to 1.53), 0.051	1.26 (1.02 to 1.57), 0.035
Expected standard	*1.24 (0.99 to 1.56), 0.056*	*1.27 (1.01 to 1.59), 0.042*
Higher standard	*1.20 (0.88 to 1.65), 0.250*	*1.26 (0.91 to 1.75), 0.161*
**Maths**		
Reaching at least the expected standard	1.04 (0.84 to 1.28), 0.731	1.06 (0.85 to 1.31), 0.613
Expected standard	*1.06 (0.85 to 1.32), 0.611*	*1.07 (0.86 to 1.34), 0.527*
Higher standard	*0.93 (0.64 to 1.35), 0.711*	*0.93 (0.74 to 1.17), 0.522*
**Science**		
Reaching at least the expected standard	1.14 (0.91 to 1.43), 0.254	1.16 (0.92 to 1.47), 0.197
**Writing academic year 2017/18**		
Reaching at least the expected standard	1.24 (0.97 to 1.60), 0.090	1.26 (0.98 to 1.62), 0.076
Expected standard	*1.29 (1.00 to 1.68), 0.054*	*1.30 (1.00 to 1.69), 0.050*
Higher standard	*0.92 (0.51 to 1.64), 0.769*	*0.97 (0.54 to 1.75), 0.917*
**Early Years Assessment**		
Achieving GLD in all five areas of learning[d]	1.26 (1.03 to 1.55), 0.026	1.31 (1.05 to 1.62), 0.015
Achieving GLD in all 17 early learning goals	1.24 (1.01 to 1.52), 0.043	1.27 (1.03 to 1.58), 0.027
Total point score	0.65 (-0.11 to 1.41), 0.094	0.70 (-0.03 to 1.42), 0.060

a Quarter of birth runs from September to November, December to February, March to May, June to August; b Odds ratio represents the FNP arm compared to Usual care; c gestational age, smoking status at recruitment, and first or preferred language; d Children achieving a good level of development (GLD) are those achieving at least the expected level within the prime and specific areas of learning.

Source: National Pupil Database, The Department for Education

For peer review only

VII. Results of planned sub-group analysis

No differential effects due to maternal age, deprivation, participation in employment, education or training, or basic life skills were found in planned sub-group analyses for any of the objective maltreatment outcomes (Table S10 and Table S11). The beneficial impact of FNP on EYFSP total point score was mostly observed for children with mothers aged <16 years rather than those aged ≥16 at study entry but no other differences by study sub-group were found (Table S12).

The differential effects due to maternal age, deprivation, participation in employment, education or training, or basic life skills for KS1 reading, maths and science are displayed in Tables S13a to e and for writing in Table S14. At KS1, sex differences were large with girls outperforming boys in all four subject areas (Table S13a). The intervention effect was greater for boys than girls with a higher proportion reaching the expected standard in the FNP arm, particularly for reading and writing (and strengthened after adjusting for month of birth) and for the writing assessment (academic year 2016/17) this interaction reached statistical significance after adjusting for month of birth (post-hoc analysis). When examining how the intervention effects differed in mothers aged less than 16 years at recruitment compared to mothers aged 16 years or more in each KS1 assessment, differences were observed in maths (Table S13b) and writing (Table S14), with a larger effect seen in mothers aged less than 16 at recruitment, when compared to those aged 16 or more.

Table S13 Subgroup analyses for referrals to Children's social care services

N(%) referred	FNP	Usual care	Adjusted[a] OR (95% CI), p-value	Interaction p-value
**Child Gender**				
Boys	99 (26.3)	108 (27.7)	0.93 (0.65 to 1.33), 0.69	0.78
Girls	107 (27.9)	100 (28.1)	1.01 (0.69 to 1.48), 0.94	
**Maternal age at recruitment**				
<16 years	17 (34.7)	19 (34.6)	1.05 (0.44 to 2.46), 0.92	0.96
≥16 years	189 (26.6)	189 (27.4)	0.96 (0.72 to 1.30), 0.81	
**Adaptive functioning**				
No difficulty	140 (25.4)	149 (26.3)	0.99 (0.71 to 1.38), 0.96	0.67
Difficulty in basic skills	66 (31.7)	59 (33.0)	0.81 (0.59 to 1.41), 0.67	
**NEET status**				
EET	70 (20.3)	75 (23.1)	0.83 (0.54 to 1.29), 0.42	0.18
NEET	100 (32.5)	89 (29.2)	1.19 (0.80 to 1.78), 0.39	
**Deprivation quintile**				

For peer review only - http://bmjopen.bmj.com/site/about/guidelines.xhtml

1 – least deprivation	33 (20.4)	34 (23.9)	0.77 (0.44 to 1.36), 0.37	0.32
2	36 (23.8)	30 (20.5)	1.26 (0.61 to 2.60), 0.53	
3	42 (28.8)	43 (27.9)	0.96 (0.50 to 1.86), 0.91	
4	41 (31.1)	39 (25.3)	1.27 (0.69 to 2.31), 0.44	
5 – most deprivation	54 (33.3)	61 (42.4)	0.65 (0.35 to 1.22), 0.18	

[a] FNP compared to usual care. Analysis adjusted for stratification (site), minimisation variables (gestational age, smoking status at recruitment, and first or preferred language). N/EET – Not/In Education, Employment or Training (Source: National Pupil Database, The Department for Education)

Table S14 Subgroup analyses for child in need status

**N(%) CIN**	**FNP**	**Usual care**	**Adjusted[a] OR (95% CI), p-value**	**Interaction p-value**
**Gender**				
Boys	80 (21.3)	80 (20.5)	1.05 (0.72 to 1.53), 0.81	0.56
Girls	81 (21.1)	82 (23.0)	0.91 (0.60 to 1.38), 0.65	
**Maternal age at recruitment**				
<16 years	14 (28.6)	16 (29.1)	1.00 (0.41 to 2.43), 0.99	0.98
≥16 years	147 (20.7)	146 (21.1)	0.98 (0.72 to 1.33), 0.88	
**Adaptive functioning**				
No difficulty	106 (19.2)	113 (20.0)	0.99 (0.70 to 1.42), 0.98	0.76
Difficulty in basic skills	55 (26.4)	49 (27.4)	0.93 (0.59 to 1.47), 0.76	
**NEET**				
EET	52 (16.0)	52 (15.1)	0.93 (0.57 to 1.53), 0.78	0.42
NEET	81 (26.3)	72 (23.6)	1.18 (0.77 to 1.77), 0.46	
**Deprivation quintile**				
1 – least deprivation	24 (14.8)	22 (15.5)	0.92 (0.49 to 1.73), 0.79	0.62
2	30 (19.9)	22 (15.1)	1.53 (0.68 to 3.47), 0.31	
3	32 (21.9)	36 (23,4)	0.89 (0.52 to 1.54), 0.68	
4	32 (24.2)	34 (22.1)	1.15 (0.64 to 2.06), 0.64	
5 – most deprivation	43 (26.5)	48 (33.3)	0.72 (0.38 to 1.38), 0.32	

[a] FNP compared to usual care. Analysis adjusted for stratification (site), minimisation variables (gestational age, smoking status at recruitment, and first or preferred language). N/EET – Not/In Education, Employment or Training (Source: National Pupil Database, The Department for Education).

For peer review only

For peer review only - http://bmjopen.bmj.com/site/about/guidelines.xhtml

Table S15 Subgroup analyses for Early Years Assessment total point score

Mean (SD) total point score	FNP	Usual care	Adjusted[a] mean difference (95% CI), p-value	Interaction p-value
**Gender**				
Boys	N=369<br>30.57 (7.33)	N=383<br>29.84 (7.56)	0.75 (-0.31 to 1.82), 0.17	0.57
Girls	N=374<br>33.85 (6.79)	N=345<br>33.53 (7.22)	0.34 (-0.67 to 1.35), 0.51	
**Maternal age at recruitment**				
<16 years	N=45<br>32.44 (6.71)	N=52<br>28.91 (7.70)	3.65 (0.76 to 6.55), 0.013	0.046
≥16 years	N= 698<br>32.21 (7.28)	N= 676<br>31.80 (7.58)	0.42 (-0.36 to 1.21), 0.29	
**Adaptive functioning**				
No difficulty	N=540<br>32.99 (7.12)	N=552<br>32.11 (7.65)	0.88 (0.003 to 1.75), 0.049	0.50
Difficulty in basic skills	N=203<br>30.18 (7.20)	N=175<br>29.94 (7.33)	0.34 (-1.11 to 1.79), 0.65	
**NEET status**				
In EET	N=339<br>32.45 (7.12)	N=322<br>32.37 (7.68)	0.15 (-0.99 to 1.28), 0.80	0.95
NEET	N= 304<br>31.72 (7.57)	N=299<br>31.50 (7.43)	0.19 (-1.00 to 1.38), 0.76	
**Index of Multiple Deprivation quintiles**				
1 = least deprived	N=159<br>33.16 (7.44)	N=139<br>31.53 (8.24)	1.72 (-0.07 to 3.50), 0.060	ref
2	N=148<br>32.16 (6.97)	N=144<br>31.59 (7.05)	0.65 (-0.96 to 2.27), 0.43	0.84
3	N=146<br>32.16 (6.79)	N=151<br>32.59 (7.27)	-0.20 (-1.79 to 1.39), 0.80	0.21
4	N=128<br>30.95 (7.53)	N=148<br>31.11 (7.50)	-0.31 (-2.11 to 1.48), 0.73	0.85
5 = most deprived	N=156<br>32.41 (7.46)	N=140<br>31.03 (8.12)	1.45 (-0.33 to 3.22), 0.11	0.74

[a] FNP compared to usual care. Analysis adjusted for stratification (site), minimisation variables (gestational age, smoking status at recruitment, and first or preferred language). N/EET – Not/In Education, Employment or Training (Source: National Pupil Database, The Department for Education).

Table S16 Subgroup analyses for KS1 assessments reaching the expected standard (RES)

(a) Gender

N(%)	Boys		Girls	
	Not RES	RES	Not RES	RES
**Reading**				
FNP	152 (41.8)	212 (58.2)	105 (27.9)	271 (72.1)
Usual care	182 (47.3)	203 (52.7)	107 (30.8)	240 (69.2)
Adjusted[a] OR (95% CI), p-value	1.27 (0.95 to 1.69), 0.11		1.16 (0.84 to 1.59), 0.38	
Interaction p-value=0.68				

For peer review only - http://bmjopen.bmj.com/site/about/guidelines.xhtml

Adjusted[b] OR (95% CI), p-value	1.30 (0.96 to 1.75), 0.09		1.17 (0.84 to 1.62), 0.36	
Interaction p-value=0.68				
**Maths**				
FNP	149 (40.9)	215 (59.1)	132 (35.1)	244 (64.9)
Usual care	166 (43.1)	219 (56.9)	117 (33.7)	230 (66.3)
Adjusted[a] OR (95% CI), p-value	1.11 (0.83 to 1.49), 0.49		0.95 (0.69 to 1.29), 0.72*	
Interaction p-value=0.46				
Adjusted[b] OR (95% CI), p-value	1.14 (0.84 to 1.54), 0.40		0.94 (0.69 to 1.29), 0.71	
Interaction p-value=0.47				
**Science**				
FNP	120 (33.0)	244 (67.0)	83 (22.1)	293 (77.9)
Usual care	138 (35.8)	247 (64.2)	81 (23.3)	266 (76.7)
Adjusted[a] OR (95% CI), p-value	1.16 (0.86 to 1.58), 0.34		1.08 (0.76 to 1.53), 0.68	
Interaction p-value=0.77				
Adjusted[b] OR (95% CI), p-value	1.18 (0.87 to 1.63), 0.28		1.08 (0.76 to 1.55), 0.66	
Interaction p-value=0.72				

RES = Reaching the expected standard; [a] Analysis adjusted for stratification (site), minimisation variables (gestational age, smoking status at recruitment, and first or preferred language); [b] Analysis adjusted for stratification (site), minimisation variables (gestational age, smoking status at recruitment, and first or preferred language) and month of birth. (Source: National Pupil Database, The Department for Education).

(b) Maternal age at recruitment

N(%)	Maternal age<16 years		Maternal age≥16 years	
	**Not RES**	**RES**	**Not RES**	**RES**
**Reading**				
FNP	21 (44.7)	26 (55.3)	236 (34.1)	457 (65.9)
Usual care	29 (52.7)	26 (47.3)	260 (38.4)	417 (61.6)
Adjusted[a] OR (95% CI), p-value	1.21 (0.97 to 1.52), 0.09		1.49 (0.65 to 3.41), 0.34	
Interaction p-value=p=0.66				
Adjusted[b] OR (95% CI), p-value	1.50 (0.65 to 3.48), 0.34		1.25 (0.99 to 1.57), 0.07	
Interaction p-value=0.62				
**Maths**				
FNP	16 (34.0)	31 (66.0)	265 (38.2)	428 (61.8)
Usual care	32 (58.2)	23 (41.8)	251 (37.1)	426 (62.9)
Adjusted[a] OR (95% CI), p-value	3.15 (0.94 to 10.48), 0.06		0.96 (0.77 to 1.19), 0.69	
Interaction p-value=0.011				

For peer review only

For peer review only - http://bmjopen.bmj.com/site/about/guidelines.xhtml

Adjusted[b] OR (95% CI), p-value	3.23 (1.36 to 7.67), 0.008		0.98 (0.78 to 1.22), 0.83	
Interaction p-value=0.014				
**Science**				
FNP	18 (38.3)	29 (61.7)	185 (26.7)	508 (73.3)
Usual care	25 (45.4)	30 (54.6)	194 (28.7)	483 (71.3)
Adjusted[a] OR (95% CI), p-value	1.54 (0.53 to 4.47), 0.42		1.11 (0.88 to 1.41), 0.39	
Interaction p-value=0.56				
Adjusted[b] OR (95% CI), p-value	1.37 (0.59 to 3.19), 0.467		1.14 (0.89 to 1.45), 0.29	
Interaction p-value=0.69				

RES = Reaching the expected standard; [a] FNP compared to Usual care. Analysis adjusted for stratification (site), minimisation variables (gestational age, smoking status at recruitment, and first or preferred language); [b] Analysis adjusted for stratification (site), minimisation variables (gestational age, smoking status at recruitment, and first or preferred language) and month of birth. (Source: National Pupil Database, The Department for Education).

(c) Adaptive functioning

N(%)	No difficulty with basic skills		Difficulty with basic skills	
	**Not RES**	**RES**	**Not RES**	**RES**
**Reading**				
FNP	152 (30.5)	347 (69.5)	87 (42.9)	116 (57.1)
Usual care	188 (36.2)	332 (63.8)	84 (48.0)	91 (52.0)
Adjusted[a] OR (95% CI), p-value	1.29 (0.98 to 1.70), 0.07		1.23 (0.82 to 1.86), 0.32	
Interaction p-value=0.61				
Adjusted[b] OR (95% CI), p-value	1.29 (0.98 to 1.69), 0.07		1.25 (0.83 to 1.91), 0.29	
Interaction p-value=0.95				
**Maths**				
FNP	186 (34.6)	351 (65.4)	95 (46.8)	108 (53.2)
Usual care	192 (34.5)	364 (65.5)	91 (52.0)	84 (48.0)
Adjusted[a] OR (95% CI), p-value	1.01 (0.78 to 1.29), 0.97		1.27 (0.84 to 1.93), 0.26	
Interaction p-value=0.38				
Adjusted[b] OR (95% CI), p-value	1.01 (0.78 to 1.31), 0.93		1.32 (0.86 to 2.03), 0.20	
Interaction p-value=0.28				
**Science**				
FNP	130 (24.2)	407 (75.8)	73 (36.0)	130 (64.0)
Usual care	153 (27.5)	403 (72.5)	66 (37.7)	109 (62.3)
Adjusted[a] OR (95% CI), p-value	1.20 (0.92 to 1.58), 0.19		1.10 (0.71 to 1.69), 0.68	
Interaction p-value=0.71				

For peer review only

Adjusted[b] OR (95% CI), p-value	1.22 (0.93 to 1.62), 0.16	1.13 (0.73 to 1.74), 0.59
Interaction p-value=0.82		

RES = Reaching the expected standard; [a] FNP compared to Usual care. Analysis adjusted for stratification (site), minimisation variables (gestational age, smoking status at recruitment, and first or preferred language); [b] Analysis adjusted for stratification (site), minimisation variables (gestational age, smoking status at recruitment, and first or preferred language) and month of birth. (Source: National Pupil Database, The Department for Education).

(d) Not in Education, employment or training (NEET) status

**N(%)**	**NEET**		**In EET**	
	**Not RES**	**RES**	**Not RES**	**RES**
**Reading**				
FNP	105 (31.2)	232 (68.8)	109 (36.0)	194 (64.0)
Usual care	115 (35.8)	206 (61.2)	121 (40.5)	178 (59.5)
Adjusted[a] OR (95% CI), p-value	1.26 (0.91 to 1.75), 0.17		1.21 (0.87 to 1.69), 0.26	
Interaction p-value=0.86				
Adjusted[b] OR (95% CI), p-value	1.31 (0.94 to 1.83), 0.17		1.25 (0.89 to 1.76), 0.19	
Interaction p-value=0.83				
**Maths**				
FNP	122 (36.2)	215 (63.8)	122 (40.3)	181 (59.7)
Usual care	107 (33.3)	214 (66.7)	120 (40.1)	179 (59.9)
Adjusted[a] OR (95% CI), p-value	0.89 (0.65 to 1.23), 0.48		0.99 (0.72 to 1.38), 0.97	
Interaction p-value=0.64				
Adjusted[b] OR (95% CI), p-value	0.93 (0.66 to 1.29), 0.65		1.03 (0.73 to 1.42), 0.90	
Interaction p-value=0.68				
**Science**				
FNP	87 (25.8)	250 (74.2)	821 (27.1)	221 (72.9)
Usual care	78 (24.3)	243 (75.7)	98 (32.8)	201 (67.2)
Adjusted[a] OR (95% CI), p-value	0.93 (0.65 to 1.33), 0.69		1.31 (0.92 to 1.87), 0.14	
Interaction p-value=0.20				
Adjusted[b] OR (95% CI), p-value	0.96 (0.67 to 1.39), 0.84		1.36 (0.95 to 1.95), 0.10	
Interaction p-value=0.22				

RES = Reaching the expected standard; [a] FNP compared to Usual care. Analysis adjusted for stratification (site), minimisation variables (gestational age, smoking status at recruitment, and first or preferred language); [b] Analysis adjusted for stratification (site), minimisation variables (gestational age, smoking status at recruitment, and first or preferred language) and month of birth. N/EET – Not/In Education, Employment or Training (Source: National Pupil Database, The Department for Education).

For peer review only - http://bmjopen.bmj.com/site/about/guidelines.xhtml

(e) Index of Multiple Deprivation quintiles

	N(%)	Q1=least deprived		Q2		Q3		Q4		Q5=most deprived	
		**Not RES**	**RES**	**Not RES**	**RES**	**Not RES**	**RES**	**Not RES**	**RES**	**Not RES**	**RES**
**Reading**	FNP	49 (30.3)	113 (67.7)	53 (36.3)	93 (63.7)	49 (33.6)	97 (66.4)	48 (37.2)	81 (62.8)	57 (37.8)	94 (62.2)
	Usual care	54 (38.9)	85 (61.1)	64 (44.1)	81 (55.9)	48 (31.8)	103 (68.2)	64 (43.0)	85 (57.0)	56 (39.4)	86 (60.6)
	Adjusted[a] OR (95% CI), p-value	1.57 (0.88 to 2.77), 0.12		1.42 (0.88 to 2.29), 0.16		0.96 (0.59 to 1.57), 0.88		1.33 (0.81 to 2.18), 0.25		1.07 (0.60 to 1.89), 0.83	
	Adjusted[b] OR (95% CI), p-value	1.75 (1.00 to 3.07), 0.05		1.42 (0.85 to 2.37), 0.18		0.89 (0.53 to 1.48), 0.64		1.41 (0.86 to 2.34), 0.18		1.00 (0.57 to 1.75), 0.99	
	Interaction p-value=0.70										
	Interaction p-value=0.37										
Maths	FNP	56 (34.6)	106 (65.4)	57 (39.0)	89 (61.0)	50 (34.3)	96 (65.7)	55 (42.6)	74 (57.4)	62 (41.1)	89 (58.9)
	Usual care	52 (37.4)	87 (62.6)	60 (41.4)	85 (58.6)	56 (37.1)	95 (62.9)	60 (40.3)	89 (59.7)	54 (38.0)	88 (62.0)
	Adjusted[a] OR (95% CI), p-value	1.15 (0.72 to 1.85), 0.56		1.12 (0.69 to 1.82), 0.65		1.17 (0.69 to 1.97), 0.56		0.93 (0.57 to 1.52), 0.77		0.91 (0.56 to 1.47), 0.69	
	Adjusted[b] OR (95% CI), p-value	1.36 (0.82 to 2.24), 0.23		1.12 (0.68 to 1.82), 0.66		1.11 (0.65 to 1.90), 0.69		0.98 (0.60 to 1.62), 0.95		0.88 (0.54 to 1.44), 0.62	
	Interaction p-value=0.91										
	Interaction p-value=0.81										

For peer review only - http://bmjopen.bmj.com/site/about/guidelines.xhtml

	N(%)	Q1=least deprived		Q2		Q3		Q4		Q5=most deprived	
		Not RES	RES	Not RES	RES	Not RES	RES	Not RES	RES	Not RES	RES
**Science**	FNP	37 (22.8)	125 (77.2)	36 (24.7)	110 (75.3)	40 (27.4)	106 (72.6)	45 (34.9)	84 (65.1)	44 (29.1)	107 (70.9)
	Usual care	45 (32.4)	94 (67.6)	45 (31.0)	100 (69.0)	35 (23.2)	116 (76.8)	47 (31.5)	102 (68.5)	46 (32.4)	96 (67.6)
	Adjusted[a] OR (95% CI), p-value	1.68 (1.00 to 2.81), 0.048		1.39 (0.80 to 2.41), 0.25		0.82 (0.46 to 1.47), 0.51		0.89 (0.52 to 1.51), 0.65		1.24 (0.74 to 2.06), 0.42	
	Adjusted[b] OR (95% CI), p-value	1.94 (1.13 to 3.30), 0.015		1.40 (0.76 to 2.57), 0.28		0.78 (0.43 to 1.40), 0.41		0.92 (0.55 to 1.55), 0.76		1.16 (0.69 to 1.96), 0.57	
	Interaction p-value=0.25										
	Interaction p-value=0.11										

RES = Reaching the expected standard; a FNP compared to Usual care; [a] Analysis adjusted for stratification (site), minimisation variables (gestational age, smoking status at recruitment, and first or preferred language); [b] Analysis adjusted for stratification (site), minimisation variables (gestational age, smoking status at recruitment, and first or preferred language) and month of birth. (Source: National Pupil Database, The Department for Education).

For peer review only - http://bmjopen.bmj.com/site/about/guidelines.xhtml

Table S17 Subgroup analyses for reaching the expected standard in the writing KS1 assessment for 2016/17 academic year

N(%)	FNP	Usual care	Adjusted[a] OR (95% CI), p-value	Interaction p-value	Adjusted[b] OR (95% CI), p-value	Interaction p-value
**Gender**						
Boys	109 (42.9)	83 (31.8)	1.61 (1.13 to 2.32), 0.009	0.034	1.62 (1.13 to 2.33), 0.009	0.039
Girls	132 (54.1)	126 (55.8)	1.03 (0.76 to 1.39), 0.87		0.94 (0.65 to 1.37), 0.76	
**Maternal age at recruitment**						
<16 years	18 (54.6)	9 (22.5)	4.67 (1.47 to 14.84), 0.009	0.016	5.28 (1.49 to 18.73), 0.010	0.018
≥16 years	223 (48.0)	200 (44.7)	1.13 (0.87 to 1.47), 0.35		1.15 (0.89 to 1.50), 0.29	
**Adaptive functioning**						
No difficulty	189 (52.5)	174 (46.7)	1.26 (0.93 to 1.70), 0.14	0.72	1.26 (0.93 to 1.70), 0.14	0.67
Difficulty in basic skills	52 (37.7)	34 (30.1)	1.41 (0.77 to 2.58), 0.26		1.53 (0.81 to 2.90), 0.19	
**NEET**						
EET (no not EET)	109 (47.8)	119 (52.2)	0.82 (0.56 to 1.19), 0.29	0.019	0.82 (0.56 to 1.19), 0.29	0.028
NEET	105 (49.1)	77 (38.3)	1.56 (1.05 to 2.30), 0.027		1.56 (1.05 to 2.30), 0.027	
**Deprivation quintile**						
1 – least deprived	63 (53.9)	39 (42.9)	1.65 (0.90 to 3.05), 0.11	0.89	1.83 (0.95 to 3.51), 0.07	0.86
2	48 (46.2)	46 (44.7)	1.00 (0.52 to 1.95), 0.99		1.04 (0.53 to 2.03), 0.92	
3	46 (50.6)	49 (46.7)	1.20 (0.67 to 2.15), 0.53		1.22 (0.66 to 2.26), 0.53	
4	40 (45.5)	35 (38.5)	1.28 (0.70 to 2.35), 0.42		1.28 (0.69 to 2.35), 0.43	
5 – most deprived	42 (44.2)	39 (41.1)	1.16 (0.64 to 2.08), 0.629		1.17 (0.64 to 2.14), 0.602	

For peer review only - http://bmjopen.bmj.com/site/about/guidelines.xhtml

[a] FNP compared to usual care. Analysis adjusted for stratification (site), minimisation variables (gestational age, smoking status at recruitment, and first or preferred language); [b] Analysis additionally adjusted for month of birth. N/EET – Not/In Education, Employment or Training (Source: National Pupil Database, The Department for Education).

For peer review only

Domestic abuse and CIN status:

Domestic abuse experienced by the mother was assessed using the Composite Abuse Scale (CAS) in the BB:0-2 trial at 24 months follow-up (with a score 1 or greater indicating some sort of abuse). The CAS could only be administered if the interview was face-to-face (as opposed to by telephone) and only if the participant was alone (N=607). In the BB:2-6 sample, this could be examined in 595 participants (FNP:322, Usual Care:273) and the proportion of participants experiencing some form of abuse was comparable to that found in the BB:0-2 trial (FNP: 122 (27.9%) vs UC:113 (41.4%)). There was also a relationship found between CAS and CIN status with 49 (20.9%) participants who experienced some abuse having a child being in need versus 42 (11.8%) of those who did not experience abuse. Domestic abuse at 24 months was to be explored as a possible effect mediator but due to lack of intervention effect in the child's CIN status, the need to examine the proportion of effect mediated by experience of domestic abuse at 24 months was redundant.

Care-experienced mothers and CIN status:

In addition, care-experienced mothers at time of recruitment to the BB:0-2 trial was examined. This was defined from the CLA dataset. Sixty-one mothers were identified as having experienced a period of care, four of whom were identified as care-experienced only after their BB:0-2 recruitment date. Fifty-seven mothers were examined for their duration of care and the women randomised to receive FNP experienced on average nine months more care than the Usual care arm (median (25th to 75th centile) FNP: 40 (15.5 to 113.0) UC: 31 (7.0 to 69.5) months). After merging these care-experienced mothers into the children's dataset, 56 mothers remained; seven mothers were excluded from the subgroup analyses since their child did not have any NPD data. Children of these care-experienced mothers were at a higher risk of being defined as in need themselves but no interaction was observed between trial arm and mothers care status. The duration of the maternal care status as a possible mediator effect was also stated in the statistical analysis plan but similarly to domestic abuse, due to lack of intervention effect in child's CIN status, then the need to examine the proportion of effect mediated by length of mother's care experience was redundant.

VIII. Treatment efficacy

The analyses of the following outcomes were re-run to investigate the effect of FNP dosage (number of visits over all delivery phases) using complier averaged causal effects modelling by fitting a structural mean model. The programme stated maximum number of visits over the whole period of FNP is 64 (14 in the pregnancy phase, 28 in the infancy phase, 22 in the toddler phase).

For peer review only - http://bmjopen.bmj.com/site/about/guidelines.xhtml

Referrals to CSC services: Adjusting the analysis for receipt of full dosage of FNP visits, made no difference to the conclusion of the main analysis for referral to social services (efficacy per visit odds ratio (OR)=1.00, 95% CI=0.99 to 1.004, p=0.613).

CIN status: Adjusting the analysis for receipt of FNP made no difference to the conclusion of the primary outcome (efficacy per visit OR=1.00, 95% CI=0.99 to 1.004, p=0.613).

Early Years assessment total point score: Adjusting the analysis for receipt of FNP we observe a 0.02 increase in point score in the between-arm mean difference in early years total score (as the number of visits increase so does the score) however the confidence interval around this estimate is wide (-0.004 to 0.040). The estimated treatment efficacy for participants receiving all 64 visits was 1.15 (95% CI -0.26 to 2.56), p=0.106 showing no change to the overall conclusions compared to the primary analysis.

KS1 attainment: Adjusting the analysis for FNP receipt we observe an increase in the odds of an event occurring in the FNP arm compared UC arm for every unit increase in FNP visit received. For all areas of learning (reading, writing, maths and science), adjusting the analysis for FNP receipt strengthens the effect of FNP, but it is the reading and writing assessment where given the full dosage of 64 FNP visits the odds make a significant difference. For reading assessments, the odds of reaching the expected standard increases from OR=1.23 (95% CI=0.99 to 1.53), p=0.051 in the main analysis to 1.38 (1.07 to 1.77), p=0.015. The efficacy per visit for reading was 1.005 (1.001 to 1.009), p=0.015; maths: 1.001 (0.997 to 1.005), p=0.535; science: 1.004 (0.999 to 1.008), p=0.112; writing: 1.013 (1.09 to 1.017), p=0.001.

IX. Exploration of surveillance bias upon Child In Need outcome assessment

Table S18 Baseline maternal characteristics, school readiness outcomes for children referred to CSC and classed as a Child In Need, before the age of 4 years old by intervention arm.

	**FNP**<br>**N=60**	**Usual Care**<br>**N=62**	**OR (95% CI)**
Maternal age (years) at recruitment<br>*Median (25th to 75th centiles)*	17.7 (16.9 to 18.3)	17.8 (16.7 to 18.7)	0.97 (0.73 to 1.27)
Not in Education, Employment or Training at recruitment *N(%)*			
Education, Employment or Training	21 (42.0)	11 (22.0)	
Not in Education, Employment or Training	30 (58.0)	39 (78.0)	2.45 (1.04 to 5.93)
Not applicable (<16 years at interview)/ missing	9	12	

For peer review only - http://bmjopen.bmj.com/site/about/guidelines.xhtml

Ethnicity *N(%)*	Numbers too small		
Deprivation score[1] *Median (25th to 75th centiles)*	43.0 (25.0 to 56.1)	45.0 (31.8 to 62.7)	0.99 (0.98 to 1.01)
Adaptive functioning			
Had 3 or less life skills			
No	38 (63.3)	43 (69.4)	
Yes	22 (36.7)	19 (30.6)	1.41 (0.67 to 2.98)
Difficulty in at least one basic skills *N(%)*			
No	37 (61.7)	43 (69.4)	
Yes	23 (38.3)	19 (30.6)	1.31 (0.62 to 2.8)
At least one burden *N(%)*			
No	39 (65.0)	42 (68.9)	
Yes	21 (35.0)	19 (31.1)	1.19 (0.56 to 2.54)
Social support			
Maximum social support N(%) (score=100)	10 (16.9)	9 (14.5)	1.20 (0.45 to 3.21)
Relationship quality *(score 7 to 35)*[2]	28 (25 to 30)	30 (25 to 32)	0.97 (0.89 to 1.05)
Self-efficacy *(score 10 to 40)*[3] *Median (25th to 75th centiles)*	30 (28 to 32)	30 (27.25 to 31)	1.05 (0.97 to 1.15)
Family resource *(score 4 to 20)*[4] *Mean (SD)*	12.6 (4.2)	12.7 (3.9)	0.91 (0.64 to 1.30)
CRAFFT *(score 0 to 6)*[5] *Median (25th to 75th centiles)*	1 (0 to 2)	1 (0 to 2)	1.07 (0.85 to 1.35)
**Early Years**			
GLD in all 5 areas of learning *N(%)*	26 (44.1)	22 (37.9)	1.29 (0.62 to 2.70)
GLD in all 17 early learning goals *N(%)*	26 (44.1)	20 (34.5)	1.50 (0.71 to 3.16)
Total point score over all 17 early learning goals *Median (25th to 75th centiles)*	32 (22 to 34)	30.5 (21 to 34)	Mean difference = 1.66 (-1.14 to 4.45)

[1] Higher score indicates more deprived, [2] Higher score indicates a better relationship quality, [3] Higher score indicates higher level of self-efficacy, [4] Higher score indicates higher level of family resource, [5] Greater risk of problems related to drugs and alcohol

For peer review only

X. Missing data

Since only a small number (n=40 (2.7%)) of children were not linked to any NPD dataset and not included in the primary outcome analyses, no multiple imputation was carried out.

XI. Cost Consequence Analysis

Table S19 Cost-consequence balance sheet for the family-nurse based intervention for the four years between children's ages 2 and 6.

**Mothers**	Mean resource use per participant			Mean cost per participant (£)		
	FNP	Usual Care	Incremental (FNP-Usual Care)	FNP	Usual Care	Incremental (FNP-Usual Care)
**Health related resource use**						
Inpatient Attendances						
Overnight admissions (N)	3.04	2.99	0.05			
Overnight length of stay (N nights)	2.32	2.32	0.00	2,826.79	2,844.58	-17.79
Day Admittances	2.93	2.91	0.02	1,874.61	1,890.11	-15.50
Outpatient Attendances						
All Outpatient Attendances	23.29	22.61	0.68	2,244.62	2,189.32	55.30
Accident and Emergency						
A&E Visits (N)	5.19	4.93	0.26	411.34	392.03	19.31
Total Resource Use						
				7,357.36	7,316.04	41.32 p=0.393

**Children**	Mean resource use per participant			Mean cost per participant (£)		
	FNP	Usual Care	Incremental (FNP-Usual Care)	FNP	Usual Care	Incremental (FNP-Usual Care)
**Health related resource use**						
Inpatient Attendances - All						
Overnight admissions (N)	1.57	1.55	0.02			
Overnight length of stay (N nights)	3.57	3.39	0.18	1,962.95	2,033.71	-70.76
Day Admittances	0.98	0.97	0.01	744.94	724.90	20.04
Inpatient Attendances – All (Excluding Injuries and Ingestions)						
Overnight admissions (N)	1.51	1.49	0.02	1,883.44	1,951.15	-67.71
Overnight length of stay (N nights)	3.61	3.44	0.17			
Day Admittances	0.90	0.88	0.02	689.22	667.09	22.13

For peer review only

For peer review only - http://bmjopen.bmj.com/site/about/guidelines.xhtml

Inpatient Attendances – Injuries and Ingestions Only						
Overnight admissions (N)	0.06	0.06	0.00			
Overnight length of stay (N nights)	2.50	2.05	0.45	79.51	82.55	-3.04
Day Admittances	0.08	0.09	-0.01	55.72	57.81	-2.09
Outpatient Attendances						
All Outpatient Attendances	6.89	7.44	-0.55	673.24	727.79	-54.55
Accident and Emergency - All						
A&E Visits (N)	4.93	4.62	0.31	395.73	366.83	28.90
Accident and Emergency - All (Excluding Injuries and Ingestions)						
A&E Visits (N)	3.80	3.52	0.28	305.12	280.20	24.92
Accident and Emergency – Injuries and Ingestions Only						
A&E Visits (N)	1.12	1.10	0.02	75.09	69.91	5.18
Total Resource Use						
				3,778.13	3,853.22	-75.09 p=0.865
**Consequences**						
Child in Need status recorded at any time during the follow-up period.				OR: 0.98 (95%CI: 0.74 to 1.31) p=0.902		
Referral to Social Services				OR: 0.97 (0.74 to 1.28) p=0.829		
Child Protection registration (CPP)				OR: 1.04 (0.69 to 1.57) p=0.846		
Details of a CPP						
• Neglect				Reference		
• Emotional				OR: 2.77 (1.02 to 7.56) p=0.046		
• Physical				OR: 1.25 (0.32 to 4.88) p=0.743		
• Sexual and multiple				OR: 1.13 (0.33 to 3.86) p=0.848		
CIN categorisation						
• Abuse or Neglect				Reference		
• Family dysfunction				OR: 0.95 (0.54 to 1.69) p=0.868		
• Family in acute stress				OR: 1.47 (0.61 to 3.54) p=0.389		
• Low income, absent parenting, socially unacceptable behaviour				OR: 2.38 (0.86 to 6.57) p=0.093		
• Child/parent disability				OR: 1.47 (0.45 to 4.84) p=0.525		
Looked after status				OR: 0.90 (0.52 to 1.57) p=0.712		
Child looked after period of care (months)				OR: 0.75 (0.65 to 0.86) p<0.001		
DNA appointments						
• DNA at least once				OR: 1.00 (0.79 to 1.26) p=0.997		
Injuries and ingestions						
• Emergency attendance						
○ No				Reference		

For peer review only

For peer review only - http://bmjopen.bmj.com/site/about/guidelines.xhtml

○ Yes	OR: 1.17 (0.95 to 1.45) p=0.149
• Admission to hospital	
○ No	Reference
○ Yes	OR: 0.87 (0.63 to 1.20) p=0.407
• Either hospital attendance and/or admission to hospital	
○ No	Reference
○ Yes	OR: 1.11 (0.89 to 1.37) p=0.351
Special Educational Needs	OR: 0.83 (0.67 to 1.03) p=0.097
Early Years assessment	
• Achieving good level of development in all five areas of learning	OR: 1.26 (1.03 to 1.55) p=0.026
• Achieving good level of development in all 17 early learning goals	OR: 1.24 (1.01 to 1.52) p=0.043
School attendance	
• Overall absences	
○ No absences	Reference
○ At least one absence	OR: 1.00 (0.47 to 2.12) p=0.998
• Overall unauthorised absences	
○ No absences	Reference
○ At least one absence	OR: 0.95 (0.76 to 1.18) p=0.620
• Overall authorised absences	
○ No absences	Reference
○ At least one absence	OR: 1.01 (0.58 to 1.75) p=0.984
KS1 attainment	
• Reading	
○ Lower than expected	Reference
○ Reaching the expected standard	OR: 1.23 (0.99 to 1.53) p=0.051
• Writing academic year 2016/17	
○ Lower than expected	Reference
○ Reaching the expected standard	OR: 1.24 (0.97 to 1.60) p=0.090
• Maths	
○ Lower than expected	Reference
○ Reaching the expected standard	OR: 1.04 (0.84 to 1.28) p=0.731
• Science	
○ Lower than expected	Reference
○ Reaching the expected standard	OR: 1.14 (0.91 to 1.43) p=0.254

For peer review only - http://bmjopen.bmj.com/site/about/guidelines.xhtml

XII. Postpartum resource use data collected for SPHN contacts

The 6-, 12- and 18-months telephone interview CRF included the following items regarding SPHN (ie health visitor) contacts:

6-month interview question:

Since your baby(ies) was born can I ask how many times you have seen any of the following people?

Note to interviewer: For each positive question ask & record

1. How many times do you think you have seen them in that time?
2. Where did you see them (e.g. hospital, clinic, home)?

	Number of home visits	Number of clinic visits
Health visitor		

12-month interview question:

In the last 6 months can I ask how many times you have seen any of the following people?

Note to interviewer: For each positive question ask & record

1. How many times do you think you have seen them in that time?
2. Where did you see them (e.g. hospital, clinic, home)?

	Number of home visits	Number of clinic visits
Health visitor		

18-month interview question:

In the last 6 months can I ask how many times you have seen any of the following people?

Note to interviewer: For each positive question ask & record

1. How many times do you think you have seen them in that time?
2. Where did you see them (e.g. hospital, clinic, home)?

	Number of home visits	Number of clinic visits
Health visitor		

For peer review only - http://bmjopen.bmj.com/site/about/guidelines.xhtml

The RECORD statement – checklist of items, extended from the STROBE statement, that should be reported in observational studies using routinely collected health data.

	Item No.	STROBE items	Location in manuscript where items are reported	RECORD items	Location in manuscript where items are reported
**Title and abstract**					
	1	(a) Indicate the study's design with a commonly used term in the title or the abstract (b) Provide in the abstract an informative and balanced summary of what was done and what was found	1a/b Abstract	RECORD 1.1: The type of data used should be specified in the title or abstract. When possible, the name of the databases used should be included.<br><br>RECORD 1.2: If applicable, the geographic region and timeframe within which the study took place should be reported in the title or abstract.<br><br>RECORD 1.3: If linkage between databases was conducted for the study, this should be clearly stated in the title or abstract.	1.1 Abstract<br>1.2 Abstract<br>1.3 Abstract
**Introduction**					
Background rationale	2	Explain the scientific background and rationale for the investigation being reported	Introduction		
Objectives	3	State specific objectives, including any prespecified hypotheses	Introduction > Objectives		
**Methods**					
Study Design	4	Present key elements of study design early in the paper	Methods> Study design, setting and participants		
Setting	5	Describe the setting, locations, and relevant dates, including periods of recruitment, exposure, follow-up, and data collection	Methods>Procedure and data sources		

For peer review only

Participants	6	*(a) Cohort study* - Give the eligibility criteria, and the sources and methods of selection of participants. Describe methods of follow-up<br>*Case-control study* - Give the eligibility criteria, and the sources and methods of case ascertainment and control selection. Give the rationale for the choice of cases and controls<br>*Cross-sectional study* - Give the eligibility criteria, and the sources and methods of selection of participants<br><br>*(b) Cohort study* - For matched studies, give matching criteria and number of exposed and unexposed<br>*Case-control study* - For matched studies, give matching criteria and the number of controls per case	6a Methods> Study design, setting and participants<br><br>6b NA	RECORD 6.1: The methods of study population selection (such as codes or algorithms used to identify subjects) should be listed in detail. If this is not possible, an explanation should be provided.<br><br>RECORD 6.2: Any validation studies of the codes or algorithms used to select the population should be referenced. If validation was conducted for this study and not published elsewhere, detailed methods and results should be provided.<br><br>RECORD 6.3: If the study involved linkage of databases, consider use of a flow diagram or other graphical display to demonstrate the data linkage process, including the number of individuals with linked data at each stage.	6.1 Methods> Study design, setting and participants<br><br>6.2 NA<br><br>6.3 Protocol paper cited, Figure 1
Variables	7	Clearly define all outcomes, exposures, predictors, potential confounders, and effect modifiers. Give diagnostic criteria, if applicable.	Methods> Study design, setting and participants, Outcomes; Appendix	RECORD 7.1: A complete list of codes and algorithms used to classify exposures, outcomes, confounders, and effect modifiers should be provided. If these cannot be reported, an explanation should be provided.	Methods> Study design, setting and participants, Outcomes
Data sources/ measurement	8	For each variable of interest, give sources of data and details of methods of assessment (measurement).<br>Describe comparability of assessment methods if there is more than one group	Methods> Study design, setting and participants, Outcomes; Appendix		

For peer review only

Bias	9	Describe any efforts to address potential sources of bias	Methods> Efforts to address sources of bias		
Study size	10	Explain how the study size was arrived at	Methods> Power calculation		
Quantitative variables	11	Explain how quantitative variables were handled in the analyses. If applicable, describe which groupings were chosen, and why	Methods>Statistical analysis		
Statistical methods	12	(a) Describe all statistical methods, including those used to control for confounding<br>(b) Describe any methods used to examine subgroups and interactions<br>(c) Explain how missing data were addressed<br>(d) *Cohort study* - If applicable, explain how loss to follow-up was addressed<br>*Case-control study* - If applicable, explain how matching of cases and controls was addressed<br>*Cross-sectional study* - If applicable, describe analytical methods taking account of sampling strategy<br>(e) Describe any sensitivity analyses	12a-c, e: Methods>Statistical analysis; Economic analysis<br>12d: NA		
Data access and cleaning methods		..		RECORD 12.1: Authors should describe the extent to which the investigators had access to the database population used to create the study population.	Methods> Study design, setting and participants, Outcomes; Appendix

For peer review only

				RECORD 12.2: Authors should provide information on the data cleaning methods used in the study.	
Linkage		..		RECORD 12.3: State whether the study included person-level, institutional-level, or other data linkage across two or more databases. The methods of linkage and methods of linkage quality evaluation should be provided.	Methods> Procedure and data sources
**Results**					
Participants	13	(a) Report the numbers of individuals at each stage of the study (*e.g.*, numbers potentially eligible, examined for eligibility, confirmed eligible, included in the study, completing follow-up, and analysed)<br>(b) Give reasons for non-participation at each stage.<br>(c) Consider use of a flow diagram	Table S3, Figure 1	RECORD 13.1: Describe in detail the selection of the persons included in the study (*i.e.,* study population selection) including filtering based on data quality, data availability and linkage. The selection of included persons can be described in the text and/or by means of the study flow diagram.	Figure 1
Descriptive data	14	(a) Give characteristics of study participants (*e.g.*, demographic, clinical, social) and information on exposures and potential confounders<br>(b) Indicate the number of participants with missing data for each variable of interest<br>(c) *Cohort study* - summarise follow-up time (*e.g.*, average and total amount)	14a: Table 1<br>14b: Table S3, Tables 2-4		
Outcome data	15	*Cohort study* - Report numbers of outcome events or summary measures over time<br>*Case-control study* - Report numbers in each exposure	Tables 2-4		

For peer review only

		category, or summary measures of exposure<br>*Cross-sectional study* - Report numbers of outcome events or summary measures			
Main results	16	(a) Give unadjusted estimates and, if applicable, confounder-adjusted estimates and their precision (e.g., 95% confidence interval). Make clear which confounders were adjusted for and why they were included<br>(b) Report category boundaries when continuous variables were categorized<br>(c) If relevant, consider translating estimates of relative risk into absolute risk for a meaningful time period	Tables 2-4		
Other analyses	17	Report other analyses done—e.g., analyses of subgroups and interactions, and sensitivity analyses	Appendix		
**Discussion**					
Key results	18	Summarise key results with reference to study objectives	Discussion> Principal findings		
Limitations	19	Discuss limitations of the study, taking into account sources of potential bias or imprecision. Discuss both direction and magnitude of any potential bias		RECORD 19.1: Discuss the implications of using data that were not created or collected to answer the specific research question(s). Include discussion of misclassification bias, unmeasured confounding, missing data, and changing eligibility over time, as they pertain to the study being reported.	Discussion> Strengths and weaknesses of this study, Strengths and weaknesses in comparison to other studies
Interpretation	20	Give a cautious overall interpretation of results considering objectives,			Discussion> Strengths and weaknesses of this study, Strengths

		limitations, multiplicity of analyses, results from similar studies, and other relevant evidence			and weaknesses in comparison to other studies
Generalisability	21	Discuss the generalisability (external validity) of the study results			Discussion> Strengths and weaknesses in comparison to other studies
**Other Information**					
Funding	22	Give the source of funding and the role of the funders for the present study and, if applicable, for the original study on which the present article is based			Described in Funding section
Accessibility of protocol, raw data, and programming code		..		RECORD 22.1: Authors should provide information on how to access any supplemental information such as the study protocol, raw data, or programming code.	Protocol paper cited in references; access to data described in Data sharing statement

*Reference: Benchimol EI, Smeeth L, Guttmann A, Harron K, Moher D, Petersen I, Sørensen HT, von Elm E, Langan SM, the RECORD Working Committee. The REporting of studies Conducted using Observational Routinely-collected health Data (RECORD) Statement. *PLoS Medicine* 2015; in press.

*Checklist is protected under Creative Commons Attribution (CC BY) license.

For peer review only
